# Supplementary material for: Cretaceous dinosaur bone contains recent organic material and provides an environment conducive to microbial communities
Source: eLife. 2019 Jun 18;8:e46205. doi: 10.7554/eLife.46205 (PMC6581507; doi:10.7554/eLife.46205)
Supplement: Source data 1. [file elife-46205-data1.zip › Raw data files/ReadMe.pdf]

The raw data is associated with the following publication:

Saitta, ET, Liang, R, Lau, MCY, Brown, CM, Longrich, NR, Kaye, TG, Novak, BJ, Salzberg, SL, Norell, MA, Abbott, GD, Dickinson, MR, Vinther, J, Bull, ID, Brooker, RA, Martin, P, Donohoe, P, Knowles, TDJ, Penkman, KEH, and Onstott, T (2019) Cretaceous dinosaur bone contains recent organic material and provides an environment conducive to microbial communities. *eLife* 8, e46205. https://doi.org/10.7554/eLife.46205.001

Uploaded onto the EMu repository is the zipped folder "Rawdatafiles.zip" containing four sub-folders:

1) Folder "16S rRNA amplicon sequencing" contains supplemental .TXT, .BIOM, .BIOM.GZ, .HTML, .PNG, .CSS, .JS, .ICO, .PDF files associated with the 16S rRNA amplicon sequencing analysis. The associated software is QIIME (Quantitative Insights Into Microbial Ecology).

Note that in addition to these supplemental files in the folder "16S rRNA amplicon sequencing", the raw 16S amplicon sequencing data were deposited in the Sequence Read Archive (SRA) of NCBI under the accession number of SRR7947417.

2) Folder "ATR FTIR" contains .CSV files associated with the attenuated total reflectance Fourier-transform infrared spectroscopy.

3) Folder "EDS" contains .CSV files associated with the energy dispersive X-ray spectroscopy.

4) Folder "Py-GC-MS" contains .CDF and .RAW files associated with the pyrolysis-gas chromatography-mass spectrometry. The associated software is Xcalibur, but other software options are available.
