# Supplementary material for: Cretaceous dinosaur bone contains recent organic material and provides an environment conducive to microbial communities
Source: eLife. 2019 Jun 18;8:e46205. doi: 10.7554/eLife.46205 (PMC6581507; doi:10.7554/eLife.46205)
Supplement: Source data 1. [file elife-46205-data1.zip › Raw data files/16S rRNA amplicon sequencing/index.html]

QIIME results


|  |  |
| --- | --- |
| Run summary data | |
| Master run log | log\_20170820202628.txt |
| BIOM table statistics | biom\_table\_summary.txt |
| Filtered BIOM table (minimum sequence count: 620000) | table\_mc620000.biom.gz |
| rarefied BIOM table (sampling depth: 620000) | table\_even620000.biom.gz |
| Taxonomic summary results | |
| Taxa summary bar plots | bar\_charts.html |
| Taxa summary area plots | area\_charts.html |
| Alpha diversity results | |
| Alpha rarefaction plots | rarefaction\_plots.html |
| Beta diversity results (even sampling: 620000) | |
| PCoA plot (weighted\_unifrac) | index.html |
| Distance matrix (weighted\_unifrac) | weighted\_unifrac\_dm.txt |
| Principal coordinate matrix (weighted\_unifrac) | weighted\_unifrac\_pc.txt |
| PCoA plot (unweighted\_unifrac) | index.html |
| Distance matrix (unweighted\_unifrac) | unweighted\_unifrac\_dm.txt |
| Principal coordinate matrix (unweighted\_unifrac) | unweighted\_unifrac\_pc.txt |

**Need help?** See http://help.qiime.org.
