# Supplementary material for: Cretaceous dinosaur bone contains recent organic material and provides an environment conducive to microbial communities
Source: eLife. 2019 Jun 18;8:e46205. doi: 10.7554/eLife.46205 (PMC6581507; doi:10.7554/eLife.46205)
Supplement: Source data 1. [file elife-46205-data1.zip › Raw data files/16S rRNA amplicon sequencing/taxa_plots/taxa_summary_plots/bar_charts.html]

Taxa Summaries


|  |  |
| --- | --- |
|  | |
| Taxonomy Summary. Current Level: | |
| View Figure (.pdf)  View Legend (.pdf) |  |
|  |


|  |
| --- |
| View Table (.txt) |

|  |  |  |  |  |  |  |  |  |  |  |
| --- | --- | --- | --- | --- | --- | --- | --- | --- | --- | --- |
|  | | Total | 1B5g1 | 1B5g2 | 1BEDTA1 | 1BEDTA2 | 1M1 | 1M2 | 1S1 | 1S2 |
| Legend | Taxonomy | % | % | % | % | % | % | % | % | % |
|  | D\_0\_\_Archaea;D\_1\_\_Thaumarchaeota | 0.6% | 0.7% | 0.7% | 0.4% | 0.5% | 1.0% | 0.8% | 0.2% | 0.3% |
|  | D\_0\_\_Bacteria;D\_1\_\_Acidobacteria | 2.9% | 4.0% | 3.4% | 3.5% | 3.5% | 3.2% | 1.2% | 2.6% | 1.6% |
|  | D\_0\_\_Bacteria;D\_1\_\_Actinobacteria | 80.4% | 71.1% | 73.6% | 73.7% | 73.1% | 83.3% | 91.4% | 92.1% | 84.9% |
|  | D\_0\_\_Bacteria;D\_1\_\_Armatimonadetes | 0.0% | 0.0% | 0.0% | 0.0% | 0.0% | 0.0% | 0.0% | 0.0% | 0.0% |
|  | D\_0\_\_Bacteria;D\_1\_\_BRC1 | 0.0% | 0.0% | 0.0% | 0.0% | 0.0% | 0.0% | 0.0% | 0.0% | 0.0% |
|  | D\_0\_\_Bacteria;D\_1\_\_Bacteroidetes | 0.2% | 0.1% | 0.1% | 0.2% | 0.2% | 0.1% | 0.1% | 0.2% | 0.8% |
|  | D\_0\_\_Bacteria;D\_1\_\_Chlamydiae | 0.0% | 0.0% | 0.0% | 0.0% | 0.0% | 0.0% | 0.0% | 0.0% | 0.0% |
|  | D\_0\_\_Bacteria;D\_1\_\_Chlorobi | 0.0% | 0.0% | 0.0% | 0.0% | 0.0% | 0.0% | 0.0% | 0.0% | 0.0% |
|  | D\_0\_\_Bacteria;D\_1\_\_Chloroflexi | 2.7% | 4.1% | 3.8% | 3.4% | 3.3% | 2.7% | 0.7% | 1.7% | 1.8% |
|  | D\_0\_\_Bacteria;D\_1\_\_Cyanobacteria | 0.0% | 0.0% | 0.0% | 0.0% | 0.0% | 0.0% | 0.0% | 0.0% | 0.0% |
|  | D\_0\_\_Bacteria;D\_1\_\_Deinococcus-Thermus | 0.0% | 0.0% | 0.0% | 0.0% | 0.0% | 0.0% | 0.0% | 0.0% | 0.1% |
|  | D\_0\_\_Bacteria;D\_1\_\_Elusimicrobia | 0.0% | 0.0% | 0.0% | 0.0% | 0.0% | 0.0% | 0.0% | 0.0% | 0.0% |
|  | D\_0\_\_Bacteria;D\_1\_\_FBP | 0.0% | 0.0% | 0.0% | 0.0% | 0.0% | 0.0% | 0.0% | 0.0% | 0.1% |
|  | D\_0\_\_Bacteria;D\_1\_\_Fibrobacteres | 0.0% | 0.0% | 0.0% | 0.0% | 0.0% | 0.0% | 0.0% | 0.0% | 0.0% |
|  | D\_0\_\_Bacteria;D\_1\_\_Firmicutes | 0.3% | 0.0% | 0.0% | 0.1% | 0.1% | 0.1% | 0.0% | 0.3% | 1.7% |
|  | D\_0\_\_Bacteria;D\_1\_\_Fusobacteria | 0.1% | 0.0% | 0.0% | 0.0% | 0.0% | 0.0% | 0.0% | 0.1% | 0.5% |
|  | D\_0\_\_Bacteria;D\_1\_\_Gemmatimonadetes | 0.3% | 0.3% | 0.3% | 0.2% | 0.2% | 0.8% | 0.2% | 0.2% | 0.1% |
|  | D\_0\_\_Bacteria;D\_1\_\_Hydrogenedentes | 0.0% | 0.0% | 0.0% | 0.0% | 0.0% | 0.0% | 0.0% | 0.0% | 0.0% |
|  | D\_0\_\_Bacteria;D\_1\_\_Nitrospirae | 0.2% | 0.5% | 0.4% | 0.2% | 0.2% | 0.3% | 0.1% | 0.1% | 0.2% |
|  | D\_0\_\_Bacteria;D\_1\_\_Planctomycetes | 0.1% | 0.1% | 0.1% | 0.2% | 0.2% | 0.0% | 0.0% | 0.1% | 0.1% |
|  | D\_0\_\_Bacteria;D\_1\_\_Proteobacteria | 11.8% | 18.6% | 17.2% | 17.6% | 18.3% | 8.0% | 5.4% | 2.2% | 7.3% |
|  | D\_0\_\_Bacteria;D\_1\_\_SBR1093 | 0.0% | 0.0% | 0.0% | 0.0% | 0.0% | 0.0% | 0.0% | 0.0% | 0.0% |
|  | D\_0\_\_Bacteria;D\_1\_\_SR1 (Absconditabacteria) | 0.0% | 0.0% | 0.0% | 0.0% | 0.0% | 0.0% | 0.0% | 0.0% | 0.1% |
|  | D\_0\_\_Bacteria;D\_1\_\_Saccharibacteria | 0.0% | 0.0% | 0.0% | 0.0% | 0.0% | 0.0% | 0.0% | 0.0% | 0.0% |
|  | D\_0\_\_Bacteria;D\_1\_\_Spirochaetae | 0.0% | 0.0% | 0.0% | 0.0% | 0.0% | 0.0% | 0.0% | 0.0% | 0.1% |
|  | D\_0\_\_Bacteria;D\_1\_\_Synergistetes | 0.0% | 0.0% | 0.0% | 0.0% | 0.0% | 0.0% | 0.0% | 0.0% | 0.0% |
|  | D\_0\_\_Bacteria;D\_1\_\_TM6 (Dependentiae) | 0.0% | 0.0% | 0.0% | 0.0% | 0.0% | 0.0% | 0.0% | 0.0% | 0.0% |
|  | D\_0\_\_Bacteria;D\_1\_\_Tectomicrobia | 0.0% | 0.0% | 0.0% | 0.0% | 0.0% | 0.0% | 0.0% | 0.0% | 0.0% |
|  | D\_0\_\_Bacteria;D\_1\_\_Verrucomicrobia | 0.0% | 0.0% | 0.0% | 0.0% | 0.0% | 0.0% | 0.0% | 0.0% | 0.0% |
|  | D\_0\_\_Bacteria;Other | 0.0% | 0.0% | 0.0% | 0.0% | 0.0% | 0.0% | 0.0% | 0.0% | 0.0% |
|  | Unassigned;Other | 0.3% | 0.4% | 0.4% | 0.3% | 0.3% | 0.4% | 0.1% | 0.2% | 0.2% |

|  |  |
| --- | --- |
|  | |
| Taxonomy Summary. Current Level: | |
| View Figure (.pdf)  View Legend (.pdf) |  |
|  |


|  |
| --- |
| View Table (.txt) |

|  |  |  |  |  |  |  |  |  |  |  |
| --- | --- | --- | --- | --- | --- | --- | --- | --- | --- | --- |
|  | | Total | 1B5g1 | 1B5g2 | 1BEDTA1 | 1BEDTA2 | 1M1 | 1M2 | 1S1 | 1S2 |
| Legend | Taxonomy | % | % | % | % | % | % | % | % | % |
|  | D\_0\_\_Archaea;D\_1\_\_Thaumarchaeota;D\_2\_\_Soil Crenarchaeotic Group(SCG) | 0.6% | 0.7% | 0.7% | 0.4% | 0.5% | 1.0% | 0.8% | 0.2% | 0.3% |
|  | D\_0\_\_Bacteria;D\_1\_\_Acidobacteria;D\_2\_\_Blastocatellia | 0.0% | 0.0% | 0.0% | 0.0% | 0.0% | 0.0% | 0.0% | 0.0% | 0.0% |
|  | D\_0\_\_Bacteria;D\_1\_\_Acidobacteria;D\_2\_\_Holophagae | 0.0% | 0.0% | 0.0% | 0.0% | 0.0% | 0.1% | 0.0% | 0.0% | 0.0% |
|  | D\_0\_\_Bacteria;D\_1\_\_Acidobacteria;D\_2\_\_Solibacteres | 0.0% | 0.0% | 0.0% | 0.0% | 0.0% | 0.0% | 0.0% | 0.0% | 0.0% |
|  | D\_0\_\_Bacteria;D\_1\_\_Acidobacteria;D\_2\_\_Subgroup 17 | 2.5% | 3.3% | 2.9% | 3.0% | 3.0% | 2.8% | 1.1% | 2.5% | 1.6% |
|  | D\_0\_\_Bacteria;D\_1\_\_Acidobacteria;D\_2\_\_Subgroup 22 | 0.0% | 0.0% | 0.0% | 0.0% | 0.0% | 0.0% | 0.0% | 0.0% | 0.0% |
|  | D\_0\_\_Bacteria;D\_1\_\_Acidobacteria;D\_2\_\_Subgroup 5 | 0.0% | 0.0% | 0.0% | 0.0% | 0.0% | 0.0% | 0.0% | 0.0% | 0.0% |
|  | D\_0\_\_Bacteria;D\_1\_\_Acidobacteria;D\_2\_\_Subgroup 6 | 0.3% | 0.6% | 0.4% | 0.4% | 0.4% | 0.2% | 0.1% | 0.1% | 0.0% |
|  | D\_0\_\_Bacteria;D\_1\_\_Acidobacteria;Other | 0.0% | 0.0% | 0.0% | 0.0% | 0.0% | 0.0% | 0.0% | 0.0% | 0.0% |
|  | D\_0\_\_Bacteria;D\_1\_\_Actinobacteria;D\_2\_\_Acidimicrobiia | 3.5% | 6.1% | 6.3% | 4.4% | 4.8% | 3.0% | 1.0% | 1.4% | 1.0% |
|  | D\_0\_\_Bacteria;D\_1\_\_Actinobacteria;D\_2\_\_Actinobacteria | 58.1% | 32.1% | 34.4% | 35.7% | 35.4% | 74.5% | 86.4% | 86.5% | 79.8% |
|  | D\_0\_\_Bacteria;D\_1\_\_Actinobacteria;D\_2\_\_Coriobacteriia | 0.0% | 0.0% | 0.0% | 0.0% | 0.0% | 0.0% | 0.0% | 0.0% | 0.0% |
|  | D\_0\_\_Bacteria;D\_1\_\_Actinobacteria;D\_2\_\_MB-A2-108 | 0.0% | 0.0% | 0.0% | 0.0% | 0.0% | 0.0% | 0.0% | 0.0% | 0.0% |
|  | D\_0\_\_Bacteria;D\_1\_\_Actinobacteria;D\_2\_\_Nitriliruptoria | 15.3% | 27.5% | 28.1% | 29.1% | 28.6% | 2.3% | 2.6% | 2.4% | 2.1% |
|  | D\_0\_\_Bacteria;D\_1\_\_Actinobacteria;D\_2\_\_OPB41 | 0.0% | 0.0% | 0.0% | 0.0% | 0.0% | 0.0% | 0.0% | 0.0% | 0.0% |
|  | D\_0\_\_Bacteria;D\_1\_\_Actinobacteria;D\_2\_\_Rubrobacteria | 0.0% | 0.0% | 0.0% | 0.0% | 0.0% | 0.0% | 0.0% | 0.0% | 0.1% |
|  | D\_0\_\_Bacteria;D\_1\_\_Actinobacteria;D\_2\_\_TakashiAC-B11 | 2.3% | 3.9% | 3.6% | 3.5% | 3.2% | 1.4% | 0.2% | 1.3% | 1.1% |
|  | D\_0\_\_Bacteria;D\_1\_\_Actinobacteria;D\_2\_\_Thermoleophilia | 1.2% | 1.5% | 1.3% | 1.0% | 1.0% | 2.0% | 1.3% | 0.5% | 0.8% |
|  | D\_0\_\_Bacteria;D\_1\_\_Actinobacteria;Other | 0.0% | 0.0% | 0.0% | 0.0% | 0.0% | 0.0% | 0.0% | 0.0% | 0.0% |
|  | D\_0\_\_Bacteria;D\_1\_\_Armatimonadetes;D\_2\_\_Armatimonadia | 0.0% | 0.0% | 0.0% | 0.0% | 0.0% | 0.0% | 0.0% | 0.0% | 0.0% |
|  | D\_0\_\_Bacteria;D\_1\_\_Armatimonadetes;D\_2\_\_Fimbriimonadia | 0.0% | 0.0% | 0.0% | 0.0% | 0.0% | 0.0% | 0.0% | 0.0% | 0.0% |
|  | D\_0\_\_Bacteria;D\_1\_\_Armatimonadetes;D\_2\_\_uncultured | 0.0% | 0.0% | 0.0% | 0.0% | 0.0% | 0.0% | 0.0% | 0.0% | 0.0% |
|  | D\_0\_\_Bacteria;D\_1\_\_BRC1;D\_2\_\_uncultured bacterium | 0.0% | 0.0% | 0.0% | 0.0% | 0.0% | 0.0% | 0.0% | 0.0% | 0.0% |
|  | D\_0\_\_Bacteria;D\_1\_\_BRC1;Other | 0.0% | 0.0% | 0.0% | 0.0% | 0.0% | 0.0% | 0.0% | 0.0% | 0.0% |
|  | D\_0\_\_Bacteria;D\_1\_\_Bacteroidetes;D\_2\_\_Bacteroidetes BD2-2 | 0.0% | 0.0% | 0.0% | 0.0% | 0.0% | 0.0% | 0.0% | 0.0% | 0.0% |
|  | D\_0\_\_Bacteria;D\_1\_\_Bacteroidetes;D\_2\_\_Bacteroidetes Incertae Sedis | 0.0% | 0.0% | 0.0% | 0.0% | 0.0% | 0.0% | 0.0% | 0.0% | 0.0% |
|  | D\_0\_\_Bacteria;D\_1\_\_Bacteroidetes;D\_2\_\_Bacteroidia | 0.1% | 0.0% | 0.0% | 0.0% | 0.0% | 0.0% | 0.0% | 0.1% | 0.5% |
|  | D\_0\_\_Bacteria;D\_1\_\_Bacteroidetes;D\_2\_\_Cytophagia | 0.1% | 0.0% | 0.0% | 0.1% | 0.1% | 0.1% | 0.0% | 0.1% | 0.2% |
|  | D\_0\_\_Bacteria;D\_1\_\_Bacteroidetes;D\_2\_\_Flavobacteriia | 0.0% | 0.0% | 0.0% | 0.0% | 0.0% | 0.0% | 0.0% | 0.0% | 0.2% |
|  | D\_0\_\_Bacteria;D\_1\_\_Bacteroidetes;D\_2\_\_Sphingobacteriia | 0.1% | 0.1% | 0.1% | 0.1% | 0.1% | 0.0% | 0.0% | 0.0% | 0.0% |
|  | D\_0\_\_Bacteria;D\_1\_\_Chlamydiae;D\_2\_\_Chlamydiae | 0.0% | 0.0% | 0.0% | 0.0% | 0.0% | 0.0% | 0.0% | 0.0% | 0.0% |
|  | D\_0\_\_Bacteria;D\_1\_\_Chlorobi;D\_2\_\_Chlorobia | 0.0% | 0.0% | 0.0% | 0.0% | 0.0% | 0.0% | 0.0% | 0.0% | 0.0% |
|  | D\_0\_\_Bacteria;D\_1\_\_Chloroflexi;D\_2\_\_Anaerolineae | 0.0% | 0.0% | 0.0% | 0.0% | 0.0% | 0.0% | 0.0% | 0.0% | 0.0% |
|  | D\_0\_\_Bacteria;D\_1\_\_Chloroflexi;D\_2\_\_Ardenticatenia | 0.0% | 0.0% | 0.0% | 0.0% | 0.0% | 0.0% | 0.0% | 0.0% | 0.0% |
|  | D\_0\_\_Bacteria;D\_1\_\_Chloroflexi;D\_2\_\_Caldilineae | 0.0% | 0.0% | 0.0% | 0.0% | 0.0% | 0.0% | 0.0% | 0.0% | 0.0% |
|  | D\_0\_\_Bacteria;D\_1\_\_Chloroflexi;D\_2\_\_Chloroflexi Incertae Sedis | 0.0% | 0.0% | 0.0% | 0.0% | 0.0% | 0.0% | 0.0% | 0.0% | 0.0% |
|  | D\_0\_\_Bacteria;D\_1\_\_Chloroflexi;D\_2\_\_Chloroflexia | 0.0% | 0.0% | 0.0% | 0.0% | 0.0% | 0.0% | 0.0% | 0.0% | 0.0% |
|  | D\_0\_\_Bacteria;D\_1\_\_Chloroflexi;D\_2\_\_Gitt-GS-136 | 0.7% | 1.1% | 1.1% | 1.1% | 1.1% | 0.9% | 0.1% | 0.2% | 0.1% |
|  | D\_0\_\_Bacteria;D\_1\_\_Chloroflexi;D\_2\_\_JG30-KF-CM66 | 0.7% | 0.9% | 0.9% | 0.9% | 0.9% | 0.9% | 0.3% | 0.4% | 0.5% |
|  | D\_0\_\_Bacteria;D\_1\_\_Chloroflexi;D\_2\_\_JG37-AG-4 | 0.0% | 0.0% | 0.0% | 0.0% | 0.0% | 0.0% | 0.0% | 0.0% | 0.0% |
|  | D\_0\_\_Bacteria;D\_1\_\_Chloroflexi;D\_2\_\_KD4-96 | 0.1% | 0.1% | 0.1% | 0.1% | 0.1% | 0.0% | 0.0% | 0.0% | 0.2% |
|  | D\_0\_\_Bacteria;D\_1\_\_Chloroflexi;D\_2\_\_Ktedonobacteria | 0.0% | 0.0% | 0.0% | 0.0% | 0.0% | 0.0% | 0.0% | 0.0% | 0.0% |
|  | D\_0\_\_Bacteria;D\_1\_\_Chloroflexi;D\_2\_\_NLS2-31 | 0.0% | 0.0% | 0.0% | 0.0% | 0.0% | 0.0% | 0.0% | 0.0% | 0.0% |
|  | D\_0\_\_Bacteria;D\_1\_\_Chloroflexi;D\_2\_\_P2-11E | 0.0% | 0.0% | 0.0% | 0.0% | 0.0% | 0.0% | 0.0% | 0.0% | 0.0% |
|  | D\_0\_\_Bacteria;D\_1\_\_Chloroflexi;D\_2\_\_S085 | 0.5% | 0.9% | 0.8% | 0.6% | 0.6% | 0.4% | 0.1% | 0.2% | 0.5% |
|  | D\_0\_\_Bacteria;D\_1\_\_Chloroflexi;D\_2\_\_SAR202 clade | 0.1% | 0.1% | 0.0% | 0.1% | 0.1% | 0.0% | 0.0% | 0.1% | 0.0% |
|  | D\_0\_\_Bacteria;D\_1\_\_Chloroflexi;D\_2\_\_TK10 | 0.3% | 0.5% | 0.4% | 0.3% | 0.3% | 0.3% | 0.1% | 0.2% | 0.1% |
|  | D\_0\_\_Bacteria;D\_1\_\_Chloroflexi;D\_2\_\_Thermomicrobia | 0.3% | 0.5% | 0.5% | 0.2% | 0.2% | 0.2% | 0.1% | 0.5% | 0.4% |
|  | D\_0\_\_Bacteria;D\_1\_\_Chloroflexi;Other | 0.0% | 0.0% | 0.0% | 0.0% | 0.0% | 0.0% | 0.0% | 0.0% | 0.0% |
|  | D\_0\_\_Bacteria;D\_1\_\_Cyanobacteria;D\_2\_\_Chloroplast | 0.0% | 0.0% | 0.0% | 0.0% | 0.0% | 0.0% | 0.0% | 0.0% | 0.0% |
|  | D\_0\_\_Bacteria;D\_1\_\_Cyanobacteria;D\_2\_\_Cyanobacteria | 0.0% | 0.0% | 0.0% | 0.0% | 0.0% | 0.0% | 0.0% | 0.0% | 0.0% |
|  | D\_0\_\_Bacteria;D\_1\_\_Cyanobacteria;D\_2\_\_Melainabacteria | 0.0% | 0.0% | 0.0% | 0.0% | 0.0% | 0.0% | 0.0% | 0.0% | 0.0% |
|  | D\_0\_\_Bacteria;D\_1\_\_Deinococcus-Thermus;D\_2\_\_Deinococci | 0.0% | 0.0% | 0.0% | 0.0% | 0.0% | 0.0% | 0.0% | 0.0% | 0.1% |
|  | D\_0\_\_Bacteria;D\_1\_\_Elusimicrobia;D\_2\_\_Elusimicrobia | 0.0% | 0.0% | 0.0% | 0.0% | 0.0% | 0.0% | 0.0% | 0.0% | 0.0% |
|  | D\_0\_\_Bacteria;D\_1\_\_FBP;D\_2\_\_uncultured bacterium | 0.0% | 0.0% | 0.0% | 0.0% | 0.0% | 0.0% | 0.0% | 0.0% | 0.1% |
|  | D\_0\_\_Bacteria;D\_1\_\_Fibrobacteres;D\_2\_\_Fibrobacteria | 0.0% | 0.0% | 0.0% | 0.0% | 0.0% | 0.0% | 0.0% | 0.0% | 0.0% |
|  | D\_0\_\_Bacteria;D\_1\_\_Firmicutes;D\_2\_\_Bacilli | 0.2% | 0.0% | 0.0% | 0.1% | 0.1% | 0.1% | 0.0% | 0.2% | 1.2% |
|  | D\_0\_\_Bacteria;D\_1\_\_Firmicutes;D\_2\_\_Clostridia | 0.1% | 0.0% | 0.0% | 0.0% | 0.0% | 0.0% | 0.0% | 0.0% | 0.5% |
|  | D\_0\_\_Bacteria;D\_1\_\_Firmicutes;D\_2\_\_Erysipelotrichia | 0.0% | 0.0% | 0.0% | 0.0% | 0.0% | 0.0% | 0.0% | 0.0% | 0.1% |
|  | D\_0\_\_Bacteria;D\_1\_\_Firmicutes;D\_2\_\_Negativicutes | 0.0% | 0.0% | 0.0% | 0.0% | 0.0% | 0.0% | 0.0% | 0.0% | 0.0% |
|  | D\_0\_\_Bacteria;D\_1\_\_Fusobacteria;D\_2\_\_Fusobacteriia | 0.1% | 0.0% | 0.0% | 0.0% | 0.0% | 0.0% | 0.0% | 0.1% | 0.5% |
|  | D\_0\_\_Bacteria;D\_1\_\_Gemmatimonadetes;D\_2\_\_BD2-11 terrestrial group | 0.0% | 0.0% | 0.0% | 0.0% | 0.0% | 0.0% | 0.0% | 0.0% | 0.0% |
|  | D\_0\_\_Bacteria;D\_1\_\_Gemmatimonadetes;D\_2\_\_Gemmatimonadetes | 0.0% | 0.0% | 0.0% | 0.0% | 0.0% | 0.0% | 0.0% | 0.0% | 0.0% |
|  | D\_0\_\_Bacteria;D\_1\_\_Gemmatimonadetes;D\_2\_\_Longimicrobia | 0.3% | 0.3% | 0.3% | 0.2% | 0.2% | 0.8% | 0.2% | 0.1% | 0.1% |
|  | D\_0\_\_Bacteria;D\_1\_\_Gemmatimonadetes;D\_2\_\_S0134 terrestrial group | 0.0% | 0.0% | 0.0% | 0.0% | 0.0% | 0.0% | 0.0% | 0.0% | 0.1% |
|  | D\_0\_\_Bacteria;D\_1\_\_Hydrogenedentes;D\_2\_\_uncultured bacterium | 0.0% | 0.0% | 0.0% | 0.0% | 0.0% | 0.0% | 0.0% | 0.0% | 0.0% |
|  | D\_0\_\_Bacteria;D\_1\_\_Nitrospirae;D\_2\_\_Nitrospira | 0.2% | 0.5% | 0.4% | 0.2% | 0.2% | 0.3% | 0.1% | 0.1% | 0.2% |
|  | D\_0\_\_Bacteria;D\_1\_\_Planctomycetes;D\_2\_\_Phycisphaerae | 0.0% | 0.0% | 0.0% | 0.0% | 0.0% | 0.0% | 0.0% | 0.0% | 0.0% |
|  | D\_0\_\_Bacteria;D\_1\_\_Planctomycetes;D\_2\_\_Planctomycetacia | 0.1% | 0.1% | 0.1% | 0.2% | 0.2% | 0.0% | 0.0% | 0.1% | 0.1% |
|  | D\_0\_\_Bacteria;D\_1\_\_Proteobacteria;D\_2\_\_Alphaproteobacteria | 5.3% | 7.3% | 6.3% | 6.8% | 6.9% | 6.0% | 4.6% | 1.3% | 2.9% |
|  | D\_0\_\_Bacteria;D\_1\_\_Proteobacteria;D\_2\_\_Betaproteobacteria | 1.9% | 3.1% | 2.9% | 3.7% | 3.3% | 0.6% | 0.2% | 0.3% | 1.3% |
|  | D\_0\_\_Bacteria;D\_1\_\_Proteobacteria;D\_2\_\_Deltaproteobacteria | 4.2% | 8.1% | 7.9% | 7.0% | 7.9% | 1.2% | 0.6% | 0.3% | 0.6% |
|  | D\_0\_\_Bacteria;D\_1\_\_Proteobacteria;D\_2\_\_Epsilonproteobacteria | 0.0% | 0.0% | 0.0% | 0.0% | 0.0% | 0.0% | 0.0% | 0.0% | 0.0% |
|  | D\_0\_\_Bacteria;D\_1\_\_Proteobacteria;D\_2\_\_Gammaproteobacteria | 0.4% | 0.1% | 0.1% | 0.1% | 0.1% | 0.3% | 0.1% | 0.3% | 2.5% |
|  | D\_0\_\_Bacteria;D\_1\_\_Proteobacteria;D\_2\_\_JTB23 | 0.0% | 0.0% | 0.0% | 0.0% | 0.0% | 0.0% | 0.0% | 0.0% | 0.0% |
|  | D\_0\_\_Bacteria;D\_1\_\_Proteobacteria;D\_2\_\_SPOTSOCT00m83 | 0.0% | 0.0% | 0.0% | 0.0% | 0.0% | 0.0% | 0.0% | 0.0% | 0.0% |
|  | D\_0\_\_Bacteria;D\_1\_\_Proteobacteria;Other | 0.0% | 0.0% | 0.0% | 0.0% | 0.0% | 0.0% | 0.0% | 0.0% | 0.0% |
|  | D\_0\_\_Bacteria;D\_1\_\_SBR1093;Other | 0.0% | 0.0% | 0.0% | 0.0% | 0.0% | 0.0% | 0.0% | 0.0% | 0.0% |
|  | D\_0\_\_Bacteria;D\_1\_\_SR1 (Absconditabacteria);Ambiguous\_taxa | 0.0% | 0.0% | 0.0% | 0.0% | 0.0% | 0.0% | 0.0% | 0.0% | 0.0% |
|  | D\_0\_\_Bacteria;D\_1\_\_SR1 (Absconditabacteria);D\_2\_\_uncultured bacterium | 0.0% | 0.0% | 0.0% | 0.0% | 0.0% | 0.0% | 0.0% | 0.0% | 0.1% |
|  | D\_0\_\_Bacteria;D\_1\_\_Saccharibacteria;D\_2\_\_uncultured bacterium | 0.0% | 0.0% | 0.0% | 0.0% | 0.0% | 0.0% | 0.0% | 0.0% | 0.0% |
|  | D\_0\_\_Bacteria;D\_1\_\_Saccharibacteria;Other | 0.0% | 0.0% | 0.0% | 0.0% | 0.0% | 0.0% | 0.0% | 0.0% | 0.0% |
|  | D\_0\_\_Bacteria;D\_1\_\_Spirochaetae;D\_2\_\_Spirochaetes | 0.0% | 0.0% | 0.0% | 0.0% | 0.0% | 0.0% | 0.0% | 0.0% | 0.1% |
|  | D\_0\_\_Bacteria;D\_1\_\_Synergistetes;D\_2\_\_Synergistia | 0.0% | 0.0% | 0.0% | 0.0% | 0.0% | 0.0% | 0.0% | 0.0% | 0.0% |
|  | D\_0\_\_Bacteria;D\_1\_\_TM6 (Dependentiae);Other | 0.0% | 0.0% | 0.0% | 0.0% | 0.0% | 0.0% | 0.0% | 0.0% | 0.0% |
|  | D\_0\_\_Bacteria;D\_1\_\_Tectomicrobia;D\_2\_\_Tectomicrobia Incertae Sedis | 0.0% | 0.0% | 0.0% | 0.0% | 0.0% | 0.0% | 0.0% | 0.0% | 0.0% |
|  | D\_0\_\_Bacteria;D\_1\_\_Tectomicrobia;D\_2\_\_uncultured bacterium | 0.0% | 0.0% | 0.0% | 0.0% | 0.0% | 0.0% | 0.0% | 0.0% | 0.0% |
|  | D\_0\_\_Bacteria;D\_1\_\_Verrucomicrobia;D\_2\_\_OPB35 soil group | 0.0% | 0.0% | 0.0% | 0.0% | 0.0% | 0.0% | 0.0% | 0.0% | 0.0% |
|  | D\_0\_\_Bacteria;D\_1\_\_Verrucomicrobia;D\_2\_\_Opitutae | 0.0% | 0.0% | 0.0% | 0.0% | 0.0% | 0.0% | 0.0% | 0.0% | 0.0% |
|  | D\_0\_\_Bacteria;D\_1\_\_Verrucomicrobia;D\_2\_\_Spartobacteria | 0.0% | 0.0% | 0.0% | 0.0% | 0.0% | 0.0% | 0.0% | 0.0% | 0.0% |
|  | D\_0\_\_Bacteria;D\_1\_\_Verrucomicrobia;D\_2\_\_Verrucomicrobiae | 0.0% | 0.0% | 0.0% | 0.0% | 0.0% | 0.0% | 0.0% | 0.0% | 0.0% |
|  | D\_0\_\_Bacteria;Other;Other | 0.0% | 0.0% | 0.0% | 0.0% | 0.0% | 0.0% | 0.0% | 0.0% | 0.0% |
|  | Unassigned;Other;Other | 0.3% | 0.4% | 0.4% | 0.3% | 0.3% | 0.4% | 0.1% | 0.2% | 0.2% |

|  |  |
| --- | --- |
|  | |
| Taxonomy Summary. Current Level: | |
| View Figure (.pdf)  View Legend (.pdf) |  |
|  |


|  |
| --- |
| View Table (.txt) |

|  |  |  |  |  |  |  |  |  |  |  |
| --- | --- | --- | --- | --- | --- | --- | --- | --- | --- | --- |
|  | | Total | 1B5g1 | 1B5g2 | 1BEDTA1 | 1BEDTA2 | 1M1 | 1M2 | 1S1 | 1S2 |
| Legend | Taxonomy | % | % | % | % | % | % | % | % | % |
|  | D\_0\_\_Archaea;D\_1\_\_Thaumarchaeota;D\_2\_\_Soil Crenarchaeotic Group(SCG);D\_3\_\_Unknown Order | 0.0% | 0.0% | 0.0% | 0.0% | 0.0% | 0.0% | 0.0% | 0.0% | 0.0% |
|  | D\_0\_\_Archaea;D\_1\_\_Thaumarchaeota;D\_2\_\_Soil Crenarchaeotic Group(SCG);D\_3\_\_uncultured archaeon | 0.0% | 0.0% | 0.0% | 0.0% | 0.0% | 0.0% | 0.0% | 0.0% | 0.0% |
|  | D\_0\_\_Archaea;D\_1\_\_Thaumarchaeota;D\_2\_\_Soil Crenarchaeotic Group(SCG);D\_3\_\_uncultured euryarchaeote | 0.0% | 0.0% | 0.0% | 0.0% | 0.0% | 0.0% | 0.0% | 0.0% | 0.0% |
|  | D\_0\_\_Archaea;D\_1\_\_Thaumarchaeota;D\_2\_\_Soil Crenarchaeotic Group(SCG);Other | 0.6% | 0.7% | 0.7% | 0.4% | 0.5% | 1.0% | 0.8% | 0.2% | 0.3% |
|  | D\_0\_\_Bacteria;D\_1\_\_Acidobacteria;D\_2\_\_Blastocatellia;D\_3\_\_Blastocatellales | 0.0% | 0.0% | 0.0% | 0.0% | 0.0% | 0.0% | 0.0% | 0.0% | 0.0% |
|  | D\_0\_\_Bacteria;D\_1\_\_Acidobacteria;D\_2\_\_Holophagae;D\_3\_\_Holophagales | 0.0% | 0.0% | 0.0% | 0.0% | 0.0% | 0.0% | 0.0% | 0.0% | 0.0% |
|  | D\_0\_\_Bacteria;D\_1\_\_Acidobacteria;D\_2\_\_Holophagae;D\_3\_\_Subgroup 10 | 0.0% | 0.0% | 0.0% | 0.0% | 0.0% | 0.1% | 0.0% | 0.0% | 0.0% |
|  | D\_0\_\_Bacteria;D\_1\_\_Acidobacteria;D\_2\_\_Holophagae;D\_3\_\_Subgroup 7 | 0.0% | 0.0% | 0.0% | 0.0% | 0.0% | 0.0% | 0.0% | 0.0% | 0.0% |
|  | D\_0\_\_Bacteria;D\_1\_\_Acidobacteria;D\_2\_\_Solibacteres;D\_3\_\_Solibacterales | 0.0% | 0.0% | 0.0% | 0.0% | 0.0% | 0.0% | 0.0% | 0.0% | 0.0% |
|  | D\_0\_\_Bacteria;D\_1\_\_Acidobacteria;D\_2\_\_Subgroup 17;Ambiguous\_taxa | 0.0% | 0.0% | 0.0% | 0.0% | 0.0% | 0.0% | 0.0% | 0.0% | 0.0% |
|  | D\_0\_\_Bacteria;D\_1\_\_Acidobacteria;D\_2\_\_Subgroup 17;D\_3\_\_uncultured Acidobacteria bacterium | 0.0% | 0.0% | 0.0% | 0.0% | 0.0% | 0.0% | 0.0% | 0.0% | 0.0% |
|  | D\_0\_\_Bacteria;D\_1\_\_Acidobacteria;D\_2\_\_Subgroup 17;Other | 2.5% | 3.3% | 2.9% | 3.0% | 3.0% | 2.8% | 1.1% | 2.5% | 1.6% |
|  | D\_0\_\_Bacteria;D\_1\_\_Acidobacteria;D\_2\_\_Subgroup 22;Ambiguous\_taxa | 0.0% | 0.0% | 0.0% | 0.0% | 0.0% | 0.0% | 0.0% | 0.0% | 0.0% |
|  | D\_0\_\_Bacteria;D\_1\_\_Acidobacteria;D\_2\_\_Subgroup 22;D\_3\_\_uncultured Acidobacteria bacterium | 0.0% | 0.0% | 0.0% | 0.0% | 0.0% | 0.0% | 0.0% | 0.0% | 0.0% |
|  | D\_0\_\_Bacteria;D\_1\_\_Acidobacteria;D\_2\_\_Subgroup 22;D\_3\_\_uncultured bacterium | 0.0% | 0.0% | 0.0% | 0.0% | 0.0% | 0.0% | 0.0% | 0.0% | 0.0% |
|  | D\_0\_\_Bacteria;D\_1\_\_Acidobacteria;D\_2\_\_Subgroup 22;Other | 0.0% | 0.0% | 0.0% | 0.0% | 0.0% | 0.0% | 0.0% | 0.0% | 0.0% |
|  | D\_0\_\_Bacteria;D\_1\_\_Acidobacteria;D\_2\_\_Subgroup 5;D\_3\_\_uncultured bacterium | 0.0% | 0.0% | 0.0% | 0.0% | 0.0% | 0.0% | 0.0% | 0.0% | 0.0% |
|  | D\_0\_\_Bacteria;D\_1\_\_Acidobacteria;D\_2\_\_Subgroup 5;Other | 0.0% | 0.0% | 0.0% | 0.0% | 0.0% | 0.0% | 0.0% | 0.0% | 0.0% |
|  | D\_0\_\_Bacteria;D\_1\_\_Acidobacteria;D\_2\_\_Subgroup 6;Ambiguous\_taxa | 0.1% | 0.1% | 0.1% | 0.1% | 0.1% | 0.1% | 0.0% | 0.0% | 0.0% |
|  | D\_0\_\_Bacteria;D\_1\_\_Acidobacteria;D\_2\_\_Subgroup 6;D\_3\_\_uncultured Acidobacteria bacterium | 0.0% | 0.0% | 0.0% | 0.0% | 0.0% | 0.0% | 0.0% | 0.0% | 0.0% |
|  | D\_0\_\_Bacteria;D\_1\_\_Acidobacteria;D\_2\_\_Subgroup 6;D\_3\_\_uncultured bacterium | 0.2% | 0.5% | 0.3% | 0.3% | 0.3% | 0.0% | 0.0% | 0.0% | 0.0% |
|  | D\_0\_\_Bacteria;D\_1\_\_Acidobacteria;D\_2\_\_Subgroup 6;D\_3\_\_uncultured organism | 0.0% | 0.0% | 0.0% | 0.0% | 0.0% | 0.0% | 0.0% | 0.0% | 0.0% |
|  | D\_0\_\_Bacteria;D\_1\_\_Acidobacteria;D\_2\_\_Subgroup 6;D\_3\_\_uncultured soil bacterium | 0.0% | 0.0% | 0.0% | 0.0% | 0.0% | 0.0% | 0.0% | 0.0% | 0.0% |
|  | D\_0\_\_Bacteria;D\_1\_\_Acidobacteria;D\_2\_\_Subgroup 6;Other | 0.0% | 0.0% | 0.0% | 0.0% | 0.0% | 0.0% | 0.0% | 0.0% | 0.0% |
|  | D\_0\_\_Bacteria;D\_1\_\_Acidobacteria;Other;Other | 0.0% | 0.0% | 0.0% | 0.0% | 0.0% | 0.0% | 0.0% | 0.0% | 0.0% |
|  | D\_0\_\_Bacteria;D\_1\_\_Actinobacteria;D\_2\_\_Acidimicrobiia;D\_3\_\_Acidimicrobiales | 3.5% | 6.1% | 6.3% | 4.4% | 4.8% | 3.0% | 1.0% | 1.4% | 1.0% |
|  | D\_0\_\_Bacteria;D\_1\_\_Actinobacteria;D\_2\_\_Actinobacteria;D\_3\_\_Actinomycetales | 0.0% | 0.0% | 0.0% | 0.0% | 0.0% | 0.0% | 0.0% | 0.0% | 0.3% |
|  | D\_0\_\_Bacteria;D\_1\_\_Actinobacteria;D\_2\_\_Actinobacteria;D\_3\_\_Actinopolysporales | 0.0% | 0.0% | 0.0% | 0.0% | 0.0% | 0.0% | 0.0% | 0.0% | 0.0% |
|  | D\_0\_\_Bacteria;D\_1\_\_Actinobacteria;D\_2\_\_Actinobacteria;D\_3\_\_Bifidobacteriales | 0.0% | 0.0% | 0.0% | 0.0% | 0.0% | 0.0% | 0.0% | 0.0% | 0.0% |
|  | D\_0\_\_Bacteria;D\_1\_\_Actinobacteria;D\_2\_\_Actinobacteria;D\_3\_\_Corynebacteriales | 0.1% | 0.0% | 0.0% | 0.0% | 0.0% | 0.0% | 0.0% | 0.1% | 0.7% |
|  | D\_0\_\_Bacteria;D\_1\_\_Actinobacteria;D\_2\_\_Actinobacteria;D\_3\_\_Elev-16S-976 | 0.0% | 0.0% | 0.0% | 0.0% | 0.0% | 0.0% | 0.0% | 0.0% | 0.0% |
|  | D\_0\_\_Bacteria;D\_1\_\_Actinobacteria;D\_2\_\_Actinobacteria;D\_3\_\_Frankiales | 14.0% | 11.4% | 11.9% | 10.9% | 11.4% | 8.6% | 7.4% | 27.0% | 23.1% |
|  | D\_0\_\_Bacteria;D\_1\_\_Actinobacteria;D\_2\_\_Actinobacteria;D\_3\_\_Glycomycetales | 0.0% | 0.0% | 0.0% | 0.0% | 0.0% | 0.0% | 0.0% | 0.0% | 0.0% |
|  | D\_0\_\_Bacteria;D\_1\_\_Actinobacteria;D\_2\_\_Actinobacteria;D\_3\_\_Kineosporiales | 0.0% | 0.0% | 0.0% | 0.0% | 0.0% | 0.0% | 0.0% | 0.0% | 0.0% |
|  | D\_0\_\_Bacteria;D\_1\_\_Actinobacteria;D\_2\_\_Actinobacteria;D\_3\_\_Micrococcales | 1.7% | 0.6% | 0.6% | 0.8% | 0.8% | 1.8% | 3.1% | 3.6% | 2.5% |
|  | D\_0\_\_Bacteria;D\_1\_\_Actinobacteria;D\_2\_\_Actinobacteria;D\_3\_\_Micromonosporales | 3.5% | 5.0% | 5.3% | 5.0% | 5.2% | 3.3% | 2.7% | 0.7% | 0.4% |
|  | D\_0\_\_Bacteria;D\_1\_\_Actinobacteria;D\_2\_\_Actinobacteria;D\_3\_\_PeM15 | 0.0% | 0.0% | 0.0% | 0.0% | 0.0% | 0.0% | 0.0% | 0.0% | 0.0% |
|  | D\_0\_\_Bacteria;D\_1\_\_Actinobacteria;D\_2\_\_Actinobacteria;D\_3\_\_Propionibacteriales | 5.0% | 4.1% | 4.2% | 2.4% | 2.4% | 9.2% | 7.6% | 5.7% | 4.2% |
|  | D\_0\_\_Bacteria;D\_1\_\_Actinobacteria;D\_2\_\_Actinobacteria;D\_3\_\_Pseudonocardiales | 29.5% | 10.9% | 12.2% | 16.4% | 15.5% | 40.1% | 42.9% | 49.2% | 48.6% |
|  | D\_0\_\_Bacteria;D\_1\_\_Actinobacteria;D\_2\_\_Actinobacteria;D\_3\_\_Streptomycetales | 4.3% | 0.1% | 0.1% | 0.1% | 0.1% | 11.4% | 22.7% | 0.1% | 0.0% |
|  | D\_0\_\_Bacteria;D\_1\_\_Actinobacteria;D\_2\_\_Actinobacteria;D\_3\_\_Streptosporangiales | 0.0% | 0.0% | 0.0% | 0.0% | 0.0% | 0.0% | 0.0% | 0.0% | 0.0% |
|  | D\_0\_\_Bacteria;D\_1\_\_Actinobacteria;D\_2\_\_Actinobacteria;Other | 0.0% | 0.0% | 0.0% | 0.0% | 0.0% | 0.1% | 0.1% | 0.0% | 0.0% |
|  | D\_0\_\_Bacteria;D\_1\_\_Actinobacteria;D\_2\_\_Coriobacteriia;D\_3\_\_Coriobacteriales | 0.0% | 0.0% | 0.0% | 0.0% | 0.0% | 0.0% | 0.0% | 0.0% | 0.0% |
|  | D\_0\_\_Bacteria;D\_1\_\_Actinobacteria;D\_2\_\_MB-A2-108;Ambiguous\_taxa | 0.0% | 0.0% | 0.0% | 0.0% | 0.0% | 0.0% | 0.0% | 0.0% | 0.0% |
|  | D\_0\_\_Bacteria;D\_1\_\_Actinobacteria;D\_2\_\_MB-A2-108;D\_3\_\_uncultured bacterium | 0.0% | 0.0% | 0.0% | 0.0% | 0.0% | 0.0% | 0.0% | 0.0% | 0.0% |
|  | D\_0\_\_Bacteria;D\_1\_\_Actinobacteria;D\_2\_\_MB-A2-108;Other | 0.0% | 0.0% | 0.0% | 0.0% | 0.0% | 0.0% | 0.0% | 0.0% | 0.0% |
|  | D\_0\_\_Bacteria;D\_1\_\_Actinobacteria;D\_2\_\_Nitriliruptoria;D\_3\_\_Euzebyales | 15.3% | 27.5% | 28.0% | 29.1% | 28.6% | 2.3% | 2.6% | 2.4% | 2.1% |
|  | D\_0\_\_Bacteria;D\_1\_\_Actinobacteria;D\_2\_\_Nitriliruptoria;D\_3\_\_Nitriliruptorales | 0.0% | 0.1% | 0.0% | 0.0% | 0.0% | 0.0% | 0.0% | 0.0% | 0.0% |
|  | D\_0\_\_Bacteria;D\_1\_\_Actinobacteria;D\_2\_\_Nitriliruptoria;Other | 0.0% | 0.0% | 0.0% | 0.0% | 0.0% | 0.0% | 0.0% | 0.0% | 0.0% |
|  | D\_0\_\_Bacteria;D\_1\_\_Actinobacteria;D\_2\_\_OPB41;D\_3\_\_uncultured bacterium | 0.0% | 0.0% | 0.0% | 0.0% | 0.0% | 0.0% | 0.0% | 0.0% | 0.0% |
|  | D\_0\_\_Bacteria;D\_1\_\_Actinobacteria;D\_2\_\_Rubrobacteria;D\_3\_\_Rubrobacterales | 0.0% | 0.0% | 0.0% | 0.0% | 0.0% | 0.0% | 0.0% | 0.0% | 0.1% |
|  | D\_0\_\_Bacteria;D\_1\_\_Actinobacteria;D\_2\_\_TakashiAC-B11;D\_3\_\_uncultured Actinomycetales bacterium | 0.0% | 0.0% | 0.0% | 0.0% | 0.0% | 0.0% | 0.0% | 0.0% | 0.0% |
|  | D\_0\_\_Bacteria;D\_1\_\_Actinobacteria;D\_2\_\_TakashiAC-B11;D\_3\_\_uncultured actinobacterium | 0.0% | 0.0% | 0.0% | 0.0% | 0.0% | 0.0% | 0.0% | 0.0% | 0.0% |
|  | D\_0\_\_Bacteria;D\_1\_\_Actinobacteria;D\_2\_\_TakashiAC-B11;D\_3\_\_uncultured bacterium | 0.6% | 0.8% | 0.7% | 1.0% | 1.0% | 0.4% | 0.1% | 0.3% | 0.3% |
|  | D\_0\_\_Bacteria;D\_1\_\_Actinobacteria;D\_2\_\_TakashiAC-B11;Other | 1.7% | 3.1% | 2.9% | 2.4% | 2.2% | 1.0% | 0.1% | 1.0% | 0.8% |
|  | D\_0\_\_Bacteria;D\_1\_\_Actinobacteria;D\_2\_\_Thermoleophilia;D\_3\_\_Gaiellales | 0.3% | 0.5% | 0.4% | 0.4% | 0.4% | 0.4% | 0.1% | 0.1% | 0.3% |
|  | D\_0\_\_Bacteria;D\_1\_\_Actinobacteria;D\_2\_\_Thermoleophilia;D\_3\_\_Solirubrobacterales | 0.8% | 1.0% | 0.9% | 0.6% | 0.6% | 1.6% | 1.1% | 0.4% | 0.4% |
|  | D\_0\_\_Bacteria;D\_1\_\_Actinobacteria;D\_2\_\_Thermoleophilia;Other | 0.0% | 0.0% | 0.0% | 0.0% | 0.0% | 0.0% | 0.0% | 0.0% | 0.0% |
|  | D\_0\_\_Bacteria;D\_1\_\_Actinobacteria;Other;Other | 0.0% | 0.0% | 0.0% | 0.0% | 0.0% | 0.0% | 0.0% | 0.0% | 0.0% |
|  | D\_0\_\_Bacteria;D\_1\_\_Armatimonadetes;D\_2\_\_Armatimonadia;D\_3\_\_Armatimonadales | 0.0% | 0.0% | 0.0% | 0.0% | 0.0% | 0.0% | 0.0% | 0.0% | 0.0% |
|  | D\_0\_\_Bacteria;D\_1\_\_Armatimonadetes;D\_2\_\_Fimbriimonadia;D\_3\_\_Fimbriimonadales | 0.0% | 0.0% | 0.0% | 0.0% | 0.0% | 0.0% | 0.0% | 0.0% | 0.0% |
|  | D\_0\_\_Bacteria;D\_1\_\_Armatimonadetes;D\_2\_\_uncultured;D\_3\_\_uncultured bacterium | 0.0% | 0.0% | 0.0% | 0.0% | 0.0% | 0.0% | 0.0% | 0.0% | 0.0% |
|  | D\_0\_\_Bacteria;D\_1\_\_BRC1;D\_2\_\_uncultured bacterium;D\_3\_\_uncultured bacterium | 0.0% | 0.0% | 0.0% | 0.0% | 0.0% | 0.0% | 0.0% | 0.0% | 0.0% |
|  | D\_0\_\_Bacteria;D\_1\_\_BRC1;Other;Other | 0.0% | 0.0% | 0.0% | 0.0% | 0.0% | 0.0% | 0.0% | 0.0% | 0.0% |
|  | D\_0\_\_Bacteria;D\_1\_\_Bacteroidetes;D\_2\_\_Bacteroidetes BD2-2;D\_3\_\_uncultured bacterium | 0.0% | 0.0% | 0.0% | 0.0% | 0.0% | 0.0% | 0.0% | 0.0% | 0.0% |
|  | D\_0\_\_Bacteria;D\_1\_\_Bacteroidetes;D\_2\_\_Bacteroidetes Incertae Sedis;D\_3\_\_Order II | 0.0% | 0.0% | 0.0% | 0.0% | 0.0% | 0.0% | 0.0% | 0.0% | 0.0% |
|  | D\_0\_\_Bacteria;D\_1\_\_Bacteroidetes;D\_2\_\_Bacteroidia;D\_3\_\_Bacteroidales | 0.1% | 0.0% | 0.0% | 0.0% | 0.0% | 0.0% | 0.0% | 0.1% | 0.5% |
|  | D\_0\_\_Bacteria;D\_1\_\_Bacteroidetes;D\_2\_\_Cytophagia;D\_3\_\_Cytophagales | 0.1% | 0.0% | 0.0% | 0.1% | 0.1% | 0.1% | 0.0% | 0.1% | 0.2% |
|  | D\_0\_\_Bacteria;D\_1\_\_Bacteroidetes;D\_2\_\_Flavobacteriia;D\_3\_\_Flavobacteriales | 0.0% | 0.0% | 0.0% | 0.0% | 0.0% | 0.0% | 0.0% | 0.0% | 0.2% |
|  | D\_0\_\_Bacteria;D\_1\_\_Bacteroidetes;D\_2\_\_Sphingobacteriia;D\_3\_\_Sphingobacteriales | 0.1% | 0.1% | 0.1% | 0.1% | 0.1% | 0.0% | 0.0% | 0.0% | 0.0% |
|  | D\_0\_\_Bacteria;D\_1\_\_Chlamydiae;D\_2\_\_Chlamydiae;D\_3\_\_Chlamydiales | 0.0% | 0.0% | 0.0% | 0.0% | 0.0% | 0.0% | 0.0% | 0.0% | 0.0% |
|  | D\_0\_\_Bacteria;D\_1\_\_Chlorobi;D\_2\_\_Chlorobia;D\_3\_\_Chlorobiales | 0.0% | 0.0% | 0.0% | 0.0% | 0.0% | 0.0% | 0.0% | 0.0% | 0.0% |
|  | D\_0\_\_Bacteria;D\_1\_\_Chloroflexi;D\_2\_\_Anaerolineae;D\_3\_\_Anaerolineales | 0.0% | 0.0% | 0.0% | 0.0% | 0.0% | 0.0% | 0.0% | 0.0% | 0.0% |
|  | D\_0\_\_Bacteria;D\_1\_\_Chloroflexi;D\_2\_\_Ardenticatenia;D\_3\_\_Ardenticatenales | 0.0% | 0.0% | 0.0% | 0.0% | 0.0% | 0.0% | 0.0% | 0.0% | 0.0% |
|  | D\_0\_\_Bacteria;D\_1\_\_Chloroflexi;D\_2\_\_Caldilineae;D\_3\_\_Caldilineales | 0.0% | 0.0% | 0.0% | 0.0% | 0.0% | 0.0% | 0.0% | 0.0% | 0.0% |
|  | D\_0\_\_Bacteria;D\_1\_\_Chloroflexi;D\_2\_\_Chloroflexi Incertae Sedis;D\_3\_\_Unknown Order | 0.0% | 0.0% | 0.0% | 0.0% | 0.0% | 0.0% | 0.0% | 0.0% | 0.0% |
|  | D\_0\_\_Bacteria;D\_1\_\_Chloroflexi;D\_2\_\_Chloroflexia;D\_3\_\_Chloroflexales | 0.0% | 0.0% | 0.0% | 0.0% | 0.0% | 0.0% | 0.0% | 0.0% | 0.0% |
|  | D\_0\_\_Bacteria;D\_1\_\_Chloroflexi;D\_2\_\_Chloroflexia;D\_3\_\_Herpetosiphonales | 0.0% | 0.0% | 0.0% | 0.0% | 0.0% | 0.0% | 0.0% | 0.0% | 0.0% |
|  | D\_0\_\_Bacteria;D\_1\_\_Chloroflexi;D\_2\_\_Chloroflexia;D\_3\_\_Kallotenuales | 0.0% | 0.0% | 0.0% | 0.0% | 0.0% | 0.0% | 0.0% | 0.0% | 0.0% |
|  | D\_0\_\_Bacteria;D\_1\_\_Chloroflexi;D\_2\_\_Gitt-GS-136;Ambiguous\_taxa | 0.0% | 0.0% | 0.0% | 0.0% | 0.0% | 0.0% | 0.0% | 0.0% | 0.0% |
|  | D\_0\_\_Bacteria;D\_1\_\_Chloroflexi;D\_2\_\_Gitt-GS-136;D\_3\_\_uncultured bacterium | 0.0% | 0.0% | 0.0% | 0.0% | 0.0% | 0.0% | 0.0% | 0.0% | 0.0% |
|  | D\_0\_\_Bacteria;D\_1\_\_Chloroflexi;D\_2\_\_Gitt-GS-136;D\_3\_\_uncultured bacterium #0319-6C24 | 0.0% | 0.0% | 0.0% | 0.0% | 0.0% | 0.0% | 0.0% | 0.0% | 0.0% |
|  | D\_0\_\_Bacteria;D\_1\_\_Chloroflexi;D\_2\_\_Gitt-GS-136;Other | 0.7% | 1.1% | 1.1% | 1.1% | 1.1% | 0.8% | 0.1% | 0.2% | 0.1% |
|  | D\_0\_\_Bacteria;D\_1\_\_Chloroflexi;D\_2\_\_JG30-KF-CM66;Ambiguous\_taxa | 0.0% | 0.0% | 0.0% | 0.0% | 0.0% | 0.0% | 0.0% | 0.0% | 0.0% |
|  | D\_0\_\_Bacteria;D\_1\_\_Chloroflexi;D\_2\_\_JG30-KF-CM66;D\_3\_\_uncultured Chloroflexi bacterium | 0.0% | 0.0% | 0.0% | 0.0% | 0.0% | 0.0% | 0.0% | 0.0% | 0.0% |
|  | D\_0\_\_Bacteria;D\_1\_\_Chloroflexi;D\_2\_\_JG30-KF-CM66;D\_3\_\_uncultured bacterium | 0.6% | 0.8% | 0.7% | 0.7% | 0.7% | 0.8% | 0.3% | 0.4% | 0.5% |
|  | D\_0\_\_Bacteria;D\_1\_\_Chloroflexi;D\_2\_\_JG30-KF-CM66;Other | 0.1% | 0.2% | 0.2% | 0.2% | 0.2% | 0.1% | 0.0% | 0.1% | 0.0% |
|  | D\_0\_\_Bacteria;D\_1\_\_Chloroflexi;D\_2\_\_JG37-AG-4;D\_3\_\_uncultured bacterium | 0.0% | 0.0% | 0.0% | 0.0% | 0.0% | 0.0% | 0.0% | 0.0% | 0.0% |
|  | D\_0\_\_Bacteria;D\_1\_\_Chloroflexi;D\_2\_\_KD4-96;Ambiguous\_taxa | 0.0% | 0.0% | 0.0% | 0.0% | 0.0% | 0.0% | 0.0% | 0.0% | 0.0% |
|  | D\_0\_\_Bacteria;D\_1\_\_Chloroflexi;D\_2\_\_KD4-96;D\_3\_\_uncultured bacterium | 0.1% | 0.1% | 0.1% | 0.1% | 0.1% | 0.0% | 0.0% | 0.0% | 0.2% |
|  | D\_0\_\_Bacteria;D\_1\_\_Chloroflexi;D\_2\_\_KD4-96;Other | 0.0% | 0.0% | 0.0% | 0.0% | 0.0% | 0.0% | 0.0% | 0.0% | 0.0% |
|  | D\_0\_\_Bacteria;D\_1\_\_Chloroflexi;D\_2\_\_Ktedonobacteria;D\_3\_\_C0119 | 0.0% | 0.0% | 0.0% | 0.0% | 0.0% | 0.0% | 0.0% | 0.0% | 0.0% |
|  | D\_0\_\_Bacteria;D\_1\_\_Chloroflexi;D\_2\_\_NLS2-31;D\_3\_\_uncultured bacterium | 0.0% | 0.0% | 0.0% | 0.0% | 0.0% | 0.0% | 0.0% | 0.0% | 0.0% |
|  | D\_0\_\_Bacteria;D\_1\_\_Chloroflexi;D\_2\_\_P2-11E;D\_3\_\_uncultured bacterium | 0.0% | 0.0% | 0.0% | 0.0% | 0.0% | 0.0% | 0.0% | 0.0% | 0.0% |
|  | D\_0\_\_Bacteria;D\_1\_\_Chloroflexi;D\_2\_\_S085;Ambiguous\_taxa | 0.0% | 0.0% | 0.0% | 0.0% | 0.0% | 0.0% | 0.0% | 0.0% | 0.0% |
|  | D\_0\_\_Bacteria;D\_1\_\_Chloroflexi;D\_2\_\_S085;D\_3\_\_uncultured Chloroflexi bacterium | 0.0% | 0.0% | 0.0% | 0.0% | 0.0% | 0.0% | 0.0% | 0.0% | 0.0% |
|  | D\_0\_\_Bacteria;D\_1\_\_Chloroflexi;D\_2\_\_S085;D\_3\_\_uncultured bacterium | 0.5% | 0.9% | 0.8% | 0.6% | 0.6% | 0.4% | 0.1% | 0.2% | 0.5% |
|  | D\_0\_\_Bacteria;D\_1\_\_Chloroflexi;D\_2\_\_S085;Other | 0.0% | 0.0% | 0.0% | 0.0% | 0.0% | 0.0% | 0.0% | 0.0% | 0.0% |
|  | D\_0\_\_Bacteria;D\_1\_\_Chloroflexi;D\_2\_\_SAR202 clade;Ambiguous\_taxa | 0.0% | 0.0% | 0.0% | 0.0% | 0.0% | 0.0% | 0.0% | 0.0% | 0.0% |
|  | D\_0\_\_Bacteria;D\_1\_\_Chloroflexi;D\_2\_\_SAR202 clade;D\_3\_\_uncultured Chloroflexi bacterium | 0.0% | 0.0% | 0.0% | 0.0% | 0.0% | 0.0% | 0.0% | 0.0% | 0.0% |
|  | D\_0\_\_Bacteria;D\_1\_\_Chloroflexi;D\_2\_\_SAR202 clade;D\_3\_\_uncultured bacterium | 0.1% | 0.1% | 0.0% | 0.1% | 0.1% | 0.0% | 0.0% | 0.1% | 0.0% |
|  | D\_0\_\_Bacteria;D\_1\_\_Chloroflexi;D\_2\_\_SAR202 clade;Other | 0.0% | 0.0% | 0.0% | 0.0% | 0.0% | 0.0% | 0.0% | 0.0% | 0.0% |
|  | D\_0\_\_Bacteria;D\_1\_\_Chloroflexi;D\_2\_\_TK10;D\_3\_\_uncultured Chloroflexi bacterium | 0.0% | 0.0% | 0.0% | 0.0% | 0.0% | 0.0% | 0.0% | 0.0% | 0.0% |
|  | D\_0\_\_Bacteria;D\_1\_\_Chloroflexi;D\_2\_\_TK10;D\_3\_\_uncultured bacterium | 0.2% | 0.4% | 0.3% | 0.3% | 0.3% | 0.3% | 0.1% | 0.2% | 0.1% |
|  | D\_0\_\_Bacteria;D\_1\_\_Chloroflexi;D\_2\_\_TK10;D\_3\_\_uncultured soil bacterium | 0.0% | 0.0% | 0.0% | 0.0% | 0.0% | 0.0% | 0.0% | 0.0% | 0.0% |
|  | D\_0\_\_Bacteria;D\_1\_\_Chloroflexi;D\_2\_\_TK10;Other | 0.0% | 0.0% | 0.0% | 0.0% | 0.0% | 0.0% | 0.0% | 0.0% | 0.0% |
|  | D\_0\_\_Bacteria;D\_1\_\_Chloroflexi;D\_2\_\_Thermomicrobia;D\_3\_\_AKYG1722 | 0.2% | 0.4% | 0.3% | 0.2% | 0.2% | 0.1% | 0.1% | 0.2% | 0.2% |
|  | D\_0\_\_Bacteria;D\_1\_\_Chloroflexi;D\_2\_\_Thermomicrobia;D\_3\_\_JG30-KF-CM45 | 0.1% | 0.2% | 0.2% | 0.0% | 0.0% | 0.1% | 0.0% | 0.3% | 0.2% |
|  | D\_0\_\_Bacteria;D\_1\_\_Chloroflexi;D\_2\_\_Thermomicrobia;D\_3\_\_Sphaerobacterales | 0.0% | 0.0% | 0.0% | 0.0% | 0.0% | 0.0% | 0.0% | 0.0% | 0.0% |
|  | D\_0\_\_Bacteria;D\_1\_\_Chloroflexi;Other;Other | 0.0% | 0.0% | 0.0% | 0.0% | 0.0% | 0.0% | 0.0% | 0.0% | 0.0% |
|  | D\_0\_\_Bacteria;D\_1\_\_Cyanobacteria;D\_2\_\_Chloroplast;Ambiguous\_taxa | 0.0% | 0.0% | 0.0% | 0.0% | 0.0% | 0.0% | 0.0% | 0.0% | 0.0% |
|  | D\_0\_\_Bacteria;D\_1\_\_Cyanobacteria;D\_2\_\_Chloroplast;D\_3\_\_uncultured bacterium | 0.0% | 0.0% | 0.0% | 0.0% | 0.0% | 0.0% | 0.0% | 0.0% | 0.0% |
|  | D\_0\_\_Bacteria;D\_1\_\_Cyanobacteria;D\_2\_\_Cyanobacteria;D\_3\_\_SubsectionIII | 0.0% | 0.0% | 0.0% | 0.0% | 0.0% | 0.0% | 0.0% | 0.0% | 0.0% |
|  | D\_0\_\_Bacteria;D\_1\_\_Cyanobacteria;D\_2\_\_Cyanobacteria;D\_3\_\_uncultured | 0.0% | 0.0% | 0.0% | 0.0% | 0.0% | 0.0% | 0.0% | 0.0% | 0.0% |
|  | D\_0\_\_Bacteria;D\_1\_\_Cyanobacteria;D\_2\_\_Melainabacteria;D\_3\_\_Gastranaerophilales | 0.0% | 0.0% | 0.0% | 0.0% | 0.0% | 0.0% | 0.0% | 0.0% | 0.0% |
|  | D\_0\_\_Bacteria;D\_1\_\_Cyanobacteria;D\_2\_\_Melainabacteria;D\_3\_\_Vampirovibrionales | 0.0% | 0.0% | 0.0% | 0.0% | 0.0% | 0.0% | 0.0% | 0.0% | 0.0% |
|  | D\_0\_\_Bacteria;D\_1\_\_Deinococcus-Thermus;D\_2\_\_Deinococci;D\_3\_\_Deinococcales | 0.0% | 0.0% | 0.0% | 0.0% | 0.0% | 0.0% | 0.0% | 0.0% | 0.1% |
|  | D\_0\_\_Bacteria;D\_1\_\_Deinococcus-Thermus;D\_2\_\_Deinococci;D\_3\_\_Thermales | 0.0% | 0.0% | 0.0% | 0.0% | 0.0% | 0.0% | 0.0% | 0.0% | 0.0% |
|  | D\_0\_\_Bacteria;D\_1\_\_Elusimicrobia;D\_2\_\_Elusimicrobia;D\_3\_\_Lineage IIa | 0.0% | 0.0% | 0.0% | 0.0% | 0.0% | 0.0% | 0.0% | 0.0% | 0.0% |
|  | D\_0\_\_Bacteria;D\_1\_\_Elusimicrobia;D\_2\_\_Elusimicrobia;D\_3\_\_Lineage IIb | 0.0% | 0.0% | 0.0% | 0.0% | 0.0% | 0.0% | 0.0% | 0.0% | 0.0% |
|  | D\_0\_\_Bacteria;D\_1\_\_Elusimicrobia;D\_2\_\_Elusimicrobia;D\_3\_\_Lineage IV | 0.0% | 0.0% | 0.0% | 0.0% | 0.0% | 0.0% | 0.0% | 0.0% | 0.0% |
|  | D\_0\_\_Bacteria;D\_1\_\_FBP;D\_2\_\_uncultured bacterium;D\_3\_\_uncultured bacterium | 0.0% | 0.0% | 0.0% | 0.0% | 0.0% | 0.0% | 0.0% | 0.0% | 0.1% |
|  | D\_0\_\_Bacteria;D\_1\_\_Fibrobacteres;D\_2\_\_Fibrobacteria;D\_3\_\_Fibrobacterales | 0.0% | 0.0% | 0.0% | 0.0% | 0.0% | 0.0% | 0.0% | 0.0% | 0.0% |
|  | D\_0\_\_Bacteria;D\_1\_\_Firmicutes;D\_2\_\_Bacilli;D\_3\_\_Bacillales | 0.1% | 0.0% | 0.0% | 0.1% | 0.1% | 0.1% | 0.0% | 0.1% | 0.4% |
|  | D\_0\_\_Bacteria;D\_1\_\_Firmicutes;D\_2\_\_Bacilli;D\_3\_\_Lactobacillales | 0.1% | 0.0% | 0.0% | 0.0% | 0.0% | 0.0% | 0.0% | 0.1% | 0.8% |
|  | D\_0\_\_Bacteria;D\_1\_\_Firmicutes;D\_2\_\_Clostridia;D\_3\_\_Clostridiales | 0.1% | 0.0% | 0.0% | 0.0% | 0.0% | 0.0% | 0.0% | 0.0% | 0.5% |
|  | D\_0\_\_Bacteria;D\_1\_\_Firmicutes;D\_2\_\_Clostridia;D\_3\_\_Halanaerobiales | 0.0% | 0.0% | 0.0% | 0.0% | 0.0% | 0.0% | 0.0% | 0.0% | 0.0% |
|  | D\_0\_\_Bacteria;D\_1\_\_Firmicutes;D\_2\_\_Clostridia;D\_3\_\_Thermoanaerobacterales | 0.0% | 0.0% | 0.0% | 0.0% | 0.0% | 0.0% | 0.0% | 0.0% | 0.0% |
|  | D\_0\_\_Bacteria;D\_1\_\_Firmicutes;D\_2\_\_Erysipelotrichia;D\_3\_\_Erysipelotrichales | 0.0% | 0.0% | 0.0% | 0.0% | 0.0% | 0.0% | 0.0% | 0.0% | 0.1% |
|  | D\_0\_\_Bacteria;D\_1\_\_Firmicutes;D\_2\_\_Negativicutes;D\_3\_\_Selenomonadales | 0.0% | 0.0% | 0.0% | 0.0% | 0.0% | 0.0% | 0.0% | 0.0% | 0.0% |
|  | D\_0\_\_Bacteria;D\_1\_\_Fusobacteria;D\_2\_\_Fusobacteriia;D\_3\_\_Fusobacteriales | 0.1% | 0.0% | 0.0% | 0.0% | 0.0% | 0.0% | 0.0% | 0.1% | 0.5% |
|  | D\_0\_\_Bacteria;D\_1\_\_Gemmatimonadetes;D\_2\_\_BD2-11 terrestrial group;D\_3\_\_uncultured bacterium | 0.0% | 0.0% | 0.0% | 0.0% | 0.0% | 0.0% | 0.0% | 0.0% | 0.0% |
|  | D\_0\_\_Bacteria;D\_1\_\_Gemmatimonadetes;D\_2\_\_Gemmatimonadetes;D\_3\_\_Gemmatimonadales | 0.0% | 0.0% | 0.0% | 0.0% | 0.0% | 0.0% | 0.0% | 0.0% | 0.0% |
|  | D\_0\_\_Bacteria;D\_1\_\_Gemmatimonadetes;D\_2\_\_Longimicrobia;D\_3\_\_Longimicrobiales | 0.3% | 0.3% | 0.3% | 0.2% | 0.2% | 0.8% | 0.2% | 0.1% | 0.1% |
|  | D\_0\_\_Bacteria;D\_1\_\_Gemmatimonadetes;D\_2\_\_S0134 terrestrial group;D\_3\_\_uncultured Gemmatimonadetes bacterium | 0.0% | 0.0% | 0.0% | 0.0% | 0.0% | 0.0% | 0.0% | 0.0% | 0.0% |
|  | D\_0\_\_Bacteria;D\_1\_\_Gemmatimonadetes;D\_2\_\_S0134 terrestrial group;D\_3\_\_uncultured bacterium | 0.0% | 0.0% | 0.0% | 0.0% | 0.0% | 0.0% | 0.0% | 0.0% | 0.1% |
|  | D\_0\_\_Bacteria;D\_1\_\_Hydrogenedentes;D\_2\_\_uncultured bacterium;D\_3\_\_uncultured bacterium | 0.0% | 0.0% | 0.0% | 0.0% | 0.0% | 0.0% | 0.0% | 0.0% | 0.0% |
|  | D\_0\_\_Bacteria;D\_1\_\_Nitrospirae;D\_2\_\_Nitrospira;D\_3\_\_Nitrospirales | 0.2% | 0.5% | 0.4% | 0.2% | 0.2% | 0.3% | 0.1% | 0.1% | 0.2% |
|  | D\_0\_\_Bacteria;D\_1\_\_Planctomycetes;D\_2\_\_Phycisphaerae;D\_3\_\_Phycisphaerales | 0.0% | 0.0% | 0.0% | 0.0% | 0.0% | 0.0% | 0.0% | 0.0% | 0.0% |
|  | D\_0\_\_Bacteria;D\_1\_\_Planctomycetes;D\_2\_\_Phycisphaerae;D\_3\_\_Tepidisphaerales | 0.0% | 0.0% | 0.0% | 0.0% | 0.0% | 0.0% | 0.0% | 0.0% | 0.0% |
|  | D\_0\_\_Bacteria;D\_1\_\_Planctomycetes;D\_2\_\_Planctomycetacia;D\_3\_\_Planctomycetales | 0.1% | 0.1% | 0.1% | 0.2% | 0.2% | 0.0% | 0.0% | 0.1% | 0.1% |
|  | D\_0\_\_Bacteria;D\_1\_\_Proteobacteria;D\_2\_\_Alphaproteobacteria;D\_3\_\_Alphaproteobacteria Incertae Sedis | 0.0% | 0.0% | 0.0% | 0.0% | 0.0% | 0.0% | 0.0% | 0.0% | 0.0% |
|  | D\_0\_\_Bacteria;D\_1\_\_Proteobacteria;D\_2\_\_Alphaproteobacteria;D\_3\_\_Caulobacterales | 0.1% | 0.2% | 0.2% | 0.1% | 0.1% | 0.1% | 0.0% | 0.1% | 0.1% |
|  | D\_0\_\_Bacteria;D\_1\_\_Proteobacteria;D\_2\_\_Alphaproteobacteria;D\_3\_\_Rhizobiales | 1.8% | 1.9% | 1.6% | 1.9% | 1.9% | 2.7% | 2.8% | 0.6% | 1.4% |
|  | D\_0\_\_Bacteria;D\_1\_\_Proteobacteria;D\_2\_\_Alphaproteobacteria;D\_3\_\_Rhodobacterales | 0.1% | 0.0% | 0.0% | 0.0% | 0.0% | 0.0% | 0.0% | 0.2% | 0.6% |
|  | D\_0\_\_Bacteria;D\_1\_\_Proteobacteria;D\_2\_\_Alphaproteobacteria;D\_3\_\_Rhodospirillales | 3.1% | 5.2% | 4.4% | 4.7% | 4.8% | 3.2% | 1.8% | 0.4% | 0.7% |
|  | D\_0\_\_Bacteria;D\_1\_\_Proteobacteria;D\_2\_\_Alphaproteobacteria;D\_3\_\_Rickettsiales | 0.0% | 0.0% | 0.0% | 0.0% | 0.0% | 0.0% | 0.0% | 0.0% | 0.1% |
|  | D\_0\_\_Bacteria;D\_1\_\_Proteobacteria;D\_2\_\_Alphaproteobacteria;D\_3\_\_Sphingomonadales | 0.0% | 0.0% | 0.0% | 0.1% | 0.1% | 0.0% | 0.0% | 0.0% | 0.0% |
|  | D\_0\_\_Bacteria;D\_1\_\_Proteobacteria;D\_2\_\_Alphaproteobacteria;Other | 0.0% | 0.0% | 0.0% | 0.0% | 0.0% | 0.0% | 0.0% | 0.0% | 0.0% |
|  | D\_0\_\_Bacteria;D\_1\_\_Proteobacteria;D\_2\_\_Betaproteobacteria;D\_3\_\_Burkholderiales | 0.1% | 0.0% | 0.0% | 0.0% | 0.0% | 0.0% | 0.0% | 0.1% | 0.5% |
|  | D\_0\_\_Bacteria;D\_1\_\_Proteobacteria;D\_2\_\_Betaproteobacteria;D\_3\_\_Hydrogenophilales | 0.0% | 0.0% | 0.0% | 0.0% | 0.0% | 0.3% | 0.1% | 0.0% | 0.0% |
|  | D\_0\_\_Bacteria;D\_1\_\_Proteobacteria;D\_2\_\_Betaproteobacteria;D\_3\_\_Methylophilales | 0.0% | 0.0% | 0.0% | 0.0% | 0.0% | 0.0% | 0.0% | 0.0% | 0.0% |
|  | D\_0\_\_Bacteria;D\_1\_\_Proteobacteria;D\_2\_\_Betaproteobacteria;D\_3\_\_Neisseriales | 0.1% | 0.0% | 0.0% | 0.0% | 0.0% | 0.0% | 0.0% | 0.1% | 0.8% |
|  | D\_0\_\_Bacteria;D\_1\_\_Proteobacteria;D\_2\_\_Betaproteobacteria;D\_3\_\_Nitrosomonadales | 0.0% | 0.0% | 0.0% | 0.0% | 0.0% | 0.2% | 0.0% | 0.0% | 0.0% |
|  | D\_0\_\_Bacteria;D\_1\_\_Proteobacteria;D\_2\_\_Betaproteobacteria;D\_3\_\_Rhodocyclales | 0.0% | 0.0% | 0.0% | 0.0% | 0.0% | 0.0% | 0.0% | 0.0% | 0.0% |
|  | D\_0\_\_Bacteria;D\_1\_\_Proteobacteria;D\_2\_\_Betaproteobacteria;D\_3\_\_SC-I-84 | 0.0% | 0.0% | 0.0% | 0.0% | 0.0% | 0.0% | 0.0% | 0.0% | 0.0% |
|  | D\_0\_\_Bacteria;D\_1\_\_Proteobacteria;D\_2\_\_Betaproteobacteria;D\_3\_\_TRA3-20 | 1.7% | 3.1% | 2.9% | 3.7% | 3.3% | 0.2% | 0.0% | 0.1% | 0.1% |
|  | D\_0\_\_Bacteria;D\_1\_\_Proteobacteria;D\_2\_\_Betaproteobacteria;Other | 0.0% | 0.0% | 0.0% | 0.0% | 0.0% | 0.0% | 0.0% | 0.0% | 0.0% |
|  | D\_0\_\_Bacteria;D\_1\_\_Proteobacteria;D\_2\_\_Deltaproteobacteria;D\_3\_\_Bdellovibrionales | 0.0% | 0.0% | 0.0% | 0.0% | 0.0% | 0.0% | 0.0% | 0.0% | 0.0% |
|  | D\_0\_\_Bacteria;D\_1\_\_Proteobacteria;D\_2\_\_Deltaproteobacteria;D\_3\_\_Desulfurellales | 4.2% | 8.1% | 7.9% | 7.0% | 7.9% | 1.1% | 0.6% | 0.3% | 0.6% |
|  | D\_0\_\_Bacteria;D\_1\_\_Proteobacteria;D\_2\_\_Deltaproteobacteria;D\_3\_\_Myxococcales | 0.0% | 0.0% | 0.0% | 0.0% | 0.0% | 0.0% | 0.0% | 0.0% | 0.0% |
|  | D\_0\_\_Bacteria;D\_1\_\_Proteobacteria;D\_2\_\_Deltaproteobacteria;D\_3\_\_Oligoflexales | 0.0% | 0.0% | 0.0% | 0.0% | 0.0% | 0.0% | 0.0% | 0.0% | 0.0% |
|  | D\_0\_\_Bacteria;D\_1\_\_Proteobacteria;D\_2\_\_Deltaproteobacteria;D\_3\_\_SAR324 clade(Marine group B) | 0.0% | 0.0% | 0.0% | 0.0% | 0.0% | 0.0% | 0.0% | 0.0% | 0.0% |
|  | D\_0\_\_Bacteria;D\_1\_\_Proteobacteria;D\_2\_\_Epsilonproteobacteria;D\_3\_\_Campylobacterales | 0.0% | 0.0% | 0.0% | 0.0% | 0.0% | 0.0% | 0.0% | 0.0% | 0.0% |
|  | D\_0\_\_Bacteria;D\_1\_\_Proteobacteria;D\_2\_\_Gammaproteobacteria;D\_3\_\_Alteromonadales | 0.0% | 0.0% | 0.0% | 0.0% | 0.0% | 0.0% | 0.0% | 0.0% | 0.0% |
|  | D\_0\_\_Bacteria;D\_1\_\_Proteobacteria;D\_2\_\_Gammaproteobacteria;D\_3\_\_Cardiobacteriales | 0.0% | 0.0% | 0.0% | 0.0% | 0.0% | 0.0% | 0.0% | 0.0% | 0.0% |
|  | D\_0\_\_Bacteria;D\_1\_\_Proteobacteria;D\_2\_\_Gammaproteobacteria;D\_3\_\_Cellvibrionales | 0.0% | 0.0% | 0.0% | 0.0% | 0.0% | 0.0% | 0.0% | 0.0% | 0.0% |
|  | D\_0\_\_Bacteria;D\_1\_\_Proteobacteria;D\_2\_\_Gammaproteobacteria;D\_3\_\_Chromatiales | 0.0% | 0.0% | 0.0% | 0.0% | 0.0% | 0.0% | 0.0% | 0.0% | 0.0% |
|  | D\_0\_\_Bacteria;D\_1\_\_Proteobacteria;D\_2\_\_Gammaproteobacteria;D\_3\_\_Enterobacteriales | 0.0% | 0.0% | 0.0% | 0.0% | 0.0% | 0.1% | 0.0% | 0.0% | 0.1% |
|  | D\_0\_\_Bacteria;D\_1\_\_Proteobacteria;D\_2\_\_Gammaproteobacteria;D\_3\_\_Gammaproteobacteria Incertae Sedis | 0.0% | 0.0% | 0.0% | 0.0% | 0.0% | 0.0% | 0.0% | 0.0% | 0.0% |
|  | D\_0\_\_Bacteria;D\_1\_\_Proteobacteria;D\_2\_\_Gammaproteobacteria;D\_3\_\_HTA4 | 0.0% | 0.0% | 0.0% | 0.0% | 0.0% | 0.0% | 0.0% | 0.0% | 0.0% |
|  | D\_0\_\_Bacteria;D\_1\_\_Proteobacteria;D\_2\_\_Gammaproteobacteria;D\_3\_\_KI89A clade | 0.0% | 0.0% | 0.0% | 0.0% | 0.0% | 0.0% | 0.0% | 0.0% | 0.0% |
|  | D\_0\_\_Bacteria;D\_1\_\_Proteobacteria;D\_2\_\_Gammaproteobacteria;D\_3\_\_Legionellales | 0.0% | 0.0% | 0.0% | 0.0% | 0.0% | 0.0% | 0.0% | 0.0% | 0.0% |
|  | D\_0\_\_Bacteria;D\_1\_\_Proteobacteria;D\_2\_\_Gammaproteobacteria;D\_3\_\_Oceanospirillales | 0.0% | 0.0% | 0.0% | 0.0% | 0.0% | 0.0% | 0.0% | 0.0% | 0.0% |
|  | D\_0\_\_Bacteria;D\_1\_\_Proteobacteria;D\_2\_\_Gammaproteobacteria;D\_3\_\_PYR10d3 | 0.0% | 0.0% | 0.0% | 0.0% | 0.0% | 0.0% | 0.0% | 0.0% | 0.0% |
|  | D\_0\_\_Bacteria;D\_1\_\_Proteobacteria;D\_2\_\_Gammaproteobacteria;D\_3\_\_Pasteurellales | 0.1% | 0.0% | 0.0% | 0.0% | 0.0% | 0.0% | 0.0% | 0.0% | 0.5% |
|  | D\_0\_\_Bacteria;D\_1\_\_Proteobacteria;D\_2\_\_Gammaproteobacteria;D\_3\_\_Pseudomonadales | 0.1% | 0.0% | 0.0% | 0.0% | 0.0% | 0.0% | 0.0% | 0.1% | 0.8% |
|  | D\_0\_\_Bacteria;D\_1\_\_Proteobacteria;D\_2\_\_Gammaproteobacteria;D\_3\_\_Thiotrichales | 0.0% | 0.0% | 0.0% | 0.0% | 0.0% | 0.0% | 0.0% | 0.0% | 0.0% |
|  | D\_0\_\_Bacteria;D\_1\_\_Proteobacteria;D\_2\_\_Gammaproteobacteria;D\_3\_\_Vibrionales | 0.0% | 0.0% | 0.0% | 0.0% | 0.0% | 0.0% | 0.0% | 0.0% | 0.0% |
|  | D\_0\_\_Bacteria;D\_1\_\_Proteobacteria;D\_2\_\_Gammaproteobacteria;D\_3\_\_X35 | 0.0% | 0.0% | 0.0% | 0.0% | 0.0% | 0.0% | 0.0% | 0.0% | 0.0% |
|  | D\_0\_\_Bacteria;D\_1\_\_Proteobacteria;D\_2\_\_Gammaproteobacteria;D\_3\_\_Xanthomonadales | 0.1% | 0.0% | 0.0% | 0.1% | 0.1% | 0.1% | 0.0% | 0.1% | 0.2% |
|  | D\_0\_\_Bacteria;D\_1\_\_Proteobacteria;D\_2\_\_Gammaproteobacteria;D\_3\_\_uncultured | 0.0% | 0.0% | 0.0% | 0.0% | 0.0% | 0.0% | 0.0% | 0.0% | 0.0% |
|  | D\_0\_\_Bacteria;D\_1\_\_Proteobacteria;D\_2\_\_Gammaproteobacteria;Other | 0.1% | 0.0% | 0.0% | 0.0% | 0.0% | 0.0% | 0.0% | 0.1% | 0.9% |
|  | D\_0\_\_Bacteria;D\_1\_\_Proteobacteria;D\_2\_\_JTB23;D\_3\_\_uncultured bacterium | 0.0% | 0.0% | 0.0% | 0.0% | 0.0% | 0.0% | 0.0% | 0.0% | 0.0% |
|  | D\_0\_\_Bacteria;D\_1\_\_Proteobacteria;D\_2\_\_JTB23;Other | 0.0% | 0.0% | 0.0% | 0.0% | 0.0% | 0.0% | 0.0% | 0.0% | 0.0% |
|  | D\_0\_\_Bacteria;D\_1\_\_Proteobacteria;D\_2\_\_SPOTSOCT00m83;Ambiguous\_taxa | 0.0% | 0.0% | 0.0% | 0.0% | 0.0% | 0.0% | 0.0% | 0.0% | 0.0% |
|  | D\_0\_\_Bacteria;D\_1\_\_Proteobacteria;D\_2\_\_SPOTSOCT00m83;Other | 0.0% | 0.0% | 0.0% | 0.0% | 0.0% | 0.0% | 0.0% | 0.0% | 0.0% |
|  | D\_0\_\_Bacteria;D\_1\_\_Proteobacteria;Other;Other | 0.0% | 0.0% | 0.0% | 0.0% | 0.0% | 0.0% | 0.0% | 0.0% | 0.0% |
|  | D\_0\_\_Bacteria;D\_1\_\_SBR1093;Other;Other | 0.0% | 0.0% | 0.0% | 0.0% | 0.0% | 0.0% | 0.0% | 0.0% | 0.0% |
|  | D\_0\_\_Bacteria;D\_1\_\_SR1 (Absconditabacteria);Ambiguous\_taxa;Ambiguous\_taxa | 0.0% | 0.0% | 0.0% | 0.0% | 0.0% | 0.0% | 0.0% | 0.0% | 0.0% |
|  | D\_0\_\_Bacteria;D\_1\_\_SR1 (Absconditabacteria);D\_2\_\_uncultured bacterium;D\_3\_\_uncultured bacterium | 0.0% | 0.0% | 0.0% | 0.0% | 0.0% | 0.0% | 0.0% | 0.0% | 0.1% |
|  | D\_0\_\_Bacteria;D\_1\_\_Saccharibacteria;D\_2\_\_uncultured bacterium;D\_3\_\_uncultured bacterium | 0.0% | 0.0% | 0.0% | 0.0% | 0.0% | 0.0% | 0.0% | 0.0% | 0.0% |
|  | D\_0\_\_Bacteria;D\_1\_\_Saccharibacteria;Other;Other | 0.0% | 0.0% | 0.0% | 0.0% | 0.0% | 0.0% | 0.0% | 0.0% | 0.0% |
|  | D\_0\_\_Bacteria;D\_1\_\_Spirochaetae;D\_2\_\_Spirochaetes;D\_3\_\_Spirochaetales | 0.0% | 0.0% | 0.0% | 0.0% | 0.0% | 0.0% | 0.0% | 0.0% | 0.1% |
|  | D\_0\_\_Bacteria;D\_1\_\_Synergistetes;D\_2\_\_Synergistia;D\_3\_\_Synergistales | 0.0% | 0.0% | 0.0% | 0.0% | 0.0% | 0.0% | 0.0% | 0.0% | 0.0% |
|  | D\_0\_\_Bacteria;D\_1\_\_TM6 (Dependentiae);Other;Other | 0.0% | 0.0% | 0.0% | 0.0% | 0.0% | 0.0% | 0.0% | 0.0% | 0.0% |
|  | D\_0\_\_Bacteria;D\_1\_\_Tectomicrobia;D\_2\_\_Tectomicrobia Incertae Sedis;D\_3\_\_Unknown Order | 0.0% | 0.0% | 0.0% | 0.0% | 0.0% | 0.0% | 0.0% | 0.0% | 0.0% |
|  | D\_0\_\_Bacteria;D\_1\_\_Tectomicrobia;D\_2\_\_uncultured bacterium;D\_3\_\_uncultured bacterium | 0.0% | 0.0% | 0.0% | 0.0% | 0.0% | 0.0% | 0.0% | 0.0% | 0.0% |
|  | D\_0\_\_Bacteria;D\_1\_\_Verrucomicrobia;D\_2\_\_OPB35 soil group;Ambiguous\_taxa | 0.0% | 0.0% | 0.0% | 0.0% | 0.0% | 0.0% | 0.0% | 0.0% | 0.0% |
|  | D\_0\_\_Bacteria;D\_1\_\_Verrucomicrobia;D\_2\_\_OPB35 soil group;D\_3\_\_uncultured bacterium | 0.0% | 0.0% | 0.0% | 0.0% | 0.0% | 0.0% | 0.0% | 0.0% | 0.0% |
|  | D\_0\_\_Bacteria;D\_1\_\_Verrucomicrobia;D\_2\_\_OPB35 soil group;Other | 0.0% | 0.0% | 0.0% | 0.0% | 0.0% | 0.0% | 0.0% | 0.0% | 0.0% |
|  | D\_0\_\_Bacteria;D\_1\_\_Verrucomicrobia;D\_2\_\_Opitutae;D\_3\_\_BC-COM435 | 0.0% | 0.0% | 0.0% | 0.0% | 0.0% | 0.0% | 0.0% | 0.0% | 0.0% |
|  | D\_0\_\_Bacteria;D\_1\_\_Verrucomicrobia;D\_2\_\_Opitutae;D\_3\_\_Opitutales | 0.0% | 0.0% | 0.0% | 0.0% | 0.0% | 0.0% | 0.0% | 0.0% | 0.0% |
|  | D\_0\_\_Bacteria;D\_1\_\_Verrucomicrobia;D\_2\_\_Spartobacteria;D\_3\_\_Chthoniobacterales | 0.0% | 0.0% | 0.0% | 0.0% | 0.0% | 0.0% | 0.0% | 0.0% | 0.0% |
|  | D\_0\_\_Bacteria;D\_1\_\_Verrucomicrobia;D\_2\_\_Verrucomicrobiae;D\_3\_\_Verrucomicrobiales | 0.0% | 0.0% | 0.0% | 0.0% | 0.0% | 0.0% | 0.0% | 0.0% | 0.0% |
|  | D\_0\_\_Bacteria;Other;Other;Other | 0.0% | 0.0% | 0.0% | 0.0% | 0.0% | 0.0% | 0.0% | 0.0% | 0.0% |
|  | Unassigned;Other;Other;Other | 0.3% | 0.4% | 0.4% | 0.3% | 0.3% | 0.4% | 0.1% | 0.2% | 0.2% |

|  |  |
| --- | --- |
|  | |
| Taxonomy Summary. Current Level: | |
| View Figure (.pdf)  View Legend (.pdf) |  |
|  |


|  |
| --- |
| View Table (.txt) |

|  |  |  |  |  |  |  |  |  |  |  |
| --- | --- | --- | --- | --- | --- | --- | --- | --- | --- | --- |
|  | | Total | 1B5g1 | 1B5g2 | 1BEDTA1 | 1BEDTA2 | 1M1 | 1M2 | 1S1 | 1S2 |
| Legend | Taxonomy | % | % | % | % | % | % | % | % | % |
|  | D\_0\_\_Archaea;D\_1\_\_Thaumarchaeota;D\_2\_\_Soil Crenarchaeotic Group(SCG);D\_3\_\_Unknown Order;D\_4\_\_Unknown Family | 0.0% | 0.0% | 0.0% | 0.0% | 0.0% | 0.0% | 0.0% | 0.0% | 0.0% |
|  | D\_0\_\_Archaea;D\_1\_\_Thaumarchaeota;D\_2\_\_Soil Crenarchaeotic Group(SCG);D\_3\_\_uncultured archaeon;D\_4\_\_uncultured archaeon | 0.0% | 0.0% | 0.0% | 0.0% | 0.0% | 0.0% | 0.0% | 0.0% | 0.0% |
|  | D\_0\_\_Archaea;D\_1\_\_Thaumarchaeota;D\_2\_\_Soil Crenarchaeotic Group(SCG);D\_3\_\_uncultured euryarchaeote;D\_4\_\_uncultured euryarchaeote | 0.0% | 0.0% | 0.0% | 0.0% | 0.0% | 0.0% | 0.0% | 0.0% | 0.0% |
|  | D\_0\_\_Archaea;D\_1\_\_Thaumarchaeota;D\_2\_\_Soil Crenarchaeotic Group(SCG);Other;Other | 0.6% | 0.7% | 0.7% | 0.4% | 0.5% | 1.0% | 0.8% | 0.2% | 0.3% |
|  | D\_0\_\_Bacteria;D\_1\_\_Acidobacteria;D\_2\_\_Blastocatellia;D\_3\_\_Blastocatellales;D\_4\_\_Blastocatellaceae (Subgroup 4) | 0.0% | 0.0% | 0.0% | 0.0% | 0.0% | 0.0% | 0.0% | 0.0% | 0.0% |
|  | D\_0\_\_Bacteria;D\_1\_\_Acidobacteria;D\_2\_\_Holophagae;D\_3\_\_Holophagales;D\_4\_\_Holophagaceae | 0.0% | 0.0% | 0.0% | 0.0% | 0.0% | 0.0% | 0.0% | 0.0% | 0.0% |
|  | D\_0\_\_Bacteria;D\_1\_\_Acidobacteria;D\_2\_\_Holophagae;D\_3\_\_Subgroup 10;D\_4\_\_CA002 | 0.0% | 0.0% | 0.0% | 0.0% | 0.0% | 0.0% | 0.0% | 0.0% | 0.0% |
|  | D\_0\_\_Bacteria;D\_1\_\_Acidobacteria;D\_2\_\_Holophagae;D\_3\_\_Subgroup 10;D\_4\_\_NS72 | 0.0% | 0.0% | 0.0% | 0.0% | 0.0% | 0.1% | 0.0% | 0.0% | 0.0% |
|  | D\_0\_\_Bacteria;D\_1\_\_Acidobacteria;D\_2\_\_Holophagae;D\_3\_\_Subgroup 10;D\_4\_\_Sva0725 | 0.0% | 0.0% | 0.0% | 0.0% | 0.0% | 0.0% | 0.0% | 0.0% | 0.0% |
|  | D\_0\_\_Bacteria;D\_1\_\_Acidobacteria;D\_2\_\_Holophagae;D\_3\_\_Subgroup 10;Other | 0.0% | 0.0% | 0.0% | 0.0% | 0.0% | 0.0% | 0.0% | 0.0% | 0.0% |
|  | D\_0\_\_Bacteria;D\_1\_\_Acidobacteria;D\_2\_\_Holophagae;D\_3\_\_Subgroup 7;Ambiguous\_taxa | 0.0% | 0.0% | 0.0% | 0.0% | 0.0% | 0.0% | 0.0% | 0.0% | 0.0% |
|  | D\_0\_\_Bacteria;D\_1\_\_Acidobacteria;D\_2\_\_Holophagae;D\_3\_\_Subgroup 7;D\_4\_\_uncultured bacterium | 0.0% | 0.0% | 0.0% | 0.0% | 0.0% | 0.0% | 0.0% | 0.0% | 0.0% |
|  | D\_0\_\_Bacteria;D\_1\_\_Acidobacteria;D\_2\_\_Holophagae;D\_3\_\_Subgroup 7;Other | 0.0% | 0.0% | 0.0% | 0.0% | 0.0% | 0.0% | 0.0% | 0.0% | 0.0% |
|  | D\_0\_\_Bacteria;D\_1\_\_Acidobacteria;D\_2\_\_Solibacteres;D\_3\_\_Solibacterales;D\_4\_\_Solibacteraceae (Subgroup 3) | 0.0% | 0.0% | 0.0% | 0.0% | 0.0% | 0.0% | 0.0% | 0.0% | 0.0% |
|  | D\_0\_\_Bacteria;D\_1\_\_Acidobacteria;D\_2\_\_Subgroup 17;Ambiguous\_taxa;Ambiguous\_taxa | 0.0% | 0.0% | 0.0% | 0.0% | 0.0% | 0.0% | 0.0% | 0.0% | 0.0% |
|  | D\_0\_\_Bacteria;D\_1\_\_Acidobacteria;D\_2\_\_Subgroup 17;D\_3\_\_uncultured Acidobacteria bacterium;D\_4\_\_uncultured Acidobacteria bacterium | 0.0% | 0.0% | 0.0% | 0.0% | 0.0% | 0.0% | 0.0% | 0.0% | 0.0% |
|  | D\_0\_\_Bacteria;D\_1\_\_Acidobacteria;D\_2\_\_Subgroup 17;Other;Other | 2.5% | 3.3% | 2.9% | 3.0% | 3.0% | 2.8% | 1.1% | 2.5% | 1.6% |
|  | D\_0\_\_Bacteria;D\_1\_\_Acidobacteria;D\_2\_\_Subgroup 22;Ambiguous\_taxa;Ambiguous\_taxa | 0.0% | 0.0% | 0.0% | 0.0% | 0.0% | 0.0% | 0.0% | 0.0% | 0.0% |
|  | D\_0\_\_Bacteria;D\_1\_\_Acidobacteria;D\_2\_\_Subgroup 22;D\_3\_\_uncultured Acidobacteria bacterium;D\_4\_\_uncultured Acidobacteria bacterium | 0.0% | 0.0% | 0.0% | 0.0% | 0.0% | 0.0% | 0.0% | 0.0% | 0.0% |
|  | D\_0\_\_Bacteria;D\_1\_\_Acidobacteria;D\_2\_\_Subgroup 22;D\_3\_\_uncultured bacterium;D\_4\_\_uncultured bacterium | 0.0% | 0.0% | 0.0% | 0.0% | 0.0% | 0.0% | 0.0% | 0.0% | 0.0% |
|  | D\_0\_\_Bacteria;D\_1\_\_Acidobacteria;D\_2\_\_Subgroup 22;Other;Other | 0.0% | 0.0% | 0.0% | 0.0% | 0.0% | 0.0% | 0.0% | 0.0% | 0.0% |
|  | D\_0\_\_Bacteria;D\_1\_\_Acidobacteria;D\_2\_\_Subgroup 5;D\_3\_\_uncultured bacterium;D\_4\_\_uncultured bacterium | 0.0% | 0.0% | 0.0% | 0.0% | 0.0% | 0.0% | 0.0% | 0.0% | 0.0% |
|  | D\_0\_\_Bacteria;D\_1\_\_Acidobacteria;D\_2\_\_Subgroup 5;Other;Other | 0.0% | 0.0% | 0.0% | 0.0% | 0.0% | 0.0% | 0.0% | 0.0% | 0.0% |
|  | D\_0\_\_Bacteria;D\_1\_\_Acidobacteria;D\_2\_\_Subgroup 6;Ambiguous\_taxa;Ambiguous\_taxa | 0.1% | 0.1% | 0.1% | 0.1% | 0.1% | 0.1% | 0.0% | 0.0% | 0.0% |
|  | D\_0\_\_Bacteria;D\_1\_\_Acidobacteria;D\_2\_\_Subgroup 6;D\_3\_\_uncultured Acidobacteria bacterium;D\_4\_\_uncultured Acidobacteria bacterium | 0.0% | 0.0% | 0.0% | 0.0% | 0.0% | 0.0% | 0.0% | 0.0% | 0.0% |
|  | D\_0\_\_Bacteria;D\_1\_\_Acidobacteria;D\_2\_\_Subgroup 6;D\_3\_\_uncultured bacterium;D\_4\_\_uncultured bacterium | 0.2% | 0.5% | 0.3% | 0.3% | 0.3% | 0.0% | 0.0% | 0.0% | 0.0% |
|  | D\_0\_\_Bacteria;D\_1\_\_Acidobacteria;D\_2\_\_Subgroup 6;D\_3\_\_uncultured organism;D\_4\_\_uncultured organism | 0.0% | 0.0% | 0.0% | 0.0% | 0.0% | 0.0% | 0.0% | 0.0% | 0.0% |
|  | D\_0\_\_Bacteria;D\_1\_\_Acidobacteria;D\_2\_\_Subgroup 6;D\_3\_\_uncultured soil bacterium;D\_4\_\_uncultured soil bacterium | 0.0% | 0.0% | 0.0% | 0.0% | 0.0% | 0.0% | 0.0% | 0.0% | 0.0% |
|  | D\_0\_\_Bacteria;D\_1\_\_Acidobacteria;D\_2\_\_Subgroup 6;Other;Other | 0.0% | 0.0% | 0.0% | 0.0% | 0.0% | 0.0% | 0.0% | 0.0% | 0.0% |
|  | D\_0\_\_Bacteria;D\_1\_\_Acidobacteria;Other;Other;Other | 0.0% | 0.0% | 0.0% | 0.0% | 0.0% | 0.0% | 0.0% | 0.0% | 0.0% |
|  | D\_0\_\_Bacteria;D\_1\_\_Actinobacteria;D\_2\_\_Acidimicrobiia;D\_3\_\_Acidimicrobiales;D\_4\_\_Acidimicrobiaceae | 0.0% | 0.0% | 0.0% | 0.0% | 0.0% | 0.1% | 0.0% | 0.0% | 0.0% |
|  | D\_0\_\_Bacteria;D\_1\_\_Actinobacteria;D\_2\_\_Acidimicrobiia;D\_3\_\_Acidimicrobiales;D\_4\_\_Acidimicrobiales Incertae Sedis | 0.0% | 0.0% | 0.0% | 0.0% | 0.0% | 0.0% | 0.0% | 0.0% | 0.0% |
|  | D\_0\_\_Bacteria;D\_1\_\_Actinobacteria;D\_2\_\_Acidimicrobiia;D\_3\_\_Acidimicrobiales;D\_4\_\_Iamiaceae | 0.0% | 0.1% | 0.1% | 0.1% | 0.1% | 0.1% | 0.0% | 0.0% | 0.0% |
|  | D\_0\_\_Bacteria;D\_1\_\_Actinobacteria;D\_2\_\_Acidimicrobiia;D\_3\_\_Acidimicrobiales;D\_4\_\_OM1 clade | 2.2% | 4.2% | 4.4% | 2.7% | 3.2% | 1.7% | 0.2% | 0.7% | 0.6% |
|  | D\_0\_\_Bacteria;D\_1\_\_Actinobacteria;D\_2\_\_Acidimicrobiia;D\_3\_\_Acidimicrobiales;D\_4\_\_Sva0996 marine group | 0.0% | 0.0% | 0.0% | 0.0% | 0.0% | 0.0% | 0.0% | 0.0% | 0.0% |
|  | D\_0\_\_Bacteria;D\_1\_\_Actinobacteria;D\_2\_\_Acidimicrobiia;D\_3\_\_Acidimicrobiales;D\_4\_\_uncultured | 1.2% | 1.8% | 1.8% | 1.6% | 1.6% | 1.1% | 0.7% | 0.7% | 0.4% |
|  | D\_0\_\_Bacteria;D\_1\_\_Actinobacteria;D\_2\_\_Acidimicrobiia;D\_3\_\_Acidimicrobiales;Other | 0.0% | 0.0% | 0.0% | 0.0% | 0.0% | 0.0% | 0.0% | 0.0% | 0.0% |
|  | D\_0\_\_Bacteria;D\_1\_\_Actinobacteria;D\_2\_\_Actinobacteria;D\_3\_\_Actinomycetales;D\_4\_\_Actinomycetaceae | 0.0% | 0.0% | 0.0% | 0.0% | 0.0% | 0.0% | 0.0% | 0.0% | 0.3% |
|  | D\_0\_\_Bacteria;D\_1\_\_Actinobacteria;D\_2\_\_Actinobacteria;D\_3\_\_Actinopolysporales;D\_4\_\_Actinopolysporaceae | 0.0% | 0.0% | 0.0% | 0.0% | 0.0% | 0.0% | 0.0% | 0.0% | 0.0% |
|  | D\_0\_\_Bacteria;D\_1\_\_Actinobacteria;D\_2\_\_Actinobacteria;D\_3\_\_Bifidobacteriales;D\_4\_\_Bifidobacteriaceae | 0.0% | 0.0% | 0.0% | 0.0% | 0.0% | 0.0% | 0.0% | 0.0% | 0.0% |
|  | D\_0\_\_Bacteria;D\_1\_\_Actinobacteria;D\_2\_\_Actinobacteria;D\_3\_\_Corynebacteriales;D\_4\_\_Corynebacteriaceae | 0.1% | 0.0% | 0.0% | 0.0% | 0.0% | 0.0% | 0.0% | 0.1% | 0.6% |
|  | D\_0\_\_Bacteria;D\_1\_\_Actinobacteria;D\_2\_\_Actinobacteria;D\_3\_\_Corynebacteriales;D\_4\_\_Dietziaceae | 0.0% | 0.0% | 0.0% | 0.0% | 0.0% | 0.0% | 0.0% | 0.0% | 0.0% |
|  | D\_0\_\_Bacteria;D\_1\_\_Actinobacteria;D\_2\_\_Actinobacteria;D\_3\_\_Corynebacteriales;D\_4\_\_Mycobacteriaceae | 0.0% | 0.0% | 0.0% | 0.0% | 0.0% | 0.0% | 0.0% | 0.0% | 0.0% |
|  | D\_0\_\_Bacteria;D\_1\_\_Actinobacteria;D\_2\_\_Actinobacteria;D\_3\_\_Corynebacteriales;D\_4\_\_Nocardiaceae | 0.0% | 0.0% | 0.0% | 0.0% | 0.0% | 0.0% | 0.0% | 0.0% | 0.0% |
|  | D\_0\_\_Bacteria;D\_1\_\_Actinobacteria;D\_2\_\_Actinobacteria;D\_3\_\_Corynebacteriales;Other | 0.0% | 0.0% | 0.0% | 0.0% | 0.0% | 0.0% | 0.0% | 0.0% | 0.0% |
|  | D\_0\_\_Bacteria;D\_1\_\_Actinobacteria;D\_2\_\_Actinobacteria;D\_3\_\_Elev-16S-976;D\_4\_\_uncultured Nakamurellaceae bacterium | 0.0% | 0.0% | 0.0% | 0.0% | 0.0% | 0.0% | 0.0% | 0.0% | 0.0% |
|  | D\_0\_\_Bacteria;D\_1\_\_Actinobacteria;D\_2\_\_Actinobacteria;D\_3\_\_Frankiales;D\_4\_\_Acidothermaceae | 0.5% | 0.0% | 0.0% | 0.1% | 0.1% | 0.6% | 0.4% | 1.3% | 1.3% |
|  | D\_0\_\_Bacteria;D\_1\_\_Actinobacteria;D\_2\_\_Actinobacteria;D\_3\_\_Frankiales;D\_4\_\_Cryptosporangiaceae | 0.0% | 0.0% | 0.0% | 0.0% | 0.0% | 0.0% | 0.0% | 0.0% | 0.0% |
|  | D\_0\_\_Bacteria;D\_1\_\_Actinobacteria;D\_2\_\_Actinobacteria;D\_3\_\_Frankiales;D\_4\_\_Frankiaceae | 13.3% | 11.2% | 11.8% | 10.7% | 11.2% | 7.8% | 6.7% | 25.4% | 21.6% |
|  | D\_0\_\_Bacteria;D\_1\_\_Actinobacteria;D\_2\_\_Actinobacteria;D\_3\_\_Frankiales;D\_4\_\_Geodermatophilaceae | 0.0% | 0.0% | 0.0% | 0.0% | 0.0% | 0.0% | 0.0% | 0.1% | 0.0% |
|  | D\_0\_\_Bacteria;D\_1\_\_Actinobacteria;D\_2\_\_Actinobacteria;D\_3\_\_Frankiales;D\_4\_\_Nakamurellaceae | 0.0% | 0.0% | 0.0% | 0.0% | 0.0% | 0.1% | 0.0% | 0.1% | 0.0% |
|  | D\_0\_\_Bacteria;D\_1\_\_Actinobacteria;D\_2\_\_Actinobacteria;D\_3\_\_Frankiales;D\_4\_\_Sporichthyaceae | 0.0% | 0.0% | 0.0% | 0.0% | 0.0% | 0.0% | 0.0% | 0.0% | 0.0% |
|  | D\_0\_\_Bacteria;D\_1\_\_Actinobacteria;D\_2\_\_Actinobacteria;D\_3\_\_Frankiales;D\_4\_\_uncultured | 0.1% | 0.0% | 0.0% | 0.1% | 0.1% | 0.1% | 0.1% | 0.1% | 0.0% |
|  | D\_0\_\_Bacteria;D\_1\_\_Actinobacteria;D\_2\_\_Actinobacteria;D\_3\_\_Frankiales;Other | 0.0% | 0.0% | 0.0% | 0.0% | 0.0% | 0.1% | 0.1% | 0.0% | 0.0% |
|  | D\_0\_\_Bacteria;D\_1\_\_Actinobacteria;D\_2\_\_Actinobacteria;D\_3\_\_Glycomycetales;D\_4\_\_Glycomycetaceae | 0.0% | 0.0% | 0.0% | 0.0% | 0.0% | 0.0% | 0.0% | 0.0% | 0.0% |
|  | D\_0\_\_Bacteria;D\_1\_\_Actinobacteria;D\_2\_\_Actinobacteria;D\_3\_\_Kineosporiales;D\_4\_\_Kineosporiaceae | 0.0% | 0.0% | 0.0% | 0.0% | 0.0% | 0.0% | 0.0% | 0.0% | 0.0% |
|  | D\_0\_\_Bacteria;D\_1\_\_Actinobacteria;D\_2\_\_Actinobacteria;D\_3\_\_Micrococcales;D\_4\_\_Beutenbergiaceae | 0.0% | 0.0% | 0.0% | 0.0% | 0.0% | 0.0% | 0.0% | 0.0% | 0.0% |
|  | D\_0\_\_Bacteria;D\_1\_\_Actinobacteria;D\_2\_\_Actinobacteria;D\_3\_\_Micrococcales;D\_4\_\_Bogoriellaceae | 0.0% | 0.0% | 0.0% | 0.0% | 0.0% | 0.0% | 0.0% | 0.0% | 0.0% |
|  | D\_0\_\_Bacteria;D\_1\_\_Actinobacteria;D\_2\_\_Actinobacteria;D\_3\_\_Micrococcales;D\_4\_\_Cellulomonadaceae | 0.6% | 0.0% | 0.0% | 0.1% | 0.1% | 1.6% | 2.9% | 0.0% | 0.0% |
|  | D\_0\_\_Bacteria;D\_1\_\_Actinobacteria;D\_2\_\_Actinobacteria;D\_3\_\_Micrococcales;D\_4\_\_Demequinaceae | 0.0% | 0.0% | 0.0% | 0.0% | 0.0% | 0.0% | 0.0% | 0.0% | 0.0% |
|  | D\_0\_\_Bacteria;D\_1\_\_Actinobacteria;D\_2\_\_Actinobacteria;D\_3\_\_Micrococcales;D\_4\_\_Dermabacteraceae | 0.0% | 0.0% | 0.0% | 0.0% | 0.0% | 0.0% | 0.0% | 0.0% | 0.0% |
|  | D\_0\_\_Bacteria;D\_1\_\_Actinobacteria;D\_2\_\_Actinobacteria;D\_3\_\_Micrococcales;D\_4\_\_Dermacoccaceae | 0.0% | 0.0% | 0.0% | 0.0% | 0.0% | 0.0% | 0.0% | 0.0% | 0.0% |
|  | D\_0\_\_Bacteria;D\_1\_\_Actinobacteria;D\_2\_\_Actinobacteria;D\_3\_\_Micrococcales;D\_4\_\_Dermatophilaceae | 0.0% | 0.0% | 0.0% | 0.0% | 0.0% | 0.0% | 0.0% | 0.0% | 0.0% |
|  | D\_0\_\_Bacteria;D\_1\_\_Actinobacteria;D\_2\_\_Actinobacteria;D\_3\_\_Micrococcales;D\_4\_\_Intrasporangiaceae | 0.1% | 0.0% | 0.0% | 0.0% | 0.0% | 0.0% | 0.0% | 0.1% | 0.1% |
|  | D\_0\_\_Bacteria;D\_1\_\_Actinobacteria;D\_2\_\_Actinobacteria;D\_3\_\_Micrococcales;D\_4\_\_Microbacteriaceae | 0.0% | 0.0% | 0.0% | 0.0% | 0.0% | 0.0% | 0.0% | 0.0% | 0.0% |
|  | D\_0\_\_Bacteria;D\_1\_\_Actinobacteria;D\_2\_\_Actinobacteria;D\_3\_\_Micrococcales;D\_4\_\_Micrococcaceae | 1.0% | 0.5% | 0.5% | 0.7% | 0.7% | 0.1% | 0.1% | 3.4% | 2.4% |
|  | D\_0\_\_Bacteria;D\_1\_\_Actinobacteria;D\_2\_\_Actinobacteria;D\_3\_\_Micrococcales;D\_4\_\_Promicromonosporaceae | 0.0% | 0.0% | 0.0% | 0.0% | 0.0% | 0.0% | 0.0% | 0.1% | 0.0% |
|  | D\_0\_\_Bacteria;D\_1\_\_Actinobacteria;D\_2\_\_Actinobacteria;D\_3\_\_Micrococcales;Other | 0.0% | 0.0% | 0.0% | 0.0% | 0.0% | 0.0% | 0.0% | 0.0% | 0.0% |
|  | D\_0\_\_Bacteria;D\_1\_\_Actinobacteria;D\_2\_\_Actinobacteria;D\_3\_\_Micromonosporales;D\_4\_\_Micromonosporaceae | 3.5% | 5.0% | 5.3% | 5.0% | 5.2% | 3.3% | 2.7% | 0.7% | 0.4% |
|  | D\_0\_\_Bacteria;D\_1\_\_Actinobacteria;D\_2\_\_Actinobacteria;D\_3\_\_PeM15;D\_4\_\_uncultured bacterium | 0.0% | 0.0% | 0.0% | 0.0% | 0.0% | 0.0% | 0.0% | 0.0% | 0.0% |
|  | D\_0\_\_Bacteria;D\_1\_\_Actinobacteria;D\_2\_\_Actinobacteria;D\_3\_\_Propionibacteriales;D\_4\_\_Nocardioidaceae | 3.7% | 3.0% | 3.1% | 1.5% | 1.6% | 6.5% | 4.8% | 5.0% | 3.8% |
|  | D\_0\_\_Bacteria;D\_1\_\_Actinobacteria;D\_2\_\_Actinobacteria;D\_3\_\_Propionibacteriales;D\_4\_\_Propionibacteriaceae | 1.3% | 1.1% | 1.1% | 0.8% | 0.8% | 2.7% | 2.8% | 0.7% | 0.5% |
|  | D\_0\_\_Bacteria;D\_1\_\_Actinobacteria;D\_2\_\_Actinobacteria;D\_3\_\_Propionibacteriales;Other | 0.0% | 0.0% | 0.0% | 0.0% | 0.0% | 0.0% | 0.0% | 0.0% | 0.0% |
|  | D\_0\_\_Bacteria;D\_1\_\_Actinobacteria;D\_2\_\_Actinobacteria;D\_3\_\_Pseudonocardiales;D\_4\_\_Pseudonocardiaceae | 29.5% | 10.9% | 12.2% | 16.4% | 15.5% | 40.1% | 42.9% | 49.2% | 48.6% |
|  | D\_0\_\_Bacteria;D\_1\_\_Actinobacteria;D\_2\_\_Actinobacteria;D\_3\_\_Streptomycetales;D\_4\_\_Streptomycetaceae | 4.3% | 0.1% | 0.1% | 0.1% | 0.1% | 11.4% | 22.7% | 0.1% | 0.0% |
|  | D\_0\_\_Bacteria;D\_1\_\_Actinobacteria;D\_2\_\_Actinobacteria;D\_3\_\_Streptosporangiales;D\_4\_\_Nocardiopsaceae | 0.0% | 0.0% | 0.0% | 0.0% | 0.0% | 0.0% | 0.0% | 0.0% | 0.0% |
|  | D\_0\_\_Bacteria;D\_1\_\_Actinobacteria;D\_2\_\_Actinobacteria;D\_3\_\_Streptosporangiales;D\_4\_\_Streptosporangiaceae | 0.0% | 0.0% | 0.0% | 0.0% | 0.0% | 0.0% | 0.0% | 0.0% | 0.0% |
|  | D\_0\_\_Bacteria;D\_1\_\_Actinobacteria;D\_2\_\_Actinobacteria;D\_3\_\_Streptosporangiales;D\_4\_\_Thermomonosporaceae | 0.0% | 0.0% | 0.0% | 0.0% | 0.0% | 0.0% | 0.0% | 0.0% | 0.0% |
|  | D\_0\_\_Bacteria;D\_1\_\_Actinobacteria;D\_2\_\_Actinobacteria;D\_3\_\_Streptosporangiales;D\_4\_\_uncultured bacterium | 0.0% | 0.0% | 0.0% | 0.0% | 0.0% | 0.0% | 0.0% | 0.0% | 0.0% |
|  | D\_0\_\_Bacteria;D\_1\_\_Actinobacteria;D\_2\_\_Actinobacteria;D\_3\_\_Streptosporangiales;Other | 0.0% | 0.0% | 0.0% | 0.0% | 0.0% | 0.0% | 0.0% | 0.0% | 0.0% |
|  | D\_0\_\_Bacteria;D\_1\_\_Actinobacteria;D\_2\_\_Actinobacteria;Other;Other | 0.0% | 0.0% | 0.0% | 0.0% | 0.0% | 0.1% | 0.1% | 0.0% | 0.0% |
|  | D\_0\_\_Bacteria;D\_1\_\_Actinobacteria;D\_2\_\_Coriobacteriia;D\_3\_\_Coriobacteriales;D\_4\_\_Coriobacteriaceae | 0.0% | 0.0% | 0.0% | 0.0% | 0.0% | 0.0% | 0.0% | 0.0% | 0.0% |
|  | D\_0\_\_Bacteria;D\_1\_\_Actinobacteria;D\_2\_\_MB-A2-108;Ambiguous\_taxa;Ambiguous\_taxa | 0.0% | 0.0% | 0.0% | 0.0% | 0.0% | 0.0% | 0.0% | 0.0% | 0.0% |
|  | D\_0\_\_Bacteria;D\_1\_\_Actinobacteria;D\_2\_\_MB-A2-108;D\_3\_\_uncultured bacterium;D\_4\_\_uncultured bacterium | 0.0% | 0.0% | 0.0% | 0.0% | 0.0% | 0.0% | 0.0% | 0.0% | 0.0% |
|  | D\_0\_\_Bacteria;D\_1\_\_Actinobacteria;D\_2\_\_MB-A2-108;Other;Other | 0.0% | 0.0% | 0.0% | 0.0% | 0.0% | 0.0% | 0.0% | 0.0% | 0.0% |
|  | D\_0\_\_Bacteria;D\_1\_\_Actinobacteria;D\_2\_\_Nitriliruptoria;D\_3\_\_Euzebyales;D\_4\_\_Euzebyaceae | 15.3% | 27.5% | 28.0% | 29.1% | 28.6% | 2.3% | 2.6% | 2.4% | 2.1% |
|  | D\_0\_\_Bacteria;D\_1\_\_Actinobacteria;D\_2\_\_Nitriliruptoria;D\_3\_\_Nitriliruptorales;D\_4\_\_Nitriliruptoraceae | 0.0% | 0.1% | 0.0% | 0.0% | 0.0% | 0.0% | 0.0% | 0.0% | 0.0% |
|  | D\_0\_\_Bacteria;D\_1\_\_Actinobacteria;D\_2\_\_Nitriliruptoria;Other;Other | 0.0% | 0.0% | 0.0% | 0.0% | 0.0% | 0.0% | 0.0% | 0.0% | 0.0% |
|  | D\_0\_\_Bacteria;D\_1\_\_Actinobacteria;D\_2\_\_OPB41;D\_3\_\_uncultured bacterium;D\_4\_\_uncultured bacterium | 0.0% | 0.0% | 0.0% | 0.0% | 0.0% | 0.0% | 0.0% | 0.0% | 0.0% |
|  | D\_0\_\_Bacteria;D\_1\_\_Actinobacteria;D\_2\_\_Rubrobacteria;D\_3\_\_Rubrobacterales;D\_4\_\_Rubrobacteriaceae | 0.0% | 0.0% | 0.0% | 0.0% | 0.0% | 0.0% | 0.0% | 0.0% | 0.1% |
|  | D\_0\_\_Bacteria;D\_1\_\_Actinobacteria;D\_2\_\_TakashiAC-B11;D\_3\_\_uncultured Actinomycetales bacterium;D\_4\_\_uncultured Actinomycetales bacterium | 0.0% | 0.0% | 0.0% | 0.0% | 0.0% | 0.0% | 0.0% | 0.0% | 0.0% |
|  | D\_0\_\_Bacteria;D\_1\_\_Actinobacteria;D\_2\_\_TakashiAC-B11;D\_3\_\_uncultured actinobacterium;D\_4\_\_uncultured actinobacterium | 0.0% | 0.0% | 0.0% | 0.0% | 0.0% | 0.0% | 0.0% | 0.0% | 0.0% |
|  | D\_0\_\_Bacteria;D\_1\_\_Actinobacteria;D\_2\_\_TakashiAC-B11;D\_3\_\_uncultured bacterium;D\_4\_\_uncultured bacterium | 0.6% | 0.8% | 0.7% | 1.0% | 1.0% | 0.4% | 0.1% | 0.3% | 0.3% |
|  | D\_0\_\_Bacteria;D\_1\_\_Actinobacteria;D\_2\_\_TakashiAC-B11;Other;Other | 1.7% | 3.1% | 2.9% | 2.4% | 2.2% | 1.0% | 0.1% | 1.0% | 0.8% |
|  | D\_0\_\_Bacteria;D\_1\_\_Actinobacteria;D\_2\_\_Thermoleophilia;D\_3\_\_Gaiellales;D\_4\_\_Gaiellaceae | 0.1% | 0.1% | 0.1% | 0.1% | 0.1% | 0.1% | 0.0% | 0.0% | 0.1% |
|  | D\_0\_\_Bacteria;D\_1\_\_Actinobacteria;D\_2\_\_Thermoleophilia;D\_3\_\_Gaiellales;D\_4\_\_uncultured | 0.3% | 0.4% | 0.3% | 0.3% | 0.4% | 0.3% | 0.1% | 0.1% | 0.2% |
|  | D\_0\_\_Bacteria;D\_1\_\_Actinobacteria;D\_2\_\_Thermoleophilia;D\_3\_\_Solirubrobacterales;Ambiguous\_taxa | 0.0% | 0.0% | 0.0% | 0.0% | 0.0% | 0.0% | 0.0% | 0.0% | 0.0% |
|  | D\_0\_\_Bacteria;D\_1\_\_Actinobacteria;D\_2\_\_Thermoleophilia;D\_3\_\_Solirubrobacterales;D\_4\_\_0319-6M6 | 0.0% | 0.0% | 0.0% | 0.0% | 0.0% | 0.0% | 0.0% | 0.0% | 0.0% |
|  | D\_0\_\_Bacteria;D\_1\_\_Actinobacteria;D\_2\_\_Thermoleophilia;D\_3\_\_Solirubrobacterales;D\_4\_\_288-2 | 0.0% | 0.0% | 0.0% | 0.0% | 0.0% | 0.1% | 0.0% | 0.0% | 0.1% |
|  | D\_0\_\_Bacteria;D\_1\_\_Actinobacteria;D\_2\_\_Thermoleophilia;D\_3\_\_Solirubrobacterales;D\_4\_\_Elev-16S-1332 | 0.6% | 0.7% | 0.6% | 0.3% | 0.4% | 1.3% | 0.9% | 0.3% | 0.2% |
|  | D\_0\_\_Bacteria;D\_1\_\_Actinobacteria;D\_2\_\_Thermoleophilia;D\_3\_\_Solirubrobacterales;D\_4\_\_FFCH11085 | 0.0% | 0.0% | 0.0% | 0.0% | 0.0% | 0.0% | 0.0% | 0.0% | 0.0% |
|  | D\_0\_\_Bacteria;D\_1\_\_Actinobacteria;D\_2\_\_Thermoleophilia;D\_3\_\_Solirubrobacterales;D\_4\_\_Gsoil-1167 | 0.0% | 0.0% | 0.0% | 0.1% | 0.0% | 0.1% | 0.0% | 0.0% | 0.0% |
|  | D\_0\_\_Bacteria;D\_1\_\_Actinobacteria;D\_2\_\_Thermoleophilia;D\_3\_\_Solirubrobacterales;D\_4\_\_Parviterribacteraceae | 0.0% | 0.0% | 0.0% | 0.0% | 0.0% | 0.0% | 0.0% | 0.0% | 0.0% |
|  | D\_0\_\_Bacteria;D\_1\_\_Actinobacteria;D\_2\_\_Thermoleophilia;D\_3\_\_Solirubrobacterales;D\_4\_\_Patulibacteraceae | 0.0% | 0.0% | 0.0% | 0.0% | 0.0% | 0.0% | 0.0% | 0.0% | 0.0% |
|  | D\_0\_\_Bacteria;D\_1\_\_Actinobacteria;D\_2\_\_Thermoleophilia;D\_3\_\_Solirubrobacterales;D\_4\_\_Q3-6C1 | 0.0% | 0.0% | 0.0% | 0.0% | 0.0% | 0.0% | 0.0% | 0.0% | 0.0% |
|  | D\_0\_\_Bacteria;D\_1\_\_Actinobacteria;D\_2\_\_Thermoleophilia;D\_3\_\_Solirubrobacterales;D\_4\_\_S1-80 | 0.0% | 0.0% | 0.0% | 0.0% | 0.0% | 0.0% | 0.0% | 0.0% | 0.0% |
|  | D\_0\_\_Bacteria;D\_1\_\_Actinobacteria;D\_2\_\_Thermoleophilia;D\_3\_\_Solirubrobacterales;D\_4\_\_Solirubrobacteraceae | 0.1% | 0.2% | 0.2% | 0.1% | 0.2% | 0.2% | 0.1% | 0.1% | 0.1% |
|  | D\_0\_\_Bacteria;D\_1\_\_Actinobacteria;D\_2\_\_Thermoleophilia;D\_3\_\_Solirubrobacterales;D\_4\_\_TM146 | 0.0% | 0.0% | 0.0% | 0.0% | 0.0% | 0.0% | 0.0% | 0.0% | 0.0% |
|  | D\_0\_\_Bacteria;D\_1\_\_Actinobacteria;D\_2\_\_Thermoleophilia;D\_3\_\_Solirubrobacterales;D\_4\_\_YNPFFP1 | 0.0% | 0.0% | 0.0% | 0.0% | 0.0% | 0.0% | 0.0% | 0.0% | 0.0% |
|  | D\_0\_\_Bacteria;D\_1\_\_Actinobacteria;D\_2\_\_Thermoleophilia;D\_3\_\_Solirubrobacterales;D\_4\_\_uncultured | 0.0% | 0.0% | 0.0% | 0.0% | 0.0% | 0.0% | 0.0% | 0.0% | 0.0% |
|  | D\_0\_\_Bacteria;D\_1\_\_Actinobacteria;D\_2\_\_Thermoleophilia;D\_3\_\_Solirubrobacterales;Other | 0.0% | 0.0% | 0.0% | 0.0% | 0.0% | 0.0% | 0.0% | 0.0% | 0.1% |
|  | D\_0\_\_Bacteria;D\_1\_\_Actinobacteria;D\_2\_\_Thermoleophilia;Other;Other | 0.0% | 0.0% | 0.0% | 0.0% | 0.0% | 0.0% | 0.0% | 0.0% | 0.0% |
|  | D\_0\_\_Bacteria;D\_1\_\_Actinobacteria;Other;Other;Other | 0.0% | 0.0% | 0.0% | 0.0% | 0.0% | 0.0% | 0.0% | 0.0% | 0.0% |
|  | D\_0\_\_Bacteria;D\_1\_\_Armatimonadetes;D\_2\_\_Armatimonadia;D\_3\_\_Armatimonadales;Ambiguous\_taxa | 0.0% | 0.0% | 0.0% | 0.0% | 0.0% | 0.0% | 0.0% | 0.0% | 0.0% |
|  | D\_0\_\_Bacteria;D\_1\_\_Armatimonadetes;D\_2\_\_Armatimonadia;D\_3\_\_Armatimonadales;D\_4\_\_uncultured Armatimonadetes bacterium | 0.0% | 0.0% | 0.0% | 0.0% | 0.0% | 0.0% | 0.0% | 0.0% | 0.0% |
|  | D\_0\_\_Bacteria;D\_1\_\_Armatimonadetes;D\_2\_\_Armatimonadia;D\_3\_\_Armatimonadales;D\_4\_\_uncultured bacterium | 0.0% | 0.0% | 0.0% | 0.0% | 0.0% | 0.0% | 0.0% | 0.0% | 0.0% |
|  | D\_0\_\_Bacteria;D\_1\_\_Armatimonadetes;D\_2\_\_Fimbriimonadia;D\_3\_\_Fimbriimonadales;D\_4\_\_Fimbriimonadaceae | 0.0% | 0.0% | 0.0% | 0.0% | 0.0% | 0.0% | 0.0% | 0.0% | 0.0% |
|  | D\_0\_\_Bacteria;D\_1\_\_Armatimonadetes;D\_2\_\_uncultured;D\_3\_\_uncultured bacterium;D\_4\_\_uncultured bacterium | 0.0% | 0.0% | 0.0% | 0.0% | 0.0% | 0.0% | 0.0% | 0.0% | 0.0% |
|  | D\_0\_\_Bacteria;D\_1\_\_BRC1;D\_2\_\_uncultured bacterium;D\_3\_\_uncultured bacterium;D\_4\_\_uncultured bacterium | 0.0% | 0.0% | 0.0% | 0.0% | 0.0% | 0.0% | 0.0% | 0.0% | 0.0% |
|  | D\_0\_\_Bacteria;D\_1\_\_BRC1;Other;Other;Other | 0.0% | 0.0% | 0.0% | 0.0% | 0.0% | 0.0% | 0.0% | 0.0% | 0.0% |
|  | D\_0\_\_Bacteria;D\_1\_\_Bacteroidetes;D\_2\_\_Bacteroidetes BD2-2;D\_3\_\_uncultured bacterium;D\_4\_\_uncultured bacterium | 0.0% | 0.0% | 0.0% | 0.0% | 0.0% | 0.0% | 0.0% | 0.0% | 0.0% |
|  | D\_0\_\_Bacteria;D\_1\_\_Bacteroidetes;D\_2\_\_Bacteroidetes Incertae Sedis;D\_3\_\_Order II;D\_4\_\_Rhodothermaceae | 0.0% | 0.0% | 0.0% | 0.0% | 0.0% | 0.0% | 0.0% | 0.0% | 0.0% |
|  | D\_0\_\_Bacteria;D\_1\_\_Bacteroidetes;D\_2\_\_Bacteroidia;D\_3\_\_Bacteroidales;D\_4\_\_Bacteroidaceae | 0.0% | 0.0% | 0.0% | 0.0% | 0.0% | 0.0% | 0.0% | 0.0% | 0.0% |
|  | D\_0\_\_Bacteria;D\_1\_\_Bacteroidetes;D\_2\_\_Bacteroidia;D\_3\_\_Bacteroidales;D\_4\_\_Porphyromonadaceae | 0.0% | 0.0% | 0.0% | 0.0% | 0.0% | 0.0% | 0.0% | 0.0% | 0.2% |
|  | D\_0\_\_Bacteria;D\_1\_\_Bacteroidetes;D\_2\_\_Bacteroidia;D\_3\_\_Bacteroidales;D\_4\_\_Prevotellaceae | 0.0% | 0.0% | 0.0% | 0.0% | 0.0% | 0.0% | 0.0% | 0.0% | 0.2% |
|  | D\_0\_\_Bacteria;D\_1\_\_Bacteroidetes;D\_2\_\_Cytophagia;D\_3\_\_Cytophagales;D\_4\_\_Cyclobacteriaceae | 0.0% | 0.0% | 0.0% | 0.0% | 0.0% | 0.0% | 0.0% | 0.0% | 0.0% |
|  | D\_0\_\_Bacteria;D\_1\_\_Bacteroidetes;D\_2\_\_Cytophagia;D\_3\_\_Cytophagales;D\_4\_\_Cytophagaceae | 0.1% | 0.0% | 0.0% | 0.1% | 0.1% | 0.1% | 0.0% | 0.1% | 0.1% |
|  | D\_0\_\_Bacteria;D\_1\_\_Bacteroidetes;D\_2\_\_Cytophagia;D\_3\_\_Cytophagales;D\_4\_\_Flammeovirgaceae | 0.0% | 0.0% | 0.0% | 0.0% | 0.0% | 0.0% | 0.0% | 0.0% | 0.1% |
|  | D\_0\_\_Bacteria;D\_1\_\_Bacteroidetes;D\_2\_\_Cytophagia;D\_3\_\_Cytophagales;D\_4\_\_MWH-CFBk5 | 0.0% | 0.0% | 0.0% | 0.0% | 0.0% | 0.0% | 0.0% | 0.0% | 0.0% |
|  | D\_0\_\_Bacteria;D\_1\_\_Bacteroidetes;D\_2\_\_Flavobacteriia;D\_3\_\_Flavobacteriales;D\_4\_\_Flavobacteriaceae | 0.0% | 0.0% | 0.0% | 0.0% | 0.0% | 0.0% | 0.0% | 0.0% | 0.2% |
|  | D\_0\_\_Bacteria;D\_1\_\_Bacteroidetes;D\_2\_\_Flavobacteriia;D\_3\_\_Flavobacteriales;D\_4\_\_NS9 marine group | 0.0% | 0.0% | 0.0% | 0.0% | 0.0% | 0.0% | 0.0% | 0.0% | 0.0% |
|  | D\_0\_\_Bacteria;D\_1\_\_Bacteroidetes;D\_2\_\_Sphingobacteriia;D\_3\_\_Sphingobacteriales;D\_4\_\_Chitinophagaceae | 0.0% | 0.0% | 0.0% | 0.0% | 0.0% | 0.0% | 0.0% | 0.0% | 0.0% |
|  | D\_0\_\_Bacteria;D\_1\_\_Bacteroidetes;D\_2\_\_Sphingobacteriia;D\_3\_\_Sphingobacteriales;D\_4\_\_KD3-93 | 0.0% | 0.1% | 0.1% | 0.1% | 0.1% | 0.0% | 0.0% | 0.0% | 0.0% |
|  | D\_0\_\_Bacteria;D\_1\_\_Bacteroidetes;D\_2\_\_Sphingobacteriia;D\_3\_\_Sphingobacteriales;D\_4\_\_Lentimicrobiaceae | 0.0% | 0.0% | 0.0% | 0.0% | 0.0% | 0.0% | 0.0% | 0.0% | 0.0% |
|  | D\_0\_\_Bacteria;D\_1\_\_Bacteroidetes;D\_2\_\_Sphingobacteriia;D\_3\_\_Sphingobacteriales;D\_4\_\_LiUU-11-161 | 0.0% | 0.0% | 0.0% | 0.0% | 0.0% | 0.0% | 0.0% | 0.0% | 0.0% |
|  | D\_0\_\_Bacteria;D\_1\_\_Bacteroidetes;D\_2\_\_Sphingobacteriia;D\_3\_\_Sphingobacteriales;D\_4\_\_NS11-12 marine group | 0.0% | 0.0% | 0.0% | 0.0% | 0.0% | 0.0% | 0.0% | 0.0% | 0.0% |
|  | D\_0\_\_Bacteria;D\_1\_\_Bacteroidetes;D\_2\_\_Sphingobacteriia;D\_3\_\_Sphingobacteriales;D\_4\_\_Saprospiraceae | 0.0% | 0.0% | 0.0% | 0.0% | 0.0% | 0.0% | 0.0% | 0.0% | 0.0% |
|  | D\_0\_\_Bacteria;D\_1\_\_Bacteroidetes;D\_2\_\_Sphingobacteriia;D\_3\_\_Sphingobacteriales;D\_4\_\_Sphingobacteriaceae | 0.0% | 0.0% | 0.0% | 0.0% | 0.0% | 0.0% | 0.0% | 0.0% | 0.0% |
|  | D\_0\_\_Bacteria;D\_1\_\_Bacteroidetes;D\_2\_\_Sphingobacteriia;D\_3\_\_Sphingobacteriales;D\_4\_\_env.OPS 17 | 0.0% | 0.0% | 0.0% | 0.0% | 0.0% | 0.0% | 0.0% | 0.0% | 0.0% |
|  | D\_0\_\_Bacteria;D\_1\_\_Bacteroidetes;D\_2\_\_Sphingobacteriia;D\_3\_\_Sphingobacteriales;Other | 0.0% | 0.0% | 0.0% | 0.0% | 0.0% | 0.0% | 0.0% | 0.0% | 0.0% |
|  | D\_0\_\_Bacteria;D\_1\_\_Chlamydiae;D\_2\_\_Chlamydiae;D\_3\_\_Chlamydiales;D\_4\_\_Parachlamydiaceae | 0.0% | 0.0% | 0.0% | 0.0% | 0.0% | 0.0% | 0.0% | 0.0% | 0.0% |
|  | D\_0\_\_Bacteria;D\_1\_\_Chlamydiae;D\_2\_\_Chlamydiae;D\_3\_\_Chlamydiales;D\_4\_\_Simkaniaceae | 0.0% | 0.0% | 0.0% | 0.0% | 0.0% | 0.0% | 0.0% | 0.0% | 0.0% |
|  | D\_0\_\_Bacteria;D\_1\_\_Chlamydiae;D\_2\_\_Chlamydiae;D\_3\_\_Chlamydiales;D\_4\_\_cvE6 | 0.0% | 0.0% | 0.0% | 0.0% | 0.0% | 0.0% | 0.0% | 0.0% | 0.0% |
|  | D\_0\_\_Bacteria;D\_1\_\_Chlorobi;D\_2\_\_Chlorobia;D\_3\_\_Chlorobiales;D\_4\_\_OPB56 | 0.0% | 0.0% | 0.0% | 0.0% | 0.0% | 0.0% | 0.0% | 0.0% | 0.0% |
|  | D\_0\_\_Bacteria;D\_1\_\_Chlorobi;D\_2\_\_Chlorobia;D\_3\_\_Chlorobiales;D\_4\_\_SJA-28 | 0.0% | 0.0% | 0.0% | 0.0% | 0.0% | 0.0% | 0.0% | 0.0% | 0.0% |
|  | D\_0\_\_Bacteria;D\_1\_\_Chloroflexi;D\_2\_\_Anaerolineae;D\_3\_\_Anaerolineales;D\_4\_\_Anaerolineaceae | 0.0% | 0.0% | 0.0% | 0.0% | 0.0% | 0.0% | 0.0% | 0.0% | 0.0% |
|  | D\_0\_\_Bacteria;D\_1\_\_Chloroflexi;D\_2\_\_Ardenticatenia;D\_3\_\_Ardenticatenales;D\_4\_\_uncultured bacterium | 0.0% | 0.0% | 0.0% | 0.0% | 0.0% | 0.0% | 0.0% | 0.0% | 0.0% |
|  | D\_0\_\_Bacteria;D\_1\_\_Chloroflexi;D\_2\_\_Caldilineae;D\_3\_\_Caldilineales;D\_4\_\_Caldilineaceae | 0.0% | 0.0% | 0.0% | 0.0% | 0.0% | 0.0% | 0.0% | 0.0% | 0.0% |
|  | D\_0\_\_Bacteria;D\_1\_\_Chloroflexi;D\_2\_\_Chloroflexi Incertae Sedis;D\_3\_\_Unknown Order;D\_4\_\_Unknown Family | 0.0% | 0.0% | 0.0% | 0.0% | 0.0% | 0.0% | 0.0% | 0.0% | 0.0% |
|  | D\_0\_\_Bacteria;D\_1\_\_Chloroflexi;D\_2\_\_Chloroflexia;D\_3\_\_Chloroflexales;D\_4\_\_Roseiflexaceae | 0.0% | 0.0% | 0.0% | 0.0% | 0.0% | 0.0% | 0.0% | 0.0% | 0.0% |
|  | D\_0\_\_Bacteria;D\_1\_\_Chloroflexi;D\_2\_\_Chloroflexia;D\_3\_\_Herpetosiphonales;D\_4\_\_Herpetosiphonaceae | 0.0% | 0.0% | 0.0% | 0.0% | 0.0% | 0.0% | 0.0% | 0.0% | 0.0% |
|  | D\_0\_\_Bacteria;D\_1\_\_Chloroflexi;D\_2\_\_Chloroflexia;D\_3\_\_Kallotenuales;D\_4\_\_AKIW781 | 0.0% | 0.0% | 0.0% | 0.0% | 0.0% | 0.0% | 0.0% | 0.0% | 0.0% |
|  | D\_0\_\_Bacteria;D\_1\_\_Chloroflexi;D\_2\_\_Gitt-GS-136;Ambiguous\_taxa;Ambiguous\_taxa | 0.0% | 0.0% | 0.0% | 0.0% | 0.0% | 0.0% | 0.0% | 0.0% | 0.0% |
|  | D\_0\_\_Bacteria;D\_1\_\_Chloroflexi;D\_2\_\_Gitt-GS-136;D\_3\_\_uncultured bacterium;D\_4\_\_uncultured bacterium | 0.0% | 0.0% | 0.0% | 0.0% | 0.0% | 0.0% | 0.0% | 0.0% | 0.0% |
|  | D\_0\_\_Bacteria;D\_1\_\_Chloroflexi;D\_2\_\_Gitt-GS-136;D\_3\_\_uncultured bacterium #0319-6C24;D\_4\_\_uncultured bacterium #0319-6C24 | 0.0% | 0.0% | 0.0% | 0.0% | 0.0% | 0.0% | 0.0% | 0.0% | 0.0% |
|  | D\_0\_\_Bacteria;D\_1\_\_Chloroflexi;D\_2\_\_Gitt-GS-136;Other;Other | 0.7% | 1.1% | 1.1% | 1.1% | 1.1% | 0.8% | 0.1% | 0.2% | 0.1% |
|  | D\_0\_\_Bacteria;D\_1\_\_Chloroflexi;D\_2\_\_JG30-KF-CM66;Ambiguous\_taxa;Ambiguous\_taxa | 0.0% | 0.0% | 0.0% | 0.0% | 0.0% | 0.0% | 0.0% | 0.0% | 0.0% |
|  | D\_0\_\_Bacteria;D\_1\_\_Chloroflexi;D\_2\_\_JG30-KF-CM66;D\_3\_\_uncultured Chloroflexi bacterium;D\_4\_\_uncultured Chloroflexi bacterium | 0.0% | 0.0% | 0.0% | 0.0% | 0.0% | 0.0% | 0.0% | 0.0% | 0.0% |
|  | D\_0\_\_Bacteria;D\_1\_\_Chloroflexi;D\_2\_\_JG30-KF-CM66;D\_3\_\_uncultured bacterium;D\_4\_\_uncultured bacterium | 0.6% | 0.8% | 0.7% | 0.7% | 0.7% | 0.8% | 0.3% | 0.4% | 0.5% |
|  | D\_0\_\_Bacteria;D\_1\_\_Chloroflexi;D\_2\_\_JG30-KF-CM66;Other;Other | 0.1% | 0.2% | 0.2% | 0.2% | 0.2% | 0.1% | 0.0% | 0.1% | 0.0% |
|  | D\_0\_\_Bacteria;D\_1\_\_Chloroflexi;D\_2\_\_JG37-AG-4;D\_3\_\_uncultured bacterium;D\_4\_\_uncultured bacterium | 0.0% | 0.0% | 0.0% | 0.0% | 0.0% | 0.0% | 0.0% | 0.0% | 0.0% |
|  | D\_0\_\_Bacteria;D\_1\_\_Chloroflexi;D\_2\_\_KD4-96;Ambiguous\_taxa;Ambiguous\_taxa | 0.0% | 0.0% | 0.0% | 0.0% | 0.0% | 0.0% | 0.0% | 0.0% | 0.0% |
|  | D\_0\_\_Bacteria;D\_1\_\_Chloroflexi;D\_2\_\_KD4-96;D\_3\_\_uncultured bacterium;D\_4\_\_uncultured bacterium | 0.1% | 0.1% | 0.1% | 0.1% | 0.1% | 0.0% | 0.0% | 0.0% | 0.2% |
|  | D\_0\_\_Bacteria;D\_1\_\_Chloroflexi;D\_2\_\_KD4-96;Other;Other | 0.0% | 0.0% | 0.0% | 0.0% | 0.0% | 0.0% | 0.0% | 0.0% | 0.0% |
|  | D\_0\_\_Bacteria;D\_1\_\_Chloroflexi;D\_2\_\_Ktedonobacteria;D\_3\_\_C0119;D\_4\_\_uncultured bacterium | 0.0% | 0.0% | 0.0% | 0.0% | 0.0% | 0.0% | 0.0% | 0.0% | 0.0% |
|  | D\_0\_\_Bacteria;D\_1\_\_Chloroflexi;D\_2\_\_NLS2-31;D\_3\_\_uncultured bacterium;D\_4\_\_uncultured bacterium | 0.0% | 0.0% | 0.0% | 0.0% | 0.0% | 0.0% | 0.0% | 0.0% | 0.0% |
|  | D\_0\_\_Bacteria;D\_1\_\_Chloroflexi;D\_2\_\_P2-11E;D\_3\_\_uncultured bacterium;D\_4\_\_uncultured bacterium | 0.0% | 0.0% | 0.0% | 0.0% | 0.0% | 0.0% | 0.0% | 0.0% | 0.0% |
|  | D\_0\_\_Bacteria;D\_1\_\_Chloroflexi;D\_2\_\_S085;Ambiguous\_taxa;Ambiguous\_taxa | 0.0% | 0.0% | 0.0% | 0.0% | 0.0% | 0.0% | 0.0% | 0.0% | 0.0% |
|  | D\_0\_\_Bacteria;D\_1\_\_Chloroflexi;D\_2\_\_S085;D\_3\_\_uncultured Chloroflexi bacterium;D\_4\_\_uncultured Chloroflexi bacterium | 0.0% | 0.0% | 0.0% | 0.0% | 0.0% | 0.0% | 0.0% | 0.0% | 0.0% |
|  | D\_0\_\_Bacteria;D\_1\_\_Chloroflexi;D\_2\_\_S085;D\_3\_\_uncultured bacterium;D\_4\_\_uncultured bacterium | 0.5% | 0.9% | 0.8% | 0.6% | 0.6% | 0.4% | 0.1% | 0.2% | 0.5% |
|  | D\_0\_\_Bacteria;D\_1\_\_Chloroflexi;D\_2\_\_S085;Other;Other | 0.0% | 0.0% | 0.0% | 0.0% | 0.0% | 0.0% | 0.0% | 0.0% | 0.0% |
|  | D\_0\_\_Bacteria;D\_1\_\_Chloroflexi;D\_2\_\_SAR202 clade;Ambiguous\_taxa;Ambiguous\_taxa | 0.0% | 0.0% | 0.0% | 0.0% | 0.0% | 0.0% | 0.0% | 0.0% | 0.0% |
|  | D\_0\_\_Bacteria;D\_1\_\_Chloroflexi;D\_2\_\_SAR202 clade;D\_3\_\_uncultured Chloroflexi bacterium;D\_4\_\_uncultured Chloroflexi bacterium | 0.0% | 0.0% | 0.0% | 0.0% | 0.0% | 0.0% | 0.0% | 0.0% | 0.0% |
|  | D\_0\_\_Bacteria;D\_1\_\_Chloroflexi;D\_2\_\_SAR202 clade;D\_3\_\_uncultured bacterium;D\_4\_\_uncultured bacterium | 0.1% | 0.1% | 0.0% | 0.1% | 0.1% | 0.0% | 0.0% | 0.1% | 0.0% |
|  | D\_0\_\_Bacteria;D\_1\_\_Chloroflexi;D\_2\_\_SAR202 clade;Other;Other | 0.0% | 0.0% | 0.0% | 0.0% | 0.0% | 0.0% | 0.0% | 0.0% | 0.0% |
|  | D\_0\_\_Bacteria;D\_1\_\_Chloroflexi;D\_2\_\_TK10;D\_3\_\_uncultured Chloroflexi bacterium;D\_4\_\_uncultured Chloroflexi bacterium | 0.0% | 0.0% | 0.0% | 0.0% | 0.0% | 0.0% | 0.0% | 0.0% | 0.0% |
|  | D\_0\_\_Bacteria;D\_1\_\_Chloroflexi;D\_2\_\_TK10;D\_3\_\_uncultured bacterium;D\_4\_\_uncultured bacterium | 0.2% | 0.4% | 0.3% | 0.3% | 0.3% | 0.3% | 0.1% | 0.2% | 0.1% |
|  | D\_0\_\_Bacteria;D\_1\_\_Chloroflexi;D\_2\_\_TK10;D\_3\_\_uncultured soil bacterium;D\_4\_\_uncultured soil bacterium | 0.0% | 0.0% | 0.0% | 0.0% | 0.0% | 0.0% | 0.0% | 0.0% | 0.0% |
|  | D\_0\_\_Bacteria;D\_1\_\_Chloroflexi;D\_2\_\_TK10;Other;Other | 0.0% | 0.0% | 0.0% | 0.0% | 0.0% | 0.0% | 0.0% | 0.0% | 0.0% |
|  | D\_0\_\_Bacteria;D\_1\_\_Chloroflexi;D\_2\_\_Thermomicrobia;D\_3\_\_AKYG1722;D\_4\_\_uncultured Chloroflexi bacterium | 0.0% | 0.0% | 0.0% | 0.0% | 0.0% | 0.0% | 0.0% | 0.0% | 0.0% |
|  | D\_0\_\_Bacteria;D\_1\_\_Chloroflexi;D\_2\_\_Thermomicrobia;D\_3\_\_AKYG1722;D\_4\_\_uncultured Sphaerobacter sp. | 0.0% | 0.0% | 0.0% | 0.0% | 0.0% | 0.0% | 0.0% | 0.0% | 0.0% |
|  | D\_0\_\_Bacteria;D\_1\_\_Chloroflexi;D\_2\_\_Thermomicrobia;D\_3\_\_AKYG1722;D\_4\_\_uncultured bacterium | 0.1% | 0.3% | 0.2% | 0.2% | 0.2% | 0.1% | 0.0% | 0.1% | 0.0% |
|  | D\_0\_\_Bacteria;D\_1\_\_Chloroflexi;D\_2\_\_Thermomicrobia;D\_3\_\_AKYG1722;Other | 0.1% | 0.1% | 0.1% | 0.0% | 0.0% | 0.0% | 0.1% | 0.1% | 0.2% |
|  | D\_0\_\_Bacteria;D\_1\_\_Chloroflexi;D\_2\_\_Thermomicrobia;D\_3\_\_JG30-KF-CM45;Ambiguous\_taxa | 0.0% | 0.0% | 0.0% | 0.0% | 0.0% | 0.0% | 0.0% | 0.0% | 0.0% |
|  | D\_0\_\_Bacteria;D\_1\_\_Chloroflexi;D\_2\_\_Thermomicrobia;D\_3\_\_JG30-KF-CM45;D\_4\_\_uncultured Chloroflexi bacterium | 0.0% | 0.0% | 0.0% | 0.0% | 0.0% | 0.0% | 0.0% | 0.0% | 0.0% |
|  | D\_0\_\_Bacteria;D\_1\_\_Chloroflexi;D\_2\_\_Thermomicrobia;D\_3\_\_JG30-KF-CM45;D\_4\_\_uncultured bacterium | 0.1% | 0.2% | 0.2% | 0.0% | 0.0% | 0.1% | 0.0% | 0.3% | 0.2% |
|  | D\_0\_\_Bacteria;D\_1\_\_Chloroflexi;D\_2\_\_Thermomicrobia;D\_3\_\_Sphaerobacterales;D\_4\_\_Sphaerobacteraceae | 0.0% | 0.0% | 0.0% | 0.0% | 0.0% | 0.0% | 0.0% | 0.0% | 0.0% |
|  | D\_0\_\_Bacteria;D\_1\_\_Chloroflexi;Other;Other;Other | 0.0% | 0.0% | 0.0% | 0.0% | 0.0% | 0.0% | 0.0% | 0.0% | 0.0% |
|  | D\_0\_\_Bacteria;D\_1\_\_Cyanobacteria;D\_2\_\_Chloroplast;Ambiguous\_taxa;Ambiguous\_taxa | 0.0% | 0.0% | 0.0% | 0.0% | 0.0% | 0.0% | 0.0% | 0.0% | 0.0% |
|  | D\_0\_\_Bacteria;D\_1\_\_Cyanobacteria;D\_2\_\_Chloroplast;D\_3\_\_uncultured bacterium;D\_4\_\_uncultured bacterium | 0.0% | 0.0% | 0.0% | 0.0% | 0.0% | 0.0% | 0.0% | 0.0% | 0.0% |
|  | D\_0\_\_Bacteria;D\_1\_\_Cyanobacteria;D\_2\_\_Cyanobacteria;D\_3\_\_SubsectionIII;D\_4\_\_FamilyI | 0.0% | 0.0% | 0.0% | 0.0% | 0.0% | 0.0% | 0.0% | 0.0% | 0.0% |
|  | D\_0\_\_Bacteria;D\_1\_\_Cyanobacteria;D\_2\_\_Cyanobacteria;D\_3\_\_uncultured;D\_4\_\_uncultured bacterium | 0.0% | 0.0% | 0.0% | 0.0% | 0.0% | 0.0% | 0.0% | 0.0% | 0.0% |
|  | D\_0\_\_Bacteria;D\_1\_\_Cyanobacteria;D\_2\_\_Melainabacteria;D\_3\_\_Gastranaerophilales;Other | 0.0% | 0.0% | 0.0% | 0.0% | 0.0% | 0.0% | 0.0% | 0.0% | 0.0% |
|  | D\_0\_\_Bacteria;D\_1\_\_Cyanobacteria;D\_2\_\_Melainabacteria;D\_3\_\_Vampirovibrionales;D\_4\_\_uncultured bacterium | 0.0% | 0.0% | 0.0% | 0.0% | 0.0% | 0.0% | 0.0% | 0.0% | 0.0% |
|  | D\_0\_\_Bacteria;D\_1\_\_Deinococcus-Thermus;D\_2\_\_Deinococci;D\_3\_\_Deinococcales;D\_4\_\_Deinococcaceae | 0.0% | 0.0% | 0.0% | 0.0% | 0.0% | 0.0% | 0.0% | 0.0% | 0.0% |
|  | D\_0\_\_Bacteria;D\_1\_\_Deinococcus-Thermus;D\_2\_\_Deinococci;D\_3\_\_Deinococcales;D\_4\_\_Trueperaceae | 0.0% | 0.0% | 0.0% | 0.0% | 0.0% | 0.0% | 0.0% | 0.0% | 0.1% |
|  | D\_0\_\_Bacteria;D\_1\_\_Deinococcus-Thermus;D\_2\_\_Deinococci;D\_3\_\_Thermales;D\_4\_\_Thermaceae | 0.0% | 0.0% | 0.0% | 0.0% | 0.0% | 0.0% | 0.0% | 0.0% | 0.0% |
|  | D\_0\_\_Bacteria;D\_1\_\_Elusimicrobia;D\_2\_\_Elusimicrobia;D\_3\_\_Lineage IIa;D\_4\_\_uncultured bacterium | 0.0% | 0.0% | 0.0% | 0.0% | 0.0% | 0.0% | 0.0% | 0.0% | 0.0% |
|  | D\_0\_\_Bacteria;D\_1\_\_Elusimicrobia;D\_2\_\_Elusimicrobia;D\_3\_\_Lineage IIb;D\_4\_\_uncultured bacterium | 0.0% | 0.0% | 0.0% | 0.0% | 0.0% | 0.0% | 0.0% | 0.0% | 0.0% |
|  | D\_0\_\_Bacteria;D\_1\_\_Elusimicrobia;D\_2\_\_Elusimicrobia;D\_3\_\_Lineage IV;D\_4\_\_uncultured bacterium | 0.0% | 0.0% | 0.0% | 0.0% | 0.0% | 0.0% | 0.0% | 0.0% | 0.0% |
|  | D\_0\_\_Bacteria;D\_1\_\_FBP;D\_2\_\_uncultured bacterium;D\_3\_\_uncultured bacterium;D\_4\_\_uncultured bacterium | 0.0% | 0.0% | 0.0% | 0.0% | 0.0% | 0.0% | 0.0% | 0.0% | 0.1% |
|  | D\_0\_\_Bacteria;D\_1\_\_Fibrobacteres;D\_2\_\_Fibrobacteria;D\_3\_\_Fibrobacterales;D\_4\_\_Fibrobacteraceae | 0.0% | 0.0% | 0.0% | 0.0% | 0.0% | 0.0% | 0.0% | 0.0% | 0.0% |
|  | D\_0\_\_Bacteria;D\_1\_\_Firmicutes;D\_2\_\_Bacilli;D\_3\_\_Bacillales;D\_4\_\_Alicyclobacillaceae | 0.0% | 0.0% | 0.0% | 0.0% | 0.0% | 0.0% | 0.0% | 0.0% | 0.0% |
|  | D\_0\_\_Bacteria;D\_1\_\_Firmicutes;D\_2\_\_Bacilli;D\_3\_\_Bacillales;D\_4\_\_Bacillaceae | 0.0% | 0.0% | 0.0% | 0.0% | 0.0% | 0.0% | 0.0% | 0.0% | 0.1% |
|  | D\_0\_\_Bacteria;D\_1\_\_Firmicutes;D\_2\_\_Bacilli;D\_3\_\_Bacillales;D\_4\_\_Family X | 0.0% | 0.0% | 0.0% | 0.0% | 0.0% | 0.0% | 0.0% | 0.0% | 0.0% |
|  | D\_0\_\_Bacteria;D\_1\_\_Firmicutes;D\_2\_\_Bacilli;D\_3\_\_Bacillales;D\_4\_\_Family XI | 0.0% | 0.0% | 0.0% | 0.0% | 0.0% | 0.0% | 0.0% | 0.0% | 0.0% |
|  | D\_0\_\_Bacteria;D\_1\_\_Firmicutes;D\_2\_\_Bacilli;D\_3\_\_Bacillales;D\_4\_\_Paenibacillaceae | 0.0% | 0.0% | 0.0% | 0.1% | 0.1% | 0.0% | 0.0% | 0.0% | 0.0% |
|  | D\_0\_\_Bacteria;D\_1\_\_Firmicutes;D\_2\_\_Bacilli;D\_3\_\_Bacillales;D\_4\_\_Planococcaceae | 0.0% | 0.0% | 0.0% | 0.0% | 0.0% | 0.0% | 0.0% | 0.0% | 0.0% |
|  | D\_0\_\_Bacteria;D\_1\_\_Firmicutes;D\_2\_\_Bacilli;D\_3\_\_Bacillales;D\_4\_\_Staphylococcaceae | 0.0% | 0.0% | 0.0% | 0.0% | 0.0% | 0.0% | 0.0% | 0.1% | 0.2% |
|  | D\_0\_\_Bacteria;D\_1\_\_Firmicutes;D\_2\_\_Bacilli;D\_3\_\_Bacillales;D\_4\_\_Thermoactinomycetaceae | 0.0% | 0.0% | 0.0% | 0.0% | 0.0% | 0.0% | 0.0% | 0.0% | 0.0% |
|  | D\_0\_\_Bacteria;D\_1\_\_Firmicutes;D\_2\_\_Bacilli;D\_3\_\_Bacillales;Other | 0.0% | 0.0% | 0.0% | 0.0% | 0.0% | 0.0% | 0.0% | 0.0% | 0.0% |
|  | D\_0\_\_Bacteria;D\_1\_\_Firmicutes;D\_2\_\_Bacilli;D\_3\_\_Lactobacillales;D\_4\_\_Aerococcaceae | 0.0% | 0.0% | 0.0% | 0.0% | 0.0% | 0.0% | 0.0% | 0.0% | 0.0% |
|  | D\_0\_\_Bacteria;D\_1\_\_Firmicutes;D\_2\_\_Bacilli;D\_3\_\_Lactobacillales;D\_4\_\_Carnobacteriaceae | 0.0% | 0.0% | 0.0% | 0.0% | 0.0% | 0.0% | 0.0% | 0.0% | 0.1% |
|  | D\_0\_\_Bacteria;D\_1\_\_Firmicutes;D\_2\_\_Bacilli;D\_3\_\_Lactobacillales;D\_4\_\_Enterococcaceae | 0.0% | 0.0% | 0.0% | 0.0% | 0.0% | 0.0% | 0.0% | 0.0% | 0.0% |
|  | D\_0\_\_Bacteria;D\_1\_\_Firmicutes;D\_2\_\_Bacilli;D\_3\_\_Lactobacillales;D\_4\_\_Streptococcaceae | 0.1% | 0.0% | 0.0% | 0.0% | 0.0% | 0.0% | 0.0% | 0.1% | 0.7% |
|  | D\_0\_\_Bacteria;D\_1\_\_Firmicutes;D\_2\_\_Bacilli;D\_3\_\_Lactobacillales;Other | 0.0% | 0.0% | 0.0% | 0.0% | 0.0% | 0.0% | 0.0% | 0.0% | 0.0% |
|  | D\_0\_\_Bacteria;D\_1\_\_Firmicutes;D\_2\_\_Clostridia;D\_3\_\_Clostridiales;D\_4\_\_Clostridiaceae 1 | 0.0% | 0.0% | 0.0% | 0.0% | 0.0% | 0.0% | 0.0% | 0.0% | 0.0% |
|  | D\_0\_\_Bacteria;D\_1\_\_Firmicutes;D\_2\_\_Clostridia;D\_3\_\_Clostridiales;D\_4\_\_Family XI | 0.0% | 0.0% | 0.0% | 0.0% | 0.0% | 0.0% | 0.0% | 0.0% | 0.2% |
|  | D\_0\_\_Bacteria;D\_1\_\_Firmicutes;D\_2\_\_Clostridia;D\_3\_\_Clostridiales;D\_4\_\_Family XIII | 0.0% | 0.0% | 0.0% | 0.0% | 0.0% | 0.0% | 0.0% | 0.0% | 0.0% |
|  | D\_0\_\_Bacteria;D\_1\_\_Firmicutes;D\_2\_\_Clostridia;D\_3\_\_Clostridiales;D\_4\_\_Heliobacteriaceae | 0.0% | 0.0% | 0.0% | 0.0% | 0.0% | 0.0% | 0.0% | 0.0% | 0.0% |
|  | D\_0\_\_Bacteria;D\_1\_\_Firmicutes;D\_2\_\_Clostridia;D\_3\_\_Clostridiales;D\_4\_\_Lachnospiraceae | 0.0% | 0.0% | 0.0% | 0.0% | 0.0% | 0.0% | 0.0% | 0.0% | 0.2% |
|  | D\_0\_\_Bacteria;D\_1\_\_Firmicutes;D\_2\_\_Clostridia;D\_3\_\_Clostridiales;D\_4\_\_Peptococcaceae | 0.0% | 0.0% | 0.0% | 0.0% | 0.0% | 0.0% | 0.0% | 0.0% | 0.0% |
|  | D\_0\_\_Bacteria;D\_1\_\_Firmicutes;D\_2\_\_Clostridia;D\_3\_\_Clostridiales;D\_4\_\_Peptostreptococcaceae | 0.0% | 0.0% | 0.0% | 0.0% | 0.0% | 0.0% | 0.0% | 0.0% | 0.1% |
|  | D\_0\_\_Bacteria;D\_1\_\_Firmicutes;D\_2\_\_Clostridia;D\_3\_\_Clostridiales;D\_4\_\_Ruminococcaceae | 0.0% | 0.0% | 0.0% | 0.0% | 0.0% | 0.0% | 0.0% | 0.0% | 0.0% |
|  | D\_0\_\_Bacteria;D\_1\_\_Firmicutes;D\_2\_\_Clostridia;D\_3\_\_Halanaerobiales;D\_4\_\_ODP1230B8.23 | 0.0% | 0.0% | 0.0% | 0.0% | 0.0% | 0.0% | 0.0% | 0.0% | 0.0% |
|  | D\_0\_\_Bacteria;D\_1\_\_Firmicutes;D\_2\_\_Clostridia;D\_3\_\_Thermoanaerobacterales;D\_4\_\_Thermoanaerobacteraceae | 0.0% | 0.0% | 0.0% | 0.0% | 0.0% | 0.0% | 0.0% | 0.0% | 0.0% |
|  | D\_0\_\_Bacteria;D\_1\_\_Firmicutes;D\_2\_\_Erysipelotrichia;D\_3\_\_Erysipelotrichales;D\_4\_\_Erysipelotrichaceae | 0.0% | 0.0% | 0.0% | 0.0% | 0.0% | 0.0% | 0.0% | 0.0% | 0.1% |
|  | D\_0\_\_Bacteria;D\_1\_\_Firmicutes;D\_2\_\_Negativicutes;D\_3\_\_Selenomonadales;D\_4\_\_Veillonellaceae | 0.0% | 0.0% | 0.0% | 0.0% | 0.0% | 0.0% | 0.0% | 0.0% | 0.0% |
|  | D\_0\_\_Bacteria;D\_1\_\_Fusobacteria;D\_2\_\_Fusobacteriia;D\_3\_\_Fusobacteriales;D\_4\_\_Fusobacteriaceae | 0.1% | 0.0% | 0.0% | 0.0% | 0.0% | 0.0% | 0.0% | 0.1% | 0.5% |
|  | D\_0\_\_Bacteria;D\_1\_\_Fusobacteria;D\_2\_\_Fusobacteriia;D\_3\_\_Fusobacteriales;D\_4\_\_Leptotrichiaceae | 0.0% | 0.0% | 0.0% | 0.0% | 0.0% | 0.0% | 0.0% | 0.0% | 0.1% |
|  | D\_0\_\_Bacteria;D\_1\_\_Gemmatimonadetes;D\_2\_\_BD2-11 terrestrial group;D\_3\_\_uncultured bacterium;D\_4\_\_uncultured bacterium | 0.0% | 0.0% | 0.0% | 0.0% | 0.0% | 0.0% | 0.0% | 0.0% | 0.0% |
|  | D\_0\_\_Bacteria;D\_1\_\_Gemmatimonadetes;D\_2\_\_Gemmatimonadetes;D\_3\_\_Gemmatimonadales;D\_4\_\_Gemmatimonadaceae | 0.0% | 0.0% | 0.0% | 0.0% | 0.0% | 0.0% | 0.0% | 0.0% | 0.0% |
|  | D\_0\_\_Bacteria;D\_1\_\_Gemmatimonadetes;D\_2\_\_Longimicrobia;D\_3\_\_Longimicrobiales;D\_4\_\_Longimicrobiaceae | 0.3% | 0.3% | 0.3% | 0.2% | 0.2% | 0.8% | 0.2% | 0.1% | 0.1% |
|  | D\_0\_\_Bacteria;D\_1\_\_Gemmatimonadetes;D\_2\_\_S0134 terrestrial group;D\_3\_\_uncultured Gemmatimonadetes bacterium;D\_4\_\_uncultured Gemmatimonadetes bacterium | 0.0% | 0.0% | 0.0% | 0.0% | 0.0% | 0.0% | 0.0% | 0.0% | 0.0% |
|  | D\_0\_\_Bacteria;D\_1\_\_Gemmatimonadetes;D\_2\_\_S0134 terrestrial group;D\_3\_\_uncultured bacterium;D\_4\_\_uncultured bacterium | 0.0% | 0.0% | 0.0% | 0.0% | 0.0% | 0.0% | 0.0% | 0.0% | 0.1% |
|  | D\_0\_\_Bacteria;D\_1\_\_Hydrogenedentes;D\_2\_\_uncultured bacterium;D\_3\_\_uncultured bacterium;D\_4\_\_uncultured bacterium | 0.0% | 0.0% | 0.0% | 0.0% | 0.0% | 0.0% | 0.0% | 0.0% | 0.0% |
|  | D\_0\_\_Bacteria;D\_1\_\_Nitrospirae;D\_2\_\_Nitrospira;D\_3\_\_Nitrospirales;D\_4\_\_0319-6A21 | 0.0% | 0.0% | 0.0% | 0.0% | 0.0% | 0.0% | 0.0% | 0.0% | 0.0% |
|  | D\_0\_\_Bacteria;D\_1\_\_Nitrospirae;D\_2\_\_Nitrospira;D\_3\_\_Nitrospirales;D\_4\_\_Nitrospiraceae | 0.2% | 0.5% | 0.4% | 0.2% | 0.2% | 0.3% | 0.1% | 0.1% | 0.2% |
|  | D\_0\_\_Bacteria;D\_1\_\_Planctomycetes;D\_2\_\_Phycisphaerae;D\_3\_\_Phycisphaerales;D\_4\_\_Phycisphaeraceae | 0.0% | 0.0% | 0.0% | 0.0% | 0.0% | 0.0% | 0.0% | 0.0% | 0.0% |
|  | D\_0\_\_Bacteria;D\_1\_\_Planctomycetes;D\_2\_\_Phycisphaerae;D\_3\_\_Tepidisphaerales;D\_4\_\_Tepidisphaeraceae | 0.0% | 0.0% | 0.0% | 0.0% | 0.0% | 0.0% | 0.0% | 0.0% | 0.0% |
|  | D\_0\_\_Bacteria;D\_1\_\_Planctomycetes;D\_2\_\_Planctomycetacia;D\_3\_\_Planctomycetales;D\_4\_\_Planctomycetaceae | 0.1% | 0.1% | 0.1% | 0.2% | 0.2% | 0.0% | 0.0% | 0.1% | 0.1% |
|  | D\_0\_\_Bacteria;D\_1\_\_Proteobacteria;D\_2\_\_Alphaproteobacteria;D\_3\_\_Alphaproteobacteria Incertae Sedis;D\_4\_\_Unknown Family | 0.0% | 0.0% | 0.0% | 0.0% | 0.0% | 0.0% | 0.0% | 0.0% | 0.0% |
|  | D\_0\_\_Bacteria;D\_1\_\_Proteobacteria;D\_2\_\_Alphaproteobacteria;D\_3\_\_Caulobacterales;D\_4\_\_Caulobacteraceae | 0.1% | 0.2% | 0.2% | 0.1% | 0.1% | 0.1% | 0.0% | 0.1% | 0.1% |
|  | D\_0\_\_Bacteria;D\_1\_\_Proteobacteria;D\_2\_\_Alphaproteobacteria;D\_3\_\_Rhizobiales;D\_4\_\_1174-901-12 | 0.0% | 0.0% | 0.0% | 0.0% | 0.0% | 0.0% | 0.0% | 0.0% | 0.0% |
|  | D\_0\_\_Bacteria;D\_1\_\_Proteobacteria;D\_2\_\_Alphaproteobacteria;D\_3\_\_Rhizobiales;D\_4\_\_A0839 | 0.0% | 0.0% | 0.0% | 0.0% | 0.0% | 0.0% | 0.0% | 0.0% | 0.0% |
|  | D\_0\_\_Bacteria;D\_1\_\_Proteobacteria;D\_2\_\_Alphaproteobacteria;D\_3\_\_Rhizobiales;D\_4\_\_Aurantimonadaceae | 0.0% | 0.0% | 0.0% | 0.0% | 0.0% | 0.0% | 0.0% | 0.0% | 0.0% |
|  | D\_0\_\_Bacteria;D\_1\_\_Proteobacteria;D\_2\_\_Alphaproteobacteria;D\_3\_\_Rhizobiales;D\_4\_\_Beijerinckiaceae | 0.0% | 0.0% | 0.0% | 0.0% | 0.0% | 0.0% | 0.0% | 0.0% | 0.0% |
|  | D\_0\_\_Bacteria;D\_1\_\_Proteobacteria;D\_2\_\_Alphaproteobacteria;D\_3\_\_Rhizobiales;D\_4\_\_Bradyrhizobiaceae | 0.1% | 0.1% | 0.1% | 0.2% | 0.2% | 0.0% | 0.0% | 0.0% | 0.0% |
|  | D\_0\_\_Bacteria;D\_1\_\_Proteobacteria;D\_2\_\_Alphaproteobacteria;D\_3\_\_Rhizobiales;D\_4\_\_Brucellaceae | 0.0% | 0.0% | 0.0% | 0.0% | 0.0% | 0.0% | 0.0% | 0.0% | 0.0% |
|  | D\_0\_\_Bacteria;D\_1\_\_Proteobacteria;D\_2\_\_Alphaproteobacteria;D\_3\_\_Rhizobiales;D\_4\_\_C2U | 0.0% | 0.0% | 0.0% | 0.0% | 0.0% | 0.0% | 0.0% | 0.0% | 0.0% |
|  | D\_0\_\_Bacteria;D\_1\_\_Proteobacteria;D\_2\_\_Alphaproteobacteria;D\_3\_\_Rhizobiales;D\_4\_\_DUNssu044 | 0.1% | 0.1% | 0.1% | 0.2% | 0.2% | 0.4% | 0.1% | 0.1% | 0.0% |
|  | D\_0\_\_Bacteria;D\_1\_\_Proteobacteria;D\_2\_\_Alphaproteobacteria;D\_3\_\_Rhizobiales;D\_4\_\_FukuN57 | 0.0% | 0.0% | 0.0% | 0.0% | 0.0% | 0.0% | 0.0% | 0.0% | 0.0% |
|  | D\_0\_\_Bacteria;D\_1\_\_Proteobacteria;D\_2\_\_Alphaproteobacteria;D\_3\_\_Rhizobiales;D\_4\_\_Hyphomicrobiaceae | 0.1% | 0.0% | 0.0% | 0.1% | 0.1% | 0.2% | 0.2% | 0.0% | 0.0% |
|  | D\_0\_\_Bacteria;D\_1\_\_Proteobacteria;D\_2\_\_Alphaproteobacteria;D\_3\_\_Rhizobiales;D\_4\_\_JG34-KF-361 | 0.0% | 0.0% | 0.0% | 0.0% | 0.0% | 0.0% | 0.0% | 0.0% | 0.0% |
|  | D\_0\_\_Bacteria;D\_1\_\_Proteobacteria;D\_2\_\_Alphaproteobacteria;D\_3\_\_Rhizobiales;D\_4\_\_KF-JG30-B3 | 0.0% | 0.0% | 0.0% | 0.0% | 0.0% | 0.0% | 0.0% | 0.0% | 0.0% |
|  | D\_0\_\_Bacteria;D\_1\_\_Proteobacteria;D\_2\_\_Alphaproteobacteria;D\_3\_\_Rhizobiales;D\_4\_\_MNG7 | 0.0% | 0.0% | 0.0% | 0.0% | 0.0% | 0.0% | 0.0% | 0.0% | 0.0% |
|  | D\_0\_\_Bacteria;D\_1\_\_Proteobacteria;D\_2\_\_Alphaproteobacteria;D\_3\_\_Rhizobiales;D\_4\_\_Methylobacteriaceae | 0.0% | 0.0% | 0.0% | 0.0% | 0.0% | 0.0% | 0.0% | 0.0% | 0.0% |
|  | D\_0\_\_Bacteria;D\_1\_\_Proteobacteria;D\_2\_\_Alphaproteobacteria;D\_3\_\_Rhizobiales;D\_4\_\_Methylocystaceae | 0.0% | 0.0% | 0.0% | 0.0% | 0.0% | 0.0% | 0.0% | 0.0% | 0.0% |
|  | D\_0\_\_Bacteria;D\_1\_\_Proteobacteria;D\_2\_\_Alphaproteobacteria;D\_3\_\_Rhizobiales;D\_4\_\_Phyllobacteriaceae | 0.1% | 0.1% | 0.1% | 0.1% | 0.1% | 0.1% | 0.0% | 0.0% | 0.1% |
|  | D\_0\_\_Bacteria;D\_1\_\_Proteobacteria;D\_2\_\_Alphaproteobacteria;D\_3\_\_Rhizobiales;D\_4\_\_Rhizobiaceae | 0.0% | 0.0% | 0.0% | 0.0% | 0.0% | 0.0% | 0.0% | 0.0% | 0.3% |
|  | D\_0\_\_Bacteria;D\_1\_\_Proteobacteria;D\_2\_\_Alphaproteobacteria;D\_3\_\_Rhizobiales;D\_4\_\_Rhizobiales Incertae Sedis | 0.0% | 0.0% | 0.0% | 0.0% | 0.0% | 0.0% | 0.0% | 0.0% | 0.1% |
|  | D\_0\_\_Bacteria;D\_1\_\_Proteobacteria;D\_2\_\_Alphaproteobacteria;D\_3\_\_Rhizobiales;D\_4\_\_Rhodobiaceae | 0.1% | 0.2% | 0.1% | 0.1% | 0.1% | 0.0% | 0.0% | 0.0% | 0.0% |
|  | D\_0\_\_Bacteria;D\_1\_\_Proteobacteria;D\_2\_\_Alphaproteobacteria;D\_3\_\_Rhizobiales;D\_4\_\_Roseiarcaceae | 0.0% | 0.0% | 0.0% | 0.0% | 0.0% | 0.0% | 0.0% | 0.0% | 0.0% |
|  | D\_0\_\_Bacteria;D\_1\_\_Proteobacteria;D\_2\_\_Alphaproteobacteria;D\_3\_\_Rhizobiales;D\_4\_\_Xanthobacteraceae | 1.1% | 1.2% | 1.0% | 1.0% | 1.0% | 1.7% | 2.1% | 0.3% | 0.8% |
|  | D\_0\_\_Bacteria;D\_1\_\_Proteobacteria;D\_2\_\_Alphaproteobacteria;D\_3\_\_Rhizobiales;D\_4\_\_alphaI cluster | 0.0% | 0.0% | 0.0% | 0.0% | 0.0% | 0.0% | 0.0% | 0.0% | 0.1% |
|  | D\_0\_\_Bacteria;D\_1\_\_Proteobacteria;D\_2\_\_Alphaproteobacteria;D\_3\_\_Rhizobiales;D\_4\_\_uncultured | 0.1% | 0.0% | 0.0% | 0.0% | 0.0% | 0.3% | 0.3% | 0.0% | 0.0% |
|  | D\_0\_\_Bacteria;D\_1\_\_Proteobacteria;D\_2\_\_Alphaproteobacteria;D\_3\_\_Rhizobiales;Other | 0.0% | 0.0% | 0.0% | 0.1% | 0.1% | 0.0% | 0.0% | 0.0% | 0.0% |
|  | D\_0\_\_Bacteria;D\_1\_\_Proteobacteria;D\_2\_\_Alphaproteobacteria;D\_3\_\_Rhodobacterales;D\_4\_\_Rhodobacteraceae | 0.1% | 0.0% | 0.0% | 0.0% | 0.0% | 0.0% | 0.0% | 0.2% | 0.6% |
|  | D\_0\_\_Bacteria;D\_1\_\_Proteobacteria;D\_2\_\_Alphaproteobacteria;D\_3\_\_Rhodospirillales;D\_4\_\_AT-s3-44 | 0.0% | 0.0% | 0.0% | 0.0% | 0.0% | 0.0% | 0.0% | 0.0% | 0.0% |
|  | D\_0\_\_Bacteria;D\_1\_\_Proteobacteria;D\_2\_\_Alphaproteobacteria;D\_3\_\_Rhodospirillales;D\_4\_\_Acetobacteraceae | 0.1% | 0.1% | 0.1% | 0.1% | 0.1% | 0.0% | 0.0% | 0.1% | 0.0% |
|  | D\_0\_\_Bacteria;D\_1\_\_Proteobacteria;D\_2\_\_Alphaproteobacteria;D\_3\_\_Rhodospirillales;D\_4\_\_DA111 | 0.0% | 0.0% | 0.0% | 0.0% | 0.0% | 0.0% | 0.0% | 0.0% | 0.0% |
|  | D\_0\_\_Bacteria;D\_1\_\_Proteobacteria;D\_2\_\_Alphaproteobacteria;D\_3\_\_Rhodospirillales;D\_4\_\_KCM-B-15 | 0.0% | 0.0% | 0.0% | 0.0% | 0.0% | 0.0% | 0.0% | 0.0% | 0.0% |
|  | D\_0\_\_Bacteria;D\_1\_\_Proteobacteria;D\_2\_\_Alphaproteobacteria;D\_3\_\_Rhodospirillales;D\_4\_\_MND8 | 0.0% | 0.0% | 0.0% | 0.0% | 0.0% | 0.1% | 0.0% | 0.0% | 0.1% |
|  | D\_0\_\_Bacteria;D\_1\_\_Proteobacteria;D\_2\_\_Alphaproteobacteria;D\_3\_\_Rhodospirillales;D\_4\_\_MSB-1E8 | 0.0% | 0.0% | 0.0% | 0.0% | 0.0% | 0.0% | 0.0% | 0.0% | 0.0% |
|  | D\_0\_\_Bacteria;D\_1\_\_Proteobacteria;D\_2\_\_Alphaproteobacteria;D\_3\_\_Rhodospirillales;D\_4\_\_Rhodospirillaceae | 2.9% | 4.9% | 4.2% | 4.4% | 4.6% | 2.5% | 1.5% | 0.3% | 0.5% |
|  | D\_0\_\_Bacteria;D\_1\_\_Proteobacteria;D\_2\_\_Alphaproteobacteria;D\_3\_\_Rhodospirillales;D\_4\_\_Rhodospirillales Incertae Sedis | 0.2% | 0.1% | 0.1% | 0.2% | 0.1% | 0.6% | 0.2% | 0.1% | 0.0% |
|  | D\_0\_\_Bacteria;D\_1\_\_Proteobacteria;D\_2\_\_Alphaproteobacteria;D\_3\_\_Rhodospirillales;Other | 0.0% | 0.0% | 0.0% | 0.0% | 0.0% | 0.0% | 0.0% | 0.0% | 0.0% |
|  | D\_0\_\_Bacteria;D\_1\_\_Proteobacteria;D\_2\_\_Alphaproteobacteria;D\_3\_\_Rickettsiales;D\_4\_\_EF100-94H03 | 0.0% | 0.0% | 0.0% | 0.0% | 0.0% | 0.0% | 0.0% | 0.0% | 0.0% |
|  | D\_0\_\_Bacteria;D\_1\_\_Proteobacteria;D\_2\_\_Alphaproteobacteria;D\_3\_\_Rickettsiales;D\_4\_\_Mitochondria | 0.0% | 0.0% | 0.0% | 0.0% | 0.0% | 0.0% | 0.0% | 0.0% | 0.1% |
|  | D\_0\_\_Bacteria;D\_1\_\_Proteobacteria;D\_2\_\_Alphaproteobacteria;D\_3\_\_Rickettsiales;D\_4\_\_Rickettsiaceae | 0.0% | 0.0% | 0.0% | 0.0% | 0.0% | 0.0% | 0.0% | 0.0% | 0.0% |
|  | D\_0\_\_Bacteria;D\_1\_\_Proteobacteria;D\_2\_\_Alphaproteobacteria;D\_3\_\_Rickettsiales;D\_4\_\_SM2D12 | 0.0% | 0.0% | 0.0% | 0.0% | 0.0% | 0.0% | 0.0% | 0.0% | 0.0% |
|  | D\_0\_\_Bacteria;D\_1\_\_Proteobacteria;D\_2\_\_Alphaproteobacteria;D\_3\_\_Rickettsiales;D\_4\_\_TK34 | 0.0% | 0.0% | 0.0% | 0.0% | 0.0% | 0.0% | 0.0% | 0.0% | 0.0% |
|  | D\_0\_\_Bacteria;D\_1\_\_Proteobacteria;D\_2\_\_Alphaproteobacteria;D\_3\_\_Sphingomonadales;D\_4\_\_7B-8 | 0.0% | 0.0% | 0.0% | 0.0% | 0.0% | 0.0% | 0.0% | 0.0% | 0.0% |
|  | D\_0\_\_Bacteria;D\_1\_\_Proteobacteria;D\_2\_\_Alphaproteobacteria;D\_3\_\_Sphingomonadales;D\_4\_\_Ellin6055 | 0.0% | 0.0% | 0.0% | 0.0% | 0.0% | 0.0% | 0.0% | 0.0% | 0.0% |
|  | D\_0\_\_Bacteria;D\_1\_\_Proteobacteria;D\_2\_\_Alphaproteobacteria;D\_3\_\_Sphingomonadales;D\_4\_\_Erythrobacteraceae | 0.0% | 0.0% | 0.0% | 0.0% | 0.0% | 0.0% | 0.0% | 0.0% | 0.0% |
|  | D\_0\_\_Bacteria;D\_1\_\_Proteobacteria;D\_2\_\_Alphaproteobacteria;D\_3\_\_Sphingomonadales;D\_4\_\_Sphingomonadaceae | 0.0% | 0.0% | 0.0% | 0.0% | 0.0% | 0.0% | 0.0% | 0.0% | 0.0% |
|  | D\_0\_\_Bacteria;D\_1\_\_Proteobacteria;D\_2\_\_Alphaproteobacteria;D\_3\_\_Sphingomonadales;Other | 0.0% | 0.0% | 0.0% | 0.0% | 0.0% | 0.0% | 0.0% | 0.0% | 0.0% |
|  | D\_0\_\_Bacteria;D\_1\_\_Proteobacteria;D\_2\_\_Alphaproteobacteria;Other;Other | 0.0% | 0.0% | 0.0% | 0.0% | 0.0% | 0.0% | 0.0% | 0.0% | 0.0% |
|  | D\_0\_\_Bacteria;D\_1\_\_Proteobacteria;D\_2\_\_Betaproteobacteria;D\_3\_\_Burkholderiales;D\_4\_\_Alcaligenaceae | 0.0% | 0.0% | 0.0% | 0.0% | 0.0% | 0.0% | 0.0% | 0.0% | 0.0% |
|  | D\_0\_\_Bacteria;D\_1\_\_Proteobacteria;D\_2\_\_Betaproteobacteria;D\_3\_\_Burkholderiales;D\_4\_\_Burkholderiaceae | 0.0% | 0.0% | 0.0% | 0.0% | 0.0% | 0.0% | 0.0% | 0.0% | 0.1% |
|  | D\_0\_\_Bacteria;D\_1\_\_Proteobacteria;D\_2\_\_Betaproteobacteria;D\_3\_\_Burkholderiales;D\_4\_\_Comamonadaceae | 0.1% | 0.0% | 0.0% | 0.0% | 0.0% | 0.0% | 0.0% | 0.0% | 0.4% |
|  | D\_0\_\_Bacteria;D\_1\_\_Proteobacteria;D\_2\_\_Betaproteobacteria;D\_3\_\_Burkholderiales;D\_4\_\_Oxalobacteraceae | 0.0% | 0.0% | 0.0% | 0.0% | 0.0% | 0.0% | 0.0% | 0.0% | 0.1% |
|  | D\_0\_\_Bacteria;D\_1\_\_Proteobacteria;D\_2\_\_Betaproteobacteria;D\_3\_\_Hydrogenophilales;D\_4\_\_Hydrogenophilaceae | 0.0% | 0.0% | 0.0% | 0.0% | 0.0% | 0.3% | 0.1% | 0.0% | 0.0% |
|  | D\_0\_\_Bacteria;D\_1\_\_Proteobacteria;D\_2\_\_Betaproteobacteria;D\_3\_\_Methylophilales;D\_4\_\_Methylophilaceae | 0.0% | 0.0% | 0.0% | 0.0% | 0.0% | 0.0% | 0.0% | 0.0% | 0.0% |
|  | D\_0\_\_Bacteria;D\_1\_\_Proteobacteria;D\_2\_\_Betaproteobacteria;D\_3\_\_Neisseriales;D\_4\_\_Neisseriaceae | 0.1% | 0.0% | 0.0% | 0.0% | 0.0% | 0.0% | 0.0% | 0.1% | 0.8% |
|  | D\_0\_\_Bacteria;D\_1\_\_Proteobacteria;D\_2\_\_Betaproteobacteria;D\_3\_\_Nitrosomonadales;D\_4\_\_Gallionellaceae | 0.0% | 0.0% | 0.0% | 0.0% | 0.0% | 0.2% | 0.0% | 0.0% | 0.0% |
|  | D\_0\_\_Bacteria;D\_1\_\_Proteobacteria;D\_2\_\_Betaproteobacteria;D\_3\_\_Nitrosomonadales;D\_4\_\_Nitrosomonadaceae | 0.0% | 0.0% | 0.0% | 0.0% | 0.0% | 0.0% | 0.0% | 0.0% | 0.0% |
|  | D\_0\_\_Bacteria;D\_1\_\_Proteobacteria;D\_2\_\_Betaproteobacteria;D\_3\_\_Rhodocyclales;D\_4\_\_Rhodocyclaceae | 0.0% | 0.0% | 0.0% | 0.0% | 0.0% | 0.0% | 0.0% | 0.0% | 0.0% |
|  | D\_0\_\_Bacteria;D\_1\_\_Proteobacteria;D\_2\_\_Betaproteobacteria;D\_3\_\_SC-I-84;D\_4\_\_uncultured bacterium | 0.0% | 0.0% | 0.0% | 0.0% | 0.0% | 0.0% | 0.0% | 0.0% | 0.0% |
|  | D\_0\_\_Bacteria;D\_1\_\_Proteobacteria;D\_2\_\_Betaproteobacteria;D\_3\_\_TRA3-20;Ambiguous\_taxa | 0.0% | 0.0% | 0.0% | 0.0% | 0.0% | 0.0% | 0.0% | 0.0% | 0.0% |
|  | D\_0\_\_Bacteria;D\_1\_\_Proteobacteria;D\_2\_\_Betaproteobacteria;D\_3\_\_TRA3-20;D\_4\_\_uncultured bacterium | 1.7% | 3.1% | 2.9% | 3.7% | 3.2% | 0.2% | 0.0% | 0.1% | 0.1% |
|  | D\_0\_\_Bacteria;D\_1\_\_Proteobacteria;D\_2\_\_Betaproteobacteria;D\_3\_\_TRA3-20;D\_4\_\_uncultured gamma proteobacterium | 0.0% | 0.0% | 0.0% | 0.0% | 0.0% | 0.0% | 0.0% | 0.0% | 0.0% |
|  | D\_0\_\_Bacteria;D\_1\_\_Proteobacteria;D\_2\_\_Betaproteobacteria;D\_3\_\_TRA3-20;Other | 0.0% | 0.0% | 0.0% | 0.0% | 0.0% | 0.0% | 0.0% | 0.0% | 0.0% |
|  | D\_0\_\_Bacteria;D\_1\_\_Proteobacteria;D\_2\_\_Betaproteobacteria;Other;Other | 0.0% | 0.0% | 0.0% | 0.0% | 0.0% | 0.0% | 0.0% | 0.0% | 0.0% |
|  | D\_0\_\_Bacteria;D\_1\_\_Proteobacteria;D\_2\_\_Deltaproteobacteria;D\_3\_\_Bdellovibrionales;D\_4\_\_Bacteriovoracaceae | 0.0% | 0.0% | 0.0% | 0.0% | 0.0% | 0.0% | 0.0% | 0.0% | 0.0% |
|  | D\_0\_\_Bacteria;D\_1\_\_Proteobacteria;D\_2\_\_Deltaproteobacteria;D\_3\_\_Bdellovibrionales;D\_4\_\_Bdellovibrionaceae | 0.0% | 0.0% | 0.0% | 0.0% | 0.0% | 0.0% | 0.0% | 0.0% | 0.0% |
|  | D\_0\_\_Bacteria;D\_1\_\_Proteobacteria;D\_2\_\_Deltaproteobacteria;D\_3\_\_Desulfurellales;D\_4\_\_Desulfurellaceae | 4.2% | 8.1% | 7.9% | 7.0% | 7.9% | 1.1% | 0.6% | 0.3% | 0.6% |
|  | D\_0\_\_Bacteria;D\_1\_\_Proteobacteria;D\_2\_\_Deltaproteobacteria;D\_3\_\_Myxococcales;Ambiguous\_taxa | 0.0% | 0.0% | 0.0% | 0.0% | 0.0% | 0.0% | 0.0% | 0.0% | 0.0% |
|  | D\_0\_\_Bacteria;D\_1\_\_Proteobacteria;D\_2\_\_Deltaproteobacteria;D\_3\_\_Myxococcales;D\_4\_\_Archangiaceae | 0.0% | 0.0% | 0.0% | 0.0% | 0.0% | 0.0% | 0.0% | 0.0% | 0.0% |
|  | D\_0\_\_Bacteria;D\_1\_\_Proteobacteria;D\_2\_\_Deltaproteobacteria;D\_3\_\_Myxococcales;D\_4\_\_BIrii41 | 0.0% | 0.0% | 0.0% | 0.0% | 0.0% | 0.0% | 0.0% | 0.0% | 0.0% |
|  | D\_0\_\_Bacteria;D\_1\_\_Proteobacteria;D\_2\_\_Deltaproteobacteria;D\_3\_\_Myxococcales;D\_4\_\_Haliangiaceae | 0.0% | 0.0% | 0.0% | 0.0% | 0.0% | 0.0% | 0.0% | 0.0% | 0.0% |
|  | D\_0\_\_Bacteria;D\_1\_\_Proteobacteria;D\_2\_\_Deltaproteobacteria;D\_3\_\_Myxococcales;D\_4\_\_Nannocystaceae | 0.0% | 0.0% | 0.0% | 0.0% | 0.0% | 0.0% | 0.0% | 0.0% | 0.0% |
|  | D\_0\_\_Bacteria;D\_1\_\_Proteobacteria;D\_2\_\_Deltaproteobacteria;D\_3\_\_Myxococcales;D\_4\_\_P3OB-42 | 0.0% | 0.0% | 0.0% | 0.0% | 0.0% | 0.0% | 0.0% | 0.0% | 0.0% |
|  | D\_0\_\_Bacteria;D\_1\_\_Proteobacteria;D\_2\_\_Deltaproteobacteria;D\_3\_\_Myxococcales;D\_4\_\_Phaselicystidaceae | 0.0% | 0.0% | 0.0% | 0.0% | 0.0% | 0.0% | 0.0% | 0.0% | 0.0% |
|  | D\_0\_\_Bacteria;D\_1\_\_Proteobacteria;D\_2\_\_Deltaproteobacteria;D\_3\_\_Myxococcales;D\_4\_\_Polyangiaceae | 0.0% | 0.0% | 0.0% | 0.0% | 0.0% | 0.0% | 0.0% | 0.0% | 0.0% |
|  | D\_0\_\_Bacteria;D\_1\_\_Proteobacteria;D\_2\_\_Deltaproteobacteria;D\_3\_\_Myxococcales;D\_4\_\_Sandaracinaceae | 0.0% | 0.0% | 0.0% | 0.0% | 0.0% | 0.0% | 0.0% | 0.0% | 0.0% |
|  | D\_0\_\_Bacteria;D\_1\_\_Proteobacteria;D\_2\_\_Deltaproteobacteria;D\_3\_\_Myxococcales;D\_4\_\_VHS-B3-70 | 0.0% | 0.0% | 0.0% | 0.0% | 0.0% | 0.0% | 0.0% | 0.0% | 0.0% |
|  | D\_0\_\_Bacteria;D\_1\_\_Proteobacteria;D\_2\_\_Deltaproteobacteria;D\_3\_\_Myxococcales;D\_4\_\_mle1-27 | 0.0% | 0.0% | 0.0% | 0.0% | 0.0% | 0.0% | 0.0% | 0.0% | 0.0% |
|  | D\_0\_\_Bacteria;D\_1\_\_Proteobacteria;D\_2\_\_Deltaproteobacteria;D\_3\_\_Myxococcales;D\_4\_\_uncultured | 0.0% | 0.0% | 0.0% | 0.0% | 0.0% | 0.0% | 0.0% | 0.0% | 0.0% |
|  | D\_0\_\_Bacteria;D\_1\_\_Proteobacteria;D\_2\_\_Deltaproteobacteria;D\_3\_\_Myxococcales;D\_4\_\_uncultured bacterium | 0.0% | 0.0% | 0.0% | 0.0% | 0.0% | 0.0% | 0.0% | 0.0% | 0.0% |
|  | D\_0\_\_Bacteria;D\_1\_\_Proteobacteria;D\_2\_\_Deltaproteobacteria;D\_3\_\_Myxococcales;Other | 0.0% | 0.0% | 0.0% | 0.0% | 0.0% | 0.0% | 0.0% | 0.0% | 0.0% |
|  | D\_0\_\_Bacteria;D\_1\_\_Proteobacteria;D\_2\_\_Deltaproteobacteria;D\_3\_\_Oligoflexales;D\_4\_\_0319-6G20 | 0.0% | 0.0% | 0.0% | 0.0% | 0.0% | 0.0% | 0.0% | 0.0% | 0.0% |
|  | D\_0\_\_Bacteria;D\_1\_\_Proteobacteria;D\_2\_\_Deltaproteobacteria;D\_3\_\_Oligoflexales;D\_4\_\_Oligoflexaceae | 0.0% | 0.0% | 0.0% | 0.0% | 0.0% | 0.0% | 0.0% | 0.0% | 0.0% |
|  | D\_0\_\_Bacteria;D\_1\_\_Proteobacteria;D\_2\_\_Deltaproteobacteria;D\_3\_\_SAR324 clade(Marine group B);D\_4\_\_uncultured bacterium | 0.0% | 0.0% | 0.0% | 0.0% | 0.0% | 0.0% | 0.0% | 0.0% | 0.0% |
|  | D\_0\_\_Bacteria;D\_1\_\_Proteobacteria;D\_2\_\_Deltaproteobacteria;D\_3\_\_SAR324 clade(Marine group B);D\_4\_\_uncultured delta proteobacterium | 0.0% | 0.0% | 0.0% | 0.0% | 0.0% | 0.0% | 0.0% | 0.0% | 0.0% |
|  | D\_0\_\_Bacteria;D\_1\_\_Proteobacteria;D\_2\_\_Epsilonproteobacteria;D\_3\_\_Campylobacterales;D\_4\_\_Campylobacteraceae | 0.0% | 0.0% | 0.0% | 0.0% | 0.0% | 0.0% | 0.0% | 0.0% | 0.0% |
|  | D\_0\_\_Bacteria;D\_1\_\_Proteobacteria;D\_2\_\_Gammaproteobacteria;D\_3\_\_Alteromonadales;D\_4\_\_Alteromonadaceae | 0.0% | 0.0% | 0.0% | 0.0% | 0.0% | 0.0% | 0.0% | 0.0% | 0.0% |
|  | D\_0\_\_Bacteria;D\_1\_\_Proteobacteria;D\_2\_\_Gammaproteobacteria;D\_3\_\_Alteromonadales;D\_4\_\_Psychromonadaceae | 0.0% | 0.0% | 0.0% | 0.0% | 0.0% | 0.0% | 0.0% | 0.0% | 0.0% |
|  | D\_0\_\_Bacteria;D\_1\_\_Proteobacteria;D\_2\_\_Gammaproteobacteria;D\_3\_\_Cardiobacteriales;D\_4\_\_Cardiobacteriaceae | 0.0% | 0.0% | 0.0% | 0.0% | 0.0% | 0.0% | 0.0% | 0.0% | 0.0% |
|  | D\_0\_\_Bacteria;D\_1\_\_Proteobacteria;D\_2\_\_Gammaproteobacteria;D\_3\_\_Cellvibrionales;D\_4\_\_Cellvibrionaceae | 0.0% | 0.0% | 0.0% | 0.0% | 0.0% | 0.0% | 0.0% | 0.0% | 0.0% |
|  | D\_0\_\_Bacteria;D\_1\_\_Proteobacteria;D\_2\_\_Gammaproteobacteria;D\_3\_\_Chromatiales;D\_4\_\_Chromatiaceae | 0.0% | 0.0% | 0.0% | 0.0% | 0.0% | 0.0% | 0.0% | 0.0% | 0.0% |
|  | D\_0\_\_Bacteria;D\_1\_\_Proteobacteria;D\_2\_\_Gammaproteobacteria;D\_3\_\_Chromatiales;D\_4\_\_Ectothiorhodospiraceae | 0.0% | 0.0% | 0.0% | 0.0% | 0.0% | 0.0% | 0.0% | 0.0% | 0.0% |
|  | D\_0\_\_Bacteria;D\_1\_\_Proteobacteria;D\_2\_\_Gammaproteobacteria;D\_3\_\_Chromatiales;Other | 0.0% | 0.0% | 0.0% | 0.0% | 0.0% | 0.0% | 0.0% | 0.0% | 0.0% |
|  | D\_0\_\_Bacteria;D\_1\_\_Proteobacteria;D\_2\_\_Gammaproteobacteria;D\_3\_\_Enterobacteriales;D\_4\_\_Enterobacteriaceae | 0.0% | 0.0% | 0.0% | 0.0% | 0.0% | 0.1% | 0.0% | 0.0% | 0.1% |
|  | D\_0\_\_Bacteria;D\_1\_\_Proteobacteria;D\_2\_\_Gammaproteobacteria;D\_3\_\_Gammaproteobacteria Incertae Sedis;D\_4\_\_Unknown Family | 0.0% | 0.0% | 0.0% | 0.0% | 0.0% | 0.0% | 0.0% | 0.0% | 0.0% |
|  | D\_0\_\_Bacteria;D\_1\_\_Proteobacteria;D\_2\_\_Gammaproteobacteria;D\_3\_\_HTA4;D\_4\_\_uncultured bacterium | 0.0% | 0.0% | 0.0% | 0.0% | 0.0% | 0.0% | 0.0% | 0.0% | 0.0% |
|  | D\_0\_\_Bacteria;D\_1\_\_Proteobacteria;D\_2\_\_Gammaproteobacteria;D\_3\_\_KI89A clade;D\_4\_\_uncultured bacterium | 0.0% | 0.0% | 0.0% | 0.0% | 0.0% | 0.0% | 0.0% | 0.0% | 0.0% |
|  | D\_0\_\_Bacteria;D\_1\_\_Proteobacteria;D\_2\_\_Gammaproteobacteria;D\_3\_\_Legionellales;D\_4\_\_Coxiellaceae | 0.0% | 0.0% | 0.0% | 0.0% | 0.0% | 0.0% | 0.0% | 0.0% | 0.0% |
|  | D\_0\_\_Bacteria;D\_1\_\_Proteobacteria;D\_2\_\_Gammaproteobacteria;D\_3\_\_Legionellales;D\_4\_\_Legionellaceae | 0.0% | 0.0% | 0.0% | 0.0% | 0.0% | 0.0% | 0.0% | 0.0% | 0.0% |
|  | D\_0\_\_Bacteria;D\_1\_\_Proteobacteria;D\_2\_\_Gammaproteobacteria;D\_3\_\_Oceanospirillales;D\_4\_\_Halomonadaceae | 0.0% | 0.0% | 0.0% | 0.0% | 0.0% | 0.0% | 0.0% | 0.0% | 0.0% |
|  | D\_0\_\_Bacteria;D\_1\_\_Proteobacteria;D\_2\_\_Gammaproteobacteria;D\_3\_\_Oceanospirillales;D\_4\_\_Oceanospirillaceae | 0.0% | 0.0% | 0.0% | 0.0% | 0.0% | 0.0% | 0.0% | 0.0% | 0.0% |
|  | D\_0\_\_Bacteria;D\_1\_\_Proteobacteria;D\_2\_\_Gammaproteobacteria;D\_3\_\_PYR10d3;Other | 0.0% | 0.0% | 0.0% | 0.0% | 0.0% | 0.0% | 0.0% | 0.0% | 0.0% |
|  | D\_0\_\_Bacteria;D\_1\_\_Proteobacteria;D\_2\_\_Gammaproteobacteria;D\_3\_\_Pasteurellales;D\_4\_\_Pasteurellaceae | 0.1% | 0.0% | 0.0% | 0.0% | 0.0% | 0.0% | 0.0% | 0.0% | 0.5% |
|  | D\_0\_\_Bacteria;D\_1\_\_Proteobacteria;D\_2\_\_Gammaproteobacteria;D\_3\_\_Pseudomonadales;D\_4\_\_Moraxellaceae | 0.0% | 0.0% | 0.0% | 0.0% | 0.0% | 0.0% | 0.0% | 0.1% | 0.3% |
|  | D\_0\_\_Bacteria;D\_1\_\_Proteobacteria;D\_2\_\_Gammaproteobacteria;D\_3\_\_Pseudomonadales;D\_4\_\_Pseudomonadaceae | 0.1% | 0.0% | 0.0% | 0.0% | 0.0% | 0.0% | 0.0% | 0.0% | 0.5% |
|  | D\_0\_\_Bacteria;D\_1\_\_Proteobacteria;D\_2\_\_Gammaproteobacteria;D\_3\_\_Thiotrichales;D\_4\_\_Piscirickettsiaceae | 0.0% | 0.0% | 0.0% | 0.0% | 0.0% | 0.0% | 0.0% | 0.0% | 0.0% |
|  | D\_0\_\_Bacteria;D\_1\_\_Proteobacteria;D\_2\_\_Gammaproteobacteria;D\_3\_\_Thiotrichales;D\_4\_\_Thiotrichaceae | 0.0% | 0.0% | 0.0% | 0.0% | 0.0% | 0.0% | 0.0% | 0.0% | 0.0% |
|  | D\_0\_\_Bacteria;D\_1\_\_Proteobacteria;D\_2\_\_Gammaproteobacteria;D\_3\_\_Vibrionales;D\_4\_\_Vibrionaceae | 0.0% | 0.0% | 0.0% | 0.0% | 0.0% | 0.0% | 0.0% | 0.0% | 0.0% |
|  | D\_0\_\_Bacteria;D\_1\_\_Proteobacteria;D\_2\_\_Gammaproteobacteria;D\_3\_\_X35;Other | 0.0% | 0.0% | 0.0% | 0.0% | 0.0% | 0.0% | 0.0% | 0.0% | 0.0% |
|  | D\_0\_\_Bacteria;D\_1\_\_Proteobacteria;D\_2\_\_Gammaproteobacteria;D\_3\_\_Xanthomonadales;D\_4\_\_JTB255 marine benthic group | 0.1% | 0.0% | 0.0% | 0.1% | 0.1% | 0.1% | 0.0% | 0.0% | 0.0% |
|  | D\_0\_\_Bacteria;D\_1\_\_Proteobacteria;D\_2\_\_Gammaproteobacteria;D\_3\_\_Xanthomonadales;D\_4\_\_Nevskiaceae | 0.0% | 0.0% | 0.0% | 0.0% | 0.0% | 0.0% | 0.0% | 0.0% | 0.0% |
|  | D\_0\_\_Bacteria;D\_1\_\_Proteobacteria;D\_2\_\_Gammaproteobacteria;D\_3\_\_Xanthomonadales;D\_4\_\_Xanthomonadaceae | 0.0% | 0.0% | 0.0% | 0.0% | 0.0% | 0.0% | 0.0% | 0.0% | 0.2% |
|  | D\_0\_\_Bacteria;D\_1\_\_Proteobacteria;D\_2\_\_Gammaproteobacteria;D\_3\_\_Xanthomonadales;D\_4\_\_Xanthomonadales Incertae Sedis | 0.0% | 0.0% | 0.0% | 0.0% | 0.0% | 0.0% | 0.0% | 0.0% | 0.0% |
|  | D\_0\_\_Bacteria;D\_1\_\_Proteobacteria;D\_2\_\_Gammaproteobacteria;D\_3\_\_Xanthomonadales;D\_4\_\_uncultured | 0.0% | 0.0% | 0.0% | 0.0% | 0.0% | 0.0% | 0.0% | 0.0% | 0.0% |
|  | D\_0\_\_Bacteria;D\_1\_\_Proteobacteria;D\_2\_\_Gammaproteobacteria;D\_3\_\_Xanthomonadales;Other | 0.0% | 0.0% | 0.0% | 0.0% | 0.0% | 0.0% | 0.0% | 0.0% | 0.0% |
|  | D\_0\_\_Bacteria;D\_1\_\_Proteobacteria;D\_2\_\_Gammaproteobacteria;D\_3\_\_uncultured;D\_4\_\_uncultured bacterium | 0.0% | 0.0% | 0.0% | 0.0% | 0.0% | 0.0% | 0.0% | 0.0% | 0.0% |
|  | D\_0\_\_Bacteria;D\_1\_\_Proteobacteria;D\_2\_\_Gammaproteobacteria;D\_3\_\_uncultured;Other | 0.0% | 0.0% | 0.0% | 0.0% | 0.0% | 0.0% | 0.0% | 0.0% | 0.0% |
|  | D\_0\_\_Bacteria;D\_1\_\_Proteobacteria;D\_2\_\_Gammaproteobacteria;Other;Other | 0.1% | 0.0% | 0.0% | 0.0% | 0.0% | 0.0% | 0.0% | 0.1% | 0.9% |
|  | D\_0\_\_Bacteria;D\_1\_\_Proteobacteria;D\_2\_\_JTB23;D\_3\_\_uncultured bacterium;D\_4\_\_uncultured bacterium | 0.0% | 0.0% | 0.0% | 0.0% | 0.0% | 0.0% | 0.0% | 0.0% | 0.0% |
|  | D\_0\_\_Bacteria;D\_1\_\_Proteobacteria;D\_2\_\_JTB23;Other;Other | 0.0% | 0.0% | 0.0% | 0.0% | 0.0% | 0.0% | 0.0% | 0.0% | 0.0% |
|  | D\_0\_\_Bacteria;D\_1\_\_Proteobacteria;D\_2\_\_SPOTSOCT00m83;Ambiguous\_taxa;Ambiguous\_taxa | 0.0% | 0.0% | 0.0% | 0.0% | 0.0% | 0.0% | 0.0% | 0.0% | 0.0% |
|  | D\_0\_\_Bacteria;D\_1\_\_Proteobacteria;D\_2\_\_SPOTSOCT00m83;Other;Other | 0.0% | 0.0% | 0.0% | 0.0% | 0.0% | 0.0% | 0.0% | 0.0% | 0.0% |
|  | D\_0\_\_Bacteria;D\_1\_\_Proteobacteria;Other;Other;Other | 0.0% | 0.0% | 0.0% | 0.0% | 0.0% | 0.0% | 0.0% | 0.0% | 0.0% |
|  | D\_0\_\_Bacteria;D\_1\_\_SBR1093;Other;Other;Other | 0.0% | 0.0% | 0.0% | 0.0% | 0.0% | 0.0% | 0.0% | 0.0% | 0.0% |
|  | D\_0\_\_Bacteria;D\_1\_\_SR1 (Absconditabacteria);Ambiguous\_taxa;Ambiguous\_taxa;Ambiguous\_taxa | 0.0% | 0.0% | 0.0% | 0.0% | 0.0% | 0.0% | 0.0% | 0.0% | 0.0% |
|  | D\_0\_\_Bacteria;D\_1\_\_SR1 (Absconditabacteria);D\_2\_\_uncultured bacterium;D\_3\_\_uncultured bacterium;D\_4\_\_uncultured bacterium | 0.0% | 0.0% | 0.0% | 0.0% | 0.0% | 0.0% | 0.0% | 0.0% | 0.1% |
|  | D\_0\_\_Bacteria;D\_1\_\_Saccharibacteria;D\_2\_\_uncultured bacterium;D\_3\_\_uncultured bacterium;D\_4\_\_uncultured bacterium | 0.0% | 0.0% | 0.0% | 0.0% | 0.0% | 0.0% | 0.0% | 0.0% | 0.0% |
|  | D\_0\_\_Bacteria;D\_1\_\_Saccharibacteria;Other;Other;Other | 0.0% | 0.0% | 0.0% | 0.0% | 0.0% | 0.0% | 0.0% | 0.0% | 0.0% |
|  | D\_0\_\_Bacteria;D\_1\_\_Spirochaetae;D\_2\_\_Spirochaetes;D\_3\_\_Spirochaetales;D\_4\_\_Spirochaetaceae | 0.0% | 0.0% | 0.0% | 0.0% | 0.0% | 0.0% | 0.0% | 0.0% | 0.1% |
|  | D\_0\_\_Bacteria;D\_1\_\_Synergistetes;D\_2\_\_Synergistia;D\_3\_\_Synergistales;D\_4\_\_Synergistaceae | 0.0% | 0.0% | 0.0% | 0.0% | 0.0% | 0.0% | 0.0% | 0.0% | 0.0% |
|  | D\_0\_\_Bacteria;D\_1\_\_TM6 (Dependentiae);Other;Other;Other | 0.0% | 0.0% | 0.0% | 0.0% | 0.0% | 0.0% | 0.0% | 0.0% | 0.0% |
|  | D\_0\_\_Bacteria;D\_1\_\_Tectomicrobia;D\_2\_\_Tectomicrobia Incertae Sedis;D\_3\_\_Unknown Order;D\_4\_\_Unknown Family | 0.0% | 0.0% | 0.0% | 0.0% | 0.0% | 0.0% | 0.0% | 0.0% | 0.0% |
|  | D\_0\_\_Bacteria;D\_1\_\_Tectomicrobia;D\_2\_\_uncultured bacterium;D\_3\_\_uncultured bacterium;D\_4\_\_uncultured bacterium | 0.0% | 0.0% | 0.0% | 0.0% | 0.0% | 0.0% | 0.0% | 0.0% | 0.0% |
|  | D\_0\_\_Bacteria;D\_1\_\_Verrucomicrobia;D\_2\_\_OPB35 soil group;Ambiguous\_taxa;Ambiguous\_taxa | 0.0% | 0.0% | 0.0% | 0.0% | 0.0% | 0.0% | 0.0% | 0.0% | 0.0% |
|  | D\_0\_\_Bacteria;D\_1\_\_Verrucomicrobia;D\_2\_\_OPB35 soil group;D\_3\_\_uncultured bacterium;D\_4\_\_uncultured bacterium | 0.0% | 0.0% | 0.0% | 0.0% | 0.0% | 0.0% | 0.0% | 0.0% | 0.0% |
|  | D\_0\_\_Bacteria;D\_1\_\_Verrucomicrobia;D\_2\_\_OPB35 soil group;Other;Other | 0.0% | 0.0% | 0.0% | 0.0% | 0.0% | 0.0% | 0.0% | 0.0% | 0.0% |
|  | D\_0\_\_Bacteria;D\_1\_\_Verrucomicrobia;D\_2\_\_Opitutae;D\_3\_\_BC-COM435;D\_4\_\_uncultured bacterium | 0.0% | 0.0% | 0.0% | 0.0% | 0.0% | 0.0% | 0.0% | 0.0% | 0.0% |
|  | D\_0\_\_Bacteria;D\_1\_\_Verrucomicrobia;D\_2\_\_Opitutae;D\_3\_\_Opitutales;D\_4\_\_Opitutaceae | 0.0% | 0.0% | 0.0% | 0.0% | 0.0% | 0.0% | 0.0% | 0.0% | 0.0% |
|  | D\_0\_\_Bacteria;D\_1\_\_Verrucomicrobia;D\_2\_\_Spartobacteria;D\_3\_\_Chthoniobacterales;D\_4\_\_Chthoniobacteraceae | 0.0% | 0.0% | 0.0% | 0.0% | 0.0% | 0.0% | 0.0% | 0.0% | 0.0% |
|  | D\_0\_\_Bacteria;D\_1\_\_Verrucomicrobia;D\_2\_\_Spartobacteria;D\_3\_\_Chthoniobacterales;D\_4\_\_DA101 soil group | 0.0% | 0.0% | 0.0% | 0.0% | 0.0% | 0.0% | 0.0% | 0.0% | 0.0% |
|  | D\_0\_\_Bacteria;D\_1\_\_Verrucomicrobia;D\_2\_\_Spartobacteria;D\_3\_\_Chthoniobacterales;D\_4\_\_Xiphinematobacteraceae | 0.0% | 0.0% | 0.0% | 0.0% | 0.0% | 0.0% | 0.0% | 0.0% | 0.0% |
|  | D\_0\_\_Bacteria;D\_1\_\_Verrucomicrobia;D\_2\_\_Spartobacteria;D\_3\_\_Chthoniobacterales;Other | 0.0% | 0.0% | 0.0% | 0.0% | 0.0% | 0.0% | 0.0% | 0.0% | 0.0% |
|  | D\_0\_\_Bacteria;D\_1\_\_Verrucomicrobia;D\_2\_\_Verrucomicrobiae;D\_3\_\_Verrucomicrobiales;D\_4\_\_DEV007 | 0.0% | 0.0% | 0.0% | 0.0% | 0.0% | 0.0% | 0.0% | 0.0% | 0.0% |
|  | D\_0\_\_Bacteria;D\_1\_\_Verrucomicrobia;D\_2\_\_Verrucomicrobiae;D\_3\_\_Verrucomicrobiales;D\_4\_\_Verrucomicrobiaceae | 0.0% | 0.0% | 0.0% | 0.0% | 0.0% | 0.0% | 0.0% | 0.0% | 0.0% |
|  | D\_0\_\_Bacteria;Other;Other;Other;Other | 0.0% | 0.0% | 0.0% | 0.0% | 0.0% | 0.0% | 0.0% | 0.0% | 0.0% |
|  | Unassigned;Other;Other;Other;Other | 0.3% | 0.4% | 0.4% | 0.3% | 0.3% | 0.4% | 0.1% | 0.2% | 0.2% |

|  |  |
| --- | --- |
|  | |
| Taxonomy Summary. Current Level: | |
| View Figure (.pdf)  View Legend (.pdf) |  |
|  |


|  |
| --- |
| View Table (.txt) |

|  |  |  |  |  |  |  |  |  |  |  |
| --- | --- | --- | --- | --- | --- | --- | --- | --- | --- | --- |
|  | | Total | 1B5g1 | 1B5g2 | 1BEDTA1 | 1BEDTA2 | 1M1 | 1M2 | 1S1 | 1S2 |
| Legend | Taxonomy | % | % | % | % | % | % | % | % | % |
|  | D\_0\_\_Archaea;D\_1\_\_Thaumarchaeota;D\_2\_\_Soil Crenarchaeotic Group(SCG);D\_3\_\_Unknown Order;D\_4\_\_Unknown Family;D\_5\_\_Candidatus Nitrososphaera | 0.0% | 0.0% | 0.0% | 0.0% | 0.0% | 0.0% | 0.0% | 0.0% | 0.0% |
|  | D\_0\_\_Archaea;D\_1\_\_Thaumarchaeota;D\_2\_\_Soil Crenarchaeotic Group(SCG);D\_3\_\_uncultured archaeon;D\_4\_\_uncultured archaeon;D\_5\_\_uncultured archaeon | 0.0% | 0.0% | 0.0% | 0.0% | 0.0% | 0.0% | 0.0% | 0.0% | 0.0% |
|  | D\_0\_\_Archaea;D\_1\_\_Thaumarchaeota;D\_2\_\_Soil Crenarchaeotic Group(SCG);D\_3\_\_uncultured euryarchaeote;D\_4\_\_uncultured euryarchaeote;D\_5\_\_uncultured euryarchaeote | 0.0% | 0.0% | 0.0% | 0.0% | 0.0% | 0.0% | 0.0% | 0.0% | 0.0% |
|  | D\_0\_\_Archaea;D\_1\_\_Thaumarchaeota;D\_2\_\_Soil Crenarchaeotic Group(SCG);Other;Other;Other | 0.6% | 0.7% | 0.7% | 0.4% | 0.5% | 1.0% | 0.8% | 0.2% | 0.3% |
|  | D\_0\_\_Bacteria;D\_1\_\_Acidobacteria;D\_2\_\_Blastocatellia;D\_3\_\_Blastocatellales;D\_4\_\_Blastocatellaceae (Subgroup 4);D\_5\_\_11-24 | 0.0% | 0.0% | 0.0% | 0.0% | 0.0% | 0.0% | 0.0% | 0.0% | 0.0% |
|  | D\_0\_\_Bacteria;D\_1\_\_Acidobacteria;D\_2\_\_Blastocatellia;D\_3\_\_Blastocatellales;D\_4\_\_Blastocatellaceae (Subgroup 4);D\_5\_\_Aridibacter | 0.0% | 0.0% | 0.0% | 0.0% | 0.0% | 0.0% | 0.0% | 0.0% | 0.0% |
|  | D\_0\_\_Bacteria;D\_1\_\_Acidobacteria;D\_2\_\_Blastocatellia;D\_3\_\_Blastocatellales;D\_4\_\_Blastocatellaceae (Subgroup 4);D\_5\_\_Blastocatella | 0.0% | 0.0% | 0.0% | 0.0% | 0.0% | 0.0% | 0.0% | 0.0% | 0.0% |
|  | D\_0\_\_Bacteria;D\_1\_\_Acidobacteria;D\_2\_\_Blastocatellia;D\_3\_\_Blastocatellales;D\_4\_\_Blastocatellaceae (Subgroup 4);D\_5\_\_DS-100 | 0.0% | 0.0% | 0.0% | 0.0% | 0.0% | 0.0% | 0.0% | 0.0% | 0.0% |
|  | D\_0\_\_Bacteria;D\_1\_\_Acidobacteria;D\_2\_\_Blastocatellia;D\_3\_\_Blastocatellales;D\_4\_\_Blastocatellaceae (Subgroup 4);D\_5\_\_RB41 | 0.0% | 0.0% | 0.0% | 0.0% | 0.0% | 0.0% | 0.0% | 0.0% | 0.0% |
|  | D\_0\_\_Bacteria;D\_1\_\_Acidobacteria;D\_2\_\_Blastocatellia;D\_3\_\_Blastocatellales;D\_4\_\_Blastocatellaceae (Subgroup 4);D\_5\_\_Stenotrophobacter | 0.0% | 0.0% | 0.0% | 0.0% | 0.0% | 0.0% | 0.0% | 0.0% | 0.0% |
|  | D\_0\_\_Bacteria;D\_1\_\_Acidobacteria;D\_2\_\_Blastocatellia;D\_3\_\_Blastocatellales;D\_4\_\_Blastocatellaceae (Subgroup 4);D\_5\_\_uncultured | 0.0% | 0.0% | 0.0% | 0.0% | 0.0% | 0.0% | 0.0% | 0.0% | 0.0% |
|  | D\_0\_\_Bacteria;D\_1\_\_Acidobacteria;D\_2\_\_Blastocatellia;D\_3\_\_Blastocatellales;D\_4\_\_Blastocatellaceae (Subgroup 4);Other | 0.0% | 0.0% | 0.0% | 0.0% | 0.0% | 0.0% | 0.0% | 0.0% | 0.0% |
|  | D\_0\_\_Bacteria;D\_1\_\_Acidobacteria;D\_2\_\_Holophagae;D\_3\_\_Holophagales;D\_4\_\_Holophagaceae;D\_5\_\_uncultured | 0.0% | 0.0% | 0.0% | 0.0% | 0.0% | 0.0% | 0.0% | 0.0% | 0.0% |
|  | D\_0\_\_Bacteria;D\_1\_\_Acidobacteria;D\_2\_\_Holophagae;D\_3\_\_Subgroup 10;D\_4\_\_CA002;D\_5\_\_uncultured Acidobacteria bacterium | 0.0% | 0.0% | 0.0% | 0.0% | 0.0% | 0.0% | 0.0% | 0.0% | 0.0% |
|  | D\_0\_\_Bacteria;D\_1\_\_Acidobacteria;D\_2\_\_Holophagae;D\_3\_\_Subgroup 10;D\_4\_\_CA002;D\_5\_\_uncultured bacterium | 0.0% | 0.0% | 0.0% | 0.0% | 0.0% | 0.0% | 0.0% | 0.0% | 0.0% |
|  | D\_0\_\_Bacteria;D\_1\_\_Acidobacteria;D\_2\_\_Holophagae;D\_3\_\_Subgroup 10;D\_4\_\_CA002;Other | 0.0% | 0.0% | 0.0% | 0.0% | 0.0% | 0.0% | 0.0% | 0.0% | 0.0% |
|  | D\_0\_\_Bacteria;D\_1\_\_Acidobacteria;D\_2\_\_Holophagae;D\_3\_\_Subgroup 10;D\_4\_\_NS72;D\_5\_\_uncultured bacterium | 0.0% | 0.0% | 0.0% | 0.0% | 0.0% | 0.1% | 0.0% | 0.0% | 0.0% |
|  | D\_0\_\_Bacteria;D\_1\_\_Acidobacteria;D\_2\_\_Holophagae;D\_3\_\_Subgroup 10;D\_4\_\_NS72;Other | 0.0% | 0.0% | 0.0% | 0.0% | 0.0% | 0.0% | 0.0% | 0.0% | 0.0% |
|  | D\_0\_\_Bacteria;D\_1\_\_Acidobacteria;D\_2\_\_Holophagae;D\_3\_\_Subgroup 10;D\_4\_\_Sva0725;D\_5\_\_uncultured bacterium | 0.0% | 0.0% | 0.0% | 0.0% | 0.0% | 0.0% | 0.0% | 0.0% | 0.0% |
|  | D\_0\_\_Bacteria;D\_1\_\_Acidobacteria;D\_2\_\_Holophagae;D\_3\_\_Subgroup 10;Other;Other | 0.0% | 0.0% | 0.0% | 0.0% | 0.0% | 0.0% | 0.0% | 0.0% | 0.0% |
|  | D\_0\_\_Bacteria;D\_1\_\_Acidobacteria;D\_2\_\_Holophagae;D\_3\_\_Subgroup 7;Ambiguous\_taxa;Ambiguous\_taxa | 0.0% | 0.0% | 0.0% | 0.0% | 0.0% | 0.0% | 0.0% | 0.0% | 0.0% |
|  | D\_0\_\_Bacteria;D\_1\_\_Acidobacteria;D\_2\_\_Holophagae;D\_3\_\_Subgroup 7;D\_4\_\_uncultured bacterium;D\_5\_\_uncultured bacterium | 0.0% | 0.0% | 0.0% | 0.0% | 0.0% | 0.0% | 0.0% | 0.0% | 0.0% |
|  | D\_0\_\_Bacteria;D\_1\_\_Acidobacteria;D\_2\_\_Holophagae;D\_3\_\_Subgroup 7;Other;Other | 0.0% | 0.0% | 0.0% | 0.0% | 0.0% | 0.0% | 0.0% | 0.0% | 0.0% |
|  | D\_0\_\_Bacteria;D\_1\_\_Acidobacteria;D\_2\_\_Solibacteres;D\_3\_\_Solibacterales;D\_4\_\_Solibacteraceae (Subgroup 3);D\_5\_\_Bryobacter | 0.0% | 0.0% | 0.0% | 0.0% | 0.0% | 0.0% | 0.0% | 0.0% | 0.0% |
|  | D\_0\_\_Bacteria;D\_1\_\_Acidobacteria;D\_2\_\_Solibacteres;D\_3\_\_Solibacterales;D\_4\_\_Solibacteraceae (Subgroup 3);D\_5\_\_Candidatus Solibacter | 0.0% | 0.0% | 0.0% | 0.0% | 0.0% | 0.0% | 0.0% | 0.0% | 0.0% |
|  | D\_0\_\_Bacteria;D\_1\_\_Acidobacteria;D\_2\_\_Solibacteres;D\_3\_\_Solibacterales;D\_4\_\_Solibacteraceae (Subgroup 3);D\_5\_\_PAUC26f | 0.0% | 0.0% | 0.0% | 0.0% | 0.0% | 0.0% | 0.0% | 0.0% | 0.0% |
|  | D\_0\_\_Bacteria;D\_1\_\_Acidobacteria;D\_2\_\_Subgroup 17;Ambiguous\_taxa;Ambiguous\_taxa;Ambiguous\_taxa | 0.0% | 0.0% | 0.0% | 0.0% | 0.0% | 0.0% | 0.0% | 0.0% | 0.0% |
|  | D\_0\_\_Bacteria;D\_1\_\_Acidobacteria;D\_2\_\_Subgroup 17;D\_3\_\_uncultured Acidobacteria bacterium;D\_4\_\_uncultured Acidobacteria bacterium;D\_5\_\_uncultured Acidobacteria bacterium | 0.0% | 0.0% | 0.0% | 0.0% | 0.0% | 0.0% | 0.0% | 0.0% | 0.0% |
|  | D\_0\_\_Bacteria;D\_1\_\_Acidobacteria;D\_2\_\_Subgroup 17;Other;Other;Other | 2.5% | 3.3% | 2.9% | 3.0% | 3.0% | 2.8% | 1.1% | 2.5% | 1.6% |
|  | D\_0\_\_Bacteria;D\_1\_\_Acidobacteria;D\_2\_\_Subgroup 22;Ambiguous\_taxa;Ambiguous\_taxa;Ambiguous\_taxa | 0.0% | 0.0% | 0.0% | 0.0% | 0.0% | 0.0% | 0.0% | 0.0% | 0.0% |
|  | D\_0\_\_Bacteria;D\_1\_\_Acidobacteria;D\_2\_\_Subgroup 22;D\_3\_\_uncultured Acidobacteria bacterium;D\_4\_\_uncultured Acidobacteria bacterium;D\_5\_\_uncultured Acidobacteria bacterium | 0.0% | 0.0% | 0.0% | 0.0% | 0.0% | 0.0% | 0.0% | 0.0% | 0.0% |
|  | D\_0\_\_Bacteria;D\_1\_\_Acidobacteria;D\_2\_\_Subgroup 22;D\_3\_\_uncultured bacterium;D\_4\_\_uncultured bacterium;D\_5\_\_uncultured bacterium | 0.0% | 0.0% | 0.0% | 0.0% | 0.0% | 0.0% | 0.0% | 0.0% | 0.0% |
|  | D\_0\_\_Bacteria;D\_1\_\_Acidobacteria;D\_2\_\_Subgroup 22;Other;Other;Other | 0.0% | 0.0% | 0.0% | 0.0% | 0.0% | 0.0% | 0.0% | 0.0% | 0.0% |
|  | D\_0\_\_Bacteria;D\_1\_\_Acidobacteria;D\_2\_\_Subgroup 5;D\_3\_\_uncultured bacterium;D\_4\_\_uncultured bacterium;D\_5\_\_uncultured bacterium | 0.0% | 0.0% | 0.0% | 0.0% | 0.0% | 0.0% | 0.0% | 0.0% | 0.0% |
|  | D\_0\_\_Bacteria;D\_1\_\_Acidobacteria;D\_2\_\_Subgroup 5;Other;Other;Other | 0.0% | 0.0% | 0.0% | 0.0% | 0.0% | 0.0% | 0.0% | 0.0% | 0.0% |
|  | D\_0\_\_Bacteria;D\_1\_\_Acidobacteria;D\_2\_\_Subgroup 6;Ambiguous\_taxa;Ambiguous\_taxa;Ambiguous\_taxa | 0.1% | 0.1% | 0.1% | 0.1% | 0.1% | 0.1% | 0.0% | 0.0% | 0.0% |
|  | D\_0\_\_Bacteria;D\_1\_\_Acidobacteria;D\_2\_\_Subgroup 6;D\_3\_\_uncultured Acidobacteria bacterium;D\_4\_\_uncultured Acidobacteria bacterium;D\_5\_\_uncultured Acidobacteria bacterium | 0.0% | 0.0% | 0.0% | 0.0% | 0.0% | 0.0% | 0.0% | 0.0% | 0.0% |
|  | D\_0\_\_Bacteria;D\_1\_\_Acidobacteria;D\_2\_\_Subgroup 6;D\_3\_\_uncultured bacterium;D\_4\_\_uncultured bacterium;D\_5\_\_uncultured bacterium | 0.2% | 0.5% | 0.3% | 0.3% | 0.3% | 0.0% | 0.0% | 0.0% | 0.0% |
|  | D\_0\_\_Bacteria;D\_1\_\_Acidobacteria;D\_2\_\_Subgroup 6;D\_3\_\_uncultured organism;D\_4\_\_uncultured organism;D\_5\_\_uncultured organism | 0.0% | 0.0% | 0.0% | 0.0% | 0.0% | 0.0% | 0.0% | 0.0% | 0.0% |
|  | D\_0\_\_Bacteria;D\_1\_\_Acidobacteria;D\_2\_\_Subgroup 6;D\_3\_\_uncultured soil bacterium;D\_4\_\_uncultured soil bacterium;D\_5\_\_uncultured soil bacterium | 0.0% | 0.0% | 0.0% | 0.0% | 0.0% | 0.0% | 0.0% | 0.0% | 0.0% |
|  | D\_0\_\_Bacteria;D\_1\_\_Acidobacteria;D\_2\_\_Subgroup 6;Other;Other;Other | 0.0% | 0.0% | 0.0% | 0.0% | 0.0% | 0.0% | 0.0% | 0.0% | 0.0% |
|  | D\_0\_\_Bacteria;D\_1\_\_Acidobacteria;Other;Other;Other;Other | 0.0% | 0.0% | 0.0% | 0.0% | 0.0% | 0.0% | 0.0% | 0.0% | 0.0% |
|  | D\_0\_\_Bacteria;D\_1\_\_Actinobacteria;D\_2\_\_Acidimicrobiia;D\_3\_\_Acidimicrobiales;D\_4\_\_Acidimicrobiaceae;D\_5\_\_CL500-29 marine group | 0.0% | 0.0% | 0.0% | 0.0% | 0.0% | 0.0% | 0.0% | 0.0% | 0.0% |
|  | D\_0\_\_Bacteria;D\_1\_\_Actinobacteria;D\_2\_\_Acidimicrobiia;D\_3\_\_Acidimicrobiales;D\_4\_\_Acidimicrobiaceae;D\_5\_\_Ilumatobacter | 0.0% | 0.0% | 0.0% | 0.0% | 0.0% | 0.0% | 0.0% | 0.0% | 0.0% |
|  | D\_0\_\_Bacteria;D\_1\_\_Actinobacteria;D\_2\_\_Acidimicrobiia;D\_3\_\_Acidimicrobiales;D\_4\_\_Acidimicrobiaceae;D\_5\_\_uncultured | 0.0% | 0.0% | 0.0% | 0.0% | 0.0% | 0.1% | 0.0% | 0.0% | 0.0% |
|  | D\_0\_\_Bacteria;D\_1\_\_Actinobacteria;D\_2\_\_Acidimicrobiia;D\_3\_\_Acidimicrobiales;D\_4\_\_Acidimicrobiaceae;Other | 0.0% | 0.0% | 0.0% | 0.0% | 0.0% | 0.0% | 0.0% | 0.0% | 0.0% |
|  | D\_0\_\_Bacteria;D\_1\_\_Actinobacteria;D\_2\_\_Acidimicrobiia;D\_3\_\_Acidimicrobiales;D\_4\_\_Acidimicrobiales Incertae Sedis;D\_5\_\_Aciditerrimonas | 0.0% | 0.0% | 0.0% | 0.0% | 0.0% | 0.0% | 0.0% | 0.0% | 0.0% |
|  | D\_0\_\_Bacteria;D\_1\_\_Actinobacteria;D\_2\_\_Acidimicrobiia;D\_3\_\_Acidimicrobiales;D\_4\_\_Iamiaceae;D\_5\_\_Iamia | 0.0% | 0.1% | 0.1% | 0.1% | 0.1% | 0.1% | 0.0% | 0.0% | 0.0% |
|  | D\_0\_\_Bacteria;D\_1\_\_Actinobacteria;D\_2\_\_Acidimicrobiia;D\_3\_\_Acidimicrobiales;D\_4\_\_OM1 clade;Ambiguous\_taxa | 0.0% | 0.0% | 0.0% | 0.0% | 0.0% | 0.0% | 0.0% | 0.0% | 0.0% |
|  | D\_0\_\_Bacteria;D\_1\_\_Actinobacteria;D\_2\_\_Acidimicrobiia;D\_3\_\_Acidimicrobiales;D\_4\_\_OM1 clade;D\_5\_\_uncultured actinobacterium | 0.0% | 0.0% | 0.0% | 0.0% | 0.0% | 0.0% | 0.0% | 0.0% | 0.0% |
|  | D\_0\_\_Bacteria;D\_1\_\_Actinobacteria;D\_2\_\_Acidimicrobiia;D\_3\_\_Acidimicrobiales;D\_4\_\_OM1 clade;D\_5\_\_uncultured bacterium | 2.2% | 4.2% | 4.4% | 2.7% | 3.2% | 1.7% | 0.2% | 0.7% | 0.6% |
|  | D\_0\_\_Bacteria;D\_1\_\_Actinobacteria;D\_2\_\_Acidimicrobiia;D\_3\_\_Acidimicrobiales;D\_4\_\_OM1 clade;D\_5\_\_uncultured compost bacterium | 0.0% | 0.0% | 0.0% | 0.0% | 0.0% | 0.0% | 0.0% | 0.0% | 0.0% |
|  | D\_0\_\_Bacteria;D\_1\_\_Actinobacteria;D\_2\_\_Acidimicrobiia;D\_3\_\_Acidimicrobiales;D\_4\_\_OM1 clade;Other | 0.0% | 0.0% | 0.0% | 0.0% | 0.0% | 0.0% | 0.0% | 0.0% | 0.0% |
|  | D\_0\_\_Bacteria;D\_1\_\_Actinobacteria;D\_2\_\_Acidimicrobiia;D\_3\_\_Acidimicrobiales;D\_4\_\_Sva0996 marine group;D\_5\_\_uncultured bacterium | 0.0% | 0.0% | 0.0% | 0.0% | 0.0% | 0.0% | 0.0% | 0.0% | 0.0% |
|  | D\_0\_\_Bacteria;D\_1\_\_Actinobacteria;D\_2\_\_Acidimicrobiia;D\_3\_\_Acidimicrobiales;D\_4\_\_Sva0996 marine group;Other | 0.0% | 0.0% | 0.0% | 0.0% | 0.0% | 0.0% | 0.0% | 0.0% | 0.0% |
|  | D\_0\_\_Bacteria;D\_1\_\_Actinobacteria;D\_2\_\_Acidimicrobiia;D\_3\_\_Acidimicrobiales;D\_4\_\_uncultured;Ambiguous\_taxa | 0.1% | 0.1% | 0.1% | 0.1% | 0.1% | 0.0% | 0.0% | 0.0% | 0.0% |
|  | D\_0\_\_Bacteria;D\_1\_\_Actinobacteria;D\_2\_\_Acidimicrobiia;D\_3\_\_Acidimicrobiales;D\_4\_\_uncultured;D\_5\_\_uncultured Acidimicrobidae bacterium | 0.0% | 0.0% | 0.0% | 0.0% | 0.0% | 0.0% | 0.0% | 0.0% | 0.0% |
|  | D\_0\_\_Bacteria;D\_1\_\_Actinobacteria;D\_2\_\_Acidimicrobiia;D\_3\_\_Acidimicrobiales;D\_4\_\_uncultured;D\_5\_\_uncultured Actinomycetales bacterium | 0.0% | 0.0% | 0.0% | 0.0% | 0.0% | 0.0% | 0.0% | 0.0% | 0.0% |
|  | D\_0\_\_Bacteria;D\_1\_\_Actinobacteria;D\_2\_\_Acidimicrobiia;D\_3\_\_Acidimicrobiales;D\_4\_\_uncultured;D\_5\_\_uncultured actinobacterium | 0.0% | 0.0% | 0.0% | 0.0% | 0.0% | 0.0% | 0.0% | 0.0% | 0.0% |
|  | D\_0\_\_Bacteria;D\_1\_\_Actinobacteria;D\_2\_\_Acidimicrobiia;D\_3\_\_Acidimicrobiales;D\_4\_\_uncultured;D\_5\_\_uncultured bacterium | 0.9% | 1.3% | 1.3% | 1.1% | 1.0% | 0.9% | 0.6% | 0.4% | 0.3% |
|  | D\_0\_\_Bacteria;D\_1\_\_Actinobacteria;D\_2\_\_Acidimicrobiia;D\_3\_\_Acidimicrobiales;D\_4\_\_uncultured;Other | 0.3% | 0.4% | 0.4% | 0.5% | 0.4% | 0.2% | 0.1% | 0.2% | 0.1% |
|  | D\_0\_\_Bacteria;D\_1\_\_Actinobacteria;D\_2\_\_Acidimicrobiia;D\_3\_\_Acidimicrobiales;Other;Other | 0.0% | 0.0% | 0.0% | 0.0% | 0.0% | 0.0% | 0.0% | 0.0% | 0.0% |
|  | D\_0\_\_Bacteria;D\_1\_\_Actinobacteria;D\_2\_\_Actinobacteria;D\_3\_\_Actinomycetales;D\_4\_\_Actinomycetaceae;D\_5\_\_Actinomyces | 0.0% | 0.0% | 0.0% | 0.0% | 0.0% | 0.0% | 0.0% | 0.0% | 0.3% |
|  | D\_0\_\_Bacteria;D\_1\_\_Actinobacteria;D\_2\_\_Actinobacteria;D\_3\_\_Actinomycetales;D\_4\_\_Actinomycetaceae;D\_5\_\_Actinotignum | 0.0% | 0.0% | 0.0% | 0.0% | 0.0% | 0.0% | 0.0% | 0.0% | 0.0% |
|  | D\_0\_\_Bacteria;D\_1\_\_Actinobacteria;D\_2\_\_Actinobacteria;D\_3\_\_Actinomycetales;D\_4\_\_Actinomycetaceae;D\_5\_\_Mobiluncus | 0.0% | 0.0% | 0.0% | 0.0% | 0.0% | 0.0% | 0.0% | 0.0% | 0.0% |
|  | D\_0\_\_Bacteria;D\_1\_\_Actinobacteria;D\_2\_\_Actinobacteria;D\_3\_\_Actinomycetales;D\_4\_\_Actinomycetaceae;D\_5\_\_Varibaculum | 0.0% | 0.0% | 0.0% | 0.0% | 0.0% | 0.0% | 0.0% | 0.0% | 0.0% |
|  | D\_0\_\_Bacteria;D\_1\_\_Actinobacteria;D\_2\_\_Actinobacteria;D\_3\_\_Actinomycetales;D\_4\_\_Actinomycetaceae;D\_5\_\_uncultured | 0.0% | 0.0% | 0.0% | 0.0% | 0.0% | 0.0% | 0.0% | 0.0% | 0.0% |
|  | D\_0\_\_Bacteria;D\_1\_\_Actinobacteria;D\_2\_\_Actinobacteria;D\_3\_\_Actinopolysporales;D\_4\_\_Actinopolysporaceae;D\_5\_\_Actinopolyspora | 0.0% | 0.0% | 0.0% | 0.0% | 0.0% | 0.0% | 0.0% | 0.0% | 0.0% |
|  | D\_0\_\_Bacteria;D\_1\_\_Actinobacteria;D\_2\_\_Actinobacteria;D\_3\_\_Bifidobacteriales;D\_4\_\_Bifidobacteriaceae;D\_5\_\_Bifidobacterium | 0.0% | 0.0% | 0.0% | 0.0% | 0.0% | 0.0% | 0.0% | 0.0% | 0.0% |
|  | D\_0\_\_Bacteria;D\_1\_\_Actinobacteria;D\_2\_\_Actinobacteria;D\_3\_\_Corynebacteriales;D\_4\_\_Corynebacteriaceae;D\_5\_\_Corynebacterium | 0.0% | 0.0% | 0.0% | 0.0% | 0.0% | 0.0% | 0.0% | 0.0% | 0.1% |
|  | D\_0\_\_Bacteria;D\_1\_\_Actinobacteria;D\_2\_\_Actinobacteria;D\_3\_\_Corynebacteriales;D\_4\_\_Corynebacteriaceae;D\_5\_\_Corynebacterium 1 | 0.0% | 0.0% | 0.0% | 0.0% | 0.0% | 0.0% | 0.0% | 0.0% | 0.2% |
|  | D\_0\_\_Bacteria;D\_1\_\_Actinobacteria;D\_2\_\_Actinobacteria;D\_3\_\_Corynebacteriales;D\_4\_\_Corynebacteriaceae;D\_5\_\_Lawsonella | 0.0% | 0.0% | 0.0% | 0.0% | 0.0% | 0.0% | 0.0% | 0.0% | 0.3% |
|  | D\_0\_\_Bacteria;D\_1\_\_Actinobacteria;D\_2\_\_Actinobacteria;D\_3\_\_Corynebacteriales;D\_4\_\_Dietziaceae;D\_5\_\_Dietzia | 0.0% | 0.0% | 0.0% | 0.0% | 0.0% | 0.0% | 0.0% | 0.0% | 0.0% |
|  | D\_0\_\_Bacteria;D\_1\_\_Actinobacteria;D\_2\_\_Actinobacteria;D\_3\_\_Corynebacteriales;D\_4\_\_Mycobacteriaceae;D\_5\_\_Mycobacterium | 0.0% | 0.0% | 0.0% | 0.0% | 0.0% | 0.0% | 0.0% | 0.0% | 0.0% |
|  | D\_0\_\_Bacteria;D\_1\_\_Actinobacteria;D\_2\_\_Actinobacteria;D\_3\_\_Corynebacteriales;D\_4\_\_Nocardiaceae;D\_5\_\_Nocardia | 0.0% | 0.0% | 0.0% | 0.0% | 0.0% | 0.0% | 0.0% | 0.0% | 0.0% |
|  | D\_0\_\_Bacteria;D\_1\_\_Actinobacteria;D\_2\_\_Actinobacteria;D\_3\_\_Corynebacteriales;D\_4\_\_Nocardiaceae;D\_5\_\_Rhodococcus | 0.0% | 0.0% | 0.0% | 0.0% | 0.0% | 0.0% | 0.0% | 0.0% | 0.0% |
|  | D\_0\_\_Bacteria;D\_1\_\_Actinobacteria;D\_2\_\_Actinobacteria;D\_3\_\_Corynebacteriales;Other;Other | 0.0% | 0.0% | 0.0% | 0.0% | 0.0% | 0.0% | 0.0% | 0.0% | 0.0% |
|  | D\_0\_\_Bacteria;D\_1\_\_Actinobacteria;D\_2\_\_Actinobacteria;D\_3\_\_Elev-16S-976;D\_4\_\_uncultured Nakamurellaceae bacterium;D\_5\_\_uncultured Nakamurellaceae bacterium | 0.0% | 0.0% | 0.0% | 0.0% | 0.0% | 0.0% | 0.0% | 0.0% | 0.0% |
|  | D\_0\_\_Bacteria;D\_1\_\_Actinobacteria;D\_2\_\_Actinobacteria;D\_3\_\_Frankiales;D\_4\_\_Acidothermaceae;D\_5\_\_Acidothermus | 0.5% | 0.0% | 0.0% | 0.1% | 0.1% | 0.6% | 0.4% | 1.3% | 1.3% |
|  | D\_0\_\_Bacteria;D\_1\_\_Actinobacteria;D\_2\_\_Actinobacteria;D\_3\_\_Frankiales;D\_4\_\_Cryptosporangiaceae;D\_5\_\_Fodinicola | 0.0% | 0.0% | 0.0% | 0.0% | 0.0% | 0.0% | 0.0% | 0.0% | 0.0% |
|  | D\_0\_\_Bacteria;D\_1\_\_Actinobacteria;D\_2\_\_Actinobacteria;D\_3\_\_Frankiales;D\_4\_\_Frankiaceae;D\_5\_\_Frankia | 13.2% | 11.1% | 11.7% | 10.5% | 11.1% | 7.7% | 6.7% | 25.4% | 21.6% |
|  | D\_0\_\_Bacteria;D\_1\_\_Actinobacteria;D\_2\_\_Actinobacteria;D\_3\_\_Frankiales;D\_4\_\_Frankiaceae;D\_5\_\_Jatrophihabitans | 0.1% | 0.1% | 0.1% | 0.1% | 0.1% | 0.0% | 0.0% | 0.0% | 0.0% |
|  | D\_0\_\_Bacteria;D\_1\_\_Actinobacteria;D\_2\_\_Actinobacteria;D\_3\_\_Frankiales;D\_4\_\_Frankiaceae;Other | 0.0% | 0.0% | 0.0% | 0.0% | 0.0% | 0.0% | 0.0% | 0.0% | 0.0% |
|  | D\_0\_\_Bacteria;D\_1\_\_Actinobacteria;D\_2\_\_Actinobacteria;D\_3\_\_Frankiales;D\_4\_\_Geodermatophilaceae;Ambiguous\_taxa | 0.0% | 0.0% | 0.0% | 0.0% | 0.0% | 0.0% | 0.0% | 0.0% | 0.0% |
|  | D\_0\_\_Bacteria;D\_1\_\_Actinobacteria;D\_2\_\_Actinobacteria;D\_3\_\_Frankiales;D\_4\_\_Geodermatophilaceae;D\_5\_\_Blastococcus | 0.0% | 0.0% | 0.0% | 0.0% | 0.0% | 0.0% | 0.0% | 0.0% | 0.0% |
|  | D\_0\_\_Bacteria;D\_1\_\_Actinobacteria;D\_2\_\_Actinobacteria;D\_3\_\_Frankiales;D\_4\_\_Geodermatophilaceae;D\_5\_\_Geodermatophilus | 0.0% | 0.0% | 0.0% | 0.0% | 0.0% | 0.0% | 0.0% | 0.0% | 0.0% |
|  | D\_0\_\_Bacteria;D\_1\_\_Actinobacteria;D\_2\_\_Actinobacteria;D\_3\_\_Frankiales;D\_4\_\_Geodermatophilaceae;D\_5\_\_Modestobacter | 0.0% | 0.0% | 0.0% | 0.0% | 0.0% | 0.0% | 0.0% | 0.0% | 0.0% |
|  | D\_0\_\_Bacteria;D\_1\_\_Actinobacteria;D\_2\_\_Actinobacteria;D\_3\_\_Frankiales;D\_4\_\_Geodermatophilaceae;D\_5\_\_uncultured | 0.0% | 0.0% | 0.0% | 0.0% | 0.0% | 0.0% | 0.0% | 0.0% | 0.0% |
|  | D\_0\_\_Bacteria;D\_1\_\_Actinobacteria;D\_2\_\_Actinobacteria;D\_3\_\_Frankiales;D\_4\_\_Geodermatophilaceae;Other | 0.0% | 0.0% | 0.0% | 0.0% | 0.0% | 0.0% | 0.0% | 0.0% | 0.0% |
|  | D\_0\_\_Bacteria;D\_1\_\_Actinobacteria;D\_2\_\_Actinobacteria;D\_3\_\_Frankiales;D\_4\_\_Nakamurellaceae;D\_5\_\_Nakamurella | 0.0% | 0.0% | 0.0% | 0.0% | 0.0% | 0.1% | 0.0% | 0.1% | 0.0% |
|  | D\_0\_\_Bacteria;D\_1\_\_Actinobacteria;D\_2\_\_Actinobacteria;D\_3\_\_Frankiales;D\_4\_\_Sporichthyaceae;D\_5\_\_Sporichthya | 0.0% | 0.0% | 0.0% | 0.0% | 0.0% | 0.0% | 0.0% | 0.0% | 0.0% |
|  | D\_0\_\_Bacteria;D\_1\_\_Actinobacteria;D\_2\_\_Actinobacteria;D\_3\_\_Frankiales;D\_4\_\_Sporichthyaceae;D\_5\_\_uncultured | 0.0% | 0.0% | 0.0% | 0.0% | 0.0% | 0.0% | 0.0% | 0.0% | 0.0% |
|  | D\_0\_\_Bacteria;D\_1\_\_Actinobacteria;D\_2\_\_Actinobacteria;D\_3\_\_Frankiales;D\_4\_\_Sporichthyaceae;Other | 0.0% | 0.0% | 0.0% | 0.0% | 0.0% | 0.0% | 0.0% | 0.0% | 0.0% |
|  | D\_0\_\_Bacteria;D\_1\_\_Actinobacteria;D\_2\_\_Actinobacteria;D\_3\_\_Frankiales;D\_4\_\_uncultured;Ambiguous\_taxa | 0.1% | 0.0% | 0.0% | 0.1% | 0.1% | 0.1% | 0.1% | 0.1% | 0.0% |
|  | D\_0\_\_Bacteria;D\_1\_\_Actinobacteria;D\_2\_\_Actinobacteria;D\_3\_\_Frankiales;D\_4\_\_uncultured;D\_5\_\_uncultured Sporichthyaceae bacterium | 0.0% | 0.0% | 0.0% | 0.0% | 0.0% | 0.0% | 0.0% | 0.0% | 0.0% |
|  | D\_0\_\_Bacteria;D\_1\_\_Actinobacteria;D\_2\_\_Actinobacteria;D\_3\_\_Frankiales;D\_4\_\_uncultured;D\_5\_\_uncultured actinobacterium | 0.0% | 0.0% | 0.0% | 0.0% | 0.0% | 0.0% | 0.0% | 0.0% | 0.0% |
|  | D\_0\_\_Bacteria;D\_1\_\_Actinobacteria;D\_2\_\_Actinobacteria;D\_3\_\_Frankiales;D\_4\_\_uncultured;D\_5\_\_uncultured bacterium | 0.0% | 0.0% | 0.0% | 0.0% | 0.0% | 0.0% | 0.0% | 0.0% | 0.0% |
|  | D\_0\_\_Bacteria;D\_1\_\_Actinobacteria;D\_2\_\_Actinobacteria;D\_3\_\_Frankiales;D\_4\_\_uncultured;Other | 0.0% | 0.0% | 0.0% | 0.0% | 0.0% | 0.0% | 0.0% | 0.0% | 0.0% |
|  | D\_0\_\_Bacteria;D\_1\_\_Actinobacteria;D\_2\_\_Actinobacteria;D\_3\_\_Frankiales;Other;Other | 0.0% | 0.0% | 0.0% | 0.0% | 0.0% | 0.1% | 0.1% | 0.0% | 0.0% |
|  | D\_0\_\_Bacteria;D\_1\_\_Actinobacteria;D\_2\_\_Actinobacteria;D\_3\_\_Glycomycetales;D\_4\_\_Glycomycetaceae;D\_5\_\_Glycomyces | 0.0% | 0.0% | 0.0% | 0.0% | 0.0% | 0.0% | 0.0% | 0.0% | 0.0% |
|  | D\_0\_\_Bacteria;D\_1\_\_Actinobacteria;D\_2\_\_Actinobacteria;D\_3\_\_Kineosporiales;D\_4\_\_Kineosporiaceae;D\_5\_\_Angustibacter | 0.0% | 0.0% | 0.0% | 0.0% | 0.0% | 0.0% | 0.0% | 0.0% | 0.0% |
|  | D\_0\_\_Bacteria;D\_1\_\_Actinobacteria;D\_2\_\_Actinobacteria;D\_3\_\_Kineosporiales;D\_4\_\_Kineosporiaceae;D\_5\_\_Kineococcus | 0.0% | 0.0% | 0.0% | 0.0% | 0.0% | 0.0% | 0.0% | 0.0% | 0.0% |
|  | D\_0\_\_Bacteria;D\_1\_\_Actinobacteria;D\_2\_\_Actinobacteria;D\_3\_\_Kineosporiales;D\_4\_\_Kineosporiaceae;D\_5\_\_Quadrisphaera | 0.0% | 0.0% | 0.0% | 0.0% | 0.0% | 0.0% | 0.0% | 0.0% | 0.0% |
|  | D\_0\_\_Bacteria;D\_1\_\_Actinobacteria;D\_2\_\_Actinobacteria;D\_3\_\_Kineosporiales;D\_4\_\_Kineosporiaceae;D\_5\_\_uncultured | 0.0% | 0.0% | 0.0% | 0.0% | 0.0% | 0.0% | 0.0% | 0.0% | 0.0% |
|  | D\_0\_\_Bacteria;D\_1\_\_Actinobacteria;D\_2\_\_Actinobacteria;D\_3\_\_Kineosporiales;D\_4\_\_Kineosporiaceae;Other | 0.0% | 0.0% | 0.0% | 0.0% | 0.0% | 0.0% | 0.0% | 0.0% | 0.0% |
|  | D\_0\_\_Bacteria;D\_1\_\_Actinobacteria;D\_2\_\_Actinobacteria;D\_3\_\_Micrococcales;D\_4\_\_Beutenbergiaceae;Ambiguous\_taxa | 0.0% | 0.0% | 0.0% | 0.0% | 0.0% | 0.0% | 0.0% | 0.0% | 0.0% |
|  | D\_0\_\_Bacteria;D\_1\_\_Actinobacteria;D\_2\_\_Actinobacteria;D\_3\_\_Micrococcales;D\_4\_\_Bogoriellaceae;D\_5\_\_Georgenia | 0.0% | 0.0% | 0.0% | 0.0% | 0.0% | 0.0% | 0.0% | 0.0% | 0.0% |
|  | D\_0\_\_Bacteria;D\_1\_\_Actinobacteria;D\_2\_\_Actinobacteria;D\_3\_\_Micrococcales;D\_4\_\_Bogoriellaceae;Other | 0.0% | 0.0% | 0.0% | 0.0% | 0.0% | 0.0% | 0.0% | 0.0% | 0.0% |
|  | D\_0\_\_Bacteria;D\_1\_\_Actinobacteria;D\_2\_\_Actinobacteria;D\_3\_\_Micrococcales;D\_4\_\_Cellulomonadaceae;Ambiguous\_taxa | 0.0% | 0.0% | 0.0% | 0.0% | 0.0% | 0.0% | 0.0% | 0.0% | 0.0% |
|  | D\_0\_\_Bacteria;D\_1\_\_Actinobacteria;D\_2\_\_Actinobacteria;D\_3\_\_Micrococcales;D\_4\_\_Cellulomonadaceae;D\_5\_\_Actinotalea | 0.0% | 0.0% | 0.0% | 0.0% | 0.0% | 0.0% | 0.0% | 0.0% | 0.0% |
|  | D\_0\_\_Bacteria;D\_1\_\_Actinobacteria;D\_2\_\_Actinobacteria;D\_3\_\_Micrococcales;D\_4\_\_Cellulomonadaceae;D\_5\_\_Cellulomonas | 0.0% | 0.0% | 0.0% | 0.0% | 0.0% | 0.0% | 0.0% | 0.0% | 0.0% |
|  | D\_0\_\_Bacteria;D\_1\_\_Actinobacteria;D\_2\_\_Actinobacteria;D\_3\_\_Micrococcales;D\_4\_\_Cellulomonadaceae;Other | 0.6% | 0.0% | 0.0% | 0.0% | 0.0% | 1.6% | 2.9% | 0.0% | 0.0% |
|  | D\_0\_\_Bacteria;D\_1\_\_Actinobacteria;D\_2\_\_Actinobacteria;D\_3\_\_Micrococcales;D\_4\_\_Demequinaceae;Ambiguous\_taxa | 0.0% | 0.0% | 0.0% | 0.0% | 0.0% | 0.0% | 0.0% | 0.0% | 0.0% |
|  | D\_0\_\_Bacteria;D\_1\_\_Actinobacteria;D\_2\_\_Actinobacteria;D\_3\_\_Micrococcales;D\_4\_\_Demequinaceae;Other | 0.0% | 0.0% | 0.0% | 0.0% | 0.0% | 0.0% | 0.0% | 0.0% | 0.0% |
|  | D\_0\_\_Bacteria;D\_1\_\_Actinobacteria;D\_2\_\_Actinobacteria;D\_3\_\_Micrococcales;D\_4\_\_Dermabacteraceae;D\_5\_\_Brachybacterium | 0.0% | 0.0% | 0.0% | 0.0% | 0.0% | 0.0% | 0.0% | 0.0% | 0.0% |
|  | D\_0\_\_Bacteria;D\_1\_\_Actinobacteria;D\_2\_\_Actinobacteria;D\_3\_\_Micrococcales;D\_4\_\_Dermabacteraceae;D\_5\_\_Dermabacter | 0.0% | 0.0% | 0.0% | 0.0% | 0.0% | 0.0% | 0.0% | 0.0% | 0.0% |
|  | D\_0\_\_Bacteria;D\_1\_\_Actinobacteria;D\_2\_\_Actinobacteria;D\_3\_\_Micrococcales;D\_4\_\_Dermacoccaceae;Other | 0.0% | 0.0% | 0.0% | 0.0% | 0.0% | 0.0% | 0.0% | 0.0% | 0.0% |
|  | D\_0\_\_Bacteria;D\_1\_\_Actinobacteria;D\_2\_\_Actinobacteria;D\_3\_\_Micrococcales;D\_4\_\_Dermatophilaceae;D\_5\_\_Dermatophilus | 0.0% | 0.0% | 0.0% | 0.0% | 0.0% | 0.0% | 0.0% | 0.0% | 0.0% |
|  | D\_0\_\_Bacteria;D\_1\_\_Actinobacteria;D\_2\_\_Actinobacteria;D\_3\_\_Micrococcales;D\_4\_\_Intrasporangiaceae;Ambiguous\_taxa | 0.0% | 0.0% | 0.0% | 0.0% | 0.0% | 0.0% | 0.0% | 0.0% | 0.0% |
|  | D\_0\_\_Bacteria;D\_1\_\_Actinobacteria;D\_2\_\_Actinobacteria;D\_3\_\_Micrococcales;D\_4\_\_Intrasporangiaceae;D\_5\_\_Knoellia | 0.0% | 0.0% | 0.0% | 0.0% | 0.0% | 0.0% | 0.0% | 0.0% | 0.0% |
|  | D\_0\_\_Bacteria;D\_1\_\_Actinobacteria;D\_2\_\_Actinobacteria;D\_3\_\_Micrococcales;D\_4\_\_Intrasporangiaceae;D\_5\_\_Lapillicoccus | 0.0% | 0.0% | 0.0% | 0.0% | 0.0% | 0.0% | 0.0% | 0.0% | 0.0% |
|  | D\_0\_\_Bacteria;D\_1\_\_Actinobacteria;D\_2\_\_Actinobacteria;D\_3\_\_Micrococcales;D\_4\_\_Intrasporangiaceae;D\_5\_\_Ornithinicoccus | 0.0% | 0.0% | 0.0% | 0.0% | 0.0% | 0.0% | 0.0% | 0.0% | 0.0% |
|  | D\_0\_\_Bacteria;D\_1\_\_Actinobacteria;D\_2\_\_Actinobacteria;D\_3\_\_Micrococcales;D\_4\_\_Intrasporangiaceae;D\_5\_\_Ornithinimicrobium | 0.0% | 0.0% | 0.0% | 0.0% | 0.0% | 0.0% | 0.0% | 0.1% | 0.1% |
|  | D\_0\_\_Bacteria;D\_1\_\_Actinobacteria;D\_2\_\_Actinobacteria;D\_3\_\_Micrococcales;D\_4\_\_Intrasporangiaceae;D\_5\_\_Oryzihumus | 0.0% | 0.0% | 0.0% | 0.0% | 0.0% | 0.0% | 0.0% | 0.0% | 0.0% |
|  | D\_0\_\_Bacteria;D\_1\_\_Actinobacteria;D\_2\_\_Actinobacteria;D\_3\_\_Micrococcales;D\_4\_\_Intrasporangiaceae;D\_5\_\_Terrabacter | 0.0% | 0.0% | 0.0% | 0.0% | 0.0% | 0.0% | 0.0% | 0.0% | 0.0% |
|  | D\_0\_\_Bacteria;D\_1\_\_Actinobacteria;D\_2\_\_Actinobacteria;D\_3\_\_Micrococcales;D\_4\_\_Intrasporangiaceae;D\_5\_\_Tetrasphaera | 0.0% | 0.0% | 0.0% | 0.0% | 0.0% | 0.0% | 0.0% | 0.0% | 0.0% |
|  | D\_0\_\_Bacteria;D\_1\_\_Actinobacteria;D\_2\_\_Actinobacteria;D\_3\_\_Micrococcales;D\_4\_\_Intrasporangiaceae;Other | 0.0% | 0.0% | 0.0% | 0.0% | 0.0% | 0.0% | 0.0% | 0.0% | 0.0% |
|  | D\_0\_\_Bacteria;D\_1\_\_Actinobacteria;D\_2\_\_Actinobacteria;D\_3\_\_Micrococcales;D\_4\_\_Microbacteriaceae;Ambiguous\_taxa | 0.0% | 0.0% | 0.0% | 0.0% | 0.0% | 0.0% | 0.0% | 0.0% | 0.0% |
|  | D\_0\_\_Bacteria;D\_1\_\_Actinobacteria;D\_2\_\_Actinobacteria;D\_3\_\_Micrococcales;D\_4\_\_Microbacteriaceae;D\_5\_\_Agrococcus | 0.0% | 0.0% | 0.0% | 0.0% | 0.0% | 0.0% | 0.0% | 0.0% | 0.0% |
|  | D\_0\_\_Bacteria;D\_1\_\_Actinobacteria;D\_2\_\_Actinobacteria;D\_3\_\_Micrococcales;D\_4\_\_Microbacteriaceae;D\_5\_\_Agromyces | 0.0% | 0.0% | 0.0% | 0.0% | 0.0% | 0.0% | 0.0% | 0.0% | 0.0% |
|  | D\_0\_\_Bacteria;D\_1\_\_Actinobacteria;D\_2\_\_Actinobacteria;D\_3\_\_Micrococcales;D\_4\_\_Microbacteriaceae;D\_5\_\_Clavibacter | 0.0% | 0.0% | 0.0% | 0.0% | 0.0% | 0.0% | 0.0% | 0.0% | 0.0% |
|  | D\_0\_\_Bacteria;D\_1\_\_Actinobacteria;D\_2\_\_Actinobacteria;D\_3\_\_Micrococcales;D\_4\_\_Microbacteriaceae;D\_5\_\_Curtobacterium | 0.0% | 0.0% | 0.0% | 0.0% | 0.0% | 0.0% | 0.0% | 0.0% | 0.0% |
|  | D\_0\_\_Bacteria;D\_1\_\_Actinobacteria;D\_2\_\_Actinobacteria;D\_3\_\_Micrococcales;D\_4\_\_Microbacteriaceae;D\_5\_\_Frigoribacterium | 0.0% | 0.0% | 0.0% | 0.0% | 0.0% | 0.0% | 0.0% | 0.0% | 0.0% |
|  | D\_0\_\_Bacteria;D\_1\_\_Actinobacteria;D\_2\_\_Actinobacteria;D\_3\_\_Micrococcales;D\_4\_\_Microbacteriaceae;D\_5\_\_Leifsonia | 0.0% | 0.0% | 0.0% | 0.0% | 0.0% | 0.0% | 0.0% | 0.0% | 0.0% |
|  | D\_0\_\_Bacteria;D\_1\_\_Actinobacteria;D\_2\_\_Actinobacteria;D\_3\_\_Micrococcales;D\_4\_\_Microbacteriaceae;D\_5\_\_Leucobacter | 0.0% | 0.0% | 0.0% | 0.0% | 0.0% | 0.0% | 0.0% | 0.0% | 0.0% |
|  | D\_0\_\_Bacteria;D\_1\_\_Actinobacteria;D\_2\_\_Actinobacteria;D\_3\_\_Micrococcales;D\_4\_\_Microbacteriaceae;D\_5\_\_Microbacterium | 0.0% | 0.0% | 0.0% | 0.0% | 0.0% | 0.0% | 0.0% | 0.0% | 0.0% |
|  | D\_0\_\_Bacteria;D\_1\_\_Actinobacteria;D\_2\_\_Actinobacteria;D\_3\_\_Micrococcales;D\_4\_\_Microbacteriaceae;D\_5\_\_Pseudoclavibacter | 0.0% | 0.0% | 0.0% | 0.0% | 0.0% | 0.0% | 0.0% | 0.0% | 0.0% |
|  | D\_0\_\_Bacteria;D\_1\_\_Actinobacteria;D\_2\_\_Actinobacteria;D\_3\_\_Micrococcales;D\_4\_\_Microbacteriaceae;Other | 0.0% | 0.0% | 0.0% | 0.0% | 0.0% | 0.0% | 0.0% | 0.0% | 0.0% |
|  | D\_0\_\_Bacteria;D\_1\_\_Actinobacteria;D\_2\_\_Actinobacteria;D\_3\_\_Micrococcales;D\_4\_\_Micrococcaceae;Ambiguous\_taxa | 0.0% | 0.0% | 0.0% | 0.0% | 0.0% | 0.0% | 0.0% | 0.0% | 0.0% |
|  | D\_0\_\_Bacteria;D\_1\_\_Actinobacteria;D\_2\_\_Actinobacteria;D\_3\_\_Micrococcales;D\_4\_\_Micrococcaceae;D\_5\_\_Arthrobacter | 1.0% | 0.4% | 0.5% | 0.6% | 0.6% | 0.1% | 0.1% | 3.3% | 2.2% |
|  | D\_0\_\_Bacteria;D\_1\_\_Actinobacteria;D\_2\_\_Actinobacteria;D\_3\_\_Micrococcales;D\_4\_\_Micrococcaceae;D\_5\_\_Kocuria | 0.0% | 0.0% | 0.0% | 0.0% | 0.0% | 0.0% | 0.0% | 0.1% | 0.0% |
|  | D\_0\_\_Bacteria;D\_1\_\_Actinobacteria;D\_2\_\_Actinobacteria;D\_3\_\_Micrococcales;D\_4\_\_Micrococcaceae;D\_5\_\_Micrococcus | 0.0% | 0.0% | 0.0% | 0.0% | 0.0% | 0.0% | 0.0% | 0.0% | 0.0% |
|  | D\_0\_\_Bacteria;D\_1\_\_Actinobacteria;D\_2\_\_Actinobacteria;D\_3\_\_Micrococcales;D\_4\_\_Micrococcaceae;D\_5\_\_Paeniglutamicibacter | 0.0% | 0.0% | 0.0% | 0.0% | 0.0% | 0.0% | 0.0% | 0.0% | 0.0% |
|  | D\_0\_\_Bacteria;D\_1\_\_Actinobacteria;D\_2\_\_Actinobacteria;D\_3\_\_Micrococcales;D\_4\_\_Micrococcaceae;D\_5\_\_Pseudarthrobacter | 0.0% | 0.0% | 0.0% | 0.0% | 0.0% | 0.0% | 0.0% | 0.0% | 0.0% |
|  | D\_0\_\_Bacteria;D\_1\_\_Actinobacteria;D\_2\_\_Actinobacteria;D\_3\_\_Micrococcales;D\_4\_\_Micrococcaceae;D\_5\_\_Rothia | 0.0% | 0.0% | 0.0% | 0.0% | 0.0% | 0.0% | 0.0% | 0.0% | 0.1% |
|  | D\_0\_\_Bacteria;D\_1\_\_Actinobacteria;D\_2\_\_Actinobacteria;D\_3\_\_Micrococcales;D\_4\_\_Micrococcaceae;D\_5\_\_Zhihengliuella | 0.0% | 0.0% | 0.0% | 0.0% | 0.0% | 0.0% | 0.0% | 0.0% | 0.0% |
|  | D\_0\_\_Bacteria;D\_1\_\_Actinobacteria;D\_2\_\_Actinobacteria;D\_3\_\_Micrococcales;D\_4\_\_Micrococcaceae;Other | 0.0% | 0.0% | 0.0% | 0.0% | 0.0% | 0.0% | 0.0% | 0.0% | 0.0% |
|  | D\_0\_\_Bacteria;D\_1\_\_Actinobacteria;D\_2\_\_Actinobacteria;D\_3\_\_Micrococcales;D\_4\_\_Promicromonosporaceae;D\_5\_\_Cellulosimicrobium | 0.0% | 0.0% | 0.0% | 0.0% | 0.0% | 0.0% | 0.0% | 0.0% | 0.0% |
|  | D\_0\_\_Bacteria;D\_1\_\_Actinobacteria;D\_2\_\_Actinobacteria;D\_3\_\_Micrococcales;D\_4\_\_Promicromonosporaceae;D\_5\_\_Isoptericola | 0.0% | 0.0% | 0.0% | 0.0% | 0.0% | 0.0% | 0.0% | 0.1% | 0.0% |
|  | D\_0\_\_Bacteria;D\_1\_\_Actinobacteria;D\_2\_\_Actinobacteria;D\_3\_\_Micrococcales;D\_4\_\_Promicromonosporaceae;D\_5\_\_Myceligenerans | 0.0% | 0.0% | 0.0% | 0.0% | 0.0% | 0.0% | 0.0% | 0.0% | 0.0% |
|  | D\_0\_\_Bacteria;D\_1\_\_Actinobacteria;D\_2\_\_Actinobacteria;D\_3\_\_Micrococcales;D\_4\_\_Promicromonosporaceae;D\_5\_\_Promicromonospora | 0.0% | 0.0% | 0.0% | 0.0% | 0.0% | 0.0% | 0.0% | 0.0% | 0.0% |
|  | D\_0\_\_Bacteria;D\_1\_\_Actinobacteria;D\_2\_\_Actinobacteria;D\_3\_\_Micrococcales;D\_4\_\_Promicromonosporaceae;D\_5\_\_Xylanimonas | 0.0% | 0.0% | 0.0% | 0.0% | 0.0% | 0.0% | 0.0% | 0.0% | 0.0% |
|  | D\_0\_\_Bacteria;D\_1\_\_Actinobacteria;D\_2\_\_Actinobacteria;D\_3\_\_Micrococcales;D\_4\_\_Promicromonosporaceae;Other | 0.0% | 0.0% | 0.0% | 0.0% | 0.0% | 0.0% | 0.0% | 0.0% | 0.0% |
|  | D\_0\_\_Bacteria;D\_1\_\_Actinobacteria;D\_2\_\_Actinobacteria;D\_3\_\_Micrococcales;Other;Other | 0.0% | 0.0% | 0.0% | 0.0% | 0.0% | 0.0% | 0.0% | 0.0% | 0.0% |
|  | D\_0\_\_Bacteria;D\_1\_\_Actinobacteria;D\_2\_\_Actinobacteria;D\_3\_\_Micromonosporales;D\_4\_\_Micromonosporaceae;Ambiguous\_taxa | 0.0% | 0.0% | 0.0% | 0.0% | 0.0% | 0.0% | 0.0% | 0.0% | 0.0% |
|  | D\_0\_\_Bacteria;D\_1\_\_Actinobacteria;D\_2\_\_Actinobacteria;D\_3\_\_Micromonosporales;D\_4\_\_Micromonosporaceae;D\_5\_\_Actinocatenispora | 0.0% | 0.0% | 0.0% | 0.0% | 0.0% | 0.0% | 0.0% | 0.0% | 0.0% |
|  | D\_0\_\_Bacteria;D\_1\_\_Actinobacteria;D\_2\_\_Actinobacteria;D\_3\_\_Micromonosporales;D\_4\_\_Micromonosporaceae;D\_5\_\_Actinoplanes | 0.0% | 0.0% | 0.0% | 0.0% | 0.0% | 0.0% | 0.0% | 0.0% | 0.0% |
|  | D\_0\_\_Bacteria;D\_1\_\_Actinobacteria;D\_2\_\_Actinobacteria;D\_3\_\_Micromonosporales;D\_4\_\_Micromonosporaceae;D\_5\_\_Allocatelliglobosispora | 0.0% | 0.0% | 0.0% | 0.0% | 0.0% | 0.0% | 0.0% | 0.0% | 0.0% |
|  | D\_0\_\_Bacteria;D\_1\_\_Actinobacteria;D\_2\_\_Actinobacteria;D\_3\_\_Micromonosporales;D\_4\_\_Micromonosporaceae;D\_5\_\_Asanoa | 0.0% | 0.0% | 0.0% | 0.0% | 0.0% | 0.0% | 0.0% | 0.0% | 0.0% |
|  | D\_0\_\_Bacteria;D\_1\_\_Actinobacteria;D\_2\_\_Actinobacteria;D\_3\_\_Micromonosporales;D\_4\_\_Micromonosporaceae;D\_5\_\_Catellatospora | 0.0% | 0.0% | 0.0% | 0.0% | 0.0% | 0.0% | 0.0% | 0.0% | 0.0% |
|  | D\_0\_\_Bacteria;D\_1\_\_Actinobacteria;D\_2\_\_Actinobacteria;D\_3\_\_Micromonosporales;D\_4\_\_Micromonosporaceae;D\_5\_\_Dactylosporangium | 0.0% | 0.0% | 0.0% | 0.0% | 0.0% | 0.0% | 0.0% | 0.0% | 0.0% |
|  | D\_0\_\_Bacteria;D\_1\_\_Actinobacteria;D\_2\_\_Actinobacteria;D\_3\_\_Micromonosporales;D\_4\_\_Micromonosporaceae;D\_5\_\_Hamadaea | 0.0% | 0.1% | 0.0% | 0.1% | 0.0% | 0.1% | 0.0% | 0.0% | 0.0% |
|  | D\_0\_\_Bacteria;D\_1\_\_Actinobacteria;D\_2\_\_Actinobacteria;D\_3\_\_Micromonosporales;D\_4\_\_Micromonosporaceae;D\_5\_\_Longispora | 0.0% | 0.0% | 0.0% | 0.0% | 0.0% | 0.0% | 0.0% | 0.0% | 0.0% |
|  | D\_0\_\_Bacteria;D\_1\_\_Actinobacteria;D\_2\_\_Actinobacteria;D\_3\_\_Micromonosporales;D\_4\_\_Micromonosporaceae;D\_5\_\_Micromonospora | 0.0% | 0.0% | 0.0% | 0.0% | 0.0% | 0.0% | 0.0% | 0.0% | 0.0% |
|  | D\_0\_\_Bacteria;D\_1\_\_Actinobacteria;D\_2\_\_Actinobacteria;D\_3\_\_Micromonosporales;D\_4\_\_Micromonosporaceae;D\_5\_\_Salinispora | 0.0% | 0.0% | 0.0% | 0.0% | 0.0% | 0.0% | 0.0% | 0.0% | 0.0% |
|  | D\_0\_\_Bacteria;D\_1\_\_Actinobacteria;D\_2\_\_Actinobacteria;D\_3\_\_Micromonosporales;D\_4\_\_Micromonosporaceae;D\_5\_\_Stackebrandtia | 0.0% | 0.0% | 0.0% | 0.0% | 0.0% | 0.0% | 0.0% | 0.0% | 0.0% |
|  | D\_0\_\_Bacteria;D\_1\_\_Actinobacteria;D\_2\_\_Actinobacteria;D\_3\_\_Micromonosporales;D\_4\_\_Micromonosporaceae;D\_5\_\_uncultured | 2.6% | 4.5% | 4.9% | 3.3% | 3.6% | 1.9% | 1.8% | 0.6% | 0.3% |
|  | D\_0\_\_Bacteria;D\_1\_\_Actinobacteria;D\_2\_\_Actinobacteria;D\_3\_\_Micromonosporales;D\_4\_\_Micromonosporaceae;Other | 0.8% | 0.4% | 0.4% | 1.7% | 1.5% | 1.3% | 0.9% | 0.1% | 0.1% |
|  | D\_0\_\_Bacteria;D\_1\_\_Actinobacteria;D\_2\_\_Actinobacteria;D\_3\_\_PeM15;D\_4\_\_uncultured bacterium;D\_5\_\_uncultured bacterium | 0.0% | 0.0% | 0.0% | 0.0% | 0.0% | 0.0% | 0.0% | 0.0% | 0.0% |
|  | D\_0\_\_Bacteria;D\_1\_\_Actinobacteria;D\_2\_\_Actinobacteria;D\_3\_\_Propionibacteriales;D\_4\_\_Nocardioidaceae;D\_5\_\_Actinopolymorpha | 0.0% | 0.0% | 0.0% | 0.0% | 0.0% | 0.0% | 0.0% | 0.0% | 0.0% |
|  | D\_0\_\_Bacteria;D\_1\_\_Actinobacteria;D\_2\_\_Actinobacteria;D\_3\_\_Propionibacteriales;D\_4\_\_Nocardioidaceae;D\_5\_\_Aeromicrobium | 0.0% | 0.0% | 0.0% | 0.0% | 0.0% | 0.0% | 0.0% | 0.0% | 0.0% |
|  | D\_0\_\_Bacteria;D\_1\_\_Actinobacteria;D\_2\_\_Actinobacteria;D\_3\_\_Propionibacteriales;D\_4\_\_Nocardioidaceae;D\_5\_\_Flindersiella | 0.1% | 0.0% | 0.0% | 0.0% | 0.0% | 0.3% | 0.6% | 0.0% | 0.0% |
|  | D\_0\_\_Bacteria;D\_1\_\_Actinobacteria;D\_2\_\_Actinobacteria;D\_3\_\_Propionibacteriales;D\_4\_\_Nocardioidaceae;D\_5\_\_Kribbella | 0.0% | 0.0% | 0.0% | 0.0% | 0.0% | 0.0% | 0.0% | 0.0% | 0.0% |
|  | D\_0\_\_Bacteria;D\_1\_\_Actinobacteria;D\_2\_\_Actinobacteria;D\_3\_\_Propionibacteriales;D\_4\_\_Nocardioidaceae;D\_5\_\_Marmoricola | 0.0% | 0.0% | 0.0% | 0.0% | 0.0% | 0.0% | 0.0% | 0.0% | 0.0% |
|  | D\_0\_\_Bacteria;D\_1\_\_Actinobacteria;D\_2\_\_Actinobacteria;D\_3\_\_Propionibacteriales;D\_4\_\_Nocardioidaceae;D\_5\_\_Mumia | 0.0% | 0.0% | 0.0% | 0.0% | 0.0% | 0.0% | 0.0% | 0.0% | 0.0% |
|  | D\_0\_\_Bacteria;D\_1\_\_Actinobacteria;D\_2\_\_Actinobacteria;D\_3\_\_Propionibacteriales;D\_4\_\_Nocardioidaceae;D\_5\_\_Nocardioides | 3.0% | 2.2% | 2.2% | 1.1% | 1.1% | 5.8% | 3.4% | 4.6% | 3.3% |
|  | D\_0\_\_Bacteria;D\_1\_\_Actinobacteria;D\_2\_\_Actinobacteria;D\_3\_\_Propionibacteriales;D\_4\_\_Nocardioidaceae;D\_5\_\_uncultured | 0.6% | 0.8% | 0.8% | 0.4% | 0.4% | 0.4% | 0.7% | 0.4% | 0.5% |
|  | D\_0\_\_Bacteria;D\_1\_\_Actinobacteria;D\_2\_\_Actinobacteria;D\_3\_\_Propionibacteriales;D\_4\_\_Nocardioidaceae;Other | 0.0% | 0.0% | 0.0% | 0.0% | 0.0% | 0.0% | 0.0% | 0.0% | 0.0% |
|  | D\_0\_\_Bacteria;D\_1\_\_Actinobacteria;D\_2\_\_Actinobacteria;D\_3\_\_Propionibacteriales;D\_4\_\_Propionibacteriaceae;Ambiguous\_taxa | 0.0% | 0.0% | 0.0% | 0.0% | 0.0% | 0.0% | 0.0% | 0.0% | 0.0% |
|  | D\_0\_\_Bacteria;D\_1\_\_Actinobacteria;D\_2\_\_Actinobacteria;D\_3\_\_Propionibacteriales;D\_4\_\_Propionibacteriaceae;D\_5\_\_Haloactinopolyspora | 0.0% | 0.0% | 0.0% | 0.0% | 0.0% | 0.0% | 0.0% | 0.0% | 0.0% |
|  | D\_0\_\_Bacteria;D\_1\_\_Actinobacteria;D\_2\_\_Actinobacteria;D\_3\_\_Propionibacteriales;D\_4\_\_Propionibacteriaceae;D\_5\_\_Jiangella | 0.0% | 0.0% | 0.0% | 0.0% | 0.0% | 0.0% | 0.0% | 0.0% | 0.0% |
|  | D\_0\_\_Bacteria;D\_1\_\_Actinobacteria;D\_2\_\_Actinobacteria;D\_3\_\_Propionibacteriales;D\_4\_\_Propionibacteriaceae;D\_5\_\_Phytoactinopolyspora | 0.0% | 0.0% | 0.0% | 0.0% | 0.0% | 0.0% | 0.0% | 0.0% | 0.0% |
|  | D\_0\_\_Bacteria;D\_1\_\_Actinobacteria;D\_2\_\_Actinobacteria;D\_3\_\_Propionibacteriales;D\_4\_\_Propionibacteriaceae;D\_5\_\_Propionibacterium | 0.0% | 0.0% | 0.0% | 0.0% | 0.0% | 0.0% | 0.0% | 0.0% | 0.0% |
|  | D\_0\_\_Bacteria;D\_1\_\_Actinobacteria;D\_2\_\_Actinobacteria;D\_3\_\_Propionibacteriales;D\_4\_\_Propionibacteriaceae;D\_5\_\_Propioniciclava | 0.0% | 0.1% | 0.1% | 0.0% | 0.0% | 0.0% | 0.0% | 0.1% | 0.0% |
|  | D\_0\_\_Bacteria;D\_1\_\_Actinobacteria;D\_2\_\_Actinobacteria;D\_3\_\_Propionibacteriales;D\_4\_\_Propionibacteriaceae;D\_5\_\_uncultured | 0.8% | 0.7% | 0.7% | 0.5% | 0.5% | 2.0% | 1.5% | 0.5% | 0.4% |
|  | D\_0\_\_Bacteria;D\_1\_\_Actinobacteria;D\_2\_\_Actinobacteria;D\_3\_\_Propionibacteriales;D\_4\_\_Propionibacteriaceae;Other | 0.4% | 0.3% | 0.4% | 0.2% | 0.2% | 0.7% | 1.3% | 0.2% | 0.1% |
|  | D\_0\_\_Bacteria;D\_1\_\_Actinobacteria;D\_2\_\_Actinobacteria;D\_3\_\_Propionibacteriales;Other;Other | 0.0% | 0.0% | 0.0% | 0.0% | 0.0% | 0.0% | 0.0% | 0.0% | 0.0% |
|  | D\_0\_\_Bacteria;D\_1\_\_Actinobacteria;D\_2\_\_Actinobacteria;D\_3\_\_Pseudonocardiales;D\_4\_\_Pseudonocardiaceae;Ambiguous\_taxa | 0.0% | 0.0% | 0.0% | 0.0% | 0.0% | 0.0% | 0.0% | 0.0% | 0.0% |
|  | D\_0\_\_Bacteria;D\_1\_\_Actinobacteria;D\_2\_\_Actinobacteria;D\_3\_\_Pseudonocardiales;D\_4\_\_Pseudonocardiaceae;D\_5\_\_Actinoalloteichus | 0.0% | 0.0% | 0.0% | 0.0% | 0.0% | 0.0% | 0.0% | 0.0% | 0.0% |
|  | D\_0\_\_Bacteria;D\_1\_\_Actinobacteria;D\_2\_\_Actinobacteria;D\_3\_\_Pseudonocardiales;D\_4\_\_Pseudonocardiaceae;D\_5\_\_Actinokineospora | 0.0% | 0.0% | 0.0% | 0.0% | 0.0% | 0.0% | 0.0% | 0.0% | 0.0% |
|  | D\_0\_\_Bacteria;D\_1\_\_Actinobacteria;D\_2\_\_Actinobacteria;D\_3\_\_Pseudonocardiales;D\_4\_\_Pseudonocardiaceae;D\_5\_\_Actinomycetospora | 0.0% | 0.0% | 0.0% | 0.0% | 0.0% | 0.0% | 0.0% | 0.0% | 0.0% |
|  | D\_0\_\_Bacteria;D\_1\_\_Actinobacteria;D\_2\_\_Actinobacteria;D\_3\_\_Pseudonocardiales;D\_4\_\_Pseudonocardiaceae;D\_5\_\_Actinophytocola | 0.5% | 0.0% | 0.0% | 0.1% | 0.1% | 1.4% | 2.3% | 0.1% | 0.1% |
|  | D\_0\_\_Bacteria;D\_1\_\_Actinobacteria;D\_2\_\_Actinobacteria;D\_3\_\_Pseudonocardiales;D\_4\_\_Pseudonocardiaceae;D\_5\_\_Amycolatopsis | 5.1% | 2.6% | 2.9% | 7.9% | 7.6% | 4.7% | 7.2% | 4.0% | 4.0% |
|  | D\_0\_\_Bacteria;D\_1\_\_Actinobacteria;D\_2\_\_Actinobacteria;D\_3\_\_Pseudonocardiales;D\_4\_\_Pseudonocardiaceae;D\_5\_\_Crossiella | 7.5% | 5.3% | 5.9% | 4.2% | 3.9% | 2.5% | 2.0% | 16.7% | 19.3% |
|  | D\_0\_\_Bacteria;D\_1\_\_Actinobacteria;D\_2\_\_Actinobacteria;D\_3\_\_Pseudonocardiales;D\_4\_\_Pseudonocardiaceae;D\_5\_\_Kutzneria | 0.0% | 0.0% | 0.0% | 0.0% | 0.0% | 0.0% | 0.0% | 0.0% | 0.0% |
|  | D\_0\_\_Bacteria;D\_1\_\_Actinobacteria;D\_2\_\_Actinobacteria;D\_3\_\_Pseudonocardiales;D\_4\_\_Pseudonocardiaceae;D\_5\_\_Longimycelium | 0.0% | 0.0% | 0.0% | 0.0% | 0.0% | 0.0% | 0.0% | 0.0% | 0.0% |
|  | D\_0\_\_Bacteria;D\_1\_\_Actinobacteria;D\_2\_\_Actinobacteria;D\_3\_\_Pseudonocardiales;D\_4\_\_Pseudonocardiaceae;D\_5\_\_Prauserella | 0.0% | 0.0% | 0.0% | 0.0% | 0.0% | 0.0% | 0.0% | 0.0% | 0.0% |
|  | D\_0\_\_Bacteria;D\_1\_\_Actinobacteria;D\_2\_\_Actinobacteria;D\_3\_\_Pseudonocardiales;D\_4\_\_Pseudonocardiaceae;D\_5\_\_Pseudonocardia | 0.0% | 0.1% | 0.0% | 0.0% | 0.0% | 0.0% | 0.0% | 0.1% | 0.1% |
|  | D\_0\_\_Bacteria;D\_1\_\_Actinobacteria;D\_2\_\_Actinobacteria;D\_3\_\_Pseudonocardiales;D\_4\_\_Pseudonocardiaceae;D\_5\_\_Saccharopolyspora | 0.1% | 0.0% | 0.0% | 0.0% | 0.0% | 0.3% | 0.2% | 0.1% | 0.0% |
|  | D\_0\_\_Bacteria;D\_1\_\_Actinobacteria;D\_2\_\_Actinobacteria;D\_3\_\_Pseudonocardiales;D\_4\_\_Pseudonocardiaceae;D\_5\_\_Saccharothrix | 0.0% | 0.0% | 0.0% | 0.0% | 0.0% | 0.0% | 0.0% | 0.0% | 0.0% |
|  | D\_0\_\_Bacteria;D\_1\_\_Actinobacteria;D\_2\_\_Actinobacteria;D\_3\_\_Pseudonocardiales;D\_4\_\_Pseudonocardiaceae;D\_5\_\_Sciscionella | 0.0% | 0.0% | 0.0% | 0.0% | 0.0% | 0.0% | 0.0% | 0.0% | 0.0% |
|  | D\_0\_\_Bacteria;D\_1\_\_Actinobacteria;D\_2\_\_Actinobacteria;D\_3\_\_Pseudonocardiales;D\_4\_\_Pseudonocardiaceae;D\_5\_\_Thermocrispum | 0.0% | 0.0% | 0.0% | 0.0% | 0.0% | 0.0% | 0.0% | 0.0% | 0.0% |
|  | D\_0\_\_Bacteria;D\_1\_\_Actinobacteria;D\_2\_\_Actinobacteria;D\_3\_\_Pseudonocardiales;D\_4\_\_Pseudonocardiaceae;D\_5\_\_Thermotunica | 0.0% | 0.0% | 0.0% | 0.0% | 0.0% | 0.0% | 0.0% | 0.0% | 0.0% |
|  | D\_0\_\_Bacteria;D\_1\_\_Actinobacteria;D\_2\_\_Actinobacteria;D\_3\_\_Pseudonocardiales;D\_4\_\_Pseudonocardiaceae;D\_5\_\_Umezawaea | 0.0% | 0.0% | 0.0% | 0.0% | 0.0% | 0.0% | 0.0% | 0.0% | 0.0% |
|  | D\_0\_\_Bacteria;D\_1\_\_Actinobacteria;D\_2\_\_Actinobacteria;D\_3\_\_Pseudonocardiales;D\_4\_\_Pseudonocardiaceae;D\_5\_\_Yuhushiella | 0.0% | 0.0% | 0.0% | 0.0% | 0.0% | 0.1% | 0.0% | 0.0% | 0.0% |
|  | D\_0\_\_Bacteria;D\_1\_\_Actinobacteria;D\_2\_\_Actinobacteria;D\_3\_\_Pseudonocardiales;D\_4\_\_Pseudonocardiaceae;Other | 16.2% | 2.9% | 3.2% | 4.1% | 3.9% | 31.2% | 31.1% | 28.3% | 25.1% |
|  | D\_0\_\_Bacteria;D\_1\_\_Actinobacteria;D\_2\_\_Actinobacteria;D\_3\_\_Streptomycetales;D\_4\_\_Streptomycetaceae;D\_5\_\_Kitasatospora | 0.0% | 0.0% | 0.0% | 0.0% | 0.0% | 0.0% | 0.0% | 0.0% | 0.0% |
|  | D\_0\_\_Bacteria;D\_1\_\_Actinobacteria;D\_2\_\_Actinobacteria;D\_3\_\_Streptomycetales;D\_4\_\_Streptomycetaceae;D\_5\_\_Streptomyces | 4.3% | 0.1% | 0.1% | 0.1% | 0.1% | 11.4% | 22.7% | 0.1% | 0.0% |
|  | D\_0\_\_Bacteria;D\_1\_\_Actinobacteria;D\_2\_\_Actinobacteria;D\_3\_\_Streptosporangiales;D\_4\_\_Nocardiopsaceae;D\_5\_\_Nocardiopsis | 0.0% | 0.0% | 0.0% | 0.0% | 0.0% | 0.0% | 0.0% | 0.0% | 0.0% |
|  | D\_0\_\_Bacteria;D\_1\_\_Actinobacteria;D\_2\_\_Actinobacteria;D\_3\_\_Streptosporangiales;D\_4\_\_Nocardiopsaceae;D\_5\_\_Thermobifida | 0.0% | 0.0% | 0.0% | 0.0% | 0.0% | 0.0% | 0.0% | 0.0% | 0.0% |
|  | D\_0\_\_Bacteria;D\_1\_\_Actinobacteria;D\_2\_\_Actinobacteria;D\_3\_\_Streptosporangiales;D\_4\_\_Nocardiopsaceae;Other | 0.0% | 0.0% | 0.0% | 0.0% | 0.0% | 0.0% | 0.0% | 0.0% | 0.0% |
|  | D\_0\_\_Bacteria;D\_1\_\_Actinobacteria;D\_2\_\_Actinobacteria;D\_3\_\_Streptosporangiales;D\_4\_\_Streptosporangiaceae;Ambiguous\_taxa | 0.0% | 0.0% | 0.0% | 0.0% | 0.0% | 0.0% | 0.0% | 0.0% | 0.0% |
|  | D\_0\_\_Bacteria;D\_1\_\_Actinobacteria;D\_2\_\_Actinobacteria;D\_3\_\_Streptosporangiales;D\_4\_\_Streptosporangiaceae;D\_5\_\_Microtetraspora | 0.0% | 0.0% | 0.0% | 0.0% | 0.0% | 0.0% | 0.0% | 0.0% | 0.0% |
|  | D\_0\_\_Bacteria;D\_1\_\_Actinobacteria;D\_2\_\_Actinobacteria;D\_3\_\_Streptosporangiales;D\_4\_\_Streptosporangiaceae;D\_5\_\_Nonomuraea | 0.0% | 0.0% | 0.0% | 0.0% | 0.0% | 0.0% | 0.0% | 0.0% | 0.0% |
|  | D\_0\_\_Bacteria;D\_1\_\_Actinobacteria;D\_2\_\_Actinobacteria;D\_3\_\_Streptosporangiales;D\_4\_\_Streptosporangiaceae;D\_5\_\_Sphaerimonospora | 0.0% | 0.0% | 0.0% | 0.0% | 0.0% | 0.0% | 0.0% | 0.0% | 0.0% |
|  | D\_0\_\_Bacteria;D\_1\_\_Actinobacteria;D\_2\_\_Actinobacteria;D\_3\_\_Streptosporangiales;D\_4\_\_Streptosporangiaceae;D\_5\_\_Streptosporangium | 0.0% | 0.0% | 0.0% | 0.0% | 0.0% | 0.0% | 0.0% | 0.0% | 0.0% |
|  | D\_0\_\_Bacteria;D\_1\_\_Actinobacteria;D\_2\_\_Actinobacteria;D\_3\_\_Streptosporangiales;D\_4\_\_Streptosporangiaceae;Other | 0.0% | 0.0% | 0.0% | 0.0% | 0.0% | 0.0% | 0.0% | 0.0% | 0.0% |
|  | D\_0\_\_Bacteria;D\_1\_\_Actinobacteria;D\_2\_\_Actinobacteria;D\_3\_\_Streptosporangiales;D\_4\_\_Thermomonosporaceae;D\_5\_\_Actinoallomurus | 0.0% | 0.0% | 0.0% | 0.0% | 0.0% | 0.0% | 0.0% | 0.0% | 0.0% |
|  | D\_0\_\_Bacteria;D\_1\_\_Actinobacteria;D\_2\_\_Actinobacteria;D\_3\_\_Streptosporangiales;D\_4\_\_Thermomonosporaceae;D\_5\_\_Actinomadura | 0.0% | 0.0% | 0.0% | 0.0% | 0.0% | 0.0% | 0.0% | 0.0% | 0.0% |
|  | D\_0\_\_Bacteria;D\_1\_\_Actinobacteria;D\_2\_\_Actinobacteria;D\_3\_\_Streptosporangiales;D\_4\_\_Thermomonosporaceae;Other | 0.0% | 0.0% | 0.0% | 0.0% | 0.0% | 0.0% | 0.0% | 0.0% | 0.0% |
|  | D\_0\_\_Bacteria;D\_1\_\_Actinobacteria;D\_2\_\_Actinobacteria;D\_3\_\_Streptosporangiales;D\_4\_\_uncultured bacterium;D\_5\_\_uncultured bacterium | 0.0% | 0.0% | 0.0% | 0.0% | 0.0% | 0.0% | 0.0% | 0.0% | 0.0% |
|  | D\_0\_\_Bacteria;D\_1\_\_Actinobacteria;D\_2\_\_Actinobacteria;D\_3\_\_Streptosporangiales;Other;Other | 0.0% | 0.0% | 0.0% | 0.0% | 0.0% | 0.0% | 0.0% | 0.0% | 0.0% |
|  | D\_0\_\_Bacteria;D\_1\_\_Actinobacteria;D\_2\_\_Actinobacteria;Other;Other;Other | 0.0% | 0.0% | 0.0% | 0.0% | 0.0% | 0.1% | 0.1% | 0.0% | 0.0% |
|  | D\_0\_\_Bacteria;D\_1\_\_Actinobacteria;D\_2\_\_Coriobacteriia;D\_3\_\_Coriobacteriales;D\_4\_\_Coriobacteriaceae;D\_5\_\_Atopobium | 0.0% | 0.0% | 0.0% | 0.0% | 0.0% | 0.0% | 0.0% | 0.0% | 0.0% |
|  | D\_0\_\_Bacteria;D\_1\_\_Actinobacteria;D\_2\_\_Coriobacteriia;D\_3\_\_Coriobacteriales;D\_4\_\_Coriobacteriaceae;D\_5\_\_uncultured | 0.0% | 0.0% | 0.0% | 0.0% | 0.0% | 0.0% | 0.0% | 0.0% | 0.0% |
|  | D\_0\_\_Bacteria;D\_1\_\_Actinobacteria;D\_2\_\_MB-A2-108;Ambiguous\_taxa;Ambiguous\_taxa;Ambiguous\_taxa | 0.0% | 0.0% | 0.0% | 0.0% | 0.0% | 0.0% | 0.0% | 0.0% | 0.0% |
|  | D\_0\_\_Bacteria;D\_1\_\_Actinobacteria;D\_2\_\_MB-A2-108;D\_3\_\_uncultured bacterium;D\_4\_\_uncultured bacterium;D\_5\_\_uncultured bacterium | 0.0% | 0.0% | 0.0% | 0.0% | 0.0% | 0.0% | 0.0% | 0.0% | 0.0% |
|  | D\_0\_\_Bacteria;D\_1\_\_Actinobacteria;D\_2\_\_MB-A2-108;Other;Other;Other | 0.0% | 0.0% | 0.0% | 0.0% | 0.0% | 0.0% | 0.0% | 0.0% | 0.0% |
|  | D\_0\_\_Bacteria;D\_1\_\_Actinobacteria;D\_2\_\_Nitriliruptoria;D\_3\_\_Euzebyales;D\_4\_\_Euzebyaceae;D\_5\_\_Egibacter | 0.0% | 0.0% | 0.0% | 0.0% | 0.0% | 0.0% | 0.0% | 0.0% | 0.0% |
|  | D\_0\_\_Bacteria;D\_1\_\_Actinobacteria;D\_2\_\_Nitriliruptoria;D\_3\_\_Euzebyales;D\_4\_\_Euzebyaceae;D\_5\_\_Euzebya | 0.0% | 0.0% | 0.0% | 0.1% | 0.1% | 0.0% | 0.0% | 0.0% | 0.0% |
|  | D\_0\_\_Bacteria;D\_1\_\_Actinobacteria;D\_2\_\_Nitriliruptoria;D\_3\_\_Euzebyales;D\_4\_\_Euzebyaceae;D\_5\_\_uncultured | 15.3% | 27.4% | 28.0% | 29.0% | 28.5% | 2.3% | 2.6% | 2.4% | 2.1% |
|  | D\_0\_\_Bacteria;D\_1\_\_Actinobacteria;D\_2\_\_Nitriliruptoria;D\_3\_\_Euzebyales;D\_4\_\_Euzebyaceae;Other | 0.0% | 0.0% | 0.0% | 0.0% | 0.0% | 0.0% | 0.0% | 0.0% | 0.0% |
|  | D\_0\_\_Bacteria;D\_1\_\_Actinobacteria;D\_2\_\_Nitriliruptoria;D\_3\_\_Nitriliruptorales;D\_4\_\_Nitriliruptoraceae;D\_5\_\_Egicoccus | 0.0% | 0.0% | 0.0% | 0.0% | 0.0% | 0.0% | 0.0% | 0.0% | 0.0% |
|  | D\_0\_\_Bacteria;D\_1\_\_Actinobacteria;D\_2\_\_Nitriliruptoria;D\_3\_\_Nitriliruptorales;D\_4\_\_Nitriliruptoraceae;D\_5\_\_uncultured actinobacterium | 0.0% | 0.0% | 0.0% | 0.0% | 0.0% | 0.0% | 0.0% | 0.0% | 0.0% |
|  | D\_0\_\_Bacteria;D\_1\_\_Actinobacteria;D\_2\_\_Nitriliruptoria;D\_3\_\_Nitriliruptorales;D\_4\_\_Nitriliruptoraceae;D\_5\_\_uncultured bacterium | 0.0% | 0.1% | 0.0% | 0.0% | 0.0% | 0.0% | 0.0% | 0.0% | 0.0% |
|  | D\_0\_\_Bacteria;D\_1\_\_Actinobacteria;D\_2\_\_Nitriliruptoria;D\_3\_\_Nitriliruptorales;D\_4\_\_Nitriliruptoraceae;Other | 0.0% | 0.0% | 0.0% | 0.0% | 0.0% | 0.0% | 0.0% | 0.0% | 0.0% |
|  | D\_0\_\_Bacteria;D\_1\_\_Actinobacteria;D\_2\_\_Nitriliruptoria;Other;Other;Other | 0.0% | 0.0% | 0.0% | 0.0% | 0.0% | 0.0% | 0.0% | 0.0% | 0.0% |
|  | D\_0\_\_Bacteria;D\_1\_\_Actinobacteria;D\_2\_\_OPB41;D\_3\_\_uncultured bacterium;D\_4\_\_uncultured bacterium;D\_5\_\_uncultured bacterium | 0.0% | 0.0% | 0.0% | 0.0% | 0.0% | 0.0% | 0.0% | 0.0% | 0.0% |
|  | D\_0\_\_Bacteria;D\_1\_\_Actinobacteria;D\_2\_\_Rubrobacteria;D\_3\_\_Rubrobacterales;D\_4\_\_Rubrobacteriaceae;D\_5\_\_Rubrobacter | 0.0% | 0.0% | 0.0% | 0.0% | 0.0% | 0.0% | 0.0% | 0.0% | 0.1% |
|  | D\_0\_\_Bacteria;D\_1\_\_Actinobacteria;D\_2\_\_TakashiAC-B11;D\_3\_\_uncultured Actinomycetales bacterium;D\_4\_\_uncultured Actinomycetales bacterium;D\_5\_\_uncultured Actinomycetales bacterium | 0.0% | 0.0% | 0.0% | 0.0% | 0.0% | 0.0% | 0.0% | 0.0% | 0.0% |
|  | D\_0\_\_Bacteria;D\_1\_\_Actinobacteria;D\_2\_\_TakashiAC-B11;D\_3\_\_uncultured actinobacterium;D\_4\_\_uncultured actinobacterium;D\_5\_\_uncultured actinobacterium | 0.0% | 0.0% | 0.0% | 0.0% | 0.0% | 0.0% | 0.0% | 0.0% | 0.0% |
|  | D\_0\_\_Bacteria;D\_1\_\_Actinobacteria;D\_2\_\_TakashiAC-B11;D\_3\_\_uncultured bacterium;D\_4\_\_uncultured bacterium;D\_5\_\_uncultured bacterium | 0.6% | 0.8% | 0.7% | 1.0% | 1.0% | 0.4% | 0.1% | 0.3% | 0.3% |
|  | D\_0\_\_Bacteria;D\_1\_\_Actinobacteria;D\_2\_\_TakashiAC-B11;Other;Other;Other | 1.7% | 3.1% | 2.9% | 2.4% | 2.2% | 1.0% | 0.1% | 1.0% | 0.8% |
|  | D\_0\_\_Bacteria;D\_1\_\_Actinobacteria;D\_2\_\_Thermoleophilia;D\_3\_\_Gaiellales;D\_4\_\_Gaiellaceae;D\_5\_\_Gaiella | 0.1% | 0.1% | 0.1% | 0.1% | 0.1% | 0.1% | 0.0% | 0.0% | 0.1% |
|  | D\_0\_\_Bacteria;D\_1\_\_Actinobacteria;D\_2\_\_Thermoleophilia;D\_3\_\_Gaiellales;D\_4\_\_uncultured;Ambiguous\_taxa | 0.0% | 0.0% | 0.0% | 0.0% | 0.0% | 0.0% | 0.0% | 0.0% | 0.0% |
|  | D\_0\_\_Bacteria;D\_1\_\_Actinobacteria;D\_2\_\_Thermoleophilia;D\_3\_\_Gaiellales;D\_4\_\_uncultured;D\_5\_\_uncultured bacterium | 0.3% | 0.4% | 0.3% | 0.3% | 0.3% | 0.3% | 0.1% | 0.1% | 0.2% |
|  | D\_0\_\_Bacteria;D\_1\_\_Actinobacteria;D\_2\_\_Thermoleophilia;D\_3\_\_Gaiellales;D\_4\_\_uncultured;D\_5\_\_uncultured microorganism | 0.0% | 0.0% | 0.0% | 0.0% | 0.0% | 0.0% | 0.0% | 0.0% | 0.0% |
|  | D\_0\_\_Bacteria;D\_1\_\_Actinobacteria;D\_2\_\_Thermoleophilia;D\_3\_\_Gaiellales;D\_4\_\_uncultured;Other | 0.0% | 0.0% | 0.0% | 0.0% | 0.0% | 0.0% | 0.0% | 0.0% | 0.0% |
|  | D\_0\_\_Bacteria;D\_1\_\_Actinobacteria;D\_2\_\_Thermoleophilia;D\_3\_\_Solirubrobacterales;Ambiguous\_taxa;Ambiguous\_taxa | 0.0% | 0.0% | 0.0% | 0.0% | 0.0% | 0.0% | 0.0% | 0.0% | 0.0% |
|  | D\_0\_\_Bacteria;D\_1\_\_Actinobacteria;D\_2\_\_Thermoleophilia;D\_3\_\_Solirubrobacterales;D\_4\_\_0319-6M6;D\_5\_\_uncultured actinobacterium | 0.0% | 0.0% | 0.0% | 0.0% | 0.0% | 0.0% | 0.0% | 0.0% | 0.0% |
|  | D\_0\_\_Bacteria;D\_1\_\_Actinobacteria;D\_2\_\_Thermoleophilia;D\_3\_\_Solirubrobacterales;D\_4\_\_0319-6M6;D\_5\_\_uncultured bacterium | 0.0% | 0.0% | 0.0% | 0.0% | 0.0% | 0.0% | 0.0% | 0.0% | 0.0% |
|  | D\_0\_\_Bacteria;D\_1\_\_Actinobacteria;D\_2\_\_Thermoleophilia;D\_3\_\_Solirubrobacterales;D\_4\_\_0319-6M6;Other | 0.0% | 0.0% | 0.0% | 0.0% | 0.0% | 0.0% | 0.0% | 0.0% | 0.0% |
|  | D\_0\_\_Bacteria;D\_1\_\_Actinobacteria;D\_2\_\_Thermoleophilia;D\_3\_\_Solirubrobacterales;D\_4\_\_288-2;D\_5\_\_uncultured bacterium | 0.0% | 0.0% | 0.0% | 0.0% | 0.0% | 0.1% | 0.0% | 0.0% | 0.1% |
|  | D\_0\_\_Bacteria;D\_1\_\_Actinobacteria;D\_2\_\_Thermoleophilia;D\_3\_\_Solirubrobacterales;D\_4\_\_288-2;Other | 0.0% | 0.0% | 0.0% | 0.0% | 0.0% | 0.0% | 0.0% | 0.0% | 0.0% |
|  | D\_0\_\_Bacteria;D\_1\_\_Actinobacteria;D\_2\_\_Thermoleophilia;D\_3\_\_Solirubrobacterales;D\_4\_\_Elev-16S-1332;D\_5\_\_uncultured bacterium | 0.6% | 0.6% | 0.6% | 0.3% | 0.3% | 1.2% | 0.9% | 0.3% | 0.1% |
|  | D\_0\_\_Bacteria;D\_1\_\_Actinobacteria;D\_2\_\_Thermoleophilia;D\_3\_\_Solirubrobacterales;D\_4\_\_Elev-16S-1332;Other | 0.0% | 0.0% | 0.0% | 0.0% | 0.0% | 0.0% | 0.0% | 0.0% | 0.0% |
|  | D\_0\_\_Bacteria;D\_1\_\_Actinobacteria;D\_2\_\_Thermoleophilia;D\_3\_\_Solirubrobacterales;D\_4\_\_FFCH11085;Ambiguous\_taxa | 0.0% | 0.0% | 0.0% | 0.0% | 0.0% | 0.0% | 0.0% | 0.0% | 0.0% |
|  | D\_0\_\_Bacteria;D\_1\_\_Actinobacteria;D\_2\_\_Thermoleophilia;D\_3\_\_Solirubrobacterales;D\_4\_\_Gsoil-1167;D\_5\_\_uncultured bacterium | 0.0% | 0.0% | 0.0% | 0.1% | 0.0% | 0.1% | 0.0% | 0.0% | 0.0% |
|  | D\_0\_\_Bacteria;D\_1\_\_Actinobacteria;D\_2\_\_Thermoleophilia;D\_3\_\_Solirubrobacterales;D\_4\_\_Gsoil-1167;Other | 0.0% | 0.0% | 0.0% | 0.0% | 0.0% | 0.0% | 0.0% | 0.0% | 0.0% |
|  | D\_0\_\_Bacteria;D\_1\_\_Actinobacteria;D\_2\_\_Thermoleophilia;D\_3\_\_Solirubrobacterales;D\_4\_\_Parviterribacteraceae;D\_5\_\_Parviterribacter | 0.0% | 0.0% | 0.0% | 0.0% | 0.0% | 0.0% | 0.0% | 0.0% | 0.0% |
|  | D\_0\_\_Bacteria;D\_1\_\_Actinobacteria;D\_2\_\_Thermoleophilia;D\_3\_\_Solirubrobacterales;D\_4\_\_Patulibacteraceae;D\_5\_\_Patulibacter | 0.0% | 0.0% | 0.0% | 0.0% | 0.0% | 0.0% | 0.0% | 0.0% | 0.0% |
|  | D\_0\_\_Bacteria;D\_1\_\_Actinobacteria;D\_2\_\_Thermoleophilia;D\_3\_\_Solirubrobacterales;D\_4\_\_Q3-6C1;D\_5\_\_uncultured bacterium | 0.0% | 0.0% | 0.0% | 0.0% | 0.0% | 0.0% | 0.0% | 0.0% | 0.0% |
|  | D\_0\_\_Bacteria;D\_1\_\_Actinobacteria;D\_2\_\_Thermoleophilia;D\_3\_\_Solirubrobacterales;D\_4\_\_S1-80;Other | 0.0% | 0.0% | 0.0% | 0.0% | 0.0% | 0.0% | 0.0% | 0.0% | 0.0% |
|  | D\_0\_\_Bacteria;D\_1\_\_Actinobacteria;D\_2\_\_Thermoleophilia;D\_3\_\_Solirubrobacterales;D\_4\_\_Solirubrobacteraceae;D\_5\_\_Solirubrobacter | 0.1% | 0.2% | 0.2% | 0.1% | 0.2% | 0.2% | 0.1% | 0.1% | 0.1% |
|  | D\_0\_\_Bacteria;D\_1\_\_Actinobacteria;D\_2\_\_Thermoleophilia;D\_3\_\_Solirubrobacterales;D\_4\_\_TM146;Ambiguous\_taxa | 0.0% | 0.0% | 0.0% | 0.0% | 0.0% | 0.0% | 0.0% | 0.0% | 0.0% |
|  | D\_0\_\_Bacteria;D\_1\_\_Actinobacteria;D\_2\_\_Thermoleophilia;D\_3\_\_Solirubrobacterales;D\_4\_\_TM146;D\_5\_\_uncultured bacterium | 0.0% | 0.0% | 0.0% | 0.0% | 0.0% | 0.0% | 0.0% | 0.0% | 0.0% |
|  | D\_0\_\_Bacteria;D\_1\_\_Actinobacteria;D\_2\_\_Thermoleophilia;D\_3\_\_Solirubrobacterales;D\_4\_\_TM146;Other | 0.0% | 0.0% | 0.0% | 0.0% | 0.0% | 0.0% | 0.0% | 0.0% | 0.0% |
|  | D\_0\_\_Bacteria;D\_1\_\_Actinobacteria;D\_2\_\_Thermoleophilia;D\_3\_\_Solirubrobacterales;D\_4\_\_YNPFFP1;Other | 0.0% | 0.0% | 0.0% | 0.0% | 0.0% | 0.0% | 0.0% | 0.0% | 0.0% |
|  | D\_0\_\_Bacteria;D\_1\_\_Actinobacteria;D\_2\_\_Thermoleophilia;D\_3\_\_Solirubrobacterales;D\_4\_\_uncultured;D\_5\_\_uncultured bacterium | 0.0% | 0.0% | 0.0% | 0.0% | 0.0% | 0.0% | 0.0% | 0.0% | 0.0% |
|  | D\_0\_\_Bacteria;D\_1\_\_Actinobacteria;D\_2\_\_Thermoleophilia;D\_3\_\_Solirubrobacterales;Other;Other | 0.0% | 0.0% | 0.0% | 0.0% | 0.0% | 0.0% | 0.0% | 0.0% | 0.1% |
|  | D\_0\_\_Bacteria;D\_1\_\_Actinobacteria;D\_2\_\_Thermoleophilia;Other;Other;Other | 0.0% | 0.0% | 0.0% | 0.0% | 0.0% | 0.0% | 0.0% | 0.0% | 0.0% |
|  | D\_0\_\_Bacteria;D\_1\_\_Actinobacteria;Other;Other;Other;Other | 0.0% | 0.0% | 0.0% | 0.0% | 0.0% | 0.0% | 0.0% | 0.0% | 0.0% |
|  | D\_0\_\_Bacteria;D\_1\_\_Armatimonadetes;D\_2\_\_Armatimonadia;D\_3\_\_Armatimonadales;Ambiguous\_taxa;Ambiguous\_taxa | 0.0% | 0.0% | 0.0% | 0.0% | 0.0% | 0.0% | 0.0% | 0.0% | 0.0% |
|  | D\_0\_\_Bacteria;D\_1\_\_Armatimonadetes;D\_2\_\_Armatimonadia;D\_3\_\_Armatimonadales;D\_4\_\_uncultured Armatimonadetes bacterium;D\_5\_\_uncultured Armatimonadetes bacterium | 0.0% | 0.0% | 0.0% | 0.0% | 0.0% | 0.0% | 0.0% | 0.0% | 0.0% |
|  | D\_0\_\_Bacteria;D\_1\_\_Armatimonadetes;D\_2\_\_Armatimonadia;D\_3\_\_Armatimonadales;D\_4\_\_uncultured bacterium;D\_5\_\_uncultured bacterium | 0.0% | 0.0% | 0.0% | 0.0% | 0.0% | 0.0% | 0.0% | 0.0% | 0.0% |
|  | D\_0\_\_Bacteria;D\_1\_\_Armatimonadetes;D\_2\_\_Fimbriimonadia;D\_3\_\_Fimbriimonadales;D\_4\_\_Fimbriimonadaceae;Other | 0.0% | 0.0% | 0.0% | 0.0% | 0.0% | 0.0% | 0.0% | 0.0% | 0.0% |
|  | D\_0\_\_Bacteria;D\_1\_\_Armatimonadetes;D\_2\_\_uncultured;D\_3\_\_uncultured bacterium;D\_4\_\_uncultured bacterium;D\_5\_\_uncultured bacterium | 0.0% | 0.0% | 0.0% | 0.0% | 0.0% | 0.0% | 0.0% | 0.0% | 0.0% |
|  | D\_0\_\_Bacteria;D\_1\_\_BRC1;D\_2\_\_uncultured bacterium;D\_3\_\_uncultured bacterium;D\_4\_\_uncultured bacterium;D\_5\_\_uncultured bacterium | 0.0% | 0.0% | 0.0% | 0.0% | 0.0% | 0.0% | 0.0% | 0.0% | 0.0% |
|  | D\_0\_\_Bacteria;D\_1\_\_BRC1;Other;Other;Other;Other | 0.0% | 0.0% | 0.0% | 0.0% | 0.0% | 0.0% | 0.0% | 0.0% | 0.0% |
|  | D\_0\_\_Bacteria;D\_1\_\_Bacteroidetes;D\_2\_\_Bacteroidetes BD2-2;D\_3\_\_uncultured bacterium;D\_4\_\_uncultured bacterium;D\_5\_\_uncultured bacterium | 0.0% | 0.0% | 0.0% | 0.0% | 0.0% | 0.0% | 0.0% | 0.0% | 0.0% |
|  | D\_0\_\_Bacteria;D\_1\_\_Bacteroidetes;D\_2\_\_Bacteroidetes Incertae Sedis;D\_3\_\_Order II;D\_4\_\_Rhodothermaceae;D\_5\_\_Rubrivirga | 0.0% | 0.0% | 0.0% | 0.0% | 0.0% | 0.0% | 0.0% | 0.0% | 0.0% |
|  | D\_0\_\_Bacteria;D\_1\_\_Bacteroidetes;D\_2\_\_Bacteroidetes Incertae Sedis;D\_3\_\_Order II;D\_4\_\_Rhodothermaceae;Other | 0.0% | 0.0% | 0.0% | 0.0% | 0.0% | 0.0% | 0.0% | 0.0% | 0.0% |
|  | D\_0\_\_Bacteria;D\_1\_\_Bacteroidetes;D\_2\_\_Bacteroidia;D\_3\_\_Bacteroidales;D\_4\_\_Bacteroidaceae;D\_5\_\_Bacteroides | 0.0% | 0.0% | 0.0% | 0.0% | 0.0% | 0.0% | 0.0% | 0.0% | 0.0% |
|  | D\_0\_\_Bacteria;D\_1\_\_Bacteroidetes;D\_2\_\_Bacteroidia;D\_3\_\_Bacteroidales;D\_4\_\_Porphyromonadaceae;D\_5\_\_Parabacteroides | 0.0% | 0.0% | 0.0% | 0.0% | 0.0% | 0.0% | 0.0% | 0.0% | 0.0% |
|  | D\_0\_\_Bacteria;D\_1\_\_Bacteroidetes;D\_2\_\_Bacteroidia;D\_3\_\_Bacteroidales;D\_4\_\_Porphyromonadaceae;D\_5\_\_Porphyromonas | 0.0% | 0.0% | 0.0% | 0.0% | 0.0% | 0.0% | 0.0% | 0.0% | 0.2% |
|  | D\_0\_\_Bacteria;D\_1\_\_Bacteroidetes;D\_2\_\_Bacteroidia;D\_3\_\_Bacteroidales;D\_4\_\_Porphyromonadaceae;D\_5\_\_Proteiniphilum | 0.0% | 0.0% | 0.0% | 0.0% | 0.0% | 0.0% | 0.0% | 0.0% | 0.0% |
|  | D\_0\_\_Bacteria;D\_1\_\_Bacteroidetes;D\_2\_\_Bacteroidia;D\_3\_\_Bacteroidales;D\_4\_\_Porphyromonadaceae;D\_5\_\_Tannerella | 0.0% | 0.0% | 0.0% | 0.0% | 0.0% | 0.0% | 0.0% | 0.0% | 0.0% |
|  | D\_0\_\_Bacteria;D\_1\_\_Bacteroidetes;D\_2\_\_Bacteroidia;D\_3\_\_Bacteroidales;D\_4\_\_Prevotellaceae;D\_5\_\_Alloprevotella | 0.0% | 0.0% | 0.0% | 0.0% | 0.0% | 0.0% | 0.0% | 0.0% | 0.1% |
|  | D\_0\_\_Bacteria;D\_1\_\_Bacteroidetes;D\_2\_\_Bacteroidia;D\_3\_\_Bacteroidales;D\_4\_\_Prevotellaceae;D\_5\_\_Prevotella | 0.0% | 0.0% | 0.0% | 0.0% | 0.0% | 0.0% | 0.0% | 0.0% | 0.2% |
|  | D\_0\_\_Bacteria;D\_1\_\_Bacteroidetes;D\_2\_\_Bacteroidia;D\_3\_\_Bacteroidales;D\_4\_\_Prevotellaceae;D\_5\_\_Prevotella 2 | 0.0% | 0.0% | 0.0% | 0.0% | 0.0% | 0.0% | 0.0% | 0.0% | 0.0% |
|  | D\_0\_\_Bacteria;D\_1\_\_Bacteroidetes;D\_2\_\_Bacteroidia;D\_3\_\_Bacteroidales;D\_4\_\_Prevotellaceae;D\_5\_\_Prevotella 7 | 0.0% | 0.0% | 0.0% | 0.0% | 0.0% | 0.0% | 0.0% | 0.0% | 0.0% |
|  | D\_0\_\_Bacteria;D\_1\_\_Bacteroidetes;D\_2\_\_Cytophagia;D\_3\_\_Cytophagales;D\_4\_\_Cyclobacteriaceae;D\_5\_\_uncultured | 0.0% | 0.0% | 0.0% | 0.0% | 0.0% | 0.0% | 0.0% | 0.0% | 0.0% |
|  | D\_0\_\_Bacteria;D\_1\_\_Bacteroidetes;D\_2\_\_Cytophagia;D\_3\_\_Cytophagales;D\_4\_\_Cytophagaceae;Ambiguous\_taxa | 0.0% | 0.0% | 0.0% | 0.0% | 0.0% | 0.0% | 0.0% | 0.0% | 0.0% |
|  | D\_0\_\_Bacteria;D\_1\_\_Bacteroidetes;D\_2\_\_Cytophagia;D\_3\_\_Cytophagales;D\_4\_\_Cytophagaceae;D\_5\_\_Adhaeribacter | 0.0% | 0.0% | 0.0% | 0.0% | 0.0% | 0.0% | 0.0% | 0.0% | 0.0% |
|  | D\_0\_\_Bacteria;D\_1\_\_Bacteroidetes;D\_2\_\_Cytophagia;D\_3\_\_Cytophagales;D\_4\_\_Cytophagaceae;D\_5\_\_Cytophaga | 0.0% | 0.0% | 0.0% | 0.0% | 0.0% | 0.0% | 0.0% | 0.0% | 0.0% |
|  | D\_0\_\_Bacteria;D\_1\_\_Bacteroidetes;D\_2\_\_Cytophagia;D\_3\_\_Cytophagales;D\_4\_\_Cytophagaceae;D\_5\_\_Hymenobacter | 0.0% | 0.0% | 0.0% | 0.0% | 0.0% | 0.0% | 0.0% | 0.0% | 0.0% |
|  | D\_0\_\_Bacteria;D\_1\_\_Bacteroidetes;D\_2\_\_Cytophagia;D\_3\_\_Cytophagales;D\_4\_\_Cytophagaceae;D\_5\_\_Nibribacter | 0.0% | 0.0% | 0.0% | 0.0% | 0.0% | 0.0% | 0.0% | 0.0% | 0.0% |
|  | D\_0\_\_Bacteria;D\_1\_\_Bacteroidetes;D\_2\_\_Cytophagia;D\_3\_\_Cytophagales;D\_4\_\_Cytophagaceae;D\_5\_\_Ohtaekwangia | 0.0% | 0.0% | 0.0% | 0.0% | 0.0% | 0.0% | 0.0% | 0.0% | 0.0% |
|  | D\_0\_\_Bacteria;D\_1\_\_Bacteroidetes;D\_2\_\_Cytophagia;D\_3\_\_Cytophagales;D\_4\_\_Cytophagaceae;D\_5\_\_Pontibacter | 0.0% | 0.0% | 0.0% | 0.0% | 0.0% | 0.0% | 0.0% | 0.0% | 0.0% |
|  | D\_0\_\_Bacteria;D\_1\_\_Bacteroidetes;D\_2\_\_Cytophagia;D\_3\_\_Cytophagales;D\_4\_\_Cytophagaceae;D\_5\_\_Rhodocytophaga | 0.0% | 0.0% | 0.0% | 0.0% | 0.0% | 0.0% | 0.0% | 0.0% | 0.0% |
|  | D\_0\_\_Bacteria;D\_1\_\_Bacteroidetes;D\_2\_\_Cytophagia;D\_3\_\_Cytophagales;D\_4\_\_Cytophagaceae;D\_5\_\_Sporocytophaga | 0.0% | 0.0% | 0.0% | 0.0% | 0.0% | 0.0% | 0.0% | 0.0% | 0.0% |
|  | D\_0\_\_Bacteria;D\_1\_\_Bacteroidetes;D\_2\_\_Cytophagia;D\_3\_\_Cytophagales;D\_4\_\_Cytophagaceae;D\_5\_\_uncultured | 0.0% | 0.0% | 0.0% | 0.0% | 0.0% | 0.0% | 0.0% | 0.0% | 0.1% |
|  | D\_0\_\_Bacteria;D\_1\_\_Bacteroidetes;D\_2\_\_Cytophagia;D\_3\_\_Cytophagales;D\_4\_\_Cytophagaceae;D\_5\_\_uncultured bacterium | 0.0% | 0.0% | 0.0% | 0.0% | 0.0% | 0.0% | 0.0% | 0.0% | 0.0% |
|  | D\_0\_\_Bacteria;D\_1\_\_Bacteroidetes;D\_2\_\_Cytophagia;D\_3\_\_Cytophagales;D\_4\_\_Flammeovirgaceae;D\_5\_\_Cesiribacter | 0.0% | 0.0% | 0.0% | 0.0% | 0.0% | 0.0% | 0.0% | 0.0% | 0.1% |
|  | D\_0\_\_Bacteria;D\_1\_\_Bacteroidetes;D\_2\_\_Cytophagia;D\_3\_\_Cytophagales;D\_4\_\_Flammeovirgaceae;D\_5\_\_Fulvivirga | 0.0% | 0.0% | 0.0% | 0.0% | 0.0% | 0.0% | 0.0% | 0.0% | 0.0% |
|  | D\_0\_\_Bacteria;D\_1\_\_Bacteroidetes;D\_2\_\_Cytophagia;D\_3\_\_Cytophagales;D\_4\_\_Flammeovirgaceae;D\_5\_\_Imperialibacter | 0.0% | 0.0% | 0.0% | 0.0% | 0.0% | 0.0% | 0.0% | 0.0% | 0.0% |
|  | D\_0\_\_Bacteria;D\_1\_\_Bacteroidetes;D\_2\_\_Cytophagia;D\_3\_\_Cytophagales;D\_4\_\_Flammeovirgaceae;D\_5\_\_Nafulsella | 0.0% | 0.0% | 0.0% | 0.0% | 0.0% | 0.0% | 0.0% | 0.0% | 0.0% |
|  | D\_0\_\_Bacteria;D\_1\_\_Bacteroidetes;D\_2\_\_Cytophagia;D\_3\_\_Cytophagales;D\_4\_\_MWH-CFBk5;D\_5\_\_uncultured bacterium | 0.0% | 0.0% | 0.0% | 0.0% | 0.0% | 0.0% | 0.0% | 0.0% | 0.0% |
|  | D\_0\_\_Bacteria;D\_1\_\_Bacteroidetes;D\_2\_\_Flavobacteriia;D\_3\_\_Flavobacteriales;D\_4\_\_Flavobacteriaceae;D\_5\_\_Bergeyella | 0.0% | 0.0% | 0.0% | 0.0% | 0.0% | 0.0% | 0.0% | 0.0% | 0.1% |
|  | D\_0\_\_Bacteria;D\_1\_\_Bacteroidetes;D\_2\_\_Flavobacteriia;D\_3\_\_Flavobacteriales;D\_4\_\_Flavobacteriaceae;D\_5\_\_Capnocytophaga | 0.0% | 0.0% | 0.0% | 0.0% | 0.0% | 0.0% | 0.0% | 0.0% | 0.1% |
|  | D\_0\_\_Bacteria;D\_1\_\_Bacteroidetes;D\_2\_\_Flavobacteriia;D\_3\_\_Flavobacteriales;D\_4\_\_Flavobacteriaceae;D\_5\_\_Chryseobacterium | 0.0% | 0.0% | 0.0% | 0.0% | 0.0% | 0.0% | 0.0% | 0.0% | 0.0% |
|  | D\_0\_\_Bacteria;D\_1\_\_Bacteroidetes;D\_2\_\_Flavobacteriia;D\_3\_\_Flavobacteriales;D\_4\_\_Flavobacteriaceae;D\_5\_\_Cloacibacterium | 0.0% | 0.0% | 0.0% | 0.0% | 0.0% | 0.0% | 0.0% | 0.0% | 0.0% |
|  | D\_0\_\_Bacteria;D\_1\_\_Bacteroidetes;D\_2\_\_Flavobacteriia;D\_3\_\_Flavobacteriales;D\_4\_\_Flavobacteriaceae;D\_5\_\_Empedobacter | 0.0% | 0.0% | 0.0% | 0.0% | 0.0% | 0.0% | 0.0% | 0.0% | 0.0% |
|  | D\_0\_\_Bacteria;D\_1\_\_Bacteroidetes;D\_2\_\_Flavobacteriia;D\_3\_\_Flavobacteriales;D\_4\_\_Flavobacteriaceae;D\_5\_\_Epilithonimonas | 0.0% | 0.0% | 0.0% | 0.0% | 0.0% | 0.0% | 0.0% | 0.0% | 0.0% |
|  | D\_0\_\_Bacteria;D\_1\_\_Bacteroidetes;D\_2\_\_Flavobacteriia;D\_3\_\_Flavobacteriales;D\_4\_\_Flavobacteriaceae;D\_5\_\_Flavobacterium | 0.0% | 0.0% | 0.0% | 0.0% | 0.0% | 0.0% | 0.0% | 0.0% | 0.0% |
|  | D\_0\_\_Bacteria;D\_1\_\_Bacteroidetes;D\_2\_\_Flavobacteriia;D\_3\_\_Flavobacteriales;D\_4\_\_Flavobacteriaceae;D\_5\_\_Gillisia | 0.0% | 0.0% | 0.0% | 0.0% | 0.0% | 0.0% | 0.0% | 0.0% | 0.0% |
|  | D\_0\_\_Bacteria;D\_1\_\_Bacteroidetes;D\_2\_\_Flavobacteriia;D\_3\_\_Flavobacteriales;D\_4\_\_Flavobacteriaceae;D\_5\_\_Moheibacter | 0.0% | 0.0% | 0.0% | 0.0% | 0.0% | 0.0% | 0.0% | 0.0% | 0.0% |
|  | D\_0\_\_Bacteria;D\_1\_\_Bacteroidetes;D\_2\_\_Flavobacteriia;D\_3\_\_Flavobacteriales;D\_4\_\_Flavobacteriaceae;D\_5\_\_Salinimicrobium | 0.0% | 0.0% | 0.0% | 0.0% | 0.0% | 0.0% | 0.0% | 0.0% | 0.0% |
|  | D\_0\_\_Bacteria;D\_1\_\_Bacteroidetes;D\_2\_\_Flavobacteriia;D\_3\_\_Flavobacteriales;D\_4\_\_NS9 marine group;D\_5\_\_uncultured bacterium | 0.0% | 0.0% | 0.0% | 0.0% | 0.0% | 0.0% | 0.0% | 0.0% | 0.0% |
|  | D\_0\_\_Bacteria;D\_1\_\_Bacteroidetes;D\_2\_\_Sphingobacteriia;D\_3\_\_Sphingobacteriales;D\_4\_\_Chitinophagaceae;D\_5\_\_Chitinophaga | 0.0% | 0.0% | 0.0% | 0.0% | 0.0% | 0.0% | 0.0% | 0.0% | 0.0% |
|  | D\_0\_\_Bacteria;D\_1\_\_Bacteroidetes;D\_2\_\_Sphingobacteriia;D\_3\_\_Sphingobacteriales;D\_4\_\_Chitinophagaceae;D\_5\_\_Cnuella | 0.0% | 0.0% | 0.0% | 0.0% | 0.0% | 0.0% | 0.0% | 0.0% | 0.0% |
|  | D\_0\_\_Bacteria;D\_1\_\_Bacteroidetes;D\_2\_\_Sphingobacteriia;D\_3\_\_Sphingobacteriales;D\_4\_\_Chitinophagaceae;D\_5\_\_Ferruginibacter | 0.0% | 0.0% | 0.0% | 0.0% | 0.0% | 0.0% | 0.0% | 0.0% | 0.0% |
|  | D\_0\_\_Bacteria;D\_1\_\_Bacteroidetes;D\_2\_\_Sphingobacteriia;D\_3\_\_Sphingobacteriales;D\_4\_\_Chitinophagaceae;D\_5\_\_Flavisolibacter | 0.0% | 0.0% | 0.0% | 0.0% | 0.0% | 0.0% | 0.0% | 0.0% | 0.0% |
|  | D\_0\_\_Bacteria;D\_1\_\_Bacteroidetes;D\_2\_\_Sphingobacteriia;D\_3\_\_Sphingobacteriales;D\_4\_\_Chitinophagaceae;D\_5\_\_Heliimonas | 0.0% | 0.0% | 0.0% | 0.0% | 0.0% | 0.0% | 0.0% | 0.0% | 0.0% |
|  | D\_0\_\_Bacteria;D\_1\_\_Bacteroidetes;D\_2\_\_Sphingobacteriia;D\_3\_\_Sphingobacteriales;D\_4\_\_Chitinophagaceae;D\_5\_\_Lacibacter | 0.0% | 0.0% | 0.0% | 0.0% | 0.0% | 0.0% | 0.0% | 0.0% | 0.0% |
|  | D\_0\_\_Bacteria;D\_1\_\_Bacteroidetes;D\_2\_\_Sphingobacteriia;D\_3\_\_Sphingobacteriales;D\_4\_\_Chitinophagaceae;D\_5\_\_Sediminibacterium | 0.0% | 0.0% | 0.0% | 0.0% | 0.0% | 0.0% | 0.0% | 0.0% | 0.0% |
|  | D\_0\_\_Bacteria;D\_1\_\_Bacteroidetes;D\_2\_\_Sphingobacteriia;D\_3\_\_Sphingobacteriales;D\_4\_\_Chitinophagaceae;D\_5\_\_Segetibacter | 0.0% | 0.0% | 0.0% | 0.0% | 0.0% | 0.0% | 0.0% | 0.0% | 0.0% |
|  | D\_0\_\_Bacteria;D\_1\_\_Bacteroidetes;D\_2\_\_Sphingobacteriia;D\_3\_\_Sphingobacteriales;D\_4\_\_Chitinophagaceae;D\_5\_\_uncultured | 0.0% | 0.0% | 0.0% | 0.0% | 0.0% | 0.0% | 0.0% | 0.0% | 0.0% |
|  | D\_0\_\_Bacteria;D\_1\_\_Bacteroidetes;D\_2\_\_Sphingobacteriia;D\_3\_\_Sphingobacteriales;D\_4\_\_Chitinophagaceae;Other | 0.0% | 0.0% | 0.0% | 0.0% | 0.0% | 0.0% | 0.0% | 0.0% | 0.0% |
|  | D\_0\_\_Bacteria;D\_1\_\_Bacteroidetes;D\_2\_\_Sphingobacteriia;D\_3\_\_Sphingobacteriales;D\_4\_\_KD3-93;Ambiguous\_taxa | 0.0% | 0.0% | 0.0% | 0.0% | 0.0% | 0.0% | 0.0% | 0.0% | 0.0% |
|  | D\_0\_\_Bacteria;D\_1\_\_Bacteroidetes;D\_2\_\_Sphingobacteriia;D\_3\_\_Sphingobacteriales;D\_4\_\_KD3-93;D\_5\_\_uncultured bacterium | 0.0% | 0.1% | 0.1% | 0.1% | 0.1% | 0.0% | 0.0% | 0.0% | 0.0% |
|  | D\_0\_\_Bacteria;D\_1\_\_Bacteroidetes;D\_2\_\_Sphingobacteriia;D\_3\_\_Sphingobacteriales;D\_4\_\_Lentimicrobiaceae;Other | 0.0% | 0.0% | 0.0% | 0.0% | 0.0% | 0.0% | 0.0% | 0.0% | 0.0% |
|  | D\_0\_\_Bacteria;D\_1\_\_Bacteroidetes;D\_2\_\_Sphingobacteriia;D\_3\_\_Sphingobacteriales;D\_4\_\_LiUU-11-161;D\_5\_\_uncultured Bacteroidetes bacterium | 0.0% | 0.0% | 0.0% | 0.0% | 0.0% | 0.0% | 0.0% | 0.0% | 0.0% |
|  | D\_0\_\_Bacteria;D\_1\_\_Bacteroidetes;D\_2\_\_Sphingobacteriia;D\_3\_\_Sphingobacteriales;D\_4\_\_NS11-12 marine group;D\_5\_\_uncultured Bacteroidetes bacterium | 0.0% | 0.0% | 0.0% | 0.0% | 0.0% | 0.0% | 0.0% | 0.0% | 0.0% |
|  | D\_0\_\_Bacteria;D\_1\_\_Bacteroidetes;D\_2\_\_Sphingobacteriia;D\_3\_\_Sphingobacteriales;D\_4\_\_Saprospiraceae;D\_5\_\_uncultured | 0.0% | 0.0% | 0.0% | 0.0% | 0.0% | 0.0% | 0.0% | 0.0% | 0.0% |
|  | D\_0\_\_Bacteria;D\_1\_\_Bacteroidetes;D\_2\_\_Sphingobacteriia;D\_3\_\_Sphingobacteriales;D\_4\_\_Sphingobacteriaceae;D\_5\_\_Anseongella | 0.0% | 0.0% | 0.0% | 0.0% | 0.0% | 0.0% | 0.0% | 0.0% | 0.0% |
|  | D\_0\_\_Bacteria;D\_1\_\_Bacteroidetes;D\_2\_\_Sphingobacteriia;D\_3\_\_Sphingobacteriales;D\_4\_\_Sphingobacteriaceae;D\_5\_\_Mucilaginibacter | 0.0% | 0.0% | 0.0% | 0.0% | 0.0% | 0.0% | 0.0% | 0.0% | 0.0% |
|  | D\_0\_\_Bacteria;D\_1\_\_Bacteroidetes;D\_2\_\_Sphingobacteriia;D\_3\_\_Sphingobacteriales;D\_4\_\_Sphingobacteriaceae;D\_5\_\_Pedobacter | 0.0% | 0.0% | 0.0% | 0.0% | 0.0% | 0.0% | 0.0% | 0.0% | 0.0% |
|  | D\_0\_\_Bacteria;D\_1\_\_Bacteroidetes;D\_2\_\_Sphingobacteriia;D\_3\_\_Sphingobacteriales;D\_4\_\_Sphingobacteriaceae;Other | 0.0% | 0.0% | 0.0% | 0.0% | 0.0% | 0.0% | 0.0% | 0.0% | 0.0% |
|  | D\_0\_\_Bacteria;D\_1\_\_Bacteroidetes;D\_2\_\_Sphingobacteriia;D\_3\_\_Sphingobacteriales;D\_4\_\_env.OPS 17;Ambiguous\_taxa | 0.0% | 0.0% | 0.0% | 0.0% | 0.0% | 0.0% | 0.0% | 0.0% | 0.0% |
|  | D\_0\_\_Bacteria;D\_1\_\_Bacteroidetes;D\_2\_\_Sphingobacteriia;D\_3\_\_Sphingobacteriales;D\_4\_\_env.OPS 17;D\_5\_\_uncultured bacterium | 0.0% | 0.0% | 0.0% | 0.0% | 0.0% | 0.0% | 0.0% | 0.0% | 0.0% |
|  | D\_0\_\_Bacteria;D\_1\_\_Bacteroidetes;D\_2\_\_Sphingobacteriia;D\_3\_\_Sphingobacteriales;Other;Other | 0.0% | 0.0% | 0.0% | 0.0% | 0.0% | 0.0% | 0.0% | 0.0% | 0.0% |
|  | D\_0\_\_Bacteria;D\_1\_\_Chlamydiae;D\_2\_\_Chlamydiae;D\_3\_\_Chlamydiales;D\_4\_\_Parachlamydiaceae;D\_5\_\_Candidatus Protochlamydia | 0.0% | 0.0% | 0.0% | 0.0% | 0.0% | 0.0% | 0.0% | 0.0% | 0.0% |
|  | D\_0\_\_Bacteria;D\_1\_\_Chlamydiae;D\_2\_\_Chlamydiae;D\_3\_\_Chlamydiales;D\_4\_\_Parachlamydiaceae;D\_5\_\_Neochlamydia | 0.0% | 0.0% | 0.0% | 0.0% | 0.0% | 0.0% | 0.0% | 0.0% | 0.0% |
|  | D\_0\_\_Bacteria;D\_1\_\_Chlamydiae;D\_2\_\_Chlamydiae;D\_3\_\_Chlamydiales;D\_4\_\_Parachlamydiaceae;D\_5\_\_Parachlamydia | 0.0% | 0.0% | 0.0% | 0.0% | 0.0% | 0.0% | 0.0% | 0.0% | 0.0% |
|  | D\_0\_\_Bacteria;D\_1\_\_Chlamydiae;D\_2\_\_Chlamydiae;D\_3\_\_Chlamydiales;D\_4\_\_Parachlamydiaceae;Other | 0.0% | 0.0% | 0.0% | 0.0% | 0.0% | 0.0% | 0.0% | 0.0% | 0.0% |
|  | D\_0\_\_Bacteria;D\_1\_\_Chlamydiae;D\_2\_\_Chlamydiae;D\_3\_\_Chlamydiales;D\_4\_\_Simkaniaceae;D\_5\_\_uncultured | 0.0% | 0.0% | 0.0% | 0.0% | 0.0% | 0.0% | 0.0% | 0.0% | 0.0% |
|  | D\_0\_\_Bacteria;D\_1\_\_Chlamydiae;D\_2\_\_Chlamydiae;D\_3\_\_Chlamydiales;D\_4\_\_cvE6;Ambiguous\_taxa | 0.0% | 0.0% | 0.0% | 0.0% | 0.0% | 0.0% | 0.0% | 0.0% | 0.0% |
|  | D\_0\_\_Bacteria;D\_1\_\_Chlorobi;D\_2\_\_Chlorobia;D\_3\_\_Chlorobiales;D\_4\_\_OPB56;D\_5\_\_uncultured bacterium | 0.0% | 0.0% | 0.0% | 0.0% | 0.0% | 0.0% | 0.0% | 0.0% | 0.0% |
|  | D\_0\_\_Bacteria;D\_1\_\_Chlorobi;D\_2\_\_Chlorobia;D\_3\_\_Chlorobiales;D\_4\_\_SJA-28;Ambiguous\_taxa | 0.0% | 0.0% | 0.0% | 0.0% | 0.0% | 0.0% | 0.0% | 0.0% | 0.0% |
|  | D\_0\_\_Bacteria;D\_1\_\_Chloroflexi;D\_2\_\_Anaerolineae;D\_3\_\_Anaerolineales;D\_4\_\_Anaerolineaceae;D\_5\_\_Ornatilinea | 0.0% | 0.0% | 0.0% | 0.0% | 0.0% | 0.0% | 0.0% | 0.0% | 0.0% |
|  | D\_0\_\_Bacteria;D\_1\_\_Chloroflexi;D\_2\_\_Anaerolineae;D\_3\_\_Anaerolineales;D\_4\_\_Anaerolineaceae;D\_5\_\_uncultured | 0.0% | 0.0% | 0.0% | 0.0% | 0.0% | 0.0% | 0.0% | 0.0% | 0.0% |
|  | D\_0\_\_Bacteria;D\_1\_\_Chloroflexi;D\_2\_\_Ardenticatenia;D\_3\_\_Ardenticatenales;D\_4\_\_uncultured bacterium;D\_5\_\_uncultured bacterium | 0.0% | 0.0% | 0.0% | 0.0% | 0.0% | 0.0% | 0.0% | 0.0% | 0.0% |
|  | D\_0\_\_Bacteria;D\_1\_\_Chloroflexi;D\_2\_\_Caldilineae;D\_3\_\_Caldilineales;D\_4\_\_Caldilineaceae;D\_5\_\_uncultured | 0.0% | 0.0% | 0.0% | 0.0% | 0.0% | 0.0% | 0.0% | 0.0% | 0.0% |
|  | D\_0\_\_Bacteria;D\_1\_\_Chloroflexi;D\_2\_\_Chloroflexi Incertae Sedis;D\_3\_\_Unknown Order;D\_4\_\_Unknown Family;D\_5\_\_Thermobaculum | 0.0% | 0.0% | 0.0% | 0.0% | 0.0% | 0.0% | 0.0% | 0.0% | 0.0% |
|  | D\_0\_\_Bacteria;D\_1\_\_Chloroflexi;D\_2\_\_Chloroflexia;D\_3\_\_Chloroflexales;D\_4\_\_Roseiflexaceae;D\_5\_\_Roseiflexus | 0.0% | 0.0% | 0.0% | 0.0% | 0.0% | 0.0% | 0.0% | 0.0% | 0.0% |
|  | D\_0\_\_Bacteria;D\_1\_\_Chloroflexi;D\_2\_\_Chloroflexia;D\_3\_\_Herpetosiphonales;D\_4\_\_Herpetosiphonaceae;D\_5\_\_Herpetosiphon | 0.0% | 0.0% | 0.0% | 0.0% | 0.0% | 0.0% | 0.0% | 0.0% | 0.0% |
|  | D\_0\_\_Bacteria;D\_1\_\_Chloroflexi;D\_2\_\_Chloroflexia;D\_3\_\_Kallotenuales;D\_4\_\_AKIW781;D\_5\_\_uncultured bacterium | 0.0% | 0.0% | 0.0% | 0.0% | 0.0% | 0.0% | 0.0% | 0.0% | 0.0% |
|  | D\_0\_\_Bacteria;D\_1\_\_Chloroflexi;D\_2\_\_Gitt-GS-136;Ambiguous\_taxa;Ambiguous\_taxa;Ambiguous\_taxa | 0.0% | 0.0% | 0.0% | 0.0% | 0.0% | 0.0% | 0.0% | 0.0% | 0.0% |
|  | D\_0\_\_Bacteria;D\_1\_\_Chloroflexi;D\_2\_\_Gitt-GS-136;D\_3\_\_uncultured bacterium;D\_4\_\_uncultured bacterium;D\_5\_\_uncultured bacterium | 0.0% | 0.0% | 0.0% | 0.0% | 0.0% | 0.0% | 0.0% | 0.0% | 0.0% |
|  | D\_0\_\_Bacteria;D\_1\_\_Chloroflexi;D\_2\_\_Gitt-GS-136;D\_3\_\_uncultured bacterium #0319-6C24;D\_4\_\_uncultured bacterium #0319-6C24;D\_5\_\_uncultured bacterium #0319-6C24 | 0.0% | 0.0% | 0.0% | 0.0% | 0.0% | 0.0% | 0.0% | 0.0% | 0.0% |
|  | D\_0\_\_Bacteria;D\_1\_\_Chloroflexi;D\_2\_\_Gitt-GS-136;Other;Other;Other | 0.7% | 1.1% | 1.1% | 1.1% | 1.1% | 0.8% | 0.1% | 0.2% | 0.1% |
|  | D\_0\_\_Bacteria;D\_1\_\_Chloroflexi;D\_2\_\_JG30-KF-CM66;Ambiguous\_taxa;Ambiguous\_taxa;Ambiguous\_taxa | 0.0% | 0.0% | 0.0% | 0.0% | 0.0% | 0.0% | 0.0% | 0.0% | 0.0% |
|  | D\_0\_\_Bacteria;D\_1\_\_Chloroflexi;D\_2\_\_JG30-KF-CM66;D\_3\_\_uncultured Chloroflexi bacterium;D\_4\_\_uncultured Chloroflexi bacterium;D\_5\_\_uncultured Chloroflexi bacterium | 0.0% | 0.0% | 0.0% | 0.0% | 0.0% | 0.0% | 0.0% | 0.0% | 0.0% |
|  | D\_0\_\_Bacteria;D\_1\_\_Chloroflexi;D\_2\_\_JG30-KF-CM66;D\_3\_\_uncultured bacterium;D\_4\_\_uncultured bacterium;D\_5\_\_uncultured bacterium | 0.6% | 0.8% | 0.7% | 0.7% | 0.7% | 0.8% | 0.3% | 0.4% | 0.5% |
|  | D\_0\_\_Bacteria;D\_1\_\_Chloroflexi;D\_2\_\_JG30-KF-CM66;Other;Other;Other | 0.1% | 0.2% | 0.2% | 0.2% | 0.2% | 0.1% | 0.0% | 0.1% | 0.0% |
|  | D\_0\_\_Bacteria;D\_1\_\_Chloroflexi;D\_2\_\_JG37-AG-4;D\_3\_\_uncultured bacterium;D\_4\_\_uncultured bacterium;D\_5\_\_uncultured bacterium | 0.0% | 0.0% | 0.0% | 0.0% | 0.0% | 0.0% | 0.0% | 0.0% | 0.0% |
|  | D\_0\_\_Bacteria;D\_1\_\_Chloroflexi;D\_2\_\_KD4-96;Ambiguous\_taxa;Ambiguous\_taxa;Ambiguous\_taxa | 0.0% | 0.0% | 0.0% | 0.0% | 0.0% | 0.0% | 0.0% | 0.0% | 0.0% |
|  | D\_0\_\_Bacteria;D\_1\_\_Chloroflexi;D\_2\_\_KD4-96;D\_3\_\_uncultured bacterium;D\_4\_\_uncultured bacterium;D\_5\_\_uncultured bacterium | 0.1% | 0.1% | 0.1% | 0.1% | 0.1% | 0.0% | 0.0% | 0.0% | 0.2% |
|  | D\_0\_\_Bacteria;D\_1\_\_Chloroflexi;D\_2\_\_KD4-96;Other;Other;Other | 0.0% | 0.0% | 0.0% | 0.0% | 0.0% | 0.0% | 0.0% | 0.0% | 0.0% |
|  | D\_0\_\_Bacteria;D\_1\_\_Chloroflexi;D\_2\_\_Ktedonobacteria;D\_3\_\_C0119;D\_4\_\_uncultured bacterium;D\_5\_\_uncultured bacterium | 0.0% | 0.0% | 0.0% | 0.0% | 0.0% | 0.0% | 0.0% | 0.0% | 0.0% |
|  | D\_0\_\_Bacteria;D\_1\_\_Chloroflexi;D\_2\_\_NLS2-31;D\_3\_\_uncultured bacterium;D\_4\_\_uncultured bacterium;D\_5\_\_uncultured bacterium | 0.0% | 0.0% | 0.0% | 0.0% | 0.0% | 0.0% | 0.0% | 0.0% | 0.0% |
|  | D\_0\_\_Bacteria;D\_1\_\_Chloroflexi;D\_2\_\_P2-11E;D\_3\_\_uncultured bacterium;D\_4\_\_uncultured bacterium;D\_5\_\_uncultured bacterium | 0.0% | 0.0% | 0.0% | 0.0% | 0.0% | 0.0% | 0.0% | 0.0% | 0.0% |
|  | D\_0\_\_Bacteria;D\_1\_\_Chloroflexi;D\_2\_\_S085;Ambiguous\_taxa;Ambiguous\_taxa;Ambiguous\_taxa | 0.0% | 0.0% | 0.0% | 0.0% | 0.0% | 0.0% | 0.0% | 0.0% | 0.0% |
|  | D\_0\_\_Bacteria;D\_1\_\_Chloroflexi;D\_2\_\_S085;D\_3\_\_uncultured Chloroflexi bacterium;D\_4\_\_uncultured Chloroflexi bacterium;D\_5\_\_uncultured Chloroflexi bacterium | 0.0% | 0.0% | 0.0% | 0.0% | 0.0% | 0.0% | 0.0% | 0.0% | 0.0% |
|  | D\_0\_\_Bacteria;D\_1\_\_Chloroflexi;D\_2\_\_S085;D\_3\_\_uncultured bacterium;D\_4\_\_uncultured bacterium;D\_5\_\_uncultured bacterium | 0.5% | 0.9% | 0.8% | 0.6% | 0.6% | 0.4% | 0.1% | 0.2% | 0.5% |
|  | D\_0\_\_Bacteria;D\_1\_\_Chloroflexi;D\_2\_\_S085;Other;Other;Other | 0.0% | 0.0% | 0.0% | 0.0% | 0.0% | 0.0% | 0.0% | 0.0% | 0.0% |
|  | D\_0\_\_Bacteria;D\_1\_\_Chloroflexi;D\_2\_\_SAR202 clade;Ambiguous\_taxa;Ambiguous\_taxa;Ambiguous\_taxa | 0.0% | 0.0% | 0.0% | 0.0% | 0.0% | 0.0% | 0.0% | 0.0% | 0.0% |
|  | D\_0\_\_Bacteria;D\_1\_\_Chloroflexi;D\_2\_\_SAR202 clade;D\_3\_\_uncultured Chloroflexi bacterium;D\_4\_\_uncultured Chloroflexi bacterium;D\_5\_\_uncultured Chloroflexi bacterium | 0.0% | 0.0% | 0.0% | 0.0% | 0.0% | 0.0% | 0.0% | 0.0% | 0.0% |
|  | D\_0\_\_Bacteria;D\_1\_\_Chloroflexi;D\_2\_\_SAR202 clade;D\_3\_\_uncultured bacterium;D\_4\_\_uncultured bacterium;D\_5\_\_uncultured bacterium | 0.1% | 0.1% | 0.0% | 0.1% | 0.1% | 0.0% | 0.0% | 0.1% | 0.0% |
|  | D\_0\_\_Bacteria;D\_1\_\_Chloroflexi;D\_2\_\_SAR202 clade;Other;Other;Other | 0.0% | 0.0% | 0.0% | 0.0% | 0.0% | 0.0% | 0.0% | 0.0% | 0.0% |
|  | D\_0\_\_Bacteria;D\_1\_\_Chloroflexi;D\_2\_\_TK10;D\_3\_\_uncultured Chloroflexi bacterium;D\_4\_\_uncultured Chloroflexi bacterium;D\_5\_\_uncultured Chloroflexi bacterium | 0.0% | 0.0% | 0.0% | 0.0% | 0.0% | 0.0% | 0.0% | 0.0% | 0.0% |
|  | D\_0\_\_Bacteria;D\_1\_\_Chloroflexi;D\_2\_\_TK10;D\_3\_\_uncultured bacterium;D\_4\_\_uncultured bacterium;D\_5\_\_uncultured bacterium | 0.2% | 0.4% | 0.3% | 0.3% | 0.3% | 0.3% | 0.1% | 0.2% | 0.1% |
|  | D\_0\_\_Bacteria;D\_1\_\_Chloroflexi;D\_2\_\_TK10;D\_3\_\_uncultured soil bacterium;D\_4\_\_uncultured soil bacterium;D\_5\_\_uncultured soil bacterium | 0.0% | 0.0% | 0.0% | 0.0% | 0.0% | 0.0% | 0.0% | 0.0% | 0.0% |
|  | D\_0\_\_Bacteria;D\_1\_\_Chloroflexi;D\_2\_\_TK10;Other;Other;Other | 0.0% | 0.0% | 0.0% | 0.0% | 0.0% | 0.0% | 0.0% | 0.0% | 0.0% |
|  | D\_0\_\_Bacteria;D\_1\_\_Chloroflexi;D\_2\_\_Thermomicrobia;D\_3\_\_AKYG1722;D\_4\_\_uncultured Chloroflexi bacterium;D\_5\_\_uncultured Chloroflexi bacterium | 0.0% | 0.0% | 0.0% | 0.0% | 0.0% | 0.0% | 0.0% | 0.0% | 0.0% |
|  | D\_0\_\_Bacteria;D\_1\_\_Chloroflexi;D\_2\_\_Thermomicrobia;D\_3\_\_AKYG1722;D\_4\_\_uncultured Sphaerobacter sp.;D\_5\_\_uncultured Sphaerobacter sp. | 0.0% | 0.0% | 0.0% | 0.0% | 0.0% | 0.0% | 0.0% | 0.0% | 0.0% |
|  | D\_0\_\_Bacteria;D\_1\_\_Chloroflexi;D\_2\_\_Thermomicrobia;D\_3\_\_AKYG1722;D\_4\_\_uncultured bacterium;D\_5\_\_uncultured bacterium | 0.1% | 0.3% | 0.2% | 0.2% | 0.2% | 0.1% | 0.0% | 0.1% | 0.0% |
|  | D\_0\_\_Bacteria;D\_1\_\_Chloroflexi;D\_2\_\_Thermomicrobia;D\_3\_\_AKYG1722;Other;Other | 0.1% | 0.1% | 0.1% | 0.0% | 0.0% | 0.0% | 0.1% | 0.1% | 0.2% |
|  | D\_0\_\_Bacteria;D\_1\_\_Chloroflexi;D\_2\_\_Thermomicrobia;D\_3\_\_JG30-KF-CM45;Ambiguous\_taxa;Ambiguous\_taxa | 0.0% | 0.0% | 0.0% | 0.0% | 0.0% | 0.0% | 0.0% | 0.0% | 0.0% |
|  | D\_0\_\_Bacteria;D\_1\_\_Chloroflexi;D\_2\_\_Thermomicrobia;D\_3\_\_JG30-KF-CM45;D\_4\_\_uncultured Chloroflexi bacterium;D\_5\_\_uncultured Chloroflexi bacterium | 0.0% | 0.0% | 0.0% | 0.0% | 0.0% | 0.0% | 0.0% | 0.0% | 0.0% |
|  | D\_0\_\_Bacteria;D\_1\_\_Chloroflexi;D\_2\_\_Thermomicrobia;D\_3\_\_JG30-KF-CM45;D\_4\_\_uncultured bacterium;D\_5\_\_uncultured bacterium | 0.1% | 0.2% | 0.2% | 0.0% | 0.0% | 0.1% | 0.0% | 0.3% | 0.2% |
|  | D\_0\_\_Bacteria;D\_1\_\_Chloroflexi;D\_2\_\_Thermomicrobia;D\_3\_\_Sphaerobacterales;D\_4\_\_Sphaerobacteraceae;D\_5\_\_Nitrolancea | 0.0% | 0.0% | 0.0% | 0.0% | 0.0% | 0.0% | 0.0% | 0.0% | 0.0% |
|  | D\_0\_\_Bacteria;D\_1\_\_Chloroflexi;D\_2\_\_Thermomicrobia;D\_3\_\_Sphaerobacterales;D\_4\_\_Sphaerobacteraceae;D\_5\_\_Sphaerobacter | 0.0% | 0.0% | 0.0% | 0.0% | 0.0% | 0.0% | 0.0% | 0.0% | 0.0% |
|  | D\_0\_\_Bacteria;D\_1\_\_Chloroflexi;Other;Other;Other;Other | 0.0% | 0.0% | 0.0% | 0.0% | 0.0% | 0.0% | 0.0% | 0.0% | 0.0% |
|  | D\_0\_\_Bacteria;D\_1\_\_Cyanobacteria;D\_2\_\_Chloroplast;Ambiguous\_taxa;Ambiguous\_taxa;Ambiguous\_taxa | 0.0% | 0.0% | 0.0% | 0.0% | 0.0% | 0.0% | 0.0% | 0.0% | 0.0% |
|  | D\_0\_\_Bacteria;D\_1\_\_Cyanobacteria;D\_2\_\_Chloroplast;D\_3\_\_uncultured bacterium;D\_4\_\_uncultured bacterium;D\_5\_\_uncultured bacterium | 0.0% | 0.0% | 0.0% | 0.0% | 0.0% | 0.0% | 0.0% | 0.0% | 0.0% |
|  | D\_0\_\_Bacteria;D\_1\_\_Cyanobacteria;D\_2\_\_Cyanobacteria;D\_3\_\_SubsectionIII;D\_4\_\_FamilyI;D\_5\_\_Leptolyngbya | 0.0% | 0.0% | 0.0% | 0.0% | 0.0% | 0.0% | 0.0% | 0.0% | 0.0% |
|  | D\_0\_\_Bacteria;D\_1\_\_Cyanobacteria;D\_2\_\_Cyanobacteria;D\_3\_\_SubsectionIII;D\_4\_\_FamilyI;D\_5\_\_Microcoleus | 0.0% | 0.0% | 0.0% | 0.0% | 0.0% | 0.0% | 0.0% | 0.0% | 0.0% |
|  | D\_0\_\_Bacteria;D\_1\_\_Cyanobacteria;D\_2\_\_Cyanobacteria;D\_3\_\_uncultured;D\_4\_\_uncultured bacterium;D\_5\_\_uncultured bacterium | 0.0% | 0.0% | 0.0% | 0.0% | 0.0% | 0.0% | 0.0% | 0.0% | 0.0% |
|  | D\_0\_\_Bacteria;D\_1\_\_Cyanobacteria;D\_2\_\_Melainabacteria;D\_3\_\_Gastranaerophilales;Other;Other | 0.0% | 0.0% | 0.0% | 0.0% | 0.0% | 0.0% | 0.0% | 0.0% | 0.0% |
|  | D\_0\_\_Bacteria;D\_1\_\_Cyanobacteria;D\_2\_\_Melainabacteria;D\_3\_\_Vampirovibrionales;D\_4\_\_uncultured bacterium;D\_5\_\_uncultured bacterium | 0.0% | 0.0% | 0.0% | 0.0% | 0.0% | 0.0% | 0.0% | 0.0% | 0.0% |
|  | D\_0\_\_Bacteria;D\_1\_\_Deinococcus-Thermus;D\_2\_\_Deinococci;D\_3\_\_Deinococcales;D\_4\_\_Deinococcaceae;D\_5\_\_Deinococcus | 0.0% | 0.0% | 0.0% | 0.0% | 0.0% | 0.0% | 0.0% | 0.0% | 0.0% |
|  | D\_0\_\_Bacteria;D\_1\_\_Deinococcus-Thermus;D\_2\_\_Deinococci;D\_3\_\_Deinococcales;D\_4\_\_Trueperaceae;D\_5\_\_Truepera | 0.0% | 0.0% | 0.0% | 0.0% | 0.0% | 0.0% | 0.0% | 0.0% | 0.1% |
|  | D\_0\_\_Bacteria;D\_1\_\_Deinococcus-Thermus;D\_2\_\_Deinococci;D\_3\_\_Thermales;D\_4\_\_Thermaceae;D\_5\_\_Thermus | 0.0% | 0.0% | 0.0% | 0.0% | 0.0% | 0.0% | 0.0% | 0.0% | 0.0% |
|  | D\_0\_\_Bacteria;D\_1\_\_Elusimicrobia;D\_2\_\_Elusimicrobia;D\_3\_\_Lineage IIa;D\_4\_\_uncultured bacterium;D\_5\_\_uncultured bacterium | 0.0% | 0.0% | 0.0% | 0.0% | 0.0% | 0.0% | 0.0% | 0.0% | 0.0% |
|  | D\_0\_\_Bacteria;D\_1\_\_Elusimicrobia;D\_2\_\_Elusimicrobia;D\_3\_\_Lineage IIb;D\_4\_\_uncultured bacterium;D\_5\_\_uncultured bacterium | 0.0% | 0.0% | 0.0% | 0.0% | 0.0% | 0.0% | 0.0% | 0.0% | 0.0% |
|  | D\_0\_\_Bacteria;D\_1\_\_Elusimicrobia;D\_2\_\_Elusimicrobia;D\_3\_\_Lineage IV;D\_4\_\_uncultured bacterium;D\_5\_\_uncultured bacterium | 0.0% | 0.0% | 0.0% | 0.0% | 0.0% | 0.0% | 0.0% | 0.0% | 0.0% |
|  | D\_0\_\_Bacteria;D\_1\_\_FBP;D\_2\_\_uncultured bacterium;D\_3\_\_uncultured bacterium;D\_4\_\_uncultured bacterium;D\_5\_\_uncultured bacterium | 0.0% | 0.0% | 0.0% | 0.0% | 0.0% | 0.0% | 0.0% | 0.0% | 0.1% |
|  | D\_0\_\_Bacteria;D\_1\_\_Fibrobacteres;D\_2\_\_Fibrobacteria;D\_3\_\_Fibrobacterales;D\_4\_\_Fibrobacteraceae;D\_5\_\_possible genus 04 | 0.0% | 0.0% | 0.0% | 0.0% | 0.0% | 0.0% | 0.0% | 0.0% | 0.0% |
|  | D\_0\_\_Bacteria;D\_1\_\_Fibrobacteres;D\_2\_\_Fibrobacteria;D\_3\_\_Fibrobacterales;D\_4\_\_Fibrobacteraceae;D\_5\_\_uncultured | 0.0% | 0.0% | 0.0% | 0.0% | 0.0% | 0.0% | 0.0% | 0.0% | 0.0% |
|  | D\_0\_\_Bacteria;D\_1\_\_Firmicutes;D\_2\_\_Bacilli;D\_3\_\_Bacillales;D\_4\_\_Alicyclobacillaceae;D\_5\_\_Tumebacillus | 0.0% | 0.0% | 0.0% | 0.0% | 0.0% | 0.0% | 0.0% | 0.0% | 0.0% |
|  | D\_0\_\_Bacteria;D\_1\_\_Firmicutes;D\_2\_\_Bacilli;D\_3\_\_Bacillales;D\_4\_\_Bacillaceae;D\_5\_\_Bacillus | 0.0% | 0.0% | 0.0% | 0.0% | 0.0% | 0.0% | 0.0% | 0.0% | 0.1% |
|  | D\_0\_\_Bacteria;D\_1\_\_Firmicutes;D\_2\_\_Bacilli;D\_3\_\_Bacillales;D\_4\_\_Bacillaceae;D\_5\_\_Oceanobacillus | 0.0% | 0.0% | 0.0% | 0.0% | 0.0% | 0.0% | 0.0% | 0.0% | 0.0% |
|  | D\_0\_\_Bacteria;D\_1\_\_Firmicutes;D\_2\_\_Bacilli;D\_3\_\_Bacillales;D\_4\_\_Bacillaceae;D\_5\_\_Virgibacillus | 0.0% | 0.0% | 0.0% | 0.0% | 0.0% | 0.0% | 0.0% | 0.0% | 0.0% |
|  | D\_0\_\_Bacteria;D\_1\_\_Firmicutes;D\_2\_\_Bacilli;D\_3\_\_Bacillales;D\_4\_\_Bacillaceae;Other | 0.0% | 0.0% | 0.0% | 0.0% | 0.0% | 0.0% | 0.0% | 0.0% | 0.0% |
|  | D\_0\_\_Bacteria;D\_1\_\_Firmicutes;D\_2\_\_Bacilli;D\_3\_\_Bacillales;D\_4\_\_Family X;D\_5\_\_Thermicanus | 0.0% | 0.0% | 0.0% | 0.0% | 0.0% | 0.0% | 0.0% | 0.0% | 0.0% |
|  | D\_0\_\_Bacteria;D\_1\_\_Firmicutes;D\_2\_\_Bacilli;D\_3\_\_Bacillales;D\_4\_\_Family XI;D\_5\_\_Gemella | 0.0% | 0.0% | 0.0% | 0.0% | 0.0% | 0.0% | 0.0% | 0.0% | 0.0% |
|  | D\_0\_\_Bacteria;D\_1\_\_Firmicutes;D\_2\_\_Bacilli;D\_3\_\_Bacillales;D\_4\_\_Paenibacillaceae;D\_5\_\_Ammoniphilus | 0.0% | 0.0% | 0.0% | 0.0% | 0.0% | 0.0% | 0.0% | 0.0% | 0.0% |
|  | D\_0\_\_Bacteria;D\_1\_\_Firmicutes;D\_2\_\_Bacilli;D\_3\_\_Bacillales;D\_4\_\_Paenibacillaceae;D\_5\_\_Paenibacillus | 0.0% | 0.0% | 0.0% | 0.1% | 0.1% | 0.0% | 0.0% | 0.0% | 0.0% |
|  | D\_0\_\_Bacteria;D\_1\_\_Firmicutes;D\_2\_\_Bacilli;D\_3\_\_Bacillales;D\_4\_\_Paenibacillaceae;D\_5\_\_Saccharibacillus | 0.0% | 0.0% | 0.0% | 0.0% | 0.0% | 0.0% | 0.0% | 0.0% | 0.0% |
|  | D\_0\_\_Bacteria;D\_1\_\_Firmicutes;D\_2\_\_Bacilli;D\_3\_\_Bacillales;D\_4\_\_Planococcaceae;Ambiguous\_taxa | 0.0% | 0.0% | 0.0% | 0.0% | 0.0% | 0.0% | 0.0% | 0.0% | 0.0% |
|  | D\_0\_\_Bacteria;D\_1\_\_Firmicutes;D\_2\_\_Bacilli;D\_3\_\_Bacillales;D\_4\_\_Planococcaceae;D\_5\_\_Chryseomicrobium | 0.0% | 0.0% | 0.0% | 0.0% | 0.0% | 0.0% | 0.0% | 0.0% | 0.0% |
|  | D\_0\_\_Bacteria;D\_1\_\_Firmicutes;D\_2\_\_Bacilli;D\_3\_\_Bacillales;D\_4\_\_Planococcaceae;D\_5\_\_Lysinibacillus | 0.0% | 0.0% | 0.0% | 0.0% | 0.0% | 0.0% | 0.0% | 0.0% | 0.0% |
|  | D\_0\_\_Bacteria;D\_1\_\_Firmicutes;D\_2\_\_Bacilli;D\_3\_\_Bacillales;D\_4\_\_Planococcaceae;D\_5\_\_Paenisporosarcina | 0.0% | 0.0% | 0.0% | 0.0% | 0.0% | 0.0% | 0.0% | 0.0% | 0.0% |
|  | D\_0\_\_Bacteria;D\_1\_\_Firmicutes;D\_2\_\_Bacilli;D\_3\_\_Bacillales;D\_4\_\_Planococcaceae;D\_5\_\_Planococcus | 0.0% | 0.0% | 0.0% | 0.0% | 0.0% | 0.0% | 0.0% | 0.0% | 0.0% |
|  | D\_0\_\_Bacteria;D\_1\_\_Firmicutes;D\_2\_\_Bacilli;D\_3\_\_Bacillales;D\_4\_\_Planococcaceae;D\_5\_\_Planomicrobium | 0.0% | 0.0% | 0.0% | 0.0% | 0.0% | 0.0% | 0.0% | 0.0% | 0.0% |
|  | D\_0\_\_Bacteria;D\_1\_\_Firmicutes;D\_2\_\_Bacilli;D\_3\_\_Bacillales;D\_4\_\_Planococcaceae;Other | 0.0% | 0.0% | 0.0% | 0.0% | 0.0% | 0.0% | 0.0% | 0.0% | 0.0% |
|  | D\_0\_\_Bacteria;D\_1\_\_Firmicutes;D\_2\_\_Bacilli;D\_3\_\_Bacillales;D\_4\_\_Staphylococcaceae;D\_5\_\_Nosocomiicoccus | 0.0% | 0.0% | 0.0% | 0.0% | 0.0% | 0.0% | 0.0% | 0.0% | 0.0% |
|  | D\_0\_\_Bacteria;D\_1\_\_Firmicutes;D\_2\_\_Bacilli;D\_3\_\_Bacillales;D\_4\_\_Staphylococcaceae;D\_5\_\_Staphylococcus | 0.0% | 0.0% | 0.0% | 0.0% | 0.0% | 0.0% | 0.0% | 0.1% | 0.2% |
|  | D\_0\_\_Bacteria;D\_1\_\_Firmicutes;D\_2\_\_Bacilli;D\_3\_\_Bacillales;D\_4\_\_Thermoactinomycetaceae;D\_5\_\_Thermoactinomyces | 0.0% | 0.0% | 0.0% | 0.0% | 0.0% | 0.0% | 0.0% | 0.0% | 0.0% |
|  | D\_0\_\_Bacteria;D\_1\_\_Firmicutes;D\_2\_\_Bacilli;D\_3\_\_Bacillales;Other;Other | 0.0% | 0.0% | 0.0% | 0.0% | 0.0% | 0.0% | 0.0% | 0.0% | 0.0% |
|  | D\_0\_\_Bacteria;D\_1\_\_Firmicutes;D\_2\_\_Bacilli;D\_3\_\_Lactobacillales;D\_4\_\_Aerococcaceae;D\_5\_\_Aerococcus | 0.0% | 0.0% | 0.0% | 0.0% | 0.0% | 0.0% | 0.0% | 0.0% | 0.0% |
|  | D\_0\_\_Bacteria;D\_1\_\_Firmicutes;D\_2\_\_Bacilli;D\_3\_\_Lactobacillales;D\_4\_\_Aerococcaceae;D\_5\_\_Facklamia | 0.0% | 0.0% | 0.0% | 0.0% | 0.0% | 0.0% | 0.0% | 0.0% | 0.0% |
|  | D\_0\_\_Bacteria;D\_1\_\_Firmicutes;D\_2\_\_Bacilli;D\_3\_\_Lactobacillales;D\_4\_\_Aerococcaceae;Other | 0.0% | 0.0% | 0.0% | 0.0% | 0.0% | 0.0% | 0.0% | 0.0% | 0.0% |
|  | D\_0\_\_Bacteria;D\_1\_\_Firmicutes;D\_2\_\_Bacilli;D\_3\_\_Lactobacillales;D\_4\_\_Carnobacteriaceae;D\_5\_\_Dolosigranulum | 0.0% | 0.0% | 0.0% | 0.0% | 0.0% | 0.0% | 0.0% | 0.0% | 0.0% |
|  | D\_0\_\_Bacteria;D\_1\_\_Firmicutes;D\_2\_\_Bacilli;D\_3\_\_Lactobacillales;D\_4\_\_Carnobacteriaceae;D\_5\_\_Granulicatella | 0.0% | 0.0% | 0.0% | 0.0% | 0.0% | 0.0% | 0.0% | 0.0% | 0.1% |
|  | D\_0\_\_Bacteria;D\_1\_\_Firmicutes;D\_2\_\_Bacilli;D\_3\_\_Lactobacillales;D\_4\_\_Carnobacteriaceae;D\_5\_\_Marinilactibacillus | 0.0% | 0.0% | 0.0% | 0.0% | 0.0% | 0.0% | 0.0% | 0.0% | 0.0% |
|  | D\_0\_\_Bacteria;D\_1\_\_Firmicutes;D\_2\_\_Bacilli;D\_3\_\_Lactobacillales;D\_4\_\_Carnobacteriaceae;D\_5\_\_Trichococcus | 0.0% | 0.0% | 0.0% | 0.0% | 0.0% | 0.0% | 0.0% | 0.0% | 0.0% |
|  | D\_0\_\_Bacteria;D\_1\_\_Firmicutes;D\_2\_\_Bacilli;D\_3\_\_Lactobacillales;D\_4\_\_Carnobacteriaceae;Other | 0.0% | 0.0% | 0.0% | 0.0% | 0.0% | 0.0% | 0.0% | 0.0% | 0.0% |
|  | D\_0\_\_Bacteria;D\_1\_\_Firmicutes;D\_2\_\_Bacilli;D\_3\_\_Lactobacillales;D\_4\_\_Enterococcaceae;D\_5\_\_Enterococcus | 0.0% | 0.0% | 0.0% | 0.0% | 0.0% | 0.0% | 0.0% | 0.0% | 0.0% |
|  | D\_0\_\_Bacteria;D\_1\_\_Firmicutes;D\_2\_\_Bacilli;D\_3\_\_Lactobacillales;D\_4\_\_Streptococcaceae;D\_5\_\_Lactococcus | 0.0% | 0.0% | 0.0% | 0.0% | 0.0% | 0.0% | 0.0% | 0.0% | 0.1% |
|  | D\_0\_\_Bacteria;D\_1\_\_Firmicutes;D\_2\_\_Bacilli;D\_3\_\_Lactobacillales;D\_4\_\_Streptococcaceae;D\_5\_\_Streptococcus | 0.1% | 0.0% | 0.0% | 0.0% | 0.0% | 0.0% | 0.0% | 0.1% | 0.6% |
|  | D\_0\_\_Bacteria;D\_1\_\_Firmicutes;D\_2\_\_Bacilli;D\_3\_\_Lactobacillales;Other;Other | 0.0% | 0.0% | 0.0% | 0.0% | 0.0% | 0.0% | 0.0% | 0.0% | 0.0% |
|  | D\_0\_\_Bacteria;D\_1\_\_Firmicutes;D\_2\_\_Clostridia;D\_3\_\_Clostridiales;D\_4\_\_Clostridiaceae 1;D\_5\_\_Clostridium sensu stricto 13 | 0.0% | 0.0% | 0.0% | 0.0% | 0.0% | 0.0% | 0.0% | 0.0% | 0.0% |
|  | D\_0\_\_Bacteria;D\_1\_\_Firmicutes;D\_2\_\_Clostridia;D\_3\_\_Clostridiales;D\_4\_\_Family XI;D\_5\_\_Anaerococcus | 0.0% | 0.0% | 0.0% | 0.0% | 0.0% | 0.0% | 0.0% | 0.0% | 0.2% |
|  | D\_0\_\_Bacteria;D\_1\_\_Firmicutes;D\_2\_\_Clostridia;D\_3\_\_Clostridiales;D\_4\_\_Family XI;D\_5\_\_Ezakiella | 0.0% | 0.0% | 0.0% | 0.0% | 0.0% | 0.0% | 0.0% | 0.0% | 0.0% |
|  | D\_0\_\_Bacteria;D\_1\_\_Firmicutes;D\_2\_\_Clostridia;D\_3\_\_Clostridiales;D\_4\_\_Family XI;D\_5\_\_Finegoldia | 0.0% | 0.0% | 0.0% | 0.0% | 0.0% | 0.0% | 0.0% | 0.0% | 0.0% |
|  | D\_0\_\_Bacteria;D\_1\_\_Firmicutes;D\_2\_\_Clostridia;D\_3\_\_Clostridiales;D\_4\_\_Family XI;D\_5\_\_Helcococcus | 0.0% | 0.0% | 0.0% | 0.0% | 0.0% | 0.0% | 0.0% | 0.0% | 0.0% |
|  | D\_0\_\_Bacteria;D\_1\_\_Firmicutes;D\_2\_\_Clostridia;D\_3\_\_Clostridiales;D\_4\_\_Family XI;D\_5\_\_Murdochiella | 0.0% | 0.0% | 0.0% | 0.0% | 0.0% | 0.0% | 0.0% | 0.0% | 0.0% |
|  | D\_0\_\_Bacteria;D\_1\_\_Firmicutes;D\_2\_\_Clostridia;D\_3\_\_Clostridiales;D\_4\_\_Family XI;D\_5\_\_Parvimonas | 0.0% | 0.0% | 0.0% | 0.0% | 0.0% | 0.0% | 0.0% | 0.0% | 0.0% |
|  | D\_0\_\_Bacteria;D\_1\_\_Firmicutes;D\_2\_\_Clostridia;D\_3\_\_Clostridiales;D\_4\_\_Family XI;D\_5\_\_Peptoniphilus | 0.0% | 0.0% | 0.0% | 0.0% | 0.0% | 0.0% | 0.0% | 0.0% | 0.0% |
|  | D\_0\_\_Bacteria;D\_1\_\_Firmicutes;D\_2\_\_Clostridia;D\_3\_\_Clostridiales;D\_4\_\_Family XI;D\_5\_\_uncultured | 0.0% | 0.0% | 0.0% | 0.0% | 0.0% | 0.0% | 0.0% | 0.0% | 0.0% |
|  | D\_0\_\_Bacteria;D\_1\_\_Firmicutes;D\_2\_\_Clostridia;D\_3\_\_Clostridiales;D\_4\_\_Family XIII;D\_5\_\_Mogibacterium | 0.0% | 0.0% | 0.0% | 0.0% | 0.0% | 0.0% | 0.0% | 0.0% | 0.0% |
|  | D\_0\_\_Bacteria;D\_1\_\_Firmicutes;D\_2\_\_Clostridia;D\_3\_\_Clostridiales;D\_4\_\_Family XIII;D\_5\_\_[Eubacterium] brachy group | 0.0% | 0.0% | 0.0% | 0.0% | 0.0% | 0.0% | 0.0% | 0.0% | 0.0% |
|  | D\_0\_\_Bacteria;D\_1\_\_Firmicutes;D\_2\_\_Clostridia;D\_3\_\_Clostridiales;D\_4\_\_Family XIII;D\_5\_\_[Eubacterium] nodatum group | 0.0% | 0.0% | 0.0% | 0.0% | 0.0% | 0.0% | 0.0% | 0.0% | 0.0% |
|  | D\_0\_\_Bacteria;D\_1\_\_Firmicutes;D\_2\_\_Clostridia;D\_3\_\_Clostridiales;D\_4\_\_Family XIII;D\_5\_\_[Eubacterium] saphenum group | 0.0% | 0.0% | 0.0% | 0.0% | 0.0% | 0.0% | 0.0% | 0.0% | 0.0% |
|  | D\_0\_\_Bacteria;D\_1\_\_Firmicutes;D\_2\_\_Clostridia;D\_3\_\_Clostridiales;D\_4\_\_Family XIII;D\_5\_\_uncultured | 0.0% | 0.0% | 0.0% | 0.0% | 0.0% | 0.0% | 0.0% | 0.0% | 0.0% |
|  | D\_0\_\_Bacteria;D\_1\_\_Firmicutes;D\_2\_\_Clostridia;D\_3\_\_Clostridiales;D\_4\_\_Heliobacteriaceae;D\_5\_\_Hydrogenispora | 0.0% | 0.0% | 0.0% | 0.0% | 0.0% | 0.0% | 0.0% | 0.0% | 0.0% |
|  | D\_0\_\_Bacteria;D\_1\_\_Firmicutes;D\_2\_\_Clostridia;D\_3\_\_Clostridiales;D\_4\_\_Lachnospiraceae;D\_5\_\_Anaerostipes | 0.0% | 0.0% | 0.0% | 0.0% | 0.0% | 0.0% | 0.0% | 0.0% | 0.0% |
|  | D\_0\_\_Bacteria;D\_1\_\_Firmicutes;D\_2\_\_Clostridia;D\_3\_\_Clostridiales;D\_4\_\_Lachnospiraceae;D\_5\_\_Blautia | 0.0% | 0.0% | 0.0% | 0.0% | 0.0% | 0.0% | 0.0% | 0.0% | 0.0% |
|  | D\_0\_\_Bacteria;D\_1\_\_Firmicutes;D\_2\_\_Clostridia;D\_3\_\_Clostridiales;D\_4\_\_Lachnospiraceae;D\_5\_\_Catonella | 0.0% | 0.0% | 0.0% | 0.0% | 0.0% | 0.0% | 0.0% | 0.0% | 0.0% |
|  | D\_0\_\_Bacteria;D\_1\_\_Firmicutes;D\_2\_\_Clostridia;D\_3\_\_Clostridiales;D\_4\_\_Lachnospiraceae;D\_5\_\_Coprococcus 1 | 0.0% | 0.0% | 0.0% | 0.0% | 0.0% | 0.0% | 0.0% | 0.0% | 0.0% |
|  | D\_0\_\_Bacteria;D\_1\_\_Firmicutes;D\_2\_\_Clostridia;D\_3\_\_Clostridiales;D\_4\_\_Lachnospiraceae;D\_5\_\_Johnsonella | 0.0% | 0.0% | 0.0% | 0.0% | 0.0% | 0.0% | 0.0% | 0.0% | 0.0% |
|  | D\_0\_\_Bacteria;D\_1\_\_Firmicutes;D\_2\_\_Clostridia;D\_3\_\_Clostridiales;D\_4\_\_Lachnospiraceae;D\_5\_\_Lachnoanaerobaculum | 0.0% | 0.0% | 0.0% | 0.0% | 0.0% | 0.0% | 0.0% | 0.0% | 0.0% |
|  | D\_0\_\_Bacteria;D\_1\_\_Firmicutes;D\_2\_\_Clostridia;D\_3\_\_Clostridiales;D\_4\_\_Lachnospiraceae;D\_5\_\_Lachnospiraceae ND3007 group | 0.0% | 0.0% | 0.0% | 0.0% | 0.0% | 0.0% | 0.0% | 0.0% | 0.0% |
|  | D\_0\_\_Bacteria;D\_1\_\_Firmicutes;D\_2\_\_Clostridia;D\_3\_\_Clostridiales;D\_4\_\_Lachnospiraceae;D\_5\_\_Lachnospiraceae UCG-008 | 0.0% | 0.0% | 0.0% | 0.0% | 0.0% | 0.0% | 0.0% | 0.0% | 0.0% |
|  | D\_0\_\_Bacteria;D\_1\_\_Firmicutes;D\_2\_\_Clostridia;D\_3\_\_Clostridiales;D\_4\_\_Lachnospiraceae;D\_5\_\_Oribacterium | 0.0% | 0.0% | 0.0% | 0.0% | 0.0% | 0.0% | 0.0% | 0.0% | 0.1% |
|  | D\_0\_\_Bacteria;D\_1\_\_Firmicutes;D\_2\_\_Clostridia;D\_3\_\_Clostridiales;D\_4\_\_Lachnospiraceae;D\_5\_\_Roseburia | 0.0% | 0.0% | 0.0% | 0.0% | 0.0% | 0.0% | 0.0% | 0.0% | 0.0% |
|  | D\_0\_\_Bacteria;D\_1\_\_Firmicutes;D\_2\_\_Clostridia;D\_3\_\_Clostridiales;D\_4\_\_Lachnospiraceae;D\_5\_\_Shuttleworthia | 0.0% | 0.0% | 0.0% | 0.0% | 0.0% | 0.0% | 0.0% | 0.0% | 0.1% |
|  | D\_0\_\_Bacteria;D\_1\_\_Firmicutes;D\_2\_\_Clostridia;D\_3\_\_Clostridiales;D\_4\_\_Lachnospiraceae;D\_5\_\_uncultured | 0.0% | 0.0% | 0.0% | 0.0% | 0.0% | 0.0% | 0.0% | 0.0% | 0.0% |
|  | D\_0\_\_Bacteria;D\_1\_\_Firmicutes;D\_2\_\_Clostridia;D\_3\_\_Clostridiales;D\_4\_\_Lachnospiraceae;Other | 0.0% | 0.0% | 0.0% | 0.0% | 0.0% | 0.0% | 0.0% | 0.0% | 0.0% |
|  | D\_0\_\_Bacteria;D\_1\_\_Firmicutes;D\_2\_\_Clostridia;D\_3\_\_Clostridiales;D\_4\_\_Peptococcaceae;D\_5\_\_Desulfosporosinus | 0.0% | 0.0% | 0.0% | 0.0% | 0.0% | 0.0% | 0.0% | 0.0% | 0.0% |
|  | D\_0\_\_Bacteria;D\_1\_\_Firmicutes;D\_2\_\_Clostridia;D\_3\_\_Clostridiales;D\_4\_\_Peptococcaceae;D\_5\_\_Peptococcus | 0.0% | 0.0% | 0.0% | 0.0% | 0.0% | 0.0% | 0.0% | 0.0% | 0.0% |
|  | D\_0\_\_Bacteria;D\_1\_\_Firmicutes;D\_2\_\_Clostridia;D\_3\_\_Clostridiales;D\_4\_\_Peptostreptococcaceae;D\_5\_\_Filifactor | 0.0% | 0.0% | 0.0% | 0.0% | 0.0% | 0.0% | 0.0% | 0.0% | 0.1% |
|  | D\_0\_\_Bacteria;D\_1\_\_Firmicutes;D\_2\_\_Clostridia;D\_3\_\_Clostridiales;D\_4\_\_Peptostreptococcaceae;D\_5\_\_Peptoclostridium | 0.0% | 0.0% | 0.0% | 0.0% | 0.0% | 0.0% | 0.0% | 0.0% | 0.0% |
|  | D\_0\_\_Bacteria;D\_1\_\_Firmicutes;D\_2\_\_Clostridia;D\_3\_\_Clostridiales;D\_4\_\_Peptostreptococcaceae;D\_5\_\_Peptostreptococcus | 0.0% | 0.0% | 0.0% | 0.0% | 0.0% | 0.0% | 0.0% | 0.0% | 0.0% |
|  | D\_0\_\_Bacteria;D\_1\_\_Firmicutes;D\_2\_\_Clostridia;D\_3\_\_Clostridiales;D\_4\_\_Peptostreptococcaceae;D\_5\_\_Sporacetigenium | 0.0% | 0.0% | 0.0% | 0.0% | 0.0% | 0.0% | 0.0% | 0.0% | 0.0% |
|  | D\_0\_\_Bacteria;D\_1\_\_Firmicutes;D\_2\_\_Clostridia;D\_3\_\_Clostridiales;D\_4\_\_Peptostreptococcaceae;D\_5\_\_[Eubacterium] yurii group | 0.0% | 0.0% | 0.0% | 0.0% | 0.0% | 0.0% | 0.0% | 0.0% | 0.0% |
|  | D\_0\_\_Bacteria;D\_1\_\_Firmicutes;D\_2\_\_Clostridia;D\_3\_\_Clostridiales;D\_4\_\_Peptostreptococcaceae;Other | 0.0% | 0.0% | 0.0% | 0.0% | 0.0% | 0.0% | 0.0% | 0.0% | 0.0% |
|  | D\_0\_\_Bacteria;D\_1\_\_Firmicutes;D\_2\_\_Clostridia;D\_3\_\_Clostridiales;D\_4\_\_Ruminococcaceae;D\_5\_\_Caproiciproducens | 0.0% | 0.0% | 0.0% | 0.0% | 0.0% | 0.0% | 0.0% | 0.0% | 0.0% |
|  | D\_0\_\_Bacteria;D\_1\_\_Firmicutes;D\_2\_\_Clostridia;D\_3\_\_Clostridiales;D\_4\_\_Ruminococcaceae;D\_5\_\_Faecalibacterium | 0.0% | 0.0% | 0.0% | 0.0% | 0.0% | 0.0% | 0.0% | 0.0% | 0.0% |
|  | D\_0\_\_Bacteria;D\_1\_\_Firmicutes;D\_2\_\_Clostridia;D\_3\_\_Clostridiales;D\_4\_\_Ruminococcaceae;D\_5\_\_Fastidiosipila | 0.0% | 0.0% | 0.0% | 0.0% | 0.0% | 0.0% | 0.0% | 0.0% | 0.0% |
|  | D\_0\_\_Bacteria;D\_1\_\_Firmicutes;D\_2\_\_Clostridia;D\_3\_\_Halanaerobiales;D\_4\_\_ODP1230B8.23;D\_5\_\_uncultured bacterium | 0.0% | 0.0% | 0.0% | 0.0% | 0.0% | 0.0% | 0.0% | 0.0% | 0.0% |
|  | D\_0\_\_Bacteria;D\_1\_\_Firmicutes;D\_2\_\_Clostridia;D\_3\_\_Thermoanaerobacterales;D\_4\_\_Thermoanaerobacteraceae;D\_5\_\_Gelria | 0.0% | 0.0% | 0.0% | 0.0% | 0.0% | 0.0% | 0.0% | 0.0% | 0.0% |
|  | D\_0\_\_Bacteria;D\_1\_\_Firmicutes;D\_2\_\_Erysipelotrichia;D\_3\_\_Erysipelotrichales;D\_4\_\_Erysipelotrichaceae;D\_5\_\_Erysipelotrichaceae UCG-007 | 0.0% | 0.0% | 0.0% | 0.0% | 0.0% | 0.0% | 0.0% | 0.0% | 0.1% |
|  | D\_0\_\_Bacteria;D\_1\_\_Firmicutes;D\_2\_\_Erysipelotrichia;D\_3\_\_Erysipelotrichales;D\_4\_\_Erysipelotrichaceae;D\_5\_\_Solobacterium | 0.0% | 0.0% | 0.0% | 0.0% | 0.0% | 0.0% | 0.0% | 0.0% | 0.0% |
|  | D\_0\_\_Bacteria;D\_1\_\_Firmicutes;D\_2\_\_Erysipelotrichia;D\_3\_\_Erysipelotrichales;D\_4\_\_Erysipelotrichaceae;Other | 0.0% | 0.0% | 0.0% | 0.0% | 0.0% | 0.0% | 0.0% | 0.0% | 0.0% |
|  | D\_0\_\_Bacteria;D\_1\_\_Firmicutes;D\_2\_\_Negativicutes;D\_3\_\_Selenomonadales;D\_4\_\_Veillonellaceae;D\_5\_\_Dialister | 0.0% | 0.0% | 0.0% | 0.0% | 0.0% | 0.0% | 0.0% | 0.0% | 0.0% |
|  | D\_0\_\_Bacteria;D\_1\_\_Firmicutes;D\_2\_\_Negativicutes;D\_3\_\_Selenomonadales;D\_4\_\_Veillonellaceae;D\_5\_\_Selenomonas 3 | 0.0% | 0.0% | 0.0% | 0.0% | 0.0% | 0.0% | 0.0% | 0.0% | 0.0% |
|  | D\_0\_\_Bacteria;D\_1\_\_Firmicutes;D\_2\_\_Negativicutes;D\_3\_\_Selenomonadales;D\_4\_\_Veillonellaceae;D\_5\_\_Veillonella | 0.0% | 0.0% | 0.0% | 0.0% | 0.0% | 0.0% | 0.0% | 0.0% | 0.0% |
|  | D\_0\_\_Bacteria;D\_1\_\_Fusobacteria;D\_2\_\_Fusobacteriia;D\_3\_\_Fusobacteriales;D\_4\_\_Fusobacteriaceae;D\_5\_\_Fusobacterium | 0.1% | 0.0% | 0.0% | 0.0% | 0.0% | 0.0% | 0.0% | 0.1% | 0.5% |
|  | D\_0\_\_Bacteria;D\_1\_\_Fusobacteria;D\_2\_\_Fusobacteriia;D\_3\_\_Fusobacteriales;D\_4\_\_Leptotrichiaceae;D\_5\_\_Leptotrichia | 0.0% | 0.0% | 0.0% | 0.0% | 0.0% | 0.0% | 0.0% | 0.0% | 0.1% |
|  | D\_0\_\_Bacteria;D\_1\_\_Gemmatimonadetes;D\_2\_\_BD2-11 terrestrial group;D\_3\_\_uncultured bacterium;D\_4\_\_uncultured bacterium;D\_5\_\_uncultured bacterium | 0.0% | 0.0% | 0.0% | 0.0% | 0.0% | 0.0% | 0.0% | 0.0% | 0.0% |
|  | D\_0\_\_Bacteria;D\_1\_\_Gemmatimonadetes;D\_2\_\_Gemmatimonadetes;D\_3\_\_Gemmatimonadales;D\_4\_\_Gemmatimonadaceae;D\_5\_\_Gemmatimonas | 0.0% | 0.0% | 0.0% | 0.0% | 0.0% | 0.0% | 0.0% | 0.0% | 0.0% |
|  | D\_0\_\_Bacteria;D\_1\_\_Gemmatimonadetes;D\_2\_\_Gemmatimonadetes;D\_3\_\_Gemmatimonadales;D\_4\_\_Gemmatimonadaceae;D\_5\_\_Gemmatirosa | 0.0% | 0.0% | 0.0% | 0.0% | 0.0% | 0.0% | 0.0% | 0.0% | 0.0% |
|  | D\_0\_\_Bacteria;D\_1\_\_Gemmatimonadetes;D\_2\_\_Gemmatimonadetes;D\_3\_\_Gemmatimonadales;D\_4\_\_Gemmatimonadaceae;D\_5\_\_uncultured | 0.0% | 0.0% | 0.0% | 0.0% | 0.0% | 0.0% | 0.0% | 0.0% | 0.0% |
|  | D\_0\_\_Bacteria;D\_1\_\_Gemmatimonadetes;D\_2\_\_Gemmatimonadetes;D\_3\_\_Gemmatimonadales;D\_4\_\_Gemmatimonadaceae;Other | 0.0% | 0.0% | 0.0% | 0.0% | 0.0% | 0.0% | 0.0% | 0.0% | 0.0% |
|  | D\_0\_\_Bacteria;D\_1\_\_Gemmatimonadetes;D\_2\_\_Longimicrobia;D\_3\_\_Longimicrobiales;D\_4\_\_Longimicrobiaceae;D\_5\_\_Longimicrobium | 0.0% | 0.0% | 0.0% | 0.0% | 0.0% | 0.0% | 0.0% | 0.0% | 0.0% |
|  | D\_0\_\_Bacteria;D\_1\_\_Gemmatimonadetes;D\_2\_\_Longimicrobia;D\_3\_\_Longimicrobiales;D\_4\_\_Longimicrobiaceae;D\_5\_\_uncultured bacterium | 0.3% | 0.3% | 0.3% | 0.2% | 0.2% | 0.8% | 0.2% | 0.1% | 0.1% |
|  | D\_0\_\_Bacteria;D\_1\_\_Gemmatimonadetes;D\_2\_\_S0134 terrestrial group;D\_3\_\_uncultured Gemmatimonadetes bacterium;D\_4\_\_uncultured Gemmatimonadetes bacterium;D\_5\_\_uncultured Gemmatimonadetes bacterium | 0.0% | 0.0% | 0.0% | 0.0% | 0.0% | 0.0% | 0.0% | 0.0% | 0.0% |
|  | D\_0\_\_Bacteria;D\_1\_\_Gemmatimonadetes;D\_2\_\_S0134 terrestrial group;D\_3\_\_uncultured bacterium;D\_4\_\_uncultured bacterium;D\_5\_\_uncultured bacterium | 0.0% | 0.0% | 0.0% | 0.0% | 0.0% | 0.0% | 0.0% | 0.0% | 0.1% |
|  | D\_0\_\_Bacteria;D\_1\_\_Hydrogenedentes;D\_2\_\_uncultured bacterium;D\_3\_\_uncultured bacterium;D\_4\_\_uncultured bacterium;D\_5\_\_uncultured bacterium | 0.0% | 0.0% | 0.0% | 0.0% | 0.0% | 0.0% | 0.0% | 0.0% | 0.0% |
|  | D\_0\_\_Bacteria;D\_1\_\_Nitrospirae;D\_2\_\_Nitrospira;D\_3\_\_Nitrospirales;D\_4\_\_0319-6A21;Other | 0.0% | 0.0% | 0.0% | 0.0% | 0.0% | 0.0% | 0.0% | 0.0% | 0.0% |
|  | D\_0\_\_Bacteria;D\_1\_\_Nitrospirae;D\_2\_\_Nitrospira;D\_3\_\_Nitrospirales;D\_4\_\_Nitrospiraceae;D\_5\_\_Nitrospira | 0.2% | 0.5% | 0.4% | 0.2% | 0.2% | 0.3% | 0.1% | 0.1% | 0.2% |
|  | D\_0\_\_Bacteria;D\_1\_\_Nitrospirae;D\_2\_\_Nitrospira;D\_3\_\_Nitrospirales;D\_4\_\_Nitrospiraceae;D\_5\_\_uncultured | 0.0% | 0.0% | 0.0% | 0.0% | 0.0% | 0.0% | 0.0% | 0.0% | 0.0% |
|  | D\_0\_\_Bacteria;D\_1\_\_Planctomycetes;D\_2\_\_Phycisphaerae;D\_3\_\_Phycisphaerales;D\_4\_\_Phycisphaeraceae;D\_5\_\_I-8 | 0.0% | 0.0% | 0.0% | 0.0% | 0.0% | 0.0% | 0.0% | 0.0% | 0.0% |
|  | D\_0\_\_Bacteria;D\_1\_\_Planctomycetes;D\_2\_\_Phycisphaerae;D\_3\_\_Phycisphaerales;D\_4\_\_Phycisphaeraceae;D\_5\_\_SM1A02 | 0.0% | 0.0% | 0.0% | 0.0% | 0.0% | 0.0% | 0.0% | 0.0% | 0.0% |
|  | D\_0\_\_Bacteria;D\_1\_\_Planctomycetes;D\_2\_\_Phycisphaerae;D\_3\_\_Phycisphaerales;D\_4\_\_Phycisphaeraceae;D\_5\_\_uncultured | 0.0% | 0.0% | 0.0% | 0.0% | 0.0% | 0.0% | 0.0% | 0.0% | 0.0% |
|  | D\_0\_\_Bacteria;D\_1\_\_Planctomycetes;D\_2\_\_Phycisphaerae;D\_3\_\_Tepidisphaerales;D\_4\_\_Tepidisphaeraceae;Ambiguous\_taxa | 0.0% | 0.0% | 0.0% | 0.0% | 0.0% | 0.0% | 0.0% | 0.0% | 0.0% |
|  | D\_0\_\_Bacteria;D\_1\_\_Planctomycetes;D\_2\_\_Phycisphaerae;D\_3\_\_Tepidisphaerales;D\_4\_\_Tepidisphaeraceae;D\_5\_\_uncultured bacterium | 0.0% | 0.0% | 0.0% | 0.0% | 0.0% | 0.0% | 0.0% | 0.0% | 0.0% |
|  | D\_0\_\_Bacteria;D\_1\_\_Planctomycetes;D\_2\_\_Phycisphaerae;D\_3\_\_Tepidisphaerales;D\_4\_\_Tepidisphaeraceae;Other | 0.0% | 0.0% | 0.0% | 0.0% | 0.0% | 0.0% | 0.0% | 0.0% | 0.0% |
|  | D\_0\_\_Bacteria;D\_1\_\_Planctomycetes;D\_2\_\_Planctomycetacia;D\_3\_\_Planctomycetales;D\_4\_\_Planctomycetaceae;D\_5\_\_Blastopirellula | 0.0% | 0.0% | 0.0% | 0.0% | 0.0% | 0.0% | 0.0% | 0.0% | 0.0% |
|  | D\_0\_\_Bacteria;D\_1\_\_Planctomycetes;D\_2\_\_Planctomycetacia;D\_3\_\_Planctomycetales;D\_4\_\_Planctomycetaceae;D\_5\_\_Gemmata | 0.0% | 0.0% | 0.0% | 0.1% | 0.1% | 0.0% | 0.0% | 0.1% | 0.0% |
|  | D\_0\_\_Bacteria;D\_1\_\_Planctomycetes;D\_2\_\_Planctomycetacia;D\_3\_\_Planctomycetales;D\_4\_\_Planctomycetaceae;D\_5\_\_Isosphaera | 0.0% | 0.0% | 0.0% | 0.0% | 0.0% | 0.0% | 0.0% | 0.0% | 0.0% |
|  | D\_0\_\_Bacteria;D\_1\_\_Planctomycetes;D\_2\_\_Planctomycetacia;D\_3\_\_Planctomycetales;D\_4\_\_Planctomycetaceae;D\_5\_\_Pir4 lineage | 0.0% | 0.0% | 0.0% | 0.0% | 0.0% | 0.0% | 0.0% | 0.0% | 0.0% |
|  | D\_0\_\_Bacteria;D\_1\_\_Planctomycetes;D\_2\_\_Planctomycetacia;D\_3\_\_Planctomycetales;D\_4\_\_Planctomycetaceae;D\_5\_\_Pirellula | 0.0% | 0.0% | 0.0% | 0.0% | 0.0% | 0.0% | 0.0% | 0.0% | 0.0% |
|  | D\_0\_\_Bacteria;D\_1\_\_Planctomycetes;D\_2\_\_Planctomycetacia;D\_3\_\_Planctomycetales;D\_4\_\_Planctomycetaceae;D\_5\_\_Planctomyces | 0.0% | 0.0% | 0.0% | 0.0% | 0.0% | 0.0% | 0.0% | 0.0% | 0.0% |
|  | D\_0\_\_Bacteria;D\_1\_\_Planctomycetes;D\_2\_\_Planctomycetacia;D\_3\_\_Planctomycetales;D\_4\_\_Planctomycetaceae;D\_5\_\_Rhodopirellula | 0.0% | 0.0% | 0.0% | 0.0% | 0.0% | 0.0% | 0.0% | 0.0% | 0.0% |
|  | D\_0\_\_Bacteria;D\_1\_\_Planctomycetes;D\_2\_\_Planctomycetacia;D\_3\_\_Planctomycetales;D\_4\_\_Planctomycetaceae;D\_5\_\_Singulisphaera | 0.0% | 0.0% | 0.0% | 0.0% | 0.0% | 0.0% | 0.0% | 0.0% | 0.0% |
|  | D\_0\_\_Bacteria;D\_1\_\_Planctomycetes;D\_2\_\_Planctomycetacia;D\_3\_\_Planctomycetales;D\_4\_\_Planctomycetaceae;D\_5\_\_uncultured | 0.0% | 0.1% | 0.0% | 0.1% | 0.1% | 0.0% | 0.0% | 0.0% | 0.1% |
|  | D\_0\_\_Bacteria;D\_1\_\_Planctomycetes;D\_2\_\_Planctomycetacia;D\_3\_\_Planctomycetales;D\_4\_\_Planctomycetaceae;Other | 0.0% | 0.0% | 0.0% | 0.0% | 0.0% | 0.0% | 0.0% | 0.0% | 0.0% |
|  | D\_0\_\_Bacteria;D\_1\_\_Proteobacteria;D\_2\_\_Alphaproteobacteria;D\_3\_\_Alphaproteobacteria Incertae Sedis;D\_4\_\_Unknown Family;D\_5\_\_uncultured | 0.0% | 0.0% | 0.0% | 0.0% | 0.0% | 0.0% | 0.0% | 0.0% | 0.0% |
|  | D\_0\_\_Bacteria;D\_1\_\_Proteobacteria;D\_2\_\_Alphaproteobacteria;D\_3\_\_Caulobacterales;D\_4\_\_Caulobacteraceae;Ambiguous\_taxa | 0.0% | 0.0% | 0.0% | 0.0% | 0.0% | 0.0% | 0.0% | 0.0% | 0.0% |
|  | D\_0\_\_Bacteria;D\_1\_\_Proteobacteria;D\_2\_\_Alphaproteobacteria;D\_3\_\_Caulobacterales;D\_4\_\_Caulobacteraceae;D\_5\_\_Brevundimonas | 0.0% | 0.0% | 0.0% | 0.0% | 0.0% | 0.0% | 0.0% | 0.0% | 0.1% |
|  | D\_0\_\_Bacteria;D\_1\_\_Proteobacteria;D\_2\_\_Alphaproteobacteria;D\_3\_\_Caulobacterales;D\_4\_\_Caulobacteraceae;D\_5\_\_Phenylobacterium | 0.0% | 0.0% | 0.0% | 0.0% | 0.0% | 0.0% | 0.0% | 0.0% | 0.0% |
|  | D\_0\_\_Bacteria;D\_1\_\_Proteobacteria;D\_2\_\_Alphaproteobacteria;D\_3\_\_Caulobacterales;D\_4\_\_Caulobacteraceae;D\_5\_\_uncultured | 0.1% | 0.2% | 0.2% | 0.1% | 0.1% | 0.1% | 0.0% | 0.0% | 0.0% |
|  | D\_0\_\_Bacteria;D\_1\_\_Proteobacteria;D\_2\_\_Alphaproteobacteria;D\_3\_\_Rhizobiales;D\_4\_\_1174-901-12;Ambiguous\_taxa | 0.0% | 0.0% | 0.0% | 0.0% | 0.0% | 0.0% | 0.0% | 0.0% | 0.0% |
|  | D\_0\_\_Bacteria;D\_1\_\_Proteobacteria;D\_2\_\_Alphaproteobacteria;D\_3\_\_Rhizobiales;D\_4\_\_1174-901-12;D\_5\_\_uncultured Rhizobiales bacterium | 0.0% | 0.0% | 0.0% | 0.0% | 0.0% | 0.0% | 0.0% | 0.0% | 0.0% |
|  | D\_0\_\_Bacteria;D\_1\_\_Proteobacteria;D\_2\_\_Alphaproteobacteria;D\_3\_\_Rhizobiales;D\_4\_\_1174-901-12;Other | 0.0% | 0.0% | 0.0% | 0.0% | 0.0% | 0.0% | 0.0% | 0.0% | 0.0% |
|  | D\_0\_\_Bacteria;D\_1\_\_Proteobacteria;D\_2\_\_Alphaproteobacteria;D\_3\_\_Rhizobiales;D\_4\_\_A0839;D\_5\_\_uncultured bacterium | 0.0% | 0.0% | 0.0% | 0.0% | 0.0% | 0.0% | 0.0% | 0.0% | 0.0% |
|  | D\_0\_\_Bacteria;D\_1\_\_Proteobacteria;D\_2\_\_Alphaproteobacteria;D\_3\_\_Rhizobiales;D\_4\_\_Aurantimonadaceae;D\_5\_\_Aureimonas | 0.0% | 0.0% | 0.0% | 0.0% | 0.0% | 0.0% | 0.0% | 0.0% | 0.0% |
|  | D\_0\_\_Bacteria;D\_1\_\_Proteobacteria;D\_2\_\_Alphaproteobacteria;D\_3\_\_Rhizobiales;D\_4\_\_Aurantimonadaceae;Other | 0.0% | 0.0% | 0.0% | 0.0% | 0.0% | 0.0% | 0.0% | 0.0% | 0.0% |
|  | D\_0\_\_Bacteria;D\_1\_\_Proteobacteria;D\_2\_\_Alphaproteobacteria;D\_3\_\_Rhizobiales;D\_4\_\_Beijerinckiaceae;D\_5\_\_uncultured | 0.0% | 0.0% | 0.0% | 0.0% | 0.0% | 0.0% | 0.0% | 0.0% | 0.0% |
|  | D\_0\_\_Bacteria;D\_1\_\_Proteobacteria;D\_2\_\_Alphaproteobacteria;D\_3\_\_Rhizobiales;D\_4\_\_Beijerinckiaceae;Other | 0.0% | 0.0% | 0.0% | 0.0% | 0.0% | 0.0% | 0.0% | 0.0% | 0.0% |
|  | D\_0\_\_Bacteria;D\_1\_\_Proteobacteria;D\_2\_\_Alphaproteobacteria;D\_3\_\_Rhizobiales;D\_4\_\_Bradyrhizobiaceae;D\_5\_\_Afipia | 0.1% | 0.1% | 0.1% | 0.1% | 0.1% | 0.0% | 0.0% | 0.0% | 0.0% |
|  | D\_0\_\_Bacteria;D\_1\_\_Proteobacteria;D\_2\_\_Alphaproteobacteria;D\_3\_\_Rhizobiales;D\_4\_\_Bradyrhizobiaceae;D\_5\_\_Bosea | 0.0% | 0.0% | 0.0% | 0.0% | 0.0% | 0.0% | 0.0% | 0.0% | 0.0% |
|  | D\_0\_\_Bacteria;D\_1\_\_Proteobacteria;D\_2\_\_Alphaproteobacteria;D\_3\_\_Rhizobiales;D\_4\_\_Bradyrhizobiaceae;D\_5\_\_Bradyrhizobium | 0.0% | 0.0% | 0.0% | 0.0% | 0.0% | 0.0% | 0.0% | 0.0% | 0.0% |
|  | D\_0\_\_Bacteria;D\_1\_\_Proteobacteria;D\_2\_\_Alphaproteobacteria;D\_3\_\_Rhizobiales;D\_4\_\_Bradyrhizobiaceae;D\_5\_\_Nitrobacter | 0.0% | 0.0% | 0.0% | 0.0% | 0.0% | 0.0% | 0.0% | 0.0% | 0.0% |
|  | D\_0\_\_Bacteria;D\_1\_\_Proteobacteria;D\_2\_\_Alphaproteobacteria;D\_3\_\_Rhizobiales;D\_4\_\_Bradyrhizobiaceae;D\_5\_\_uncultured | 0.0% | 0.0% | 0.0% | 0.1% | 0.1% | 0.0% | 0.0% | 0.0% | 0.0% |
|  | D\_0\_\_Bacteria;D\_1\_\_Proteobacteria;D\_2\_\_Alphaproteobacteria;D\_3\_\_Rhizobiales;D\_4\_\_Bradyrhizobiaceae;Other | 0.0% | 0.0% | 0.0% | 0.0% | 0.0% | 0.0% | 0.0% | 0.0% | 0.0% |
|  | D\_0\_\_Bacteria;D\_1\_\_Proteobacteria;D\_2\_\_Alphaproteobacteria;D\_3\_\_Rhizobiales;D\_4\_\_Brucellaceae;Other | 0.0% | 0.0% | 0.0% | 0.0% | 0.0% | 0.0% | 0.0% | 0.0% | 0.0% |
|  | D\_0\_\_Bacteria;D\_1\_\_Proteobacteria;D\_2\_\_Alphaproteobacteria;D\_3\_\_Rhizobiales;D\_4\_\_C2U;D\_5\_\_uncultured bacterium | 0.0% | 0.0% | 0.0% | 0.0% | 0.0% | 0.0% | 0.0% | 0.0% | 0.0% |
|  | D\_0\_\_Bacteria;D\_1\_\_Proteobacteria;D\_2\_\_Alphaproteobacteria;D\_3\_\_Rhizobiales;D\_4\_\_DUNssu044;Ambiguous\_taxa | 0.1% | 0.1% | 0.1% | 0.2% | 0.2% | 0.4% | 0.1% | 0.1% | 0.0% |
|  | D\_0\_\_Bacteria;D\_1\_\_Proteobacteria;D\_2\_\_Alphaproteobacteria;D\_3\_\_Rhizobiales;D\_4\_\_DUNssu044;D\_5\_\_uncultured bacterium | 0.0% | 0.0% | 0.0% | 0.0% | 0.0% | 0.0% | 0.0% | 0.0% | 0.0% |
|  | D\_0\_\_Bacteria;D\_1\_\_Proteobacteria;D\_2\_\_Alphaproteobacteria;D\_3\_\_Rhizobiales;D\_4\_\_DUNssu044;Other | 0.0% | 0.0% | 0.0% | 0.0% | 0.0% | 0.0% | 0.0% | 0.0% | 0.0% |
|  | D\_0\_\_Bacteria;D\_1\_\_Proteobacteria;D\_2\_\_Alphaproteobacteria;D\_3\_\_Rhizobiales;D\_4\_\_FukuN57;D\_5\_\_uncultured bacterium | 0.0% | 0.0% | 0.0% | 0.0% | 0.0% | 0.0% | 0.0% | 0.0% | 0.0% |
|  | D\_0\_\_Bacteria;D\_1\_\_Proteobacteria;D\_2\_\_Alphaproteobacteria;D\_3\_\_Rhizobiales;D\_4\_\_Hyphomicrobiaceae;Ambiguous\_taxa | 0.0% | 0.0% | 0.0% | 0.0% | 0.0% | 0.0% | 0.0% | 0.0% | 0.0% |
|  | D\_0\_\_Bacteria;D\_1\_\_Proteobacteria;D\_2\_\_Alphaproteobacteria;D\_3\_\_Rhizobiales;D\_4\_\_Hyphomicrobiaceae;D\_5\_\_Devosia | 0.0% | 0.0% | 0.0% | 0.0% | 0.0% | 0.0% | 0.0% | 0.0% | 0.0% |
|  | D\_0\_\_Bacteria;D\_1\_\_Proteobacteria;D\_2\_\_Alphaproteobacteria;D\_3\_\_Rhizobiales;D\_4\_\_Hyphomicrobiaceae;D\_5\_\_Hyphomicrobium | 0.0% | 0.0% | 0.0% | 0.0% | 0.0% | 0.0% | 0.0% | 0.0% | 0.0% |
|  | D\_0\_\_Bacteria;D\_1\_\_Proteobacteria;D\_2\_\_Alphaproteobacteria;D\_3\_\_Rhizobiales;D\_4\_\_Hyphomicrobiaceae;D\_5\_\_Pedomicrobium | 0.1% | 0.0% | 0.0% | 0.1% | 0.1% | 0.2% | 0.2% | 0.0% | 0.0% |
|  | D\_0\_\_Bacteria;D\_1\_\_Proteobacteria;D\_2\_\_Alphaproteobacteria;D\_3\_\_Rhizobiales;D\_4\_\_Hyphomicrobiaceae;D\_5\_\_Rhodomicrobium | 0.0% | 0.0% | 0.0% | 0.0% | 0.0% | 0.0% | 0.0% | 0.0% | 0.0% |
|  | D\_0\_\_Bacteria;D\_1\_\_Proteobacteria;D\_2\_\_Alphaproteobacteria;D\_3\_\_Rhizobiales;D\_4\_\_Hyphomicrobiaceae;D\_5\_\_Rhodoplanes | 0.0% | 0.0% | 0.0% | 0.0% | 0.0% | 0.0% | 0.0% | 0.0% | 0.0% |
|  | D\_0\_\_Bacteria;D\_1\_\_Proteobacteria;D\_2\_\_Alphaproteobacteria;D\_3\_\_Rhizobiales;D\_4\_\_Hyphomicrobiaceae;D\_5\_\_uncultured | 0.0% | 0.0% | 0.0% | 0.0% | 0.0% | 0.0% | 0.0% | 0.0% | 0.0% |
|  | D\_0\_\_Bacteria;D\_1\_\_Proteobacteria;D\_2\_\_Alphaproteobacteria;D\_3\_\_Rhizobiales;D\_4\_\_Hyphomicrobiaceae;Other | 0.0% | 0.0% | 0.0% | 0.0% | 0.0% | 0.0% | 0.0% | 0.0% | 0.0% |
|  | D\_0\_\_Bacteria;D\_1\_\_Proteobacteria;D\_2\_\_Alphaproteobacteria;D\_3\_\_Rhizobiales;D\_4\_\_JG34-KF-361;Ambiguous\_taxa | 0.0% | 0.0% | 0.0% | 0.0% | 0.0% | 0.0% | 0.0% | 0.0% | 0.0% |
|  | D\_0\_\_Bacteria;D\_1\_\_Proteobacteria;D\_2\_\_Alphaproteobacteria;D\_3\_\_Rhizobiales;D\_4\_\_JG34-KF-361;D\_5\_\_uncultured bacterium | 0.0% | 0.0% | 0.0% | 0.0% | 0.0% | 0.0% | 0.0% | 0.0% | 0.0% |
|  | D\_0\_\_Bacteria;D\_1\_\_Proteobacteria;D\_2\_\_Alphaproteobacteria;D\_3\_\_Rhizobiales;D\_4\_\_JG34-KF-361;D\_5\_\_uncultured forest soil bacterium | 0.0% | 0.0% | 0.0% | 0.0% | 0.0% | 0.0% | 0.0% | 0.0% | 0.0% |
|  | D\_0\_\_Bacteria;D\_1\_\_Proteobacteria;D\_2\_\_Alphaproteobacteria;D\_3\_\_Rhizobiales;D\_4\_\_JG34-KF-361;Other | 0.0% | 0.0% | 0.0% | 0.0% | 0.0% | 0.0% | 0.0% | 0.0% | 0.0% |
|  | D\_0\_\_Bacteria;D\_1\_\_Proteobacteria;D\_2\_\_Alphaproteobacteria;D\_3\_\_Rhizobiales;D\_4\_\_KF-JG30-B3;D\_5\_\_uncultured bacterium | 0.0% | 0.0% | 0.0% | 0.0% | 0.0% | 0.0% | 0.0% | 0.0% | 0.0% |
|  | D\_0\_\_Bacteria;D\_1\_\_Proteobacteria;D\_2\_\_Alphaproteobacteria;D\_3\_\_Rhizobiales;D\_4\_\_MNG7;Other | 0.0% | 0.0% | 0.0% | 0.0% | 0.0% | 0.0% | 0.0% | 0.0% | 0.0% |
|  | D\_0\_\_Bacteria;D\_1\_\_Proteobacteria;D\_2\_\_Alphaproteobacteria;D\_3\_\_Rhizobiales;D\_4\_\_Methylobacteriaceae;D\_5\_\_Methylobacterium | 0.0% | 0.0% | 0.0% | 0.0% | 0.0% | 0.0% | 0.0% | 0.0% | 0.0% |
|  | D\_0\_\_Bacteria;D\_1\_\_Proteobacteria;D\_2\_\_Alphaproteobacteria;D\_3\_\_Rhizobiales;D\_4\_\_Methylobacteriaceae;D\_5\_\_Microvirga | 0.0% | 0.0% | 0.0% | 0.0% | 0.0% | 0.0% | 0.0% | 0.0% | 0.0% |
|  | D\_0\_\_Bacteria;D\_1\_\_Proteobacteria;D\_2\_\_Alphaproteobacteria;D\_3\_\_Rhizobiales;D\_4\_\_Methylobacteriaceae;D\_5\_\_Psychroglaciecola | 0.0% | 0.0% | 0.0% | 0.0% | 0.0% | 0.0% | 0.0% | 0.0% | 0.0% |
|  | D\_0\_\_Bacteria;D\_1\_\_Proteobacteria;D\_2\_\_Alphaproteobacteria;D\_3\_\_Rhizobiales;D\_4\_\_Methylobacteriaceae;D\_5\_\_uncultured | 0.0% | 0.0% | 0.0% | 0.0% | 0.0% | 0.0% | 0.0% | 0.0% | 0.0% |
|  | D\_0\_\_Bacteria;D\_1\_\_Proteobacteria;D\_2\_\_Alphaproteobacteria;D\_3\_\_Rhizobiales;D\_4\_\_Methylobacteriaceae;Other | 0.0% | 0.0% | 0.0% | 0.0% | 0.0% | 0.0% | 0.0% | 0.0% | 0.0% |
|  | D\_0\_\_Bacteria;D\_1\_\_Proteobacteria;D\_2\_\_Alphaproteobacteria;D\_3\_\_Rhizobiales;D\_4\_\_Methylocystaceae;D\_5\_\_Pleomorphomonas | 0.0% | 0.0% | 0.0% | 0.0% | 0.0% | 0.0% | 0.0% | 0.0% | 0.0% |
|  | D\_0\_\_Bacteria;D\_1\_\_Proteobacteria;D\_2\_\_Alphaproteobacteria;D\_3\_\_Rhizobiales;D\_4\_\_Methylocystaceae;Other | 0.0% | 0.0% | 0.0% | 0.0% | 0.0% | 0.0% | 0.0% | 0.0% | 0.0% |
|  | D\_0\_\_Bacteria;D\_1\_\_Proteobacteria;D\_2\_\_Alphaproteobacteria;D\_3\_\_Rhizobiales;D\_4\_\_Phyllobacteriaceae;D\_5\_\_Aliihoeflea | 0.0% | 0.0% | 0.0% | 0.0% | 0.0% | 0.0% | 0.0% | 0.0% | 0.0% |
|  | D\_0\_\_Bacteria;D\_1\_\_Proteobacteria;D\_2\_\_Alphaproteobacteria;D\_3\_\_Rhizobiales;D\_4\_\_Phyllobacteriaceae;D\_5\_\_Cohaesibacter | 0.0% | 0.0% | 0.0% | 0.0% | 0.0% | 0.0% | 0.0% | 0.0% | 0.0% |
|  | D\_0\_\_Bacteria;D\_1\_\_Proteobacteria;D\_2\_\_Alphaproteobacteria;D\_3\_\_Rhizobiales;D\_4\_\_Phyllobacteriaceae;D\_5\_\_Mesorhizobium | 0.1% | 0.1% | 0.1% | 0.1% | 0.1% | 0.1% | 0.0% | 0.0% | 0.1% |
|  | D\_0\_\_Bacteria;D\_1\_\_Proteobacteria;D\_2\_\_Alphaproteobacteria;D\_3\_\_Rhizobiales;D\_4\_\_Phyllobacteriaceae;D\_5\_\_Phyllobacterium | 0.0% | 0.0% | 0.0% | 0.0% | 0.0% | 0.0% | 0.0% | 0.0% | 0.0% |
|  | D\_0\_\_Bacteria;D\_1\_\_Proteobacteria;D\_2\_\_Alphaproteobacteria;D\_3\_\_Rhizobiales;D\_4\_\_Phyllobacteriaceae;Other | 0.0% | 0.0% | 0.0% | 0.0% | 0.0% | 0.0% | 0.0% | 0.0% | 0.0% |
|  | D\_0\_\_Bacteria;D\_1\_\_Proteobacteria;D\_2\_\_Alphaproteobacteria;D\_3\_\_Rhizobiales;D\_4\_\_Rhizobiaceae;D\_5\_\_Ensifer | 0.0% | 0.0% | 0.0% | 0.0% | 0.0% | 0.0% | 0.0% | 0.0% | 0.0% |
|  | D\_0\_\_Bacteria;D\_1\_\_Proteobacteria;D\_2\_\_Alphaproteobacteria;D\_3\_\_Rhizobiales;D\_4\_\_Rhizobiaceae;D\_5\_\_Kaistia | 0.0% | 0.0% | 0.0% | 0.0% | 0.0% | 0.0% | 0.0% | 0.0% | 0.0% |
|  | D\_0\_\_Bacteria;D\_1\_\_Proteobacteria;D\_2\_\_Alphaproteobacteria;D\_3\_\_Rhizobiales;D\_4\_\_Rhizobiaceae;D\_5\_\_Rhizobium | 0.0% | 0.0% | 0.0% | 0.0% | 0.0% | 0.0% | 0.0% | 0.0% | 0.3% |
|  | D\_0\_\_Bacteria;D\_1\_\_Proteobacteria;D\_2\_\_Alphaproteobacteria;D\_3\_\_Rhizobiales;D\_4\_\_Rhizobiaceae;D\_5\_\_Shinella | 0.0% | 0.0% | 0.0% | 0.0% | 0.0% | 0.0% | 0.0% | 0.0% | 0.0% |
|  | D\_0\_\_Bacteria;D\_1\_\_Proteobacteria;D\_2\_\_Alphaproteobacteria;D\_3\_\_Rhizobiales;D\_4\_\_Rhizobiaceae;Other | 0.0% | 0.0% | 0.0% | 0.0% | 0.0% | 0.0% | 0.0% | 0.0% | 0.0% |
|  | D\_0\_\_Bacteria;D\_1\_\_Proteobacteria;D\_2\_\_Alphaproteobacteria;D\_3\_\_Rhizobiales;D\_4\_\_Rhizobiales Incertae Sedis;D\_5\_\_Agaricicola | 0.0% | 0.0% | 0.0% | 0.0% | 0.0% | 0.0% | 0.0% | 0.0% | 0.1% |
|  | D\_0\_\_Bacteria;D\_1\_\_Proteobacteria;D\_2\_\_Alphaproteobacteria;D\_3\_\_Rhizobiales;D\_4\_\_Rhizobiales Incertae Sedis;D\_5\_\_Bauldia | 0.0% | 0.0% | 0.0% | 0.0% | 0.0% | 0.0% | 0.0% | 0.0% | 0.0% |
|  | D\_0\_\_Bacteria;D\_1\_\_Proteobacteria;D\_2\_\_Alphaproteobacteria;D\_3\_\_Rhizobiales;D\_4\_\_Rhizobiales Incertae Sedis;D\_5\_\_Phreatobacter | 0.0% | 0.0% | 0.0% | 0.0% | 0.0% | 0.0% | 0.0% | 0.0% | 0.0% |
|  | D\_0\_\_Bacteria;D\_1\_\_Proteobacteria;D\_2\_\_Alphaproteobacteria;D\_3\_\_Rhizobiales;D\_4\_\_Rhizobiales Incertae Sedis;D\_5\_\_Rhizomicrobium | 0.0% | 0.0% | 0.0% | 0.0% | 0.0% | 0.0% | 0.0% | 0.0% | 0.0% |
|  | D\_0\_\_Bacteria;D\_1\_\_Proteobacteria;D\_2\_\_Alphaproteobacteria;D\_3\_\_Rhizobiales;D\_4\_\_Rhodobiaceae;Ambiguous\_taxa | 0.0% | 0.0% | 0.0% | 0.0% | 0.0% | 0.0% | 0.0% | 0.0% | 0.0% |
|  | D\_0\_\_Bacteria;D\_1\_\_Proteobacteria;D\_2\_\_Alphaproteobacteria;D\_3\_\_Rhizobiales;D\_4\_\_Rhodobiaceae;D\_5\_\_Anderseniella | 0.0% | 0.0% | 0.0% | 0.0% | 0.0% | 0.0% | 0.0% | 0.0% | 0.0% |
|  | D\_0\_\_Bacteria;D\_1\_\_Proteobacteria;D\_2\_\_Alphaproteobacteria;D\_3\_\_Rhizobiales;D\_4\_\_Rhodobiaceae;D\_5\_\_Rhodobium | 0.0% | 0.0% | 0.0% | 0.0% | 0.0% | 0.0% | 0.0% | 0.0% | 0.0% |
|  | D\_0\_\_Bacteria;D\_1\_\_Proteobacteria;D\_2\_\_Alphaproteobacteria;D\_3\_\_Rhizobiales;D\_4\_\_Rhodobiaceae;D\_5\_\_uncultured | 0.0% | 0.0% | 0.0% | 0.0% | 0.0% | 0.0% | 0.0% | 0.0% | 0.0% |
|  | D\_0\_\_Bacteria;D\_1\_\_Proteobacteria;D\_2\_\_Alphaproteobacteria;D\_3\_\_Rhizobiales;D\_4\_\_Rhodobiaceae;Other | 0.1% | 0.1% | 0.1% | 0.1% | 0.1% | 0.0% | 0.0% | 0.0% | 0.0% |
|  | D\_0\_\_Bacteria;D\_1\_\_Proteobacteria;D\_2\_\_Alphaproteobacteria;D\_3\_\_Rhizobiales;D\_4\_\_Roseiarcaceae;D\_5\_\_Roseiarcus | 0.0% | 0.0% | 0.0% | 0.0% | 0.0% | 0.0% | 0.0% | 0.0% | 0.0% |
|  | D\_0\_\_Bacteria;D\_1\_\_Proteobacteria;D\_2\_\_Alphaproteobacteria;D\_3\_\_Rhizobiales;D\_4\_\_Xanthobacteraceae;D\_5\_\_Pseudolabrys | 0.0% | 0.0% | 0.0% | 0.0% | 0.0% | 0.0% | 0.0% | 0.0% | 0.0% |
|  | D\_0\_\_Bacteria;D\_1\_\_Proteobacteria;D\_2\_\_Alphaproteobacteria;D\_3\_\_Rhizobiales;D\_4\_\_Xanthobacteraceae;D\_5\_\_Variibacter | 0.5% | 0.2% | 0.1% | 0.3% | 0.3% | 1.0% | 1.8% | 0.1% | 0.1% |
|  | D\_0\_\_Bacteria;D\_1\_\_Proteobacteria;D\_2\_\_Alphaproteobacteria;D\_3\_\_Rhizobiales;D\_4\_\_Xanthobacteraceae;D\_5\_\_uncultured | 0.0% | 0.0% | 0.0% | 0.0% | 0.0% | 0.0% | 0.0% | 0.0% | 0.0% |
|  | D\_0\_\_Bacteria;D\_1\_\_Proteobacteria;D\_2\_\_Alphaproteobacteria;D\_3\_\_Rhizobiales;D\_4\_\_Xanthobacteraceae;Other | 0.7% | 1.0% | 0.9% | 0.7% | 0.7% | 0.7% | 0.4% | 0.3% | 0.7% |
|  | D\_0\_\_Bacteria;D\_1\_\_Proteobacteria;D\_2\_\_Alphaproteobacteria;D\_3\_\_Rhizobiales;D\_4\_\_alphaI cluster;Ambiguous\_taxa | 0.0% | 0.0% | 0.0% | 0.0% | 0.0% | 0.0% | 0.0% | 0.0% | 0.1% |
|  | D\_0\_\_Bacteria;D\_1\_\_Proteobacteria;D\_2\_\_Alphaproteobacteria;D\_3\_\_Rhizobiales;D\_4\_\_uncultured;Ambiguous\_taxa | 0.0% | 0.0% | 0.0% | 0.0% | 0.0% | 0.1% | 0.2% | 0.0% | 0.0% |
|  | D\_0\_\_Bacteria;D\_1\_\_Proteobacteria;D\_2\_\_Alphaproteobacteria;D\_3\_\_Rhizobiales;D\_4\_\_uncultured;D\_5\_\_uncultured alpha proteobacterium | 0.0% | 0.0% | 0.0% | 0.0% | 0.0% | 0.0% | 0.0% | 0.0% | 0.0% |
|  | D\_0\_\_Bacteria;D\_1\_\_Proteobacteria;D\_2\_\_Alphaproteobacteria;D\_3\_\_Rhizobiales;D\_4\_\_uncultured;D\_5\_\_uncultured bacterium | 0.0% | 0.0% | 0.0% | 0.0% | 0.0% | 0.2% | 0.1% | 0.0% | 0.0% |
|  | D\_0\_\_Bacteria;D\_1\_\_Proteobacteria;D\_2\_\_Alphaproteobacteria;D\_3\_\_Rhizobiales;D\_4\_\_uncultured;Other | 0.0% | 0.0% | 0.0% | 0.0% | 0.0% | 0.0% | 0.0% | 0.0% | 0.0% |
|  | D\_0\_\_Bacteria;D\_1\_\_Proteobacteria;D\_2\_\_Alphaproteobacteria;D\_3\_\_Rhizobiales;Other;Other | 0.0% | 0.0% | 0.0% | 0.1% | 0.1% | 0.0% | 0.0% | 0.0% | 0.0% |
|  | D\_0\_\_Bacteria;D\_1\_\_Proteobacteria;D\_2\_\_Alphaproteobacteria;D\_3\_\_Rhodobacterales;D\_4\_\_Rhodobacteraceae;D\_5\_\_Defluviimonas | 0.0% | 0.0% | 0.0% | 0.0% | 0.0% | 0.0% | 0.0% | 0.0% | 0.0% |
|  | D\_0\_\_Bacteria;D\_1\_\_Proteobacteria;D\_2\_\_Alphaproteobacteria;D\_3\_\_Rhodobacterales;D\_4\_\_Rhodobacteraceae;D\_5\_\_Labrenzia | 0.0% | 0.0% | 0.0% | 0.0% | 0.0% | 0.0% | 0.0% | 0.0% | 0.0% |
|  | D\_0\_\_Bacteria;D\_1\_\_Proteobacteria;D\_2\_\_Alphaproteobacteria;D\_3\_\_Rhodobacterales;D\_4\_\_Rhodobacteraceae;D\_5\_\_Paracoccus | 0.0% | 0.0% | 0.0% | 0.0% | 0.0% | 0.0% | 0.0% | 0.0% | 0.1% |
|  | D\_0\_\_Bacteria;D\_1\_\_Proteobacteria;D\_2\_\_Alphaproteobacteria;D\_3\_\_Rhodobacterales;D\_4\_\_Rhodobacteraceae;D\_5\_\_Rhodobacter | 0.0% | 0.0% | 0.0% | 0.0% | 0.0% | 0.0% | 0.0% | 0.0% | 0.0% |
|  | D\_0\_\_Bacteria;D\_1\_\_Proteobacteria;D\_2\_\_Alphaproteobacteria;D\_3\_\_Rhodobacterales;D\_4\_\_Rhodobacteraceae;D\_5\_\_Rubellimicrobium | 0.1% | 0.0% | 0.0% | 0.0% | 0.0% | 0.0% | 0.0% | 0.1% | 0.5% |
|  | D\_0\_\_Bacteria;D\_1\_\_Proteobacteria;D\_2\_\_Alphaproteobacteria;D\_3\_\_Rhodobacterales;D\_4\_\_Rhodobacteraceae;D\_5\_\_Thioclava | 0.0% | 0.0% | 0.0% | 0.0% | 0.0% | 0.0% | 0.0% | 0.0% | 0.0% |
|  | D\_0\_\_Bacteria;D\_1\_\_Proteobacteria;D\_2\_\_Alphaproteobacteria;D\_3\_\_Rhodobacterales;D\_4\_\_Rhodobacteraceae;D\_5\_\_Tropicimonas | 0.0% | 0.0% | 0.0% | 0.0% | 0.0% | 0.0% | 0.0% | 0.0% | 0.0% |
|  | D\_0\_\_Bacteria;D\_1\_\_Proteobacteria;D\_2\_\_Alphaproteobacteria;D\_3\_\_Rhodobacterales;D\_4\_\_Rhodobacteraceae;D\_5\_\_uncultured | 0.0% | 0.0% | 0.0% | 0.0% | 0.0% | 0.0% | 0.0% | 0.0% | 0.0% |
|  | D\_0\_\_Bacteria;D\_1\_\_Proteobacteria;D\_2\_\_Alphaproteobacteria;D\_3\_\_Rhodobacterales;D\_4\_\_Rhodobacteraceae;Other | 0.0% | 0.0% | 0.0% | 0.0% | 0.0% | 0.0% | 0.0% | 0.0% | 0.0% |
|  | D\_0\_\_Bacteria;D\_1\_\_Proteobacteria;D\_2\_\_Alphaproteobacteria;D\_3\_\_Rhodospirillales;D\_4\_\_AT-s3-44;Other | 0.0% | 0.0% | 0.0% | 0.0% | 0.0% | 0.0% | 0.0% | 0.0% | 0.0% |
|  | D\_0\_\_Bacteria;D\_1\_\_Proteobacteria;D\_2\_\_Alphaproteobacteria;D\_3\_\_Rhodospirillales;D\_4\_\_Acetobacteraceae;D\_5\_\_Acidiphilium | 0.0% | 0.0% | 0.0% | 0.0% | 0.0% | 0.0% | 0.0% | 0.0% | 0.0% |
|  | D\_0\_\_Bacteria;D\_1\_\_Proteobacteria;D\_2\_\_Alphaproteobacteria;D\_3\_\_Rhodospirillales;D\_4\_\_Acetobacteraceae;D\_5\_\_Craurococcus | 0.0% | 0.0% | 0.0% | 0.0% | 0.0% | 0.0% | 0.0% | 0.0% | 0.0% |
|  | D\_0\_\_Bacteria;D\_1\_\_Proteobacteria;D\_2\_\_Alphaproteobacteria;D\_3\_\_Rhodospirillales;D\_4\_\_Acetobacteraceae;D\_5\_\_Endobacter | 0.0% | 0.0% | 0.0% | 0.0% | 0.0% | 0.0% | 0.0% | 0.0% | 0.0% |
|  | D\_0\_\_Bacteria;D\_1\_\_Proteobacteria;D\_2\_\_Alphaproteobacteria;D\_3\_\_Rhodospirillales;D\_4\_\_Acetobacteraceae;D\_5\_\_Roseomonas | 0.0% | 0.0% | 0.0% | 0.0% | 0.0% | 0.0% | 0.0% | 0.1% | 0.0% |
|  | D\_0\_\_Bacteria;D\_1\_\_Proteobacteria;D\_2\_\_Alphaproteobacteria;D\_3\_\_Rhodospirillales;D\_4\_\_Acetobacteraceae;D\_5\_\_Rubritepida | 0.0% | 0.0% | 0.0% | 0.0% | 0.0% | 0.0% | 0.0% | 0.0% | 0.0% |
|  | D\_0\_\_Bacteria;D\_1\_\_Proteobacteria;D\_2\_\_Alphaproteobacteria;D\_3\_\_Rhodospirillales;D\_4\_\_Acetobacteraceae;D\_5\_\_uncultured | 0.0% | 0.1% | 0.1% | 0.1% | 0.1% | 0.0% | 0.0% | 0.0% | 0.0% |
|  | D\_0\_\_Bacteria;D\_1\_\_Proteobacteria;D\_2\_\_Alphaproteobacteria;D\_3\_\_Rhodospirillales;D\_4\_\_Acetobacteraceae;Other | 0.0% | 0.0% | 0.0% | 0.0% | 0.0% | 0.0% | 0.0% | 0.0% | 0.0% |
|  | D\_0\_\_Bacteria;D\_1\_\_Proteobacteria;D\_2\_\_Alphaproteobacteria;D\_3\_\_Rhodospirillales;D\_4\_\_DA111;D\_5\_\_uncultured alpha proteobacterium | 0.0% | 0.0% | 0.0% | 0.0% | 0.0% | 0.0% | 0.0% | 0.0% | 0.0% |
|  | D\_0\_\_Bacteria;D\_1\_\_Proteobacteria;D\_2\_\_Alphaproteobacteria;D\_3\_\_Rhodospirillales;D\_4\_\_DA111;D\_5\_\_uncultured bacterium | 0.0% | 0.0% | 0.0% | 0.0% | 0.0% | 0.0% | 0.0% | 0.0% | 0.0% |
|  | D\_0\_\_Bacteria;D\_1\_\_Proteobacteria;D\_2\_\_Alphaproteobacteria;D\_3\_\_Rhodospirillales;D\_4\_\_DA111;Other | 0.0% | 0.0% | 0.0% | 0.0% | 0.0% | 0.0% | 0.0% | 0.0% | 0.0% |
|  | D\_0\_\_Bacteria;D\_1\_\_Proteobacteria;D\_2\_\_Alphaproteobacteria;D\_3\_\_Rhodospirillales;D\_4\_\_KCM-B-15;D\_5\_\_uncultured Rhodospirillaceae bacterium | 0.0% | 0.0% | 0.0% | 0.0% | 0.0% | 0.0% | 0.0% | 0.0% | 0.0% |
|  | D\_0\_\_Bacteria;D\_1\_\_Proteobacteria;D\_2\_\_Alphaproteobacteria;D\_3\_\_Rhodospirillales;D\_4\_\_MND8;Other | 0.0% | 0.0% | 0.0% | 0.0% | 0.0% | 0.1% | 0.0% | 0.0% | 0.1% |
|  | D\_0\_\_Bacteria;D\_1\_\_Proteobacteria;D\_2\_\_Alphaproteobacteria;D\_3\_\_Rhodospirillales;D\_4\_\_MSB-1E8;D\_5\_\_uncultured bacterium | 0.0% | 0.0% | 0.0% | 0.0% | 0.0% | 0.0% | 0.0% | 0.0% | 0.0% |
|  | D\_0\_\_Bacteria;D\_1\_\_Proteobacteria;D\_2\_\_Alphaproteobacteria;D\_3\_\_Rhodospirillales;D\_4\_\_MSB-1E8;Other | 0.0% | 0.0% | 0.0% | 0.0% | 0.0% | 0.0% | 0.0% | 0.0% | 0.0% |
|  | D\_0\_\_Bacteria;D\_1\_\_Proteobacteria;D\_2\_\_Alphaproteobacteria;D\_3\_\_Rhodospirillales;D\_4\_\_Rhodospirillaceae;D\_5\_\_Defluviicoccus | 0.0% | 0.0% | 0.0% | 0.0% | 0.0% | 0.0% | 0.0% | 0.0% | 0.0% |
|  | D\_0\_\_Bacteria;D\_1\_\_Proteobacteria;D\_2\_\_Alphaproteobacteria;D\_3\_\_Rhodospirillales;D\_4\_\_Rhodospirillaceae;D\_5\_\_Magnetospira | 0.0% | 0.0% | 0.0% | 0.1% | 0.1% | 0.0% | 0.0% | 0.0% | 0.0% |
|  | D\_0\_\_Bacteria;D\_1\_\_Proteobacteria;D\_2\_\_Alphaproteobacteria;D\_3\_\_Rhodospirillales;D\_4\_\_Rhodospirillaceae;D\_5\_\_Nitrospirillum | 0.0% | 0.0% | 0.0% | 0.0% | 0.0% | 0.0% | 0.0% | 0.0% | 0.0% |
|  | D\_0\_\_Bacteria;D\_1\_\_Proteobacteria;D\_2\_\_Alphaproteobacteria;D\_3\_\_Rhodospirillales;D\_4\_\_Rhodospirillaceae;D\_5\_\_Niveispirillum | 0.0% | 0.0% | 0.0% | 0.0% | 0.0% | 0.0% | 0.0% | 0.0% | 0.0% |
|  | D\_0\_\_Bacteria;D\_1\_\_Proteobacteria;D\_2\_\_Alphaproteobacteria;D\_3\_\_Rhodospirillales;D\_4\_\_Rhodospirillaceae;D\_5\_\_Pelagibius | 0.8% | 1.8% | 1.7% | 1.1% | 1.1% | 0.4% | 0.1% | 0.0% | 0.1% |
|  | D\_0\_\_Bacteria;D\_1\_\_Proteobacteria;D\_2\_\_Alphaproteobacteria;D\_3\_\_Rhodospirillales;D\_4\_\_Rhodospirillaceae;D\_5\_\_Skermanella | 0.0% | 0.0% | 0.0% | 0.0% | 0.0% | 0.0% | 0.0% | 0.0% | 0.0% |
|  | D\_0\_\_Bacteria;D\_1\_\_Proteobacteria;D\_2\_\_Alphaproteobacteria;D\_3\_\_Rhodospirillales;D\_4\_\_Rhodospirillaceae;D\_5\_\_Thalassobaculum | 0.0% | 0.0% | 0.0% | 0.0% | 0.0% | 0.0% | 0.0% | 0.0% | 0.0% |
|  | D\_0\_\_Bacteria;D\_1\_\_Proteobacteria;D\_2\_\_Alphaproteobacteria;D\_3\_\_Rhodospirillales;D\_4\_\_Rhodospirillaceae;D\_5\_\_uncultured | 2.0% | 3.1% | 2.5% | 3.2% | 3.4% | 2.0% | 1.4% | 0.2% | 0.4% |
|  | D\_0\_\_Bacteria;D\_1\_\_Proteobacteria;D\_2\_\_Alphaproteobacteria;D\_3\_\_Rhodospirillales;D\_4\_\_Rhodospirillaceae;Other | 0.0% | 0.0% | 0.0% | 0.0% | 0.0% | 0.0% | 0.0% | 0.0% | 0.0% |
|  | D\_0\_\_Bacteria;D\_1\_\_Proteobacteria;D\_2\_\_Alphaproteobacteria;D\_3\_\_Rhodospirillales;D\_4\_\_Rhodospirillales Incertae Sedis;D\_5\_\_Candidatus Alysiosphaera | 0.0% | 0.0% | 0.0% | 0.0% | 0.0% | 0.0% | 0.0% | 0.0% | 0.0% |
|  | D\_0\_\_Bacteria;D\_1\_\_Proteobacteria;D\_2\_\_Alphaproteobacteria;D\_3\_\_Rhodospirillales;D\_4\_\_Rhodospirillales Incertae Sedis;D\_5\_\_Reyranella | 0.2% | 0.1% | 0.1% | 0.2% | 0.1% | 0.6% | 0.2% | 0.1% | 0.0% |
|  | D\_0\_\_Bacteria;D\_1\_\_Proteobacteria;D\_2\_\_Alphaproteobacteria;D\_3\_\_Rhodospirillales;Other;Other | 0.0% | 0.0% | 0.0% | 0.0% | 0.0% | 0.0% | 0.0% | 0.0% | 0.0% |
|  | D\_0\_\_Bacteria;D\_1\_\_Proteobacteria;D\_2\_\_Alphaproteobacteria;D\_3\_\_Rickettsiales;D\_4\_\_EF100-94H03;D\_5\_\_uncultured Parvibaculum sp. | 0.0% | 0.0% | 0.0% | 0.0% | 0.0% | 0.0% | 0.0% | 0.0% | 0.0% |
|  | D\_0\_\_Bacteria;D\_1\_\_Proteobacteria;D\_2\_\_Alphaproteobacteria;D\_3\_\_Rickettsiales;D\_4\_\_Mitochondria;Ambiguous\_taxa | 0.0% | 0.0% | 0.0% | 0.0% | 0.0% | 0.0% | 0.0% | 0.0% | 0.1% |
|  | D\_0\_\_Bacteria;D\_1\_\_Proteobacteria;D\_2\_\_Alphaproteobacteria;D\_3\_\_Rickettsiales;D\_4\_\_Mitochondria;Other | 0.0% | 0.0% | 0.0% | 0.0% | 0.0% | 0.0% | 0.0% | 0.0% | 0.0% |
|  | D\_0\_\_Bacteria;D\_1\_\_Proteobacteria;D\_2\_\_Alphaproteobacteria;D\_3\_\_Rickettsiales;D\_4\_\_Rickettsiaceae;D\_5\_\_Orientia | 0.0% | 0.0% | 0.0% | 0.0% | 0.0% | 0.0% | 0.0% | 0.0% | 0.0% |
|  | D\_0\_\_Bacteria;D\_1\_\_Proteobacteria;D\_2\_\_Alphaproteobacteria;D\_3\_\_Rickettsiales;D\_4\_\_SM2D12;D\_5\_\_uncultured bacterium | 0.0% | 0.0% | 0.0% | 0.0% | 0.0% | 0.0% | 0.0% | 0.0% | 0.0% |
|  | D\_0\_\_Bacteria;D\_1\_\_Proteobacteria;D\_2\_\_Alphaproteobacteria;D\_3\_\_Rickettsiales;D\_4\_\_TK34;Ambiguous\_taxa | 0.0% | 0.0% | 0.0% | 0.0% | 0.0% | 0.0% | 0.0% | 0.0% | 0.0% |
|  | D\_0\_\_Bacteria;D\_1\_\_Proteobacteria;D\_2\_\_Alphaproteobacteria;D\_3\_\_Sphingomonadales;D\_4\_\_7B-8;D\_5\_\_uncultured bacterium | 0.0% | 0.0% | 0.0% | 0.0% | 0.0% | 0.0% | 0.0% | 0.0% | 0.0% |
|  | D\_0\_\_Bacteria;D\_1\_\_Proteobacteria;D\_2\_\_Alphaproteobacteria;D\_3\_\_Sphingomonadales;D\_4\_\_Ellin6055;D\_5\_\_uncultured bacterium | 0.0% | 0.0% | 0.0% | 0.0% | 0.0% | 0.0% | 0.0% | 0.0% | 0.0% |
|  | D\_0\_\_Bacteria;D\_1\_\_Proteobacteria;D\_2\_\_Alphaproteobacteria;D\_3\_\_Sphingomonadales;D\_4\_\_Ellin6055;Other | 0.0% | 0.0% | 0.0% | 0.0% | 0.0% | 0.0% | 0.0% | 0.0% | 0.0% |
|  | D\_0\_\_Bacteria;D\_1\_\_Proteobacteria;D\_2\_\_Alphaproteobacteria;D\_3\_\_Sphingomonadales;D\_4\_\_Erythrobacteraceae;D\_5\_\_Altererythrobacter | 0.0% | 0.0% | 0.0% | 0.0% | 0.0% | 0.0% | 0.0% | 0.0% | 0.0% |
|  | D\_0\_\_Bacteria;D\_1\_\_Proteobacteria;D\_2\_\_Alphaproteobacteria;D\_3\_\_Sphingomonadales;D\_4\_\_Erythrobacteraceae;Other | 0.0% | 0.0% | 0.0% | 0.0% | 0.0% | 0.0% | 0.0% | 0.0% | 0.0% |
|  | D\_0\_\_Bacteria;D\_1\_\_Proteobacteria;D\_2\_\_Alphaproteobacteria;D\_3\_\_Sphingomonadales;D\_4\_\_Sphingomonadaceae;D\_5\_\_Novosphingobium | 0.0% | 0.0% | 0.0% | 0.0% | 0.0% | 0.0% | 0.0% | 0.0% | 0.0% |
|  | D\_0\_\_Bacteria;D\_1\_\_Proteobacteria;D\_2\_\_Alphaproteobacteria;D\_3\_\_Sphingomonadales;D\_4\_\_Sphingomonadaceae;D\_5\_\_Rhizorhapis | 0.0% | 0.0% | 0.0% | 0.0% | 0.0% | 0.0% | 0.0% | 0.0% | 0.0% |
|  | D\_0\_\_Bacteria;D\_1\_\_Proteobacteria;D\_2\_\_Alphaproteobacteria;D\_3\_\_Sphingomonadales;D\_4\_\_Sphingomonadaceae;D\_5\_\_Sphingomonas | 0.0% | 0.0% | 0.0% | 0.0% | 0.0% | 0.0% | 0.0% | 0.0% | 0.0% |
|  | D\_0\_\_Bacteria;D\_1\_\_Proteobacteria;D\_2\_\_Alphaproteobacteria;D\_3\_\_Sphingomonadales;D\_4\_\_Sphingomonadaceae;D\_5\_\_Sphingopyxis | 0.0% | 0.0% | 0.0% | 0.0% | 0.0% | 0.0% | 0.0% | 0.0% | 0.0% |
|  | D\_0\_\_Bacteria;D\_1\_\_Proteobacteria;D\_2\_\_Alphaproteobacteria;D\_3\_\_Sphingomonadales;D\_4\_\_Sphingomonadaceae;D\_5\_\_Zymomonas | 0.0% | 0.0% | 0.0% | 0.0% | 0.0% | 0.0% | 0.0% | 0.0% | 0.0% |
|  | D\_0\_\_Bacteria;D\_1\_\_Proteobacteria;D\_2\_\_Alphaproteobacteria;D\_3\_\_Sphingomonadales;D\_4\_\_Sphingomonadaceae;Other | 0.0% | 0.0% | 0.0% | 0.0% | 0.0% | 0.0% | 0.0% | 0.0% | 0.0% |
|  | D\_0\_\_Bacteria;D\_1\_\_Proteobacteria;D\_2\_\_Alphaproteobacteria;D\_3\_\_Sphingomonadales;Other;Other | 0.0% | 0.0% | 0.0% | 0.0% | 0.0% | 0.0% | 0.0% | 0.0% | 0.0% |
|  | D\_0\_\_Bacteria;D\_1\_\_Proteobacteria;D\_2\_\_Alphaproteobacteria;Other;Other;Other | 0.0% | 0.0% | 0.0% | 0.0% | 0.0% | 0.0% | 0.0% | 0.0% | 0.0% |
|  | D\_0\_\_Bacteria;D\_1\_\_Proteobacteria;D\_2\_\_Betaproteobacteria;D\_3\_\_Burkholderiales;D\_4\_\_Alcaligenaceae;D\_5\_\_Parasutterella | 0.0% | 0.0% | 0.0% | 0.0% | 0.0% | 0.0% | 0.0% | 0.0% | 0.0% |
|  | D\_0\_\_Bacteria;D\_1\_\_Proteobacteria;D\_2\_\_Betaproteobacteria;D\_3\_\_Burkholderiales;D\_4\_\_Burkholderiaceae;D\_5\_\_Burkholderia-Paraburkholderia | 0.0% | 0.0% | 0.0% | 0.0% | 0.0% | 0.0% | 0.0% | 0.0% | 0.0% |
|  | D\_0\_\_Bacteria;D\_1\_\_Proteobacteria;D\_2\_\_Betaproteobacteria;D\_3\_\_Burkholderiales;D\_4\_\_Burkholderiaceae;D\_5\_\_Cupriavidus | 0.0% | 0.0% | 0.0% | 0.0% | 0.0% | 0.0% | 0.0% | 0.0% | 0.0% |
|  | D\_0\_\_Bacteria;D\_1\_\_Proteobacteria;D\_2\_\_Betaproteobacteria;D\_3\_\_Burkholderiales;D\_4\_\_Burkholderiaceae;D\_5\_\_Lautropia | 0.0% | 0.0% | 0.0% | 0.0% | 0.0% | 0.0% | 0.0% | 0.0% | 0.1% |
|  | D\_0\_\_Bacteria;D\_1\_\_Proteobacteria;D\_2\_\_Betaproteobacteria;D\_3\_\_Burkholderiales;D\_4\_\_Burkholderiaceae;D\_5\_\_Limnobacter | 0.0% | 0.0% | 0.0% | 0.0% | 0.0% | 0.0% | 0.0% | 0.0% | 0.0% |
|  | D\_0\_\_Bacteria;D\_1\_\_Proteobacteria;D\_2\_\_Betaproteobacteria;D\_3\_\_Burkholderiales;D\_4\_\_Comamonadaceae;Ambiguous\_taxa | 0.0% | 0.0% | 0.0% | 0.0% | 0.0% | 0.0% | 0.0% | 0.0% | 0.0% |
|  | D\_0\_\_Bacteria;D\_1\_\_Proteobacteria;D\_2\_\_Betaproteobacteria;D\_3\_\_Burkholderiales;D\_4\_\_Comamonadaceae;D\_5\_\_Acidovorax | 0.0% | 0.0% | 0.0% | 0.0% | 0.0% | 0.0% | 0.0% | 0.0% | 0.0% |
|  | D\_0\_\_Bacteria;D\_1\_\_Proteobacteria;D\_2\_\_Betaproteobacteria;D\_3\_\_Burkholderiales;D\_4\_\_Comamonadaceae;D\_5\_\_Aquabacterium | 0.0% | 0.0% | 0.0% | 0.0% | 0.0% | 0.0% | 0.0% | 0.0% | 0.0% |
|  | D\_0\_\_Bacteria;D\_1\_\_Proteobacteria;D\_2\_\_Betaproteobacteria;D\_3\_\_Burkholderiales;D\_4\_\_Comamonadaceae;D\_5\_\_Comamonas | 0.0% | 0.0% | 0.0% | 0.0% | 0.0% | 0.0% | 0.0% | 0.0% | 0.0% |
|  | D\_0\_\_Bacteria;D\_1\_\_Proteobacteria;D\_2\_\_Betaproteobacteria;D\_3\_\_Burkholderiales;D\_4\_\_Comamonadaceae;D\_5\_\_Delftia | 0.0% | 0.0% | 0.0% | 0.0% | 0.0% | 0.0% | 0.0% | 0.0% | 0.3% |
|  | D\_0\_\_Bacteria;D\_1\_\_Proteobacteria;D\_2\_\_Betaproteobacteria;D\_3\_\_Burkholderiales;D\_4\_\_Comamonadaceae;D\_5\_\_Polaromonas | 0.0% | 0.0% | 0.0% | 0.0% | 0.0% | 0.0% | 0.0% | 0.0% | 0.0% |
|  | D\_0\_\_Bacteria;D\_1\_\_Proteobacteria;D\_2\_\_Betaproteobacteria;D\_3\_\_Burkholderiales;D\_4\_\_Comamonadaceae;D\_5\_\_Tepidimonas | 0.0% | 0.0% | 0.0% | 0.0% | 0.0% | 0.0% | 0.0% | 0.0% | 0.0% |
|  | D\_0\_\_Bacteria;D\_1\_\_Proteobacteria;D\_2\_\_Betaproteobacteria;D\_3\_\_Burkholderiales;D\_4\_\_Comamonadaceae;D\_5\_\_uncultured | 0.0% | 0.0% | 0.0% | 0.0% | 0.0% | 0.0% | 0.0% | 0.0% | 0.0% |
|  | D\_0\_\_Bacteria;D\_1\_\_Proteobacteria;D\_2\_\_Betaproteobacteria;D\_3\_\_Burkholderiales;D\_4\_\_Comamonadaceae;Other | 0.0% | 0.0% | 0.0% | 0.0% | 0.0% | 0.0% | 0.0% | 0.0% | 0.0% |
|  | D\_0\_\_Bacteria;D\_1\_\_Proteobacteria;D\_2\_\_Betaproteobacteria;D\_3\_\_Burkholderiales;D\_4\_\_Oxalobacteraceae;D\_5\_\_Herbaspirillum | 0.0% | 0.0% | 0.0% | 0.0% | 0.0% | 0.0% | 0.0% | 0.0% | 0.0% |
|  | D\_0\_\_Bacteria;D\_1\_\_Proteobacteria;D\_2\_\_Betaproteobacteria;D\_3\_\_Burkholderiales;D\_4\_\_Oxalobacteraceae;D\_5\_\_Massilia | 0.0% | 0.0% | 0.0% | 0.0% | 0.0% | 0.0% | 0.0% | 0.0% | 0.0% |
|  | D\_0\_\_Bacteria;D\_1\_\_Proteobacteria;D\_2\_\_Betaproteobacteria;D\_3\_\_Burkholderiales;D\_4\_\_Oxalobacteraceae;D\_5\_\_Noviherbaspirillum | 0.0% | 0.0% | 0.0% | 0.0% | 0.0% | 0.0% | 0.0% | 0.0% | 0.0% |
|  | D\_0\_\_Bacteria;D\_1\_\_Proteobacteria;D\_2\_\_Betaproteobacteria;D\_3\_\_Burkholderiales;D\_4\_\_Oxalobacteraceae;D\_5\_\_Undibacterium | 0.0% | 0.0% | 0.0% | 0.0% | 0.0% | 0.0% | 0.0% | 0.0% | 0.0% |
|  | D\_0\_\_Bacteria;D\_1\_\_Proteobacteria;D\_2\_\_Betaproteobacteria;D\_3\_\_Burkholderiales;D\_4\_\_Oxalobacteraceae;D\_5\_\_uncultured | 0.0% | 0.0% | 0.0% | 0.0% | 0.0% | 0.0% | 0.0% | 0.0% | 0.0% |
|  | D\_0\_\_Bacteria;D\_1\_\_Proteobacteria;D\_2\_\_Betaproteobacteria;D\_3\_\_Burkholderiales;D\_4\_\_Oxalobacteraceae;Other | 0.0% | 0.0% | 0.0% | 0.0% | 0.0% | 0.0% | 0.0% | 0.0% | 0.1% |
|  | D\_0\_\_Bacteria;D\_1\_\_Proteobacteria;D\_2\_\_Betaproteobacteria;D\_3\_\_Hydrogenophilales;D\_4\_\_Hydrogenophilaceae;D\_5\_\_Ferritrophicum | 0.0% | 0.0% | 0.0% | 0.0% | 0.0% | 0.0% | 0.0% | 0.0% | 0.0% |
|  | D\_0\_\_Bacteria;D\_1\_\_Proteobacteria;D\_2\_\_Betaproteobacteria;D\_3\_\_Hydrogenophilales;D\_4\_\_Hydrogenophilaceae;D\_5\_\_Hydrogenophilus | 0.0% | 0.0% | 0.0% | 0.0% | 0.0% | 0.0% | 0.0% | 0.0% | 0.0% |
|  | D\_0\_\_Bacteria;D\_1\_\_Proteobacteria;D\_2\_\_Betaproteobacteria;D\_3\_\_Hydrogenophilales;D\_4\_\_Hydrogenophilaceae;D\_5\_\_Thiobacillus | 0.0% | 0.0% | 0.0% | 0.0% | 0.0% | 0.3% | 0.1% | 0.0% | 0.0% |
|  | D\_0\_\_Bacteria;D\_1\_\_Proteobacteria;D\_2\_\_Betaproteobacteria;D\_3\_\_Methylophilales;D\_4\_\_Methylophilaceae;D\_5\_\_Methylotenera | 0.0% | 0.0% | 0.0% | 0.0% | 0.0% | 0.0% | 0.0% | 0.0% | 0.0% |
|  | D\_0\_\_Bacteria;D\_1\_\_Proteobacteria;D\_2\_\_Betaproteobacteria;D\_3\_\_Methylophilales;D\_4\_\_Methylophilaceae;D\_5\_\_OM43 clade | 0.0% | 0.0% | 0.0% | 0.0% | 0.0% | 0.0% | 0.0% | 0.0% | 0.0% |
|  | D\_0\_\_Bacteria;D\_1\_\_Proteobacteria;D\_2\_\_Betaproteobacteria;D\_3\_\_Methylophilales;D\_4\_\_Methylophilaceae;D\_5\_\_uncultured | 0.0% | 0.0% | 0.0% | 0.0% | 0.0% | 0.0% | 0.0% | 0.0% | 0.0% |
|  | D\_0\_\_Bacteria;D\_1\_\_Proteobacteria;D\_2\_\_Betaproteobacteria;D\_3\_\_Neisseriales;D\_4\_\_Neisseriaceae;D\_5\_\_Eikenella | 0.0% | 0.0% | 0.0% | 0.0% | 0.0% | 0.0% | 0.0% | 0.0% | 0.0% |
|  | D\_0\_\_Bacteria;D\_1\_\_Proteobacteria;D\_2\_\_Betaproteobacteria;D\_3\_\_Neisseriales;D\_4\_\_Neisseriaceae;D\_5\_\_Neisseria | 0.1% | 0.0% | 0.0% | 0.0% | 0.0% | 0.0% | 0.0% | 0.1% | 0.8% |
|  | D\_0\_\_Bacteria;D\_1\_\_Proteobacteria;D\_2\_\_Betaproteobacteria;D\_3\_\_Neisseriales;D\_4\_\_Neisseriaceae;D\_5\_\_uncultured | 0.0% | 0.0% | 0.0% | 0.0% | 0.0% | 0.0% | 0.0% | 0.0% | 0.0% |
|  | D\_0\_\_Bacteria;D\_1\_\_Proteobacteria;D\_2\_\_Betaproteobacteria;D\_3\_\_Neisseriales;D\_4\_\_Neisseriaceae;Other | 0.0% | 0.0% | 0.0% | 0.0% | 0.0% | 0.0% | 0.0% | 0.0% | 0.0% |
|  | D\_0\_\_Bacteria;D\_1\_\_Proteobacteria;D\_2\_\_Betaproteobacteria;D\_3\_\_Nitrosomonadales;D\_4\_\_Gallionellaceae;D\_5\_\_Candidatus Nitrotoga | 0.0% | 0.0% | 0.0% | 0.0% | 0.0% | 0.0% | 0.0% | 0.0% | 0.0% |
|  | D\_0\_\_Bacteria;D\_1\_\_Proteobacteria;D\_2\_\_Betaproteobacteria;D\_3\_\_Nitrosomonadales;D\_4\_\_Gallionellaceae;D\_5\_\_Gallionella | 0.0% | 0.0% | 0.0% | 0.0% | 0.0% | 0.0% | 0.0% | 0.0% | 0.0% |
|  | D\_0\_\_Bacteria;D\_1\_\_Proteobacteria;D\_2\_\_Betaproteobacteria;D\_3\_\_Nitrosomonadales;D\_4\_\_Gallionellaceae;D\_5\_\_Sideroxydans | 0.0% | 0.0% | 0.0% | 0.0% | 0.0% | 0.2% | 0.0% | 0.0% | 0.0% |
|  | D\_0\_\_Bacteria;D\_1\_\_Proteobacteria;D\_2\_\_Betaproteobacteria;D\_3\_\_Nitrosomonadales;D\_4\_\_Gallionellaceae;Other | 0.0% | 0.0% | 0.0% | 0.0% | 0.0% | 0.0% | 0.0% | 0.0% | 0.0% |
|  | D\_0\_\_Bacteria;D\_1\_\_Proteobacteria;D\_2\_\_Betaproteobacteria;D\_3\_\_Nitrosomonadales;D\_4\_\_Nitrosomonadaceae;D\_5\_\_Nitrosospira | 0.0% | 0.0% | 0.0% | 0.0% | 0.0% | 0.0% | 0.0% | 0.0% | 0.0% |
|  | D\_0\_\_Bacteria;D\_1\_\_Proteobacteria;D\_2\_\_Betaproteobacteria;D\_3\_\_Nitrosomonadales;D\_4\_\_Nitrosomonadaceae;D\_5\_\_uncultured | 0.0% | 0.0% | 0.0% | 0.0% | 0.0% | 0.0% | 0.0% | 0.0% | 0.0% |
|  | D\_0\_\_Bacteria;D\_1\_\_Proteobacteria;D\_2\_\_Betaproteobacteria;D\_3\_\_Nitrosomonadales;D\_4\_\_Nitrosomonadaceae;Other | 0.0% | 0.0% | 0.0% | 0.0% | 0.0% | 0.0% | 0.0% | 0.0% | 0.0% |
|  | D\_0\_\_Bacteria;D\_1\_\_Proteobacteria;D\_2\_\_Betaproteobacteria;D\_3\_\_Rhodocyclales;D\_4\_\_Rhodocyclaceae;D\_5\_\_Thauera | 0.0% | 0.0% | 0.0% | 0.0% | 0.0% | 0.0% | 0.0% | 0.0% | 0.0% |
|  | D\_0\_\_Bacteria;D\_1\_\_Proteobacteria;D\_2\_\_Betaproteobacteria;D\_3\_\_Rhodocyclales;D\_4\_\_Rhodocyclaceae;D\_5\_\_Zoogloea | 0.0% | 0.0% | 0.0% | 0.0% | 0.0% | 0.0% | 0.0% | 0.0% | 0.0% |
|  | D\_0\_\_Bacteria;D\_1\_\_Proteobacteria;D\_2\_\_Betaproteobacteria;D\_3\_\_Rhodocyclales;D\_4\_\_Rhodocyclaceae;Other | 0.0% | 0.0% | 0.0% | 0.0% | 0.0% | 0.0% | 0.0% | 0.0% | 0.0% |
|  | D\_0\_\_Bacteria;D\_1\_\_Proteobacteria;D\_2\_\_Betaproteobacteria;D\_3\_\_SC-I-84;D\_4\_\_uncultured bacterium;D\_5\_\_uncultured bacterium | 0.0% | 0.0% | 0.0% | 0.0% | 0.0% | 0.0% | 0.0% | 0.0% | 0.0% |
|  | D\_0\_\_Bacteria;D\_1\_\_Proteobacteria;D\_2\_\_Betaproteobacteria;D\_3\_\_TRA3-20;Ambiguous\_taxa;Ambiguous\_taxa | 0.0% | 0.0% | 0.0% | 0.0% | 0.0% | 0.0% | 0.0% | 0.0% | 0.0% |
|  | D\_0\_\_Bacteria;D\_1\_\_Proteobacteria;D\_2\_\_Betaproteobacteria;D\_3\_\_TRA3-20;D\_4\_\_uncultured bacterium;D\_5\_\_uncultured bacterium | 1.7% | 3.1% | 2.9% | 3.7% | 3.2% | 0.2% | 0.0% | 0.1% | 0.1% |
|  | D\_0\_\_Bacteria;D\_1\_\_Proteobacteria;D\_2\_\_Betaproteobacteria;D\_3\_\_TRA3-20;D\_4\_\_uncultured gamma proteobacterium;D\_5\_\_uncultured gamma proteobacterium | 0.0% | 0.0% | 0.0% | 0.0% | 0.0% | 0.0% | 0.0% | 0.0% | 0.0% |
|  | D\_0\_\_Bacteria;D\_1\_\_Proteobacteria;D\_2\_\_Betaproteobacteria;D\_3\_\_TRA3-20;Other;Other | 0.0% | 0.0% | 0.0% | 0.0% | 0.0% | 0.0% | 0.0% | 0.0% | 0.0% |
|  | D\_0\_\_Bacteria;D\_1\_\_Proteobacteria;D\_2\_\_Betaproteobacteria;Other;Other;Other | 0.0% | 0.0% | 0.0% | 0.0% | 0.0% | 0.0% | 0.0% | 0.0% | 0.0% |
|  | D\_0\_\_Bacteria;D\_1\_\_Proteobacteria;D\_2\_\_Deltaproteobacteria;D\_3\_\_Bdellovibrionales;D\_4\_\_Bacteriovoracaceae;D\_5\_\_Bacteriovorax | 0.0% | 0.0% | 0.0% | 0.0% | 0.0% | 0.0% | 0.0% | 0.0% | 0.0% |
|  | D\_0\_\_Bacteria;D\_1\_\_Proteobacteria;D\_2\_\_Deltaproteobacteria;D\_3\_\_Bdellovibrionales;D\_4\_\_Bacteriovoracaceae;D\_5\_\_Peredibacter | 0.0% | 0.0% | 0.0% | 0.0% | 0.0% | 0.0% | 0.0% | 0.0% | 0.0% |
|  | D\_0\_\_Bacteria;D\_1\_\_Proteobacteria;D\_2\_\_Deltaproteobacteria;D\_3\_\_Bdellovibrionales;D\_4\_\_Bdellovibrionaceae;D\_5\_\_Bdellovibrio | 0.0% | 0.0% | 0.0% | 0.0% | 0.0% | 0.0% | 0.0% | 0.0% | 0.0% |
|  | D\_0\_\_Bacteria;D\_1\_\_Proteobacteria;D\_2\_\_Deltaproteobacteria;D\_3\_\_Desulfurellales;D\_4\_\_Desulfurellaceae;D\_5\_\_G55 | 0.0% | 0.0% | 0.0% | 0.0% | 0.0% | 0.0% | 0.0% | 0.0% | 0.0% |
|  | D\_0\_\_Bacteria;D\_1\_\_Proteobacteria;D\_2\_\_Deltaproteobacteria;D\_3\_\_Desulfurellales;D\_4\_\_Desulfurellaceae;D\_5\_\_H16 | 4.2% | 8.1% | 7.9% | 7.0% | 7.9% | 1.1% | 0.6% | 0.3% | 0.6% |
|  | D\_0\_\_Bacteria;D\_1\_\_Proteobacteria;D\_2\_\_Deltaproteobacteria;D\_3\_\_Myxococcales;Ambiguous\_taxa;Other | 0.0% | 0.0% | 0.0% | 0.0% | 0.0% | 0.0% | 0.0% | 0.0% | 0.0% |
|  | D\_0\_\_Bacteria;D\_1\_\_Proteobacteria;D\_2\_\_Deltaproteobacteria;D\_3\_\_Myxococcales;D\_4\_\_Archangiaceae;D\_5\_\_Anaeromyxobacter | 0.0% | 0.0% | 0.0% | 0.0% | 0.0% | 0.0% | 0.0% | 0.0% | 0.0% |
|  | D\_0\_\_Bacteria;D\_1\_\_Proteobacteria;D\_2\_\_Deltaproteobacteria;D\_3\_\_Myxococcales;D\_4\_\_BIrii41;Ambiguous\_taxa | 0.0% | 0.0% | 0.0% | 0.0% | 0.0% | 0.0% | 0.0% | 0.0% | 0.0% |
|  | D\_0\_\_Bacteria;D\_1\_\_Proteobacteria;D\_2\_\_Deltaproteobacteria;D\_3\_\_Myxococcales;D\_4\_\_BIrii41;D\_5\_\_uncultured bacterium | 0.0% | 0.0% | 0.0% | 0.0% | 0.0% | 0.0% | 0.0% | 0.0% | 0.0% |
|  | D\_0\_\_Bacteria;D\_1\_\_Proteobacteria;D\_2\_\_Deltaproteobacteria;D\_3\_\_Myxococcales;D\_4\_\_Haliangiaceae;D\_5\_\_Haliangium | 0.0% | 0.0% | 0.0% | 0.0% | 0.0% | 0.0% | 0.0% | 0.0% | 0.0% |
|  | D\_0\_\_Bacteria;D\_1\_\_Proteobacteria;D\_2\_\_Deltaproteobacteria;D\_3\_\_Myxococcales;D\_4\_\_Nannocystaceae;D\_5\_\_Nannocystis | 0.0% | 0.0% | 0.0% | 0.0% | 0.0% | 0.0% | 0.0% | 0.0% | 0.0% |
|  | D\_0\_\_Bacteria;D\_1\_\_Proteobacteria;D\_2\_\_Deltaproteobacteria;D\_3\_\_Myxococcales;D\_4\_\_Nannocystaceae;D\_5\_\_uncultured | 0.0% | 0.0% | 0.0% | 0.0% | 0.0% | 0.0% | 0.0% | 0.0% | 0.0% |
|  | D\_0\_\_Bacteria;D\_1\_\_Proteobacteria;D\_2\_\_Deltaproteobacteria;D\_3\_\_Myxococcales;D\_4\_\_P3OB-42;D\_5\_\_uncultured bacterium | 0.0% | 0.0% | 0.0% | 0.0% | 0.0% | 0.0% | 0.0% | 0.0% | 0.0% |
|  | D\_0\_\_Bacteria;D\_1\_\_Proteobacteria;D\_2\_\_Deltaproteobacteria;D\_3\_\_Myxococcales;D\_4\_\_P3OB-42;Other | 0.0% | 0.0% | 0.0% | 0.0% | 0.0% | 0.0% | 0.0% | 0.0% | 0.0% |
|  | D\_0\_\_Bacteria;D\_1\_\_Proteobacteria;D\_2\_\_Deltaproteobacteria;D\_3\_\_Myxococcales;D\_4\_\_Phaselicystidaceae;D\_5\_\_Phaselicystis | 0.0% | 0.0% | 0.0% | 0.0% | 0.0% | 0.0% | 0.0% | 0.0% | 0.0% |
|  | D\_0\_\_Bacteria;D\_1\_\_Proteobacteria;D\_2\_\_Deltaproteobacteria;D\_3\_\_Myxococcales;D\_4\_\_Polyangiaceae;D\_5\_\_Sorangium | 0.0% | 0.0% | 0.0% | 0.0% | 0.0% | 0.0% | 0.0% | 0.0% | 0.0% |
|  | D\_0\_\_Bacteria;D\_1\_\_Proteobacteria;D\_2\_\_Deltaproteobacteria;D\_3\_\_Myxococcales;D\_4\_\_Polyangiaceae;D\_5\_\_uncultured | 0.0% | 0.0% | 0.0% | 0.0% | 0.0% | 0.0% | 0.0% | 0.0% | 0.0% |
|  | D\_0\_\_Bacteria;D\_1\_\_Proteobacteria;D\_2\_\_Deltaproteobacteria;D\_3\_\_Myxococcales;D\_4\_\_Polyangiaceae;Other | 0.0% | 0.0% | 0.0% | 0.0% | 0.0% | 0.0% | 0.0% | 0.0% | 0.0% |
|  | D\_0\_\_Bacteria;D\_1\_\_Proteobacteria;D\_2\_\_Deltaproteobacteria;D\_3\_\_Myxococcales;D\_4\_\_Sandaracinaceae;D\_5\_\_Sandaracinus | 0.0% | 0.0% | 0.0% | 0.0% | 0.0% | 0.0% | 0.0% | 0.0% | 0.0% |
|  | D\_0\_\_Bacteria;D\_1\_\_Proteobacteria;D\_2\_\_Deltaproteobacteria;D\_3\_\_Myxococcales;D\_4\_\_Sandaracinaceae;D\_5\_\_uncultured | 0.0% | 0.0% | 0.0% | 0.0% | 0.0% | 0.0% | 0.0% | 0.0% | 0.0% |
|  | D\_0\_\_Bacteria;D\_1\_\_Proteobacteria;D\_2\_\_Deltaproteobacteria;D\_3\_\_Myxococcales;D\_4\_\_VHS-B3-70;D\_5\_\_uncultured bacterium | 0.0% | 0.0% | 0.0% | 0.0% | 0.0% | 0.0% | 0.0% | 0.0% | 0.0% |
|  | D\_0\_\_Bacteria;D\_1\_\_Proteobacteria;D\_2\_\_Deltaproteobacteria;D\_3\_\_Myxococcales;D\_4\_\_mle1-27;D\_5\_\_uncultured bacterium | 0.0% | 0.0% | 0.0% | 0.0% | 0.0% | 0.0% | 0.0% | 0.0% | 0.0% |
|  | D\_0\_\_Bacteria;D\_1\_\_Proteobacteria;D\_2\_\_Deltaproteobacteria;D\_3\_\_Myxococcales;D\_4\_\_mle1-27;Other | 0.0% | 0.0% | 0.0% | 0.0% | 0.0% | 0.0% | 0.0% | 0.0% | 0.0% |
|  | D\_0\_\_Bacteria;D\_1\_\_Proteobacteria;D\_2\_\_Deltaproteobacteria;D\_3\_\_Myxococcales;D\_4\_\_uncultured;D\_5\_\_uncultured bacterium | 0.0% | 0.0% | 0.0% | 0.0% | 0.0% | 0.0% | 0.0% | 0.0% | 0.0% |
|  | D\_0\_\_Bacteria;D\_1\_\_Proteobacteria;D\_2\_\_Deltaproteobacteria;D\_3\_\_Myxococcales;D\_4\_\_uncultured bacterium;D\_5\_\_uncultured bacterium | 0.0% | 0.0% | 0.0% | 0.0% | 0.0% | 0.0% | 0.0% | 0.0% | 0.0% |
|  | D\_0\_\_Bacteria;D\_1\_\_Proteobacteria;D\_2\_\_Deltaproteobacteria;D\_3\_\_Myxococcales;Other;Other | 0.0% | 0.0% | 0.0% | 0.0% | 0.0% | 0.0% | 0.0% | 0.0% | 0.0% |
|  | D\_0\_\_Bacteria;D\_1\_\_Proteobacteria;D\_2\_\_Deltaproteobacteria;D\_3\_\_Oligoflexales;D\_4\_\_0319-6G20;D\_5\_\_uncultured bacterium | 0.0% | 0.0% | 0.0% | 0.0% | 0.0% | 0.0% | 0.0% | 0.0% | 0.0% |
|  | D\_0\_\_Bacteria;D\_1\_\_Proteobacteria;D\_2\_\_Deltaproteobacteria;D\_3\_\_Oligoflexales;D\_4\_\_0319-6G20;Other | 0.0% | 0.0% | 0.0% | 0.0% | 0.0% | 0.0% | 0.0% | 0.0% | 0.0% |
|  | D\_0\_\_Bacteria;D\_1\_\_Proteobacteria;D\_2\_\_Deltaproteobacteria;D\_3\_\_Oligoflexales;D\_4\_\_Oligoflexaceae;D\_5\_\_uncultured bacterium | 0.0% | 0.0% | 0.0% | 0.0% | 0.0% | 0.0% | 0.0% | 0.0% | 0.0% |
|  | D\_0\_\_Bacteria;D\_1\_\_Proteobacteria;D\_2\_\_Deltaproteobacteria;D\_3\_\_SAR324 clade(Marine group B);D\_4\_\_uncultured bacterium;D\_5\_\_uncultured bacterium | 0.0% | 0.0% | 0.0% | 0.0% | 0.0% | 0.0% | 0.0% | 0.0% | 0.0% |
|  | D\_0\_\_Bacteria;D\_1\_\_Proteobacteria;D\_2\_\_Deltaproteobacteria;D\_3\_\_SAR324 clade(Marine group B);D\_4\_\_uncultured delta proteobacterium;D\_5\_\_uncultured delta proteobacterium | 0.0% | 0.0% | 0.0% | 0.0% | 0.0% | 0.0% | 0.0% | 0.0% | 0.0% |
|  | D\_0\_\_Bacteria;D\_1\_\_Proteobacteria;D\_2\_\_Epsilonproteobacteria;D\_3\_\_Campylobacterales;D\_4\_\_Campylobacteraceae;D\_5\_\_Campylobacter | 0.0% | 0.0% | 0.0% | 0.0% | 0.0% | 0.0% | 0.0% | 0.0% | 0.0% |
|  | D\_0\_\_Bacteria;D\_1\_\_Proteobacteria;D\_2\_\_Gammaproteobacteria;D\_3\_\_Alteromonadales;D\_4\_\_Alteromonadaceae;D\_5\_\_Alishewanella | 0.0% | 0.0% | 0.0% | 0.0% | 0.0% | 0.0% | 0.0% | 0.0% | 0.0% |
|  | D\_0\_\_Bacteria;D\_1\_\_Proteobacteria;D\_2\_\_Gammaproteobacteria;D\_3\_\_Alteromonadales;D\_4\_\_Alteromonadaceae;D\_5\_\_Marinobacter | 0.0% | 0.0% | 0.0% | 0.0% | 0.0% | 0.0% | 0.0% | 0.0% | 0.0% |
|  | D\_0\_\_Bacteria;D\_1\_\_Proteobacteria;D\_2\_\_Gammaproteobacteria;D\_3\_\_Alteromonadales;D\_4\_\_Psychromonadaceae;D\_5\_\_Psychromonas | 0.0% | 0.0% | 0.0% | 0.0% | 0.0% | 0.0% | 0.0% | 0.0% | 0.0% |
|  | D\_0\_\_Bacteria;D\_1\_\_Proteobacteria;D\_2\_\_Gammaproteobacteria;D\_3\_\_Cardiobacteriales;D\_4\_\_Cardiobacteriaceae;D\_5\_\_Cardiobacterium | 0.0% | 0.0% | 0.0% | 0.0% | 0.0% | 0.0% | 0.0% | 0.0% | 0.0% |
|  | D\_0\_\_Bacteria;D\_1\_\_Proteobacteria;D\_2\_\_Gammaproteobacteria;D\_3\_\_Cellvibrionales;D\_4\_\_Cellvibrionaceae;D\_5\_\_Cellvibrio | 0.0% | 0.0% | 0.0% | 0.0% | 0.0% | 0.0% | 0.0% | 0.0% | 0.0% |
|  | D\_0\_\_Bacteria;D\_1\_\_Proteobacteria;D\_2\_\_Gammaproteobacteria;D\_3\_\_Chromatiales;D\_4\_\_Chromatiaceae;D\_5\_\_Rheinheimera | 0.0% | 0.0% | 0.0% | 0.0% | 0.0% | 0.0% | 0.0% | 0.0% | 0.0% |
|  | D\_0\_\_Bacteria;D\_1\_\_Proteobacteria;D\_2\_\_Gammaproteobacteria;D\_3\_\_Chromatiales;D\_4\_\_Chromatiaceae;D\_5\_\_uncultured | 0.0% | 0.0% | 0.0% | 0.0% | 0.0% | 0.0% | 0.0% | 0.0% | 0.0% |
|  | D\_0\_\_Bacteria;D\_1\_\_Proteobacteria;D\_2\_\_Gammaproteobacteria;D\_3\_\_Chromatiales;D\_4\_\_Ectothiorhodospiraceae;D\_5\_\_Thioalkalispira | 0.0% | 0.0% | 0.0% | 0.0% | 0.0% | 0.0% | 0.0% | 0.0% | 0.0% |
|  | D\_0\_\_Bacteria;D\_1\_\_Proteobacteria;D\_2\_\_Gammaproteobacteria;D\_3\_\_Chromatiales;D\_4\_\_Ectothiorhodospiraceae;D\_5\_\_Thiogranum | 0.0% | 0.0% | 0.0% | 0.0% | 0.0% | 0.0% | 0.0% | 0.0% | 0.0% |
|  | D\_0\_\_Bacteria;D\_1\_\_Proteobacteria;D\_2\_\_Gammaproteobacteria;D\_3\_\_Chromatiales;D\_4\_\_Ectothiorhodospiraceae;Other | 0.0% | 0.0% | 0.0% | 0.0% | 0.0% | 0.0% | 0.0% | 0.0% | 0.0% |
|  | D\_0\_\_Bacteria;D\_1\_\_Proteobacteria;D\_2\_\_Gammaproteobacteria;D\_3\_\_Chromatiales;Other;Other | 0.0% | 0.0% | 0.0% | 0.0% | 0.0% | 0.0% | 0.0% | 0.0% | 0.0% |
|  | D\_0\_\_Bacteria;D\_1\_\_Proteobacteria;D\_2\_\_Gammaproteobacteria;D\_3\_\_Enterobacteriales;D\_4\_\_Enterobacteriaceae;Ambiguous\_taxa | 0.0% | 0.0% | 0.0% | 0.0% | 0.0% | 0.0% | 0.0% | 0.0% | 0.0% |
|  | D\_0\_\_Bacteria;D\_1\_\_Proteobacteria;D\_2\_\_Gammaproteobacteria;D\_3\_\_Enterobacteriales;D\_4\_\_Enterobacteriaceae;D\_5\_\_Candidatus Blochmannia | 0.0% | 0.0% | 0.0% | 0.0% | 0.0% | 0.0% | 0.0% | 0.0% | 0.0% |
|  | D\_0\_\_Bacteria;D\_1\_\_Proteobacteria;D\_2\_\_Gammaproteobacteria;D\_3\_\_Enterobacteriales;D\_4\_\_Enterobacteriaceae;D\_5\_\_Escherichia-Shigella | 0.0% | 0.0% | 0.0% | 0.0% | 0.0% | 0.1% | 0.0% | 0.0% | 0.1% |
|  | D\_0\_\_Bacteria;D\_1\_\_Proteobacteria;D\_2\_\_Gammaproteobacteria;D\_3\_\_Gammaproteobacteria Incertae Sedis;D\_4\_\_Unknown Family;D\_5\_\_uncultured gamma proteobacterium | 0.0% | 0.0% | 0.0% | 0.0% | 0.0% | 0.0% | 0.0% | 0.0% | 0.0% |
|  | D\_0\_\_Bacteria;D\_1\_\_Proteobacteria;D\_2\_\_Gammaproteobacteria;D\_3\_\_HTA4;D\_4\_\_uncultured bacterium;D\_5\_\_uncultured bacterium | 0.0% | 0.0% | 0.0% | 0.0% | 0.0% | 0.0% | 0.0% | 0.0% | 0.0% |
|  | D\_0\_\_Bacteria;D\_1\_\_Proteobacteria;D\_2\_\_Gammaproteobacteria;D\_3\_\_KI89A clade;D\_4\_\_uncultured bacterium;D\_5\_\_uncultured bacterium | 0.0% | 0.0% | 0.0% | 0.0% | 0.0% | 0.0% | 0.0% | 0.0% | 0.0% |
|  | D\_0\_\_Bacteria;D\_1\_\_Proteobacteria;D\_2\_\_Gammaproteobacteria;D\_3\_\_Legionellales;D\_4\_\_Coxiellaceae;D\_5\_\_Aquicella | 0.0% | 0.0% | 0.0% | 0.0% | 0.0% | 0.0% | 0.0% | 0.0% | 0.0% |
|  | D\_0\_\_Bacteria;D\_1\_\_Proteobacteria;D\_2\_\_Gammaproteobacteria;D\_3\_\_Legionellales;D\_4\_\_Coxiellaceae;D\_5\_\_Coxiella | 0.0% | 0.0% | 0.0% | 0.0% | 0.0% | 0.0% | 0.0% | 0.0% | 0.0% |
|  | D\_0\_\_Bacteria;D\_1\_\_Proteobacteria;D\_2\_\_Gammaproteobacteria;D\_3\_\_Legionellales;D\_4\_\_Coxiellaceae;D\_5\_\_Diplorickettsia | 0.0% | 0.0% | 0.0% | 0.0% | 0.0% | 0.0% | 0.0% | 0.0% | 0.0% |
|  | D\_0\_\_Bacteria;D\_1\_\_Proteobacteria;D\_2\_\_Gammaproteobacteria;D\_3\_\_Legionellales;D\_4\_\_Coxiellaceae;D\_5\_\_uncultured | 0.0% | 0.0% | 0.0% | 0.0% | 0.0% | 0.0% | 0.0% | 0.0% | 0.0% |
|  | D\_0\_\_Bacteria;D\_1\_\_Proteobacteria;D\_2\_\_Gammaproteobacteria;D\_3\_\_Legionellales;D\_4\_\_Legionellaceae;D\_5\_\_Legionella | 0.0% | 0.0% | 0.0% | 0.0% | 0.0% | 0.0% | 0.0% | 0.0% | 0.0% |
|  | D\_0\_\_Bacteria;D\_1\_\_Proteobacteria;D\_2\_\_Gammaproteobacteria;D\_3\_\_Oceanospirillales;D\_4\_\_Halomonadaceae;D\_5\_\_Halomonas | 0.0% | 0.0% | 0.0% | 0.0% | 0.0% | 0.0% | 0.0% | 0.0% | 0.0% |
|  | D\_0\_\_Bacteria;D\_1\_\_Proteobacteria;D\_2\_\_Gammaproteobacteria;D\_3\_\_Oceanospirillales;D\_4\_\_Oceanospirillaceae;D\_5\_\_Pseudohongiella | 0.0% | 0.0% | 0.0% | 0.0% | 0.0% | 0.0% | 0.0% | 0.0% | 0.0% |
|  | D\_0\_\_Bacteria;D\_1\_\_Proteobacteria;D\_2\_\_Gammaproteobacteria;D\_3\_\_PYR10d3;Other;Other | 0.0% | 0.0% | 0.0% | 0.0% | 0.0% | 0.0% | 0.0% | 0.0% | 0.0% |
|  | D\_0\_\_Bacteria;D\_1\_\_Proteobacteria;D\_2\_\_Gammaproteobacteria;D\_3\_\_Pasteurellales;D\_4\_\_Pasteurellaceae;D\_5\_\_Aggregatibacter | 0.0% | 0.0% | 0.0% | 0.0% | 0.0% | 0.0% | 0.0% | 0.0% | 0.0% |
|  | D\_0\_\_Bacteria;D\_1\_\_Proteobacteria;D\_2\_\_Gammaproteobacteria;D\_3\_\_Pasteurellales;D\_4\_\_Pasteurellaceae;D\_5\_\_Haemophilus | 0.1% | 0.0% | 0.0% | 0.0% | 0.0% | 0.0% | 0.0% | 0.0% | 0.5% |
|  | D\_0\_\_Bacteria;D\_1\_\_Proteobacteria;D\_2\_\_Gammaproteobacteria;D\_3\_\_Pasteurellales;D\_4\_\_Pasteurellaceae;Other | 0.0% | 0.0% | 0.0% | 0.0% | 0.0% | 0.0% | 0.0% | 0.0% | 0.0% |
|  | D\_0\_\_Bacteria;D\_1\_\_Proteobacteria;D\_2\_\_Gammaproteobacteria;D\_3\_\_Pseudomonadales;D\_4\_\_Moraxellaceae;D\_5\_\_Acinetobacter | 0.0% | 0.0% | 0.0% | 0.0% | 0.0% | 0.0% | 0.0% | 0.0% | 0.1% |
|  | D\_0\_\_Bacteria;D\_1\_\_Proteobacteria;D\_2\_\_Gammaproteobacteria;D\_3\_\_Pseudomonadales;D\_4\_\_Moraxellaceae;D\_5\_\_Enhydrobacter | 0.0% | 0.0% | 0.0% | 0.0% | 0.0% | 0.0% | 0.0% | 0.0% | 0.2% |
|  | D\_0\_\_Bacteria;D\_1\_\_Proteobacteria;D\_2\_\_Gammaproteobacteria;D\_3\_\_Pseudomonadales;D\_4\_\_Moraxellaceae;D\_5\_\_Moraxella | 0.0% | 0.0% | 0.0% | 0.0% | 0.0% | 0.0% | 0.0% | 0.0% | 0.0% |
|  | D\_0\_\_Bacteria;D\_1\_\_Proteobacteria;D\_2\_\_Gammaproteobacteria;D\_3\_\_Pseudomonadales;D\_4\_\_Moraxellaceae;D\_5\_\_Psychrobacter | 0.0% | 0.0% | 0.0% | 0.0% | 0.0% | 0.0% | 0.0% | 0.0% | 0.0% |
|  | D\_0\_\_Bacteria;D\_1\_\_Proteobacteria;D\_2\_\_Gammaproteobacteria;D\_3\_\_Pseudomonadales;D\_4\_\_Moraxellaceae;D\_5\_\_uncultured | 0.0% | 0.0% | 0.0% | 0.0% | 0.0% | 0.0% | 0.0% | 0.0% | 0.0% |
|  | D\_0\_\_Bacteria;D\_1\_\_Proteobacteria;D\_2\_\_Gammaproteobacteria;D\_3\_\_Pseudomonadales;D\_4\_\_Pseudomonadaceae;D\_5\_\_Pseudomonas | 0.1% | 0.0% | 0.0% | 0.0% | 0.0% | 0.0% | 0.0% | 0.0% | 0.5% |
|  | D\_0\_\_Bacteria;D\_1\_\_Proteobacteria;D\_2\_\_Gammaproteobacteria;D\_3\_\_Thiotrichales;D\_4\_\_Piscirickettsiaceae;D\_5\_\_uncultured bacterium | 0.0% | 0.0% | 0.0% | 0.0% | 0.0% | 0.0% | 0.0% | 0.0% | 0.0% |
|  | D\_0\_\_Bacteria;D\_1\_\_Proteobacteria;D\_2\_\_Gammaproteobacteria;D\_3\_\_Thiotrichales;D\_4\_\_Thiotrichaceae;D\_5\_\_uncultured | 0.0% | 0.0% | 0.0% | 0.0% | 0.0% | 0.0% | 0.0% | 0.0% | 0.0% |
|  | D\_0\_\_Bacteria;D\_1\_\_Proteobacteria;D\_2\_\_Gammaproteobacteria;D\_3\_\_Vibrionales;D\_4\_\_Vibrionaceae;D\_5\_\_Vibrio | 0.0% | 0.0% | 0.0% | 0.0% | 0.0% | 0.0% | 0.0% | 0.0% | 0.0% |
|  | D\_0\_\_Bacteria;D\_1\_\_Proteobacteria;D\_2\_\_Gammaproteobacteria;D\_3\_\_X35;Other;Other | 0.0% | 0.0% | 0.0% | 0.0% | 0.0% | 0.0% | 0.0% | 0.0% | 0.0% |
|  | D\_0\_\_Bacteria;D\_1\_\_Proteobacteria;D\_2\_\_Gammaproteobacteria;D\_3\_\_Xanthomonadales;D\_4\_\_JTB255 marine benthic group;Ambiguous\_taxa | 0.0% | 0.0% | 0.0% | 0.1% | 0.1% | 0.1% | 0.0% | 0.0% | 0.0% |
|  | D\_0\_\_Bacteria;D\_1\_\_Proteobacteria;D\_2\_\_Gammaproteobacteria;D\_3\_\_Xanthomonadales;D\_4\_\_JTB255 marine benthic group;D\_5\_\_uncultured bacterium | 0.0% | 0.0% | 0.0% | 0.0% | 0.0% | 0.0% | 0.0% | 0.0% | 0.0% |
|  | D\_0\_\_Bacteria;D\_1\_\_Proteobacteria;D\_2\_\_Gammaproteobacteria;D\_3\_\_Xanthomonadales;D\_4\_\_JTB255 marine benthic group;Other | 0.0% | 0.0% | 0.0% | 0.0% | 0.0% | 0.0% | 0.0% | 0.0% | 0.0% |
|  | D\_0\_\_Bacteria;D\_1\_\_Proteobacteria;D\_2\_\_Gammaproteobacteria;D\_3\_\_Xanthomonadales;D\_4\_\_Nevskiaceae;D\_5\_\_Nevskia | 0.0% | 0.0% | 0.0% | 0.0% | 0.0% | 0.0% | 0.0% | 0.0% | 0.0% |
|  | D\_0\_\_Bacteria;D\_1\_\_Proteobacteria;D\_2\_\_Gammaproteobacteria;D\_3\_\_Xanthomonadales;D\_4\_\_Xanthomonadaceae;D\_5\_\_Arenimonas | 0.0% | 0.0% | 0.0% | 0.0% | 0.0% | 0.0% | 0.0% | 0.0% | 0.0% |
|  | D\_0\_\_Bacteria;D\_1\_\_Proteobacteria;D\_2\_\_Gammaproteobacteria;D\_3\_\_Xanthomonadales;D\_4\_\_Xanthomonadaceae;D\_5\_\_Luteimonas | 0.0% | 0.0% | 0.0% | 0.0% | 0.0% | 0.0% | 0.0% | 0.0% | 0.0% |
|  | D\_0\_\_Bacteria;D\_1\_\_Proteobacteria;D\_2\_\_Gammaproteobacteria;D\_3\_\_Xanthomonadales;D\_4\_\_Xanthomonadaceae;D\_5\_\_Lysobacter | 0.0% | 0.0% | 0.0% | 0.0% | 0.0% | 0.0% | 0.0% | 0.0% | 0.0% |
|  | D\_0\_\_Bacteria;D\_1\_\_Proteobacteria;D\_2\_\_Gammaproteobacteria;D\_3\_\_Xanthomonadales;D\_4\_\_Xanthomonadaceae;D\_5\_\_Pseudofulvimonas | 0.0% | 0.0% | 0.0% | 0.0% | 0.0% | 0.0% | 0.0% | 0.0% | 0.0% |
|  | D\_0\_\_Bacteria;D\_1\_\_Proteobacteria;D\_2\_\_Gammaproteobacteria;D\_3\_\_Xanthomonadales;D\_4\_\_Xanthomonadaceae;D\_5\_\_Pseudoxanthomonas | 0.0% | 0.0% | 0.0% | 0.0% | 0.0% | 0.0% | 0.0% | 0.0% | 0.0% |
|  | D\_0\_\_Bacteria;D\_1\_\_Proteobacteria;D\_2\_\_Gammaproteobacteria;D\_3\_\_Xanthomonadales;D\_4\_\_Xanthomonadaceae;D\_5\_\_Stenotrophomonas | 0.0% | 0.0% | 0.0% | 0.0% | 0.0% | 0.0% | 0.0% | 0.0% | 0.2% |
|  | D\_0\_\_Bacteria;D\_1\_\_Proteobacteria;D\_2\_\_Gammaproteobacteria;D\_3\_\_Xanthomonadales;D\_4\_\_Xanthomonadaceae;D\_5\_\_Thermomonas | 0.0% | 0.0% | 0.0% | 0.0% | 0.0% | 0.0% | 0.0% | 0.0% | 0.0% |
|  | D\_0\_\_Bacteria;D\_1\_\_Proteobacteria;D\_2\_\_Gammaproteobacteria;D\_3\_\_Xanthomonadales;D\_4\_\_Xanthomonadaceae;D\_5\_\_uncultured | 0.0% | 0.0% | 0.0% | 0.0% | 0.0% | 0.0% | 0.0% | 0.0% | 0.0% |
|  | D\_0\_\_Bacteria;D\_1\_\_Proteobacteria;D\_2\_\_Gammaproteobacteria;D\_3\_\_Xanthomonadales;D\_4\_\_Xanthomonadaceae;Other | 0.0% | 0.0% | 0.0% | 0.0% | 0.0% | 0.0% | 0.0% | 0.0% | 0.0% |
|  | D\_0\_\_Bacteria;D\_1\_\_Proteobacteria;D\_2\_\_Gammaproteobacteria;D\_3\_\_Xanthomonadales;D\_4\_\_Xanthomonadales Incertae Sedis;D\_5\_\_Acidibacter | 0.0% | 0.0% | 0.0% | 0.0% | 0.0% | 0.0% | 0.0% | 0.0% | 0.0% |
|  | D\_0\_\_Bacteria;D\_1\_\_Proteobacteria;D\_2\_\_Gammaproteobacteria;D\_3\_\_Xanthomonadales;D\_4\_\_Xanthomonadales Incertae Sedis;D\_5\_\_Steroidobacter | 0.0% | 0.0% | 0.0% | 0.0% | 0.0% | 0.0% | 0.0% | 0.0% | 0.0% |
|  | D\_0\_\_Bacteria;D\_1\_\_Proteobacteria;D\_2\_\_Gammaproteobacteria;D\_3\_\_Xanthomonadales;D\_4\_\_uncultured;D\_5\_\_uncultured bacterium | 0.0% | 0.0% | 0.0% | 0.0% | 0.0% | 0.0% | 0.0% | 0.0% | 0.0% |
|  | D\_0\_\_Bacteria;D\_1\_\_Proteobacteria;D\_2\_\_Gammaproteobacteria;D\_3\_\_Xanthomonadales;Other;Other | 0.0% | 0.0% | 0.0% | 0.0% | 0.0% | 0.0% | 0.0% | 0.0% | 0.0% |
|  | D\_0\_\_Bacteria;D\_1\_\_Proteobacteria;D\_2\_\_Gammaproteobacteria;D\_3\_\_uncultured;D\_4\_\_uncultured bacterium;D\_5\_\_uncultured bacterium | 0.0% | 0.0% | 0.0% | 0.0% | 0.0% | 0.0% | 0.0% | 0.0% | 0.0% |
|  | D\_0\_\_Bacteria;D\_1\_\_Proteobacteria;D\_2\_\_Gammaproteobacteria;D\_3\_\_uncultured;Other;Other | 0.0% | 0.0% | 0.0% | 0.0% | 0.0% | 0.0% | 0.0% | 0.0% | 0.0% |
|  | D\_0\_\_Bacteria;D\_1\_\_Proteobacteria;D\_2\_\_Gammaproteobacteria;Other;Other;Other | 0.1% | 0.0% | 0.0% | 0.0% | 0.0% | 0.0% | 0.0% | 0.1% | 0.9% |
|  | D\_0\_\_Bacteria;D\_1\_\_Proteobacteria;D\_2\_\_JTB23;D\_3\_\_uncultured bacterium;D\_4\_\_uncultured bacterium;D\_5\_\_uncultured bacterium | 0.0% | 0.0% | 0.0% | 0.0% | 0.0% | 0.0% | 0.0% | 0.0% | 0.0% |
|  | D\_0\_\_Bacteria;D\_1\_\_Proteobacteria;D\_2\_\_JTB23;Other;Other;Other | 0.0% | 0.0% | 0.0% | 0.0% | 0.0% | 0.0% | 0.0% | 0.0% | 0.0% |
|  | D\_0\_\_Bacteria;D\_1\_\_Proteobacteria;D\_2\_\_SPOTSOCT00m83;Ambiguous\_taxa;Ambiguous\_taxa;Ambiguous\_taxa | 0.0% | 0.0% | 0.0% | 0.0% | 0.0% | 0.0% | 0.0% | 0.0% | 0.0% |
|  | D\_0\_\_Bacteria;D\_1\_\_Proteobacteria;D\_2\_\_SPOTSOCT00m83;Other;Other;Other | 0.0% | 0.0% | 0.0% | 0.0% | 0.0% | 0.0% | 0.0% | 0.0% | 0.0% |
|  | D\_0\_\_Bacteria;D\_1\_\_Proteobacteria;Other;Other;Other;Other | 0.0% | 0.0% | 0.0% | 0.0% | 0.0% | 0.0% | 0.0% | 0.0% | 0.0% |
|  | D\_0\_\_Bacteria;D\_1\_\_SBR1093;Other;Other;Other;Other | 0.0% | 0.0% | 0.0% | 0.0% | 0.0% | 0.0% | 0.0% | 0.0% | 0.0% |
|  | D\_0\_\_Bacteria;D\_1\_\_SR1 (Absconditabacteria);Ambiguous\_taxa;Ambiguous\_taxa;Ambiguous\_taxa;Ambiguous\_taxa | 0.0% | 0.0% | 0.0% | 0.0% | 0.0% | 0.0% | 0.0% | 0.0% | 0.0% |
|  | D\_0\_\_Bacteria;D\_1\_\_SR1 (Absconditabacteria);D\_2\_\_uncultured bacterium;D\_3\_\_uncultured bacterium;D\_4\_\_uncultured bacterium;D\_5\_\_uncultured bacterium | 0.0% | 0.0% | 0.0% | 0.0% | 0.0% | 0.0% | 0.0% | 0.0% | 0.1% |
|  | D\_0\_\_Bacteria;D\_1\_\_Saccharibacteria;D\_2\_\_uncultured bacterium;D\_3\_\_uncultured bacterium;D\_4\_\_uncultured bacterium;D\_5\_\_uncultured bacterium | 0.0% | 0.0% | 0.0% | 0.0% | 0.0% | 0.0% | 0.0% | 0.0% | 0.0% |
|  | D\_0\_\_Bacteria;D\_1\_\_Saccharibacteria;Other;Other;Other;Other | 0.0% | 0.0% | 0.0% | 0.0% | 0.0% | 0.0% | 0.0% | 0.0% | 0.0% |
|  | D\_0\_\_Bacteria;D\_1\_\_Spirochaetae;D\_2\_\_Spirochaetes;D\_3\_\_Spirochaetales;D\_4\_\_Spirochaetaceae;D\_5\_\_Treponema 2 | 0.0% | 0.0% | 0.0% | 0.0% | 0.0% | 0.0% | 0.0% | 0.0% | 0.1% |
|  | D\_0\_\_Bacteria;D\_1\_\_Synergistetes;D\_2\_\_Synergistia;D\_3\_\_Synergistales;D\_4\_\_Synergistaceae;D\_5\_\_Fretibacterium | 0.0% | 0.0% | 0.0% | 0.0% | 0.0% | 0.0% | 0.0% | 0.0% | 0.0% |
|  | D\_0\_\_Bacteria;D\_1\_\_Synergistetes;D\_2\_\_Synergistia;D\_3\_\_Synergistales;D\_4\_\_Synergistaceae;D\_5\_\_uncultured | 0.0% | 0.0% | 0.0% | 0.0% | 0.0% | 0.0% | 0.0% | 0.0% | 0.0% |
|  | D\_0\_\_Bacteria;D\_1\_\_TM6 (Dependentiae);Other;Other;Other;Other | 0.0% | 0.0% | 0.0% | 0.0% | 0.0% | 0.0% | 0.0% | 0.0% | 0.0% |
|  | D\_0\_\_Bacteria;D\_1\_\_Tectomicrobia;D\_2\_\_Tectomicrobia Incertae Sedis;D\_3\_\_Unknown Order;D\_4\_\_Unknown Family;D\_5\_\_Candidatus Entotheonella | 0.0% | 0.0% | 0.0% | 0.0% | 0.0% | 0.0% | 0.0% | 0.0% | 0.0% |
|  | D\_0\_\_Bacteria;D\_1\_\_Tectomicrobia;D\_2\_\_uncultured bacterium;D\_3\_\_uncultured bacterium;D\_4\_\_uncultured bacterium;D\_5\_\_uncultured bacterium | 0.0% | 0.0% | 0.0% | 0.0% | 0.0% | 0.0% | 0.0% | 0.0% | 0.0% |
|  | D\_0\_\_Bacteria;D\_1\_\_Verrucomicrobia;D\_2\_\_OPB35 soil group;Ambiguous\_taxa;Ambiguous\_taxa;Ambiguous\_taxa | 0.0% | 0.0% | 0.0% | 0.0% | 0.0% | 0.0% | 0.0% | 0.0% | 0.0% |
|  | D\_0\_\_Bacteria;D\_1\_\_Verrucomicrobia;D\_2\_\_OPB35 soil group;D\_3\_\_uncultured bacterium;D\_4\_\_uncultured bacterium;D\_5\_\_uncultured bacterium | 0.0% | 0.0% | 0.0% | 0.0% | 0.0% | 0.0% | 0.0% | 0.0% | 0.0% |
|  | D\_0\_\_Bacteria;D\_1\_\_Verrucomicrobia;D\_2\_\_OPB35 soil group;Other;Other;Other | 0.0% | 0.0% | 0.0% | 0.0% | 0.0% | 0.0% | 0.0% | 0.0% | 0.0% |
|  | D\_0\_\_Bacteria;D\_1\_\_Verrucomicrobia;D\_2\_\_Opitutae;D\_3\_\_BC-COM435;D\_4\_\_uncultured bacterium;D\_5\_\_uncultured bacterium | 0.0% | 0.0% | 0.0% | 0.0% | 0.0% | 0.0% | 0.0% | 0.0% | 0.0% |
|  | D\_0\_\_Bacteria;D\_1\_\_Verrucomicrobia;D\_2\_\_Opitutae;D\_3\_\_Opitutales;D\_4\_\_Opitutaceae;D\_5\_\_Opitutus | 0.0% | 0.0% | 0.0% | 0.0% | 0.0% | 0.0% | 0.0% | 0.0% | 0.0% |
|  | D\_0\_\_Bacteria;D\_1\_\_Verrucomicrobia;D\_2\_\_Spartobacteria;D\_3\_\_Chthoniobacterales;D\_4\_\_Chthoniobacteraceae;D\_5\_\_Chthoniobacter | 0.0% | 0.0% | 0.0% | 0.0% | 0.0% | 0.0% | 0.0% | 0.0% | 0.0% |
|  | D\_0\_\_Bacteria;D\_1\_\_Verrucomicrobia;D\_2\_\_Spartobacteria;D\_3\_\_Chthoniobacterales;D\_4\_\_DA101 soil group;Ambiguous\_taxa | 0.0% | 0.0% | 0.0% | 0.0% | 0.0% | 0.0% | 0.0% | 0.0% | 0.0% |
|  | D\_0\_\_Bacteria;D\_1\_\_Verrucomicrobia;D\_2\_\_Spartobacteria;D\_3\_\_Chthoniobacterales;D\_4\_\_DA101 soil group;D\_5\_\_uncultured bacterium | 0.0% | 0.0% | 0.0% | 0.0% | 0.0% | 0.0% | 0.0% | 0.0% | 0.0% |
|  | D\_0\_\_Bacteria;D\_1\_\_Verrucomicrobia;D\_2\_\_Spartobacteria;D\_3\_\_Chthoniobacterales;D\_4\_\_Xiphinematobacteraceae;D\_5\_\_Candidatus Xiphinematobacter | 0.0% | 0.0% | 0.0% | 0.0% | 0.0% | 0.0% | 0.0% | 0.0% | 0.0% |
|  | D\_0\_\_Bacteria;D\_1\_\_Verrucomicrobia;D\_2\_\_Spartobacteria;D\_3\_\_Chthoniobacterales;Other;Other | 0.0% | 0.0% | 0.0% | 0.0% | 0.0% | 0.0% | 0.0% | 0.0% | 0.0% |
|  | D\_0\_\_Bacteria;D\_1\_\_Verrucomicrobia;D\_2\_\_Verrucomicrobiae;D\_3\_\_Verrucomicrobiales;D\_4\_\_DEV007;D\_5\_\_uncultured bacterium | 0.0% | 0.0% | 0.0% | 0.0% | 0.0% | 0.0% | 0.0% | 0.0% | 0.0% |
|  | D\_0\_\_Bacteria;D\_1\_\_Verrucomicrobia;D\_2\_\_Verrucomicrobiae;D\_3\_\_Verrucomicrobiales;D\_4\_\_Verrucomicrobiaceae;Ambiguous\_taxa | 0.0% | 0.0% | 0.0% | 0.0% | 0.0% | 0.0% | 0.0% | 0.0% | 0.0% |
|  | D\_0\_\_Bacteria;D\_1\_\_Verrucomicrobia;D\_2\_\_Verrucomicrobiae;D\_3\_\_Verrucomicrobiales;D\_4\_\_Verrucomicrobiaceae;D\_5\_\_Prosthecobacter | 0.0% | 0.0% | 0.0% | 0.0% | 0.0% | 0.0% | 0.0% | 0.0% | 0.0% |
|  | D\_0\_\_Bacteria;D\_1\_\_Verrucomicrobia;D\_2\_\_Verrucomicrobiae;D\_3\_\_Verrucomicrobiales;D\_4\_\_Verrucomicrobiaceae;D\_5\_\_uncultured | 0.0% | 0.0% | 0.0% | 0.0% | 0.0% | 0.0% | 0.0% | 0.0% | 0.0% |
|  | D\_0\_\_Bacteria;D\_1\_\_Verrucomicrobia;D\_2\_\_Verrucomicrobiae;D\_3\_\_Verrucomicrobiales;D\_4\_\_Verrucomicrobiaceae;Other | 0.0% | 0.0% | 0.0% | 0.0% | 0.0% | 0.0% | 0.0% | 0.0% | 0.0% |
|  | D\_0\_\_Bacteria;Other;Other;Other;Other;Other | 0.0% | 0.0% | 0.0% | 0.0% | 0.0% | 0.0% | 0.0% | 0.0% | 0.0% |
|  | Unassigned;Other;Other;Other;Other;Other | 0.3% | 0.4% | 0.4% | 0.3% | 0.3% | 0.4% | 0.1% | 0.2% | 0.2% |
