# Supplementary material for: Cretaceous dinosaur bone contains recent organic material and provides an environment conducive to microbial communities
Source: eLife. 2019 Jun 18;8:e46205. doi: 10.7554/eLife.46205 (PMC6581507; doi:10.7554/eLife.46205)
Supplement: Source data 1. [file elife-46205-data1.zip › Raw data files/16S rRNA amplicon sequencing/arare_max620000/alpha_rarefaction_plots/rarefaction_plots.html]

Rarefaction Curves


|  |  |  |  |
| --- | --- | --- | --- |
| **Select a Metric:** | PD\_whole\_tree chao1 observed\_otus | **Select a Category:** | BarcodeSequence Description LinkerPrimerSequence SampleID well |

  

**Show Categories:
 
All
None
Invert**

**Legend**

|  |  |  |  |
| --- | --- | --- | --- |
| ▶ |  | ■ | **AGGCAGAAAGAGTAGA** |
| ∟ |  | ◆ | **1M2** |
| ▶ |  | ■ | **AGGCAGAATATCCTCT** |
| ∟ |  | ◆ | **1M1** |
| ▶ |  | ■ | **CGTACTAGAGAGTAGA** |
| ∟ |  | ◆ | **1S2** |
| ▶ |  | ■ | **CGTACTAGTATCCTCT** |
| ∟ |  | ◆ | **1S1** |
| ▶ |  | ■ | **GTAGAGGAAGAGTAGA** |
| ∟ |  | ◆ | **1B5g2** |
| ▶ |  | ■ | **GTAGAGGATATCCTCT** |
| ∟ |  | ◆ | **1B5g1** |
| ▶ |  | ■ | **TCCTGAGCAGAGTAGA** |
| ∟ |  | ◆ | **1BEDTA2** |
| ▶ |  | ■ | **TCCTGAGCTATCCTCT** |
| ∟ |  | ◆ | **1BEDTA1** |
| ▶ |  | ■ | **1B5g\_1** |
| ∟ |  | ◆ | **1B5g1** |
| ▶ |  | ■ | **1B5g\_2** |
| ∟ |  | ◆ | **1B5g2** |
| ▶ |  | ■ | **1BEDTA\_1** |
| ∟ |  | ◆ | **1BEDTA1** |
| ▶ |  | ■ | **1BEDTA\_2** |
| ∟ |  | ◆ | **1BEDTA2** |
| ▶ |  | ■ | **1Mudstone\_1** |
| ∟ |  | ◆ | **1M1** |
| ▶ |  | ■ | **1Mudstone\_2** |
| ∟ |  | ◆ | **1M2** |
| ▶ |  | ■ | **1scrappings\_1** |
| ∟ |  | ◆ | **1S1** |
| ▶ |  | ■ | **1scrappings\_2** |
| ∟ |  | ◆ | **1S2** |
| ▶ |  | ■ | **CCGTAAAACGACGGCCAG** |
| ∟ |  | ◆ | **1B5g1** |
| ∟ |  | ◆ | **1B5g2** |
| ∟ |  | ◆ | **1BEDTA1** |
| ∟ |  | ◆ | **1BEDTA2** |
| ∟ |  | ◆ | **1M1** |
| ∟ |  | ◆ | **1M2** |
| ∟ |  | ◆ | **1S1** |
| ∟ |  | ◆ | **1S2** |
| ▶ |  | ■ | **1B5g1** |
| ∟ |  | ◆ | **1B5g1** |
| ▶ |  | ■ | **1B5g2** |
| ∟ |  | ◆ | **1B5g2** |
| ▶ |  | ■ | **1BEDTA1** |
| ∟ |  | ◆ | **1BEDTA1** |
| ▶ |  | ■ | **1BEDTA2** |
| ∟ |  | ◆ | **1BEDTA2** |
| ▶ |  | ■ | **1M1** |
| ∟ |  | ◆ | **1M1** |
| ▶ |  | ■ | **1M2** |
| ∟ |  | ◆ | **1M2** |
| ▶ |  | ■ | **1S1** |
| ∟ |  | ◆ | **1S1** |
| ▶ |  | ■ | **1S2** |
| ∟ |  | ◆ | **1S2** |
| ▶ |  | ■ | **1B5g** |
| ∟ |  | ◆ | **1B5g1** |
| ∟ |  | ◆ | **1B5g2** |
| ▶ |  | ■ | **1BEDTA** |
| ∟ |  | ◆ | **1BEDTA1** |
| ∟ |  | ◆ | **1BEDTA2** |
| ▶ |  | ■ | **1Mudstone** |
| ∟ |  | ◆ | **1M1** |
| ∟ |  | ◆ | **1M2** |
| ▶ |  | ■ | **1Scrappings** |
| ∟ |  | ◆ | **1S1** |
| ∟ |  | ◆ | **1S2** |
| ▶ |  | ■ | **AGGCAGAAAGAGTAGA** |
| ∟ |  | ◆ | **1M2** |
| ▶ |  | ■ | **AGGCAGAATATCCTCT** |
| ∟ |  | ◆ | **1M1** |
| ▶ |  | ■ | **CGTACTAGAGAGTAGA** |
| ∟ |  | ◆ | **1S2** |
| ▶ |  | ■ | **CGTACTAGTATCCTCT** |
| ∟ |  | ◆ | **1S1** |
| ▶ |  | ■ | **GTAGAGGAAGAGTAGA** |
| ∟ |  | ◆ | **1B5g2** |
| ▶ |  | ■ | **GTAGAGGATATCCTCT** |
| ∟ |  | ◆ | **1B5g1** |
| ▶ |  | ■ | **TCCTGAGCAGAGTAGA** |
| ∟ |  | ◆ | **1BEDTA2** |
| ▶ |  | ■ | **TCCTGAGCTATCCTCT** |
| ∟ |  | ◆ | **1BEDTA1** |
| ▶ |  | ■ | **1B5g\_1** |
| ∟ |  | ◆ | **1B5g1** |
| ▶ |  | ■ | **1B5g\_2** |
| ∟ |  | ◆ | **1B5g2** |
| ▶ |  | ■ | **1BEDTA\_1** |
| ∟ |  | ◆ | **1BEDTA1** |
| ▶ |  | ■ | **1BEDTA\_2** |
| ∟ |  | ◆ | **1BEDTA2** |
| ▶ |  | ■ | **1Mudstone\_1** |
| ∟ |  | ◆ | **1M1** |
| ▶ |  | ■ | **1Mudstone\_2** |
| ∟ |  | ◆ | **1M2** |
| ▶ |  | ■ | **1scrappings\_1** |
| ∟ |  | ◆ | **1S1** |
| ▶ |  | ■ | **1scrappings\_2** |
| ∟ |  | ◆ | **1S2** |
| ▶ |  | ■ | **CCGTAAAACGACGGCCAG** |
| ∟ |  | ◆ | **1B5g1** |
| ∟ |  | ◆ | **1B5g2** |
| ∟ |  | ◆ | **1BEDTA1** |
| ∟ |  | ◆ | **1BEDTA2** |
| ∟ |  | ◆ | **1M1** |
| ∟ |  | ◆ | **1M2** |
| ∟ |  | ◆ | **1S1** |
| ∟ |  | ◆ | **1S2** |
| ▶ |  | ■ | **1B5g1** |
| ∟ |  | ◆ | **1B5g1** |
| ▶ |  | ■ | **1B5g2** |
| ∟ |  | ◆ | **1B5g2** |
| ▶ |  | ■ | **1BEDTA1** |
| ∟ |  | ◆ | **1BEDTA1** |
| ▶ |  | ■ | **1BEDTA2** |
| ∟ |  | ◆ | **1BEDTA2** |
| ▶ |  | ■ | **1M1** |
| ∟ |  | ◆ | **1M1** |
| ▶ |  | ■ | **1M2** |
| ∟ |  | ◆ | **1M2** |
| ▶ |  | ■ | **1S1** |
| ∟ |  | ◆ | **1S1** |
| ▶ |  | ■ | **1S2** |
| ∟ |  | ◆ | **1S2** |
| ▶ |  | ■ | **1B5g** |
| ∟ |  | ◆ | **1B5g1** |
| ∟ |  | ◆ | **1B5g2** |
| ▶ |  | ■ | **1BEDTA** |
| ∟ |  | ◆ | **1BEDTA1** |
| ∟ |  | ◆ | **1BEDTA2** |
| ▶ |  | ■ | **1Mudstone** |
| ∟ |  | ◆ | **1M1** |
| ∟ |  | ◆ | **1M2** |
| ▶ |  | ■ | **1Scrappings** |
| ∟ |  | ◆ | **1S1** |
| ∟ |  | ◆ | **1S2** |
| ▶ |  | ■ | **AGGCAGAAAGAGTAGA** |
| ∟ |  | ◆ | **1M2** |
| ▶ |  | ■ | **AGGCAGAATATCCTCT** |
| ∟ |  | ◆ | **1M1** |
| ▶ |  | ■ | **CGTACTAGAGAGTAGA** |
| ∟ |  | ◆ | **1S2** |
| ▶ |  | ■ | **CGTACTAGTATCCTCT** |
| ∟ |  | ◆ | **1S1** |
| ▶ |  | ■ | **GTAGAGGAAGAGTAGA** |
| ∟ |  | ◆ | **1B5g2** |
| ▶ |  | ■ | **GTAGAGGATATCCTCT** |
| ∟ |  | ◆ | **1B5g1** |
| ▶ |  | ■ | **TCCTGAGCAGAGTAGA** |
| ∟ |  | ◆ | **1BEDTA2** |
| ▶ |  | ■ | **TCCTGAGCTATCCTCT** |
| ∟ |  | ◆ | **1BEDTA1** |
| ▶ |  | ■ | **1B5g\_1** |
| ∟ |  | ◆ | **1B5g1** |
| ▶ |  | ■ | **1B5g\_2** |
| ∟ |  | ◆ | **1B5g2** |
| ▶ |  | ■ | **1BEDTA\_1** |
| ∟ |  | ◆ | **1BEDTA1** |
| ▶ |  | ■ | **1BEDTA\_2** |
| ∟ |  | ◆ | **1BEDTA2** |
| ▶ |  | ■ | **1Mudstone\_1** |
| ∟ |  | ◆ | **1M1** |
| ▶ |  | ■ | **1Mudstone\_2** |
| ∟ |  | ◆ | **1M2** |
| ▶ |  | ■ | **1scrappings\_1** |
| ∟ |  | ◆ | **1S1** |
| ▶ |  | ■ | **1scrappings\_2** |
| ∟ |  | ◆ | **1S2** |
| ▶ |  | ■ | **CCGTAAAACGACGGCCAG** |
| ∟ |  | ◆ | **1B5g1** |
| ∟ |  | ◆ | **1B5g2** |
| ∟ |  | ◆ | **1BEDTA1** |
| ∟ |  | ◆ | **1BEDTA2** |
| ∟ |  | ◆ | **1M1** |
| ∟ |  | ◆ | **1M2** |
| ∟ |  | ◆ | **1S1** |
| ∟ |  | ◆ | **1S2** |
| ▶ |  | ■ | **1B5g1** |
| ∟ |  | ◆ | **1B5g1** |
| ▶ |  | ■ | **1B5g2** |
| ∟ |  | ◆ | **1B5g2** |
| ▶ |  | ■ | **1BEDTA1** |
| ∟ |  | ◆ | **1BEDTA1** |
| ▶ |  | ■ | **1BEDTA2** |
| ∟ |  | ◆ | **1BEDTA2** |
| ▶ |  | ■ | **1M1** |
| ∟ |  | ◆ | **1M1** |
| ▶ |  | ■ | **1M2** |
| ∟ |  | ◆ | **1M2** |
| ▶ |  | ■ | **1S1** |
| ∟ |  | ◆ | **1S1** |
| ▶ |  | ■ | **1S2** |
| ∟ |  | ◆ | **1S2** |
| ▶ |  | ■ | **1B5g** |
| ∟ |  | ◆ | **1B5g1** |
| ∟ |  | ◆ | **1B5g2** |
| ▶ |  | ■ | **1BEDTA** |
| ∟ |  | ◆ | **1BEDTA1** |
| ∟ |  | ◆ | **1BEDTA2** |
| ▶ |  | ■ | **1Mudstone** |
| ∟ |  | ◆ | **1M1** |
| ∟ |  | ◆ | **1M2** |
| ▶ |  | ■ | **1Scrappings** |
| ∟ |  | ◆ | **1S1** |
| ∟ |  | ◆ | **1S2** |

**If the lines for some categories do not extend all the way to the right end of the x-axis, that means that at least one of the samples in that category does not have that many sequences.**

  
  

|  |  |  |  |  |  |  |  |
| --- | --- | --- | --- | --- | --- | --- | --- |
| well | Seqs/Sample | PD\_whole\_tree Ave. | PD\_whole\_tree Err. | chao1 Ave. | chao1 Err. | observed\_otus Ave. | observed\_otus Err. |
| 1B5g | 10.0 | 1.425 | 0.175 | 17.100 | 0.350 | 7.600 | 0.400 || 1B5g | 62009.0 | 64.494 | 2.157 | 2701.843 | 191.509 | 1115.700 | 65.400 || 1B5g | 124008.0 | 83.259 | 2.003 | 4077.060 | 237.084 | 1678.100 | 75.700 || 1B5g | 186007.0 | 97.512 | 2.979 | 5214.975 | 230.794 | 2134.600 | 104.400 || 1B5g | 248006.0 | 109.517 | 3.070 | 6297.632 | 396.288 | 2540.450 | 120.850 || 1B5g | 310005.0 | 118.984 | 3.314 | 7221.750 | 279.028 | 2891.000 | 126.000 || 1B5g | 372004.0 | 128.010 | 3.815 | 7897.121 | 435.845 | 3221.750 | 146.450 || 1B5g | 434003.0 | 136.575 | 4.307 | 8638.139 | 393.789 | 3535.450 | 160.950 || 1B5g | 496002.0 | 144.322 | 4.592 | 9258.659 | 608.062 | 3836.600 | 175.900 || 1B5g | 558001.0 | 151.205 | 4.668 | 9883.879 | 580.802 | 4099.800 | 181.800 || 1B5g | 620000.0 | 157.941 | 5.173 | 10519.341 | 581.018 | 4372.950 | 206.550 || 1BEDTA | 10.0 | 1.215 | 0.141 | 15.467 | 0.283 | 6.900 | 0.100 || 1BEDTA | 62009.0 | 72.284 | 0.367 | 2979.205 | 9.938 | 1221.700 | 3.100 || 1BEDTA | 124008.0 | 93.774 | 0.138 | 4558.836 | 93.673 | 1835.900 | 19.200 || 1BEDTA | 186007.0 | 109.839 | 1.069 | 5855.665 | 54.536 | 2338.750 | 13.350 || 1BEDTA | 248006.0 | 122.532 | 0.595 | 6938.348 | 18.583 | 2767.200 | 12.100 || 1BEDTA | 310005.0 | 134.592 | 0.711 | 8129.458 | 1.587 | 3186.550 | 25.150 || 1BEDTA | 372004.0 | 144.396 | 1.127 | 8921.561 | 38.726 | 3545.950 | 29.250 || 1BEDTA | 434003.0 | 153.393 | 0.707 | 9729.763 | 203.037 | 3893.950 | 43.850 || 1BEDTA | 496002.0 | 161.349 | 0.942 | 10592.400 | 161.114 | 4215.100 | 36.900 || 1BEDTA | 558001.0 | 169.336 | 1.080 | 11115.642 | 121.826 | 4517.000 | 39.700 || 1BEDTA | 620000.0 | 176.683 | 0.498 | 11885.349 | 235.772 | 4814.400 | 61.100 || 1Mudstone | 10.0 | 0.865 | 0.161 | 18.210 | 4.210 | 7.200 | 0.400 || 1Mudstone | 62009.0 | 57.192 | 8.336 | 2497.869 | 378.290 | 999.050 | 168.950 || 1Mudstone | 124008.0 | 73.371 | 10.037 | 3784.145 | 549.721 | 1498.300 | 237.000 || 1Mudstone | 186007.0 | 84.951 | 11.690 | 4834.557 | 667.051 | 1909.300 | 286.500 || 1Mudstone | 248006.0 | 94.984 | 12.736 | 5857.461 | 848.675 | 2282.750 | 338.350 || 1Mudstone | 310005.0 | 102.989 | 13.951 | 6839.714 | 863.355 | 2607.550 | 393.550 || 1Mudstone | 372004.0 | 109.795 | 16.079 | 7590.699 | 1011.464 | 2904.950 | 449.050 || 1Mudstone | 434003.0 | 115.905 | 16.896 | 8286.981 | 1087.668 | 3178.100 | 489.800 || 1Mudstone | 496002.0 | 122.477 | 17.767 | 9190.820 | 1226.095 | 3463.950 | 521.150 || 1Mudstone | 558001.0 | 128.324 | 17.923 | 9730.640 | 1251.105 | 3710.000 | 554.200 || 1Mudstone | 620000.0 | 133.316 | 19.072 | 10562.751 | 1419.178 | 3972.800 | 595.100 || 1Scrappings | 10.0 | 0.708 | 0.053 | 11.542 | 1.092 | 5.700 | 0.100 || 1Scrappings | 62009.0 | 48.870 | 4.561 | 2047.363 | 94.517 | 762.650 | 105.650 || 1Scrappings | 124008.0 | 59.581 | 4.775 | 3341.715 | 26.892 | 1124.050 | 127.450 || 1Scrappings | 186007.0 | 67.464 | 4.854 | 4526.239 | 30.227 | 1434.850 | 134.650 || 1Scrappings | 248006.0 | 74.786 | 4.421 | 5526.461 | 141.670 | 1722.150 | 144.350 || 1Scrappings | 310005.0 | 80.857 | 4.710 | 6446.843 | 191.105 | 1969.100 | 156.100 || 1Scrappings | 372004.0 | 87.028 | 4.482 | 7027.472 | 283.416 | 2218.000 | 159.400 || 1Scrappings | 434003.0 | 92.522 | 4.703 | 7890.813 | 434.458 | 2458.900 | 165.600 || 1Scrappings | 496002.0 | 97.501 | 4.605 | 8487.428 | 428.232 | 2671.600 | 176.000 || 1Scrappings | 558001.0 | 102.301 | 4.409 | 9209.047 | 465.327 | 2898.150 | 182.050 || 1Scrappings | 620000.0 | 107.002 | 4.579 | 9819.349 | 429.245 | 3105.400 | 188.000 |
| LinkerPrimerSequence | Seqs/Sample | PD\_whole\_tree Ave. | PD\_whole\_tree Err. | chao1 Ave. | chao1 Err. | observed\_otus Ave. | observed\_otus Err. |
| CCGTAAAACGACGGCCAG | 10.0 | 1.053 | 0.316 | 15.580 | 3.342 | 6.850 | 0.766 || CCGTAAAACGACGGCCAG | 62009.0 | 60.710 | 9.949 | 2556.570 | 403.508 | 1024.775 | 200.257 || CCGTAAAACGACGGCCAG | 124008.0 | 77.496 | 13.818 | 3940.439 | 536.628 | 1534.088 | 299.897 || CCGTAAAACGACGGCCAG | 186007.0 | 89.942 | 16.982 | 5107.859 | 609.462 | 1954.375 | 375.318 || CCGTAAAACGACGGCCAG | 248006.0 | 100.455 | 19.039 | 6154.976 | 709.803 | 2328.137 | 435.087 || CCGTAAAACGACGGCCAG | 310005.0 | 109.355 | 21.275 | 7159.441 | 776.949 | 2663.550 | 501.602 || CCGTAAAACGACGGCCAG | 372004.0 | 117.307 | 23.000 | 7859.213 | 892.855 | 2972.662 | 550.945 || CCGTAAAACGACGGCCAG | 434003.0 | 124.598 | 24.514 | 8636.424 | 927.565 | 3266.600 | 596.074 || CCGTAAAACGACGGCCAG | 496002.0 | 131.413 | 25.746 | 9382.327 | 1048.747 | 3546.812 | 639.985 || CCGTAAAACGACGGCCAG | 558001.0 | 137.791 | 26.869 | 9984.802 | 1011.106 | 3806.238 | 670.849 || CCGTAAAACGACGGCCAG | 620000.0 | 143.735 | 28.093 | 10696.698 | 1098.125 | 4066.387 | 710.922 |
| BarcodeSequence | Seqs/Sample | PD\_whole\_tree Ave. | PD\_whole\_tree Err. | chao1 Ave. | chao1 Err. | observed\_otus Ave. | observed\_otus Err. |
| AGGCAGAAAGAGTAGA | 10.0 | 0.703 | nan | 14.000 | nan | 6.800 | nan || AGGCAGAAAGAGTAGA | 62009.0 | 48.855 | nan | 2119.579 | nan | 830.100 | nan || AGGCAGAAAGAGTAGA | 124008.0 | 63.334 | nan | 3234.424 | nan | 1261.300 | nan || AGGCAGAAAGAGTAGA | 186007.0 | 73.261 | nan | 4167.506 | nan | 1622.800 | nan || AGGCAGAAAGAGTAGA | 248006.0 | 82.248 | nan | 5008.786 | nan | 1944.400 | nan || AGGCAGAAAGAGTAGA | 310005.0 | 89.038 | nan | 5976.360 | nan | 2214.000 | nan || AGGCAGAAAGAGTAGA | 372004.0 | 93.716 | nan | 6579.235 | nan | 2455.900 | nan || AGGCAGAAAGAGTAGA | 434003.0 | 99.009 | nan | 7199.313 | nan | 2688.300 | nan || AGGCAGAAAGAGTAGA | 496002.0 | 104.710 | nan | 7964.725 | nan | 2942.800 | nan || AGGCAGAAAGAGTAGA | 558001.0 | 110.401 | nan | 8479.535 | nan | 3155.800 | nan || AGGCAGAAAGAGTAGA | 620000.0 | 114.244 | nan | 9143.573 | nan | 3377.700 | nan || AGGCAGAATATCCTCT | 10.0 | 1.026 | nan | 22.420 | nan | 7.600 | nan || AGGCAGAATATCCTCT | 62009.0 | 65.528 | nan | 2876.159 | nan | 1168.000 | nan || AGGCAGAATATCCTCT | 124008.0 | 83.407 | nan | 4333.866 | nan | 1735.300 | nan || AGGCAGAATATCCTCT | 186007.0 | 96.641 | nan | 5501.608 | nan | 2195.800 | nan || AGGCAGAATATCCTCT | 248006.0 | 107.720 | nan | 6706.136 | nan | 2621.100 | nan || AGGCAGAATATCCTCT | 310005.0 | 116.940 | nan | 7703.069 | nan | 3001.100 | nan || AGGCAGAATATCCTCT | 372004.0 | 125.874 | nan | 8602.164 | nan | 3354.000 | nan || AGGCAGAATATCCTCT | 434003.0 | 132.801 | nan | 9374.649 | nan | 3667.900 | nan || AGGCAGAATATCCTCT | 496002.0 | 140.244 | nan | 10416.915 | nan | 3985.100 | nan || AGGCAGAATATCCTCT | 558001.0 | 146.247 | nan | 10981.745 | nan | 4264.200 | nan || AGGCAGAATATCCTCT | 620000.0 | 152.387 | nan | 11981.929 | nan | 4567.900 | nan || CGTACTAGAGAGTAGA | 10.0 | 0.760 | nan | 12.633 | nan | 5.800 | nan || CGTACTAGAGAGTAGA | 62009.0 | 44.309 | nan | 2141.880 | nan | 657.000 | nan || CGTACTAGAGAGTAGA | 124008.0 | 54.806 | nan | 3368.606 | nan | 996.600 | nan || CGTACTAGAGAGTAGA | 186007.0 | 62.611 | nan | 4496.012 | nan | 1300.200 | nan || CGTACTAGAGAGTAGA | 248006.0 | 70.365 | nan | 5384.792 | nan | 1577.800 | nan || CGTACTAGAGAGTAGA | 310005.0 | 76.147 | nan | 6255.737 | nan | 1813.000 | nan || CGTACTAGAGAGTAGA | 372004.0 | 82.546 | nan | 6744.055 | nan | 2058.600 | nan || CGTACTAGAGAGTAGA | 434003.0 | 87.819 | nan | 7456.355 | nan | 2293.300 | nan || CGTACTAGAGAGTAGA | 496002.0 | 92.897 | nan | 8059.196 | nan | 2495.600 | nan || CGTACTAGAGAGTAGA | 558001.0 | 97.892 | nan | 8743.719 | nan | 2716.100 | nan || CGTACTAGAGAGTAGA | 620000.0 | 102.423 | nan | 9390.105 | nan | 2917.400 | nan || CGTACTAGTATCCTCT | 10.0 | 0.655 | nan | 10.450 | nan | 5.600 | nan || CGTACTAGTATCCTCT | 62009.0 | 53.432 | nan | 1952.845 | nan | 868.300 | nan || CGTACTAGTATCCTCT | 124008.0 | 64.356 | nan | 3314.823 | nan | 1251.500 | nan || CGTACTAGTATCCTCT | 186007.0 | 72.318 | nan | 4556.466 | nan | 1569.500 | nan || CGTACTAGTATCCTCT | 248006.0 | 79.207 | nan | 5668.131 | nan | 1866.500 | nan || CGTACTAGTATCCTCT | 310005.0 | 85.567 | nan | 6637.948 | nan | 2125.200 | nan || CGTACTAGTATCCTCT | 372004.0 | 91.509 | nan | 7310.888 | nan | 2377.400 | nan || CGTACTAGTATCCTCT | 434003.0 | 97.225 | nan | 8325.270 | nan | 2624.500 | nan || CGTACTAGTATCCTCT | 496002.0 | 102.106 | nan | 8915.659 | nan | 2847.600 | nan || CGTACTAGTATCCTCT | 558001.0 | 106.709 | nan | 9674.374 | nan | 3080.200 | nan || CGTACTAGTATCCTCT | 620000.0 | 111.581 | nan | 10248.594 | nan | 3293.400 | nan || GTAGAGGAAGAGTAGA | 10.0 | 1.601 | nan | 17.450 | nan | 8.000 | nan || GTAGAGGAAGAGTAGA | 62009.0 | 62.337 | nan | 2510.335 | nan | 1050.300 | nan || GTAGAGGAAGAGTAGA | 124008.0 | 81.256 | nan | 3839.976 | nan | 1602.400 | nan || GTAGAGGAAGAGTAGA | 186007.0 | 94.533 | nan | 4984.181 | nan | 2030.200 | nan || GTAGAGGAAGAGTAGA | 248006.0 | 106.447 | nan | 5901.343 | nan | 2419.600 | nan || GTAGAGGAAGAGTAGA | 310005.0 | 115.670 | nan | 6942.722 | nan | 2765.000 | nan || GTAGAGGAAGAGTAGA | 372004.0 | 124.195 | nan | 7461.276 | nan | 3075.300 | nan || GTAGAGGAAGAGTAGA | 434003.0 | 132.268 | nan | 8244.349 | nan | 3374.500 | nan || GTAGAGGAAGAGTAGA | 496002.0 | 139.731 | nan | 8650.597 | nan | 3660.700 | nan || GTAGAGGAAGAGTAGA | 558001.0 | 146.537 | nan | 9303.077 | nan | 3918.000 | nan || GTAGAGGAAGAGTAGA | 620000.0 | 152.768 | nan | 9938.323 | nan | 4166.400 | nan || GTAGAGGATATCCTCT | 10.0 | 1.250 | nan | 16.750 | nan | 7.200 | nan || GTAGAGGATATCCTCT | 62009.0 | 66.651 | nan | 2893.352 | nan | 1181.100 | nan || GTAGAGGATATCCTCT | 124008.0 | 85.262 | nan | 4314.144 | nan | 1753.800 | nan || GTAGAGGATATCCTCT | 186007.0 | 100.492 | nan | 5445.769 | nan | 2239.000 | nan || GTAGAGGATATCCTCT | 248006.0 | 112.588 | nan | 6693.920 | nan | 2661.300 | nan || GTAGAGGATATCCTCT | 310005.0 | 122.297 | nan | 7500.778 | nan | 3017.000 | nan || GTAGAGGATATCCTCT | 372004.0 | 131.825 | nan | 8332.967 | nan | 3368.200 | nan || GTAGAGGATATCCTCT | 434003.0 | 140.881 | nan | 9031.928 | nan | 3696.400 | nan || GTAGAGGATATCCTCT | 496002.0 | 148.914 | nan | 9866.721 | nan | 4012.500 | nan || GTAGAGGATATCCTCT | 558001.0 | 155.874 | nan | 10464.681 | nan | 4281.600 | nan || GTAGAGGATATCCTCT | 620000.0 | 163.114 | nan | 11100.359 | nan | 4579.500 | nan || TCCTGAGCAGAGTAGA | 10.0 | 1.356 | nan | 15.750 | nan | 6.800 | nan || TCCTGAGCAGAGTAGA | 62009.0 | 72.651 | nan | 2969.266 | nan | 1218.600 | nan || TCCTGAGCAGAGTAGA | 124008.0 | 93.636 | nan | 4465.164 | nan | 1816.700 | nan || TCCTGAGCAGAGTAGA | 186007.0 | 110.908 | nan | 5910.201 | nan | 2325.400 | nan || TCCTGAGCAGAGTAGA | 248006.0 | 123.127 | nan | 6956.931 | nan | 2755.100 | nan || TCCTGAGCAGAGTAGA | 310005.0 | 135.303 | nan | 8127.870 | nan | 3161.400 | nan || TCCTGAGCAGAGTAGA | 372004.0 | 145.523 | nan | 8882.835 | nan | 3516.700 | nan || TCCTGAGCAGAGTAGA | 434003.0 | 154.100 | nan | 9526.726 | nan | 3850.100 | nan || TCCTGAGCAGAGTAGA | 496002.0 | 162.291 | nan | 10431.286 | nan | 4178.200 | nan || TCCTGAGCAGAGTAGA | 558001.0 | 170.416 | nan | 10993.816 | nan | 4477.300 | nan || TCCTGAGCAGAGTAGA | 620000.0 | 177.180 | nan | 11649.578 | nan | 4753.300 | nan || TCCTGAGCTATCCTCT | 10.0 | 1.074 | nan | 15.183 | nan | 7.000 | nan || TCCTGAGCTATCCTCT | 62009.0 | 71.916 | nan | 2989.143 | nan | 1224.800 | nan || TCCTGAGCTATCCTCT | 124008.0 | 93.912 | nan | 4652.509 | nan | 1855.100 | nan || TCCTGAGCTATCCTCT | 186007.0 | 108.769 | nan | 5801.129 | nan | 2352.100 | nan || TCCTGAGCTATCCTCT | 248006.0 | 121.937 | nan | 6919.766 | nan | 2779.300 | nan || TCCTGAGCTATCCTCT | 310005.0 | 133.880 | nan | 8131.045 | nan | 3211.700 | nan || TCCTGAGCTATCCTCT | 372004.0 | 143.269 | nan | 8960.287 | nan | 3575.200 | nan || TCCTGAGCTATCCTCT | 434003.0 | 152.685 | nan | 9932.800 | nan | 3937.800 | nan || TCCTGAGCTATCCTCT | 496002.0 | 160.408 | nan | 10753.513 | nan | 4252.000 | nan || TCCTGAGCTATCCTCT | 558001.0 | 168.256 | nan | 11237.468 | nan | 4556.700 | nan || TCCTGAGCTATCCTCT | 620000.0 | 176.185 | nan | 12121.121 | nan | 4875.500 | nan |
| Description | Seqs/Sample | PD\_whole\_tree Ave. | PD\_whole\_tree Err. | chao1 Ave. | chao1 Err. | observed\_otus Ave. | observed\_otus Err. |
| 1B5g\_1 | 10.0 | 1.250 | nan | 16.750 | nan | 7.200 | nan || 1B5g\_1 | 62009.0 | 66.651 | nan | 2893.352 | nan | 1181.100 | nan || 1B5g\_1 | 124008.0 | 85.262 | nan | 4314.144 | nan | 1753.800 | nan || 1B5g\_1 | 186007.0 | 100.492 | nan | 5445.769 | nan | 2239.000 | nan || 1B5g\_1 | 248006.0 | 112.588 | nan | 6693.920 | nan | 2661.300 | nan || 1B5g\_1 | 310005.0 | 122.297 | nan | 7500.778 | nan | 3017.000 | nan || 1B5g\_1 | 372004.0 | 131.825 | nan | 8332.967 | nan | 3368.200 | nan || 1B5g\_1 | 434003.0 | 140.881 | nan | 9031.928 | nan | 3696.400 | nan || 1B5g\_1 | 496002.0 | 148.914 | nan | 9866.721 | nan | 4012.500 | nan || 1B5g\_1 | 558001.0 | 155.874 | nan | 10464.681 | nan | 4281.600 | nan || 1B5g\_1 | 620000.0 | 163.114 | nan | 11100.359 | nan | 4579.500 | nan || 1B5g\_2 | 10.0 | 1.601 | nan | 17.450 | nan | 8.000 | nan || 1B5g\_2 | 62009.0 | 62.337 | nan | 2510.335 | nan | 1050.300 | nan || 1B5g\_2 | 124008.0 | 81.256 | nan | 3839.976 | nan | 1602.400 | nan || 1B5g\_2 | 186007.0 | 94.533 | nan | 4984.181 | nan | 2030.200 | nan || 1B5g\_2 | 248006.0 | 106.447 | nan | 5901.343 | nan | 2419.600 | nan || 1B5g\_2 | 310005.0 | 115.670 | nan | 6942.722 | nan | 2765.000 | nan || 1B5g\_2 | 372004.0 | 124.195 | nan | 7461.276 | nan | 3075.300 | nan || 1B5g\_2 | 434003.0 | 132.268 | nan | 8244.349 | nan | 3374.500 | nan || 1B5g\_2 | 496002.0 | 139.731 | nan | 8650.597 | nan | 3660.700 | nan || 1B5g\_2 | 558001.0 | 146.537 | nan | 9303.077 | nan | 3918.000 | nan || 1B5g\_2 | 620000.0 | 152.768 | nan | 9938.323 | nan | 4166.400 | nan || 1BEDTA\_1 | 10.0 | 1.074 | nan | 15.183 | nan | 7.000 | nan || 1BEDTA\_1 | 62009.0 | 71.916 | nan | 2989.143 | nan | 1224.800 | nan || 1BEDTA\_1 | 124008.0 | 93.912 | nan | 4652.509 | nan | 1855.100 | nan || 1BEDTA\_1 | 186007.0 | 108.769 | nan | 5801.129 | nan | 2352.100 | nan || 1BEDTA\_1 | 248006.0 | 121.937 | nan | 6919.766 | nan | 2779.300 | nan || 1BEDTA\_1 | 310005.0 | 133.880 | nan | 8131.045 | nan | 3211.700 | nan || 1BEDTA\_1 | 372004.0 | 143.269 | nan | 8960.287 | nan | 3575.200 | nan || 1BEDTA\_1 | 434003.0 | 152.685 | nan | 9932.800 | nan | 3937.800 | nan || 1BEDTA\_1 | 496002.0 | 160.408 | nan | 10753.513 | nan | 4252.000 | nan || 1BEDTA\_1 | 558001.0 | 168.256 | nan | 11237.468 | nan | 4556.700 | nan || 1BEDTA\_1 | 620000.0 | 176.185 | nan | 12121.121 | nan | 4875.500 | nan || 1BEDTA\_2 | 10.0 | 1.356 | nan | 15.750 | nan | 6.800 | nan || 1BEDTA\_2 | 62009.0 | 72.651 | nan | 2969.266 | nan | 1218.600 | nan || 1BEDTA\_2 | 124008.0 | 93.636 | nan | 4465.164 | nan | 1816.700 | nan || 1BEDTA\_2 | 186007.0 | 110.908 | nan | 5910.201 | nan | 2325.400 | nan || 1BEDTA\_2 | 248006.0 | 123.127 | nan | 6956.931 | nan | 2755.100 | nan || 1BEDTA\_2 | 310005.0 | 135.303 | nan | 8127.870 | nan | 3161.400 | nan || 1BEDTA\_2 | 372004.0 | 145.523 | nan | 8882.835 | nan | 3516.700 | nan || 1BEDTA\_2 | 434003.0 | 154.100 | nan | 9526.726 | nan | 3850.100 | nan || 1BEDTA\_2 | 496002.0 | 162.291 | nan | 10431.286 | nan | 4178.200 | nan || 1BEDTA\_2 | 558001.0 | 170.416 | nan | 10993.816 | nan | 4477.300 | nan || 1BEDTA\_2 | 620000.0 | 177.180 | nan | 11649.578 | nan | 4753.300 | nan || 1Mudstone\_1 | 10.0 | 1.026 | nan | 22.420 | nan | 7.600 | nan || 1Mudstone\_1 | 62009.0 | 65.528 | nan | 2876.159 | nan | 1168.000 | nan || 1Mudstone\_1 | 124008.0 | 83.407 | nan | 4333.866 | nan | 1735.300 | nan || 1Mudstone\_1 | 186007.0 | 96.641 | nan | 5501.608 | nan | 2195.800 | nan || 1Mudstone\_1 | 248006.0 | 107.720 | nan | 6706.136 | nan | 2621.100 | nan || 1Mudstone\_1 | 310005.0 | 116.940 | nan | 7703.069 | nan | 3001.100 | nan || 1Mudstone\_1 | 372004.0 | 125.874 | nan | 8602.164 | nan | 3354.000 | nan || 1Mudstone\_1 | 434003.0 | 132.801 | nan | 9374.649 | nan | 3667.900 | nan || 1Mudstone\_1 | 496002.0 | 140.244 | nan | 10416.915 | nan | 3985.100 | nan || 1Mudstone\_1 | 558001.0 | 146.247 | nan | 10981.745 | nan | 4264.200 | nan || 1Mudstone\_1 | 620000.0 | 152.387 | nan | 11981.929 | nan | 4567.900 | nan || 1Mudstone\_2 | 10.0 | 0.703 | nan | 14.000 | nan | 6.800 | nan || 1Mudstone\_2 | 62009.0 | 48.855 | nan | 2119.579 | nan | 830.100 | nan || 1Mudstone\_2 | 124008.0 | 63.334 | nan | 3234.424 | nan | 1261.300 | nan || 1Mudstone\_2 | 186007.0 | 73.261 | nan | 4167.506 | nan | 1622.800 | nan || 1Mudstone\_2 | 248006.0 | 82.248 | nan | 5008.786 | nan | 1944.400 | nan || 1Mudstone\_2 | 310005.0 | 89.038 | nan | 5976.360 | nan | 2214.000 | nan || 1Mudstone\_2 | 372004.0 | 93.716 | nan | 6579.235 | nan | 2455.900 | nan || 1Mudstone\_2 | 434003.0 | 99.009 | nan | 7199.313 | nan | 2688.300 | nan || 1Mudstone\_2 | 496002.0 | 104.710 | nan | 7964.725 | nan | 2942.800 | nan || 1Mudstone\_2 | 558001.0 | 110.401 | nan | 8479.535 | nan | 3155.800 | nan || 1Mudstone\_2 | 620000.0 | 114.244 | nan | 9143.573 | nan | 3377.700 | nan || 1scrappings\_1 | 10.0 | 0.655 | nan | 10.450 | nan | 5.600 | nan || 1scrappings\_1 | 62009.0 | 53.432 | nan | 1952.845 | nan | 868.300 | nan || 1scrappings\_1 | 124008.0 | 64.356 | nan | 3314.823 | nan | 1251.500 | nan || 1scrappings\_1 | 186007.0 | 72.318 | nan | 4556.466 | nan | 1569.500 | nan || 1scrappings\_1 | 248006.0 | 79.207 | nan | 5668.131 | nan | 1866.500 | nan || 1scrappings\_1 | 310005.0 | 85.567 | nan | 6637.948 | nan | 2125.200 | nan || 1scrappings\_1 | 372004.0 | 91.509 | nan | 7310.888 | nan | 2377.400 | nan || 1scrappings\_1 | 434003.0 | 97.225 | nan | 8325.270 | nan | 2624.500 | nan || 1scrappings\_1 | 496002.0 | 102.106 | nan | 8915.659 | nan | 2847.600 | nan || 1scrappings\_1 | 558001.0 | 106.709 | nan | 9674.374 | nan | 3080.200 | nan || 1scrappings\_1 | 620000.0 | 111.581 | nan | 10248.594 | nan | 3293.400 | nan || 1scrappings\_2 | 10.0 | 0.760 | nan | 12.633 | nan | 5.800 | nan || 1scrappings\_2 | 62009.0 | 44.309 | nan | 2141.880 | nan | 657.000 | nan || 1scrappings\_2 | 124008.0 | 54.806 | nan | 3368.606 | nan | 996.600 | nan || 1scrappings\_2 | 186007.0 | 62.611 | nan | 4496.012 | nan | 1300.200 | nan || 1scrappings\_2 | 248006.0 | 70.365 | nan | 5384.792 | nan | 1577.800 | nan || 1scrappings\_2 | 310005.0 | 76.147 | nan | 6255.737 | nan | 1813.000 | nan || 1scrappings\_2 | 372004.0 | 82.546 | nan | 6744.055 | nan | 2058.600 | nan || 1scrappings\_2 | 434003.0 | 87.819 | nan | 7456.355 | nan | 2293.300 | nan || 1scrappings\_2 | 496002.0 | 92.897 | nan | 8059.196 | nan | 2495.600 | nan || 1scrappings\_2 | 558001.0 | 97.892 | nan | 8743.719 | nan | 2716.100 | nan || 1scrappings\_2 | 620000.0 | 102.423 | nan | 9390.105 | nan | 2917.400 | nan |
| SampleID | Seqs/Sample | PD\_whole\_tree Ave. | PD\_whole\_tree Err. | chao1 Ave. | chao1 Err. | observed\_otus Ave. | observed\_otus Err. |
| 1B5g1 | 10.0 | 1.250 | nan | 16.750 | nan | 7.200 | nan || 1B5g1 | 62009.0 | 66.651 | nan | 2893.352 | nan | 1181.100 | nan || 1B5g1 | 124008.0 | 85.262 | nan | 4314.144 | nan | 1753.800 | nan || 1B5g1 | 186007.0 | 100.492 | nan | 5445.769 | nan | 2239.000 | nan || 1B5g1 | 248006.0 | 112.588 | nan | 6693.920 | nan | 2661.300 | nan || 1B5g1 | 310005.0 | 122.297 | nan | 7500.778 | nan | 3017.000 | nan || 1B5g1 | 372004.0 | 131.825 | nan | 8332.967 | nan | 3368.200 | nan || 1B5g1 | 434003.0 | 140.881 | nan | 9031.928 | nan | 3696.400 | nan || 1B5g1 | 496002.0 | 148.914 | nan | 9866.721 | nan | 4012.500 | nan || 1B5g1 | 558001.0 | 155.874 | nan | 10464.681 | nan | 4281.600 | nan || 1B5g1 | 620000.0 | 163.114 | nan | 11100.359 | nan | 4579.500 | nan || 1B5g2 | 10.0 | 1.601 | nan | 17.450 | nan | 8.000 | nan || 1B5g2 | 62009.0 | 62.337 | nan | 2510.335 | nan | 1050.300 | nan || 1B5g2 | 124008.0 | 81.256 | nan | 3839.976 | nan | 1602.400 | nan || 1B5g2 | 186007.0 | 94.533 | nan | 4984.181 | nan | 2030.200 | nan || 1B5g2 | 248006.0 | 106.447 | nan | 5901.343 | nan | 2419.600 | nan || 1B5g2 | 310005.0 | 115.670 | nan | 6942.722 | nan | 2765.000 | nan || 1B5g2 | 372004.0 | 124.195 | nan | 7461.276 | nan | 3075.300 | nan || 1B5g2 | 434003.0 | 132.268 | nan | 8244.349 | nan | 3374.500 | nan || 1B5g2 | 496002.0 | 139.731 | nan | 8650.597 | nan | 3660.700 | nan || 1B5g2 | 558001.0 | 146.537 | nan | 9303.077 | nan | 3918.000 | nan || 1B5g2 | 620000.0 | 152.768 | nan | 9938.323 | nan | 4166.400 | nan || 1BEDTA1 | 10.0 | 1.074 | nan | 15.183 | nan | 7.000 | nan || 1BEDTA1 | 62009.0 | 71.916 | nan | 2989.143 | nan | 1224.800 | nan || 1BEDTA1 | 124008.0 | 93.912 | nan | 4652.509 | nan | 1855.100 | nan || 1BEDTA1 | 186007.0 | 108.769 | nan | 5801.129 | nan | 2352.100 | nan || 1BEDTA1 | 248006.0 | 121.937 | nan | 6919.766 | nan | 2779.300 | nan || 1BEDTA1 | 310005.0 | 133.880 | nan | 8131.045 | nan | 3211.700 | nan || 1BEDTA1 | 372004.0 | 143.269 | nan | 8960.287 | nan | 3575.200 | nan || 1BEDTA1 | 434003.0 | 152.685 | nan | 9932.800 | nan | 3937.800 | nan || 1BEDTA1 | 496002.0 | 160.408 | nan | 10753.513 | nan | 4252.000 | nan || 1BEDTA1 | 558001.0 | 168.256 | nan | 11237.468 | nan | 4556.700 | nan || 1BEDTA1 | 620000.0 | 176.185 | nan | 12121.121 | nan | 4875.500 | nan || 1BEDTA2 | 10.0 | 1.356 | nan | 15.750 | nan | 6.800 | nan || 1BEDTA2 | 62009.0 | 72.651 | nan | 2969.266 | nan | 1218.600 | nan || 1BEDTA2 | 124008.0 | 93.636 | nan | 4465.164 | nan | 1816.700 | nan || 1BEDTA2 | 186007.0 | 110.908 | nan | 5910.201 | nan | 2325.400 | nan || 1BEDTA2 | 248006.0 | 123.127 | nan | 6956.931 | nan | 2755.100 | nan || 1BEDTA2 | 310005.0 | 135.303 | nan | 8127.870 | nan | 3161.400 | nan || 1BEDTA2 | 372004.0 | 145.523 | nan | 8882.835 | nan | 3516.700 | nan || 1BEDTA2 | 434003.0 | 154.100 | nan | 9526.726 | nan | 3850.100 | nan || 1BEDTA2 | 496002.0 | 162.291 | nan | 10431.286 | nan | 4178.200 | nan || 1BEDTA2 | 558001.0 | 170.416 | nan | 10993.816 | nan | 4477.300 | nan || 1BEDTA2 | 620000.0 | 177.180 | nan | 11649.578 | nan | 4753.300 | nan || 1M1 | 10.0 | 1.026 | nan | 22.420 | nan | 7.600 | nan || 1M1 | 62009.0 | 65.528 | nan | 2876.159 | nan | 1168.000 | nan || 1M1 | 124008.0 | 83.407 | nan | 4333.866 | nan | 1735.300 | nan || 1M1 | 186007.0 | 96.641 | nan | 5501.608 | nan | 2195.800 | nan || 1M1 | 248006.0 | 107.720 | nan | 6706.136 | nan | 2621.100 | nan || 1M1 | 310005.0 | 116.940 | nan | 7703.069 | nan | 3001.100 | nan || 1M1 | 372004.0 | 125.874 | nan | 8602.164 | nan | 3354.000 | nan || 1M1 | 434003.0 | 132.801 | nan | 9374.649 | nan | 3667.900 | nan || 1M1 | 496002.0 | 140.244 | nan | 10416.915 | nan | 3985.100 | nan || 1M1 | 558001.0 | 146.247 | nan | 10981.745 | nan | 4264.200 | nan || 1M1 | 620000.0 | 152.387 | nan | 11981.929 | nan | 4567.900 | nan || 1M2 | 10.0 | 0.703 | nan | 14.000 | nan | 6.800 | nan || 1M2 | 62009.0 | 48.855 | nan | 2119.579 | nan | 830.100 | nan || 1M2 | 124008.0 | 63.334 | nan | 3234.424 | nan | 1261.300 | nan || 1M2 | 186007.0 | 73.261 | nan | 4167.506 | nan | 1622.800 | nan || 1M2 | 248006.0 | 82.248 | nan | 5008.786 | nan | 1944.400 | nan || 1M2 | 310005.0 | 89.038 | nan | 5976.360 | nan | 2214.000 | nan || 1M2 | 372004.0 | 93.716 | nan | 6579.235 | nan | 2455.900 | nan || 1M2 | 434003.0 | 99.009 | nan | 7199.313 | nan | 2688.300 | nan || 1M2 | 496002.0 | 104.710 | nan | 7964.725 | nan | 2942.800 | nan || 1M2 | 558001.0 | 110.401 | nan | 8479.535 | nan | 3155.800 | nan || 1M2 | 620000.0 | 114.244 | nan | 9143.573 | nan | 3377.700 | nan || 1S1 | 10.0 | 0.655 | nan | 10.450 | nan | 5.600 | nan || 1S1 | 62009.0 | 53.432 | nan | 1952.845 | nan | 868.300 | nan || 1S1 | 124008.0 | 64.356 | nan | 3314.823 | nan | 1251.500 | nan || 1S1 | 186007.0 | 72.318 | nan | 4556.466 | nan | 1569.500 | nan || 1S1 | 248006.0 | 79.207 | nan | 5668.131 | nan | 1866.500 | nan || 1S1 | 310005.0 | 85.567 | nan | 6637.948 | nan | 2125.200 | nan || 1S1 | 372004.0 | 91.509 | nan | 7310.888 | nan | 2377.400 | nan || 1S1 | 434003.0 | 97.225 | nan | 8325.270 | nan | 2624.500 | nan || 1S1 | 496002.0 | 102.106 | nan | 8915.659 | nan | 2847.600 | nan || 1S1 | 558001.0 | 106.709 | nan | 9674.374 | nan | 3080.200 | nan || 1S1 | 620000.0 | 111.581 | nan | 10248.594 | nan | 3293.400 | nan || 1S2 | 10.0 | 0.760 | nan | 12.633 | nan | 5.800 | nan || 1S2 | 62009.0 | 44.309 | nan | 2141.880 | nan | 657.000 | nan || 1S2 | 124008.0 | 54.806 | nan | 3368.606 | nan | 996.600 | nan || 1S2 | 186007.0 | 62.611 | nan | 4496.012 | nan | 1300.200 | nan || 1S2 | 248006.0 | 70.365 | nan | 5384.792 | nan | 1577.800 | nan || 1S2 | 310005.0 | 76.147 | nan | 6255.737 | nan | 1813.000 | nan || 1S2 | 372004.0 | 82.546 | nan | 6744.055 | nan | 2058.600 | nan || 1S2 | 434003.0 | 87.819 | nan | 7456.355 | nan | 2293.300 | nan || 1S2 | 496002.0 | 92.897 | nan | 8059.196 | nan | 2495.600 | nan || 1S2 | 558001.0 | 97.892 | nan | 8743.719 | nan | 2716.100 | nan || 1S2 | 620000.0 | 102.423 | nan | 9390.105 | nan | 2917.400 | nan |
