# Supplementary material for: Cretaceous dinosaur bone contains recent organic material and provides an environment conducive to microbial communities
Source: eLife. 2019 Jun 18;8:e46205. doi: 10.7554/eLife.46205 (PMC6581507; doi:10.7554/eLife.46205)
Supplement: Source data 1. [file elife-46205-data1.zip › Raw data files/16S rRNA amplicon sequencing/taxa_plots/taxa_summary_plots/charts/K12p57GwUoS9uilaBWTnHHjbe9AOuC_legend.pdf]

D_0__Archaea;D_1__Thaumarchaeota;D_2__Soil Crenarchaeotic Group(SCG);D_3__Unknown Order
D_0__Archaea;D_1__Thaumarchaeota;D_2__Soil Crenarchaeotic Group(SCG);D_3__uncultured archaeon
D_0__Archaea;D_1__Thaumarchaeota;D_2__Soil Crenarchaeotic Group(SCG);D_3__uncultured euryarchaeote
D_0__Archaea;D_1__Thaumarchaeota;D_2__Soil Crenarchaeotic Group(SCG);Other
D_0__Bacteria;D_1__Acidobacteria;D_2__Blastocatellia;D_3__Blastocatellales
D_0__Bacteria;D_1__Acidobacteria;D_2__Holophagae;D_3__Holophagales
D_0__Bacteria;D_1__Acidobacteria;D_2__Holophagae;D_3__Subgroup 10
D_0__Bacteria;D_1__Acidobacteria;D_2__Holophagae;D_3__Subgroup 7
D_0__Bacteria;D_1__Acidobacteria;D_2__Solibacteres;D_3__Solibacterales
D_0__Bacteria;D_1__Acidobacteria;D_2__Subgroup 17;Ambiguous_taxa
D_0__Bacteria;D_1__Acidobacteria;D_2__Subgroup 17;D_3__uncultured Acidobacteria bacterium
D_0__Bacteria;D_1__Acidobacteria;D_2__Subgroup 17;Other
D_0__Bacteria;D_1__Acidobacteria;D_2__Subgroup 22;Ambiguous_taxa
D_0__Bacteria;D_1__Acidobacteria;D_2__Subgroup 22;D_3__uncultured Acidobacteria bacterium
D_0__Bacteria;D_1__Acidobacteria;D_2__Subgroup 22;D_3__uncultured bacterium
D_0__Bacteria;D_1__Acidobacteria;D_2__Subgroup 22;Other
D_0__Bacteria;D_1__Acidobacteria;D_2__Subgroup 5;D_3__uncultured bacterium
D_0__Bacteria;D_1__Acidobacteria;D_2__Subgroup 5;Other
D_0__Bacteria;D_1__Acidobacteria;D_2__Subgroup 6;Ambiguous_taxa
D_0__Bacteria;D_1__Acidobacteria;D_2__Subgroup 6;D_3__uncultured Acidobacteria bacterium
D_0__Bacteria;D_1__Acidobacteria;D_2__Subgroup 6;D_3__uncultured bacterium
D_0__Bacteria;D_1__Acidobacteria;D_2__Subgroup 6;D_3__uncultured organism
D_0__Bacteria;D_1__Acidobacteria;D_2__Subgroup 6;D_3__uncultured soil bacterium
D_0__Bacteria;D_1__Acidobacteria;D_2__Subgroup 6;Other
D_0__Bacteria;D_1__Acidobacteria;Other;Other
D_0__Bacteria;D_1__Actinobacteria;D_2__Acidimicrobiia;D_3__Acidimicrobiales
D_0__Bacteria;D_1__Actinobacteria;D_2__Actinobacteria;D_3__Actinomycetales
D_0__Bacteria;D_1__Actinobacteria;D_2__Actinobacteria;D_3__Actinopolysporales
D_0__Bacteria;D_1__Actinobacteria;D_2__Actinobacteria;D_3__Bifidobacteriales
D_0__Bacteria;D_1__Actinobacteria;D_2__Actinobacteria;D_3__Corynebacteriales
D_0__Bacteria;D_1__Actinobacteria;D_2__Actinobacteria;D_3__Elev-16S-976
D_0__Bacteria;D_1__Actinobacteria;D_2__Actinobacteria;D_3__Frankiales
D_0__Bacteria;D_1__Actinobacteria;D_2__Actinobacteria;D_3__Glycomycetales
D_0__Bacteria;D_1__Actinobacteria;D_2__Actinobacteria;D_3__Kineosporiales
D_0__Bacteria;D_1__Actinobacteria;D_2__Actinobacteria;D_3__Micrococcales
D_0__Bacteria;D_1__Actinobacteria;D_2__Actinobacteria;D_3__Micromonosporales
D_0__Bacteria;D_1__Actinobacteria;D_2__Actinobacteria;D_3__PeM15
D_0__Bacteria;D_1__Actinobacteria;D_2__Actinobacteria;D_3__Propionibacteriales
D_0__Bacteria;D_1__Actinobacteria;D_2__Actinobacteria;D_3__Pseudonocardiales
D_0__Bacteria;D_1__Actinobacteria;D_2__Actinobacteria;D_3__Streptomycetales
D_0__Bacteria;D_1__Actinobacteria;D_2__Actinobacteria;D_3__Streptosporangiales
D_0__Bacteria;D_1__Actinobacteria;D_2__Actinobacteria;Other
D_0__Bacteria;D_1__Actinobacteria;D_2__Coriobacteriia;D_3__Coriobacteriales
D_0__Bacteria;D_1__Actinobacteria;D_2__MB-A2-108;Ambiguous_taxa
D_0__Bacteria;D_1__Actinobacteria;D_2__MB-A2-108;D_3__uncultured bacterium
D_0__Bacteria;D_1__Actinobacteria;D_2__MB-A2-108;Other
D_0__Bacteria;D_1__Actinobacteria;D_2__Nitriliruptoria;D_3__Euzebyales
D_0__Bacteria;D_1__Actinobacteria;D_2__Nitriliruptoria;D_3__Nitriliruptorales
D_0__Bacteria;D_1__Actinobacteria;D_2__Nitriliruptoria;Other
D_0__Bacteria;D_1__Actinobacteria;D_2__OPB41;D_3__uncultured bacterium
D_0__Bacteria;D_1__Actinobacteria;D_2__Rubrobacteria;D_3__Rubrobacterales
D_0__Bacteria;D_1__Actinobacteria;D_2__TakashiAC-B11;D_3__uncultured Actinomycetales bacterium
D_0__Bacteria;D_1__Actinobacteria;D_2__TakashiAC-B11;D_3__uncultured actinobacterium
D_0__Bacteria;D_1__Actinobacteria;D_2__TakashiAC-B11;D_3__uncultured bacterium
D_0__Bacteria;D_1__Actinobacteria;D_2__TakashiAC-B11;Other
D_0__Bacteria;D_1__Actinobacteria;D_2__Thermoleophilia;D_3__Gaiellales
D_0__Bacteria;D_1__Actinobacteria;D_2__Thermoleophilia;D_3__Solirubrobacterales
D_0__Bacteria;D_1__Actinobacteria;D_2__Thermoleophilia;Other
D_0__Bacteria;D_1__Actinobacteria;Other;Other
D_0__Bacteria;D_1__Armatimonadetes;D_2__Armatimonadia;D_3__Armatimonadales
D_0__Bacteria;D_1__Armatimonadetes;D_2__Fimbriimonadia;D_3__Fimbriimonadales
D_0__Bacteria;D_1__Armatimonadetes;D_2__uncultured;D_3__uncultured bacterium
D_0__Bacteria;D_1__BRC1;D_2__uncultured bacterium;D_3__uncultured bacterium
D_0__Bacteria;D_1__BRC1;Other;Other
D_0__Bacteria;D_1__Bacteroidetes;D_2__Bacteroidetes BD2-2;D_3__uncultured bacterium
D_0__Bacteria;D_1__Bacteroidetes;D_2__Bacteroidetes Incertae Sedis;D_3__Order II
D_0__Bacteria;D_1__Bacteroidetes;D_2__Bacteroidia;D_3__Bacteroidales
D_0__Bacteria;D_1__Bacteroidetes;D_2__Cytophagia;D_3__Cytophagales
D_0__Bacteria;D_1__Bacteroidetes;D_2__Flavobacteriia;D_3__Flavobacteriales
D_0__Bacteria;D_1__Bacteroidetes;D_2__Sphingobacteriia;D_3__Sphingobacteriales
D_0__Bacteria;D_1__Chlamydiae;D_2__Chlamydiae;D_3__Chlamydiales
D_0__Bacteria;D_1__Chlorobi;D_2__Chlorobia;D_3__Chlorobiales
D_0__Bacteria;D_1__Chloroflexi;D_2__Anaerolineae;D_3__Anaerolineales
D_0__Bacteria;D_1__Chloroflexi;D_2__Ardenticatenia;D_3__Ardenticatenales
D_0__Bacteria;D_1__Chloroflexi;D_2__Caldilineae;D_3__Caldilineales
D_0__Bacteria;D_1__Chloroflexi;D_2__Chloroflexi Incertae Sedis;D_3__Unknown Order
D_0__Bacteria;D_1__Chloroflexi;D_2__Chloroflexia;D_3__Chloroflexales
D_0__Bacteria;D_1__Chloroflexi;D_2__Chloroflexia;D_3__Herpetosiphonales
D_0__Bacteria;D_1__Chloroflexi;D_2__Chloroflexia;D_3__Kallotenuales
D_0__Bacteria;D_1__Chloroflexi;D_2__Gitt-GS-136;Ambiguous_taxa
D_0__Bacteria;D_1__Chloroflexi;D_2__Gitt-GS-136;D_3__uncultured bacterium
D_0__Bacteria;D_1__Chloroflexi;D_2__Gitt-GS-136;D_3__uncultured bacterium #0319-6C24
D_0__Bacteria;D_1__Chloroflexi;D_2__Gitt-GS-136;Other
D_0__Bacteria;D_1__Chloroflexi;D_2__JG30-KF-CM66;Ambiguous_taxa
D_0__Bacteria;D_1__Chloroflexi;D_2__JG30-KF-CM66;D_3__uncultured Chloroflexi bacterium
D_0__Bacteria;D_1__Chloroflexi;D_2__JG30-KF-CM66;D_3__uncultured bacterium
D_0__Bacteria;D_1__Chloroflexi;D_2__JG30-KF-CM66;Other
D_0__Bacteria;D_1__Chloroflexi;D_2__JG37-AG-4;D_3__uncultured bacterium
D_0__Bacteria;D_1__Chloroflexi;D_2__KD4-96;Ambiguous_taxa
D_0__Bacteria;D_1__Chloroflexi;D_2__KD4-96;D_3__uncultured bacterium
D_0__Bacteria;D_1__Chloroflexi;D_2__KD4-96;Other
D_0__Bacteria;D_1__Chloroflexi;D_2__Ktedonobacteria;D_3__C0119
D_0__Bacteria;D_1__Chloroflexi;D_2__NLS2-31;D_3__uncultured bacterium
D_0__Bacteria;D_1__Chloroflexi;D_2__P2-11E;D_3__uncultured bacterium
D_0__Bacteria;D_1__Chloroflexi;D_2__S085;Ambiguous_taxa
D_0__Bacteria;D_1__Chloroflexi;D_2__S085;D_3__uncultured Chloroflexi bacterium
D_0__Bacteria;D_1__Chloroflexi;D_2__S085;D_3__uncultured bacterium
D_0__Bacteria;D_1__Chloroflexi;D_2__S085;Other
D_0__Bacteria;D_1__Chloroflexi;D_2__SAR202 clade;Ambiguous_taxa
D_0__Bacteria;D_1__Chloroflexi;D_2__SAR202 clade;D_3__uncultured Chloroflexi bacterium
D_0__Bacteria;D_1__Chloroflexi;D_2__SAR202 clade;D_3__uncultured bacterium
D_0__Bacteria;D_1__Chloroflexi;D_2__SAR202 clade;Other
D_0__Bacteria;D_1__Chloroflexi;D_2__TK10;D_3__uncultured Chloroflexi bacterium
D_0__Bacteria;D_1__Chloroflexi;D_2__TK10;D_3__uncultured bacterium
D_0__Bacteria;D_1__Chloroflexi;D_2__TK10;D_3__uncultured soil bacterium
D_0__Bacteria;D_1__Chloroflexi;D_2__TK10;Other
D_0__Bacteria;D_1__Chloroflexi;D_2__Thermomicrobia;D_3__AKYG1722
D_0__Bacteria;D_1__Chloroflexi;D_2__Thermomicrobia;D_3__JG30-KF-CM45
D_0__Bacteria;D_1__Chloroflexi;D_2__Thermomicrobia;D_3__Sphaerobacterales
D_0__Bacteria;D_1__Chloroflexi;Other;Other
D_0__Bacteria;D_1__Cyanobacteria;D_2__Chloroplast;Ambiguous_taxa
D_0__Bacteria;D_1__Cyanobacteria;D_2__Chloroplast;D_3__uncultured bacterium
D_0__Bacteria;D_1__Cyanobacteria;D_2__Cyanobacteria;D_3__SubsectionIII
D_0__Bacteria;D_1__Cyanobacteria;D_2__Cyanobacteria;D_3__uncultured
D_0__Bacteria;D_1__Cyanobacteria;D_2__Melainabacteria;D_3__Gastranaerophilales
D_0__Bacteria;D_1__Cyanobacteria;D_2__Melainabacteria;D_3__Vampirovibrionales
D_0__Bacteria;D_1__Deinococcus-Thermus;D_2__Deinococci;D_3__Deinococcales
D_0__Bacteria;D_1__Deinococcus-Thermus;D_2__Deinococci;D_3__Thermales
D_0__Bacteria;D_1__Elusimicrobia;D_2__Elusimicrobia;D_3__Lineage IIa
D_0__Bacteria;D_1__Elusimicrobia;D_2__Elusimicrobia;D_3__Lineage IIb
D_0__Bacteria;D_1__Elusimicrobia;D_2__Elusimicrobia;D_3__Lineage IV
D_0__Bacteria;D_1__FBP;D_2__uncultured bacterium;D_3__uncultured bacterium
D_0__Bacteria;D_1__Fibrobacteres;D_2__Fibrobacteria;D_3__Fibrobacterales
D_0__Bacteria;D_1__Firmicutes;D_2__Bacilli;D_3__Bacillales
D_0__Bacteria;D_1__Firmicutes;D_2__Bacilli;D_3__Lactobacillales
D_0__Bacteria;D_1__Firmicutes;D_2__Clostridia;D_3__Clostridiales
D_0__Bacteria;D_1__Firmicutes;D_2__Clostridia;D_3__Halanaerobiales
D_0__Bacteria;D_1__Firmicutes;D_2__Clostridia;D_3__Thermoanaerobacterales
D_0__Bacteria;D_1__Firmicutes;D_2__Erysipelotrichia;D_3__Erysipelotrichales
D_0__Bacteria;D_1__Firmicutes;D_2__Negativicutes;D_3__Selenomonadales
D_0__Bacteria;D_1__Fusobacteria;D_2__Fusobacteriia;D_3__Fusobacteriales
D_0__Bacteria;D_1__Gemmatimonadetes;D_2__BD2-11 terrestrial group;D_3__uncultured bacterium
D_0__Bacteria;D_1__Gemmatimonadetes;D_2__Gemmatimonadetes;D_3__Gemmatimonadales
D_0__Bacteria;D_1__Gemmatimonadetes;D_2__Longimicrobia;D_3__Longimicrobiales
D_0__Bacteria;D_1__Gemmatimonadetes;D_2__S0134 terrestrial group;D_3__uncultured Gemmatimonadetes bacterium
D_0__Bacteria;D_1__Gemmatimonadetes;D_2__S0134 terrestrial group;D_3__uncultured bacterium
D_0__Bacteria;D_1__Hydrogenedentes;D_2__uncultured bacterium;D_3__uncultured bacterium
D_0__Bacteria;D_1__Nitrospirae;D_2__Nitrospira;D_3__Nitrospirales
D_0__Bacteria;D_1__Planctomycetes;D_2__Phycisphaerae;D_3__Phycisphaerales
D_0__Bacteria;D_1__Planctomycetes;D_2__Phycisphaerae;D_3__Tepidisphaerales
D_0__Bacteria;D_1__Planctomycetes;D_2__Planctomycetacia;D_3__Planctomycetales
D_0__Bacteria;D_1__Proteobacteria;D_2__Alphaproteobacteria;D_3__Alphaproteobacteria Incertae Sedis
D_0__Bacteria;D_1__Proteobacteria;D_2__Alphaproteobacteria;D_3__Caulobacterales
D_0__Bacteria;D_1__Proteobacteria;D_2__Alphaproteobacteria;D_3__Rhizobiales
D_0__Bacteria;D_1__Proteobacteria;D_2__Alphaproteobacteria;D_3__Rhodobacterales
D_0__Bacteria;D_1__Proteobacteria;D_2__Alphaproteobacteria;D_3__Rhodospirillales
D_0__Bacteria;D_1__Proteobacteria;D_2__Alphaproteobacteria;D_3__Rickettsiales
D_0__Bacteria;D_1__Proteobacteria;D_2__Alphaproteobacteria;D_3__Sphingomonadales
D_0__Bacteria;D_1__Proteobacteria;D_2__Alphaproteobacteria;Other
D_0__Bacteria;D_1__Proteobacteria;D_2__Betaproteobacteria;D_3__Burkholderiales
D_0__Bacteria;D_1__Proteobacteria;D_2__Betaproteobacteria;D_3__Hydrogenophilales
D_0__Bacteria;D_1__Proteobacteria;D_2__Betaproteobacteria;D_3__Methylophilales
D_0__Bacteria;D_1__Proteobacteria;D_2__Betaproteobacteria;D_3__Neisseriales
D_0__Bacteria;D_1__Proteobacteria;D_2__Betaproteobacteria;D_3__Nitrosomonadales
D_0__Bacteria;D_1__Proteobacteria;D_2__Betaproteobacteria;D_3__Rhodocyclales
D_0__Bacteria;D_1__Proteobacteria;D_2__Betaproteobacteria;D_3__SC-I-84
D_0__Bacteria;D_1__Proteobacteria;D_2__Betaproteobacteria;D_3__TRA3-20
D_0__Bacteria;D_1__Proteobacteria;D_2__Betaproteobacteria;Other
D_0__Bacteria;D_1__Proteobacteria;D_2__Deltaproteobacteria;D_3__Bdellovibrionales
D_0__Bacteria;D_1__Proteobacteria;D_2__Deltaproteobacteria;D_3__Desulfurellales
D_0__Bacteria;D_1__Proteobacteria;D_2__Deltaproteobacteria;D_3__Myxococcales
D_0__Bacteria;D_1__Proteobacteria;D_2__Deltaproteobacteria;D_3__Oligoflexales
D_0__Bacteria;D_1__Proteobacteria;D_2__Deltaproteobacteria;D_3__SAR324 clade(Marine group B)
D_0__Bacteria;D_1__Proteobacteria;D_2__Epsilonproteobacteria;D_3__Campylobacterales
D_0__Bacteria;D_1__Proteobacteria;D_2__Gammaproteobacteria;D_3__Alteromonadales
D_0__Bacteria;D_1__Proteobacteria;D_2__Gammaproteobacteria;D_3__Cardiobacteriales
D_0__Bacteria;D_1__Proteobacteria;D_2__Gammaproteobacteria;D_3__Cellvibrionales
D_0__Bacteria;D_1__Proteobacteria;D_2__Gammaproteobacteria;D_3__Chromatiales
D_0__Bacteria;D_1__Proteobacteria;D_2__Gammaproteobacteria;D_3__Enterobacteriales
D_0__Bacteria;D_1__Proteobacteria;D_2__Gammaproteobacteria;D_3__Gammaproteobacteria Incertae Sedis
D_0__Bacteria;D_1__Proteobacteria;D_2__Gammaproteobacteria;D_3__HTA4
D_0__Bacteria;D_1__Proteobacteria;D_2__Gammaproteobacteria;D_3__KI89A clade
D_0__Bacteria;D_1__Proteobacteria;D_2__Gammaproteobacteria;D_3__Legionellales
D_0__Bacteria;D_1__Proteobacteria;D_2__Gammaproteobacteria;D_3__Oceanospirillales
D_0__Bacteria;D_1__Proteobacteria;D_2__Gammaproteobacteria;D_3__PYR10d3
D_0__Bacteria;D_1__Proteobacteria;D_2__Gammaproteobacteria;D_3__Pasteurellales
D_0__Bacteria;D_1__Proteobacteria;D_2__Gammaproteobacteria;D_3__Pseudomonadales
D_0__Bacteria;D_1__Proteobacteria;D_2__Gammaproteobacteria;D_3__Thiotrichales
D_0__Bacteria;D_1__Proteobacteria;D_2__Gammaproteobacteria;D_3__Vibrionales
D_0__Bacteria;D_1__Proteobacteria;D_2__Gammaproteobacteria;D_3__X35
D_0__Bacteria;D_1__Proteobacteria;D_2__Gammaproteobacteria;D_3__Xanthomonadales
D_0__Bacteria;D_1__Proteobacteria;D_2__Gammaproteobacteria;D_3__uncultured
D_0__Bacteria;D_1__Proteobacteria;D_2__Gammaproteobacteria;Other
D_0__Bacteria;D_1__Proteobacteria;D_2__JTB23;D_3__uncultured bacterium
D_0__Bacteria;D_1__Proteobacteria;D_2__JTB23;Other
D_0__Bacteria;D_1__Proteobacteria;D_2__SPOTSOCT00m83;Ambiguous_taxa
D_0__Bacteria;D_1__Proteobacteria;D_2__SPOTSOCT00m83;Other
D_0__Bacteria;D_1__Proteobacteria;Other;Other
D_0__Bacteria;D_1__SBR1093;Other;Other
D_0__Bacteria;D_1__SR1 (Absconditabacteria);Ambiguous_taxa;Ambiguous_taxa
D_0__Bacteria;D_1__SR1 (Absconditabacteria);D_2__uncultured bacterium;D_3__uncultured bacterium
D_0__Bacteria;D_1__Saccharibacteria;D_2__uncultured bacterium;D_3__uncultured bacterium
D_0__Bacteria;D_1__Saccharibacteria;Other;Other
D_0__Bacteria;D_1__Spirochaetae;D_2__Spirochaetes;D_3__Spirochaetales
D_0__Bacteria;D_1__Synergistetes;D_2__Synergistia;D_3__Synergistales
D_0__Bacteria;D_1__TM6 (Dependentiae);Other;Other
D_0__Bacteria;D_1__Tectomicrobia;D_2__Tectomicrobia Incertae Sedis;D_3__Unknown Order
D_0__Bacteria;D_1__Tectomicrobia;D_2__uncultured bacterium;D_3__uncultured bacterium
D_0__Bacteria;D_1__Verrucomicrobia;D_2__OPB35 soil group;Ambiguous_taxa
D_0__Bacteria;D_1__Verrucomicrobia;D_2__OPB35 soil group;D_3__uncultured bacterium
D_0__Bacteria;D_1__Verrucomicrobia;D_2__OPB35 soil group;Other
D_0__Bacteria;D_1__Verrucomicrobia;D_2__Opitutae;D_3__BC-COM435
D_0__Bacteria;D_1__Verrucomicrobia;D_2__Opitutae;D_3__Opitutales
D_0__Bacteria;D_1__Verrucomicrobia;D_2__Spartobacteria;D_3__Chthoniobacterales
D_0__Bacteria;D_1__Verrucomicrobia;D_2__Verrucomicrobiae;D_3__Verrucomicrobiales
D_0__Bacteria;Other;Other;Other
Unassigned;Other;Other;Other
