# Supplementary material for: Cretaceous dinosaur bone contains recent organic material and provides an environment conducive to microbial communities
Source: eLife. 2019 Jun 18;8:e46205. doi: 10.7554/eLife.46205 (PMC6581507; doi:10.7554/eLife.46205)
Supplement: Source data 1. [file elife-46205-data1.zip › Raw data files/16S rRNA amplicon sequencing/taxa_plots/taxa_summary_plots/charts/gxK9FzeGKWFAIYftR4ahQS0dQ14ztj_legend.pdf]

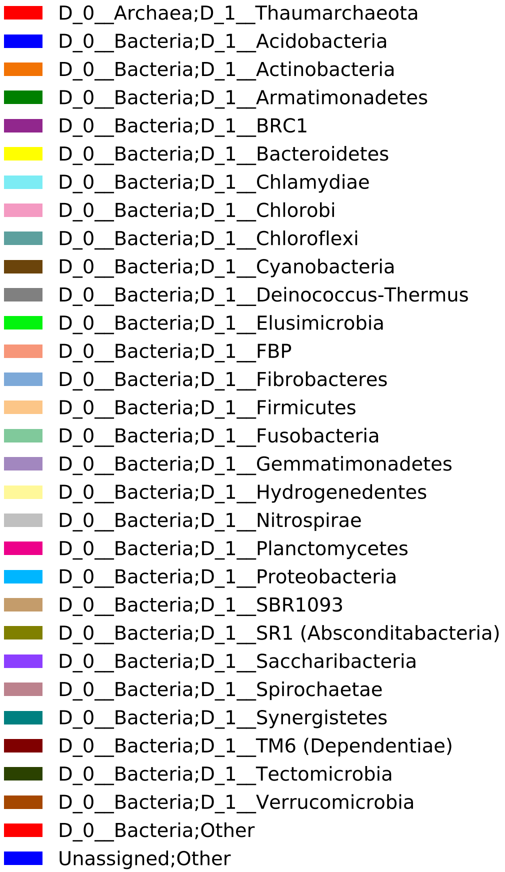
D_0__Archaea;D_1__Thaumarchaeota
D_0__Bacteria;D_1__Acidobacteria
D_0__Bacteria;D_1__Actinobacteria
D_0__Bacteria;D_1__Armatimonadetes
D_0__Bacteria;D_1__BRC1
D_0__Bacteria;D_1__Bacteroidetes
D_0__Bacteria;D_1__Chlamydiae
D_0__Bacteria;D_1__Chlorobi
D_0__Bacteria;D_1__Chloroflexi
D_0__Bacteria;D_1__Cyanobacteria
D_0__Bacteria;D_1__Deinococcus-Thermus
D_0__Bacteria;D_1__Elusimicrobia
D_0__Bacteria;D_1__FBP
D_0__Bacteria;D_1__Fibrobacteres
D_0__Bacteria;D_1__Firmicutes
D_0__Bacteria;D_1__Fusobacteria
D_0__Bacteria;D_1__Gemmatimonadetes
D_0__Bacteria;D_1__Hydrogenedentes
D_0__Bacteria;D_1__Nitrospirae
D_0__Bacteria;D_1__Planctomycetes
D_0__Bacteria;D_1__Proteobacteria
D_0__Bacteria;D_1__SBR1093
D_0__Bacteria;D_1__SR1 (Absconditabacteria)
D_0__Bacteria;D_1__Saccharibacteria
D_0__Bacteria;D_1__Spirochaetae
D_0__Bacteria;D_1__Synergistetes
D_0__Bacteria;D_1__TM6 (Dependentiae)
D_0__Bacteria;D_1__Tectomicrobia
D_0__Bacteria;D_1__Verrucomicrobia
D_0__Bacteria;Other
Unassigned;Other
