# Supplementary material for: Cretaceous dinosaur bone contains recent organic material and provides an environment conducive to microbial communities
Source: eLife. 2019 Jun 18;8:e46205. doi: 10.7554/eLife.46205 (PMC6581507; doi:10.7554/eLife.46205)
Supplement: Source data 1. [file elife-46205-data1.zip › Raw data files/16S rRNA amplicon sequencing/taxa_plots/taxa_summary_plots/charts/yzD0FdKE7haGHk6I12wafU7EJ2DpoP_legend.pdf]

D_0__Archaea;D_1__Thaumarchaeota;D_2__Soil Crenarchaeotic Group(SCG)
D_0__Bacteria;D_1__Acidobacteria;D_2__Blastocatellia
D_0__Bacteria;D_1__Acidobacteria;D_2__Holophagae
D_0__Bacteria;D_1__Acidobacteria;D_2__Solibacteres
D_0__Bacteria;D_1__Acidobacteria;D_2__Subgroup 17
D_0__Bacteria;D_1__Acidobacteria;D_2__Subgroup 22
D_0__Bacteria;D_1__Acidobacteria;D_2__Subgroup 5
D_0__Bacteria;D_1__Acidobacteria;D_2__Subgroup 6
D_0__Bacteria;D_1__Acidobacteria;Other
D_0__Bacteria;D_1__Actinobacteria;D_2__Acidimicrobiia
D_0__Bacteria;D_1__Actinobacteria;D_2__Actinobacteria
D_0__Bacteria;D_1__Actinobacteria;D_2__Coriobacteriia
D_0__Bacteria;D_1__Actinobacteria;D_2__MB-A2-108
D_0__Bacteria;D_1__Actinobacteria;D_2__Nitriliruptoria
D_0__Bacteria;D_1__Actinobacteria;D_2__OPB41
D_0__Bacteria;D_1__Actinobacteria;D_2__Rubrobacteria
D_0__Bacteria;D_1__Actinobacteria;D_2__TakashiAC-B11
D_0__Bacteria;D_1__Actinobacteria;D_2__Thermoleophilia
D_0__Bacteria;D_1__Actinobacteria;Other
D_0__Bacteria;D_1__Armatimonadetes;D_2__Armatimonadia
D_0__Bacteria;D_1__Armatimonadetes;D_2__Fimbriimonadia
D_0__Bacteria;D_1__Armatimonadetes;D_2__uncultured
D_0__Bacteria;D_1__BRC1;D_2__uncultured bacterium
D_0__Bacteria;D_1__BRC1;Other
D_0__Bacteria;D_1__Bacteroidetes;D_2__Bacteroidetes BD2-2
D_0__Bacteria;D_1__Bacteroidetes;D_2__Bacteroidetes Incertae Sedis
D_0__Bacteria;D_1__Bacteroidetes;D_2__Bacteroidia
D_0__Bacteria;D_1__Bacteroidetes;D_2__Cytophagia
D_0__Bacteria;D_1__Bacteroidetes;D_2__Flavobacteriia
D_0__Bacteria;D_1__Bacteroidetes;D_2__Sphingobacteriia
D_0__Bacteria;D_1__Chlamydiae;D_2__Chlamydiae
D_0__Bacteria;D_1__Chlorobi;D_2__Chlorobia
D_0__Bacteria;D_1__Chloroflexi;D_2__Anaerolineae
D_0__Bacteria;D_1__Chloroflexi;D_2__Ardenticatenia
D_0__Bacteria;D_1__Chloroflexi;D_2__Caldilineae
D_0__Bacteria;D_1__Chloroflexi;D_2__Chloroflexi Incertae Sedis
D_0__Bacteria;D_1__Chloroflexi;D_2__Chloroflexia
D_0__Bacteria;D_1__Chloroflexi;D_2__Gitt-GS-136
D_0__Bacteria;D_1__Chloroflexi;D_2__JG30-KF-CM66
D_0__Bacteria;D_1__Chloroflexi;D_2__JG37-AG-4
D_0__Bacteria;D_1__Chloroflexi;D_2__KD4-96
D_0__Bacteria;D_1__Chloroflexi;D_2__Ktedonobacteria
D_0__Bacteria;D_1__Chloroflexi;D_2__NLS2-31
D_0__Bacteria;D_1__Chloroflexi;D_2__P2-11E
D_0__Bacteria;D_1__Chloroflexi;D_2__S085
D_0__Bacteria;D_1__Chloroflexi;D_2__SAR202 clade
D_0__Bacteria;D_1__Chloroflexi;D_2__TK10
D_0__Bacteria;D_1__Chloroflexi;D_2__Thermomicrobia
D_0__Bacteria;D_1__Chloroflexi;Other
D_0__Bacteria;D_1__Cyanobacteria;D_2__Chloroplast
D_0__Bacteria;D_1__Cyanobacteria;D_2__Cyanobacteria
D_0__Bacteria;D_1__Cyanobacteria;D_2__Melainabacteria
D_0__Bacteria;D_1__Deinococcus-Thermus;D_2__Deinococci
D_0__Bacteria;D_1__Elusimicrobia;D_2__Elusimicrobia
D_0__Bacteria;D_1__FBP;D_2__uncultured bacterium
D_0__Bacteria;D_1__Fibrobacteres;D_2__Fibrobacteria
D_0__Bacteria;D_1__Firmicutes;D_2__Bacilli
D_0__Bacteria;D_1__Firmicutes;D_2__Clostridia
D_0__Bacteria;D_1__Firmicutes;D_2__Erysipelotrichia
D_0__Bacteria;D_1__Firmicutes;D_2__Negativicutes
D_0__Bacteria;D_1__Fusobacteria;D_2__Fusobacteriia
D_0__Bacteria;D_1__Gemmatimonadetes;D_2__BD2-11 terrestrial group
D_0__Bacteria;D_1__Gemmatimonadetes;D_2__Gemmatimonadetes
D_0__Bacteria;D_1__Gemmatimonadetes;D_2__Longimicrobia
D_0__Bacteria;D_1__Gemmatimonadetes;D_2__S0134 terrestrial group
D_0__Bacteria;D_1__Hydrogenedentes;D_2__uncultured bacterium
D_0__Bacteria;D_1__Nitrospirae;D_2__Nitrospira
D_0__Bacteria;D_1__Planctomycetes;D_2__Phycisphaerae
D_0__Bacteria;D_1__Planctomycetes;D_2__Planctomycetacia
D_0__Bacteria;D_1__Proteobacteria;D_2__Alphaproteobacteria
D_0__Bacteria;D_1__Proteobacteria;D_2__Betaproteobacteria
D_0__Bacteria;D_1__Proteobacteria;D_2__Deltaproteobacteria
D_0__Bacteria;D_1__Proteobacteria;D_2__Epsilonproteobacteria
D_0__Bacteria;D_1__Proteobacteria;D_2__Gammaproteobacteria
D_0__Bacteria;D_1__Proteobacteria;D_2__JTB23
D_0__Bacteria;D_1__Proteobacteria;D_2__SPOTSOCT00m83
D_0__Bacteria;D_1__Proteobacteria;Other
D_0__Bacteria;D_1__SBR1093;Other
D_0__Bacteria;D_1__SR1 (Absconditabacteria);Ambiguous_taxa
D_0__Bacteria;D_1__SR1 (Absconditabacteria);D_2__uncultured bacterium
D_0__Bacteria;D_1__Saccharibacteria;D_2__uncultured bacterium
D_0__Bacteria;D_1__Saccharibacteria;Other
D_0__Bacteria;D_1__Spirochaetae;D_2__Spirochaetes
D_0__Bacteria;D_1__Synergistetes;D_2__Synergistia
D_0__Bacteria;D_1__TM6 (Dependentiae);Other
D_0__Bacteria;D_1__Tectomicrobia;D_2__Tectomicrobia Incertae Sedis
D_0__Bacteria;D_1__Tectomicrobia;D_2__uncultured bacterium
D_0__Bacteria;D_1__Verrucomicrobia;D_2__OPB35 soil group
D_0__Bacteria;D_1__Verrucomicrobia;D_2__Opitutae
D_0__Bacteria;D_1__Verrucomicrobia;D_2__Spartobacteria
D_0__Bacteria;D_1__Verrucomicrobia;D_2__Verrucomicrobiae
D_0__Bacteria;Other;Other
Unassigned;Other;Other
