# Supplementary figures and images for: Cretaceous dinosaur bone contains recent organic material and provides an environment conducive to microbial communities
Source: eLife. 2019 Jun 18;8:e46205. doi: 10.7554/eLife.46205 (PMC6581507; doi:10.7554/eLife.46205)

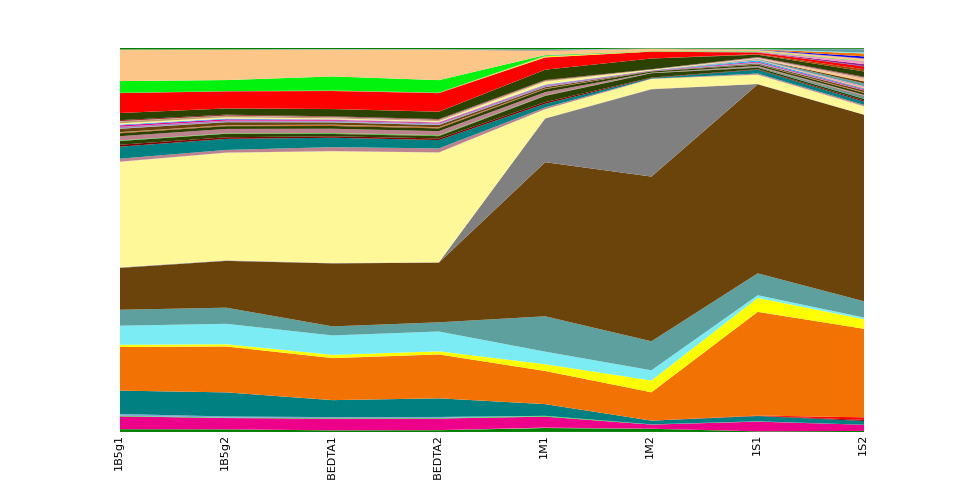

Supplement: Source data 1. [file elife-46205-data1.zip › Raw data files/16S rRNA amplicon sequencing/taxa_plots/taxa_summary_plots/charts/K12p57GwUoS9uilaBWTnHHjbe9AOuC.png]

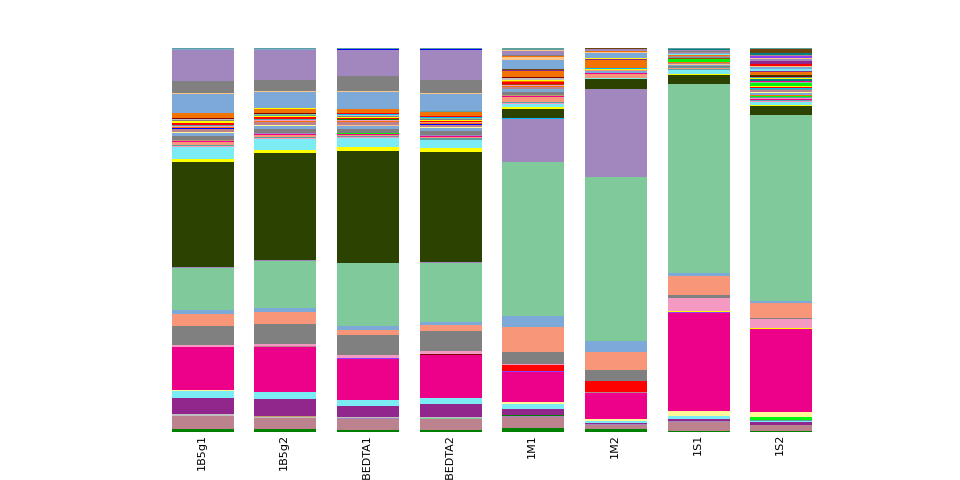

Supplement: Source data 1. [file elife-46205-data1.zip › Raw data files/16S rRNA amplicon sequencing/taxa_plots/taxa_summary_plots/charts/W0Bdpiet5GDuHKiqJfxdaehoF0kwpF.png]

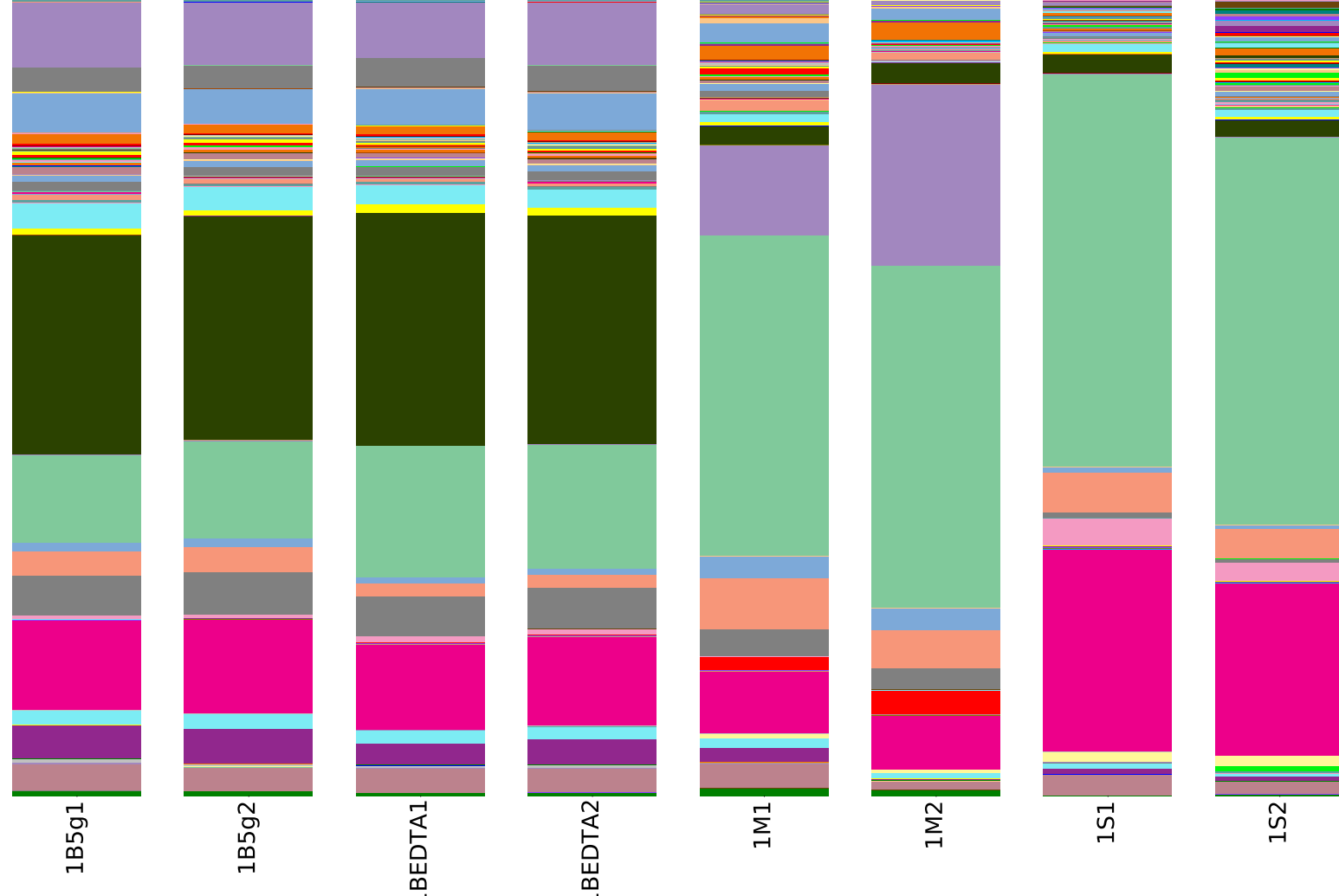

Supplement: Source data 1. [file elife-46205-data1.zip › Raw data files/16S rRNA amplicon sequencing/taxa_plots/taxa_summary_plots/charts/E0AG9cZC0Arz6yxKBxOmhdQsOQkoCs.pdf]

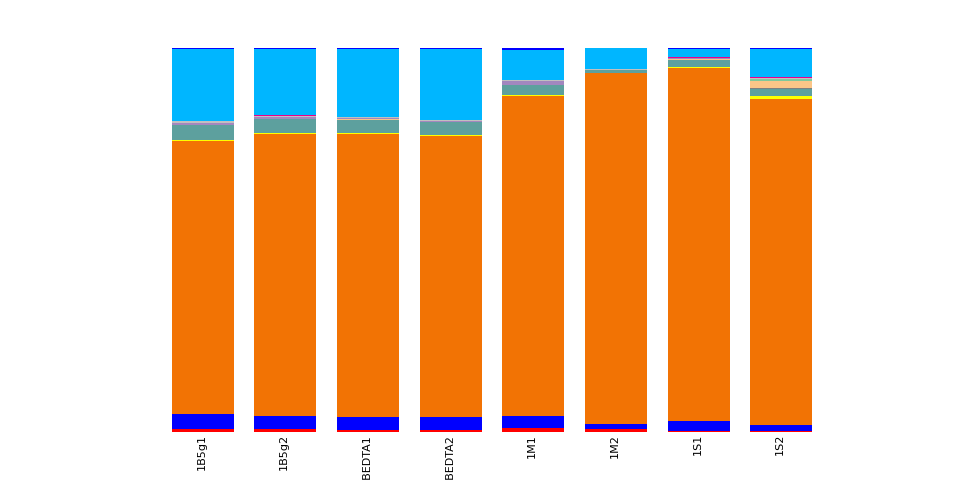

Supplement: Source data 1. [file elife-46205-data1.zip › Raw data files/16S rRNA amplicon sequencing/taxa_plots/taxa_summary_plots/charts/6K1nz3jkX0HaQBDEz05OTJ5DUGk7sX.png]

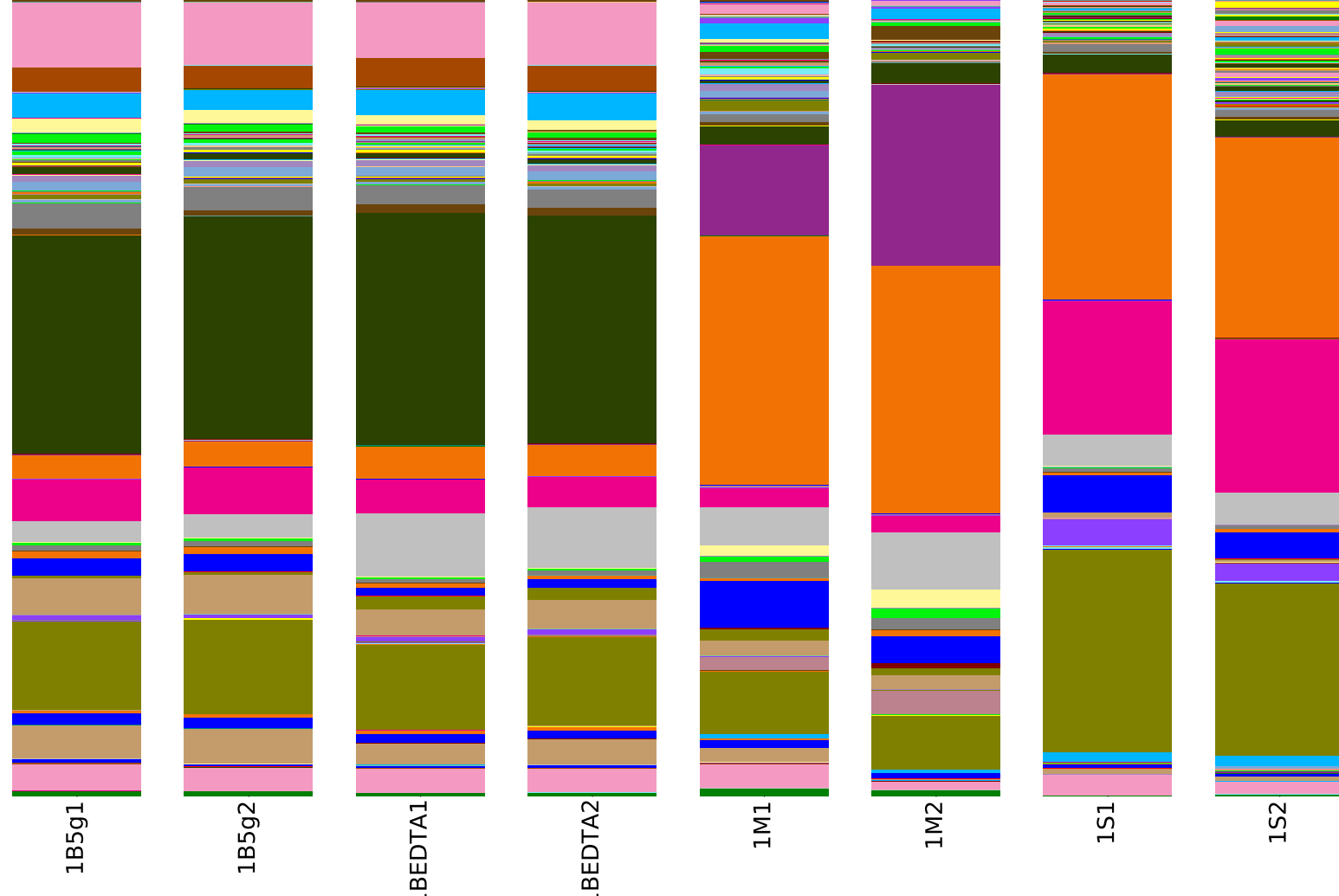

Supplement: Source data 1. [file elife-46205-data1.zip › Raw data files/16S rRNA amplicon sequencing/taxa_plots/taxa_summary_plots/charts/ft02L2EaRUIZi2ShZW5suWQy4RoK0n.pdf]

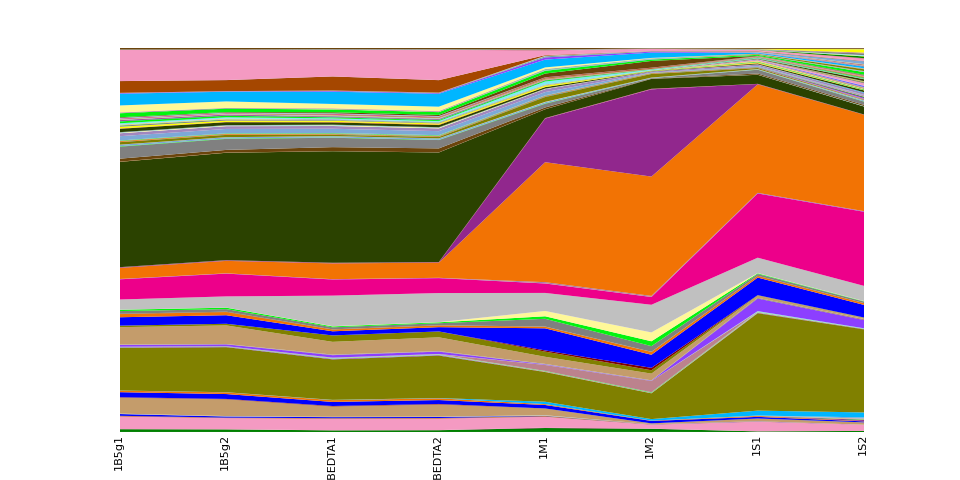

Supplement: Source data 1. [file elife-46205-data1.zip › Raw data files/16S rRNA amplicon sequencing/taxa_plots/taxa_summary_plots/charts/1jzZ6e9rzMY3toQzE7srFX0qCUAZJt.png]

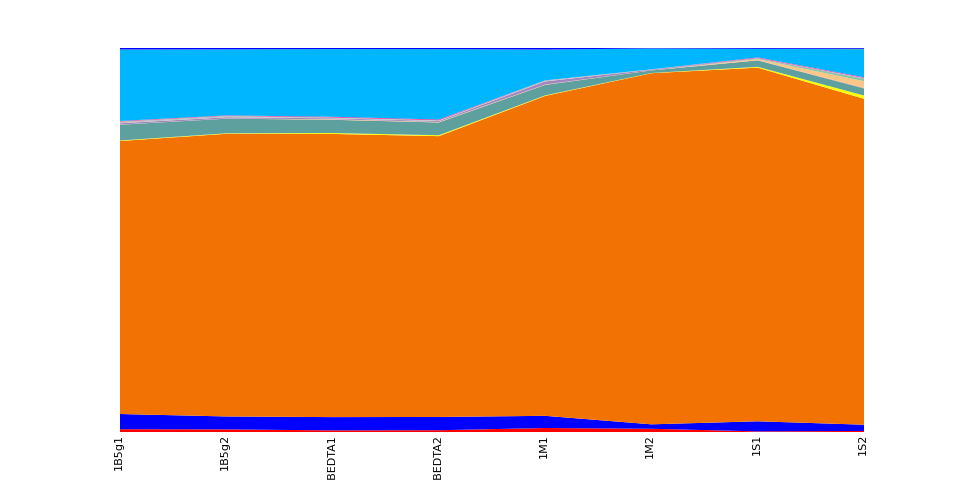

Supplement: Source data 1. [file elife-46205-data1.zip › Raw data files/16S rRNA amplicon sequencing/taxa_plots/taxa_summary_plots/charts/gxK9FzeGKWFAIYftR4ahQS0dQ14ztj.png]

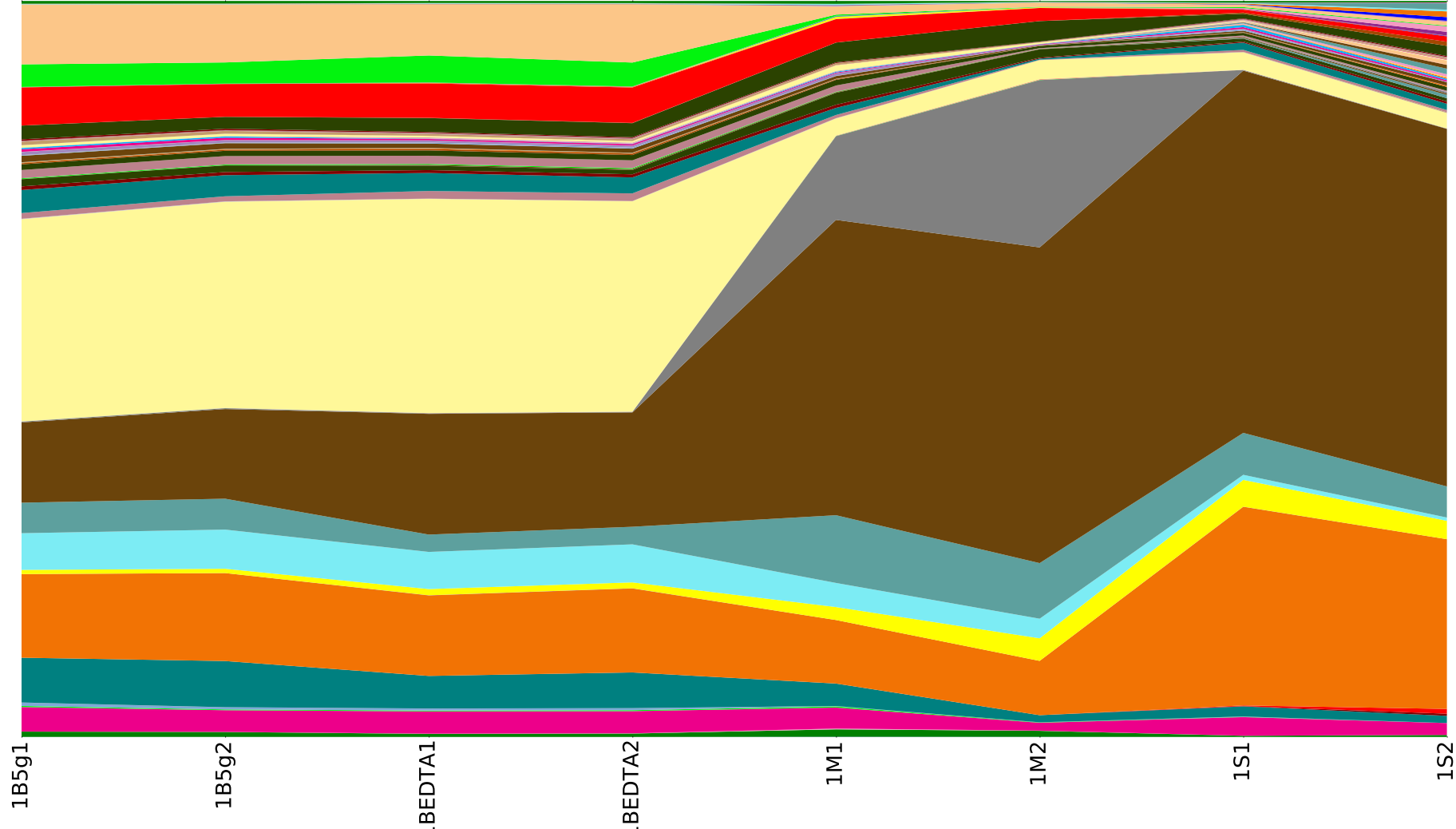

Supplement: Source data 1. [file elife-46205-data1.zip › Raw data files/16S rRNA amplicon sequencing/taxa_plots/taxa_summary_plots/charts/6UxUCpKNYbo4CZwTH0h0ONjGEm6DfE.pdf]

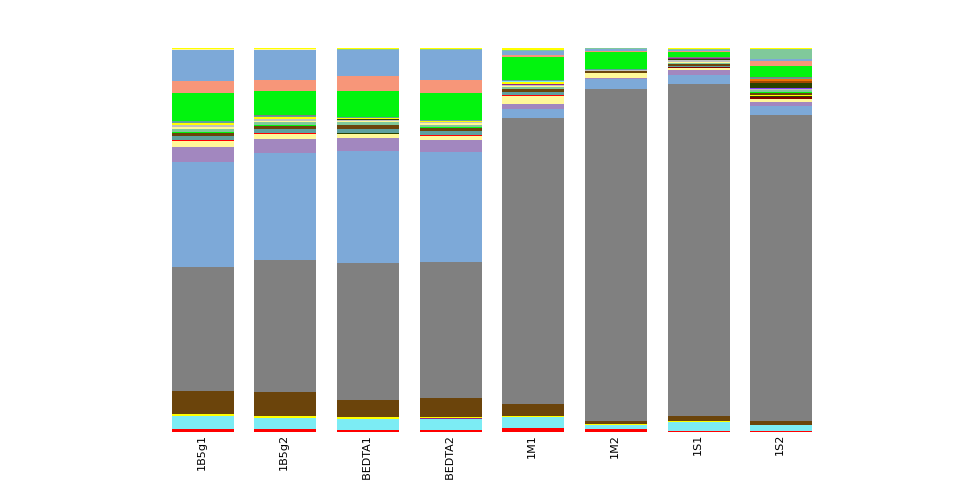

Supplement: Source data 1. [file elife-46205-data1.zip › Raw data files/16S rRNA amplicon sequencing/taxa_plots/taxa_summary_plots/charts/T9PGFLXnffcL5FMe5EzY1HiQ2bJGBA.png]

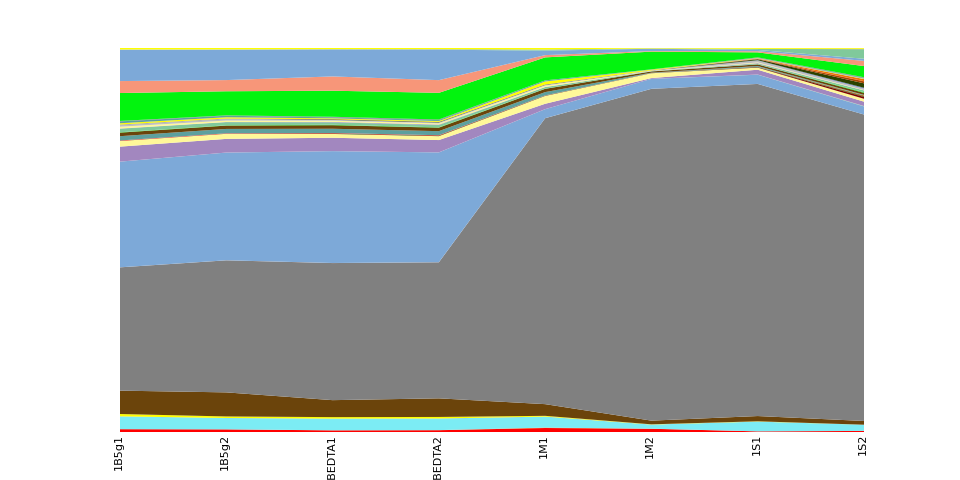

Supplement: Source data 1. [file elife-46205-data1.zip › Raw data files/16S rRNA amplicon sequencing/taxa_plots/taxa_summary_plots/charts/yzD0FdKE7haGHk6I12wafU7EJ2DpoP.png]

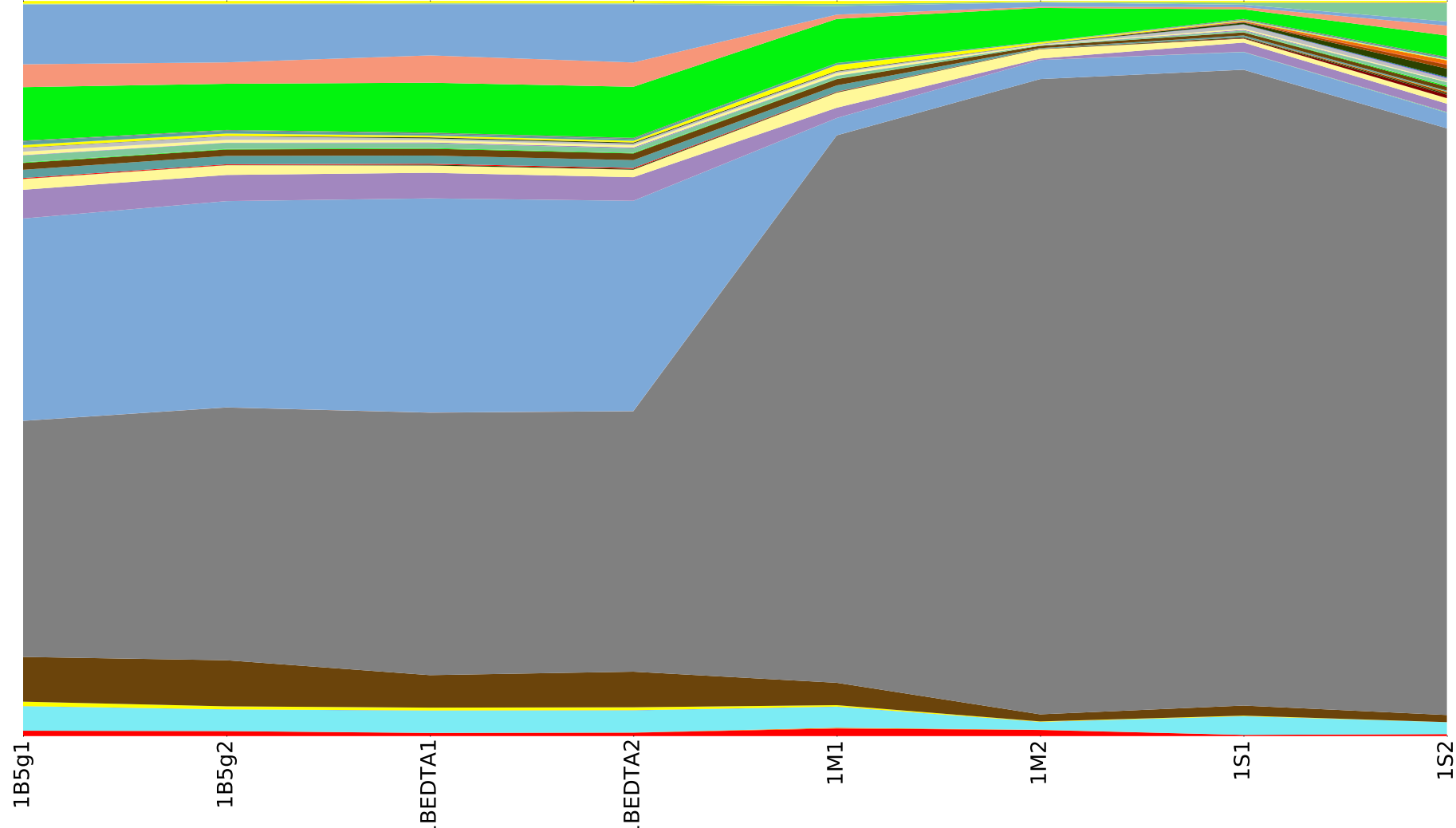

Supplement: Source data 1. [file elife-46205-data1.zip › Raw data files/16S rRNA amplicon sequencing/taxa_plots/taxa_summary_plots/charts/iOOHUmxtC0Icjw0RgsKMmbxXJlRUJF.pdf]

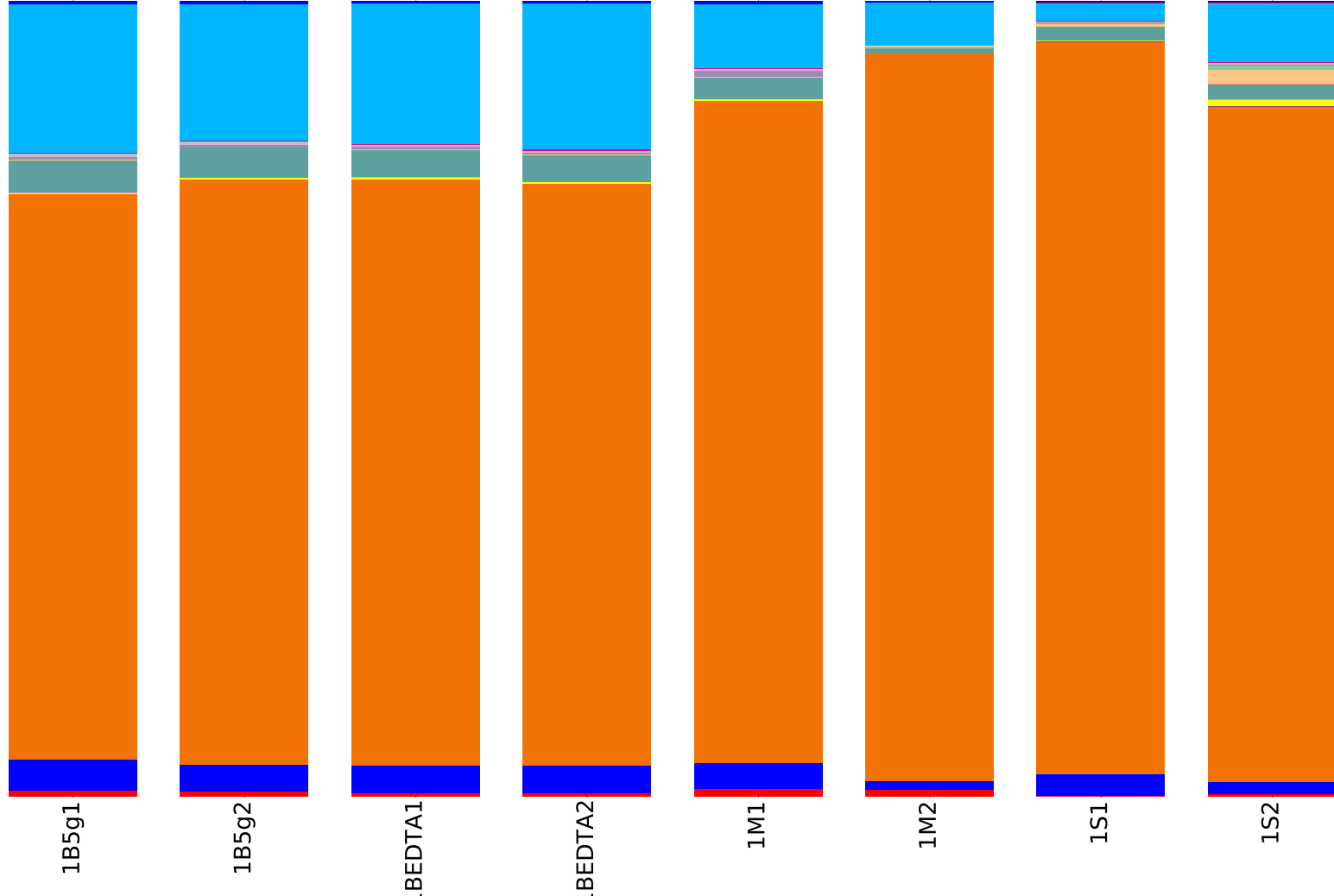

Supplement: Source data 1. [file elife-46205-data1.zip › Raw data files/16S rRNA amplicon sequencing/taxa_plots/taxa_summary_plots/charts/Lmseg8CsClUz3ZO50AQod7zSc2BPr3.pdf]

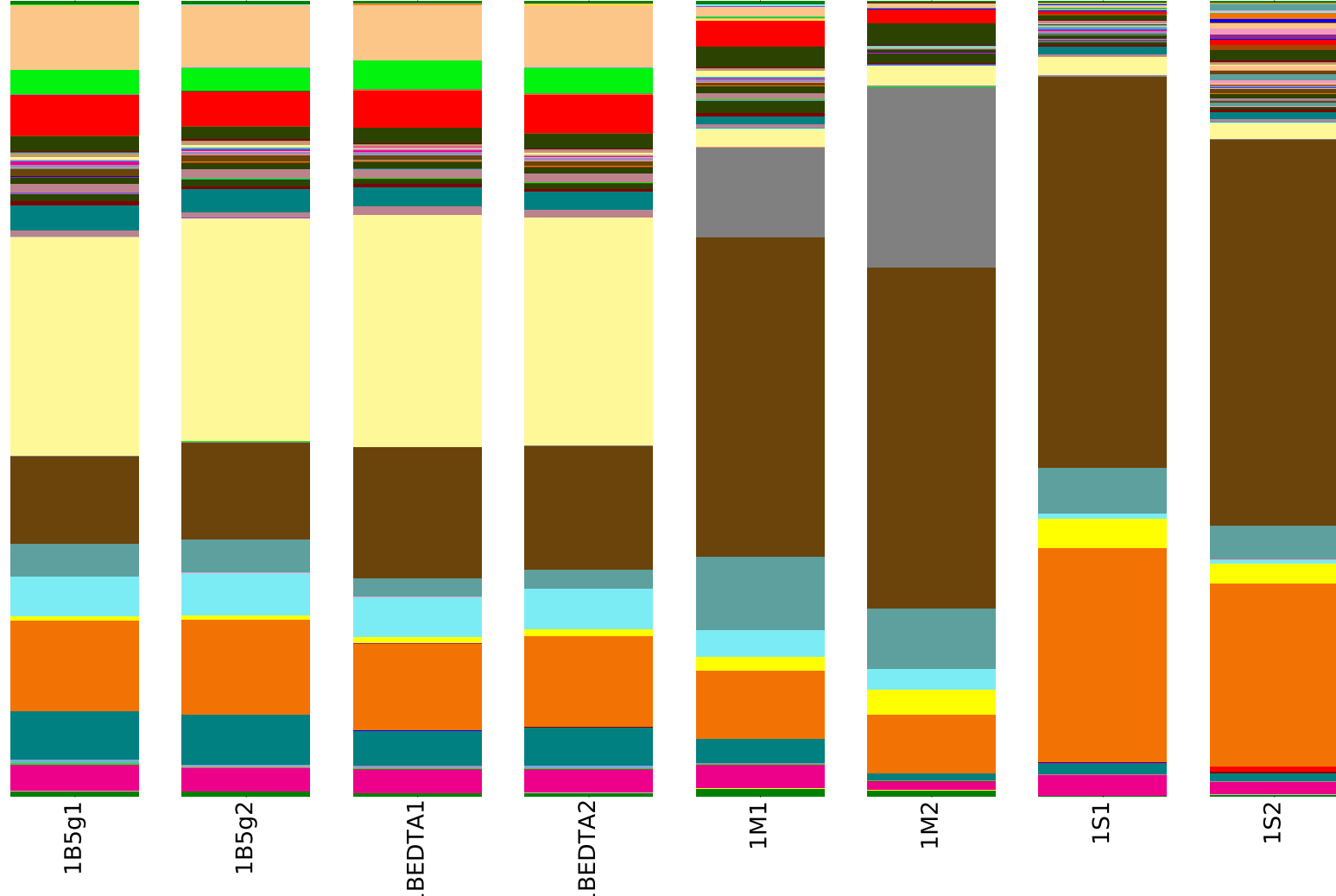

Supplement: Source data 1. [file elife-46205-data1.zip › Raw data files/16S rRNA amplicon sequencing/taxa_plots/taxa_summary_plots/charts/kWRwnPHOBp3nLCG7onHrs2hycd4l0f.pdf]

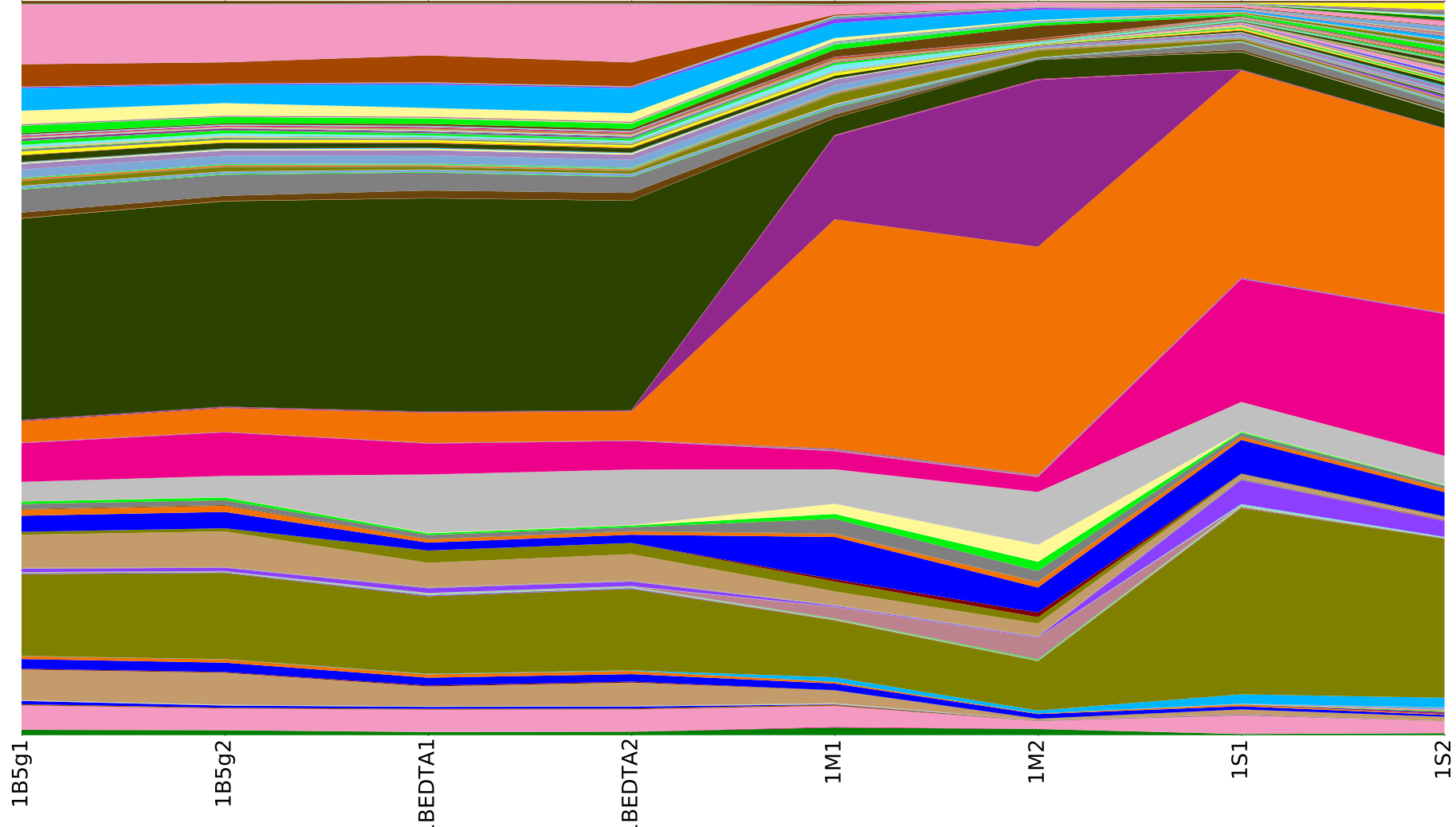

Supplement: Source data 1. [file elife-46205-data1.zip › Raw data files/16S rRNA amplicon sequencing/taxa_plots/taxa_summary_plots/charts/8zKnZQ9U10QMhxZnI4a5p04L0GAXmJ.pdf]

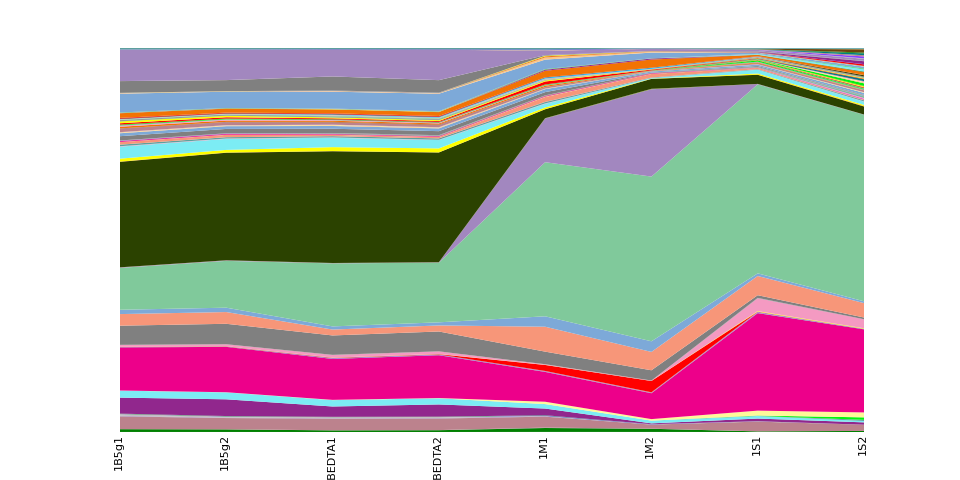

Supplement: Source data 1. [file elife-46205-data1.zip › Raw data files/16S rRNA amplicon sequencing/taxa_plots/taxa_summary_plots/charts/z6HP8zUbAyB0orEuCPPE0DWsj2i5Kl.png]

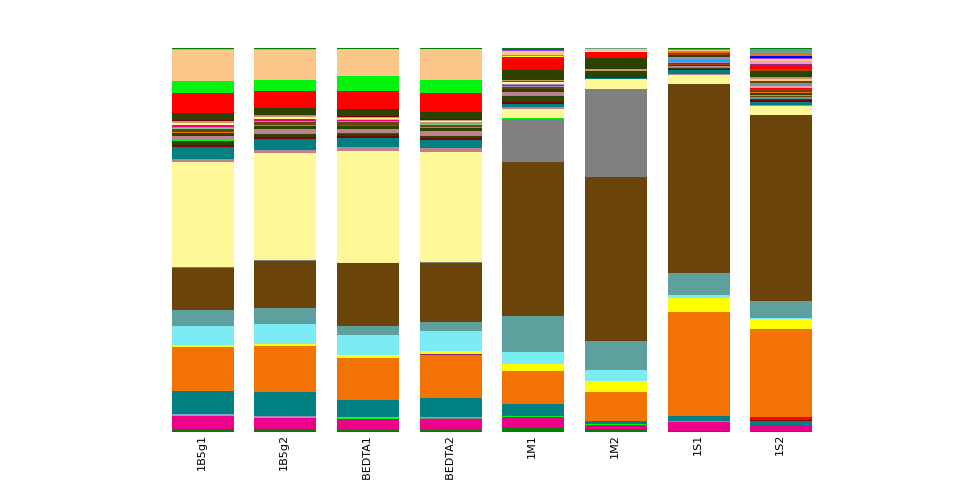

Supplement: Source data 1. [file elife-46205-data1.zip › Raw data files/16S rRNA amplicon sequencing/taxa_plots/taxa_summary_plots/charts/dWkku6eU9QMS2QIF7j6QSIU0WmUFeH.png]

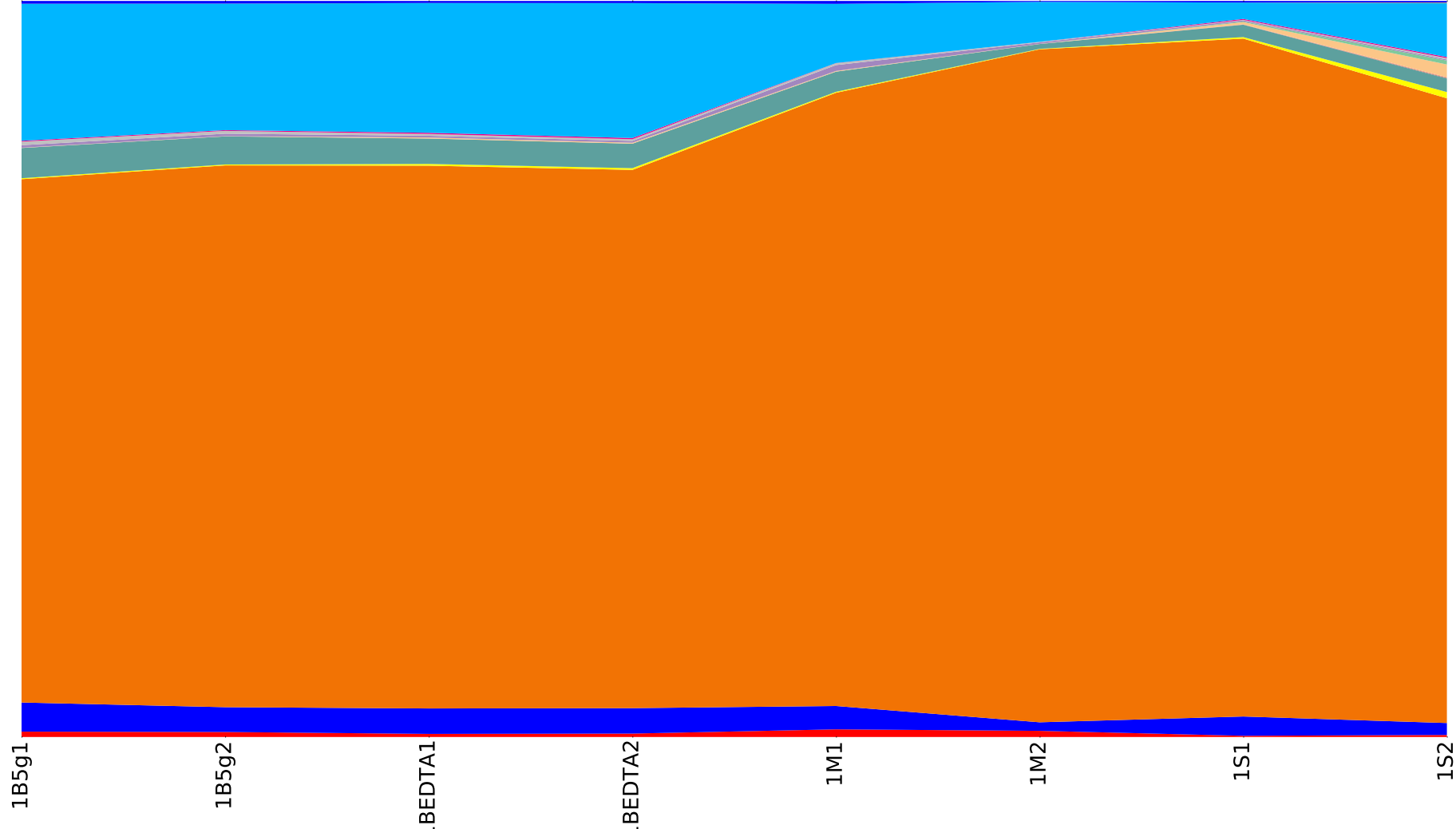

Supplement: Source data 1. [file elife-46205-data1.zip › Raw data files/16S rRNA amplicon sequencing/taxa_plots/taxa_summary_plots/charts/JWg6MSlZXm2IgbYBaSyfignZof7ERC.pdf]

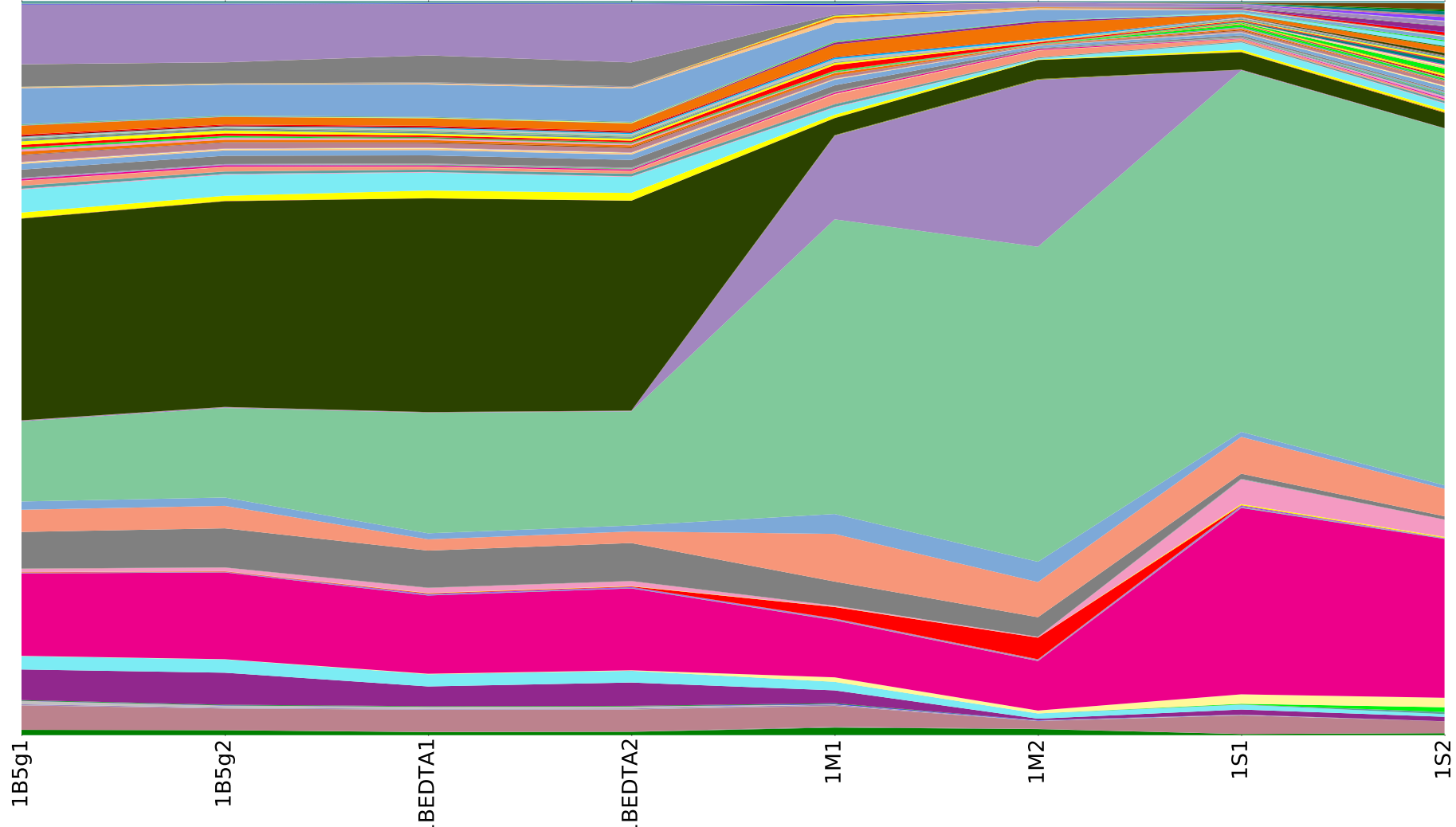

Supplement: Source data 1. [file elife-46205-data1.zip › Raw data files/16S rRNA amplicon sequencing/taxa_plots/taxa_summary_plots/charts/RjUBFqyk3YiP68S3fuDeLtEQzOqJMw.pdf]

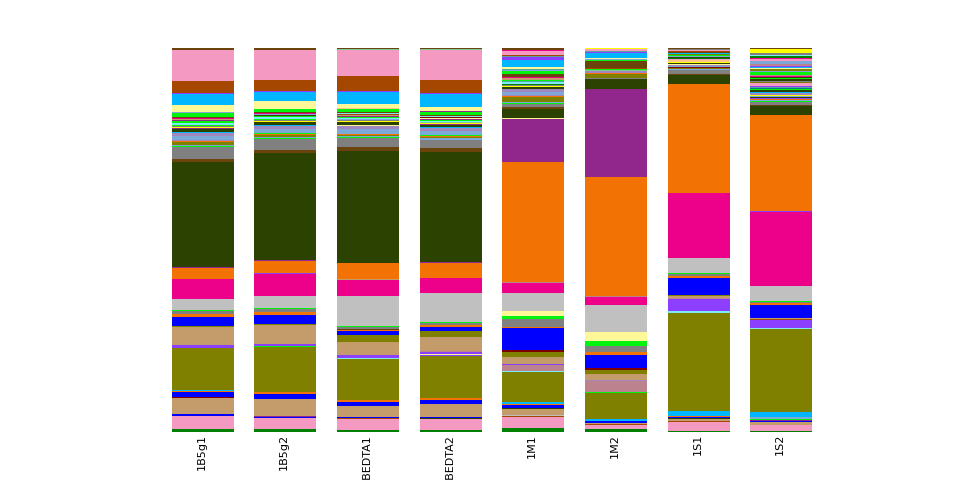

Supplement: Source data 1. [file elife-46205-data1.zip › Raw data files/16S rRNA amplicon sequencing/taxa_plots/taxa_summary_plots/charts/wawZLqFWDUTKKmJZF0o01JIZ1F95ze.png]

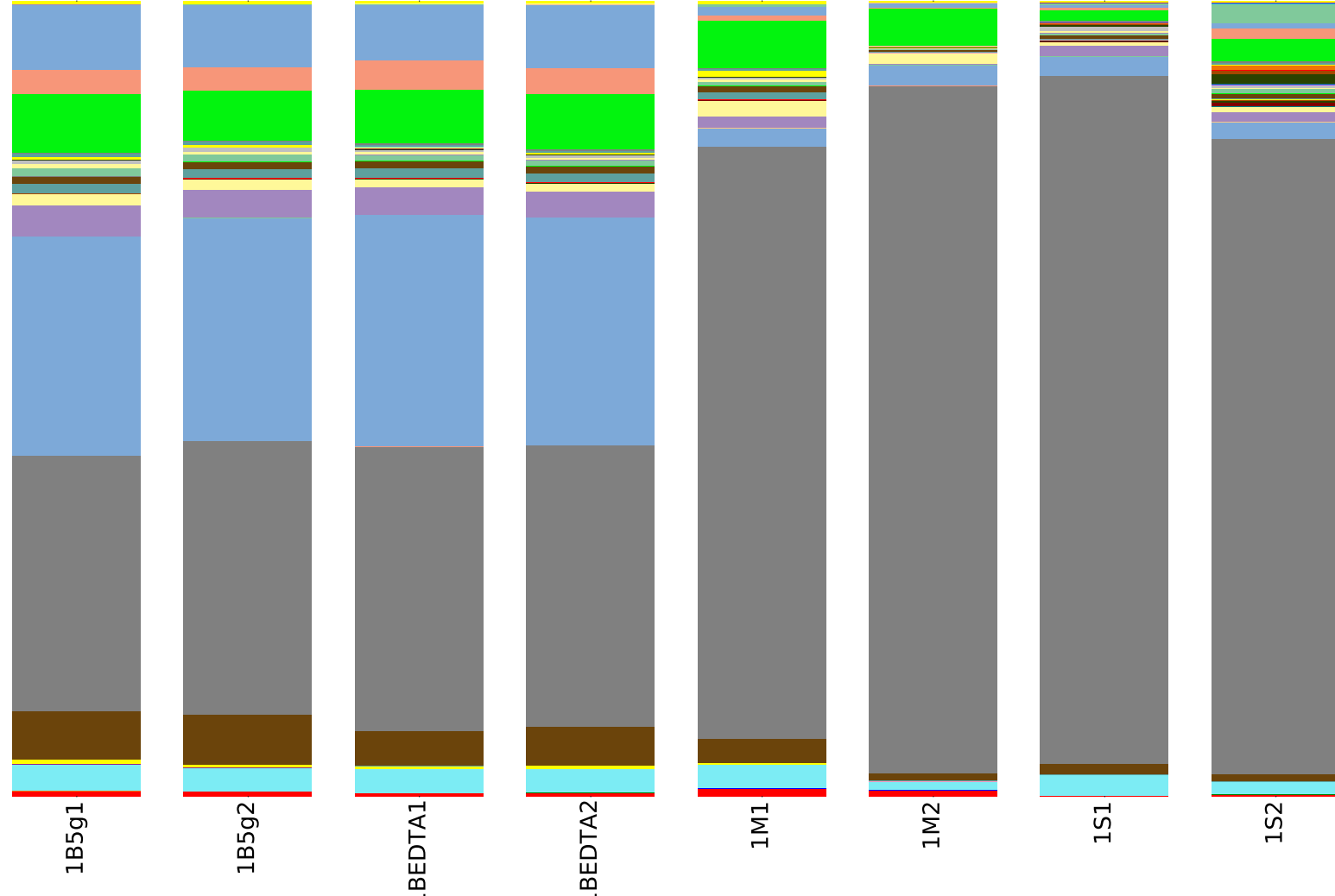

Supplement: Source data 1. [file elife-46205-data1.zip › Raw data files/16S rRNA amplicon sequencing/taxa_plots/taxa_summary_plots/charts/UIF8A3PmC7OZZLj4y1PKYYl34oy2ZU.pdf]

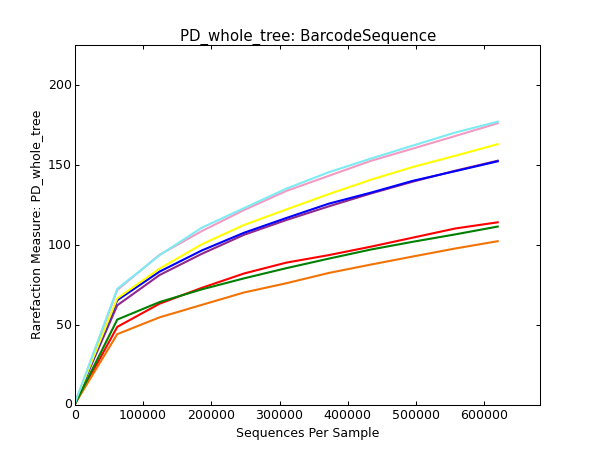

Supplement: Source data 1. [file elife-46205-data1.zip › Raw data files/16S rRNA amplicon sequencing/arare_max620000/alpha_rarefaction_plots/average_plots/PD_whole_treeBarcodeSequence.png]

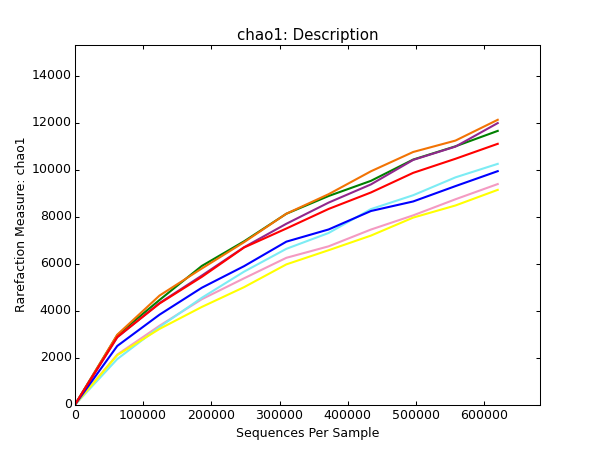

Supplement: Source data 1. [file elife-46205-data1.zip › Raw data files/16S rRNA amplicon sequencing/arare_max620000/alpha_rarefaction_plots/average_plots/chao1Description.png]

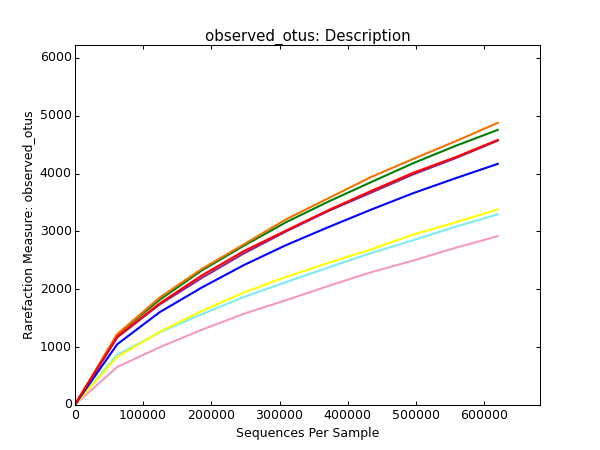

Supplement: Source data 1. [file elife-46205-data1.zip › Raw data files/16S rRNA amplicon sequencing/arare_max620000/alpha_rarefaction_plots/average_plots/observed_otusDescription.png]

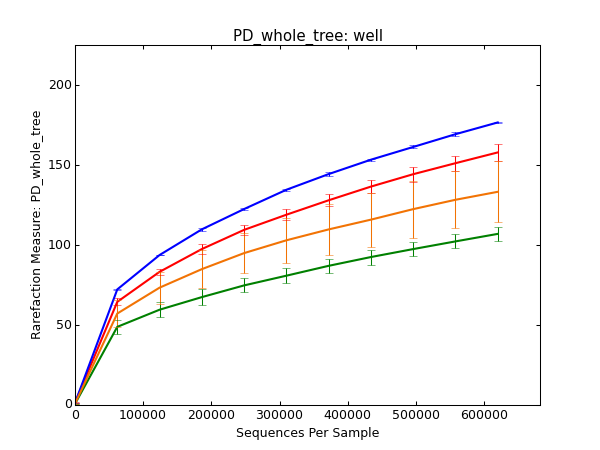

Supplement: Source data 1. [file elife-46205-data1.zip › Raw data files/16S rRNA amplicon sequencing/arare_max620000/alpha_rarefaction_plots/average_plots/PD_whole_treewell.png]

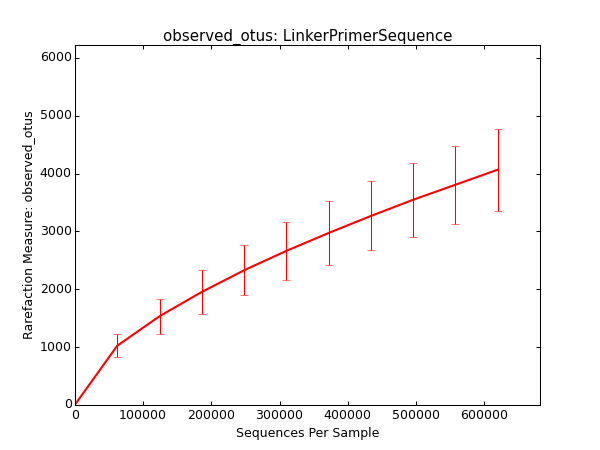

Supplement: Source data 1. [file elife-46205-data1.zip › Raw data files/16S rRNA amplicon sequencing/arare_max620000/alpha_rarefaction_plots/average_plots/observed_otusLinkerPrimerSequence.png]

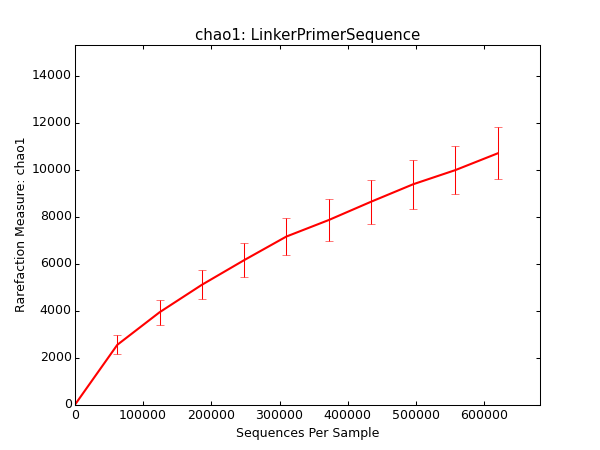

Supplement: Source data 1. [file elife-46205-data1.zip › Raw data files/16S rRNA amplicon sequencing/arare_max620000/alpha_rarefaction_plots/average_plots/chao1LinkerPrimerSequence.png]

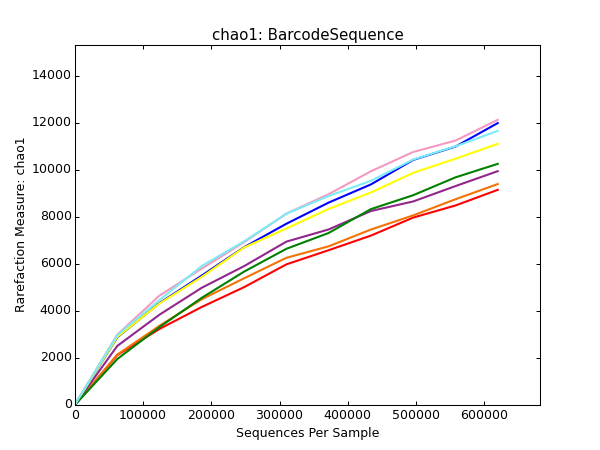

Supplement: Source data 1. [file elife-46205-data1.zip › Raw data files/16S rRNA amplicon sequencing/arare_max620000/alpha_rarefaction_plots/average_plots/chao1BarcodeSequence.png]

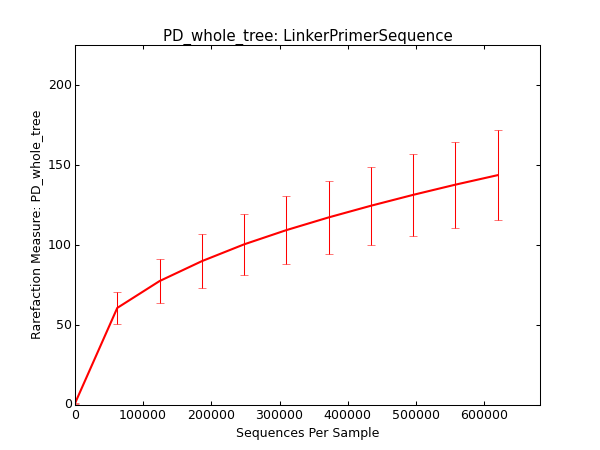

Supplement: Source data 1. [file elife-46205-data1.zip › Raw data files/16S rRNA amplicon sequencing/arare_max620000/alpha_rarefaction_plots/average_plots/PD_whole_treeLinkerPrimerSequence.png]

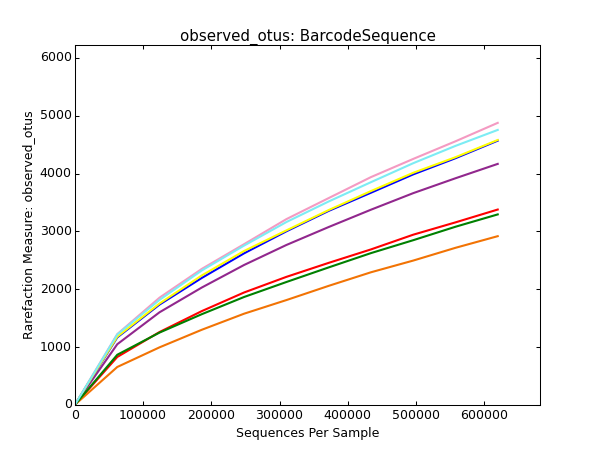

Supplement: Source data 1. [file elife-46205-data1.zip › Raw data files/16S rRNA amplicon sequencing/arare_max620000/alpha_rarefaction_plots/average_plots/observed_otusBarcodeSequence.png]

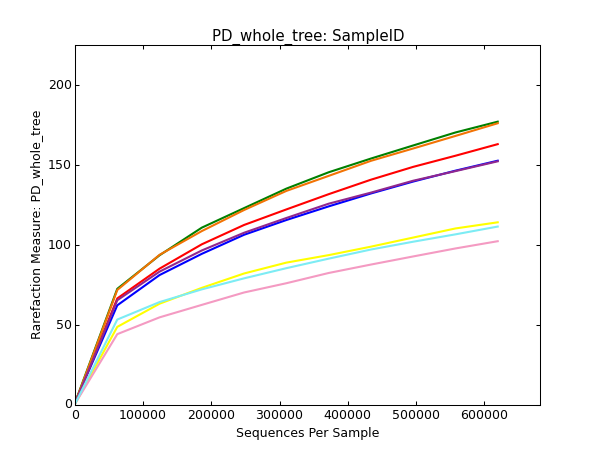

Supplement: Source data 1. [file elife-46205-data1.zip › Raw data files/16S rRNA amplicon sequencing/arare_max620000/alpha_rarefaction_plots/average_plots/PD_whole_treeSampleID.png]

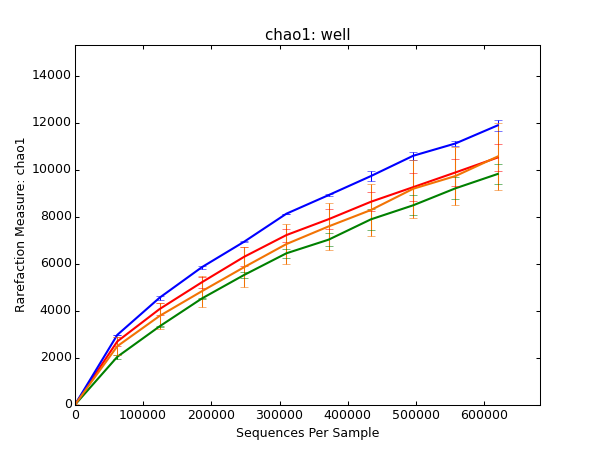

Supplement: Source data 1. [file elife-46205-data1.zip › Raw data files/16S rRNA amplicon sequencing/arare_max620000/alpha_rarefaction_plots/average_plots/chao1well.png]

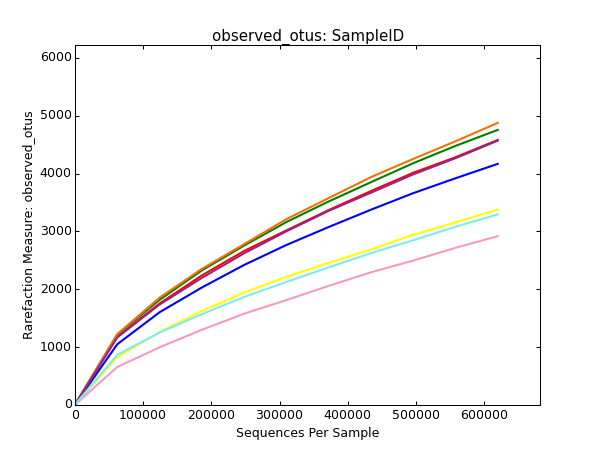

Supplement: Source data 1. [file elife-46205-data1.zip › Raw data files/16S rRNA amplicon sequencing/arare_max620000/alpha_rarefaction_plots/average_plots/observed_otusSampleID.png]

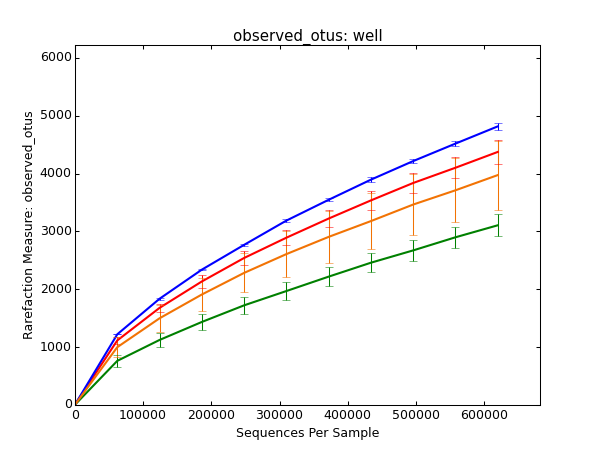

Supplement: Source data 1. [file elife-46205-data1.zip › Raw data files/16S rRNA amplicon sequencing/arare_max620000/alpha_rarefaction_plots/average_plots/observed_otuswell.png]

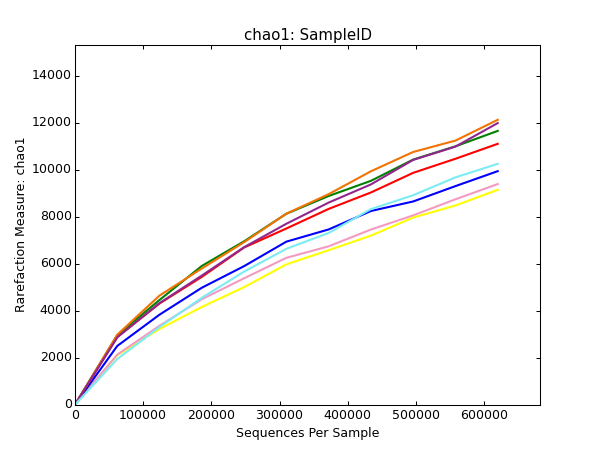

Supplement: Source data 1. [file elife-46205-data1.zip › Raw data files/16S rRNA amplicon sequencing/arare_max620000/alpha_rarefaction_plots/average_plots/chao1SampleID.png]

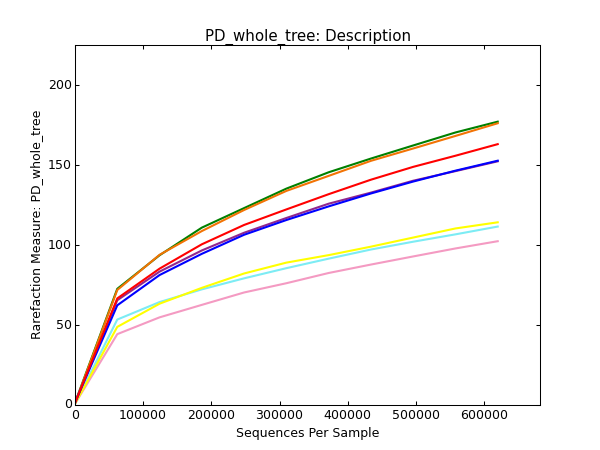

Supplement: Source data 1. [file elife-46205-data1.zip › Raw data files/16S rRNA amplicon sequencing/arare_max620000/alpha_rarefaction_plots/average_plots/PD_whole_treeDescription.png]

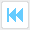

Supplement: Source data 1. [file elife-46205-data1.zip › Raw data files/16S rRNA amplicon sequencing/bdiv_even620000/weighted_unifrac_emperor_pcoa_plot/emperor_required_resources/img/reset.png]

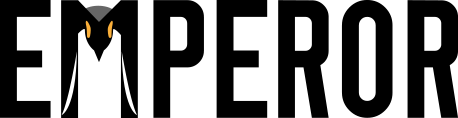

Supplement: Source data 1. [file elife-46205-data1.zip › Raw data files/16S rRNA amplicon sequencing/bdiv_even620000/weighted_unifrac_emperor_pcoa_plot/emperor_required_resources/img/emperor.png]

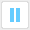

Supplement: Source data 1. [file elife-46205-data1.zip › Raw data files/16S rRNA amplicon sequencing/bdiv_even620000/weighted_unifrac_emperor_pcoa_plot/emperor_required_resources/img/pause.png]

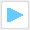

Supplement: Source data 1. [file elife-46205-data1.zip › Raw data files/16S rRNA amplicon sequencing/bdiv_even620000/weighted_unifrac_emperor_pcoa_plot/emperor_required_resources/img/play.png]

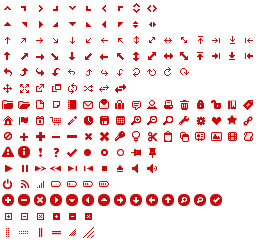

Supplement: Source data 1. [file elife-46205-data1.zip › Raw data files/16S rRNA amplicon sequencing/bdiv_even620000/weighted_unifrac_emperor_pcoa_plot/emperor_required_resources/css/images/ui-icons_cd0a0a_256x240.png]

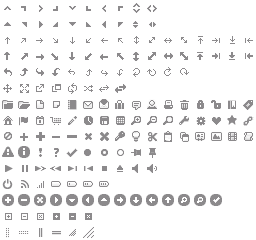

Supplement: Source data 1. [file elife-46205-data1.zip › Raw data files/16S rRNA amplicon sequencing/bdiv_even620000/weighted_unifrac_emperor_pcoa_plot/emperor_required_resources/css/images/ui-icons_888888_256x240.png]

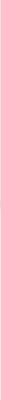

Supplement: Source data 1. [file elife-46205-data1.zip › Raw data files/16S rRNA amplicon sequencing/bdiv_even620000/weighted_unifrac_emperor_pcoa_plot/emperor_required_resources/css/images/ui-bg_glass_75_dadada_1x400.png]

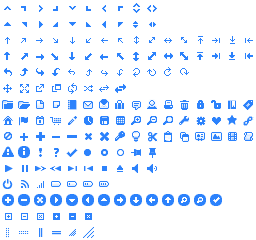

Supplement: Source data 1. [file elife-46205-data1.zip › Raw data files/16S rRNA amplicon sequencing/bdiv_even620000/weighted_unifrac_emperor_pcoa_plot/emperor_required_resources/css/images/ui-icons_2e83ff_256x240.png]

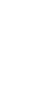

Supplement: Source data 1. [file elife-46205-data1.zip › Raw data files/16S rRNA amplicon sequencing/bdiv_even620000/weighted_unifrac_emperor_pcoa_plot/emperor_required_resources/css/images/ui-bg_flat_75_ffffff_40x100.png]

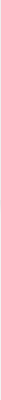

Supplement: Source data 1. [file elife-46205-data1.zip › Raw data files/16S rRNA amplicon sequencing/bdiv_even620000/weighted_unifrac_emperor_pcoa_plot/emperor_required_resources/css/images/ui-bg_glass_75_e6e6e6_1x400.png]

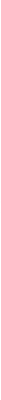

Supplement: Source data 1. [file elife-46205-data1.zip › Raw data files/16S rRNA amplicon sequencing/bdiv_even620000/weighted_unifrac_emperor_pcoa_plot/emperor_required_resources/css/images/ui-bg_glass_65_ffffff_1x400.png]

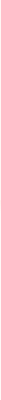

Supplement: Source data 1. [file elife-46205-data1.zip › Raw data files/16S rRNA amplicon sequencing/bdiv_even620000/weighted_unifrac_emperor_pcoa_plot/emperor_required_resources/css/images/ui-bg_glass_95_fef1ec_1x400.png]

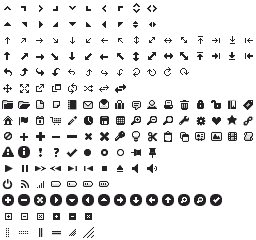

Supplement: Source data 1. [file elife-46205-data1.zip › Raw data files/16S rRNA amplicon sequencing/bdiv_even620000/weighted_unifrac_emperor_pcoa_plot/emperor_required_resources/css/images/ui-icons_222222_256x240.png]

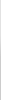

Supplement: Source data 1. [file elife-46205-data1.zip › Raw data files/16S rRNA amplicon sequencing/bdiv_even620000/weighted_unifrac_emperor_pcoa_plot/emperor_required_resources/css/images/ui-bg_highlight-soft_75_cccccc_1x100.png]

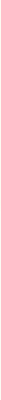

Supplement: Source data 1. [file elife-46205-data1.zip › Raw data files/16S rRNA amplicon sequencing/bdiv_even620000/weighted_unifrac_emperor_pcoa_plot/emperor_required_resources/css/images/ui-bg_glass_55_fbf9ee_1x400.png]

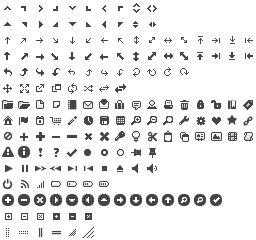

Supplement: Source data 1. [file elife-46205-data1.zip › Raw data files/16S rRNA amplicon sequencing/bdiv_even620000/weighted_unifrac_emperor_pcoa_plot/emperor_required_resources/css/images/ui-icons_454545_256x240.png]

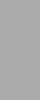

Supplement: Source data 1. [file elife-46205-data1.zip › Raw data files/16S rRNA amplicon sequencing/bdiv_even620000/weighted_unifrac_emperor_pcoa_plot/emperor_required_resources/css/images/ui-bg_flat_0_aaaaaa_40x100.png]
