# Supplementary material for: Cretaceous dinosaur bone contains recent organic material and provides an environment conducive to microbial communities
Source: eLife. 2019 Jun 18;8:e46205. doi: 10.7554/eLife.46205 (PMC6581507; doi:10.7554/eLife.46205)
Supplement: Source data 1. [file W0Bdpiet5GDuHKiqJfxdaehoF0kwpF_legend.pdf › Raw data files/16S rRNA amplicon sequencing/taxa_plots/taxa_summary_plots/charts/W0Bdpiet5GDuHKiqJfxdaehoF0kwpF_legend.pdf]

D_0__Archaea;D_1__Thaumarchaeota;D_2__Soil Crenarchaeotic Group(SCG);D_3__Unknown Order;D_4__Unknown Family
D_0__Archaea;D_1__Thaumarchaeota;D_2__Soil Crenarchaeotic Group(SCG);D_3__uncultured archaeon;D_4__uncultured archaeon
D_0__Archaea;D_1__Thaumarchaeota;D_2__Soil Crenarchaeotic Group(SCG);D_3__uncultured euryarchaeote;D_4__uncultured euryarchaeote
D_0__Archaea;D_1__Thaumarchaeota;D_2__Soil Crenarchaeotic Group(SCG);Other;Other
D_0__Bacteria;D_1__Acidobacteria;D_2__Blastocatellia;D_3__Blastocatellales;D_4__Blastocatellaceae (Subgroup 4)
D_0__Bacteria;D_1__Acidobacteria;D_2__Holophagae;D_3__Holophagales;D_4__Holophagaceae
D_0__Bacteria;D_1__Acidobacteria;D_2__Holophagae;D_3__Subgroup 10;D_4__CA002
D_0__Bacteria;D_1__Acidobacteria;D_2__Holophagae;D_3__Subgroup 10;D_4__NS72
D_0__Bacteria;D_1__Acidobacteria;D_2__Holophagae;D_3__Subgroup 10;D_4__Sva0725
D_0__Bacteria;D_1__Acidobacteria;D_2__Holophagae;D_3__Subgroup 10;Other
D_0__Bacteria;D_1__Acidobacteria;D_2__Holophagae;D_3__Subgroup 7;Ambiguous_taxa
D_0__Bacteria;D_1__Acidobacteria;D_2__Holophagae;D_3__Subgroup 7;D_4__uncultured bacterium
D_0__Bacteria;D_1__Acidobacteria;D_2__Holophagae;D_3__Subgroup 7;Other
D_0__Bacteria;D_1__Acidobacteria;D_2__Solibacteres;D_3__Solibacterales;D_4__Solibacteraceae (Subgroup 3)
D_0__Bacteria;D_1__Acidobacteria;D_2__Subgroup 17;Ambiguous_taxa;Ambiguous_taxa
D_0__Bacteria;D_1__Acidobacteria;D_2__Subgroup 17;D_3__uncultured Acidobacteria bacterium;D_4__uncultured Acidobacteria bacterium
D_0__Bacteria;D_1__Acidobacteria;D_2__Subgroup 17;Other;Other
D_0__Bacteria;D_1__Acidobacteria;D_2__Subgroup 22;Ambiguous_taxa;Ambiguous_taxa
D_0__Bacteria;D_1__Acidobacteria;D_2__Subgroup 22;D_3__uncultured Acidobacteria bacterium;D_4__uncultured Acidobacteria bacterium
D_0__Bacteria;D_1__Acidobacteria;D_2__Subgroup 22;D_3__uncultured bacterium;D_4__uncultured bacterium
D_0__Bacteria;D_1__Acidobacteria;D_2__Subgroup 22;Other;Other
D_0__Bacteria;D_1__Acidobacteria;D_2__Subgroup 5;D_3__uncultured bacterium;D_4__uncultured bacterium
D_0__Bacteria;D_1__Acidobacteria;D_2__Subgroup 5;Other;Other
D_0__Bacteria;D_1__Acidobacteria;D_2__Subgroup 6;Ambiguous_taxa;Ambiguous_taxa
D_0__Bacteria;D_1__Acidobacteria;D_2__Subgroup 6;D_3__uncultured Acidobacteria bacterium;D_4__uncultured Acidobacteria bacterium
D_0__Bacteria;D_1__Acidobacteria;D_2__Subgroup 6;D_3__uncultured bacterium;D_4__uncultured bacterium
D_0__Bacteria;D_1__Acidobacteria;D_2__Subgroup 6;D_3__uncultured organism;D_4__uncultured organism
D_0__Bacteria;D_1__Acidobacteria;D_2__Subgroup 6;D_3__uncultured soil bacterium;D_4__uncultured soil bacterium
D_0__Bacteria;D_1__Acidobacteria;D_2__Subgroup 6;Other;Other
D_0__Bacteria;D_1__Acidobacteria;Other;Other;Other
D_0__Bacteria;D_1__Actinobacteria;D_2__Acidimicrobiia;D_3__Acidimicrobiales;D_4__Acidimicrobiaceae
D_0__Bacteria;D_1__Actinobacteria;D_2__Acidimicrobiia;D_3__Acidimicrobiales;D_4__Acidimicrobiales Incertae Sedis
D_0__Bacteria;D_1__Actinobacteria;D_2__Acidimicrobiia;D_3__Acidimicrobiales;D_4__Iamiaceae
D_0__Bacteria;D_1__Actinobacteria;D_2__Acidimicrobiia;D_3__Acidimicrobiales;D_4__OM1 clade
D_0__Bacteria;D_1__Actinobacteria;D_2__Acidimicrobiia;D_3__Acidimicrobiales;D_4__Sva0996 marine group
D_0__Bacteria;D_1__Actinobacteria;D_2__Acidimicrobiia;D_3__Acidimicrobiales;D_4__uncultured
D_0__Bacteria;D_1__Actinobacteria;D_2__Acidimicrobiia;D_3__Acidimicrobiales;Other
D_0__Bacteria;D_1__Actinobacteria;D_2__Actinobacteria;D_3__Actinomycetales;D_4__Actinomycetaceae
D_0__Bacteria;D_1__Actinobacteria;D_2__Actinobacteria;D_3__Actinopolysporales;D_4__Actinopolysporaceae
D_0__Bacteria;D_1__Actinobacteria;D_2__Actinobacteria;D_3__Bifidobacteriales;D_4__Bifidobacteriaceae
D_0__Bacteria;D_1__Actinobacteria;D_2__Actinobacteria;D_3__Corynebacteriales;D_4__Corynebacteriaceae
D_0__Bacteria;D_1__Actinobacteria;D_2__Actinobacteria;D_3__Corynebacteriales;D_4__Dietziaceae
D_0__Bacteria;D_1__Actinobacteria;D_2__Actinobacteria;D_3__Corynebacteriales;D_4__Mycobacteriaceae
D_0__Bacteria;D_1__Actinobacteria;D_2__Actinobacteria;D_3__Corynebacteriales;D_4__Nocardiaceae
D_0__Bacteria;D_1__Actinobacteria;D_2__Actinobacteria;D_3__Corynebacteriales;Other
D_0__Bacteria;D_1__Actinobacteria;D_2__Actinobacteria;D_3__Elev-16S-976;D_4__uncultured Nakamurellaceae bacterium
D_0__Bacteria;D_1__Actinobacteria;D_2__Actinobacteria;D_3__Frankiales;D_4__Acidothermaceae
D_0__Bacteria;D_1__Actinobacteria;D_2__Actinobacteria;D_3__Frankiales;D_4__Cryptosporangiaceae
D_0__Bacteria;D_1__Actinobacteria;D_2__Actinobacteria;D_3__Frankiales;D_4__Frankiaceae
D_0__Bacteria;D_1__Actinobacteria;D_2__Actinobacteria;D_3__Frankiales;D_4__Geodermatophilaceae
D_0__Bacteria;D_1__Actinobacteria;D_2__Actinobacteria;D_3__Frankiales;D_4__Nakamurellaceae
D_0__Bacteria;D_1__Actinobacteria;D_2__Actinobacteria;D_3__Frankiales;D_4__Sporichthyaceae
D_0__Bacteria;D_1__Actinobacteria;D_2__Actinobacteria;D_3__Frankiales;D_4__uncultured
D_0__Bacteria;D_1__Actinobacteria;D_2__Actinobacteria;D_3__Frankiales;Other
D_0__Bacteria;D_1__Actinobacteria;D_2__Actinobacteria;D_3__Glycomycetales;D_4__Glycomycetaceae
D_0__Bacteria;D_1__Actinobacteria;D_2__Actinobacteria;D_3__Kineosporiales;D_4__Kineosporiaceae
D_0__Bacteria;D_1__Actinobacteria;D_2__Actinobacteria;D_3__Micrococcales;D_4__Beutenbergiaceae
D_0__Bacteria;D_1__Actinobacteria;D_2__Actinobacteria;D_3__Micrococcales;D_4__Bogoriellaceae
D_0__Bacteria;D_1__Actinobacteria;D_2__Actinobacteria;D_3__Micrococcales;D_4__Cellulomonadaceae
D_0__Bacteria;D_1__Actinobacteria;D_2__Actinobacteria;D_3__Micrococcales;D_4__Demequinaceae
D_0__Bacteria;D_1__Actinobacteria;D_2__Actinobacteria;D_3__Micrococcales;D_4__Dermabacteraceae
D_0__Bacteria;D_1__Actinobacteria;D_2__Actinobacteria;D_3__Micrococcales;D_4__Dermacoccaceae
D_0__Bacteria;D_1__Actinobacteria;D_2__Actinobacteria;D_3__Micrococcales;D_4__Dermatophilaceae
D_0__Bacteria;D_1__Actinobacteria;D_2__Actinobacteria;D_3__Micrococcales;D_4__Intrasporangiaceae
D_0__Bacteria;D_1__Actinobacteria;D_2__Actinobacteria;D_3__Micrococcales;D_4__Microbacteriaceae
D_0__Bacteria;D_1__Actinobacteria;D_2__Actinobacteria;D_3__Micrococcales;D_4__Micrococcaceae
D_0__Bacteria;D_1__Actinobacteria;D_2__Actinobacteria;D_3__Micrococcales;D_4__Promicromonosporaceae
D_0__Bacteria;D_1__Actinobacteria;D_2__Actinobacteria;D_3__Micrococcales;Other
D_0__Bacteria;D_1__Actinobacteria;D_2__Actinobacteria;D_3__Micromonosporales;D_4__Micromonosporaceae
D_0__Bacteria;D_1__Actinobacteria;D_2__Actinobacteria;D_3__PeM15;D_4__uncultured bacterium
D_0__Bacteria;D_1__Actinobacteria;D_2__Actinobacteria;D_3__Propionibacteriales;D_4__Nocardioidaceae
D_0__Bacteria;D_1__Actinobacteria;D_2__Actinobacteria;D_3__Propionibacteriales;D_4__Propionibacteriaceae
D_0__Bacteria;D_1__Actinobacteria;D_2__Actinobacteria;D_3__Propionibacteriales;Other
D_0__Bacteria;D_1__Actinobacteria;D_2__Actinobacteria;D_3__Pseudonocardiales;D_4__Pseudonocardiaceae
D_0__Bacteria;D_1__Actinobacteria;D_2__Actinobacteria;D_3__Streptomycetales;D_4__Streptomycetaceae
D_0__Bacteria;D_1__Actinobacteria;D_2__Actinobacteria;D_3__Streptosporangiales;D_4__Nocardiopsaceae
D_0__Bacteria;D_1__Actinobacteria;D_2__Actinobacteria;D_3__Streptosporangiales;D_4__Streptosporangiaceae
D_0__Bacteria;D_1__Actinobacteria;D_2__Actinobacteria;D_3__Streptosporangiales;D_4__Thermomonosporaceae
D_0__Bacteria;D_1__Actinobacteria;D_2__Actinobacteria;D_3__Streptosporangiales;D_4__uncultured bacterium
D_0__Bacteria;D_1__Actinobacteria;D_2__Actinobacteria;D_3__Streptosporangiales;Other
D_0__Bacteria;D_1__Actinobacteria;D_2__Actinobacteria;Other;Other
D_0__Bacteria;D_1__Actinobacteria;D_2__Coriobacteriia;D_3__Coriobacteriales;D_4__Coriobacteriaceae
D_0__Bacteria;D_1__Actinobacteria;D_2__MB-A2-108;Ambiguous_taxa;Ambiguous_taxa
D_0__Bacteria;D_1__Actinobacteria;D_2__MB-A2-108;D_3__uncultured bacterium;D_4__uncultured bacterium
D_0__Bacteria;D_1__Actinobacteria;D_2__MB-A2-108;Other;Other
D_0__Bacteria;D_1__Actinobacteria;D_2__Nitriliruptoria;D_3__Euzebyales;D_4__Euzebyaceae
D_0__Bacteria;D_1__Actinobacteria;D_2__Nitriliruptoria;D_3__Nitriliruptorales;D_4__Nitriliruptoraceae
D_0__Bacteria;D_1__Actinobacteria;D_2__Nitriliruptoria;Other;Other
D_0__Bacteria;D_1__Actinobacteria;D_2__OPB41;D_3__uncultured bacterium;D_4__uncultured bacterium
D_0__Bacteria;D_1__Actinobacteria;D_2__Rubrobacteria;D_3__Rubrobacterales;D_4__Rubrobacteriaceae
D_0__Bacteria;D_1__Actinobacteria;D_2__TakashiAC-B11;D_3__uncultured Actinomycetales bacterium;D_4__uncultured Actinomycetales bacterium
D_0__Bacteria;D_1__Actinobacteria;D_2__TakashiAC-B11;D_3__uncultured actinobacterium;D_4__uncultured actinobacterium
D_0__Bacteria;D_1__Actinobacteria;D_2__TakashiAC-B11;D_3__uncultured bacterium;D_4__uncultured bacterium
D_0__Bacteria;D_1__Actinobacteria;D_2__TakashiAC-B11;Other;Other
D_0__Bacteria;D_1__Actinobacteria;D_2__Thermoleophilia;D_3__Gaiellales;D_4__Gaiellaceae
D_0__Bacteria;D_1__Actinobacteria;D_2__Thermoleophilia;D_3__Gaiellales;D_4__uncultured
D_0__Bacteria;D_1__Actinobacteria;D_2__Thermoleophilia;D_3__Solirubrobacterales;Ambiguous_taxa
D_0__Bacteria;D_1__Actinobacteria;D_2__Thermoleophilia;D_3__Solirubrobacterales;D_4__0319-6M6
D_0__Bacteria;D_1__Actinobacteria;D_2__Thermoleophilia;D_3__Solirubrobacterales;D_4__288-2
D_0__Bacteria;D_1__Actinobacteria;D_2__Thermoleophilia;D_3__Solirubrobacterales;D_4__Elev-16S-1332
D_0__Bacteria;D_1__Actinobacteria;D_2__Thermoleophilia;D_3__Solirubrobacterales;D_4__FFCH11085
D_0__Bacteria;D_1__Actinobacteria;D_2__Thermoleophilia;D_3__Solirubrobacterales;D_4__Gsoil-1167
D_0__Bacteria;D_1__Actinobacteria;D_2__Thermoleophilia;D_3__Solirubrobacterales;D_4__Parviterribacteraceae
D_0__Bacteria;D_1__Actinobacteria;D_2__Thermoleophilia;D_3__Solirubrobacterales;D_4__Patulibacteraceae
D_0__Bacteria;D_1__Actinobacteria;D_2__Thermoleophilia;D_3__Solirubrobacterales;D_4__Q3-6C1
D_0__Bacteria;D_1__Actinobacteria;D_2__Thermoleophilia;D_3__Solirubrobacterales;D_4__S1-80
D_0__Bacteria;D_1__Actinobacteria;D_2__Thermoleophilia;D_3__Solirubrobacterales;D_4__Solirubrobacteraceae
D_0__Bacteria;D_1__Actinobacteria;D_2__Thermoleophilia;D_3__Solirubrobacterales;D_4__TM146
D_0__Bacteria;D_1__Actinobacteria;D_2__Thermoleophilia;D_3__Solirubrobacterales;D_4__YNPFFP1
D_0__Bacteria;D_1__Actinobacteria;D_2__Thermoleophilia;D_3__Solirubrobacterales;D_4__uncultured
D_0__Bacteria;D_1__Actinobacteria;D_2__Thermoleophilia;D_3__Solirubrobacterales;Other
D_0__Bacteria;D_1__Actinobacteria;D_2__Thermoleophilia;Other;Other
D_0__Bacteria;D_1__Actinobacteria;Other;Other;Other
D_0__Bacteria;D_1__Armatimonadetes;D_2__Armatimonadia;D_3__Armatimonadales;Ambiguous_taxa
D_0__Bacteria;D_1__Armatimonadetes;D_2__Armatimonadia;D_3__Armatimonadales;D_4__uncultured Armatimonadetes bacterium
D_0__Bacteria;D_1__Armatimonadetes;D_2__Armatimonadia;D_3__Armatimonadales;D_4__uncultured bacterium
D_0__Bacteria;D_1__Armatimonadetes;D_2__Fimbriimonadia;D_3__Fimbriimonadales;D_4__Fimbriimonadaceae
D_0__Bacteria;D_1__Armatimonadetes;D_2__uncultured;D_3__uncultured bacterium;D_4__uncultured bacterium
D_0__Bacteria;D_1__BRC1;D_2__uncultured bacterium;D_3__uncultured bacterium;D_4__uncultured bacterium
D_0__Bacteria;D_1__BRC1;Other;Other;Other
D_0__Bacteria;D_1__Bacteroidetes;D_2__Bacteroidetes BD2-2;D_3__uncultured bacterium;D_4__uncultured bacterium
D_0__Bacteria;D_1__Bacteroidetes;D_2__Bacteroidetes Incertae Sedis;D_3__Order II;D_4__Rhodothermaceae
D_0__Bacteria;D_1__Bacteroidetes;D_2__Bacteroidia;D_3__Bacteroidales;D_4__Bacteroidaceae
D_0__Bacteria;D_1__Bacteroidetes;D_2__Bacteroidia;D_3__Bacteroidales;D_4__Porphyromonadaceae
D_0__Bacteria;D_1__Bacteroidetes;D_2__Bacteroidia;D_3__Bacteroidales;D_4__Prevotellaceae
D_0__Bacteria;D_1__Bacteroidetes;D_2__Cytophagia;D_3__Cytophagales;D_4__Cyclobacteriaceae
D_0__Bacteria;D_1__Bacteroidetes;D_2__Cytophagia;D_3__Cytophagales;D_4__Cytophagaceae
D_0__Bacteria;D_1__Bacteroidetes;D_2__Cytophagia;D_3__Cytophagales;D_4__Flammeovirgaceae
D_0__Bacteria;D_1__Bacteroidetes;D_2__Cytophagia;D_3__Cytophagales;D_4__MWH-CFBk5
D_0__Bacteria;D_1__Bacteroidetes;D_2__Flavobacteriia;D_3__Flavobacteriales;D_4__Flavobacteriaceae
D_0__Bacteria;D_1__Bacteroidetes;D_2__Flavobacteriia;D_3__Flavobacteriales;D_4__NS9 marine group
D_0__Bacteria;D_1__Bacteroidetes;D_2__Sphingobacteriia;D_3__Sphingobacteriales;D_4__Chitinophagaceae
D_0__Bacteria;D_1__Bacteroidetes;D_2__Sphingobacteriia;D_3__Sphingobacteriales;D_4__KD3-93
D_0__Bacteria;D_1__Bacteroidetes;D_2__Sphingobacteriia;D_3__Sphingobacteriales;D_4__Lentimicrobiaceae
D_0__Bacteria;D_1__Bacteroidetes;D_2__Sphingobacteriia;D_3__Sphingobacteriales;D_4__LiUU-11-161
D_0__Bacteria;D_1__Bacteroidetes;D_2__Sphingobacteriia;D_3__Sphingobacteriales;D_4__NS11-12 marine group
D_0__Bacteria;D_1__Bacteroidetes;D_2__Sphingobacteriia;D_3__Sphingobacteriales;D_4__Saprospiraceae
D_0__Bacteria;D_1__Bacteroidetes;D_2__Sphingobacteriia;D_3__Sphingobacteriales;D_4__Sphingobacteriaceae
D_0__Bacteria;D_1__Bacteroidetes;D_2__Sphingobacteriia;D_3__Sphingobacteriales;D_4__env.OPS 17
D_0__Bacteria;D_1__Bacteroidetes;D_2__Sphingobacteriia;D_3__Sphingobacteriales;Other
D_0__Bacteria;D_1__Chlamydiae;D_2__Chlamydiae;D_3__Chlamydiales;D_4__Parachlamydiaceae
D_0__Bacteria;D_1__Chlamydiae;D_2__Chlamydiae;D_3__Chlamydiales;D_4__Simkaniaceae
D_0__Bacteria;D_1__Chlamydiae;D_2__Chlamydiae;D_3__Chlamydiales;D_4__cvE6
D_0__Bacteria;D_1__Chlorobi;D_2__Chlorobia;D_3__Chlorobiales;D_4__OPB56
D_0__Bacteria;D_1__Chlorobi;D_2__Chlorobia;D_3__Chlorobiales;D_4__SJA-28
D_0__Bacteria;D_1__Chloroflexi;D_2__Anaerolineae;D_3__Anaerolineales;D_4__Anaerolineaceae
D_0__Bacteria;D_1__Chloroflexi;D_2__Ardenticatenia;D_3__Ardenticatenales;D_4__uncultured bacterium
D_0__Bacteria;D_1__Chloroflexi;D_2__Caldilineae;D_3__Caldilineales;D_4__Caldilineaceae
D_0__Bacteria;D_1__Chloroflexi;D_2__Chloroflexi Incertae Sedis;D_3__Unknown Order;D_4__Unknown Family
D_0__Bacteria;D_1__Chloroflexi;D_2__Chloroflexia;D_3__Chloroflexales;D_4__Roseiflexaceae
D_0__Bacteria;D_1__Chloroflexi;D_2__Chloroflexia;D_3__Herpetosiphonales;D_4__Herpetosiphonaceae
D_0__Bacteria;D_1__Chloroflexi;D_2__Chloroflexia;D_3__Kallotenuales;D_4__AKIW781
D_0__Bacteria;D_1__Chloroflexi;D_2__Gitt-GS-136;Ambiguous_taxa;Ambiguous_taxa
D_0__Bacteria;D_1__Chloroflexi;D_2__Gitt-GS-136;D_3__uncultured bacterium;D_4__uncultured bacterium
D_0__Bacteria;D_1__Chloroflexi;D_2__Gitt-GS-136;D_3__uncultured bacterium #0319-6C24;D_4__uncultured bacterium #0319-6C24
D_0__Bacteria;D_1__Chloroflexi;D_2__Gitt-GS-136;Other;Other
D_0__Bacteria;D_1__Chloroflexi;D_2__JG30-KF-CM66;Ambiguous_taxa;Ambiguous_taxa
D_0__Bacteria;D_1__Chloroflexi;D_2__JG30-KF-CM66;D_3__uncultured Chloroflexi bacterium;D_4__uncultured Chloroflexi bacterium
D_0__Bacteria;D_1__Chloroflexi;D_2__JG30-KF-CM66;D_3__uncultured bacterium;D_4__uncultured bacterium
D_0__Bacteria;D_1__Chloroflexi;D_2__JG30-KF-CM66;Other;Other
D_0__Bacteria;D_1__Chloroflexi;D_2__JG37-AG-4;D_3__uncultured bacterium;D_4__uncultured bacterium
D_0__Bacteria;D_1__Chloroflexi;D_2__KD4-96;Ambiguous_taxa;Ambiguous_taxa
D_0__Bacteria;D_1__Chloroflexi;D_2__KD4-96;D_3__uncultured bacterium;D_4__uncultured bacterium
D_0__Bacteria;D_1__Chloroflexi;D_2__KD4-96;Other;Other
D_0__Bacteria;D_1__Chloroflexi;D_2__Ktedonobacteria;D_3__C0119;D_4__uncultured bacterium
D_0__Bacteria;D_1__Chloroflexi;D_2__NLS2-31;D_3__uncultured bacterium;D_4__uncultured bacterium
D_0__Bacteria;D_1__Chloroflexi;D_2__P2-11E;D_3__uncultured bacterium;D_4__uncultured bacterium
D_0__Bacteria;D_1__Chloroflexi;D_2__S085;Ambiguous_taxa;Ambiguous_taxa
D_0__Bacteria;D_1__Chloroflexi;D_2__S085;D_3__uncultured Chloroflexi bacterium;D_4__uncultured Chloroflexi bacterium
D_0__Bacteria;D_1__Chloroflexi;D_2__S085;D_3__uncultured bacterium;D_4__uncultured bacterium
D_0__Bacteria;D_1__Chloroflexi;D_2__S085;Other;Other
D_0__Bacteria;D_1__Chloroflexi;D_2__SAR202 clade;Ambiguous_taxa;Ambiguous_taxa
D_0__Bacteria;D_1__Chloroflexi;D_2__SAR202 clade;D_3__uncultured Chloroflexi bacterium;D_4__uncultured Chloroflexi bacterium
D_0__Bacteria;D_1__Chloroflexi;D_2__SAR202 clade;D_3__uncultured bacterium;D_4__uncultured bacterium
D_0__Bacteria;D_1__Chloroflexi;D_2__SAR202 clade;Other;Other
D_0__Bacteria;D_1__Chloroflexi;D_2__TK10;D_3__uncultured Chloroflexi bacterium;D_4__uncultured Chloroflexi bacterium
D_0__Bacteria;D_1__Chloroflexi;D_2__TK10;D_3__uncultured bacterium;D_4__uncultured bacterium
D_0__Bacteria;D_1__Chloroflexi;D_2__TK10;D_3__uncultured soil bacterium;D_4__uncultured soil bacterium
D_0__Bacteria;D_1__Chloroflexi;D_2__TK10;Other;Other
D_0__Bacteria;D_1__Chloroflexi;D_2__Thermomicrobia;D_3__AKYG1722;D_4__uncultured Chloroflexi bacterium
D_0__Bacteria;D_1__Chloroflexi;D_2__Thermomicrobia;D_3__AKYG1722;D_4__uncultured Sphaerobacter sp.
D_0__Bacteria;D_1__Chloroflexi;D_2__Thermomicrobia;D_3__AKYG1722;D_4__uncultured bacterium
D_0__Bacteria;D_1__Chloroflexi;D_2__Thermomicrobia;D_3__AKYG1722;Other
D_0__Bacteria;D_1__Chloroflexi;D_2__Thermomicrobia;D_3__JG30-KF-CM45;Ambiguous_taxa
D_0__Bacteria;D_1__Chloroflexi;D_2__Thermomicrobia;D_3__JG30-KF-CM45;D_4__uncultured Chloroflexi bacterium
D_0__Bacteria;D_1__Chloroflexi;D_2__Thermomicrobia;D_3__JG30-KF-CM45;D_4__uncultured bacterium
D_0__Bacteria;D_1__Chloroflexi;D_2__Thermomicrobia;D_3__Sphaerobacterales;D_4__Sphaerobacteraceae
D_0__Bacteria;D_1__Chloroflexi;Other;Other;Other
D_0__Bacteria;D_1__Cyanobacteria;D_2__Chloroplast;Ambiguous_taxa;Ambiguous_taxa
D_0__Bacteria;D_1__Cyanobacteria;D_2__Chloroplast;D_3__uncultured bacterium;D_4__uncultured bacterium
D_0__Bacteria;D_1__Cyanobacteria;D_2__Cyanobacteria;D_3__SubsectionIII;D_4__FamilyI
D_0__Bacteria;D_1__Cyanobacteria;D_2__Cyanobacteria;D_3__uncultured;D_4__uncultured bacterium
D_0__Bacteria;D_1__Cyanobacteria;D_2__Melainabacteria;D_3__Gastranaerophilales;Other
D_0__Bacteria;D_1__Cyanobacteria;D_2__Melainabacteria;D_3__Vampirovibrionales;D_4__uncultured bacterium
D_0__Bacteria;D_1__Deinococcus-Thermus;D_2__Deinococci;D_3__Deinococcales;D_4__Deinococcaceae
D_0__Bacteria;D_1__Deinococcus-Thermus;D_2__Deinococci;D_3__Deinococcales;D_4__Trueperaceae
D_0__Bacteria;D_1__Deinococcus-Thermus;D_2__Deinococci;D_3__Thermales;D_4__Thermaceae
D_0__Bacteria;D_1__Elusimicrobia;D_2__Elusimicrobia;D_3__Lineage IIa;D_4__uncultured bacterium
D_0__Bacteria;D_1__Elusimicrobia;D_2__Elusimicrobia;D_3__Lineage IIb;D_4__uncultured bacterium
D_0__Bacteria;D_1__Elusimicrobia;D_2__Elusimicrobia;D_3__Lineage IV;D_4__uncultured bacterium
D_0__Bacteria;D_1__FBP;D_2__uncultured bacterium;D_3__uncultured bacterium;D_4__uncultured bacterium
D_0__Bacteria;D_1__Fibrobacteres;D_2__Fibrobacteria;D_3__Fibrobacterales;D_4__Fibrobacteraceae
D_0__Bacteria;D_1__Firmicutes;D_2__Bacilli;D_3__Bacillales;D_4__Alicyclobacillaceae
D_0__Bacteria;D_1__Firmicutes;D_2__Bacilli;D_3__Bacillales;D_4__Bacillaceae
D_0__Bacteria;D_1__Firmicutes;D_2__Bacilli;D_3__Bacillales;D_4__Family X
D_0__Bacteria;D_1__Firmicutes;D_2__Bacilli;D_3__Bacillales;D_4__Family XI
D_0__Bacteria;D_1__Firmicutes;D_2__Bacilli;D_3__Bacillales;D_4__Paenibacillaceae
D_0__Bacteria;D_1__Firmicutes;D_2__Bacilli;D_3__Bacillales;D_4__Planococcaceae
D_0__Bacteria;D_1__Firmicutes;D_2__Bacilli;D_3__Bacillales;D_4__Staphylococcaceae
D_0__Bacteria;D_1__Firmicutes;D_2__Bacilli;D_3__Bacillales;D_4__Thermoactinomycetaceae
D_0__Bacteria;D_1__Firmicutes;D_2__Bacilli;D_3__Bacillales;Other
D_0__Bacteria;D_1__Firmicutes;D_2__Bacilli;D_3__Lactobacillales;D_4__Aerococcaceae
D_0__Bacteria;D_1__Firmicutes;D_2__Bacilli;D_3__Lactobacillales;D_4__Carnobacteriaceae
D_0__Bacteria;D_1__Firmicutes;D_2__Bacilli;D_3__Lactobacillales;D_4__Enterococcaceae
D_0__Bacteria;D_1__Firmicutes;D_2__Bacilli;D_3__Lactobacillales;D_4__Streptococcaceae
D_0__Bacteria;D_1__Firmicutes;D_2__Bacilli;D_3__Lactobacillales;Other
D_0__Bacteria;D_1__Firmicutes;D_2__Clostridia;D_3__Clostridiales;D_4__Clostridiaceae 1
D_0__Bacteria;D_1__Firmicutes;D_2__Clostridia;D_3__Clostridiales;D_4__Family XI
D_0__Bacteria;D_1__Firmicutes;D_2__Clostridia;D_3__Clostridiales;D_4__Family XIII
D_0__Bacteria;D_1__Firmicutes;D_2__Clostridia;D_3__Clostridiales;D_4__Heliobacteriaceae
D_0__Bacteria;D_1__Firmicutes;D_2__Clostridia;D_3__Clostridiales;D_4__Lachnospiraceae
D_0__Bacteria;D_1__Firmicutes;D_2__Clostridia;D_3__Clostridiales;D_4__Peptococcaceae
D_0__Bacteria;D_1__Firmicutes;D_2__Clostridia;D_3__Clostridiales;D_4__Peptostreptococcaceae
D_0__Bacteria;D_1__Firmicutes;D_2__Clostridia;D_3__Clostridiales;D_4__Ruminococcaceae
D_0__Bacteria;D_1__Firmicutes;D_2__Clostridia;D_3__Halanaerobiales;D_4__ODP1230B8.23
D_0__Bacteria;D_1__Firmicutes;D_2__Clostridia;D_3__Thermoanaerobacterales;D_4__Thermoanaerobacteraceae
D_0__Bacteria;D_1__Firmicutes;D_2__Erysipelotrichia;D_3__Erysipelotrichales;D_4__Erysipelotrichaceae
D_0__Bacteria;D_1__Firmicutes;D_2__Negativicutes;D_3__Selenomonadales;D_4__Veillonellaceae
D_0__Bacteria;D_1__Fusobacteria;D_2__Fusobacteriia;D_3__Fusobacteriales;D_4__Fusobacteriaceae
D_0__Bacteria;D_1__Fusobacteria;D_2__Fusobacteriia;D_3__Fusobacteriales;D_4__Leptotrichiaceae
D_0__Bacteria;D_1__Gemmatimonadetes;D_2__BD2-11 terrestrial group;D_3__uncultured bacterium;D_4__uncultured bacterium
D_0__Bacteria;D_1__Gemmatimonadetes;D_2__Gemmatimonadetes;D_3__Gemmatimonadales;D_4__Gemmatimonadaceae
D_0__Bacteria;D_1__Gemmatimonadetes;D_2__Longimicrobia;D_3__Longimicrobiales;D_4__Longimicrobiaceae
D_0__Bacteria;D_1__Gemmatimonadetes;D_2__S0134 terrestrial group;D_3__uncultured Gemmatimonadetes bacterium;D_4__uncultured Gemmatimonadetes bacterium
D_0__Bacteria;D_1__Gemmatimonadetes;D_2__S0134 terrestrial group;D_3__uncultured bacterium;D_4__uncultured bacterium
D_0__Bacteria;D_1__Hydrogenedentes;D_2__uncultured bacterium;D_3__uncultured bacterium;D_4__uncultured bacterium
D_0__Bacteria;D_1__Nitrospirae;D_2__Nitrospira;D_3__Nitrospirales;D_4__0319-6A21
D_0__Bacteria;D_1__Nitrospirae;D_2__Nitrospira;D_3__Nitrospirales;D_4__Nitrospiraceae
D_0__Bacteria;D_1__Planctomycetes;D_2__Phycisphaerae;D_3__Phycisphaerales;D_4__Phycisphaeraceae
D_0__Bacteria;D_1__Planctomycetes;D_2__Phycisphaerae;D_3__Tepidisphaerales;D_4__Tepidisphaeraceae
D_0__Bacteria;D_1__Planctomycetes;D_2__Planctomycetacia;D_3__Planctomycetales;D_4__Planctomycetaceae
D_0__Bacteria;D_1__Proteobacteria;D_2__Alphaproteobacteria;D_3__Alphaproteobacteria Incertae Sedis;D_4__Unknown Family
D_0__Bacteria;D_1__Proteobacteria;D_2__Alphaproteobacteria;D_3__Caulobacterales;D_4__Caulobacteraceae
D_0__Bacteria;D_1__Proteobacteria;D_2__Alphaproteobacteria;D_3__Rhizobiales;D_4__1174-901-12
D_0__Bacteria;D_1__Proteobacteria;D_2__Alphaproteobacteria;D_3__Rhizobiales;D_4__A0839
D_0__Bacteria;D_1__Proteobacteria;D_2__Alphaproteobacteria;D_3__Rhizobiales;D_4__Aurantimonadaceae
D_0__Bacteria;D_1__Proteobacteria;D_2__Alphaproteobacteria;D_3__Rhizobiales;D_4__Beijerinckiaceae
D_0__Bacteria;D_1__Proteobacteria;D_2__Alphaproteobacteria;D_3__Rhizobiales;D_4__Bradyrhizobiaceae
D_0__Bacteria;D_1__Proteobacteria;D_2__Alphaproteobacteria;D_3__Rhizobiales;D_4__Brucellaceae
D_0__Bacteria;D_1__Proteobacteria;D_2__Alphaproteobacteria;D_3__Rhizobiales;D_4__C2U
D_0__Bacteria;D_1__Proteobacteria;D_2__Alphaproteobacteria;D_3__Rhizobiales;D_4__DUNssu044
D_0__Bacteria;D_1__Proteobacteria;D_2__Alphaproteobacteria;D_3__Rhizobiales;D_4__FukuN57
D_0__Bacteria;D_1__Proteobacteria;D_2__Alphaproteobacteria;D_3__Rhizobiales;D_4__Hyphomicrobiaceae
D_0__Bacteria;D_1__Proteobacteria;D_2__Alphaproteobacteria;D_3__Rhizobiales;D_4__JG34-KF-361
D_0__Bacteria;D_1__Proteobacteria;D_2__Alphaproteobacteria;D_3__Rhizobiales;D_4__KF-JG30-B3
D_0__Bacteria;D_1__Proteobacteria;D_2__Alphaproteobacteria;D_3__Rhizobiales;D_4__MNG7
D_0__Bacteria;D_1__Proteobacteria;D_2__Alphaproteobacteria;D_3__Rhizobiales;D_4__Methylobacteriaceae
D_0__Bacteria;D_1__Proteobacteria;D_2__Alphaproteobacteria;D_3__Rhizobiales;D_4__Methylocystaceae
D_0__Bacteria;D_1__Proteobacteria;D_2__Alphaproteobacteria;D_3__Rhizobiales;D_4__Phyllobacteriaceae
D_0__Bacteria;D_1__Proteobacteria;D_2__Alphaproteobacteria;D_3__Rhizobiales;D_4__Rhizobiaceae
D_0__Bacteria;D_1__Proteobacteria;D_2__Alphaproteobacteria;D_3__Rhizobiales;D_4__Rhizobiales Incertae Sedis
D_0__Bacteria;D_1__Proteobacteria;D_2__Alphaproteobacteria;D_3__Rhizobiales;D_4__Rhodobiaceae
D_0__Bacteria;D_1__Proteobacteria;D_2__Alphaproteobacteria;D_3__Rhizobiales;D_4__Roseiarcaceae
D_0__Bacteria;D_1__Proteobacteria;D_2__Alphaproteobacteria;D_3__Rhizobiales;D_4__Xanthobacteraceae
D_0__Bacteria;D_1__Proteobacteria;D_2__Alphaproteobacteria;D_3__Rhizobiales;D_4__alphaI cluster
D_0__Bacteria;D_1__Proteobacteria;D_2__Alphaproteobacteria;D_3__Rhizobiales;D_4__uncultured
D_0__Bacteria;D_1__Proteobacteria;D_2__Alphaproteobacteria;D_3__Rhizobiales;Other
D_0__Bacteria;D_1__Proteobacteria;D_2__Alphaproteobacteria;D_3__Rhodobacterales;D_4__Rhodobacteraceae
D_0__Bacteria;D_1__Proteobacteria;D_2__Alphaproteobacteria;D_3__Rhodospirillales;D_4__AT-s3-44
D_0__Bacteria;D_1__Proteobacteria;D_2__Alphaproteobacteria;D_3__Rhodospirillales;D_4__Acetobacteraceae
D_0__Bacteria;D_1__Proteobacteria;D_2__Alphaproteobacteria;D_3__Rhodospirillales;D_4__DA111
D_0__Bacteria;D_1__Proteobacteria;D_2__Alphaproteobacteria;D_3__Rhodospirillales;D_4__KCM-B-15
D_0__Bacteria;D_1__Proteobacteria;D_2__Alphaproteobacteria;D_3__Rhodospirillales;D_4__MND8
D_0__Bacteria;D_1__Proteobacteria;D_2__Alphaproteobacteria;D_3__Rhodospirillales;D_4__MSB-1E8
D_0__Bacteria;D_1__Proteobacteria;D_2__Alphaproteobacteria;D_3__Rhodospirillales;D_4__Rhodospirillaceae
D_0__Bacteria;D_1__Proteobacteria;D_2__Alphaproteobacteria;D_3__Rhodospirillales;D_4__Rhodospirillales Incertae Sedis
D_0__Bacteria;D_1__Proteobacteria;D_2__Alphaproteobacteria;D_3__Rhodospirillales;Other
D_0__Bacteria;D_1__Proteobacteria;D_2__Alphaproteobacteria;D_3__Rickettsiales;D_4__EF100-94H03
D_0__Bacteria;D_1__Proteobacteria;D_2__Alphaproteobacteria;D_3__Rickettsiales;D_4__Mitochondria
D_0__Bacteria;D_1__Proteobacteria;D_2__Alphaproteobacteria;D_3__Rickettsiales;D_4__Rickettsiaceae
D_0__Bacteria;D_1__Proteobacteria;D_2__Alphaproteobacteria;D_3__Rickettsiales;D_4__SM2D12
D_0__Bacteria;D_1__Proteobacteria;D_2__Alphaproteobacteria;D_3__Rickettsiales;D_4__TK34
D_0__Bacteria;D_1__Proteobacteria;D_2__Alphaproteobacteria;D_3__Sphingomonadales;D_4__7B-8
D_0__Bacteria;D_1__Proteobacteria;D_2__Alphaproteobacteria;D_3__Sphingomonadales;D_4__Ellin6055
D_0__Bacteria;D_1__Proteobacteria;D_2__Alphaproteobacteria;D_3__Sphingomonadales;D_4__Erythrobacteraceae
D_0__Bacteria;D_1__Proteobacteria;D_2__Alphaproteobacteria;D_3__Sphingomonadales;D_4__Sphingomonadaceae
D_0__Bacteria;D_1__Proteobacteria;D_2__Alphaproteobacteria;D_3__Sphingomonadales;Other
D_0__Bacteria;D_1__Proteobacteria;D_2__Alphaproteobacteria;Other;Other
D_0__Bacteria;D_1__Proteobacteria;D_2__Betaproteobacteria;D_3__Burkholderiales;D_4__Alcaligenaceae
D_0__Bacteria;D_1__Proteobacteria;D_2__Betaproteobacteria;D_3__Burkholderiales;D_4__Burkholderiaceae
D_0__Bacteria;D_1__Proteobacteria;D_2__Betaproteobacteria;D_3__Burkholderiales;D_4__Comamonadaceae
D_0__Bacteria;D_1__Proteobacteria;D_2__Betaproteobacteria;D_3__Burkholderiales;D_4__Oxalobacteraceae
D_0__Bacteria;D_1__Proteobacteria;D_2__Betaproteobacteria;D_3__Hydrogenophilales;D_4__Hydrogenophilaceae
D_0__Bacteria;D_1__Proteobacteria;D_2__Betaproteobacteria;D_3__Methylophilales;D_4__Methylophilaceae
D_0__Bacteria;D_1__Proteobacteria;D_2__Betaproteobacteria;D_3__Neisseriales;D_4__Neisseriaceae
D_0__Bacteria;D_1__Proteobacteria;D_2__Betaproteobacteria;D_3__Nitrosomonadales;D_4__Gallionellaceae
D_0__Bacteria;D_1__Proteobacteria;D_2__Betaproteobacteria;D_3__Nitrosomonadales;D_4__Nitrosomonadaceae
D_0__Bacteria;D_1__Proteobacteria;D_2__Betaproteobacteria;D_3__Rhodocyclales;D_4__Rhodocyclaceae
D_0__Bacteria;D_1__Proteobacteria;D_2__Betaproteobacteria;D_3__SC-I-84;D_4__uncultured bacterium
D_0__Bacteria;D_1__Proteobacteria;D_2__Betaproteobacteria;D_3__TRA3-20;Ambiguous_taxa
D_0__Bacteria;D_1__Proteobacteria;D_2__Betaproteobacteria;D_3__TRA3-20;D_4__uncultured bacterium
D_0__Bacteria;D_1__Proteobacteria;D_2__Betaproteobacteria;D_3__TRA3-20;D_4__uncultured gamma proteobacterium
D_0__Bacteria;D_1__Proteobacteria;D_2__Betaproteobacteria;D_3__TRA3-20;Other
D_0__Bacteria;D_1__Proteobacteria;D_2__Betaproteobacteria;Other;Other
D_0__Bacteria;D_1__Proteobacteria;D_2__Deltaproteobacteria;D_3__Bdellovibrionales;D_4__Bacteriovoracaceae
D_0__Bacteria;D_1__Proteobacteria;D_2__Deltaproteobacteria;D_3__Bdellovibrionales;D_4__Bdellovibrionaceae
D_0__Bacteria;D_1__Proteobacteria;D_2__Deltaproteobacteria;D_3__Desulfurellales;D_4__Desulfurellaceae
D_0__Bacteria;D_1__Proteobacteria;D_2__Deltaproteobacteria;D_3__Myxococcales;Ambiguous_taxa
D_0__Bacteria;D_1__Proteobacteria;D_2__Deltaproteobacteria;D_3__Myxococcales;D_4__Archangiaceae
D_0__Bacteria;D_1__Proteobacteria;D_2__Deltaproteobacteria;D_3__Myxococcales;D_4__BIrii41
D_0__Bacteria;D_1__Proteobacteria;D_2__Deltaproteobacteria;D_3__Myxococcales;D_4__Haliangiaceae
D_0__Bacteria;D_1__Proteobacteria;D_2__Deltaproteobacteria;D_3__Myxococcales;D_4__Nannocystaceae
D_0__Bacteria;D_1__Proteobacteria;D_2__Deltaproteobacteria;D_3__Myxococcales;D_4__P3OB-42
D_0__Bacteria;D_1__Proteobacteria;D_2__Deltaproteobacteria;D_3__Myxococcales;D_4__Phaselicystidaceae
D_0__Bacteria;D_1__Proteobacteria;D_2__Deltaproteobacteria;D_3__Myxococcales;D_4__Polyangiaceae
D_0__Bacteria;D_1__Proteobacteria;D_2__Deltaproteobacteria;D_3__Myxococcales;D_4__Sandaracinaceae
D_0__Bacteria;D_1__Proteobacteria;D_2__Deltaproteobacteria;D_3__Myxococcales;D_4__VHS-B3-70
D_0__Bacteria;D_1__Proteobacteria;D_2__Deltaproteobacteria;D_3__Myxococcales;D_4__mle1-27
D_0__Bacteria;D_1__Proteobacteria;D_2__Deltaproteobacteria;D_3__Myxococcales;D_4__uncultured
D_0__Bacteria;D_1__Proteobacteria;D_2__Deltaproteobacteria;D_3__Myxococcales;D_4__uncultured bacterium
D_0__Bacteria;D_1__Proteobacteria;D_2__Deltaproteobacteria;D_3__Myxococcales;Other
D_0__Bacteria;D_1__Proteobacteria;D_2__Deltaproteobacteria;D_3__Oligoflexales;D_4__0319-6G20
D_0__Bacteria;D_1__Proteobacteria;D_2__Deltaproteobacteria;D_3__Oligoflexales;D_4__Oligoflexaceae
D_0__Bacteria;D_1__Proteobacteria;D_2__Deltaproteobacteria;D_3__SAR324 clade(Marine group B);D_4__uncultured bacterium
D_0__Bacteria;D_1__Proteobacteria;D_2__Deltaproteobacteria;D_3__SAR324 clade(Marine group B);D_4__uncultured delta proteobacterium
D_0__Bacteria;D_1__Proteobacteria;D_2__Epsilonproteobacteria;D_3__Campylobacterales;D_4__Campylobacteraceae
D_0__Bacteria;D_1__Proteobacteria;D_2__Gammaproteobacteria;D_3__Alteromonadales;D_4__Alteromonadaceae
D_0__Bacteria;D_1__Proteobacteria;D_2__Gammaproteobacteria;D_3__Alteromonadales;D_4__Psychromonadaceae
D_0__Bacteria;D_1__Proteobacteria;D_2__Gammaproteobacteria;D_3__Cardiobacteriales;D_4__Cardiobacteriaceae
D_0__Bacteria;D_1__Proteobacteria;D_2__Gammaproteobacteria;D_3__Cellvibrionales;D_4__Cellvibrionaceae
D_0__Bacteria;D_1__Proteobacteria;D_2__Gammaproteobacteria;D_3__Chromatiales;D_4__Chromatiaceae
D_0__Bacteria;D_1__Proteobacteria;D_2__Gammaproteobacteria;D_3__Chromatiales;D_4__Ectothiorhodospiraceae
D_0__Bacteria;D_1__Proteobacteria;D_2__Gammaproteobacteria;D_3__Chromatiales;Other
D_0__Bacteria;D_1__Proteobacteria;D_2__Gammaproteobacteria;D_3__Enterobacteriales;D_4__Enterobacteriaceae
D_0__Bacteria;D_1__Proteobacteria;D_2__Gammaproteobacteria;D_3__Gammaproteobacteria Incertae Sedis;D_4__Unknown Family
D_0__Bacteria;D_1__Proteobacteria;D_2__Gammaproteobacteria;D_3__HTA4;D_4__uncultured bacterium
D_0__Bacteria;D_1__Proteobacteria;D_2__Gammaproteobacteria;D_3__KI89A clade;D_4__uncultured bacterium
D_0__Bacteria;D_1__Proteobacteria;D_2__Gammaproteobacteria;D_3__Legionellales;D_4__Coxiellaceae
D_0__Bacteria;D_1__Proteobacteria;D_2__Gammaproteobacteria;D_3__Legionellales;D_4__Legionellaceae
D_0__Bacteria;D_1__Proteobacteria;D_2__Gammaproteobacteria;D_3__Oceanospirillales;D_4__Halomonadaceae
D_0__Bacteria;D_1__Proteobacteria;D_2__Gammaproteobacteria;D_3__Oceanospirillales;D_4__Oceanospirillaceae
D_0__Bacteria;D_1__Proteobacteria;D_2__Gammaproteobacteria;D_3__PYR10d3;Other
D_0__Bacteria;D_1__Proteobacteria;D_2__Gammaproteobacteria;D_3__Pasteurellales;D_4__Pasteurellaceae
D_0__Bacteria;D_1__Proteobacteria;D_2__Gammaproteobacteria;D_3__Pseudomonadales;D_4__Moraxellaceae
D_0__Bacteria;D_1__Proteobacteria;D_2__Gammaproteobacteria;D_3__Pseudomonadales;D_4__Pseudomonadaceae
D_0__Bacteria;D_1__Proteobacteria;D_2__Gammaproteobacteria;D_3__Thiotrichales;D_4__Piscirickettsiaceae
D_0__Bacteria;D_1__Proteobacteria;D_2__Gammaproteobacteria;D_3__Thiotrichales;D_4__Thiotrichaceae
D_0__Bacteria;D_1__Proteobacteria;D_2__Gammaproteobacteria;D_3__Vibrionales;D_4__Vibrionaceae
D_0__Bacteria;D_1__Proteobacteria;D_2__Gammaproteobacteria;D_3__X35;Other
D_0__Bacteria;D_1__Proteobacteria;D_2__Gammaproteobacteria;D_3__Xanthomonadales;D_4__JTB255 marine benthic group
D_0__Bacteria;D_1__Proteobacteria;D_2__Gammaproteobacteria;D_3__Xanthomonadales;D_4__Nevskiaceae
D_0__Bacteria;D_1__Proteobacteria;D_2__Gammaproteobacteria;D_3__Xanthomonadales;D_4__Xanthomonadaceae
D_0__Bacteria;D_1__Proteobacteria;D_2__Gammaproteobacteria;D_3__Xanthomonadales;D_4__Xanthomonadales Incertae Sedis
D_0__Bacteria;D_1__Proteobacteria;D_2__Gammaproteobacteria;D_3__Xanthomonadales;D_4__uncultured
D_0__Bacteria;D_1__Proteobacteria;D_2__Gammaproteobacteria;D_3__Xanthomonadales;Other
D_0__Bacteria;D_1__Proteobacteria;D_2__Gammaproteobacteria;D_3__uncultured;D_4__uncultured bacterium
D_0__Bacteria;D_1__Proteobacteria;D_2__Gammaproteobacteria;D_3__uncultured;Other
D_0__Bacteria;D_1__Proteobacteria;D_2__Gammaproteobacteria;Other;Other
D_0__Bacteria;D_1__Proteobacteria;D_2__JTB23;D_3__uncultured bacterium;D_4__uncultured bacterium
D_0__Bacteria;D_1__Proteobacteria;D_2__JTB23;Other;Other
D_0__Bacteria;D_1__Proteobacteria;D_2__SPOTSOCT00m83;Ambiguous_taxa;Ambiguous_taxa
D_0__Bacteria;D_1__Proteobacteria;D_2__SPOTSOCT00m83;Other;Other
D_0__Bacteria;D_1__Proteobacteria;Other;Other;Other
D_0__Bacteria;D_1__SBR1093;Other;Other;Other
D_0__Bacteria;D_1__SR1 (Absconditabacteria);Ambiguous_taxa;Ambiguous_taxa;Ambiguous_taxa
D_0__Bacteria;D_1__SR1 (Absconditabacteria);D_2__uncultured bacterium;D_3__uncultured bacterium;D_4__uncultured bacterium
D_0__Bacteria;D_1__Saccharibacteria;D_2__uncultured bacterium;D_3__uncultured bacterium;D_4__uncultured bacterium
D_0__Bacteria;D_1__Saccharibacteria;Other;Other;Other
D_0__Bacteria;D_1__Spirochaetae;D_2__Spirochaetes;D_3__Spirochaetales;D_4__Spirochaetaceae
D_0__Bacteria;D_1__Synergistetes;D_2__Synergistia;D_3__Synergistales;D_4__Synergistaceae
D_0__Bacteria;D_1__TM6 (Dependentiae);Other;Other;Other
D_0__Bacteria;D_1__Tectomicrobia;D_2__Tectomicrobia Incertae Sedis;D_3__Unknown Order;D_4__Unknown Family
D_0__Bacteria;D_1__Tectomicrobia;D_2__uncultured bacterium;D_3__uncultured bacterium;D_4__uncultured bacterium
D_0__Bacteria;D_1__Verrucomicrobia;D_2__OPB35 soil group;Ambiguous_taxa;Ambiguous_taxa
D_0__Bacteria;D_1__Verrucomicrobia;D_2__OPB35 soil group;D_3__uncultured bacterium;D_4__uncultured bacterium
D_0__Bacteria;D_1__Verrucomicrobia;D_2__OPB35 soil group;Other;Other
D_0__Bacteria;D_1__Verrucomicrobia;D_2__Opitutae;D_3__BC-COM435;D_4__uncultured bacterium
D_0__Bacteria;D_1__Verrucomicrobia;D_2__Opitutae;D_3__Opitutales;D_4__Opitutaceae
D_0__Bacteria;D_1__Verrucomicrobia;D_2__Spartobacteria;D_3__Chthoniobacterales;D_4__Chthoniobacteraceae
D_0__Bacteria;D_1__Verrucomicrobia;D_2__Spartobacteria;D_3__Chthoniobacterales;D_4__DA101 soil group
D_0__Bacteria;D_1__Verrucomicrobia;D_2__Spartobacteria;D_3__Chthoniobacterales;D_4__Xiphinematobacteraceae
D_0__Bacteria;D_1__Verrucomicrobia;D_2__Spartobacteria;D_3__Chthoniobacterales;Other
D_0__Bacteria;D_1__Verrucomicrobia;D_2__Verrucomicrobiae;D_3__Verrucomicrobiales;D_4__DEV007
D_0__Bacteria;D_1__Verrucomicrobia;D_2__Verrucomicrobiae;D_3__Verrucomicrobiales;D_4__Verrucomicrobiaceae
D_0__Bacteria;Other;Other;Other;Other
Unassigned;Other;Other;Other;Other
