# Supplementary material for: Cretaceous dinosaur bone contains recent organic material and provides an environment conducive to microbial communities
Source: eLife. 2019 Jun 18;8:e46205. doi: 10.7554/eLife.46205 (PMC6581507; doi:10.7554/eLife.46205)
Supplement: Source data 1. [file 1jzZ6e9rzMY3toQzE7srFX0qCUAZJt_legend.pdf › Raw data files/16S rRNA amplicon sequencing/taxa_plots/taxa_summary_plots/charts/1jzZ6e9rzMY3toQzE7srFX0qCUAZJt_legend.pdf]

D_0__Archaea;D_1__Thaumarchaeota;D_2__Soil Crenarchaeotic Group(SCG);D_3__Unknown Order;D_4__Unknown Family;D_5__Candidatus Nitrososphaera
D_0__Archaea;D_1__Thaumarchaeota;D_2__Soil Crenarchaeotic Group(SCG);D_3__uncultured archaeon;D_4__uncultured archaeon;D_5__uncultured archaeon
D_0__Archaea;D_1__Thaumarchaeota;D_2__Soil Crenarchaeotic Group(SCG);D_3__uncultured euryarchaeote;D_4__uncultured euryarchaeote;D_5__uncultured euryarchaeote
D_0__Archaea;D_1__Thaumarchaeota;D_2__Soil Crenarchaeotic Group(SCG);Other;Other;Other
D_0__Bacteria;D_1__Acidobacteria;D_2__Blastocatellia;D_3__Blastocatellales;D_4__Blastocatellaceae (Subgroup 4);D_5__11-24
D_0__Bacteria;D_1__Acidobacteria;D_2__Blastocatellia;D_3__Blastocatellales;D_4__Blastocatellaceae (Subgroup 4);D_5__Aridibacter
D_0__Bacteria;D_1__Acidobacteria;D_2__Blastocatellia;D_3__Blastocatellales;D_4__Blastocatellaceae (Subgroup 4);D_5__Blastocatella
D_0__Bacteria;D_1__Acidobacteria;D_2__Blastocatellia;D_3__Blastocatellales;D_4__Blastocatellaceae (Subgroup 4);D_5__DS-100
D_0__Bacteria;D_1__Acidobacteria;D_2__Blastocatellia;D_3__Blastocatellales;D_4__Blastocatellaceae (Subgroup 4);D_5__RB41
D_0__Bacteria;D_1__Acidobacteria;D_2__Blastocatellia;D_3__Blastocatellales;D_4__Blastocatellaceae (Subgroup 4);D_5__Stenotrophobacter
D_0__Bacteria;D_1__Acidobacteria;D_2__Blastocatellia;D_3__Blastocatellales;D_4__Blastocatellaceae (Subgroup 4);D_5__uncultured
D_0__Bacteria;D_1__Acidobacteria;D_2__Blastocatellia;D_3__Blastocatellales;D_4__Blastocatellaceae (Subgroup 4);Other
D_0__Bacteria;D_1__Acidobacteria;D_2__Holophagae;D_3__Holophagales;D_4__Holophagaceae;D_5__uncultured
D_0__Bacteria;D_1__Acidobacteria;D_2__Holophagae;D_3__Subgroup 10;D_4__CA002;D_5__uncultured Acidobacteria bacterium
D_0__Bacteria;D_1__Acidobacteria;D_2__Holophagae;D_3__Subgroup 10;D_4__CA002;D_5__uncultured bacterium
D_0__Bacteria;D_1__Acidobacteria;D_2__Holophagae;D_3__Subgroup 10;D_4__CA002;Other
D_0__Bacteria;D_1__Acidobacteria;D_2__Holophagae;D_3__Subgroup 10;D_4__NS72;D_5__uncultured bacterium
D_0__Bacteria;D_1__Acidobacteria;D_2__Holophagae;D_3__Subgroup 10;D_4__NS72;Other
D_0__Bacteria;D_1__Acidobacteria;D_2__Holophagae;D_3__Subgroup 10;D_4__Sva0725;D_5__uncultured bacterium
D_0__Bacteria;D_1__Acidobacteria;D_2__Holophagae;D_3__Subgroup 10;Other;Other
D_0__Bacteria;D_1__Acidobacteria;D_2__Holophagae;D_3__Subgroup 7;Ambiguous_taxa;Ambiguous_taxa
D_0__Bacteria;D_1__Acidobacteria;D_2__Holophagae;D_3__Subgroup 7;D_4__uncultured bacterium;D_5__uncultured bacterium
D_0__Bacteria;D_1__Acidobacteria;D_2__Holophagae;D_3__Subgroup 7;Other;Other
D_0__Bacteria;D_1__Acidobacteria;D_2__Solibacteres;D_3__Solibacterales;D_4__Solibacteraceae (Subgroup 3);D_5__Bryobacter
D_0__Bacteria;D_1__Acidobacteria;D_2__Solibacteres;D_3__Solibacterales;D_4__Solibacteraceae (Subgroup 3);D_5__Candidatus Solibacter
D_0__Bacteria;D_1__Acidobacteria;D_2__Solibacteres;D_3__Solibacterales;D_4__Solibacteraceae (Subgroup 3);D_5__PAUC26f
D_0__Bacteria;D_1__Acidobacteria;D_2__Subgroup 17;Ambiguous_taxa;Ambiguous_taxa;Ambiguous_taxa
D_0__Bacteria;D_1__Acidobacteria;D_2__Subgroup 17;D_3__uncultured Acidobacteria bacterium;D_4__uncultured Acidobacteria bacterium;D_5__uncultured Acidobacteria bacterium
D_0__Bacteria;D_1__Acidobacteria;D_2__Subgroup 17;Other;Other;Other
D_0__Bacteria;D_1__Acidobacteria;D_2__Subgroup 22;Ambiguous_taxa;Ambiguous_taxa;Ambiguous_taxa
D_0__Bacteria;D_1__Acidobacteria;D_2__Subgroup 22;D_3__uncultured Acidobacteria bacterium;D_4__uncultured Acidobacteria bacterium;D_5__uncultured Acidobacteria bacterium
D_0__Bacteria;D_1__Acidobacteria;D_2__Subgroup 22;D_3__uncultured bacterium;D_4__uncultured bacterium;D_5__uncultured bacterium
D_0__Bacteria;D_1__Acidobacteria;D_2__Subgroup 22;Other;Other;Other
D_0__Bacteria;D_1__Acidobacteria;D_2__Subgroup 5;D_3__uncultured bacterium;D_4__uncultured bacterium;D_5__uncultured bacterium
D_0__Bacteria;D_1__Acidobacteria;D_2__Subgroup 5;Other;Other;Other
D_0__Bacteria;D_1__Acidobacteria;D_2__Subgroup 6;Ambiguous_taxa;Ambiguous_taxa;Ambiguous_taxa
D_0__Bacteria;D_1__Acidobacteria;D_2__Subgroup 6;D_3__uncultured Acidobacteria bacterium;D_4__uncultured Acidobacteria bacterium;D_5__uncultured Acidobacteria bacterium
D_0__Bacteria;D_1__Acidobacteria;D_2__Subgroup 6;D_3__uncultured bacterium;D_4__uncultured bacterium;D_5__uncultured bacterium
D_0__Bacteria;D_1__Acidobacteria;D_2__Subgroup 6;D_3__uncultured organism;D_4__uncultured organism;D_5__uncultured organism
D_0__Bacteria;D_1__Acidobacteria;D_2__Subgroup 6;D_3__uncultured soil bacterium;D_4__uncultured soil bacterium;D_5__uncultured soil bacterium
D_0__Bacteria;D_1__Acidobacteria;D_2__Subgroup 6;Other;Other;Other
D_0__Bacteria;D_1__Acidobacteria;Other;Other;Other;Other
D_0__Bacteria;D_1__Actinobacteria;D_2__Acidimicrobiia;D_3__Acidimicrobiales;D_4__Acidimicrobiaceae;D_5__CL500-29 marine group
D_0__Bacteria;D_1__Actinobacteria;D_2__Acidimicrobiia;D_3__Acidimicrobiales;D_4__Acidimicrobiaceae;D_5__Ilumatobacter
D_0__Bacteria;D_1__Actinobacteria;D_2__Acidimicrobiia;D_3__Acidimicrobiales;D_4__Acidimicrobiaceae;D_5__uncultured
D_0__Bacteria;D_1__Actinobacteria;D_2__Acidimicrobiia;D_3__Acidimicrobiales;D_4__Acidimicrobiaceae;Other
D_0__Bacteria;D_1__Actinobacteria;D_2__Acidimicrobiia;D_3__Acidimicrobiales;D_4__Acidimicrobiales Incertae Sedis;D_5__Aciditerrimonas
D_0__Bacteria;D_1__Actinobacteria;D_2__Acidimicrobiia;D_3__Acidimicrobiales;D_4__Iamiaceae;D_5__Iamia
D_0__Bacteria;D_1__Actinobacteria;D_2__Acidimicrobiia;D_3__Acidimicrobiales;D_4__OM1 clade;Ambiguous_taxa
D_0__Bacteria;D_1__Actinobacteria;D_2__Acidimicrobiia;D_3__Acidimicrobiales;D_4__OM1 clade;D_5__uncultured actinobacterium
D_0__Bacteria;D_1__Actinobacteria;D_2__Acidimicrobiia;D_3__Acidimicrobiales;D_4__OM1 clade;D_5__uncultured bacterium
D_0__Bacteria;D_1__Actinobacteria;D_2__Acidimicrobiia;D_3__Acidimicrobiales;D_4__OM1 clade;D_5__uncultured compost bacterium
D_0__Bacteria;D_1__Actinobacteria;D_2__Acidimicrobiia;D_3__Acidimicrobiales;D_4__OM1 clade;Other
D_0__Bacteria;D_1__Actinobacteria;D_2__Acidimicrobiia;D_3__Acidimicrobiales;D_4__Sva0996 marine group;D_5__uncultured bacterium
D_0__Bacteria;D_1__Actinobacteria;D_2__Acidimicrobiia;D_3__Acidimicrobiales;D_4__Sva0996 marine group;Other
D_0__Bacteria;D_1__Actinobacteria;D_2__Acidimicrobiia;D_3__Acidimicrobiales;D_4__uncultured;Ambiguous_taxa
D_0__Bacteria;D_1__Actinobacteria;D_2__Acidimicrobiia;D_3__Acidimicrobiales;D_4__uncultured;D_5__uncultured Acidimicrobidae bacterium
D_0__Bacteria;D_1__Actinobacteria;D_2__Acidimicrobiia;D_3__Acidimicrobiales;D_4__uncultured;D_5__uncultured Actinomycetales bacterium
D_0__Bacteria;D_1__Actinobacteria;D_2__Acidimicrobiia;D_3__Acidimicrobiales;D_4__uncultured;D_5__uncultured actinobacterium
D_0__Bacteria;D_1__Actinobacteria;D_2__Acidimicrobiia;D_3__Acidimicrobiales;D_4__uncultured;D_5__uncultured bacterium
D_0__Bacteria;D_1__Actinobacteria;D_2__Acidimicrobiia;D_3__Acidimicrobiales;D_4__uncultured;Other
D_0__Bacteria;D_1__Actinobacteria;D_2__Acidimicrobiia;D_3__Acidimicrobiales;Other;Other
D_0__Bacteria;D_1__Actinobacteria;D_2__Actinobacteria;D_3__Actinomycetales;D_4__Actinomycetaceae;D_5__Actinomyces
D_0__Bacteria;D_1__Actinobacteria;D_2__Actinobacteria;D_3__Actinomycetales;D_4__Actinomycetaceae;D_5__Actinotignum
D_0__Bacteria;D_1__Actinobacteria;D_2__Actinobacteria;D_3__Actinomycetales;D_4__Actinomycetaceae;D_5__Mobiluncus
D_0__Bacteria;D_1__Actinobacteria;D_2__Actinobacteria;D_3__Actinomycetales;D_4__Actinomycetaceae;D_5__Varibaculum
D_0__Bacteria;D_1__Actinobacteria;D_2__Actinobacteria;D_3__Actinomycetales;D_4__Actinomycetaceae;D_5__uncultured
D_0__Bacteria;D_1__Actinobacteria;D_2__Actinobacteria;D_3__Actinopolysporales;D_4__Actinopolysporaceae;D_5__Actinopolyspora
D_0__Bacteria;D_1__Actinobacteria;D_2__Actinobacteria;D_3__Bifidobacteriales;D_4__Bifidobacteriaceae;D_5__Bifidobacterium
D_0__Bacteria;D_1__Actinobacteria;D_2__Actinobacteria;D_3__Corynebacteriales;D_4__Corynebacteriaceae;D_5__Corynebacterium
D_0__Bacteria;D_1__Actinobacteria;D_2__Actinobacteria;D_3__Corynebacteriales;D_4__Corynebacteriaceae;D_5__Corynebacterium 1
D_0__Bacteria;D_1__Actinobacteria;D_2__Actinobacteria;D_3__Corynebacteriales;D_4__Corynebacteriaceae;D_5__Lawsonella
D_0__Bacteria;D_1__Actinobacteria;D_2__Actinobacteria;D_3__Corynebacteriales;D_4__Dietziaceae;D_5__Dietzia
D_0__Bacteria;D_1__Actinobacteria;D_2__Actinobacteria;D_3__Corynebacteriales;D_4__Mycobacteriaceae;D_5__Mycobacterium
D_0__Bacteria;D_1__Actinobacteria;D_2__Actinobacteria;D_3__Corynebacteriales;D_4__Nocardiaceae;D_5__Nocardia
D_0__Bacteria;D_1__Actinobacteria;D_2__Actinobacteria;D_3__Corynebacteriales;D_4__Nocardiaceae;D_5__Rhodococcus
D_0__Bacteria;D_1__Actinobacteria;D_2__Actinobacteria;D_3__Corynebacteriales;Other;Other
D_0__Bacteria;D_1__Actinobacteria;D_2__Actinobacteria;D_3__Elev-16S-976;D_4__uncultured Nakamurellaceae bacterium;D_5__uncultured Nakamurellaceae bacterium
D_0__Bacteria;D_1__Actinobacteria;D_2__Actinobacteria;D_3__Frankiales;D_4__Acidothermaceae;D_5__Acidothermus
D_0__Bacteria;D_1__Actinobacteria;D_2__Actinobacteria;D_3__Frankiales;D_4__Cryptosporangiaceae;D_5__Fodinicola
D_0__Bacteria;D_1__Actinobacteria;D_2__Actinobacteria;D_3__Frankiales;D_4__Frankiaceae;D_5__Frankia
D_0__Bacteria;D_1__Actinobacteria;D_2__Actinobacteria;D_3__Frankiales;D_4__Frankiaceae;D_5__Jatrophihabitans
D_0__Bacteria;D_1__Actinobacteria;D_2__Actinobacteria;D_3__Frankiales;D_4__Frankiaceae;Other
D_0__Bacteria;D_1__Actinobacteria;D_2__Actinobacteria;D_3__Frankiales;D_4__Geodermatophilaceae;Ambiguous_taxa
D_0__Bacteria;D_1__Actinobacteria;D_2__Actinobacteria;D_3__Frankiales;D_4__Geodermatophilaceae;D_5__Blastococcus
D_0__Bacteria;D_1__Actinobacteria;D_2__Actinobacteria;D_3__Frankiales;D_4__Geodermatophilaceae;D_5__Geodermatophilus
D_0__Bacteria;D_1__Actinobacteria;D_2__Actinobacteria;D_3__Frankiales;D_4__Geodermatophilaceae;D_5__Modestobacter
D_0__Bacteria;D_1__Actinobacteria;D_2__Actinobacteria;D_3__Frankiales;D_4__Geodermatophilaceae;D_5__uncultured
D_0__Bacteria;D_1__Actinobacteria;D_2__Actinobacteria;D_3__Frankiales;D_4__Geodermatophilaceae;Other
D_0__Bacteria;D_1__Actinobacteria;D_2__Actinobacteria;D_3__Frankiales;D_4__Nakamurellaceae;D_5__Nakamurella
D_0__Bacteria;D_1__Actinobacteria;D_2__Actinobacteria;D_3__Frankiales;D_4__Sporichthyaceae;D_5__Sporichthya
D_0__Bacteria;D_1__Actinobacteria;D_2__Actinobacteria;D_3__Frankiales;D_4__Sporichthyaceae;D_5__uncultured
D_0__Bacteria;D_1__Actinobacteria;D_2__Actinobacteria;D_3__Frankiales;D_4__Sporichthyaceae;Other
D_0__Bacteria;D_1__Actinobacteria;D_2__Actinobacteria;D_3__Frankiales;D_4__uncultured;Ambiguous_taxa
D_0__Bacteria;D_1__Actinobacteria;D_2__Actinobacteria;D_3__Frankiales;D_4__uncultured;D_5__uncultured Sporichthyaceae bacterium
D_0__Bacteria;D_1__Actinobacteria;D_2__Actinobacteria;D_3__Frankiales;D_4__uncultured;D_5__uncultured actinobacterium
D_0__Bacteria;D_1__Actinobacteria;D_2__Actinobacteria;D_3__Frankiales;D_4__uncultured;D_5__uncultured bacterium
D_0__Bacteria;D_1__Actinobacteria;D_2__Actinobacteria;D_3__Frankiales;D_4__uncultured;Other
D_0__Bacteria;D_1__Actinobacteria;D_2__Actinobacteria;D_3__Frankiales;Other;Other
D_0__Bacteria;D_1__Actinobacteria;D_2__Actinobacteria;D_3__Glycomycetales;D_4__Glycomycetaceae;D_5__Glycomyces
D_0__Bacteria;D_1__Actinobacteria;D_2__Actinobacteria;D_3__Kineosporiales;D_4__Kineosporiaceae;D_5__Angustibacter
D_0__Bacteria;D_1__Actinobacteria;D_2__Actinobacteria;D_3__Kineosporiales;D_4__Kineosporiaceae;D_5__Kineococcus
D_0__Bacteria;D_1__Actinobacteria;D_2__Actinobacteria;D_3__Kineosporiales;D_4__Kineosporiaceae;D_5__Quadrisphaera
D_0__Bacteria;D_1__Actinobacteria;D_2__Actinobacteria;D_3__Kineosporiales;D_4__Kineosporiaceae;D_5__uncultured
D_0__Bacteria;D_1__Actinobacteria;D_2__Actinobacteria;D_3__Kineosporiales;D_4__Kineosporiaceae;Other
D_0__Bacteria;D_1__Actinobacteria;D_2__Actinobacteria;D_3__Micrococcales;D_4__Beutenbergiaceae;Ambiguous_taxa
D_0__Bacteria;D_1__Actinobacteria;D_2__Actinobacteria;D_3__Micrococcales;D_4__Bogoriellaceae;D_5__Georgenia
D_0__Bacteria;D_1__Actinobacteria;D_2__Actinobacteria;D_3__Micrococcales;D_4__Bogoriellaceae;Other
D_0__Bacteria;D_1__Actinobacteria;D_2__Actinobacteria;D_3__Micrococcales;D_4__Cellulomonadaceae;Ambiguous_taxa
D_0__Bacteria;D_1__Actinobacteria;D_2__Actinobacteria;D_3__Micrococcales;D_4__Cellulomonadaceae;D_5__Actinotalea
D_0__Bacteria;D_1__Actinobacteria;D_2__Actinobacteria;D_3__Micrococcales;D_4__Cellulomonadaceae;D_5__Cellulomonas
D_0__Bacteria;D_1__Actinobacteria;D_2__Actinobacteria;D_3__Micrococcales;D_4__Cellulomonadaceae;Other
D_0__Bacteria;D_1__Actinobacteria;D_2__Actinobacteria;D_3__Micrococcales;D_4__Demequinaceae;Ambiguous_taxa
D_0__Bacteria;D_1__Actinobacteria;D_2__Actinobacteria;D_3__Micrococcales;D_4__Demequinaceae;Other
D_0__Bacteria;D_1__Actinobacteria;D_2__Actinobacteria;D_3__Micrococcales;D_4__Dermabacteraceae;D_5__Brachybacterium
D_0__Bacteria;D_1__Actinobacteria;D_2__Actinobacteria;D_3__Micrococcales;D_4__Dermabacteraceae;D_5__Dermabacter
D_0__Bacteria;D_1__Actinobacteria;D_2__Actinobacteria;D_3__Micrococcales;D_4__Dermacoccaceae;Other
D_0__Bacteria;D_1__Actinobacteria;D_2__Actinobacteria;D_3__Micrococcales;D_4__Dermatophilaceae;D_5__Dermatophilus
D_0__Bacteria;D_1__Actinobacteria;D_2__Actinobacteria;D_3__Micrococcales;D_4__Intrasporangiaceae;Ambiguous_taxa
D_0__Bacteria;D_1__Actinobacteria;D_2__Actinobacteria;D_3__Micrococcales;D_4__Intrasporangiaceae;D_5__Knoellia
D_0__Bacteria;D_1__Actinobacteria;D_2__Actinobacteria;D_3__Micrococcales;D_4__Intrasporangiaceae;D_5__Lapillicoccus
D_0__Bacteria;D_1__Actinobacteria;D_2__Actinobacteria;D_3__Micrococcales;D_4__Intrasporangiaceae;D_5__Ornithinicoccus
D_0__Bacteria;D_1__Actinobacteria;D_2__Actinobacteria;D_3__Micrococcales;D_4__Intrasporangiaceae;D_5__Ornithinimicrobium
D_0__Bacteria;D_1__Actinobacteria;D_2__Actinobacteria;D_3__Micrococcales;D_4__Intrasporangiaceae;D_5__Oryzihumus
D_0__Bacteria;D_1__Actinobacteria;D_2__Actinobacteria;D_3__Micrococcales;D_4__Intrasporangiaceae;D_5__Terrabacter
D_0__Bacteria;D_1__Actinobacteria;D_2__Actinobacteria;D_3__Micrococcales;D_4__Intrasporangiaceae;D_5__Tetrasphaera
D_0__Bacteria;D_1__Actinobacteria;D_2__Actinobacteria;D_3__Micrococcales;D_4__Intrasporangiaceae;Other
D_0__Bacteria;D_1__Actinobacteria;D_2__Actinobacteria;D_3__Micrococcales;D_4__Microbacteriaceae;Ambiguous_taxa
D_0__Bacteria;D_1__Actinobacteria;D_2__Actinobacteria;D_3__Micrococcales;D_4__Microbacteriaceae;D_5__Agrococcus
D_0__Bacteria;D_1__Actinobacteria;D_2__Actinobacteria;D_3__Micrococcales;D_4__Microbacteriaceae;D_5__Agromyces
D_0__Bacteria;D_1__Actinobacteria;D_2__Actinobacteria;D_3__Micrococcales;D_4__Microbacteriaceae;D_5__Clavibacter
D_0__Bacteria;D_1__Actinobacteria;D_2__Actinobacteria;D_3__Micrococcales;D_4__Microbacteriaceae;D_5__Curtobacterium
D_0__Bacteria;D_1__Actinobacteria;D_2__Actinobacteria;D_3__Micrococcales;D_4__Microbacteriaceae;D_5__Frigoribacterium
D_0__Bacteria;D_1__Actinobacteria;D_2__Actinobacteria;D_3__Micrococcales;D_4__Microbacteriaceae;D_5__Leifsonia
D_0__Bacteria;D_1__Actinobacteria;D_2__Actinobacteria;D_3__Micrococcales;D_4__Microbacteriaceae;D_5__Leucobacter
D_0__Bacteria;D_1__Actinobacteria;D_2__Actinobacteria;D_3__Micrococcales;D_4__Microbacteriaceae;D_5__Microbacterium
D_0__Bacteria;D_1__Actinobacteria;D_2__Actinobacteria;D_3__Micrococcales;D_4__Microbacteriaceae;D_5__Pseudoclavibacter
D_0__Bacteria;D_1__Actinobacteria;D_2__Actinobacteria;D_3__Micrococcales;D_4__Microbacteriaceae;Other
D_0__Bacteria;D_1__Actinobacteria;D_2__Actinobacteria;D_3__Micrococcales;D_4__Micrococcaceae;Ambiguous_taxa
D_0__Bacteria;D_1__Actinobacteria;D_2__Actinobacteria;D_3__Micrococcales;D_4__Micrococcaceae;D_5__Arthrobacter
D_0__Bacteria;D_1__Actinobacteria;D_2__Actinobacteria;D_3__Micrococcales;D_4__Micrococcaceae;D_5__Kocuria
D_0__Bacteria;D_1__Actinobacteria;D_2__Actinobacteria;D_3__Micrococcales;D_4__Micrococcaceae;D_5__Micrococcus
D_0__Bacteria;D_1__Actinobacteria;D_2__Actinobacteria;D_3__Micrococcales;D_4__Micrococcaceae;D_5__Paeniglutamicibacter
D_0__Bacteria;D_1__Actinobacteria;D_2__Actinobacteria;D_3__Micrococcales;D_4__Micrococcaceae;D_5__Pseudarthrobacter
D_0__Bacteria;D_1__Actinobacteria;D_2__Actinobacteria;D_3__Micrococcales;D_4__Micrococcaceae;D_5__Rothia
D_0__Bacteria;D_1__Actinobacteria;D_2__Actinobacteria;D_3__Micrococcales;D_4__Micrococcaceae;D_5__Zhihengliuella
D_0__Bacteria;D_1__Actinobacteria;D_2__Actinobacteria;D_3__Micrococcales;D_4__Micrococcaceae;Other
D_0__Bacteria;D_1__Actinobacteria;D_2__Actinobacteria;D_3__Micrococcales;D_4__Promicromonosporaceae;D_5__Cellulosimicrobium
D_0__Bacteria;D_1__Actinobacteria;D_2__Actinobacteria;D_3__Micrococcales;D_4__Promicromonosporaceae;D_5__Isoptericola
D_0__Bacteria;D_1__Actinobacteria;D_2__Actinobacteria;D_3__Micrococcales;D_4__Promicromonosporaceae;D_5__Myceligenerans
D_0__Bacteria;D_1__Actinobacteria;D_2__Actinobacteria;D_3__Micrococcales;D_4__Promicromonosporaceae;D_5__Promicromonospora
D_0__Bacteria;D_1__Actinobacteria;D_2__Actinobacteria;D_3__Micrococcales;D_4__Promicromonosporaceae;D_5__Xylanimonas
D_0__Bacteria;D_1__Actinobacteria;D_2__Actinobacteria;D_3__Micrococcales;D_4__Promicromonosporaceae;Other
D_0__Bacteria;D_1__Actinobacteria;D_2__Actinobacteria;D_3__Micrococcales;Other;Other
D_0__Bacteria;D_1__Actinobacteria;D_2__Actinobacteria;D_3__Micromonosporales;D_4__Micromonosporaceae;Ambiguous_taxa
D_0__Bacteria;D_1__Actinobacteria;D_2__Actinobacteria;D_3__Micromonosporales;D_4__Micromonosporaceae;D_5__Actinocatenispora
D_0__Bacteria;D_1__Actinobacteria;D_2__Actinobacteria;D_3__Micromonosporales;D_4__Micromonosporaceae;D_5__Actinoplanes
D_0__Bacteria;D_1__Actinobacteria;D_2__Actinobacteria;D_3__Micromonosporales;D_4__Micromonosporaceae;D_5__Allocatelliglobosispora
D_0__Bacteria;D_1__Actinobacteria;D_2__Actinobacteria;D_3__Micromonosporales;D_4__Micromonosporaceae;D_5__Asanoa
D_0__Bacteria;D_1__Actinobacteria;D_2__Actinobacteria;D_3__Micromonosporales;D_4__Micromonosporaceae;D_5__Catellatospora
D_0__Bacteria;D_1__Actinobacteria;D_2__Actinobacteria;D_3__Micromonosporales;D_4__Micromonosporaceae;D_5__Dactylosporangium
D_0__Bacteria;D_1__Actinobacteria;D_2__Actinobacteria;D_3__Micromonosporales;D_4__Micromonosporaceae;D_5__Hamadaea
D_0__Bacteria;D_1__Actinobacteria;D_2__Actinobacteria;D_3__Micromonosporales;D_4__Micromonosporaceae;D_5__Longispora
D_0__Bacteria;D_1__Actinobacteria;D_2__Actinobacteria;D_3__Micromonosporales;D_4__Micromonosporaceae;D_5__Micromonospora
D_0__Bacteria;D_1__Actinobacteria;D_2__Actinobacteria;D_3__Micromonosporales;D_4__Micromonosporaceae;D_5__Salinispora
D_0__Bacteria;D_1__Actinobacteria;D_2__Actinobacteria;D_3__Micromonosporales;D_4__Micromonosporaceae;D_5__Stackebrandtia
D_0__Bacteria;D_1__Actinobacteria;D_2__Actinobacteria;D_3__Micromonosporales;D_4__Micromonosporaceae;D_5__uncultured
D_0__Bacteria;D_1__Actinobacteria;D_2__Actinobacteria;D_3__Micromonosporales;D_4__Micromonosporaceae;Other
D_0__Bacteria;D_1__Actinobacteria;D_2__Actinobacteria;D_3__PeM15;D_4__uncultured bacterium;D_5__uncultured bacterium
D_0__Bacteria;D_1__Actinobacteria;D_2__Actinobacteria;D_3__Propionibacteriales;D_4__Nocardioidaceae;D_5__Actinopolymorpha
D_0__Bacteria;D_1__Actinobacteria;D_2__Actinobacteria;D_3__Propionibacteriales;D_4__Nocardioidaceae;D_5__Aeromicrobium
D_0__Bacteria;D_1__Actinobacteria;D_2__Actinobacteria;D_3__Propionibacteriales;D_4__Nocardioidaceae;D_5__Flindersiella
D_0__Bacteria;D_1__Actinobacteria;D_2__Actinobacteria;D_3__Propionibacteriales;D_4__Nocardioidaceae;D_5__Kribbella
D_0__Bacteria;D_1__Actinobacteria;D_2__Actinobacteria;D_3__Propionibacteriales;D_4__Nocardioidaceae;D_5__Marmoricola
D_0__Bacteria;D_1__Actinobacteria;D_2__Actinobacteria;D_3__Propionibacteriales;D_4__Nocardioidaceae;D_5__Mumia
D_0__Bacteria;D_1__Actinobacteria;D_2__Actinobacteria;D_3__Propionibacteriales;D_4__Nocardioidaceae;D_5__Nocardioides
D_0__Bacteria;D_1__Actinobacteria;D_2__Actinobacteria;D_3__Propionibacteriales;D_4__Nocardioidaceae;D_5__uncultured
D_0__Bacteria;D_1__Actinobacteria;D_2__Actinobacteria;D_3__Propionibacteriales;D_4__Nocardioidaceae;Other
D_0__Bacteria;D_1__Actinobacteria;D_2__Actinobacteria;D_3__Propionibacteriales;D_4__Propionibacteriaceae;Ambiguous_taxa
D_0__Bacteria;D_1__Actinobacteria;D_2__Actinobacteria;D_3__Propionibacteriales;D_4__Propionibacteriaceae;D_5__Haloactinopolyspora
D_0__Bacteria;D_1__Actinobacteria;D_2__Actinobacteria;D_3__Propionibacteriales;D_4__Propionibacteriaceae;D_5__Jiangella
D_0__Bacteria;D_1__Actinobacteria;D_2__Actinobacteria;D_3__Propionibacteriales;D_4__Propionibacteriaceae;D_5__Phytoactinopolyspora
D_0__Bacteria;D_1__Actinobacteria;D_2__Actinobacteria;D_3__Propionibacteriales;D_4__Propionibacteriaceae;D_5__Propionibacterium
D_0__Bacteria;D_1__Actinobacteria;D_2__Actinobacteria;D_3__Propionibacteriales;D_4__Propionibacteriaceae;D_5__Propioniciclava
D_0__Bacteria;D_1__Actinobacteria;D_2__Actinobacteria;D_3__Propionibacteriales;D_4__Propionibacteriaceae;D_5__uncultured
D_0__Bacteria;D_1__Actinobacteria;D_2__Actinobacteria;D_3__Propionibacteriales;D_4__Propionibacteriaceae;Other
D_0__Bacteria;D_1__Actinobacteria;D_2__Actinobacteria;D_3__Propionibacteriales;Other;Other
D_0__Bacteria;D_1__Actinobacteria;D_2__Actinobacteria;D_3__Pseudonocardiales;D_4__Pseudonocardiaceae;Ambiguous_taxa
D_0__Bacteria;D_1__Actinobacteria;D_2__Actinobacteria;D_3__Pseudonocardiales;D_4__Pseudonocardiaceae;D_5__Actinoalloteichus
D_0__Bacteria;D_1__Actinobacteria;D_2__Actinobacteria;D_3__Pseudonocardiales;D_4__Pseudonocardiaceae;D_5__Actinokineospora
D_0__Bacteria;D_1__Actinobacteria;D_2__Actinobacteria;D_3__Pseudonocardiales;D_4__Pseudonocardiaceae;D_5__Actinomycetospora
D_0__Bacteria;D_1__Actinobacteria;D_2__Actinobacteria;D_3__Pseudonocardiales;D_4__Pseudonocardiaceae;D_5__Actinophytocola
D_0__Bacteria;D_1__Actinobacteria;D_2__Actinobacteria;D_3__Pseudonocardiales;D_4__Pseudonocardiaceae;D_5__Amycolatopsis
D_0__Bacteria;D_1__Actinobacteria;D_2__Actinobacteria;D_3__Pseudonocardiales;D_4__Pseudonocardiaceae;D_5__Crossiella
D_0__Bacteria;D_1__Actinobacteria;D_2__Actinobacteria;D_3__Pseudonocardiales;D_4__Pseudonocardiaceae;D_5__Kutzneria
D_0__Bacteria;D_1__Actinobacteria;D_2__Actinobacteria;D_3__Pseudonocardiales;D_4__Pseudonocardiaceae;D_5__Longimycelium
D_0__Bacteria;D_1__Actinobacteria;D_2__Actinobacteria;D_3__Pseudonocardiales;D_4__Pseudonocardiaceae;D_5__Prauserella
D_0__Bacteria;D_1__Actinobacteria;D_2__Actinobacteria;D_3__Pseudonocardiales;D_4__Pseudonocardiaceae;D_5__Pseudonocardia
D_0__Bacteria;D_1__Actinobacteria;D_2__Actinobacteria;D_3__Pseudonocardiales;D_4__Pseudonocardiaceae;D_5__Saccharopolyspora
D_0__Bacteria;D_1__Actinobacteria;D_2__Actinobacteria;D_3__Pseudonocardiales;D_4__Pseudonocardiaceae;D_5__Saccharothrix
D_0__Bacteria;D_1__Actinobacteria;D_2__Actinobacteria;D_3__Pseudonocardiales;D_4__Pseudonocardiaceae;D_5__Sciscionella
D_0__Bacteria;D_1__Actinobacteria;D_2__Actinobacteria;D_3__Pseudonocardiales;D_4__Pseudonocardiaceae;D_5__Thermocrispum
D_0__Bacteria;D_1__Actinobacteria;D_2__Actinobacteria;D_3__Pseudonocardiales;D_4__Pseudonocardiaceae;D_5__Thermotunica
D_0__Bacteria;D_1__Actinobacteria;D_2__Actinobacteria;D_3__Pseudonocardiales;D_4__Pseudonocardiaceae;D_5__Umezawaea
D_0__Bacteria;D_1__Actinobacteria;D_2__Actinobacteria;D_3__Pseudonocardiales;D_4__Pseudonocardiaceae;D_5__Yuhushiella
D_0__Bacteria;D_1__Actinobacteria;D_2__Actinobacteria;D_3__Pseudonocardiales;D_4__Pseudonocardiaceae;Other
D_0__Bacteria;D_1__Actinobacteria;D_2__Actinobacteria;D_3__Streptomycetales;D_4__Streptomycetaceae;D_5__Kitasatospora
D_0__Bacteria;D_1__Actinobacteria;D_2__Actinobacteria;D_3__Streptomycetales;D_4__Streptomycetaceae;D_5__Streptomyces
D_0__Bacteria;D_1__Actinobacteria;D_2__Actinobacteria;D_3__Streptosporangiales;D_4__Nocardiopsaceae;D_5__Nocardiopsis
D_0__Bacteria;D_1__Actinobacteria;D_2__Actinobacteria;D_3__Streptosporangiales;D_4__Nocardiopsaceae;D_5__Thermobifida
D_0__Bacteria;D_1__Actinobacteria;D_2__Actinobacteria;D_3__Streptosporangiales;D_4__Nocardiopsaceae;Other
D_0__Bacteria;D_1__Actinobacteria;D_2__Actinobacteria;D_3__Streptosporangiales;D_4__Streptosporangiaceae;Ambiguous_taxa
D_0__Bacteria;D_1__Actinobacteria;D_2__Actinobacteria;D_3__Streptosporangiales;D_4__Streptosporangiaceae;D_5__Microtetraspora
D_0__Bacteria;D_1__Actinobacteria;D_2__Actinobacteria;D_3__Streptosporangiales;D_4__Streptosporangiaceae;D_5__Nonomuraea
D_0__Bacteria;D_1__Actinobacteria;D_2__Actinobacteria;D_3__Streptosporangiales;D_4__Streptosporangiaceae;D_5__Sphaerimonospora
D_0__Bacteria;D_1__Actinobacteria;D_2__Actinobacteria;D_3__Streptosporangiales;D_4__Streptosporangiaceae;D_5__Streptosporangium
D_0__Bacteria;D_1__Actinobacteria;D_2__Actinobacteria;D_3__Streptosporangiales;D_4__Streptosporangiaceae;Other
D_0__Bacteria;D_1__Actinobacteria;D_2__Actinobacteria;D_3__Streptosporangiales;D_4__Thermomonosporaceae;D_5__Actinoallomurus
D_0__Bacteria;D_1__Actinobacteria;D_2__Actinobacteria;D_3__Streptosporangiales;D_4__Thermomonosporaceae;D_5__Actinomadura
D_0__Bacteria;D_1__Actinobacteria;D_2__Actinobacteria;D_3__Streptosporangiales;D_4__Thermomonosporaceae;Other
D_0__Bacteria;D_1__Actinobacteria;D_2__Actinobacteria;D_3__Streptosporangiales;D_4__uncultured bacterium;D_5__uncultured bacterium
D_0__Bacteria;D_1__Actinobacteria;D_2__Actinobacteria;D_3__Streptosporangiales;Other;Other
D_0__Bacteria;D_1__Actinobacteria;D_2__Actinobacteria;Other;Other;Other
D_0__Bacteria;D_1__Actinobacteria;D_2__Coriobacteriia;D_3__Coriobacteriales;D_4__Coriobacteriaceae;D_5__Atopobium
D_0__Bacteria;D_1__Actinobacteria;D_2__Coriobacteriia;D_3__Coriobacteriales;D_4__Coriobacteriaceae;D_5__uncultured
D_0__Bacteria;D_1__Actinobacteria;D_2__MB-A2-108;Ambiguous_taxa;Ambiguous_taxa;Ambiguous_taxa
D_0__Bacteria;D_1__Actinobacteria;D_2__MB-A2-108;D_3__uncultured bacterium;D_4__uncultured bacterium;D_5__uncultured bacterium
D_0__Bacteria;D_1__Actinobacteria;D_2__MB-A2-108;Other;Other;Other
D_0__Bacteria;D_1__Actinobacteria;D_2__Nitriliruptoria;D_3__Euzebyales;D_4__Euzebyaceae;D_5__Egibacter
D_0__Bacteria;D_1__Actinobacteria;D_2__Nitriliruptoria;D_3__Euzebyales;D_4__Euzebyaceae;D_5__Euzebya
D_0__Bacteria;D_1__Actinobacteria;D_2__Nitriliruptoria;D_3__Euzebyales;D_4__Euzebyaceae;D_5__uncultured
D_0__Bacteria;D_1__Actinobacteria;D_2__Nitriliruptoria;D_3__Euzebyales;D_4__Euzebyaceae;Other
D_0__Bacteria;D_1__Actinobacteria;D_2__Nitriliruptoria;D_3__Nitriliruptorales;D_4__Nitriliruptoraceae;D_5__Egicoccus
D_0__Bacteria;D_1__Actinobacteria;D_2__Nitriliruptoria;D_3__Nitriliruptorales;D_4__Nitriliruptoraceae;D_5__uncultured actinobacterium
D_0__Bacteria;D_1__Actinobacteria;D_2__Nitriliruptoria;D_3__Nitriliruptorales;D_4__Nitriliruptoraceae;D_5__uncultured bacterium
D_0__Bacteria;D_1__Actinobacteria;D_2__Nitriliruptoria;D_3__Nitriliruptorales;D_4__Nitriliruptoraceae;Other
D_0__Bacteria;D_1__Actinobacteria;D_2__Nitriliruptoria;Other;Other;Other
D_0__Bacteria;D_1__Actinobacteria;D_2__OPB41;D_3__uncultured bacterium;D_4__uncultured bacterium;D_5__uncultured bacterium
D_0__Bacteria;D_1__Actinobacteria;D_2__Rubrobacteria;D_3__Rubrobacterales;D_4__Rubrobacteriaceae;D_5__Rubrobacter
D_0__Bacteria;D_1__Actinobacteria;D_2__TakashiAC-B11;D_3__uncultured Actinomycetales bacterium;D_4__uncultured Actinomycetales bacterium;D_5__uncultured Actinomycetales bacterium
D_0__Bacteria;D_1__Actinobacteria;D_2__TakashiAC-B11;D_3__uncultured actinobacterium;D_4__uncultured actinobacterium;D_5__uncultured actinobacterium
D_0__Bacteria;D_1__Actinobacteria;D_2__TakashiAC-B11;D_3__uncultured bacterium;D_4__uncultured bacterium;D_5__uncultured bacterium
D_0__Bacteria;D_1__Actinobacteria;D_2__TakashiAC-B11;Other;Other;Other
D_0__Bacteria;D_1__Actinobacteria;D_2__Thermoleophilia;D_3__Gaiellales;D_4__Gaiellaceae;D_5__Gaiella
D_0__Bacteria;D_1__Actinobacteria;D_2__Thermoleophilia;D_3__Gaiellales;D_4__uncultured;Ambiguous_taxa
D_0__Bacteria;D_1__Actinobacteria;D_2__Thermoleophilia;D_3__Gaiellales;D_4__uncultured;D_5__uncultured bacterium
D_0__Bacteria;D_1__Actinobacteria;D_2__Thermoleophilia;D_3__Gaiellales;D_4__uncultured;D_5__uncultured microorganism
D_0__Bacteria;D_1__Actinobacteria;D_2__Thermoleophilia;D_3__Gaiellales;D_4__uncultured;Other
D_0__Bacteria;D_1__Actinobacteria;D_2__Thermoleophilia;D_3__Solirubrobacterales;Ambiguous_taxa;Ambiguous_taxa
D_0__Bacteria;D_1__Actinobacteria;D_2__Thermoleophilia;D_3__Solirubrobacterales;D_4__0319-6M6;D_5__uncultured actinobacterium
D_0__Bacteria;D_1__Actinobacteria;D_2__Thermoleophilia;D_3__Solirubrobacterales;D_4__0319-6M6;D_5__uncultured bacterium
D_0__Bacteria;D_1__Actinobacteria;D_2__Thermoleophilia;D_3__Solirubrobacterales;D_4__0319-6M6;Other
D_0__Bacteria;D_1__Actinobacteria;D_2__Thermoleophilia;D_3__Solirubrobacterales;D_4__288-2;D_5__uncultured bacterium
D_0__Bacteria;D_1__Actinobacteria;D_2__Thermoleophilia;D_3__Solirubrobacterales;D_4__288-2;Other
D_0__Bacteria;D_1__Actinobacteria;D_2__Thermoleophilia;D_3__Solirubrobacterales;D_4__Elev-16S-1332;D_5__uncultured bacterium
D_0__Bacteria;D_1__Actinobacteria;D_2__Thermoleophilia;D_3__Solirubrobacterales;D_4__Elev-16S-1332;Other
D_0__Bacteria;D_1__Actinobacteria;D_2__Thermoleophilia;D_3__Solirubrobacterales;D_4__FFCH11085;Ambiguous_taxa
D_0__Bacteria;D_1__Actinobacteria;D_2__Thermoleophilia;D_3__Solirubrobacterales;D_4__Gsoil-1167;D_5__uncultured bacterium
D_0__Bacteria;D_1__Actinobacteria;D_2__Thermoleophilia;D_3__Solirubrobacterales;D_4__Gsoil-1167;Other
D_0__Bacteria;D_1__Actinobacteria;D_2__Thermoleophilia;D_3__Solirubrobacterales;D_4__Parviterribacteraceae;D_5__Parviterribacter
D_0__Bacteria;D_1__Actinobacteria;D_2__Thermoleophilia;D_3__Solirubrobacterales;D_4__Patulibacteraceae;D_5__Patulibacter
D_0__Bacteria;D_1__Actinobacteria;D_2__Thermoleophilia;D_3__Solirubrobacterales;D_4__Q3-6C1;D_5__uncultured bacterium
D_0__Bacteria;D_1__Actinobacteria;D_2__Thermoleophilia;D_3__Solirubrobacterales;D_4__S1-80;Other
D_0__Bacteria;D_1__Actinobacteria;D_2__Thermoleophilia;D_3__Solirubrobacterales;D_4__Solirubrobacteraceae;D_5__Solirubrobacter
D_0__Bacteria;D_1__Actinobacteria;D_2__Thermoleophilia;D_3__Solirubrobacterales;D_4__TM146;Ambiguous_taxa
D_0__Bacteria;D_1__Actinobacteria;D_2__Thermoleophilia;D_3__Solirubrobacterales;D_4__TM146;D_5__uncultured bacterium
D_0__Bacteria;D_1__Actinobacteria;D_2__Thermoleophilia;D_3__Solirubrobacterales;D_4__TM146;Other
D_0__Bacteria;D_1__Actinobacteria;D_2__Thermoleophilia;D_3__Solirubrobacterales;D_4__YNPFFP1;Other
D_0__Bacteria;D_1__Actinobacteria;D_2__Thermoleophilia;D_3__Solirubrobacterales;D_4__uncultured;D_5__uncultured bacterium
D_0__Bacteria;D_1__Actinobacteria;D_2__Thermoleophilia;D_3__Solirubrobacterales;Other;Other
D_0__Bacteria;D_1__Actinobacteria;D_2__Thermoleophilia;Other;Other;Other
D_0__Bacteria;D_1__Actinobacteria;Other;Other;Other;Other
D_0__Bacteria;D_1__Armatimonadetes;D_2__Armatimonadia;D_3__Armatimonadales;Ambiguous_taxa;Ambiguous_taxa
D_0__Bacteria;D_1__Armatimonadetes;D_2__Armatimonadia;D_3__Armatimonadales;D_4__uncultured Armatimonadetes bacterium;D_5__uncultured Armatimonadetes bacterium
D_0__Bacteria;D_1__Armatimonadetes;D_2__Armatimonadia;D_3__Armatimonadales;D_4__uncultured bacterium;D_5__uncultured bacterium
D_0__Bacteria;D_1__Armatimonadetes;D_2__Fimbriimonadia;D_3__Fimbriimonadales;D_4__Fimbriimonadaceae;Other
D_0__Bacteria;D_1__Armatimonadetes;D_2__uncultured;D_3__uncultured bacterium;D_4__uncultured bacterium;D_5__uncultured bacterium
D_0__Bacteria;D_1__BRC1;D_2__uncultured bacterium;D_3__uncultured bacterium;D_4__uncultured bacterium;D_5__uncultured bacterium
D_0__Bacteria;D_1__BRC1;Other;Other;Other;Other
D_0__Bacteria;D_1__Bacteroidetes;D_2__Bacteroidetes BD2-2;D_3__uncultured bacterium;D_4__uncultured bacterium;D_5__uncultured bacterium
D_0__Bacteria;D_1__Bacteroidetes;D_2__Bacteroidetes Incertae Sedis;D_3__Order II;D_4__Rhodothermaceae;D_5__Rubrivirga
D_0__Bacteria;D_1__Bacteroidetes;D_2__Bacteroidetes Incertae Sedis;D_3__Order II;D_4__Rhodothermaceae;Other
D_0__Bacteria;D_1__Bacteroidetes;D_2__Bacteroidia;D_3__Bacteroidales;D_4__Bacteroidaceae;D_5__Bacteroides
D_0__Bacteria;D_1__Bacteroidetes;D_2__Bacteroidia;D_3__Bacteroidales;D_4__Porphyromonadaceae;D_5__Parabacteroides
D_0__Bacteria;D_1__Bacteroidetes;D_2__Bacteroidia;D_3__Bacteroidales;D_4__Porphyromonadaceae;D_5__Porphyromonas
D_0__Bacteria;D_1__Bacteroidetes;D_2__Bacteroidia;D_3__Bacteroidales;D_4__Porphyromonadaceae;D_5__Proteiniphilum
D_0__Bacteria;D_1__Bacteroidetes;D_2__Bacteroidia;D_3__Bacteroidales;D_4__Porphyromonadaceae;D_5__Tannerella
D_0__Bacteria;D_1__Bacteroidetes;D_2__Bacteroidia;D_3__Bacteroidales;D_4__Prevotellaceae;D_5__Alloprevotella
D_0__Bacteria;D_1__Bacteroidetes;D_2__Bacteroidia;D_3__Bacteroidales;D_4__Prevotellaceae;D_5__Prevotella
D_0__Bacteria;D_1__Bacteroidetes;D_2__Bacteroidia;D_3__Bacteroidales;D_4__Prevotellaceae;D_5__Prevotella 2
D_0__Bacteria;D_1__Bacteroidetes;D_2__Bacteroidia;D_3__Bacteroidales;D_4__Prevotellaceae;D_5__Prevotella 7
D_0__Bacteria;D_1__Bacteroidetes;D_2__Cytophagia;D_3__Cytophagales;D_4__Cyclobacteriaceae;D_5__uncultured
D_0__Bacteria;D_1__Bacteroidetes;D_2__Cytophagia;D_3__Cytophagales;D_4__Cytophagaceae;Ambiguous_taxa
D_0__Bacteria;D_1__Bacteroidetes;D_2__Cytophagia;D_3__Cytophagales;D_4__Cytophagaceae;D_5__Adhaeribacter
D_0__Bacteria;D_1__Bacteroidetes;D_2__Cytophagia;D_3__Cytophagales;D_4__Cytophagaceae;D_5__Cytophaga
D_0__Bacteria;D_1__Bacteroidetes;D_2__Cytophagia;D_3__Cytophagales;D_4__Cytophagaceae;D_5__Hymenobacter
D_0__Bacteria;D_1__Bacteroidetes;D_2__Cytophagia;D_3__Cytophagales;D_4__Cytophagaceae;D_5__Nibribacter
D_0__Bacteria;D_1__Bacteroidetes;D_2__Cytophagia;D_3__Cytophagales;D_4__Cytophagaceae;D_5__Ohtaekwangia
D_0__Bacteria;D_1__Bacteroidetes;D_2__Cytophagia;D_3__Cytophagales;D_4__Cytophagaceae;D_5__Pontibacter
D_0__Bacteria;D_1__Bacteroidetes;D_2__Cytophagia;D_3__Cytophagales;D_4__Cytophagaceae;D_5__Rhodocytophaga
D_0__Bacteria;D_1__Bacteroidetes;D_2__Cytophagia;D_3__Cytophagales;D_4__Cytophagaceae;D_5__Sporocytophaga
D_0__Bacteria;D_1__Bacteroidetes;D_2__Cytophagia;D_3__Cytophagales;D_4__Cytophagaceae;D_5__uncultured
D_0__Bacteria;D_1__Bacteroidetes;D_2__Cytophagia;D_3__Cytophagales;D_4__Cytophagaceae;D_5__uncultured bacterium
D_0__Bacteria;D_1__Bacteroidetes;D_2__Cytophagia;D_3__Cytophagales;D_4__Flammeovirgaceae;D_5__Cesiribacter
D_0__Bacteria;D_1__Bacteroidetes;D_2__Cytophagia;D_3__Cytophagales;D_4__Flammeovirgaceae;D_5__Fulvivirga
D_0__Bacteria;D_1__Bacteroidetes;D_2__Cytophagia;D_3__Cytophagales;D_4__Flammeovirgaceae;D_5__Imperialibacter
D_0__Bacteria;D_1__Bacteroidetes;D_2__Cytophagia;D_3__Cytophagales;D_4__Flammeovirgaceae;D_5__Nafulsella
D_0__Bacteria;D_1__Bacteroidetes;D_2__Cytophagia;D_3__Cytophagales;D_4__MWH-CFBk5;D_5__uncultured bacterium
D_0__Bacteria;D_1__Bacteroidetes;D_2__Flavobacteriia;D_3__Flavobacteriales;D_4__Flavobacteriaceae;D_5__Bergeyella
D_0__Bacteria;D_1__Bacteroidetes;D_2__Flavobacteriia;D_3__Flavobacteriales;D_4__Flavobacteriaceae;D_5__Capnocytophaga
D_0__Bacteria;D_1__Bacteroidetes;D_2__Flavobacteriia;D_3__Flavobacteriales;D_4__Flavobacteriaceae;D_5__Chryseobacterium
D_0__Bacteria;D_1__Bacteroidetes;D_2__Flavobacteriia;D_3__Flavobacteriales;D_4__Flavobacteriaceae;D_5__Cloacibacterium
D_0__Bacteria;D_1__Bacteroidetes;D_2__Flavobacteriia;D_3__Flavobacteriales;D_4__Flavobacteriaceae;D_5__Empedobacter
D_0__Bacteria;D_1__Bacteroidetes;D_2__Flavobacteriia;D_3__Flavobacteriales;D_4__Flavobacteriaceae;D_5__Epilithonimonas
D_0__Bacteria;D_1__Bacteroidetes;D_2__Flavobacteriia;D_3__Flavobacteriales;D_4__Flavobacteriaceae;D_5__Flavobacterium
D_0__Bacteria;D_1__Bacteroidetes;D_2__Flavobacteriia;D_3__Flavobacteriales;D_4__Flavobacteriaceae;D_5__Gillisia
D_0__Bacteria;D_1__Bacteroidetes;D_2__Flavobacteriia;D_3__Flavobacteriales;D_4__Flavobacteriaceae;D_5__Moheibacter
D_0__Bacteria;D_1__Bacteroidetes;D_2__Flavobacteriia;D_3__Flavobacteriales;D_4__Flavobacteriaceae;D_5__Salinimicrobium
D_0__Bacteria;D_1__Bacteroidetes;D_2__Flavobacteriia;D_3__Flavobacteriales;D_4__NS9 marine group;D_5__uncultured bacterium
D_0__Bacteria;D_1__Bacteroidetes;D_2__Sphingobacteriia;D_3__Sphingobacteriales;D_4__Chitinophagaceae;D_5__Chitinophaga
D_0__Bacteria;D_1__Bacteroidetes;D_2__Sphingobacteriia;D_3__Sphingobacteriales;D_4__Chitinophagaceae;D_5__Cnuella
D_0__Bacteria;D_1__Bacteroidetes;D_2__Sphingobacteriia;D_3__Sphingobacteriales;D_4__Chitinophagaceae;D_5__Ferruginibacter
D_0__Bacteria;D_1__Bacteroidetes;D_2__Sphingobacteriia;D_3__Sphingobacteriales;D_4__Chitinophagaceae;D_5__Flavisolibacter
D_0__Bacteria;D_1__Bacteroidetes;D_2__Sphingobacteriia;D_3__Sphingobacteriales;D_4__Chitinophagaceae;D_5__Heliimonas
D_0__Bacteria;D_1__Bacteroidetes;D_2__Sphingobacteriia;D_3__Sphingobacteriales;D_4__Chitinophagaceae;D_5__Lacibacter
D_0__Bacteria;D_1__Bacteroidetes;D_2__Sphingobacteriia;D_3__Sphingobacteriales;D_4__Chitinophagaceae;D_5__Sediminibacterium
D_0__Bacteria;D_1__Bacteroidetes;D_2__Sphingobacteriia;D_3__Sphingobacteriales;D_4__Chitinophagaceae;D_5__Segetibacter
D_0__Bacteria;D_1__Bacteroidetes;D_2__Sphingobacteriia;D_3__Sphingobacteriales;D_4__Chitinophagaceae;D_5__uncultured
D_0__Bacteria;D_1__Bacteroidetes;D_2__Sphingobacteriia;D_3__Sphingobacteriales;D_4__Chitinophagaceae;Other
D_0__Bacteria;D_1__Bacteroidetes;D_2__Sphingobacteriia;D_3__Sphingobacteriales;D_4__KD3-93;Ambiguous_taxa
D_0__Bacteria;D_1__Bacteroidetes;D_2__Sphingobacteriia;D_3__Sphingobacteriales;D_4__KD3-93;D_5__uncultured bacterium
D_0__Bacteria;D_1__Bacteroidetes;D_2__Sphingobacteriia;D_3__Sphingobacteriales;D_4__Lentimicrobiaceae;Other
D_0__Bacteria;D_1__Bacteroidetes;D_2__Sphingobacteriia;D_3__Sphingobacteriales;D_4__LiUU-11-161;D_5__uncultured Bacteroidetes bacterium
D_0__Bacteria;D_1__Bacteroidetes;D_2__Sphingobacteriia;D_3__Sphingobacteriales;D_4__NS11-12 marine group;D_5__uncultured Bacteroidetes bacterium
D_0__Bacteria;D_1__Bacteroidetes;D_2__Sphingobacteriia;D_3__Sphingobacteriales;D_4__Saprospiraceae;D_5__uncultured
D_0__Bacteria;D_1__Bacteroidetes;D_2__Sphingobacteriia;D_3__Sphingobacteriales;D_4__Sphingobacteriaceae;D_5__Anseongella
D_0__Bacteria;D_1__Bacteroidetes;D_2__Sphingobacteriia;D_3__Sphingobacteriales;D_4__Sphingobacteriaceae;D_5__Mucilaginibacter
D_0__Bacteria;D_1__Bacteroidetes;D_2__Sphingobacteriia;D_3__Sphingobacteriales;D_4__Sphingobacteriaceae;D_5__Pedobacter
D_0__Bacteria;D_1__Bacteroidetes;D_2__Sphingobacteriia;D_3__Sphingobacteriales;D_4__Sphingobacteriaceae;Other
D_0__Bacteria;D_1__Bacteroidetes;D_2__Sphingobacteriia;D_3__Sphingobacteriales;D_4__env.OPS 17;Ambiguous_taxa
D_0__Bacteria;D_1__Bacteroidetes;D_2__Sphingobacteriia;D_3__Sphingobacteriales;D_4__env.OPS 17;D_5__uncultured bacterium
D_0__Bacteria;D_1__Bacteroidetes;D_2__Sphingobacteriia;D_3__Sphingobacteriales;Other;Other
D_0__Bacteria;D_1__Chlamydiae;D_2__Chlamydiae;D_3__Chlamydiales;D_4__Parachlamydiaceae;D_5__Candidatus Protochlamydia
D_0__Bacteria;D_1__Chlamydiae;D_2__Chlamydiae;D_3__Chlamydiales;D_4__Parachlamydiaceae;D_5__Neochlamydia
D_0__Bacteria;D_1__Chlamydiae;D_2__Chlamydiae;D_3__Chlamydiales;D_4__Parachlamydiaceae;D_5__Parachlamydia
D_0__Bacteria;D_1__Chlamydiae;D_2__Chlamydiae;D_3__Chlamydiales;D_4__Parachlamydiaceae;Other
D_0__Bacteria;D_1__Chlamydiae;D_2__Chlamydiae;D_3__Chlamydiales;D_4__Simkaniaceae;D_5__uncultured
D_0__Bacteria;D_1__Chlamydiae;D_2__Chlamydiae;D_3__Chlamydiales;D_4__cvE6;Ambiguous_taxa
D_0__Bacteria;D_1__Chlorobi;D_2__Chlorobia;D_3__Chlorobiales;D_4__OPB56;D_5__uncultured bacterium
D_0__Bacteria;D_1__Chlorobi;D_2__Chlorobia;D_3__Chlorobiales;D_4__SJA-28;Ambiguous_taxa
D_0__Bacteria;D_1__Chloroflexi;D_2__Anaerolineae;D_3__Anaerolineales;D_4__Anaerolineaceae;D_5__Ornatilinea
D_0__Bacteria;D_1__Chloroflexi;D_2__Anaerolineae;D_3__Anaerolineales;D_4__Anaerolineaceae;D_5__uncultured
D_0__Bacteria;D_1__Chloroflexi;D_2__Ardenticatenia;D_3__Ardenticatenales;D_4__uncultured bacterium;D_5__uncultured bacterium
D_0__Bacteria;D_1__Chloroflexi;D_2__Caldilineae;D_3__Caldilineales;D_4__Caldilineaceae;D_5__uncultured
D_0__Bacteria;D_1__Chloroflexi;D_2__Chloroflexi Incertae Sedis;D_3__Unknown Order;D_4__Unknown Family;D_5__Thermobaculum
D_0__Bacteria;D_1__Chloroflexi;D_2__Chloroflexia;D_3__Chloroflexales;D_4__Roseiflexaceae;D_5__Roseiflexus
D_0__Bacteria;D_1__Chloroflexi;D_2__Chloroflexia;D_3__Herpetosiphonales;D_4__Herpetosiphonaceae;D_5__Herpetosiphon
D_0__Bacteria;D_1__Chloroflexi;D_2__Chloroflexia;D_3__Kallotenuales;D_4__AKIW781;D_5__uncultured bacterium
D_0__Bacteria;D_1__Chloroflexi;D_2__Gitt-GS-136;Ambiguous_taxa;Ambiguous_taxa;Ambiguous_taxa
D_0__Bacteria;D_1__Chloroflexi;D_2__Gitt-GS-136;D_3__uncultured bacterium;D_4__uncultured bacterium;D_5__uncultured bacterium
D_0__Bacteria;D_1__Chloroflexi;D_2__Gitt-GS-136;D_3__uncultured bacterium #0319-6C24;D_4__uncultured bacterium #0319-6C24;D_5__uncultured bacterium #0319-6C24
D_0__Bacteria;D_1__Chloroflexi;D_2__Gitt-GS-136;Other;Other;Other
D_0__Bacteria;D_1__Chloroflexi;D_2__JG30-KF-CM66;Ambiguous_taxa;Ambiguous_taxa;Ambiguous_taxa
D_0__Bacteria;D_1__Chloroflexi;D_2__JG30-KF-CM66;D_3__uncultured Chloroflexi bacterium;D_4__uncultured Chloroflexi bacterium;D_5__uncultured Chloroflexi bacterium
D_0__Bacteria;D_1__Chloroflexi;D_2__JG30-KF-CM66;D_3__uncultured bacterium;D_4__uncultured bacterium;D_5__uncultured bacterium
D_0__Bacteria;D_1__Chloroflexi;D_2__JG30-KF-CM66;Other;Other;Other
D_0__Bacteria;D_1__Chloroflexi;D_2__JG37-AG-4;D_3__uncultured bacterium;D_4__uncultured bacterium;D_5__uncultured bacterium
D_0__Bacteria;D_1__Chloroflexi;D_2__KD4-96;Ambiguous_taxa;Ambiguous_taxa;Ambiguous_taxa
D_0__Bacteria;D_1__Chloroflexi;D_2__KD4-96;D_3__uncultured bacterium;D_4__uncultured bacterium;D_5__uncultured bacterium
D_0__Bacteria;D_1__Chloroflexi;D_2__KD4-96;Other;Other;Other
D_0__Bacteria;D_1__Chloroflexi;D_2__Ktedonobacteria;D_3__C0119;D_4__uncultured bacterium;D_5__uncultured bacterium
D_0__Bacteria;D_1__Chloroflexi;D_2__NLS2-31;D_3__uncultured bacterium;D_4__uncultured bacterium;D_5__uncultured bacterium
D_0__Bacteria;D_1__Chloroflexi;D_2__P2-11E;D_3__uncultured bacterium;D_4__uncultured bacterium;D_5__uncultured bacterium
D_0__Bacteria;D_1__Chloroflexi;D_2__S085;Ambiguous_taxa;Ambiguous_taxa;Ambiguous_taxa
D_0__Bacteria;D_1__Chloroflexi;D_2__S085;D_3__uncultured Chloroflexi bacterium;D_4__uncultured Chloroflexi bacterium;D_5__uncultured Chloroflexi bacterium
D_0__Bacteria;D_1__Chloroflexi;D_2__S085;D_3__uncultured bacterium;D_4__uncultured bacterium;D_5__uncultured bacterium
D_0__Bacteria;D_1__Chloroflexi;D_2__S085;Other;Other;Other
D_0__Bacteria;D_1__Chloroflexi;D_2__SAR202 clade;Ambiguous_taxa;Ambiguous_taxa;Ambiguous_taxa
D_0__Bacteria;D_1__Chloroflexi;D_2__SAR202 clade;D_3__uncultured Chloroflexi bacterium;D_4__uncultured Chloroflexi bacterium;D_5__uncultured Chloroflexi bacterium
D_0__Bacteria;D_1__Chloroflexi;D_2__SAR202 clade;D_3__uncultured bacterium;D_4__uncultured bacterium;D_5__uncultured bacterium
D_0__Bacteria;D_1__Chloroflexi;D_2__SAR202 clade;Other;Other;Other
D_0__Bacteria;D_1__Chloroflexi;D_2__TK10;D_3__uncultured Chloroflexi bacterium;D_4__uncultured Chloroflexi bacterium;D_5__uncultured Chloroflexi bacterium
D_0__Bacteria;D_1__Chloroflexi;D_2__TK10;D_3__uncultured bacterium;D_4__uncultured bacterium;D_5__uncultured bacterium
D_0__Bacteria;D_1__Chloroflexi;D_2__TK10;D_3__uncultured soil bacterium;D_4__uncultured soil bacterium;D_5__uncultured soil bacterium
D_0__Bacteria;D_1__Chloroflexi;D_2__TK10;Other;Other;Other
D_0__Bacteria;D_1__Chloroflexi;D_2__Thermomicrobia;D_3__AKYG1722;D_4__uncultured Chloroflexi bacterium;D_5__uncultured Chloroflexi bacterium
D_0__Bacteria;D_1__Chloroflexi;D_2__Thermomicrobia;D_3__AKYG1722;D_4__uncultured Sphaerobacter sp.;D_5__uncultured Sphaerobacter sp.
D_0__Bacteria;D_1__Chloroflexi;D_2__Thermomicrobia;D_3__AKYG1722;D_4__uncultured bacterium;D_5__uncultured bacterium
D_0__Bacteria;D_1__Chloroflexi;D_2__Thermomicrobia;D_3__AKYG1722;Other;Other
D_0__Bacteria;D_1__Chloroflexi;D_2__Thermomicrobia;D_3__JG30-KF-CM45;Ambiguous_taxa;Ambiguous_taxa
D_0__Bacteria;D_1__Chloroflexi;D_2__Thermomicrobia;D_3__JG30-KF-CM45;D_4__uncultured Chloroflexi bacterium;D_5__uncultured Chloroflexi bacterium
D_0__Bacteria;D_1__Chloroflexi;D_2__Thermomicrobia;D_3__JG30-KF-CM45;D_4__uncultured bacterium;D_5__uncultured bacterium
D_0__Bacteria;D_1__Chloroflexi;D_2__Thermomicrobia;D_3__Sphaerobacterales;D_4__Sphaerobacteraceae;D_5__Nitrolancea
D_0__Bacteria;D_1__Chloroflexi;D_2__Thermomicrobia;D_3__Sphaerobacterales;D_4__Sphaerobacteraceae;D_5__Sphaerobacter
D_0__Bacteria;D_1__Chloroflexi;Other;Other;Other;Other
D_0__Bacteria;D_1__Cyanobacteria;D_2__Chloroplast;Ambiguous_taxa;Ambiguous_taxa;Ambiguous_taxa
D_0__Bacteria;D_1__Cyanobacteria;D_2__Chloroplast;D_3__uncultured bacterium;D_4__uncultured bacterium;D_5__uncultured bacterium
D_0__Bacteria;D_1__Cyanobacteria;D_2__Cyanobacteria;D_3__SubsectionIII;D_4__FamilyI;D_5__Leptolyngbya
D_0__Bacteria;D_1__Cyanobacteria;D_2__Cyanobacteria;D_3__SubsectionIII;D_4__FamilyI;D_5__Microcoleus
D_0__Bacteria;D_1__Cyanobacteria;D_2__Cyanobacteria;D_3__uncultured;D_4__uncultured bacterium;D_5__uncultured bacterium
D_0__Bacteria;D_1__Cyanobacteria;D_2__Melainabacteria;D_3__Gastranaerophilales;Other;Other
D_0__Bacteria;D_1__Cyanobacteria;D_2__Melainabacteria;D_3__Vampirovibrionales;D_4__uncultured bacterium;D_5__uncultured bacterium
D_0__Bacteria;D_1__Deinococcus-Thermus;D_2__Deinococci;D_3__Deinococcales;D_4__Deinococcaceae;D_5__Deinococcus
D_0__Bacteria;D_1__Deinococcus-Thermus;D_2__Deinococci;D_3__Deinococcales;D_4__Trueperaceae;D_5__Truepera
D_0__Bacteria;D_1__Deinococcus-Thermus;D_2__Deinococci;D_3__Thermales;D_4__Thermaceae;D_5__Thermus
D_0__Bacteria;D_1__Elusimicrobia;D_2__Elusimicrobia;D_3__Lineage IIa;D_4__uncultured bacterium;D_5__uncultured bacterium
D_0__Bacteria;D_1__Elusimicrobia;D_2__Elusimicrobia;D_3__Lineage IIb;D_4__uncultured bacterium;D_5__uncultured bacterium
D_0__Bacteria;D_1__Elusimicrobia;D_2__Elusimicrobia;D_3__Lineage IV;D_4__uncultured bacterium;D_5__uncultured bacterium
D_0__Bacteria;D_1__FBP;D_2__uncultured bacterium;D_3__uncultured bacterium;D_4__uncultured bacterium;D_5__uncultured bacterium
D_0__Bacteria;D_1__Fibrobacteres;D_2__Fibrobacteria;D_3__Fibrobacterales;D_4__Fibrobacteraceae;D_5__possible genus 04
D_0__Bacteria;D_1__Fibrobacteres;D_2__Fibrobacteria;D_3__Fibrobacterales;D_4__Fibrobacteraceae;D_5__uncultured
D_0__Bacteria;D_1__Firmicutes;D_2__Bacilli;D_3__Bacillales;D_4__Alicyclobacillaceae;D_5__Tumebacillus
D_0__Bacteria;D_1__Firmicutes;D_2__Bacilli;D_3__Bacillales;D_4__Bacillaceae;D_5__Bacillus
D_0__Bacteria;D_1__Firmicutes;D_2__Bacilli;D_3__Bacillales;D_4__Bacillaceae;D_5__Oceanobacillus
D_0__Bacteria;D_1__Firmicutes;D_2__Bacilli;D_3__Bacillales;D_4__Bacillaceae;D_5__Virgibacillus
D_0__Bacteria;D_1__Firmicutes;D_2__Bacilli;D_3__Bacillales;D_4__Bacillaceae;Other
D_0__Bacteria;D_1__Firmicutes;D_2__Bacilli;D_3__Bacillales;D_4__Family X;D_5__Thermicanus
D_0__Bacteria;D_1__Firmicutes;D_2__Bacilli;D_3__Bacillales;D_4__Family XI;D_5__Gemella
D_0__Bacteria;D_1__Firmicutes;D_2__Bacilli;D_3__Bacillales;D_4__Paenibacillaceae;D_5__Ammoniphilus
D_0__Bacteria;D_1__Firmicutes;D_2__Bacilli;D_3__Bacillales;D_4__Paenibacillaceae;D_5__Paenibacillus
D_0__Bacteria;D_1__Firmicutes;D_2__Bacilli;D_3__Bacillales;D_4__Paenibacillaceae;D_5__Saccharibacillus
D_0__Bacteria;D_1__Firmicutes;D_2__Bacilli;D_3__Bacillales;D_4__Planococcaceae;Ambiguous_taxa
D_0__Bacteria;D_1__Firmicutes;D_2__Bacilli;D_3__Bacillales;D_4__Planococcaceae;D_5__Chryseomicrobium
D_0__Bacteria;D_1__Firmicutes;D_2__Bacilli;D_3__Bacillales;D_4__Planococcaceae;D_5__Lysinibacillus
D_0__Bacteria;D_1__Firmicutes;D_2__Bacilli;D_3__Bacillales;D_4__Planococcaceae;D_5__Paenisporosarcina
D_0__Bacteria;D_1__Firmicutes;D_2__Bacilli;D_3__Bacillales;D_4__Planococcaceae;D_5__Planococcus
D_0__Bacteria;D_1__Firmicutes;D_2__Bacilli;D_3__Bacillales;D_4__Planococcaceae;D_5__Planomicrobium
D_0__Bacteria;D_1__Firmicutes;D_2__Bacilli;D_3__Bacillales;D_4__Planococcaceae;Other
D_0__Bacteria;D_1__Firmicutes;D_2__Bacilli;D_3__Bacillales;D_4__Staphylococcaceae;D_5__Nosocomiicoccus
D_0__Bacteria;D_1__Firmicutes;D_2__Bacilli;D_3__Bacillales;D_4__Staphylococcaceae;D_5__Staphylococcus
D_0__Bacteria;D_1__Firmicutes;D_2__Bacilli;D_3__Bacillales;D_4__Thermoactinomycetaceae;D_5__Thermoactinomyces
D_0__Bacteria;D_1__Firmicutes;D_2__Bacilli;D_3__Bacillales;Other;Other
D_0__Bacteria;D_1__Firmicutes;D_2__Bacilli;D_3__Lactobacillales;D_4__Aerococcaceae;D_5__Aerococcus
D_0__Bacteria;D_1__Firmicutes;D_2__Bacilli;D_3__Lactobacillales;D_4__Aerococcaceae;D_5__Facklamia
D_0__Bacteria;D_1__Firmicutes;D_2__Bacilli;D_3__Lactobacillales;D_4__Aerococcaceae;Other
D_0__Bacteria;D_1__Firmicutes;D_2__Bacilli;D_3__Lactobacillales;D_4__Carnobacteriaceae;D_5__Dolosigranulum
D_0__Bacteria;D_1__Firmicutes;D_2__Bacilli;D_3__Lactobacillales;D_4__Carnobacteriaceae;D_5__Granulicatella
D_0__Bacteria;D_1__Firmicutes;D_2__Bacilli;D_3__Lactobacillales;D_4__Carnobacteriaceae;D_5__Marinilactibacillus
D_0__Bacteria;D_1__Firmicutes;D_2__Bacilli;D_3__Lactobacillales;D_4__Carnobacteriaceae;D_5__Trichococcus
D_0__Bacteria;D_1__Firmicutes;D_2__Bacilli;D_3__Lactobacillales;D_4__Carnobacteriaceae;Other
D_0__Bacteria;D_1__Firmicutes;D_2__Bacilli;D_3__Lactobacillales;D_4__Enterococcaceae;D_5__Enterococcus
D_0__Bacteria;D_1__Firmicutes;D_2__Bacilli;D_3__Lactobacillales;D_4__Streptococcaceae;D_5__Lactococcus
D_0__Bacteria;D_1__Firmicutes;D_2__Bacilli;D_3__Lactobacillales;D_4__Streptococcaceae;D_5__Streptococcus
D_0__Bacteria;D_1__Firmicutes;D_2__Bacilli;D_3__Lactobacillales;Other;Other
D_0__Bacteria;D_1__Firmicutes;D_2__Clostridia;D_3__Clostridiales;D_4__Clostridiaceae 1;D_5__Clostridium sensu stricto 13
D_0__Bacteria;D_1__Firmicutes;D_2__Clostridia;D_3__Clostridiales;D_4__Family XI;D_5__Anaerococcus
D_0__Bacteria;D_1__Firmicutes;D_2__Clostridia;D_3__Clostridiales;D_4__Family XI;D_5__Ezakiella
D_0__Bacteria;D_1__Firmicutes;D_2__Clostridia;D_3__Clostridiales;D_4__Family XI;D_5__Finegoldia
D_0__Bacteria;D_1__Firmicutes;D_2__Clostridia;D_3__Clostridiales;D_4__Family XI;D_5__Helcococcus
D_0__Bacteria;D_1__Firmicutes;D_2__Clostridia;D_3__Clostridiales;D_4__Family XI;D_5__Murdochiella
D_0__Bacteria;D_1__Firmicutes;D_2__Clostridia;D_3__Clostridiales;D_4__Family XI;D_5__Parvimonas
D_0__Bacteria;D_1__Firmicutes;D_2__Clostridia;D_3__Clostridiales;D_4__Family XI;D_5__Peptoniphilus
D_0__Bacteria;D_1__Firmicutes;D_2__Clostridia;D_3__Clostridiales;D_4__Family XI;D_5__uncultured
D_0__Bacteria;D_1__Firmicutes;D_2__Clostridia;D_3__Clostridiales;D_4__Family XIII;D_5__Mogibacterium
D_0__Bacteria;D_1__Firmicutes;D_2__Clostridia;D_3__Clostridiales;D_4__Family XIII;D_5__[Eubacterium] brachy group
D_0__Bacteria;D_1__Firmicutes;D_2__Clostridia;D_3__Clostridiales;D_4__Family XIII;D_5__[Eubacterium] nodatum group
D_0__Bacteria;D_1__Firmicutes;D_2__Clostridia;D_3__Clostridiales;D_4__Family XIII;D_5__[Eubacterium] saphenum group
D_0__Bacteria;D_1__Firmicutes;D_2__Clostridia;D_3__Clostridiales;D_4__Family XIII;D_5__uncultured
D_0__Bacteria;D_1__Firmicutes;D_2__Clostridia;D_3__Clostridiales;D_4__Heliobacteriaceae;D_5__Hydrogenispora
D_0__Bacteria;D_1__Firmicutes;D_2__Clostridia;D_3__Clostridiales;D_4__Lachnospiraceae;D_5__Anaerostipes
D_0__Bacteria;D_1__Firmicutes;D_2__Clostridia;D_3__Clostridiales;D_4__Lachnospiraceae;D_5__Blautia
D_0__Bacteria;D_1__Firmicutes;D_2__Clostridia;D_3__Clostridiales;D_4__Lachnospiraceae;D_5__Catonella
D_0__Bacteria;D_1__Firmicutes;D_2__Clostridia;D_3__Clostridiales;D_4__Lachnospiraceae;D_5__Coprococcus 1
D_0__Bacteria;D_1__Firmicutes;D_2__Clostridia;D_3__Clostridiales;D_4__Lachnospiraceae;D_5__Johnsonella
D_0__Bacteria;D_1__Firmicutes;D_2__Clostridia;D_3__Clostridiales;D_4__Lachnospiraceae;D_5__Lachnoanaerobaculum
D_0__Bacteria;D_1__Firmicutes;D_2__Clostridia;D_3__Clostridiales;D_4__Lachnospiraceae;D_5__Lachnospiraceae ND3007 group
D_0__Bacteria;D_1__Firmicutes;D_2__Clostridia;D_3__Clostridiales;D_4__Lachnospiraceae;D_5__Lachnospiraceae UCG-008
D_0__Bacteria;D_1__Firmicutes;D_2__Clostridia;D_3__Clostridiales;D_4__Lachnospiraceae;D_5__Oribacterium
D_0__Bacteria;D_1__Firmicutes;D_2__Clostridia;D_3__Clostridiales;D_4__Lachnospiraceae;D_5__Roseburia
D_0__Bacteria;D_1__Firmicutes;D_2__Clostridia;D_3__Clostridiales;D_4__Lachnospiraceae;D_5__Shuttleworthia
D_0__Bacteria;D_1__Firmicutes;D_2__Clostridia;D_3__Clostridiales;D_4__Lachnospiraceae;D_5__uncultured
D_0__Bacteria;D_1__Firmicutes;D_2__Clostridia;D_3__Clostridiales;D_4__Lachnospiraceae;Other
D_0__Bacteria;D_1__Firmicutes;D_2__Clostridia;D_3__Clostridiales;D_4__Peptococcaceae;D_5__Desulfosporosinus
D_0__Bacteria;D_1__Firmicutes;D_2__Clostridia;D_3__Clostridiales;D_4__Peptococcaceae;D_5__Peptococcus
D_0__Bacteria;D_1__Firmicutes;D_2__Clostridia;D_3__Clostridiales;D_4__Peptostreptococcaceae;D_5__Filifactor
D_0__Bacteria;D_1__Firmicutes;D_2__Clostridia;D_3__Clostridiales;D_4__Peptostreptococcaceae;D_5__Peptoclostridium
D_0__Bacteria;D_1__Firmicutes;D_2__Clostridia;D_3__Clostridiales;D_4__Peptostreptococcaceae;D_5__Peptostreptococcus
D_0__Bacteria;D_1__Firmicutes;D_2__Clostridia;D_3__Clostridiales;D_4__Peptostreptococcaceae;D_5__Sporacetigenium
D_0__Bacteria;D_1__Firmicutes;D_2__Clostridia;D_3__Clostridiales;D_4__Peptostreptococcaceae;D_5__[Eubacterium] yurii group
D_0__Bacteria;D_1__Firmicutes;D_2__Clostridia;D_3__Clostridiales;D_4__Peptostreptococcaceae;Other
D_0__Bacteria;D_1__Firmicutes;D_2__Clostridia;D_3__Clostridiales;D_4__Ruminococcaceae;D_5__Caproiciproducens
D_0__Bacteria;D_1__Firmicutes;D_2__Clostridia;D_3__Clostridiales;D_4__Ruminococcaceae;D_5__Faecalibacterium
D_0__Bacteria;D_1__Firmicutes;D_2__Clostridia;D_3__Clostridiales;D_4__Ruminococcaceae;D_5__Fastidiosipila
D_0__Bacteria;D_1__Firmicutes;D_2__Clostridia;D_3__Halanaerobiales;D_4__ODP1230B8.23;D_5__uncultured bacterium
D_0__Bacteria;D_1__Firmicutes;D_2__Clostridia;D_3__Thermoanaerobacterales;D_4__Thermoanaerobacteraceae;D_5__Gelria
D_0__Bacteria;D_1__Firmicutes;D_2__Erysipelotrichia;D_3__Erysipelotrichales;D_4__Erysipelotrichaceae;D_5__Erysipelotrichaceae UCG-007
D_0__Bacteria;D_1__Firmicutes;D_2__Erysipelotrichia;D_3__Erysipelotrichales;D_4__Erysipelotrichaceae;D_5__Solobacterium
D_0__Bacteria;D_1__Firmicutes;D_2__Erysipelotrichia;D_3__Erysipelotrichales;D_4__Erysipelotrichaceae;Other
D_0__Bacteria;D_1__Firmicutes;D_2__Negativicutes;D_3__Selenomonadales;D_4__Veillonellaceae;D_5__Dialister
D_0__Bacteria;D_1__Firmicutes;D_2__Negativicutes;D_3__Selenomonadales;D_4__Veillonellaceae;D_5__Selenomonas 3
D_0__Bacteria;D_1__Firmicutes;D_2__Negativicutes;D_3__Selenomonadales;D_4__Veillonellaceae;D_5__Veillonella
D_0__Bacteria;D_1__Fusobacteria;D_2__Fusobacteriia;D_3__Fusobacteriales;D_4__Fusobacteriaceae;D_5__Fusobacterium
D_0__Bacteria;D_1__Fusobacteria;D_2__Fusobacteriia;D_3__Fusobacteriales;D_4__Leptotrichiaceae;D_5__Leptotrichia
D_0__Bacteria;D_1__Gemmatimonadetes;D_2__BD2-11 terrestrial group;D_3__uncultured bacterium;D_4__uncultured bacterium;D_5__uncultured bacterium
D_0__Bacteria;D_1__Gemmatimonadetes;D_2__Gemmatimonadetes;D_3__Gemmatimonadales;D_4__Gemmatimonadaceae;D_5__Gemmatimonas
D_0__Bacteria;D_1__Gemmatimonadetes;D_2__Gemmatimonadetes;D_3__Gemmatimonadales;D_4__Gemmatimonadaceae;D_5__Gemmatirosa
D_0__Bacteria;D_1__Gemmatimonadetes;D_2__Gemmatimonadetes;D_3__Gemmatimonadales;D_4__Gemmatimonadaceae;D_5__uncultured
D_0__Bacteria;D_1__Gemmatimonadetes;D_2__Gemmatimonadetes;D_3__Gemmatimonadales;D_4__Gemmatimonadaceae;Other
D_0__Bacteria;D_1__Gemmatimonadetes;D_2__Longimicrobia;D_3__Longimicrobiales;D_4__Longimicrobiaceae;D_5__Longimicrobium
D_0__Bacteria;D_1__Gemmatimonadetes;D_2__Longimicrobia;D_3__Longimicrobiales;D_4__Longimicrobiaceae;D_5__uncultured bacterium
D_0__Bacteria;D_1__Gemmatimonadetes;D_2__S0134 terrestrial group;D_3__uncultured Gemmatimonadetes bacterium;D_4__uncultured Gemmatimonadetes bacterium;D_5__uncultured Gemmatimonadetes bacterium
D_0__Bacteria;D_1__Gemmatimonadetes;D_2__S0134 terrestrial group;D_3__uncultured bacterium;D_4__uncultured bacterium;D_5__uncultured bacterium
D_0__Bacteria;D_1__Hydrogenedentes;D_2__uncultured bacterium;D_3__uncultured bacterium;D_4__uncultured bacterium;D_5__uncultured bacterium
D_0__Bacteria;D_1__Nitrospirae;D_2__Nitrospira;D_3__Nitrospirales;D_4__0319-6A21;Other
D_0__Bacteria;D_1__Nitrospirae;D_2__Nitrospira;D_3__Nitrospirales;D_4__Nitrospiraceae;D_5__Nitrospira
D_0__Bacteria;D_1__Nitrospirae;D_2__Nitrospira;D_3__Nitrospirales;D_4__Nitrospiraceae;D_5__uncultured
D_0__Bacteria;D_1__Planctomycetes;D_2__Phycisphaerae;D_3__Phycisphaerales;D_4__Phycisphaeraceae;D_5__I-8
D_0__Bacteria;D_1__Planctomycetes;D_2__Phycisphaerae;D_3__Phycisphaerales;D_4__Phycisphaeraceae;D_5__SM1A02
D_0__Bacteria;D_1__Planctomycetes;D_2__Phycisphaerae;D_3__Phycisphaerales;D_4__Phycisphaeraceae;D_5__uncultured
D_0__Bacteria;D_1__Planctomycetes;D_2__Phycisphaerae;D_3__Tepidisphaerales;D_4__Tepidisphaeraceae;Ambiguous_taxa
D_0__Bacteria;D_1__Planctomycetes;D_2__Phycisphaerae;D_3__Tepidisphaerales;D_4__Tepidisphaeraceae;D_5__uncultured bacterium
D_0__Bacteria;D_1__Planctomycetes;D_2__Phycisphaerae;D_3__Tepidisphaerales;D_4__Tepidisphaeraceae;Other
D_0__Bacteria;D_1__Planctomycetes;D_2__Planctomycetacia;D_3__Planctomycetales;D_4__Planctomycetaceae;D_5__Blastopirellula
D_0__Bacteria;D_1__Planctomycetes;D_2__Planctomycetacia;D_3__Planctomycetales;D_4__Planctomycetaceae;D_5__Gemmata
D_0__Bacteria;D_1__Planctomycetes;D_2__Planctomycetacia;D_3__Planctomycetales;D_4__Planctomycetaceae;D_5__Isosphaera
D_0__Bacteria;D_1__Planctomycetes;D_2__Planctomycetacia;D_3__Planctomycetales;D_4__Planctomycetaceae;D_5__Pir4 lineage
D_0__Bacteria;D_1__Planctomycetes;D_2__Planctomycetacia;D_3__Planctomycetales;D_4__Planctomycetaceae;D_5__Pirellula
D_0__Bacteria;D_1__Planctomycetes;D_2__Planctomycetacia;D_3__Planctomycetales;D_4__Planctomycetaceae;D_5__Planctomyces
D_0__Bacteria;D_1__Planctomycetes;D_2__Planctomycetacia;D_3__Planctomycetales;D_4__Planctomycetaceae;D_5__Rhodopirellula
D_0__Bacteria;D_1__Planctomycetes;D_2__Planctomycetacia;D_3__Planctomycetales;D_4__Planctomycetaceae;D_5__Singulisphaera
D_0__Bacteria;D_1__Planctomycetes;D_2__Planctomycetacia;D_3__Planctomycetales;D_4__Planctomycetaceae;D_5__uncultured
D_0__Bacteria;D_1__Planctomycetes;D_2__Planctomycetacia;D_3__Planctomycetales;D_4__Planctomycetaceae;Other
D_0__Bacteria;D_1__Proteobacteria;D_2__Alphaproteobacteria;D_3__Alphaproteobacteria Incertae Sedis;D_4__Unknown Family;D_5__uncultured
D_0__Bacteria;D_1__Proteobacteria;D_2__Alphaproteobacteria;D_3__Caulobacterales;D_4__Caulobacteraceae;Ambiguous_taxa
D_0__Bacteria;D_1__Proteobacteria;D_2__Alphaproteobacteria;D_3__Caulobacterales;D_4__Caulobacteraceae;D_5__Brevundimonas
D_0__Bacteria;D_1__Proteobacteria;D_2__Alphaproteobacteria;D_3__Caulobacterales;D_4__Caulobacteraceae;D_5__Phenylobacterium
D_0__Bacteria;D_1__Proteobacteria;D_2__Alphaproteobacteria;D_3__Caulobacterales;D_4__Caulobacteraceae;D_5__uncultured
D_0__Bacteria;D_1__Proteobacteria;D_2__Alphaproteobacteria;D_3__Rhizobiales;D_4__1174-901-12;Ambiguous_taxa
D_0__Bacteria;D_1__Proteobacteria;D_2__Alphaproteobacteria;D_3__Rhizobiales;D_4__1174-901-12;D_5__uncultured Rhizobiales bacterium
D_0__Bacteria;D_1__Proteobacteria;D_2__Alphaproteobacteria;D_3__Rhizobiales;D_4__1174-901-12;Other
D_0__Bacteria;D_1__Proteobacteria;D_2__Alphaproteobacteria;D_3__Rhizobiales;D_4__A0839;D_5__uncultured bacterium
D_0__Bacteria;D_1__Proteobacteria;D_2__Alphaproteobacteria;D_3__Rhizobiales;D_4__Aurantimonadaceae;D_5__Aureimonas
D_0__Bacteria;D_1__Proteobacteria;D_2__Alphaproteobacteria;D_3__Rhizobiales;D_4__Aurantimonadaceae;Other
D_0__Bacteria;D_1__Proteobacteria;D_2__Alphaproteobacteria;D_3__Rhizobiales;D_4__Beijerinckiaceae;D_5__uncultured
D_0__Bacteria;D_1__Proteobacteria;D_2__Alphaproteobacteria;D_3__Rhizobiales;D_4__Beijerinckiaceae;Other
D_0__Bacteria;D_1__Proteobacteria;D_2__Alphaproteobacteria;D_3__Rhizobiales;D_4__Bradyrhizobiaceae;D_5__Afipia
D_0__Bacteria;D_1__Proteobacteria;D_2__Alphaproteobacteria;D_3__Rhizobiales;D_4__Bradyrhizobiaceae;D_5__Bosea
D_0__Bacteria;D_1__Proteobacteria;D_2__Alphaproteobacteria;D_3__Rhizobiales;D_4__Bradyrhizobiaceae;D_5__Bradyrhizobium
D_0__Bacteria;D_1__Proteobacteria;D_2__Alphaproteobacteria;D_3__Rhizobiales;D_4__Bradyrhizobiaceae;D_5__Nitrobacter
D_0__Bacteria;D_1__Proteobacteria;D_2__Alphaproteobacteria;D_3__Rhizobiales;D_4__Bradyrhizobiaceae;D_5__uncultured
D_0__Bacteria;D_1__Proteobacteria;D_2__Alphaproteobacteria;D_3__Rhizobiales;D_4__Bradyrhizobiaceae;Other
D_0__Bacteria;D_1__Proteobacteria;D_2__Alphaproteobacteria;D_3__Rhizobiales;D_4__Brucellaceae;Other
D_0__Bacteria;D_1__Proteobacteria;D_2__Alphaproteobacteria;D_3__Rhizobiales;D_4__C2U;D_5__uncultured bacterium
D_0__Bacteria;D_1__Proteobacteria;D_2__Alphaproteobacteria;D_3__Rhizobiales;D_4__DUNssu044;Ambiguous_taxa
D_0__Bacteria;D_1__Proteobacteria;D_2__Alphaproteobacteria;D_3__Rhizobiales;D_4__DUNssu044;D_5__uncultured bacterium
D_0__Bacteria;D_1__Proteobacteria;D_2__Alphaproteobacteria;D_3__Rhizobiales;D_4__DUNssu044;Other
D_0__Bacteria;D_1__Proteobacteria;D_2__Alphaproteobacteria;D_3__Rhizobiales;D_4__FukuN57;D_5__uncultured bacterium
D_0__Bacteria;D_1__Proteobacteria;D_2__Alphaproteobacteria;D_3__Rhizobiales;D_4__Hyphomicrobiaceae;Ambiguous_taxa
D_0__Bacteria;D_1__Proteobacteria;D_2__Alphaproteobacteria;D_3__Rhizobiales;D_4__Hyphomicrobiaceae;D_5__Devosia
D_0__Bacteria;D_1__Proteobacteria;D_2__Alphaproteobacteria;D_3__Rhizobiales;D_4__Hyphomicrobiaceae;D_5__Hyphomicrobium
D_0__Bacteria;D_1__Proteobacteria;D_2__Alphaproteobacteria;D_3__Rhizobiales;D_4__Hyphomicrobiaceae;D_5__Pedomicrobium
D_0__Bacteria;D_1__Proteobacteria;D_2__Alphaproteobacteria;D_3__Rhizobiales;D_4__Hyphomicrobiaceae;D_5__Rhodomicrobium
D_0__Bacteria;D_1__Proteobacteria;D_2__Alphaproteobacteria;D_3__Rhizobiales;D_4__Hyphomicrobiaceae;D_5__Rhodoplanes
D_0__Bacteria;D_1__Proteobacteria;D_2__Alphaproteobacteria;D_3__Rhizobiales;D_4__Hyphomicrobiaceae;D_5__uncultured
D_0__Bacteria;D_1__Proteobacteria;D_2__Alphaproteobacteria;D_3__Rhizobiales;D_4__Hyphomicrobiaceae;Other
D_0__Bacteria;D_1__Proteobacteria;D_2__Alphaproteobacteria;D_3__Rhizobiales;D_4__JG34-KF-361;Ambiguous_taxa
D_0__Bacteria;D_1__Proteobacteria;D_2__Alphaproteobacteria;D_3__Rhizobiales;D_4__JG34-KF-361;D_5__uncultured bacterium
D_0__Bacteria;D_1__Proteobacteria;D_2__Alphaproteobacteria;D_3__Rhizobiales;D_4__JG34-KF-361;D_5__uncultured forest soil bacterium
D_0__Bacteria;D_1__Proteobacteria;D_2__Alphaproteobacteria;D_3__Rhizobiales;D_4__JG34-KF-361;Other
D_0__Bacteria;D_1__Proteobacteria;D_2__Alphaproteobacteria;D_3__Rhizobiales;D_4__KF-JG30-B3;D_5__uncultured bacterium
D_0__Bacteria;D_1__Proteobacteria;D_2__Alphaproteobacteria;D_3__Rhizobiales;D_4__MNG7;Other
D_0__Bacteria;D_1__Proteobacteria;D_2__Alphaproteobacteria;D_3__Rhizobiales;D_4__Methylobacteriaceae;D_5__Methylobacterium
D_0__Bacteria;D_1__Proteobacteria;D_2__Alphaproteobacteria;D_3__Rhizobiales;D_4__Methylobacteriaceae;D_5__Microvirga
D_0__Bacteria;D_1__Proteobacteria;D_2__Alphaproteobacteria;D_3__Rhizobiales;D_4__Methylobacteriaceae;D_5__Psychroglaciecola
D_0__Bacteria;D_1__Proteobacteria;D_2__Alphaproteobacteria;D_3__Rhizobiales;D_4__Methylobacteriaceae;D_5__uncultured
D_0__Bacteria;D_1__Proteobacteria;D_2__Alphaproteobacteria;D_3__Rhizobiales;D_4__Methylobacteriaceae;Other
D_0__Bacteria;D_1__Proteobacteria;D_2__Alphaproteobacteria;D_3__Rhizobiales;D_4__Methylocystaceae;D_5__Pleomorphomonas
D_0__Bacteria;D_1__Proteobacteria;D_2__Alphaproteobacteria;D_3__Rhizobiales;D_4__Methylocystaceae;Other
D_0__Bacteria;D_1__Proteobacteria;D_2__Alphaproteobacteria;D_3__Rhizobiales;D_4__Phyllobacteriaceae;D_5__Aliihoeflea
D_0__Bacteria;D_1__Proteobacteria;D_2__Alphaproteobacteria;D_3__Rhizobiales;D_4__Phyllobacteriaceae;D_5__Cohaesibacter
D_0__Bacteria;D_1__Proteobacteria;D_2__Alphaproteobacteria;D_3__Rhizobiales;D_4__Phyllobacteriaceae;D_5__Mesorhizobium
D_0__Bacteria;D_1__Proteobacteria;D_2__Alphaproteobacteria;D_3__Rhizobiales;D_4__Phyllobacteriaceae;D_5__Phyllobacterium
D_0__Bacteria;D_1__Proteobacteria;D_2__Alphaproteobacteria;D_3__Rhizobiales;D_4__Phyllobacteriaceae;Other
D_0__Bacteria;D_1__Proteobacteria;D_2__Alphaproteobacteria;D_3__Rhizobiales;D_4__Rhizobiaceae;D_5__Ensifer
D_0__Bacteria;D_1__Proteobacteria;D_2__Alphaproteobacteria;D_3__Rhizobiales;D_4__Rhizobiaceae;D_5__Kaistia
D_0__Bacteria;D_1__Proteobacteria;D_2__Alphaproteobacteria;D_3__Rhizobiales;D_4__Rhizobiaceae;D_5__Rhizobium
D_0__Bacteria;D_1__Proteobacteria;D_2__Alphaproteobacteria;D_3__Rhizobiales;D_4__Rhizobiaceae;D_5__Shinella
D_0__Bacteria;D_1__Proteobacteria;D_2__Alphaproteobacteria;D_3__Rhizobiales;D_4__Rhizobiaceae;Other
D_0__Bacteria;D_1__Proteobacteria;D_2__Alphaproteobacteria;D_3__Rhizobiales;D_4__Rhizobiales Incertae Sedis;D_5__Agaricicola
D_0__Bacteria;D_1__Proteobacteria;D_2__Alphaproteobacteria;D_3__Rhizobiales;D_4__Rhizobiales Incertae Sedis;D_5__Bauldia
D_0__Bacteria;D_1__Proteobacteria;D_2__Alphaproteobacteria;D_3__Rhizobiales;D_4__Rhizobiales Incertae Sedis;D_5__Phreatobacter
D_0__Bacteria;D_1__Proteobacteria;D_2__Alphaproteobacteria;D_3__Rhizobiales;D_4__Rhizobiales Incertae Sedis;D_5__Rhizomicrobium
D_0__Bacteria;D_1__Proteobacteria;D_2__Alphaproteobacteria;D_3__Rhizobiales;D_4__Rhodobiaceae;Ambiguous_taxa
D_0__Bacteria;D_1__Proteobacteria;D_2__Alphaproteobacteria;D_3__Rhizobiales;D_4__Rhodobiaceae;D_5__Anderseniella
D_0__Bacteria;D_1__Proteobacteria;D_2__Alphaproteobacteria;D_3__Rhizobiales;D_4__Rhodobiaceae;D_5__Rhodobium
D_0__Bacteria;D_1__Proteobacteria;D_2__Alphaproteobacteria;D_3__Rhizobiales;D_4__Rhodobiaceae;D_5__uncultured
D_0__Bacteria;D_1__Proteobacteria;D_2__Alphaproteobacteria;D_3__Rhizobiales;D_4__Rhodobiaceae;Other
D_0__Bacteria;D_1__Proteobacteria;D_2__Alphaproteobacteria;D_3__Rhizobiales;D_4__Roseiarcaceae;D_5__Roseiarcus
D_0__Bacteria;D_1__Proteobacteria;D_2__Alphaproteobacteria;D_3__Rhizobiales;D_4__Xanthobacteraceae;D_5__Pseudolabrys
D_0__Bacteria;D_1__Proteobacteria;D_2__Alphaproteobacteria;D_3__Rhizobiales;D_4__Xanthobacteraceae;D_5__Variibacter
D_0__Bacteria;D_1__Proteobacteria;D_2__Alphaproteobacteria;D_3__Rhizobiales;D_4__Xanthobacteraceae;D_5__uncultured
D_0__Bacteria;D_1__Proteobacteria;D_2__Alphaproteobacteria;D_3__Rhizobiales;D_4__Xanthobacteraceae;Other
D_0__Bacteria;D_1__Proteobacteria;D_2__Alphaproteobacteria;D_3__Rhizobiales;D_4__alphaI cluster;Ambiguous_taxa
D_0__Bacteria;D_1__Proteobacteria;D_2__Alphaproteobacteria;D_3__Rhizobiales;D_4__uncultured;Ambiguous_taxa
D_0__Bacteria;D_1__Proteobacteria;D_2__Alphaproteobacteria;D_3__Rhizobiales;D_4__uncultured;D_5__uncultured alpha proteobacterium
D_0__Bacteria;D_1__Proteobacteria;D_2__Alphaproteobacteria;D_3__Rhizobiales;D_4__uncultured;D_5__uncultured bacterium
D_0__Bacteria;D_1__Proteobacteria;D_2__Alphaproteobacteria;D_3__Rhizobiales;D_4__uncultured;Other
D_0__Bacteria;D_1__Proteobacteria;D_2__Alphaproteobacteria;D_3__Rhizobiales;Other;Other
D_0__Bacteria;D_1__Proteobacteria;D_2__Alphaproteobacteria;D_3__Rhodobacterales;D_4__Rhodobacteraceae;D_5__Defluviimonas
D_0__Bacteria;D_1__Proteobacteria;D_2__Alphaproteobacteria;D_3__Rhodobacterales;D_4__Rhodobacteraceae;D_5__Labrenzia
D_0__Bacteria;D_1__Proteobacteria;D_2__Alphaproteobacteria;D_3__Rhodobacterales;D_4__Rhodobacteraceae;D_5__Paracoccus
D_0__Bacteria;D_1__Proteobacteria;D_2__Alphaproteobacteria;D_3__Rhodobacterales;D_4__Rhodobacteraceae;D_5__Rhodobacter
D_0__Bacteria;D_1__Proteobacteria;D_2__Alphaproteobacteria;D_3__Rhodobacterales;D_4__Rhodobacteraceae;D_5__Rubellimicrobium
D_0__Bacteria;D_1__Proteobacteria;D_2__Alphaproteobacteria;D_3__Rhodobacterales;D_4__Rhodobacteraceae;D_5__Thioclava
D_0__Bacteria;D_1__Proteobacteria;D_2__Alphaproteobacteria;D_3__Rhodobacterales;D_4__Rhodobacteraceae;D_5__Tropicimonas
D_0__Bacteria;D_1__Proteobacteria;D_2__Alphaproteobacteria;D_3__Rhodobacterales;D_4__Rhodobacteraceae;D_5__uncultured
D_0__Bacteria;D_1__Proteobacteria;D_2__Alphaproteobacteria;D_3__Rhodobacterales;D_4__Rhodobacteraceae;Other
D_0__Bacteria;D_1__Proteobacteria;D_2__Alphaproteobacteria;D_3__Rhodospirillales;D_4__AT-s3-44;Other
D_0__Bacteria;D_1__Proteobacteria;D_2__Alphaproteobacteria;D_3__Rhodospirillales;D_4__Acetobacteraceae;D_5__Acidiphilium
D_0__Bacteria;D_1__Proteobacteria;D_2__Alphaproteobacteria;D_3__Rhodospirillales;D_4__Acetobacteraceae;D_5__Craurococcus
D_0__Bacteria;D_1__Proteobacteria;D_2__Alphaproteobacteria;D_3__Rhodospirillales;D_4__Acetobacteraceae;D_5__Endobacter
D_0__Bacteria;D_1__Proteobacteria;D_2__Alphaproteobacteria;D_3__Rhodospirillales;D_4__Acetobacteraceae;D_5__Roseomonas
D_0__Bacteria;D_1__Proteobacteria;D_2__Alphaproteobacteria;D_3__Rhodospirillales;D_4__Acetobacteraceae;D_5__Rubritepida
D_0__Bacteria;D_1__Proteobacteria;D_2__Alphaproteobacteria;D_3__Rhodospirillales;D_4__Acetobacteraceae;D_5__uncultured
D_0__Bacteria;D_1__Proteobacteria;D_2__Alphaproteobacteria;D_3__Rhodospirillales;D_4__Acetobacteraceae;Other
D_0__Bacteria;D_1__Proteobacteria;D_2__Alphaproteobacteria;D_3__Rhodospirillales;D_4__DA111;D_5__uncultured alpha proteobacterium
D_0__Bacteria;D_1__Proteobacteria;D_2__Alphaproteobacteria;D_3__Rhodospirillales;D_4__DA111;D_5__uncultured bacterium
D_0__Bacteria;D_1__Proteobacteria;D_2__Alphaproteobacteria;D_3__Rhodospirillales;D_4__DA111;Other
D_0__Bacteria;D_1__Proteobacteria;D_2__Alphaproteobacteria;D_3__Rhodospirillales;D_4__KCM-B-15;D_5__uncultured Rhodospirillaceae bacterium
D_0__Bacteria;D_1__Proteobacteria;D_2__Alphaproteobacteria;D_3__Rhodospirillales;D_4__MND8;Other
D_0__Bacteria;D_1__Proteobacteria;D_2__Alphaproteobacteria;D_3__Rhodospirillales;D_4__MSB-1E8;D_5__uncultured bacterium
D_0__Bacteria;D_1__Proteobacteria;D_2__Alphaproteobacteria;D_3__Rhodospirillales;D_4__MSB-1E8;Other
D_0__Bacteria;D_1__Proteobacteria;D_2__Alphaproteobacteria;D_3__Rhodospirillales;D_4__Rhodospirillaceae;D_5__Defluviicoccus
D_0__Bacteria;D_1__Proteobacteria;D_2__Alphaproteobacteria;D_3__Rhodospirillales;D_4__Rhodospirillaceae;D_5__Magnetospira
D_0__Bacteria;D_1__Proteobacteria;D_2__Alphaproteobacteria;D_3__Rhodospirillales;D_4__Rhodospirillaceae;D_5__Nitrospirillum
D_0__Bacteria;D_1__Proteobacteria;D_2__Alphaproteobacteria;D_3__Rhodospirillales;D_4__Rhodospirillaceae;D_5__Niveispirillum
D_0__Bacteria;D_1__Proteobacteria;D_2__Alphaproteobacteria;D_3__Rhodospirillales;D_4__Rhodospirillaceae;D_5__Pelagibius
D_0__Bacteria;D_1__Proteobacteria;D_2__Alphaproteobacteria;D_3__Rhodospirillales;D_4__Rhodospirillaceae;D_5__Skermanella
D_0__Bacteria;D_1__Proteobacteria;D_2__Alphaproteobacteria;D_3__Rhodospirillales;D_4__Rhodospirillaceae;D_5__Thalassobaculum
D_0__Bacteria;D_1__Proteobacteria;D_2__Alphaproteobacteria;D_3__Rhodospirillales;D_4__Rhodospirillaceae;D_5__uncultured
D_0__Bacteria;D_1__Proteobacteria;D_2__Alphaproteobacteria;D_3__Rhodospirillales;D_4__Rhodospirillaceae;Other
D_0__Bacteria;D_1__Proteobacteria;D_2__Alphaproteobacteria;D_3__Rhodospirillales;D_4__Rhodospirillales Incertae Sedis;D_5__Candidatus Alysiosphaera
D_0__Bacteria;D_1__Proteobacteria;D_2__Alphaproteobacteria;D_3__Rhodospirillales;D_4__Rhodospirillales Incertae Sedis;D_5__Reyranella
D_0__Bacteria;D_1__Proteobacteria;D_2__Alphaproteobacteria;D_3__Rhodospirillales;Other;Other
D_0__Bacteria;D_1__Proteobacteria;D_2__Alphaproteobacteria;D_3__Rickettsiales;D_4__EF100-94H03;D_5__uncultured Parvibaculum sp.
D_0__Bacteria;D_1__Proteobacteria;D_2__Alphaproteobacteria;D_3__Rickettsiales;D_4__Mitochondria;Ambiguous_taxa
D_0__Bacteria;D_1__Proteobacteria;D_2__Alphaproteobacteria;D_3__Rickettsiales;D_4__Mitochondria;Other
D_0__Bacteria;D_1__Proteobacteria;D_2__Alphaproteobacteria;D_3__Rickettsiales;D_4__Rickettsiaceae;D_5__Orientia
D_0__Bacteria;D_1__Proteobacteria;D_2__Alphaproteobacteria;D_3__Rickettsiales;D_4__SM2D12;D_5__uncultured bacterium
D_0__Bacteria;D_1__Proteobacteria;D_2__Alphaproteobacteria;D_3__Rickettsiales;D_4__TK34;Ambiguous_taxa
D_0__Bacteria;D_1__Proteobacteria;D_2__Alphaproteobacteria;D_3__Sphingomonadales;D_4__7B-8;D_5__uncultured bacterium
D_0__Bacteria;D_1__Proteobacteria;D_2__Alphaproteobacteria;D_3__Sphingomonadales;D_4__Ellin6055;D_5__uncultured bacterium
D_0__Bacteria;D_1__Proteobacteria;D_2__Alphaproteobacteria;D_3__Sphingomonadales;D_4__Ellin6055;Other
D_0__Bacteria;D_1__Proteobacteria;D_2__Alphaproteobacteria;D_3__Sphingomonadales;D_4__Erythrobacteraceae;D_5__Altererythrobacter
D_0__Bacteria;D_1__Proteobacteria;D_2__Alphaproteobacteria;D_3__Sphingomonadales;D_4__Erythrobacteraceae;Other
D_0__Bacteria;D_1__Proteobacteria;D_2__Alphaproteobacteria;D_3__Sphingomonadales;D_4__Sphingomonadaceae;D_5__Novosphingobium
D_0__Bacteria;D_1__Proteobacteria;D_2__Alphaproteobacteria;D_3__Sphingomonadales;D_4__Sphingomonadaceae;D_5__Rhizorhapis
D_0__Bacteria;D_1__Proteobacteria;D_2__Alphaproteobacteria;D_3__Sphingomonadales;D_4__Sphingomonadaceae;D_5__Sphingomonas
D_0__Bacteria;D_1__Proteobacteria;D_2__Alphaproteobacteria;D_3__Sphingomonadales;D_4__Sphingomonadaceae;D_5__Sphingopyxis
D_0__Bacteria;D_1__Proteobacteria;D_2__Alphaproteobacteria;D_3__Sphingomonadales;D_4__Sphingomonadaceae;D_5__Zymomonas
D_0__Bacteria;D_1__Proteobacteria;D_2__Alphaproteobacteria;D_3__Sphingomonadales;D_4__Sphingomonadaceae;Other
D_0__Bacteria;D_1__Proteobacteria;D_2__Alphaproteobacteria;D_3__Sphingomonadales;Other;Other
D_0__Bacteria;D_1__Proteobacteria;D_2__Alphaproteobacteria;Other;Other;Other
D_0__Bacteria;D_1__Proteobacteria;D_2__Betaproteobacteria;D_3__Burkholderiales;D_4__Alcaligenaceae;D_5__Parasutterella
D_0__Bacteria;D_1__Proteobacteria;D_2__Betaproteobacteria;D_3__Burkholderiales;D_4__Burkholderiaceae;D_5__Burkholderia-Paraburkholderia
D_0__Bacteria;D_1__Proteobacteria;D_2__Betaproteobacteria;D_3__Burkholderiales;D_4__Burkholderiaceae;D_5__Cupriavidus
D_0__Bacteria;D_1__Proteobacteria;D_2__Betaproteobacteria;D_3__Burkholderiales;D_4__Burkholderiaceae;D_5__Lautropia
D_0__Bacteria;D_1__Proteobacteria;D_2__Betaproteobacteria;D_3__Burkholderiales;D_4__Burkholderiaceae;D_5__Limnobacter
D_0__Bacteria;D_1__Proteobacteria;D_2__Betaproteobacteria;D_3__Burkholderiales;D_4__Comamonadaceae;Ambiguous_taxa
D_0__Bacteria;D_1__Proteobacteria;D_2__Betaproteobacteria;D_3__Burkholderiales;D_4__Comamonadaceae;D_5__Acidovorax
D_0__Bacteria;D_1__Proteobacteria;D_2__Betaproteobacteria;D_3__Burkholderiales;D_4__Comamonadaceae;D_5__Aquabacterium
D_0__Bacteria;D_1__Proteobacteria;D_2__Betaproteobacteria;D_3__Burkholderiales;D_4__Comamonadaceae;D_5__Comamonas
D_0__Bacteria;D_1__Proteobacteria;D_2__Betaproteobacteria;D_3__Burkholderiales;D_4__Comamonadaceae;D_5__Delftia
D_0__Bacteria;D_1__Proteobacteria;D_2__Betaproteobacteria;D_3__Burkholderiales;D_4__Comamonadaceae;D_5__Polaromonas
D_0__Bacteria;D_1__Proteobacteria;D_2__Betaproteobacteria;D_3__Burkholderiales;D_4__Comamonadaceae;D_5__Tepidimonas
D_0__Bacteria;D_1__Proteobacteria;D_2__Betaproteobacteria;D_3__Burkholderiales;D_4__Comamonadaceae;D_5__uncultured
D_0__Bacteria;D_1__Proteobacteria;D_2__Betaproteobacteria;D_3__Burkholderiales;D_4__Comamonadaceae;Other
D_0__Bacteria;D_1__Proteobacteria;D_2__Betaproteobacteria;D_3__Burkholderiales;D_4__Oxalobacteraceae;D_5__Herbaspirillum
D_0__Bacteria;D_1__Proteobacteria;D_2__Betaproteobacteria;D_3__Burkholderiales;D_4__Oxalobacteraceae;D_5__Massilia
D_0__Bacteria;D_1__Proteobacteria;D_2__Betaproteobacteria;D_3__Burkholderiales;D_4__Oxalobacteraceae;D_5__Noviherbaspirillum
D_0__Bacteria;D_1__Proteobacteria;D_2__Betaproteobacteria;D_3__Burkholderiales;D_4__Oxalobacteraceae;D_5__Undibacterium
D_0__Bacteria;D_1__Proteobacteria;D_2__Betaproteobacteria;D_3__Burkholderiales;D_4__Oxalobacteraceae;D_5__uncultured
D_0__Bacteria;D_1__Proteobacteria;D_2__Betaproteobacteria;D_3__Burkholderiales;D_4__Oxalobacteraceae;Other
D_0__Bacteria;D_1__Proteobacteria;D_2__Betaproteobacteria;D_3__Hydrogenophilales;D_4__Hydrogenophilaceae;D_5__Ferritrophicum
D_0__Bacteria;D_1__Proteobacteria;D_2__Betaproteobacteria;D_3__Hydrogenophilales;D_4__Hydrogenophilaceae;D_5__Hydrogenophilus
D_0__Bacteria;D_1__Proteobacteria;D_2__Betaproteobacteria;D_3__Hydrogenophilales;D_4__Hydrogenophilaceae;D_5__Thiobacillus
D_0__Bacteria;D_1__Proteobacteria;D_2__Betaproteobacteria;D_3__Methylophilales;D_4__Methylophilaceae;D_5__Methylotenera
D_0__Bacteria;D_1__Proteobacteria;D_2__Betaproteobacteria;D_3__Methylophilales;D_4__Methylophilaceae;D_5__OM43 clade
D_0__Bacteria;D_1__Proteobacteria;D_2__Betaproteobacteria;D_3__Methylophilales;D_4__Methylophilaceae;D_5__uncultured
D_0__Bacteria;D_1__Proteobacteria;D_2__Betaproteobacteria;D_3__Neisseriales;D_4__Neisseriaceae;D_5__Eikenella
D_0__Bacteria;D_1__Proteobacteria;D_2__Betaproteobacteria;D_3__Neisseriales;D_4__Neisseriaceae;D_5__Neisseria
D_0__Bacteria;D_1__Proteobacteria;D_2__Betaproteobacteria;D_3__Neisseriales;D_4__Neisseriaceae;D_5__uncultured
D_0__Bacteria;D_1__Proteobacteria;D_2__Betaproteobacteria;D_3__Neisseriales;D_4__Neisseriaceae;Other
D_0__Bacteria;D_1__Proteobacteria;D_2__Betaproteobacteria;D_3__Nitrosomonadales;D_4__Gallionellaceae;D_5__Candidatus Nitrotoga
D_0__Bacteria;D_1__Proteobacteria;D_2__Betaproteobacteria;D_3__Nitrosomonadales;D_4__Gallionellaceae;D_5__Gallionella
D_0__Bacteria;D_1__Proteobacteria;D_2__Betaproteobacteria;D_3__Nitrosomonadales;D_4__Gallionellaceae;D_5__Sideroxydans
D_0__Bacteria;D_1__Proteobacteria;D_2__Betaproteobacteria;D_3__Nitrosomonadales;D_4__Gallionellaceae;Other
D_0__Bacteria;D_1__Proteobacteria;D_2__Betaproteobacteria;D_3__Nitrosomonadales;D_4__Nitrosomonadaceae;D_5__Nitrosospira
D_0__Bacteria;D_1__Proteobacteria;D_2__Betaproteobacteria;D_3__Nitrosomonadales;D_4__Nitrosomonadaceae;D_5__uncultured
D_0__Bacteria;D_1__Proteobacteria;D_2__Betaproteobacteria;D_3__Nitrosomonadales;D_4__Nitrosomonadaceae;Other
D_0__Bacteria;D_1__Proteobacteria;D_2__Betaproteobacteria;D_3__Rhodocyclales;D_4__Rhodocyclaceae;D_5__Thauera
D_0__Bacteria;D_1__Proteobacteria;D_2__Betaproteobacteria;D_3__Rhodocyclales;D_4__Rhodocyclaceae;D_5__Zoogloea
D_0__Bacteria;D_1__Proteobacteria;D_2__Betaproteobacteria;D_3__Rhodocyclales;D_4__Rhodocyclaceae;Other
D_0__Bacteria;D_1__Proteobacteria;D_2__Betaproteobacteria;D_3__SC-I-84;D_4__uncultured bacterium;D_5__uncultured bacterium
D_0__Bacteria;D_1__Proteobacteria;D_2__Betaproteobacteria;D_3__TRA3-20;Ambiguous_taxa;Ambiguous_taxa
D_0__Bacteria;D_1__Proteobacteria;D_2__Betaproteobacteria;D_3__TRA3-20;D_4__uncultured bacterium;D_5__uncultured bacterium
D_0__Bacteria;D_1__Proteobacteria;D_2__Betaproteobacteria;D_3__TRA3-20;D_4__uncultured gamma proteobacterium;D_5__uncultured gamma proteobacterium
D_0__Bacteria;D_1__Proteobacteria;D_2__Betaproteobacteria;D_3__TRA3-20;Other;Other
D_0__Bacteria;D_1__Proteobacteria;D_2__Betaproteobacteria;Other;Other;Other
D_0__Bacteria;D_1__Proteobacteria;D_2__Deltaproteobacteria;D_3__Bdellovibrionales;D_4__Bacteriovoracaceae;D_5__Bacteriovorax
D_0__Bacteria;D_1__Proteobacteria;D_2__Deltaproteobacteria;D_3__Bdellovibrionales;D_4__Bacteriovoracaceae;D_5__Peredibacter
D_0__Bacteria;D_1__Proteobacteria;D_2__Deltaproteobacteria;D_3__Bdellovibrionales;D_4__Bdellovibrionaceae;D_5__Bdellovibrio
D_0__Bacteria;D_1__Proteobacteria;D_2__Deltaproteobacteria;D_3__Desulfurellales;D_4__Desulfurellaceae;D_5__G55
D_0__Bacteria;D_1__Proteobacteria;D_2__Deltaproteobacteria;D_3__Desulfurellales;D_4__Desulfurellaceae;D_5__H16
D_0__Bacteria;D_1__Proteobacteria;D_2__Deltaproteobacteria;D_3__Myxococcales;Ambiguous_taxa;Other
D_0__Bacteria;D_1__Proteobacteria;D_2__Deltaproteobacteria;D_3__Myxococcales;D_4__Archangiaceae;D_5__Anaeromyxobacter
D_0__Bacteria;D_1__Proteobacteria;D_2__Deltaproteobacteria;D_3__Myxococcales;D_4__Blrii41;Ambiguous_taxa
D_0__Bacteria;D_1__Proteobacteria;D_2__Deltaproteobacteria;D_3__Myxococcales;D_4__Blrii41;D_5__uncultured bacterium
D_0__Bacteria;D_1__Proteobacteria;D_2__Deltaproteobacteria;D_3__Myxococcales;D_4__Haliangiaceae;D_5__Haliangium
D_0__Bacteria;D_1__Proteobacteria;D_2__Deltaproteobacteria;D_3__Myxococcales;D_4__Nannocystaceae;D_5__Nannocystis
D_0__Bacteria;D_1__Proteobacteria;D_2__Deltaproteobacteria;D_3__Myxococcales;D_4__Nannocystaceae;D_5__uncultured
D_0__Bacteria;D_1__Proteobacteria;D_2__Deltaproteobacteria;D_3__Myxococcales;D_4__P3OB-42;D_5__uncultured bacterium
D_0__Bacteria;D_1__Proteobacteria;D_2__Deltaproteobacteria;D_3__Myxococcales;D_4__P3OB-42;Other
D_0__Bacteria;D_1__Proteobacteria;D_2__Deltaproteobacteria;D_3__Myxococcales;D_4__Phaselicystidaceae;D_5__Phaselicystis
D_0__Bacteria;D_1__Proteobacteria;D_2__Deltaproteobacteria;D_3__Myxococcales;D_4__Polyangiaceae;D_5__Sorangium
D_0__Bacteria;D_1__Proteobacteria;D_2__Deltaproteobacteria;D_3__Myxococcales;D_4__Polyangiaceae;D_5__uncultured
D_0__Bacteria;D_1__Proteobacteria;D_2__Deltaproteobacteria;D_3__Myxococcales;D_4__Polyangiaceae;Other
D_0__Bacteria;D_1__Proteobacteria;D_2__Deltaproteobacteria;D_3__Myxococcales;D_4__Sandaracinaceae;D_5__Sandaracinus
D_0__Bacteria;D_1__Proteobacteria;D_2__Deltaproteobacteria;D_3__Myxococcales;D_4__Sandaracinaceae;D_5__uncultured
D_0__Bacteria;D_1__Proteobacteria;D_2__Deltaproteobacteria;D_3__Myxococcales;D_4__VHS-B3-70;D_5__uncultured bacterium
D_0__Bacteria;D_1__Proteobacteria;D_2__Deltaproteobacteria;D_3__Myxococcales;D_4__mle1-27;D_5__uncultured bacterium
D_0__Bacteria;D_1__Proteobacteria;D_2__Deltaproteobacteria;D_3__Myxococcales;D_4__mle1-27;Other
D_0__Bacteria;D_1__Proteobacteria;D_2__Deltaproteobacteria;D_3__Myxococcales;D_4__uncultured;D_5__uncultured bacterium
D_0__Bacteria;D_1__Proteobacteria;D_2__Deltaproteobacteria;D_3__Myxococcales;D_4__uncultured bacterium;D_5__uncultured bacterium
D_0__Bacteria;D_1__Proteobacteria;D_2__Deltaproteobacteria;D_3__Myxococcales;Other;Other
D_0__Bacteria;D_1__Proteobacteria;D_2__Deltaproteobacteria;D_3__Oligoflexales;D_4__0319-6G20;D_5__uncultured bacterium
D_0__Bacteria;D_1__Proteobacteria;D_2__Deltaproteobacteria;D_3__Oligoflexales;D_4__0319-6G20;Other
D_0__Bacteria;D_1__Proteobacteria;D_2__Deltaproteobacteria;D_3__Oligoflexales;D_4__Oligoflexaceae;D_5__uncultured bacterium
D_0__Bacteria;D_1__Proteobacteria;D_2__Deltaproteobacteria;D_3__SAR324 clade(Marine group B);D_4__uncultured bacterium;D_5__uncultured bacterium
D_0__Bacteria;D_1__Proteobacteria;D_2__Deltaproteobacteria;D_3__SAR324 clade(Marine group B);D_4__uncultured delta proteobacterium;D_5__uncultured delta proteobacterium
D_0__Bacteria;D_1__Proteobacteria;D_2__Epsilonproteobacteria;D_3__Campylobacterales;D_4__Campylobacteraceae;D_5__Campylobacter
D_0__Bacteria;D_1__Proteobacteria;D_2__Gammaproteobacteria;D_3__Alteromonadales;D_4__Alteromonadaceae;D_5__Alishewanella
D_0__Bacteria;D_1__Proteobacteria;D_2__Gammaproteobacteria;D_3__Alteromonadales;D_4__Alteromonadaceae;D_5__Marinobacter
D_0__Bacteria;D_1__Proteobacteria;D_2__Gammaproteobacteria;D_3__Alteromonadales;D_4__Psychromonadaceae;D_5__Psychromonas
D_0__Bacteria;D_1__Proteobacteria;D_2__Gammaproteobacteria;D_3__Cardiobacteriales;D_4__Cardiobacteriaceae;D_5__Cardiobacterium
D_0__Bacteria;D_1__Proteobacteria;D_2__Gammaproteobacteria;D_3__Cellvibrionales;D_4__Cellvibrionaceae;D_5__Cellvibrio
D_0__Bacteria;D_1__Proteobacteria;D_2__Gammaproteobacteria;D_3__Chromatiales;D_4__Chromatiaceae;D_5__Rheinheimera
D_0__Bacteria;D_1__Proteobacteria;D_2__Gammaproteobacteria;D_3__Chromatiales;D_4__Chromatiaceae;D_5__uncultured
D_0__Bacteria;D_1__Proteobacteria;D_2__Gammaproteobacteria;D_3__Chromatiales;D_4__Ectothiorhodospiraceae;D_5__Thioalkalispira
D_0__Bacteria;D_1__Proteobacteria;D_2__Gammaproteobacteria;D_3__Chromatiales;D_4__Ectothiorhodospiraceae;D_5__Thiogranum
D_0__Bacteria;D_1__Proteobacteria;D_2__Gammaproteobacteria;D_3__Chromatiales;D_4__Ectothiorhodospiraceae;Other
D_0__Bacteria;D_1__Proteobacteria;D_2__Gammaproteobacteria;D_3__Chromatiales;Other;Other
D_0__Bacteria;D_1__Proteobacteria;D_2__Gammaproteobacteria;D_3__Enterobacteriales;D_4__Enterobacteriaceae;Ambiguous_taxa
D_0__Bacteria;D_1__Proteobacteria;D_2__Gammaproteobacteria;D_3__Enterobacteriales;D_4__Enterobacteriaceae;D_5__Candidatus Blochmannia
D_0__Bacteria;D_1__Proteobacteria;D_2__Gammaproteobacteria;D_3__Enterobacteriales;D_4__Enterobacteriaceae;D_5__Escherichia-Shigella
D_0__Bacteria;D_1__Proteobacteria;D_2__Gammaproteobacteria;D_3__Gammaproteobacteria Incertae Sedis;D_4__Unknown Family;D_5__uncultured gamma proteobacterium
D_0__Bacteria;D_1__Proteobacteria;D_2__Gammaproteobacteria;D_3__HTA4;D_4__uncultured bacterium;D_5__uncultured bacterium
D_0__Bacteria;D_1__Proteobacteria;D_2__Gammaproteobacteria;D_3__KI89A clade;D_4__uncultured bacterium;D_5__uncultured bacterium
D_0__Bacteria;D_1__Proteobacteria;D_2__Gammaproteobacteria;D_3__Legionellales;D_4__Coxiellaceae;D_5__Aquicella
D_0__Bacteria;D_1__Proteobacteria;D_2__Gammaproteobacteria;D_3__Legionellales;D_4__Coxiellaceae;D_5__Coxiella
D_0__Bacteria;D_1__Proteobacteria;D_2__Gammaproteobacteria;D_3__Legionellales;D_4__Coxiellaceae;D_5__Diplorickettsia
D_0__Bacteria;D_1__Proteobacteria;D_2__Gammaproteobacteria;D_3__Legionellales;D_4__Coxiellaceae;D_5__uncultured
D_0__Bacteria;D_1__Proteobacteria;D_2__Gammaproteobacteria;D_3__Legionellales;D_4__Legionellaceae;D_5__Legionella
D_0__Bacteria;D_1__Proteobacteria;D_2__Gammaproteobacteria;D_3__Oceanospirillales;D_4__Halomonadaceae;D_5__Halomonas
D_0__Bacteria;D_1__Proteobacteria;D_2__Gammaproteobacteria;D_3__Oceanospirillales;D_4__Oceanospirillaceae;D_5__Pseudohongiella
D_0__Bacteria;D_1__Proteobacteria;D_2__Gammaproteobacteria;D_3__PYR10d3;Other;Other
D_0__Bacteria;D_1__Proteobacteria;D_2__Gammaproteobacteria;D_3__Pasteurellales;D_4__Pasteurellaceae;D_5__Aggregatibacter
D_0__Bacteria;D_1__Proteobacteria;D_2__Gammaproteobacteria;D_3__Pasteurellales;D_4__Pasteurellaceae;D_5__Haemophilus
D_0__Bacteria;D_1__Proteobacteria;D_2__Gammaproteobacteria;D_3__Pasteurellales;D_4__Pasteurellaceae;Other
D_0__Bacteria;D_1__Proteobacteria;D_2__Gammaproteobacteria;D_3__Pseudomonadales;D_4__Moraxellaceae;D_5__Acinetobacter
D_0__Bacteria;D_1__Proteobacteria;D_2__Gammaproteobacteria;D_3__Pseudomonadales;D_4__Moraxellaceae;D_5__Enhydrobacter
D_0__Bacteria;D_1__Proteobacteria;D_2__Gammaproteobacteria;D_3__Pseudomonadales;D_4__Moraxellaceae;D_5__Moraxella
D_0__Bacteria;D_1__Proteobacteria;D_2__Gammaproteobacteria;D_3__Pseudomonadales;D_4__Moraxellaceae;D_5__Psychrobacter
D_0__Bacteria;D_1__Proteobacteria;D_2__Gammaproteobacteria;D_3__Pseudomonadales;D_4__Moraxellaceae;D_5__uncultured
D_0__Bacteria;D_1__Proteobacteria;D_2__Gammaproteobacteria;D_3__Pseudomonadales;D_4__Pseudomonadaceae;D_5__Pseudomonas
D_0__Bacteria;D_1__Proteobacteria;D_2__Gammaproteobacteria;D_3__Thiotrichales;D_4__Piscirickettsiaceae;D_5__uncultured bacterium
D_0__Bacteria;D_1__Proteobacteria;D_2__Gammaproteobacteria;D_3__Thiotrichales;D_4__Thiotrichaceae;D_5__uncultured
D_0__Bacteria;D_1__Proteobacteria;D_2__Gammaproteobacteria;D_3__Vibrionales;D_4__Vibrionaceae;D_5__Vibrio
D_0__Bacteria;D_1__Proteobacteria;D_2__Gammaproteobacteria;D_3__X35;Other;Other
D_0__Bacteria;D_1__Proteobacteria;D_2__Gammaproteobacteria;D_3__Xanthomonadales;D_4__JTB255 marine benthic group;Ambiguous_taxa
D_0__Bacteria;D_1__Proteobacteria;D_2__Gammaproteobacteria;D_3__Xanthomonadales;D_4__JTB255 marine benthic group;D_5__uncultured bacterium
D_0__Bacteria;D_1__Proteobacteria;D_2__Gammaproteobacteria;D_3__Xanthomonadales;D_4__JTB255 marine benthic group;Other
D_0__Bacteria;D_1__Proteobacteria;D_2__Gammaproteobacteria;D_3__Xanthomonadales;D_4__Nevskiaceae;D_5__Nevskia
D_0__Bacteria;D_1__Proteobacteria;D_2__Gammaproteobacteria;D_3__Xanthomonadales;D_4__Xanthomonadaceae;D_5__Arenimonas
D_0__Bacteria;D_1__Proteobacteria;D_2__Gammaproteobacteria;D_3__Xanthomonadales;D_4__Xanthomonadaceae;D_5__Luteimonas
D_0__Bacteria;D_1__Proteobacteria;D_2__Gammaproteobacteria;D_3__Xanthomonadales;D_4__Xanthomonadaceae;D_5__Lysobacter
D_0__Bacteria;D_1__Proteobacteria;D_2__Gammaproteobacteria;D_3__Xanthomonadales;D_4__Xanthomonadaceae;D_5__Pseudofulvimonas
D_0__Bacteria;D_1__Proteobacteria;D_2__Gammaproteobacteria;D_3__Xanthomonadales;D_4__Xanthomonadaceae;D_5__Pseudoxanthomonas
D_0__Bacteria;D_1__Proteobacteria;D_2__Gammaproteobacteria;D_3__Xanthomonadales;D_4__Xanthomonadaceae;D_5__Stenotrophomonas
D_0__Bacteria;D_1__Proteobacteria;D_2__Gammaproteobacteria;D_3__Xanthomonadales;D_4__Xanthomonadaceae;D_5__Thermomonas
D_0__Bacteria;D_1__Proteobacteria;D_2__Gammaproteobacteria;D_3__Xanthomonadales;D_4__Xanthomonadaceae;D_5__uncultured
D_0__Bacteria;D_1__Proteobacteria;D_2__Gammaproteobacteria;D_3__Xanthomonadales;D_4__Xanthomonadaceae;Other
D_0__Bacteria;D_1__Proteobacteria;D_2__Gammaproteobacteria;D_3__Xanthomonadales;D_4__Xanthomonadales Incertae Sedis;D_5__Acidibacter
D_0__Bacteria;D_1__Proteobacteria;D_2__Gammaproteobacteria;D_3__Xanthomonadales;D_4__Xanthomonadales Incertae Sedis;D_5__Steroidobacter
D_0__Bacteria;D_1__Proteobacteria;D_2__Gammaproteobacteria;D_3__Xanthomonadales;D_4__uncultured;D_5__uncultured bacterium
D_0__Bacteria;D_1__Proteobacteria;D_2__Gammaproteobacteria;D_3__Xanthomonadales;Other;Other
D_0__Bacteria;D_1__Proteobacteria;D_2__Gammaproteobacteria;D_3__uncultured;D_4__uncultured bacterium;D_5__uncultured bacterium
D_0__Bacteria;D_1__Proteobacteria;D_2__Gammaproteobacteria;D_3__uncultured;Other;Other
D_0__Bacteria;D_1__Proteobacteria;D_2__Gammaproteobacteria;Other;Other;Other
D_0__Bacteria;D_1__Proteobacteria;D_2__JTB23;D_3__uncultured bacterium;D_4__uncultured bacterium;D_5__uncultured bacterium
D_0__Bacteria;D_1__Proteobacteria;D_2__JTB23;Other;Other;Other
D_0__Bacteria;D_1__Proteobacteria;D_2__SPOTSOCT00m83;Ambiguous_taxa;Ambiguous_taxa;Ambiguous_taxa
D_0__Bacteria;D_1__Proteobacteria;D_2__SPOTSOCT00m83;Other;Other;Other
D_0__Bacteria;D_1__Proteobacteria;Other;Other;Other;Other
D_0__Bacteria;D_1__SBR1093;Other;Other;Other;Other
D_0__Bacteria;D_1__SR1 (Absconditabacteria);Ambiguous_taxa;Ambiguous_taxa;Ambiguous_taxa;Ambiguous_taxa
D_0__Bacteria;D_1__SR1 (Absconditabacteria);D_2__uncultured bacterium;D_3__uncultured bacterium;D_4__uncultured bacterium;D_5__uncultured bacterium
D_0__Bacteria;D_1__Saccharibacteria;D_2__uncultured bacterium;D_3__uncultured bacterium;D_4__uncultured bacterium;D_5__uncultured bacterium
D_0__Bacteria;D_1__Saccharibacteria;Other;Other;Other;Other
D_0__Bacteria;D_1__Spirochaetae;D_2__Spirochaetes;D_3__Spirochaetales;D_4__Spirochaetaceae;D_5__Treponema 2
D_0__Bacteria;D_1__Synergistetes;D_2__Synergistia;D_3__Synergistales;D_4__Synergistaceae;D_5__Fretibacterium
D_0__Bacteria;D_1__Synergistetes;D_2__Synergistia;D_3__Synergistales;D_4__Synergistaceae;D_5__uncultured
D_0__Bacteria;D_1__TM6 (Dependentiae);Other;Other;Other;Other
D_0__Bacteria;D_1__Tectomicrobia;D_2__Tectomicrobia Incertae Sedis;D_3__Unknown Order;D_4__Unknown Family;D_5__Candidatus Entotheonella
D_0__Bacteria;D_1__Tectomicrobia;D_2__uncultured bacterium;D_3__uncultured bacterium;D_4__uncultured bacterium;D_5__uncultured bacterium
D_0__Bacteria;D_1__Verrucomicrobia;D_2__OPB35 soil group;Ambiguous_taxa;Ambiguous_taxa;Ambiguous_taxa
D_0__Bacteria;D_1__Verrucomicrobia;D_2__OPB35 soil group;D_3__uncultured bacterium;D_4__uncultured bacterium;D_5__uncultured bacterium
D_0__Bacteria;D_1__Verrucomicrobia;D_2__OPB35 soil group;Other;Other;Other
D_0__Bacteria;D_1__Verrucomicrobia;D_2__Opitutae;D_3__BC-COM435;D_4__uncultured bacterium;D_5__uncultured bacterium
D_0__Bacteria;D_1__Verrucomicrobia;D_2__Opitutae;D_3__Opitutales;D_4__Opitutaceae;D_5__Opitutus
D_0__Bacteria;D_1__Verrucomicrobia;D_2__Spartobacteria;D_3__Chthoniobacterales;D_4__Chthoniobacteraceae;D_5__Chthoniobacter
D_0__Bacteria;D_1__Verrucomicrobia;D_2__Spartobacteria;D_3__Chthoniobacterales;D_4__DA101 soil group;Ambiguous_taxa
D_0__Bacteria;D_1__Verrucomicrobia;D_2__Spartobacteria;D_3__Chthoniobacterales;D_4__DA101 soil group;D_5__uncultured bacterium
D_0__Bacteria;D_1__Verrucomicrobia;D_2__Spartobacteria;D_3__Chthoniobacterales;D_4__Xiphinematobacteraceae;D_5__Candidatus Xiphinematobacter
D_0__Bacteria;D_1__Verrucomicrobia;D_2__Spartobacteria;D_3__Chthoniobacterales;Other;Other
D_0__Bacteria;D_1__Verrucomicrobia;D_2__Verrucomicrobiae;D_3__Verrucomicrobiales;D_4__DEV007;D_5__uncultured bacterium
D_0__Bacteria;D_1__Verrucomicrobia;D_2__Verrucomicrobiae;D_3__Verrucomicrobiales;D_4__Verrucomicrobiaceae;Ambiguous_taxa
D_0__Bacteria;D_1__Verrucomicrobia;D_2__Verrucomicrobiae;D_3__Verrucomicrobiales;D_4__Verrucomicrobiaceae;D_5__Prosthecobacter
D_0__Bacteria;D_1__Verrucomicrobia;D_2__Verrucomicrobiae;D_3__Verrucomicrobiales;D_4__Verrucomicrobiaceae;D_5__uncultured
D_0__Bacteria;D_1__Verrucomicrobia;D_2__Verrucomicrobiae;D_3__Verrucomicrobiales;D_4__Verrucomicrobiaceae;Other
D_0__Bacteria;Other;Other;Other;Other;Other
Unassigned;Other;Other;Other;Other;Other
