# Supplementary material for: Cretaceous dinosaur bone contains recent organic material and provides an environment conducive to microbial communities
Source: eLife. 2019 Jun 18;8:e46205. doi: 10.7554/eLife.46205 (PMC6581507; doi:10.7554/eLife.46205)

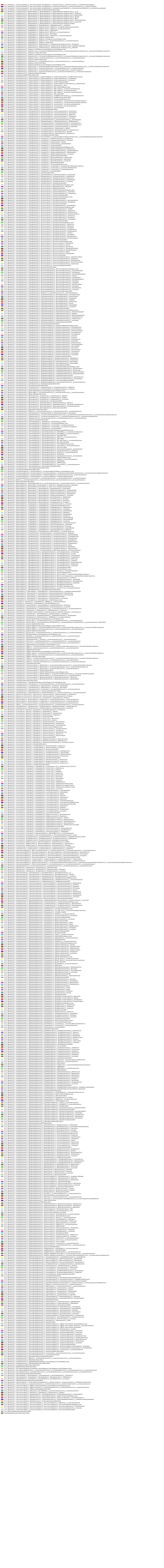

Supplement: Source data 1. [file wawZLqFWDUTKKmJZF0o01JIZ1F95ze_legend.pdf › Raw data files/16S rRNA amplicon sequencing/taxa_plots/taxa_summary_plots/charts/wawZLqFWDUTKKmJZF0o01JIZ1F95ze_legend.pdf]
